# Supplementary material for: Applying a Dynamical Systems Model and Network Theory to Major Depressive Disorder
Source: Front Psychol. 2019 Aug 7;10:1762. doi: 10.3389/fpsyg.2019.01762 (PMC6692450; doi:10.3389/fpsyg.2019.01762)
Supplement: Supplementary file 1 [file Table_1.pdf]

Supplementary Material

1 SUPPLEMENTARY TABLE S1

Structure	T	N	p	p_e/p_w	$\overline{\hat{p}}$ (SD)	$\overline{\hat{p_e}}/\overline{\hat{p_w}}$ (SD)	$\overline{\lvert\Delta\rvert}_p$ (SD)	Range $\lvert\Delta\rvert_p$	$\overline{SE}_p$ (SD)	$\overline{BIC}$ (SD)	$\overline{\lvert\Delta\rvert}_{p_e/p_w}$ (SD)	Range $\lvert\Delta\rvert_{p_e/p_w}$
grid	50	16	0.10	0.10	0.094 (0.011)		0.01 (0.008)	0 - 0.034	0.002 (0)	-158.79 (12.88)		
grid	50	16	0.20	0.10	0.174 (0.016)		0.027 (0.014)	0.001 - 0.071	0.003 (0)	-192.82 (11.15)		
grid	50	16	0.30	0.10	0.269 (0.027)		0.035 (0.02)	0.002 - 0.098	0.004 (0)	-205.64 (11.42)		
grid	50	16	0.40	0.10	0.394 (0.04)		0.033 (0.024)	0.001 - 0.117	0.005 (0)	-206.56 (10.18)		
grid	50	16	0.50	0.10	0.503 (0.041)		0.033 (0.024)	0.002 - 0.108	0.006 (0)	-200.72 (9.43)		
grid	50	16	0.60	0.10	0.579 (0.043)		0.039 (0.028)	0 - 0.121	0.006 (0)	-204.38 (10.02)		
grid	50	16	0.70	0.10	0.654 (0.042)		0.051 (0.036)	0.001 - 0.162	0.005 (0.001)	-208.18 (10.96)		
grid	50	16	0.80	0.10	0.744 (0.038)		0.056 (0.037)	0.001 - 0.197	0.004 (0.001)	-207.76 (12.04)		
grid	50	16	0.90	0.10	0.868 (0.051)		0.037 (0.048)	0 - 0.246	0.002 (0.001)	-174.5 (19.06)		
grid	50	25	0.10	0.10	0.096 (0.008)		0.008 (0.005)	0 - 0.019	0.001 (0)	-181.71 (12.99)		
grid	50	25	0.20	0.10	0.174 (0.012)		0.026 (0.012)	0 - 0.055	0.002 (0)	-214.36 (11.42)		
grid	50	25	0.30	0.10	0.26 (0.021)		0.04 (0.02)	0 - 0.079	0.003 (0)	-228.16 (11.34)		
grid	50	25	0.40	0.10	0.382 (0.039)		0.035 (0.024)	0 - 0.104	0.005 (0)	-229.12 (10.13)		
grid	50	25	0.50	0.10	0.5 (0.036)		0.03 (0.02)	0.001 - 0.081	0.005 (0)	-223.5 (9.21)		
grid	50	25	0.60	0.10	0.562 (0.034)		0.041 (0.03)	0 - 0.146	0.005 (0)	-228.62 (10.39)		
grid	50	25	0.70	0.10	0.636 (0.038)		0.064 (0.038)	0.001 - 0.168	0.004 (0)	-238.34 (9.89)		
grid	50	25	0.80	0.10	0.729 (0.043)		0.071 (0.043)	0 - 0.195	0.003 (0.001)	-245.47 (11.14)		
grid	50	25	0.90	0.10	0.876 (0.023)		0.025 (0.022)	0 - 0.099	0.002 (0)	-205.57 (21.15)		
grid	50	49	0.10	0.10	0.098 (0.007)		0.006 (0.004)	0 - 0.021	0.001 (0)	-223.13 (16.64)		
grid	50	49	0.20	0.10	0.177 (0.008)		0.023 (0.008)	0.005 - 0.041	0.001 (0)	-253.62 (15.29)		
grid	50	49	0.30	0.10	0.251 (0.013)		0.049 (0.013)	0.005 - 0.076	0.003 (0)	-263.82 (11.86)		
grid	50	49	0.40	0.10	0.368 (0.032)		0.037 (0.026)	0 - 0.109	0.004 (0)	-264.17 (10.16)		
grid	50	49	0.50	0.10	0.5 (0.034)		0.027 (0.021)	0 - 0.092	0.005 (0)	-257.18 (9.77)		
grid	50	49	0.60	0.10	0.551 (0.037)		0.053 (0.031)	0.003 - 0.138	0.005 (0)	-266.59 (10.31)		
grid	50	49	0.70	0.10	0.611 (0.041)		0.089 (0.041)	0.004 - 0.194	0.004 (0)	-289.5 (12.45)		
grid	50	49	0.80	0.10	0.7 (0.036)		0.1 (0.036)	0.018 - 0.192	0.003 (0)	-308.54 (15.66)		
grid	50	49	0.90	0.10	0.865 (0.024)		0.035 (0.024)	0.001 - 0.11	0.001 (0)	-253.99 (28.03)		
grid	50	100	0.10	0.10	0.095 (0.005)		0.006 (0.004)	0 - 0.017	0.001 (0)	-255.87 (13.26)		
grid	50	100	0.20	0.10	0.171 (0.005)		0.029 (0.005)	0.019 - 0.041	0.001 (0)	-285.89 (13.97)		
grid	50	100	0.30	0.10	0.23 (0.007)		0.07 (0.007)	0.053 - 0.082	0.002 (0)	-302.66 (12.47)		
grid	50	100	0.40	0.10	0.335 (0.028)		0.066 (0.027)	0.004 - 0.118	0.004 (0)	-305.92 (12.18)		

Continued on next page

Table S1 – *Continued from previous page*

Structure	T	N	p	p_e/p_w	$\overline{\hat{p}}$ (SD)	$\overline{\hat{p_e}}/\overline{\hat{p_w}}$ (SD)	$\overline{\lvert\Delta\rvert}_p$ (SD)	Range $\lvert\Delta\rvert_p$	$\overline{SE}\lvert\Delta\rvert_p$ (SD)	$\overline{BIC}$ (SD)	$\overline{\lvert\Delta\rvert}_{p_e/p_w}$ (SD)	Range $\lvert\Delta\rvert_{p_e/p_w}$
grid	50	100	0.50	0.10	0.498 (0.039)		0.032 (0.022)	0 - 0.093	0.005 (0.001)	-292.74 (9.41)		
grid	50	100	0.60	0.10	0.497 (0.046)		0.103 (0.046)	0.002 - 0.219	0.005 (0)	-311.13 (13.39)		
grid	50	100	0.70	0.10	0.527 (0.048)		0.173 (0.048)	0.063 - 0.31	0.004 (0)	-346.48 (18.19)		
grid	50	100	0.80	0.10	0.595 (0.069)		0.205 (0.069)	0.068 - 0.398	0.003 (0)	-380.67 (23.78)		
grid	50	100	0.90	0.10	0.764 (0.097)		0.136 (0.097)	0.016 - 0.478	0.002 (0.001)	-346.48 (29.04)		
grid	100	16	0.10	0.10	0.093 (0.007)		0.008 (0.006)	0 - 0.023	0.001 (0)	-318.35 (16.47)		
grid	100	16	0.20	0.10	0.171 (0.009)		0.029 (0.009)	0.004 - 0.051	0.001 (0)	-386.42 (15.85)		
grid	100	16	0.30	0.10	0.265 (0.021)		0.036 (0.018)	0 - 0.068	0.002 (0)	-416.78 (16.11)		
grid	100	16	0.40	0.10	0.389 (0.032)		0.027 (0.02)	0.001 - 0.086	0.003 (0)	-417.68 (14.04)		
grid	100	16	0.50	0.10	0.504 (0.028)		0.023 (0.016)	0.001 - 0.063	0.003 (0)	-411.55 (13.39)		
grid	100	16	0.60	0.10	0.577 (0.028)		0.029 (0.022)	0 - 0.09	0.003 (0)	-420 (13.44)		
grid	100	16	0.70	0.10	0.65 (0.028)		0.05 (0.027)	0.001 - 0.117	0.002 (0)	-427.97 (12.81)		
grid	100	16	0.80	0.10	0.749 (0.031)		0.052 (0.031)	0 - 0.137	0.002 (0)	-426.3 (15.67)		
grid	100	16	0.90	0.10	0.867 (0.046)		0.035 (0.045)	0 - 0.223	0.001 (0.001)	-368.7 (24.91)		
grid	100	25	0.10	0.10	0.094 (0.006)		0.007 (0.005)	0 - 0.022	0.001 (0)	-366.06 (17.79)		
grid	100	25	0.20	0.10	0.172 (0.007)		0.028 (0.007)	0.007 - 0.043	0.001 (0)	-433.85 (18.12)		
grid	100	25	0.30	0.10	0.258 (0.015)		0.043 (0.014)	0.002 - 0.073	0.002 (0)	-465.57 (16.79)		
grid	100	25	0.40	0.10	0.383 (0.026)		0.025 (0.019)	0 - 0.104	0.002 (0)	-463.78 (14.33)		
grid	100	25	0.50	0.10	0.501 (0.024)		0.02 (0.013)	0.001 - 0.058	0.003 (0)	-458.05 (12.53)		
grid	100	25	0.60	0.10	0.566 (0.032)		0.038 (0.027)	0.002 - 0.13	0.003 (0)	-466.09 (16.35)		
grid	100	25	0.70	0.10	0.628 (0.032)		0.072 (0.031)	0.003 - 0.154	0.002 (0)	-485.32 (17.06)		
grid	100	25	0.80	0.10	0.727 (0.03)		0.073 (0.03)	0.001 - 0.158	0.002 (0)	-500.77 (14.92)		
grid	100	25	0.90	0.10	0.877 (0.016)		0.024 (0.016)	0 - 0.078	0.001 (0)	-425.7 (33.3)		
grid	100	49	0.10	0.10	0.096 (0.004)		0.005 (0.003)	0 - 0.014	0 (0)	-442.91 (18.51)		
grid	100	49	0.20	0.10	0.173 (0.004)		0.027 (0.004)	0.016 - 0.038	0.001 (0)	-503.32 (16.62)		
grid	100	49	0.30	0.10	0.242 (0.01)		0.058 (0.01)	0.037 - 0.077	0.001 (0)	-537.93 (20.05)		
grid	100	49	0.40	0.10	0.363 (0.023)		0.038 (0.021)	0.002 - 0.096	0.002 (0)	-540.12 (16.63)		
grid	100	49	0.50	0.10	0.503 (0.027)		0.022 (0.015)	0 - 0.058	0.003 (0)	-524.52 (15.06)		
grid	100	49	0.60	0.10	0.543 (0.029)		0.057 (0.028)	0.001 - 0.119	0.002 (0)	-546.08 (17.26)		
grid	100	49	0.70	0.10	0.596 (0.03)		0.104 (0.03)	0.035 - 0.175	0.002 (0)	-587.03 (18.43)		
grid	100	49	0.80	0.10	0.685 (0.034)		0.115 (0.034)	0.047 - 0.215	0.002 (0)	-629.15 (21.89)		

Continued on next page

Table S1 – *Continued from previous page*

Structure	T	N	p	p_e/p_w	$\overline{\hat{p}}$ (SD)	$\overline{\hat{p}_e}/\overline{\hat{p}_w}$ (SD)	$\overline{\lvert\Delta\rvert}_p$ (SD)	Range $\lvert\Delta\rvert_p$	$\overline{SE}\lvert\Delta\rvert_p$ (SD)	$\overline{BIC}$ (SD)	$\overline{\lvert\Delta\rvert}_{p_e/p_w}$ (SD)	Range $\lvert\Delta\rvert_{p_e/p_w}$
grid	100	49	0.90	0.10	0.863 (0.017)		0.037 (0.017)	0.003 - 0.085	0.001 (0)	-533.9 (37.55)		
grid	100	100	0.10	0.10	0.095 (0.003)		0.005 (0.003)	0 - 0.014	0 (0)	-506 (17.67)		
grid	100	100	0.20	0.10	0.172 (0.004)		0.028 (0.004)	0.019 - 0.037	0.001 (0)	-571.46 (17.62)		
grid	100	100	0.30	0.10	0.23 (0.004)		0.07 (0.004)	0.058 - 0.079	0.001 (0)	-613.91 (17.83)		
grid	100	100	0.40	0.10	0.328 (0.016)		0.072 (0.016)	0.033 - 0.108	0.002 (0)	-621.03 (16.15)		
grid	100	100	0.50	0.10	0.504 (0.027)		0.022 (0.016)	0 - 0.079	0.003 (0)	-594.39 (14.29)		
grid	100	100	0.60	0.10	0.504 (0.034)		0.096 (0.034)	0.018 - 0.177	0.002 (0)	-632.41 (18.6)		
grid	100	100	0.70	0.10	0.522 (0.037)		0.178 (0.037)	0.102 - 0.295	0.002 (0)	-706.61 (26.16)		
grid	100	100	0.80	0.10	0.607 (0.041)		0.193 (0.041)	0.09 - 0.3	0.002 (0)	-791.92 (34.73)		
grid	100	100	0.90	0.10	0.786 (0.081)		0.114 (0.081)	0.025 - 0.37	0.001 (0.001)	-697.36 (44.45)		
grid	200	16	0.10	0.10	0.091 (0.005)		0.009 (0.004)	0 - 0.019	0 (0)	-635.61 (23.96)		
grid	200	16	0.20	0.10	0.17 (0.007)		0.03 (0.007)	0.012 - 0.052	0.001 (0)	-784.02 (23.79)		
grid	200	16	0.30	0.10	0.262 (0.012)		0.038 (0.012)	0.004 - 0.064	0.001 (0)	-842.85 (22.17)		
grid	200	16	0.40	0.10	0.39 (0.02)		0.019 (0.012)	0 - 0.049	0.001 (0)	-845.55 (17.38)		
grid	200	16	0.50	0.10	0.5 (0.021)		0.018 (0.011)	0 - 0.053	0.001 (0)	-835.87 (18.47)		
grid	200	16	0.60	0.10	0.575 (0.022)		0.028 (0.018)	0 - 0.08	0.001 (0)	-847.59 (20.98)		
grid	200	16	0.70	0.10	0.651 (0.022)		0.049 (0.022)	0 - 0.103	0.001 (0)	-867.16 (23.11)		
grid	200	16	0.80	0.10	0.745 (0.023)		0.055 (0.023)	0 - 0.112	0.001 (0)	-862.41 (22.61)		
grid	200	16	0.90	0.10	0.869 (0.027)		0.031 (0.026)	0.002 - 0.152	0.001 (0)	-757.95 (35.4)		
grid	200	25	0.10	0.10	0.092 (0.004)		0.008 (0.004)	0.001 - 0.019	0 (0)	-729.09 (21.28)		
grid	200	25	0.20	0.10	0.171 (0.006)		0.029 (0.006)	0.017 - 0.045	0 (0)	-873.45 (24.97)		
grid	200	25	0.30	0.10	0.253 (0.01)		0.047 (0.01)	0.025 - 0.066	0.001 (0)	-934.27 (22.32)		
grid	200	25	0.40	0.10	0.379 (0.018)		0.023 (0.015)	0.001 - 0.067	0.001 (0)	-942.7 (22.49)		
grid	200	25	0.50	0.10	0.497 (0.02)		0.017 (0.012)	0.001 - 0.05	0.001 (0)	-921.49 (17.19)		
grid	200	25	0.60	0.10	0.565 (0.018)		0.035 (0.018)	0.001 - 0.09	0.001 (0)	-941.78 (20.21)		
grid	200	25	0.70	0.10	0.628 (0.021)		0.072 (0.021)	0.028 - 0.135	0.001 (0)	-982.95 (23.4)		
grid	200	25	0.80	0.10	0.727 (0.024)		0.073 (0.024)	0.026 - 0.131	0.001 (0)	-1013.62 (25.73)		
grid	200	25	0.90	0.10	0.879 (0.01)		0.021 (0.01)	0.001 - 0.042	0 (0)	-858.14 (49.44)		
grid	200	49	0.10	0.10	0.094 (0.003)		0.006 (0.003)	0.001 - 0.015	0 (0)	-874.27 (23.57)		
grid	200	49	0.20	0.10	0.172 (0.004)		0.028 (0.004)	0.02 - 0.036	0 (0)	-1011.12 (25.16)		
grid	200	49	0.30	0.10	0.24 (0.006)		0.06 (0.006)	0.043 - 0.071	0.001 (0)	-1080.73 (25.67)		
grid	200	49	0.40	0.10	0.36 (0.019)		0.041 (0.018)	0.001 - 0.079	0.001 (0)	-1086.79 (21.6)		
grid	200	49	0.50	0.10	0.499 (0.02)		0.016 (0.012)	0 - 0.059	0.001 (0)	-1055.9 (19.45)		

Continued on next page

Table S1 – *Continued from previous page*

Structure	T	N	p	p_e/p_w	$\overline{\hat{p}}$ (SD)	$\overline{\hat{p_e}}/\overline{\hat{p_w}}$ (SD)	$\overline{\lvert\Delta\rvert}_p$ (SD)	Range $\lvert\Delta\rvert_p$	$\overline{SE}\lvert\Delta\rvert_p$ (SD)	$\overline{BIC}$ (SD)	$\overline{\lvert\Delta\rvert}_{p_e/p_w}$ (SD)	Range $\lvert\Delta\rvert_{p_e/p_w}$
grid	200	49	0.60	0.10	0.546 (0.023)		0.054 (0.022)	0.011 - 0.112	0.001 (0)	-1098.29 (25.35)		
grid	200	49	0.70	0.10	0.588 (0.024)		0.112 (0.024)	0.056 - 0.16	0.001 (0)	-1180.26 (27.18)		
grid	200	49	0.80	0.10	0.684 (0.026)		0.116 (0.026)	0.054 - 0.183	0.001 (0)	-1267.53 (29.48)		
grid	200	49	0.90	0.10	0.861 (0.015)		0.039 (0.015)	0.011 - 0.099	0 (0)	-1084.98 (66.69)		
grid	200	100	0.10	0.10	0.095 (0.002)		0.005 (0.002)	0 - 0.011	0 (0)	-1016.01 (20.12)		
grid	200	100	0.20	0.10	0.172 (0.003)		0.028 (0.003)	0.021 - 0.033	0 (0)	-1156.02 (21.15)		
grid	200	100	0.30	0.10	0.229 (0.003)		0.071 (0.003)	0.062 - 0.078	0 (0)	-1231.86 (24.45)		
grid	200	100	0.40	0.10	0.329 (0.014)		0.071 (0.014)	0.017 - 0.092	0.001 (0)	-1252.01 (27.32)		
grid	200	100	0.50	0.10	0.501 (0.019)		0.016 (0.012)	0 - 0.049	0.001 (0)	-1196.21 (18.52)		
grid	200	100	0.60	0.10	0.506 (0.026)		0.094 (0.026)	0.025 - 0.159	0.001 (0)	-1279.88 (26.08)		
grid	200	100	0.70	0.10	0.519 (0.024)		0.181 (0.024)	0.119 - 0.235	0.001 (0)	-1419.67 (35.22)		
grid	200	100	0.80	0.10	0.614 (0.032)		0.186 (0.032)	0.116 - 0.272	0.001 (0)	-1593.42 (52.25)		
grid	200	100	0.90	0.10	0.797 (0.058)		0.103 (0.058)	0.037 - 0.282	0 (0)	-1422.52 (84.69)		
grid	500	16	0.10	0.10	0.09 (0.003)		0.01 (0.003)	0.004 - 0.017	0 (0)	-1587.13 (33.79)		
grid	500	16	0.20	0.10	0.169 (0.004)		0.031 (0.004)	0.022 - 0.043	0 (0)	-1971.29 (31.32)		
grid	500	16	0.30	0.10	0.26 (0.01)		0.04 (0.01)	0.017 - 0.059	0 (0)	-2126.22 (36.5)		
grid	500	16	0.40	0.10	0.389 (0.013)		0.014 (0.01)	0 - 0.046	0.001 (0)	-2128.59 (33.79)		
grid	500	16	0.50	0.10	0.5 (0.014)		0.011 (0.008)	0 - 0.038	0.001 (0)	-2102.19 (29.45)		
grid	500	16	0.60	0.10	0.575 (0.012)		0.025 (0.012)	0 - 0.058	0.001 (0)	-2135.1 (29.47)		
grid	500	16	0.70	0.10	0.65 (0.015)		0.05 (0.015)	0.011 - 0.088	0 (0)	-2185.75 (38.57)		
grid	500	16	0.80	0.10	0.747 (0.014)		0.053 (0.014)	0.024 - 0.084	0 (0)	-2163.58 (32.37)		
grid	500	16	0.90	0.10	0.872 (0.018)		0.028 (0.018)	0.001 - 0.101	0 (0)	-1924.91 (71.3)		
grid	500	25	0.10	0.10	0.092 (0.003)		0.008 (0.003)	0.002 - 0.015	0 (0)	-1823.67 (34.43)		
grid	500	25	0.20	0.10	0.169 (0.003)		0.031 (0.003)	0.023 - 0.043	0 (0)	-2191.68 (38.11)		
grid	500	25	0.30	0.10	0.251 (0.006)		0.049 (0.006)	0.032 - 0.067	0 (0)	-2359.17 (36.27)		
grid	500	25	0.40	0.10	0.383 (0.012)		0.018 (0.01)	0 - 0.047	0.001 (0)	-2370.1 (32.39)		
grid	500	25	0.50	0.10	0.501 (0.015)		0.012 (0.009)	0 - 0.038	0.001 (0)	-2320.44 (31.19)		
grid	500	25	0.60	0.10	0.562 (0.013)		0.038 (0.013)	0.014 - 0.079	0.001 (0)	-2373.85 (35.45)		
grid	500	25	0.70	0.10	0.625 (0.014)		0.075 (0.014)	0.044 - 0.11	0 (0)	-2475.23 (31.65)		
grid	500	25	0.80	0.10	0.722 (0.014)		0.078 (0.014)	0.047 - 0.112	0 (0)	-2553.44 (43.46)		
grid	500	25	0.90	0.10	0.876 (0.007)		0.024 (0.007)	0.005 - 0.037	0 (0)	-2197.46 (75.3)		
grid	500	49	0.10	0.10	0.093 (0.002)		0.007 (0.002)	0.003 - 0.011	0 (0)	-2171.76 (35.54)		
grid	500	49	0.20	0.10	0.171 (0.002)		0.029 (0.002)	0.023 - 0.035	0 (0)	-2532.39 (30.48)		
grid	500	49	0.30	0.10	0.239 (0.004)		0.061 (0.004)	0.048 - 0.068	0 (0)	-2719.95 (40.13)		

Continued on next page

Table S1 – *Continued from previous page*

Structure	T	N	p	p_e/p_w	$\overline{\hat{p}}$ (SD)	$\overline{\hat{p}_e}/\overline{\hat{p}_w}$ (SD)	$\overline{\lvert\Delta\rvert}_p$ (SD)	Range $\lvert\Delta\rvert_p$	$\overline{SE}\lvert\Delta\rvert_p$ (SD)	$\overline{BIC}$ (SD)	$\overline{\lvert\Delta\rvert}_{p_e/p_w}$ (SD)	Range $\lvert\Delta\rvert_{p_e/p_w}$
grid	500	49	0.40	0.10	0.357 (0.01)		0.043 (0.01)	0.017 - 0.065	0 (0)	-2734.02 (36.71)		
grid	500	49	0.50	0.10	0.498 (0.011)		0.009 (0.006)	0 - 0.027	0.001 (0)	-2660.76 (28.65)		
grid	500	49	0.60	0.10	0.54 (0.014)		0.06 (0.014)	0.025 - 0.101	0 (0)	-2761.39 (34.62)		
grid	500	49	0.70	0.10	0.584 (0.015)		0.116 (0.015)	0.077 - 0.154	0 (0)	-2974.9 (44.3)		
grid	500	49	0.80	0.10	0.677 (0.015)		0.123 (0.015)	0.086 - 0.165	0 (0)	-3178.48 (47)		
grid	500	49	0.90	0.10	0.86 (0.01)		0.04 (0.01)	0.021 - 0.067	0 (0)	-2793.67 (116.95)		
grid	500	100	0.10	0.10	0.095 (0.001)		0.005 (0.001)	0.002 - 0.008	0 (0)	-2536.1 (29.93)		
grid	500	100	0.20	0.10	0.173 (0.001)		0.027 (0.001)	0.024 - 0.031	0 (0)	-2892.27 (39.77)		
grid	500	100	0.30	0.10	0.229 (0.002)		0.071 (0.002)	0.065 - 0.075	0 (0)	-3081.39 (42.9)		
grid	500	100	0.40	0.10	0.325 (0.008)		0.075 (0.008)	0.055 - 0.096	0 (0)	-3141.36 (36.84)		
grid	500	100	0.50	0.10	0.5 (0.012)		0.01 (0.007)	0 - 0.027	0.001 (0)	-3011.44 (34.51)		
grid	500	100	0.60	0.10	0.503 (0.013)		0.097 (0.013)	0.057 - 0.129	0 (0)	-3211.84 (38.68)		
grid	500	100	0.70	0.10	0.518 (0.015)		0.182 (0.015)	0.146 - 0.22	0 (0)	-3585.15 (61.68)		
grid	500	100	0.80	0.10	0.608 (0.021)		0.192 (0.021)	0.145 - 0.24	0 (0)	-4004.74 (78.89)		
grid	500	100	0.90	0.10	0.804 (0.051)		0.096 (0.051)	0.04 - 0.235	0 (0)	-3564.77 (150.61)		
grid	5000	16	0.10	0.10	0.089 (0.001)		0.011 (0.001)	0.009 - 0.014	0 (0)	-15861.59 (108.66)		
grid	5000	16	0.20	0.10	0.169 (0.001)		0.031 (0.001)	0.028 - 0.034	0 (0)	-19805.81 (122.45)		
grid	5000	16	0.30	0.10	0.26 (0.002)		0.04 (0.002)	0.034 - 0.046	0 (0)	-21339.93 (114.16)		
grid	5000	16	0.40	0.10	0.388 (0.004)		0.012 (0.004)	0.001 - 0.02	0 (0)	-21358.8 (100.23)		
grid	5000	16	0.50	0.10	0.5 (0.004)		0.003 (0.002)	0 - 0.01	0 (0)	-21103.75 (97.05)		
grid	5000	16	0.60	0.10	0.576 (0.004)		0.024 (0.004)	0.013 - 0.032	0 (0)	-21395.23 (93.97)		
grid	5000	16	0.70	0.10	0.651 (0.005)		0.049 (0.005)	0.035 - 0.059	0 (0)	-21932.22 (101.13)		
grid	5000	16	0.80	0.10	0.746 (0.006)		0.054 (0.006)	0.043 - 0.073	0 (0)	-21865.49 (118.79)		
grid	5000	16	0.90	0.10	0.871 (0.006)		0.029 (0.006)	0.014 - 0.045	0 (0)	-19566.57 (220.07)		
grid	5000	25	0.10	0.10	0.091 (0.001)		0.009 (0.001)	0.007 - 0.011	0 (0)	-18240.9 (106.09)		
grid	5000	25	0.20	0.10	0.169 (0.001)		0.031 (0.001)	0.029 - 0.034	0 (0)	-21982.21 (127.58)		
grid	5000	25	0.30	0.10	0.25 (0.002)		0.05 (0.002)	0.046 - 0.055	0 (0)	-23709.33 (108.83)		
grid	5000	25	0.40	0.10	0.378 (0.004)		0.022 (0.004)	0.012 - 0.035	0 (0)	-23763.27 (109.96)		
grid	5000	25	0.50	0.10	0.499 (0.004)		0.003 (0.003)	0 - 0.014	0 (0)	-23352.26 (98.24)		
grid	5000	25	0.60	0.10	0.564 (0.004)		0.036 (0.004)	0.026 - 0.044	0 (0)	-23868.41 (106.73)		
grid	5000	25	0.70	0.10	0.626 (0.004)		0.074 (0.004)	0.065 - 0.088	0 (0)	-24902.03 (101.36)		
grid	5000	25	0.80	0.10	0.723 (0.005)		0.077 (0.005)	0.061 - 0.087	0 (0)	-25685.47 (116.83)		
grid	5000	25	0.90	0.10	0.878 (0.002)		0.022 (0.002)	0.017 - 0.028	0 (0)	-21917.47 (264.92)		
grid	5000	49	0.10	0.10	0.093 (0.001)		0.007 (0.001)	0.005 - 0.008	0 (0)	-21757.55 (110.77)		

Continued on next page

Table S1 – *Continued from previous page*

Structure	T	N	p	p_e/p_w	$\overline{\hat{p}}$ (SD)	$\overline{\hat{p_e}}/\overline{\hat{p_w}}$ (SD)	$\overline{\lvert\Delta\rvert}_p$ (SD)	Range $\lvert\Delta\rvert_p$	$\overline{SE}\lvert\Delta\rvert_p$ (SD)	$\overline{BIC}$ (SD)	$\overline{\lvert\Delta\rvert}_{p_e/p_w}$ (SD)	Range $\lvert\Delta\rvert_{p_e/p_w}$
grid	5000	49	0.20	0.10	0.171 (0.001)		0.029 (0.001)	0.027 - 0.03	0 (0)	-25383.15 (116.97)		
grid	5000	49	0.30	0.10	0.238 (0.001)		0.062 (0.001)	0.059 - 0.065	0 (0)	-27265.17 (130.5)		
grid	5000	49	0.40	0.10	0.356 (0.003)		0.044 (0.003)	0.033 - 0.053	0 (0)	-27488.86 (104.13)		
grid	5000	49	0.50	0.10	0.5 (0.003)		0.003 (0.002)	0 - 0.01	0 (0)	-26707.99 (103.14)		
grid	5000	49	0.60	0.10	0.54 (0.005)		0.06 (0.005)	0.049 - 0.069	0 (0)	-27765.85 (121.39)		
grid	5000	49	0.70	0.10	0.582 (0.005)		0.118 (0.005)	0.107 - 0.129	0 (0)	-29883.79 (130.95)		
grid	5000	49	0.80	0.10	0.676 (0.005)		0.124 (0.005)	0.111 - 0.137	0 (0)	-31944.75 (155.19)		
grid	5000	49	0.90	0.10	0.86 (0.003)		0.04 (0.003)	0.034 - 0.049	0 (0)	-28320.8 (379.23)		
grid	5000	100	0.10	0.10	0.094 (0)		0.006 (0)	0.004 - 0.006	0 (0)	-25377.67 (98.76)		
grid	5000	100	0.20	0.10	0.172 (0)		0.028 (0)	0.026 - 0.028	0 (0)	-28952.31 (114.56)		
grid	5000	100	0.30	0.10	0.229 (0.001)		0.071 (0.001)	0.069 - 0.072	0 (0)	-30929.01 (140.39)		
grid	5000	100	0.40	0.10	0.324 (0.003)		0.076 (0.003)	0.068 - 0.082	0 (0)	-31543.95 (117.31)		
grid	5000	100	0.50	0.10	0.5 (0.004)		0.003 (0.002)	0 - 0.011	0 (0)	-30258.93 (95.89)		
grid	5000	100	0.60	0.10	0.503 (0.004)		0.097 (0.004)	0.087 - 0.105	0 (0)	-32229.79 (134.38)		
grid	5000	100	0.70	0.10	0.517 (0.005)		0.183 (0.005)	0.17 - 0.197	0 (0)	-35903.63 (195.9)		
grid	5000	100	0.80	0.10	0.611 (0.007)		0.189 (0.007)	0.172 - 0.205	0 (0)	-40260.39 (274.19)		
grid	5000	100	0.90	0.10	0.83 (0.008)		0.07 (0.008)	0.056 - 0.098	0 (0)	-36440.91 (342.13)		
RG	50	16	0.10	0.10	0.122 (0.014)	0.794 (0.034)	0.023 (0.013)	0.001 - 0.061	0.002 (0)	-157.6 (13.58)	0.694 (0.034)	0.571 - 0.816
RG	50	16	0.10	0.20	0.131 (0.017)	0.765 (0.055)	0.031 (0.016)	0.002 - 0.068	0.002 (0)	-160.1 (13.64)	0.565 (0.055)	0.344 - 0.701
RG	50	16	0.10	0.30	0.108 (0.014)	0.813 (0.054)	0.013 (0.009)	0 - 0.037	0.002 (0)	-153.62 (17.86)	0.513 (0.054)	0.388 - 0.679
RG	50	16	0.10	0.40	0.108 (0.014)	0.789 (0.085)	0.012 (0.011)	0 - 0.051	0.002 (0)	-161.07 (25.38)	0.389 (0.085)	0.111 - 0.593
RG	50	16	0.10	0.50	0.094 (0.022)	0.784 (0.094)	0.015 (0.017)	0 - 0.075	0.002 (0)	-163.11 (25.41)	0.284 (0.094)	0.04 - 0.479
RG	50	16	0.10	0.60	0.087 (0.027)	0.774 (0.089)	0.02 (0.023)	0 - 0.075	0.001 (0)	-162.64 (25.19)	0.174 (0.089)	0.068 - 0.393
RG	50	16	0.10	0.70	0.087 (0.024)	0.754 (0.092)	0.018 (0.02)	0.001 - 0.075	0.001 (0)	-165.78 (24.88)	0.069 (0.082)	0 - 0.331
RG	50	16	0.10	0.80	0.092 (0.02)	0.765 (0.085)	0.016 (0.015)	0 - 0.075	0.002 (0)	-162.96 (27.48)	0.08 (0.044)	0 - 0.166
RG	50	16	0.10	0.90	0.087 (0.024)	0.762 (0.081)	0.019 (0.019)	0 - 0.075	0.001 (0)	-165.98 (27.33)	0.142 (0.073)	0.002 - 0.212

Continued on next page

Table S1 – *Continued from previous page*

Structure	T	N	p	p_e/p_w	$\overline{\hat{p}}$ (SD)	$\overline{\hat{p_e}}/\overline{\hat{p_w}}$ (SD)	$\overline{\lvert\Delta\rvert}_p$ (SD)	Range $\lvert\Delta\rvert_p$	$\overline{SE}\lvert\Delta\rvert_p$ (SD)	$\overline{BIC}$ (SD)	$\overline{\lvert\Delta\rvert}_{p_e/p_w}$ (SD)	Range $\lvert\Delta\rvert_{p_e/p_w}$
RG	50	16	0.20	0.10	0.23 (0.018)	0.721 (0.058)	0.031 (0.016)	0.002 - 0.07	0.002 (0)	-180.13 (10.02)	0.621 (0.058)	0.451 - 0.876
RG	50	16	0.20	0.20	0.245 (0.019)	0.68 (0.064)	0.045 (0.019)	0.006 - 0.094	0.002 (0)	-181.63 (8.96)	0.48 (0.064)	0.316 - 0.612
RG	50	16	0.20	0.30	0.211 (0.016)	0.725 (0.063)	0.016 (0.011)	0.001 - 0.053	0.002 (0)	-176.4 (8.72)	0.425 (0.063)	0.259 - 0.737
RG	50	16	0.20	0.40	0.21 (0.016)	0.71 (0.079)	0.016 (0.011)	0 - 0.054	0.002 (0)	-179.86 (11.26)	0.31 (0.079)	0.131 - 0.702
RG	50	16	0.20	0.50	0.207 (0.017)	0.719 (0.068)	0.015 (0.01)	0 - 0.039	0.002 (0)	-182.42 (15.69)	0.219 (0.068)	0.02 - 0.528
RG	50	16	0.20	0.60	0.205 (0.018)	0.7 (0.077)	0.016 (0.011)	0 - 0.06	0.002 (0)	-188.97 (17.21)	0.11 (0.062)	0.002 - 0.382
RG	50	16	0.20	0.70	0.194 (0.021)	0.697 (0.07)	0.016 (0.015)	0 - 0.058	0.002 (0)	-189.11 (20.91)	0.053 (0.045)	0.002 - 0.282
RG	50	16	0.20	0.80	0.198 (0.019)	0.709 (0.086)	0.015 (0.012)	0 - 0.059	0.002 (0)	-189.94 (21.09)	0.109 (0.061)	0.006 - 0.3
RG	50	16	0.20	0.90	0.189 (0.022)	0.698 (0.091)	0.019 (0.014)	0 - 0.059	0.002 (0)	-194.2 (26.45)	0.207 (0.079)	0.019 - 0.443
RG	50	16	0.30	0.10	0.324 (0.018)	0.69 (0.074)	0.026 (0.015)	0.001 - 0.065	0.002 (0)	-190.51 (9.73)	0.59 (0.074)	0.461 - 0.897
RG	50	16	0.30	0.20	0.339 (0.02)	0.665 (0.056)	0.04 (0.019)	0.001 - 0.094	0.003 (0)	-192.1 (9.21)	0.465 (0.056)	0.314 - 0.677
RG	50	16	0.30	0.30	0.313 (0.019)	0.673 (0.053)	0.019 (0.012)	0.001 - 0.049	0.002 (0)	-189.98 (9.35)	0.373 (0.053)	0.238 - 0.629
RG	50	16	0.30	0.40	0.316 (0.02)	0.657 (0.047)	0.021 (0.013)	0 - 0.064	0.002 (0)	-191.57 (9.54)	0.257 (0.047)	0.112 - 0.511
RG	50	16	0.30	0.50	0.312 (0.018)	0.65 (0.049)	0.018 (0.012)	0.001 - 0.049	0.003 (0)	-193.47 (10.27)	0.15 (0.049)	0.016 - 0.492
RG	50	16	0.30	0.60	0.31 (0.018)	0.651 (0.065)	0.018 (0.011)	0 - 0.042	0.003 (0)	-195.59 (11.08)	0.055 (0.062)	0.001 - 0.6
RG	50	16	0.30	0.70	0.305 (0.018)	0.647 (0.045)	0.015 (0.011)	0 - 0.059	0.003 (0)	-195.2 (12.91)	0.061 (0.034)	0.005 - 0.235
RG	50	16	0.30	0.80	0.301 (0.02)	0.655 (0.086)	0.016 (0.011)	0 - 0.049	0.003 (0)	-195.88 (11.96)	0.158 (0.059)	0.046 - 0.589
RG	50	16	0.30	0.90	0.303 (0.018)	0.641 (0.046)	0.014 (0.012)	0 - 0.048	0.003 (0)	-199.25 (16.21)	0.259 (0.042)	0.038 - 0.365
RG	50	16	0.40	0.10	0.412 (0.02)	0.68 (0.131)	0.019 (0.013)	0 - 0.057	0.003 (0)	-196.53 (10.1)	0.58 (0.131)	0.455 - 1.063
RG	50	16	0.40	0.20	0.421 (0.025)	0.619 (0.107)	0.024 (0.022)	0 - 0.147	0.003 (0)	-197.21 (9.76)	0.423 (0.091)	0.186 - 0.79
RG	50	16	0.40	0.30	0.407 (0.018)	0.666 (0.165)	0.016 (0.012)	0 - 0.053	0.003 (0)	-196.56 (8.2)	0.366 (0.165)	0.214 - 1.615

Continued on next page

Table S1 – *Continued from previous page*

Structure	T	N	p	p_e/p_w	$\overline{\hat{p}}$ (SD)	$\overline{\hat{p}_e}/\overline{\hat{p}_w}$ (SD)	$\overline{\lvert\Delta\rvert}_p$ (SD)	Range $\lvert\Delta\rvert_p$	$\overline{SE}\lvert\Delta\rvert_p$ (SD)	$\overline{BIC}$ (SD)	$\overline{\lvert\Delta\rvert}_{p_e/p_w}$ (SD)	Range $\lvert\Delta\rvert_{p_e/p_w}$
RG	50	16	0.40	0.40	0.407 (0.02)	0.616 (0.075)	0.017 (0.013)	0 - 0.067	0.003 (0)	-196.08 (9.8)	0.216 (0.075)	0.064 - 0.601
RG	50	16	0.40	0.50	0.405 (0.02)	0.593 (0.053)	0.015 (0.013)	0 - 0.068	0.003 (0)	-196.51 (10.03)	0.094 (0.05)	0.017 - 0.408
RG	50	16	0.40	0.60	0.402 (0.021)	0.59 (0.058)	0.016 (0.013)	0 - 0.07	0.003 (0)	-196.67 (9.19)	0.034 (0.047)	0.001 - 0.349
RG	50	16	0.40	0.70	0.393 (0.019)	0.579 (0.067)	0.017 (0.011)	0 - 0.047	0.003 (0)	-196.4 (9.39)	0.127 (0.053)	0.013 - 0.257
RG	50	16	0.40	0.80	0.398 (0.021)	0.586 (0.055)	0.017 (0.012)	0 - 0.056	0.003 (0)	-197.22 (8.95)	0.218 (0.036)	0.154 - 0.362
RG	50	16	0.40	0.90	0.393 (0.019)	0.57 (0.05)	0.016 (0.012)	0 - 0.052	0.003 (0)	-200.36 (10.46)	0.33 (0.05)	0.233 - 0.508
RG	50	16	0.50	0.10	0.505 (0.03)	0.575 (0.152)	0.026 (0.016)	0.001 - 0.082	0.004 (0)	-196.46 (9.04)	0.475 (0.152)	0.231 - 1.564
RG	50	16	0.50	0.20	0.502 (0.032)	0.587 (0.169)	0.026 (0.018)	0.001 - 0.086	0.004 (0.001)	-197.9 (9.74)	0.389 (0.166)	0.066 - 1.546
RG	50	16	0.50	0.30	0.501 (0.031)	0.575 (0.147)	0.026 (0.017)	0.002 - 0.088	0.004 (0.001)	-198.37 (9.39)	0.28 (0.136)	0.014 - 0.703
RG	50	16	0.50	0.40	0.504 (0.031)	0.571 (0.143)	0.027 (0.017)	0.002 - 0.076	0.004 (0.001)	-197.8 (9.77)	0.187 (0.12)	0.02 - 0.676
RG	50	16	0.50	0.50	0.507 (0.032)	0.591 (0.24)	0.029 (0.015)	0 - 0.069	0.004 (0.001)	-198.69 (9.7)	0.126 (0.224)	0.008 - 1.987
RG	50	16	0.50	0.60	0.499 (0.029)	0.589 (0.129)	0.025 (0.014)	0 - 0.072	0.004 (0.001)	-197.57 (9.66)	0.093 (0.089)	0.002 - 0.397
RG	50	16	0.50	0.70	0.501 (0.032)	0.567 (0.131)	0.027 (0.016)	0.001 - 0.096	0.004 (0.001)	-197.81 (8.76)	0.165 (0.087)	0.007 - 0.616
RG	50	16	0.50	0.80	0.506 (0.032)	0.593 (0.204)	0.027 (0.017)	0.001 - 0.079	0.004 (0.001)	-196.79 (9.47)	0.251 (0.147)	0.008 - 1.478
RG	50	16	0.50	0.90	0.501 (0.032)	0.564 (0.126)	0.027 (0.017)	0.003 - 0.105	0.004 (0.001)	-198.51 (10.24)	0.342 (0.111)	0.03 - 0.805
RG	50	16	0.60	0.10	0.53 (0.053)	0.714 (0.813)	0.071 (0.051)	0.003 - 0.192	0.004 (0.001)	-205.39 (13.03)	0.618 (0.809)	0.047 - 8.103
RG	50	16	0.60	0.20	0.565 (0.034)	0.667 (0.214)	0.038 (0.031)	0 - 0.179	0.003 (0.001)	-198.95 (9.42)	0.471 (0.206)	0.191 - 1.656
RG	50	16	0.60	0.30	0.577 (0.029)	0.682 (0.234)	0.028 (0.025)	0 - 0.169	0.003 (0.001)	-198.26 (11.26)	0.382 (0.234)	0.21 - 2.386

Continued on next page

Table S1 – *Continued from previous page*

Structure	T	N	p	p_e/p_w	$\overline{\hat{p}}$ (SD)	$\overline{\hat{p_e}}/\overline{\hat{p_w}}$ (SD)	$\overline{\lvert\Delta\rvert}_p$ (SD)	Range $\lvert\Delta\rvert_p$	$\overline{SE}\lvert\Delta\rvert_p$ (SD)	$\overline{BIC}$ (SD)	$\overline{\lvert\Delta\rvert}_{p_e/p_w}$ (SD)	Range $\lvert\Delta\rvert_{p_e/p_w}$
RG	50	16	0.60	0.40	0.588 (0.024)	0.586 (0.073)	0.022 (0.016)	0.001 - 0.076	0.004 (0)	-196.64 (8.67)	0.186 (0.073)	0.111 - 0.655
RG	50	16	0.60	0.50	0.604 (0.025)	0.558 (0.038)	0.019 (0.016)	0 - 0.069	0.004 (0)	-195.26 (8.82)	0.06 (0.036)	0.01 - 0.173
RG	50	16	0.60	0.60	0.607 (0.029)	0.546 (0.034)	0.024 (0.017)	0 - 0.07	0.004 (0)	-196.43 (8.65)	0.058 (0.027)	0 - 0.133
RG	50	16	0.60	0.70	0.62 (0.021)	0.535 (0.029)	0.024 (0.016)	0.001 - 0.062	0.004 (0)	-196.58 (9.1)	0.165 (0.029)	0.076 - 0.239
RG	50	16	0.60	0.80	0.614 (0.028)	0.54 (0.032)	0.025 (0.018)	0 - 0.079	0.004 (0)	-196.66 (7.47)	0.26 (0.032)	0.182 - 0.341
RG	50	16	0.60	0.90	0.628 (0.022)	0.522 (0.03)	0.029 (0.02)	0 - 0.079	0.004 (0)	-198.44 (9.37)	0.378 (0.03)	0.275 - 0.459
RG	50	16	0.70	0.10	0.442 (0.073)	0.536 (0.179)	0.258 (0.073)	0.031 - 0.373	0.004 (0)	-233.77 (22.19)	0.44 (0.168)	0.054 - 1.756
RG	50	16	0.70	0.20	0.607 (0.061)	0.793 (0.842)	0.093 (0.061)	0.014 - 0.311	0.003 (0.001)	-203.53 (15.86)	0.597 (0.839)	0.192 - 8.175
RG	50	16	0.70	0.30	0.639 (0.051)	0.663 (0.105)	0.062 (0.05)	0.001 - 0.23	0.003 (0)	-206.05 (17.01)	0.363 (0.105)	0.21 - 0.874
RG	50	16	0.70	0.40	0.673 (0.026)	0.599 (0.042)	0.03 (0.023)	0.001 - 0.085	0.003 (0)	-196.07 (10.27)	0.199 (0.042)	0.11 - 0.286
RG	50	16	0.70	0.50	0.693 (0.025)	0.556 (0.033)	0.02 (0.017)	0 - 0.08	0.003 (0)	-194.7 (9.5)	0.057 (0.031)	0.006 - 0.124
RG	50	16	0.70	0.60	0.709 (0.018)	0.542 (0.023)	0.016 (0.012)	0 - 0.044	0.003 (0)	-193.78 (11.26)	0.058 (0.021)	0.001 - 0.138
RG	50	16	0.70	0.70	0.716 (0.018)	0.525 (0.029)	0.02 (0.014)	0.001 - 0.054	0.003 (0)	-191.81 (9.51)	0.175 (0.029)	0.105 - 0.25
RG	50	16	0.70	0.80	0.719 (0.017)	0.538 (0.03)	0.021 (0.014)	0 - 0.069	0.003 (0)	-192.66 (9.63)	0.262 (0.03)	0.182 - 0.349
RG	50	16	0.70	0.90	0.725 (0.017)	0.508 (0.033)	0.027 (0.013)	0.001 - 0.061	0.003 (0)	-194.71 (9.61)	0.392 (0.033)	0.295 - 0.45
RG	50	16	0.80	0.10	0.342 (0.037)	0.328 (0.196)	0.458 (0.037)	0.343 - 0.511	0.003 (0)	-229.45 (40.1)	0.253 (0.162)	0.005 - 0.444
RG	50	16	0.80	0.20	0.611 (0.108)	0.775 (0.747)	0.189 (0.108)	0.045 - 0.441	0.003 (0)	-216.51 (30.38)	0.585 (0.739)	0.135 - 6.92
RG	50	16	0.80	0.30	0.711 (0.057)	0.69 (0.103)	0.089 (0.057)	0.005 - 0.262	0.003 (0)	-211.14 (31.09)	0.39 (0.103)	0.21 - 0.784
RG	50	16	0.80	0.40	0.773 (0.024)	0.596 (0.033)	0.03 (0.021)	0 - 0.093	0.003 (0)	-185.57 (9.94)	0.196 (0.033)	0.111 - 0.282

Continued on next page

Table S1 – *Continued from previous page*

Structure	T	N	p	p_e/p_w	$\overline{\hat{p}}$ (SD)	$\overline{\hat{p}_e}/\overline{\hat{p}_w}$ (SD)	$\overline{\lvert\Delta\rvert}_p$ (SD)	Range $\lvert\Delta\rvert_p$	$\overline{SE}\lvert\Delta\rvert_p$ (SD)	$\overline{BIC}$ (SD)	$\overline{\lvert\Delta\rvert}_{p_e/p_w}$ (SD)	Range $\lvert\Delta\rvert_{p_e/p_w}$
RG	50	16	0.80	0.50	0.796 (0.022)	0.552 (0.041)	0.017 (0.014)	0 - 0.062	0.002 (0)	-180.42 (10.25)	0.059 (0.03)	0.008 - 0.119
RG	50	16	0.80	0.60	0.803 (0.015)	0.533 (0.044)	0.012 (0.009)	0.001 - 0.039	0.002 (0)	-180.11 (10.07)	0.069 (0.041)	0.001 - 0.196
RG	50	16	0.80	0.70	0.811 (0.013)	0.485 (0.056)	0.014 (0.01)	0 - 0.048	0.002 (0)	-180.18 (11.09)	0.215 (0.056)	0.089 - 0.315
RG	50	16	0.80	0.80	0.808 (0.015)	0.494 (0.058)	0.014 (0.01)	0 - 0.035	0.002 (0)	-179.64 (10.86)	0.306 (0.058)	0.206 - 0.417
RG	50	16	0.80	0.90	0.81 (0.014)	0.453 (0.056)	0.014 (0.009)	0 - 0.04	0.002 (0)	-181.09 (12.52)	0.447 (0.056)	0.35 - 0.523
RG	50	16	0.90	0.10	0.282 (0.022)	0.267 (0.239)	0.618 (0.022)	0.477 - 0.674	0.003 (0)	-233.72 (42.35)	0.223 (0.189)	0.011 - 0.514
RG	50	16	0.90	0.20	0.692 (0.13)	0.898 (1.969)	0.208 (0.13)	0.054 - 0.566	0.003 (0)	-213.8 (46.35)	0.704 (1.967)	0.094 - 20.092
RG	50	16	0.90	0.30	0.819 (0.048)	0.723 (0.086)	0.081 (0.048)	0.023 - 0.257	0.002 (0)	-200.08 (40.41)	0.423 (0.086)	0.211 - 0.585
RG	50	16	0.90	0.40	0.88 (0.014)	0.531 (0.067)	0.02 (0.014)	0 - 0.054	0.002 (0)	-158.77 (11.23)	0.131 (0.067)	0.003 - 0.286
RG	50	16	0.90	0.50	0.895 (0.012)	0.456 (0.048)	0.01 (0.009)	0 - 0.039	0.002 (0)	-146.97 (11.18)	0.055 (0.034)	0.003 - 0.143
RG	50	16	0.90	0.60	0.899 (0.011)	0.435 (0.055)	0.009 (0.006)	0 - 0.035	0.002 (0)	-145.86 (9.39)	0.165 (0.053)	0.026 - 0.287
RG	50	16	0.90	0.70	0.902 (0.01)	0.416 (0.048)	0.008 (0.006)	0 - 0.026	0.002 (0)	-143.47 (8.77)	0.284 (0.048)	0.117 - 0.401
RG	50	16	0.90	0.80	0.903 (0.012)	0.417 (0.042)	0.01 (0.007)	0 - 0.036	0.002 (0)	-142.05 (10.35)	0.383 (0.042)	0.283 - 0.475
RG	50	16	0.90	0.90	0.902 (0.011)	0.409 (0.043)	0.009 (0.007)	0 - 0.031	0.002 (0)	-143.57 (9.53)	0.491 (0.043)	0.366 - 0.61
RG	50	25	0.10	0.10	0.149 (0.024)	0.746 (0.097)	0.049 (0.024)	0.003 - 0.112	0.001 (0)	-199.09 (24.24)	0.646 (0.097)	0.349 - 0.737
RG	50	25	0.10	0.20	0.107 (0.011)	0.746 (0.105)	0.01 (0.007)	0 - 0.042	0.001 (0)	-177.37 (15)	0.546 (0.105)	0.348 - 0.685
RG	50	25	0.10	0.30	0.111 (0.013)	0.726 (0.12)	0.013 (0.01)	0 - 0.042	0.001 (0)	-179.23 (15.66)	0.426 (0.12)	0.23 - 0.585
RG	50	25	0.10	0.40	0.103 (0.01)	0.693 (0.143)	0.008 (0.007)	0 - 0.031	0.001 (0)	-169.01 (10.04)	0.296 (0.136)	0.061 - 0.492

Continued on next page

Table S1 – *Continued from previous page*

Structure	T	N	p	p_e/p_w	$\overline{\hat{p}}$ (SD)	$\overline{\hat{p}_e}/\overline{\hat{p}_w}$ (SD)	$\overline{\lvert\Delta\rvert}_p$ (SD)	Range $\lvert\Delta\rvert_p$	$\overline{SE}\lvert\Delta\rvert_p$ (SD)	$\overline{BIC}$ (SD)	$\overline{\lvert\Delta\rvert}_{p_e/p_w}$ (SD)	Range $\lvert\Delta\rvert_{p_e/p_w}$
RG	50	25	0.10	0.50	0.1 (0.008)	0.682 (0.169)	0.006 (0.006)	0 - 0.025	0.001 (0)	-167.38 (9.62)	0.206 (0.138)	0.001 - 0.396
RG	50	25	0.10	0.60	0.1 (0.009)	0.676 (0.177)	0.007 (0.005)	0 - 0.023	0.001 (0)	-166.8 (8.65)	0.162 (0.104)	0.001 - 0.359
RG	50	25	0.10	0.70	0.099 (0.009)	0.717 (0.171)	0.007 (0.006)	0 - 0.029	0.001 (0)	-166.39 (9.53)	0.152 (0.078)	0.037 - 0.558
RG	50	25	0.10	0.80	0.1 (0.009)	0.68 (0.164)	0.007 (0.005)	0 - 0.023	0.001 (0)	-168.2 (9.93)	0.157 (0.127)	0.001 - 0.598
RG	50	25	0.10	0.90	0.101 (0.009)	0.687 (0.177)	0.007 (0.006)	0 - 0.032	0.001 (0)	-166.57 (8.54)	0.213 (0.177)	0.008 - 0.732
RG	50	25	0.20	0.10	0.259 (0.021)	0.595 (0.101)	0.059 (0.021)	0.02 - 0.112	0.002 (0)	-208.78 (10.2)	0.495 (0.101)	0.318 - 0.854
RG	50	25	0.20	0.20	0.211 (0.013)	0.699 (0.106)	0.015 (0.009)	0 - 0.042	0.002 (0)	-201.08 (10.12)	0.499 (0.106)	0.27 - 0.631
RG	50	25	0.20	0.30	0.211 (0.014)	0.684 (0.106)	0.014 (0.011)	0 - 0.05	0.002 (0)	-199.67 (10.08)	0.384 (0.106)	0.165 - 0.617
RG	50	25	0.20	0.40	0.205 (0.013)	0.685 (0.12)	0.011 (0.009)	0 - 0.057	0.002 (0)	-199.13 (9.29)	0.285 (0.12)	0.086 - 0.5
RG	50	25	0.20	0.50	0.202 (0.012)	0.642 (0.141)	0.01 (0.008)	0 - 0.038	0.002 (0)	-197.06 (8.94)	0.15 (0.132)	0 - 0.333
RG	50	25	0.20	0.60	0.199 (0.011)	0.654 (0.155)	0.009 (0.007)	0 - 0.034	0.002 (0)	-199.1 (9.24)	0.15 (0.065)	0.003 - 0.26
RG	50	25	0.20	0.70	0.2 (0.012)	0.62 (0.198)	0.009 (0.007)	0.001 - 0.031	0.002 (0)	-198.4 (9.79)	0.179 (0.115)	0.062 - 0.539
RG	50	25	0.20	0.80	0.199 (0.012)	0.583 (0.235)	0.009 (0.008)	0 - 0.036	0.002 (0)	-197.51 (13.05)	0.223 (0.228)	0.001 - 0.717
RG	50	25	0.20	0.90	0.199 (0.011)	0.579 (0.221)	0.008 (0.007)	0 - 0.05	0.002 (0)	-198.26 (10.81)	0.321 (0.219)	0.048 - 0.836
RG	50	25	0.30	0.10	0.356 (0.021)	0.661 (0.088)	0.056 (0.021)	0.007 - 0.107	0.002 (0)	-216.05 (7.88)	0.561 (0.088)	0.391 - 0.902
RG	50	25	0.30	0.20	0.319 (0.018)	0.633 (0.071)	0.022 (0.014)	0 - 0.054	0.002 (0)	-211.69 (10.51)	0.433 (0.071)	0.3 - 0.725
RG	50	25	0.30	0.30	0.318 (0.017)	0.631 (0.063)	0.02 (0.015)	0 - 0.078	0.002 (0)	-213.13 (9.74)	0.331 (0.063)	0.191 - 0.536
RG	50	25	0.30	0.40	0.309 (0.017)	0.609 (0.058)	0.016 (0.012)	0.001 - 0.051	0.002 (0)	-213.74 (10.26)	0.209 (0.058)	0.088 - 0.342
RG	50	25	0.30	0.50	0.305 (0.018)	0.577 (0.064)	0.015 (0.012)	0 - 0.051	0.002 (0)	-213.23 (10.24)	0.078 (0.063)	0 - 0.321

Continued on next page

Table S1 – *Continued from previous page*

Structure	T	N	p	p_e/p_w	$\overline{\hat{p}}$ (SD)	$\overline{\hat{p}_e}/\overline{\hat{p}_w}$ (SD)	$\overline{\lvert\Delta\rvert}_p$ (SD)	Range $\lvert\Delta\rvert_p$	$\overline{SE}\lvert\Delta\rvert_p$ (SD)	$\overline{BIC}$ (SD)	$\overline{\lvert\Delta\rvert}_{p_e/p_w}$ (SD)	Range $\lvert\Delta\rvert_{p_e/p_w}$
RG	50	25	0.30	0.60	0.303 (0.018)	0.585 (0.058)	0.014 (0.011)	0 - 0.065	0.002 (0)	-214.89 (13.9)	0.049 (0.034)	0.001 - 0.126
RG	50	25	0.30	0.70	0.299 (0.017)	0.587 (0.059)	0.014 (0.01)	0.001 - 0.06	0.002 (0)	-215.76 (13.62)	0.114 (0.056)	0.006 - 0.206
RG	50	25	0.30	0.80	0.294 (0.016)	0.586 (0.07)	0.013 (0.011)	0 - 0.05	0.002 (0)	-219.16 (16.2)	0.214 (0.07)	0.019 - 0.314
RG	50	25	0.30	0.90	0.292 (0.017)	0.584 (0.079)	0.015 (0.012)	0 - 0.057	0.002 (0)	-217.06 (16.75)	0.316 (0.079)	0.074 - 0.491
RG	50	25	0.40	0.10	0.433 (0.017)	0.686 (0.143)	0.033 (0.016)	0.002 - 0.074	0.002 (0)	-219.89 (10.76)	0.586 (0.143)	0.397 - 1.042
RG	50	25	0.40	0.20	0.419 (0.019)	0.634 (0.109)	0.023 (0.014)	0 - 0.062	0.002 (0)	-217.06 (9.22)	0.434 (0.109)	0.289 - 0.861
RG	50	25	0.40	0.30	0.418 (0.017)	0.597 (0.072)	0.021 (0.014)	0 - 0.07	0.002 (0)	-218.5 (9.41)	0.297 (0.072)	0.198 - 0.693
RG	50	25	0.40	0.40	0.412 (0.016)	0.582 (0.054)	0.016 (0.012)	0 - 0.073	0.002 (0)	-217.45 (9.27)	0.182 (0.054)	0.082 - 0.568
RG	50	25	0.40	0.50	0.411 (0.019)	0.566 (0.059)	0.018 (0.012)	0 - 0.049	0.003 (0)	-218.13 (9.92)	0.067 (0.058)	0.001 - 0.501
RG	50	25	0.40	0.60	0.403 (0.018)	0.564 (0.051)	0.014 (0.01)	0.001 - 0.041	0.003 (0)	-218.51 (10.28)	0.044 (0.044)	0.004 - 0.314
RG	50	25	0.40	0.70	0.396 (0.02)	0.561 (0.036)	0.015 (0.014)	0 - 0.06	0.003 (0)	-219.79 (9.49)	0.139 (0.036)	0.071 - 0.226
RG	50	25	0.40	0.80	0.395 (0.017)	0.563 (0.048)	0.014 (0.012)	0 - 0.06	0.003 (0)	-222.2 (10.58)	0.237 (0.048)	0.06 - 0.349
RG	50	25	0.40	0.90	0.395 (0.016)	0.556 (0.042)	0.013 (0.011)	0 - 0.056	0.003 (0)	-223.24 (11.38)	0.344 (0.042)	0.242 - 0.451
RG	50	25	0.50	0.10	0.502 (0.028)	0.58 (0.152)	0.024 (0.014)	0.002 - 0.066	0.003 (0.001)	-218.21 (9.08)	0.48 (0.152)	0.001 - 0.959
RG	50	25	0.50	0.20	0.5 (0.027)	0.581 (0.182)	0.023 (0.013)	0.005 - 0.07	0.003 (0.001)	-219.66 (11.02)	0.39 (0.162)	0.133 - 0.899
RG	50	25	0.50	0.30	0.5 (0.025)	0.59 (0.178)	0.021 (0.013)	0 - 0.075	0.003 (0.001)	-218.07 (9.47)	0.303 (0.156)	0.041 - 0.768
RG	50	25	0.50	0.40	0.5 (0.027)	0.559 (0.16)	0.023 (0.013)	0.002 - 0.056	0.003 (0.001)	-218.42 (8.57)	0.168 (0.151)	0.009 - 1.065
RG	50	25	0.50	0.50	0.498 (0.027)	0.586 (0.163)	0.023 (0.013)	0.004 - 0.058	0.003 (0.001)	-219.39 (9.46)	0.117 (0.142)	0 - 0.532

Continued on next page

Table S1 – *Continued from previous page*

Structure	T	N	p	p_e/p_w	$\overline{\hat{p}}$ (SD)	$\overline{\hat{p}_e}/\overline{\hat{p}_w}$ (SD)	$\overline{\lvert\Delta\rvert}_p$ (SD)	Range $\lvert\Delta\rvert_p$	$\overline{SE}\lvert\Delta\rvert_p$ (SD)	$\overline{BIC}$ (SD)	$\overline{\lvert\Delta\rvert}_{p_e/p_w}$ (SD)	Range $\lvert\Delta\rvert_{p_e/p_w}$
RG	50	25	0.50	0.60	0.503 (0.028)	0.623 (0.468)	0.024 (0.015)	0.003 - 0.069	0.003 (0.001)	-217.45 (8.09)	0.164 (0.438)	0.012 - 4.305
RG	50	25	0.50	0.70	0.504 (0.029)	0.593 (0.179)	0.026 (0.013)	0.003 - 0.055	0.003 (0.001)	-219.64 (10.24)	0.164 (0.128)	0 - 1.065
RG	50	25	0.50	0.80	0.497 (0.027)	0.574 (0.153)	0.024 (0.013)	0 - 0.058	0.003 (0.001)	-218.66 (8.89)	0.257 (0.092)	0.001 - 0.453
RG	50	25	0.50	0.90	0.501 (0.027)	0.624 (0.179)	0.023 (0.013)	0.001 - 0.074	0.003 (0.001)	-219.33 (10.34)	0.304 (0.123)	0.012 - 0.601
RG	50	25	0.60	0.10	0.553 (0.032)	0.752 (0.28)	0.047 (0.032)	0.001 - 0.179	0.002 (0)	-217.66 (8.22)	0.654 (0.276)	0.075 - 2.824
RG	50	25	0.60	0.20	0.574 (0.021)	0.629 (0.075)	0.028 (0.018)	0.001 - 0.069	0.003 (0)	-218.46 (9.27)	0.429 (0.075)	0.304 - 0.885
RG	50	25	0.60	0.30	0.575 (0.02)	0.617 (0.066)	0.026 (0.018)	0 - 0.082	0.003 (0)	-218.18 (9.3)	0.317 (0.066)	0.219 - 0.71
RG	50	25	0.60	0.40	0.587 (0.021)	0.57 (0.044)	0.019 (0.015)	0.001 - 0.064	0.003 (0)	-218.87 (9.06)	0.17 (0.044)	0.056 - 0.383
RG	50	25	0.60	0.50	0.599 (0.022)	0.538 (0.029)	0.018 (0.013)	0 - 0.059	0.003 (0)	-217.04 (8.59)	0.041 (0.024)	0.001 - 0.099
RG	50	25	0.60	0.60	0.605 (0.021)	0.532 (0.027)	0.018 (0.012)	0 - 0.056	0.003 (0)	-217.49 (8.9)	0.068 (0.027)	0.005 - 0.13
RG	50	25	0.60	0.70	0.61 (0.02)	0.529 (0.025)	0.018 (0.013)	0 - 0.055	0.003 (0)	-218.08 (9.45)	0.171 (0.025)	0.104 - 0.22
RG	50	25	0.60	0.80	0.618 (0.018)	0.511 (0.029)	0.021 (0.013)	0 - 0.054	0.003 (0)	-219.12 (10.24)	0.289 (0.029)	0.22 - 0.346
RG	50	25	0.60	0.90	0.624 (0.019)	0.508 (0.026)	0.026 (0.016)	0.001 - 0.064	0.003 (0)	-219.79 (9.06)	0.392 (0.026)	0.306 - 0.474
RG	50	25	0.70	0.10	0.581 (0.071)	0.867 (0.463)	0.119 (0.071)	0.041 - 0.305	0.002 (0)	-221.48 (8.64)	0.77 (0.458)	0.053 - 3.955
RG	50	25	0.70	0.20	0.639 (0.025)	0.628 (0.075)	0.061 (0.025)	0.003 - 0.119	0.003 (0)	-216.21 (8.92)	0.428 (0.075)	0.332 - 1.114
RG	50	25	0.70	0.30	0.652 (0.021)	0.612 (0.025)	0.048 (0.02)	0.001 - 0.101	0.003 (0)	-216.55 (8.97)	0.312 (0.025)	0.268 - 0.414
RG	50	25	0.70	0.40	0.676 (0.018)	0.571 (0.026)	0.026 (0.016)	0 - 0.066	0.003 (0)	-216.81 (10.3)	0.171 (0.026)	0.068 - 0.224
RG	50	25	0.70	0.50	0.696 (0.019)	0.526 (0.037)	0.015 (0.012)	0 - 0.062	0.002 (0)	-216.18 (9.88)	0.043 (0.016)	0.002 - 0.098

Continued on next page

Table S1 – *Continued from previous page*

Structure	T	N	p	p_e/p_w	$\overline{\hat{p}}$ (SD)	$\overline{\hat{p_e}}/\overline{\hat{p_w}}$ (SD)	$\overline{\lvert\Delta\rvert}_p$ (SD)	Range $\lvert\Delta\rvert_p$	$\overline{SE}\lvert\Delta\rvert_p$ (SD)	$\overline{BIC}$ (SD)	$\overline{\lvert\Delta\rvert}_{p_e/p_w}$ (SD)	Range $\lvert\Delta\rvert_{p_e/p_w}$
RG	50	25	0.70	0.60	0.706 (0.016)	0.513 (0.042)	0.013 (0.01)	0 - 0.05	0.002 (0)	-216.12 (10.93)	0.087 (0.042)	0.004 - 0.154
RG	50	25	0.70	0.70	0.709 (0.016)	0.498 (0.035)	0.015 (0.01)	0 - 0.045	0.002 (0)	-214.1 (11.82)	0.202 (0.035)	0.145 - 0.253
RG	50	25	0.70	0.80	0.711 (0.016)	0.489 (0.027)	0.016 (0.011)	0 - 0.044	0.002 (0)	-218.87 (12.68)	0.311 (0.027)	0.252 - 0.353
RG	50	25	0.70	0.90	0.714 (0.012)	0.496 (0.03)	0.016 (0.01)	0 - 0.043	0.002 (0)	-216.93 (12.59)	0.404 (0.03)	0.309 - 0.455
RG	50	25	0.80	0.10	0.588 (0.118)	0.737 (0.452)	0.212 (0.118)	0.086 - 0.433	0.002 (0)	-230.75 (21.39)	0.653 (0.428)	0.003 - 2.544
RG	50	25	0.80	0.20	0.732 (0.023)	0.629 (0.022)	0.068 (0.023)	0.016 - 0.146	0.002 (0)	-215.72 (12.79)	0.429 (0.022)	0.368 - 0.479
RG	50	25	0.80	0.30	0.756 (0.021)	0.628 (0.022)	0.044 (0.021)	0.001 - 0.097	0.002 (0)	-211.68 (10.95)	0.328 (0.022)	0.272 - 0.377
RG	50	25	0.80	0.40	0.789 (0.015)	0.587 (0.032)	0.015 (0.012)	0 - 0.05	0.002 (0)	-202.44 (12.25)	0.187 (0.032)	0.051 - 0.265
RG	50	25	0.80	0.50	0.798 (0.012)	0.535 (0.037)	0.009 (0.008)	0 - 0.045	0.002 (0)	-196.77 (8.55)	0.043 (0.028)	0 - 0.132
RG	50	25	0.80	0.60	0.803 (0.012)	0.504 (0.04)	0.01 (0.007)	0 - 0.036	0.002 (0)	-197.25 (9.56)	0.096 (0.04)	0.011 - 0.207
RG	50	25	0.80	0.70	0.803 (0.012)	0.495 (0.039)	0.01 (0.007)	0 - 0.029	0.002 (0)	-197.21 (9.72)	0.205 (0.039)	0.101 - 0.327
RG	50	25	0.80	0.80	0.806 (0.01)	0.484 (0.048)	0.009 (0.007)	0 - 0.027	0.002 (0)	-197.59 (10.63)	0.316 (0.048)	0.189 - 0.429
RG	50	25	0.80	0.90	0.807 (0.011)	0.478 (0.046)	0.01 (0.008)	0 - 0.032	0.002 (0)	-197.9 (11.43)	0.422 (0.046)	0.315 - 0.522
RG	50	25	0.90	0.10	0.657 (0.135)	0.819 (0.431)	0.243 (0.135)	0.127 - 0.555	0.002 (0)	-238.38 (31.69)	0.724 (0.423)	0.013 - 1.829
RG	50	25	0.90	0.20	0.875 (0.014)	0.656 (0.075)	0.026 (0.013)	0.004 - 0.064	0.001 (0)	-184.6 (13.18)	0.456 (0.075)	0.17 - 0.556
RG	50	25	0.90	0.30	0.883 (0.009)	0.609 (0.102)	0.017 (0.008)	0.001 - 0.044	0.001 (0)	-181.78 (10.78)	0.309 (0.102)	0.032 - 0.44
RG	50	25	0.90	0.40	0.896 (0.009)	0.451 (0.096)	0.008 (0.006)	0 - 0.028	0.001 (0)	-168.76 (9.62)	0.07 (0.083)	0.002 - 0.322
RG	50	25	0.90	0.50	0.9 (0.008)	0.41 (0.082)	0.007 (0.005)	0 - 0.022	0.001 (0)	-165.42 (8.87)	0.112 (0.046)	0.002 - 0.259
RG	50	25	0.90	0.60	0.9 (0.009)	0.41 (0.079)	0.008 (0.005)	0 - 0.021	0.001 (0)	-167.94 (9.72)	0.192 (0.073)	0.009 - 0.316

Continued on next page

Table S1 – *Continued from previous page*

Structure	T	N	p	p_e/p_w	$\overline{\hat{p}}$ (SD)	$\overline{\hat{p}_e}/\overline{\hat{p}_w}$ (SD)	$\overline{\lvert\Delta\rvert}_p$ (SD)	Range $\lvert\Delta\rvert_p$	$\overline{SE}\lvert\Delta\rvert_p$ (SD)	$\overline{BIC}$ (SD)	$\overline{\lvert\Delta\rvert}_{p_e/p_w}$ (SD)	Range $\lvert\Delta\rvert_{p_e/p_w}$
RG	50	25	0.90	0.70	0.902 (0.009)	0.399 (0.066)	0.007 (0.005)	0 - 0.024	0.001 (0)	-166.69 (9.28)	0.301 (0.066)	0.049 - 0.446
RG	50	25	0.90	0.80	0.902 (0.008)	0.411 (0.08)	0.007 (0.005)	0 - 0.024	0.001 (0)	-166.61 (8.6)	0.389 (0.08)	0.137 - 0.558
RG	50	25	0.90	0.90	0.901 (0.008)	0.408 (0.077)	0.007 (0.005)	0 - 0.026	0.001 (0)	-166.01 (8.12)	0.492 (0.077)	0.244 - 0.624
RG	50	49	0.10	0.10	0.109 (0.009)	0.745 (0.101)	0.01 (0.007)	0 - 0.031	0.001 (0)	-223.6 (24.34)	0.645 (0.101)	0.438 - 0.766
RG	50	49	0.10	0.20	0.108 (0.011)	0.743 (0.094)	0.01 (0.009)	0 - 0.039	0.001 (0)	-207.61 (13.5)	0.543 (0.094)	0.264 - 0.661
RG	50	49	0.10	0.30	0.101 (0.006)	0.728 (0.115)	0.005 (0.004)	0 - 0.019	0.001 (0)	-200.57 (9.33)	0.428 (0.115)	0.144 - 0.566
RG	50	49	0.10	0.40	0.1 (0.006)	0.702 (0.137)	0.005 (0.004)	0 - 0.017	0.001 (0)	-201.78 (9.86)	0.302 (0.137)	0.013 - 0.466
RG	50	49	0.10	0.50	0.1 (0.007)	0.721 (0.12)	0.005 (0.004)	0 - 0.019	0.001 (0)	-202.79 (10.27)	0.226 (0.11)	0.001 - 0.366
RG	50	49	0.10	0.60	0.099 (0.007)	0.72 (0.132)	0.005 (0.004)	0 - 0.016	0.001 (0)	-200.07 (9.02)	0.15 (0.097)	0.001 - 0.287
RG	50	49	0.10	0.70	0.099 (0.006)	0.722 (0.136)	0.005 (0.004)	0 - 0.016	0.001 (0)	-200.6 (9.93)	0.121 (0.064)	0.009 - 0.311
RG	50	49	0.10	0.80	0.099 (0.006)	0.717 (0.143)	0.005 (0.004)	0 - 0.019	0.001 (0)	-201.07 (9.85)	0.13 (0.102)	0.003 - 0.39
RG	50	49	0.10	0.90	0.099 (0.006)	0.715 (0.144)	0.004 (0.003)	0 - 0.014	0.001 (0)	-201.38 (9.08)	0.185 (0.144)	0.014 - 0.476
RG	50	49	0.20	0.10	0.213 (0.01)	0.731 (0.076)	0.013 (0.008)	0 - 0.034	0.001 (0)	-236.41 (11.08)	0.631 (0.076)	0.462 - 0.774
RG	50	49	0.20	0.20	0.207 (0.01)	0.705 (0.106)	0.01 (0.007)	0 - 0.024	0.001 (0)	-237.46 (11.69)	0.505 (0.106)	0.259 - 0.63
RG	50	49	0.20	0.30	0.205 (0.011)	0.678 (0.127)	0.009 (0.009)	0 - 0.038	0.001 (0)	-234.01 (12.62)	0.378 (0.127)	0.141 - 0.533
RG	50	49	0.20	0.40	0.202 (0.008)	0.676 (0.141)	0.007 (0.004)	0 - 0.019	0.001 (0)	-231.73 (9.7)	0.279 (0.134)	0.004 - 0.433
RG	50	49	0.20	0.50	0.2 (0.01)	0.643 (0.155)	0.008 (0.006)	0 - 0.029	0.001 (0)	-230.71 (10.07)	0.17 (0.124)	0.001 - 0.326
RG	50	49	0.20	0.60	0.2 (0.008)	0.658 (0.175)	0.007 (0.005)	0 - 0.022	0.001 (0)	-231.34 (8.47)	0.168 (0.075)	0.009 - 0.342
RG	50	49	0.20	0.70	0.2 (0.008)	0.661 (0.175)	0.006 (0.005)	0 - 0.02	0.001 (0)	-230.61 (8.75)	0.157 (0.086)	0.067 - 0.447

Continued on next page

Table S1 – *Continued from previous page*

Structure	T	N	p	p_e/p_w	$\overline{\hat{p}}$ (SD)	$\overline{\hat{p_e}}/\overline{\hat{p_w}}$ (SD)	$\overline{\lvert\Delta\rvert}_p$ (SD)	Range $\lvert\Delta\rvert_p$	$\overline{SE}\lvert\Delta\rvert_p$ (SD)	$\overline{BIC}$ (SD)	$\overline{\lvert\Delta\rvert}_{p_e/p_w}$ (SD)	Range $\lvert\Delta\rvert_{p_e/p_w}$
RG	50	49	0.20	0.80	0.199 (0.009)	0.633 (0.175)	0.007 (0.005)	0 - 0.021	0.001 (0)	-230.38 (9.78)	0.175 (0.167)	0 - 0.568
RG	50	49	0.20	0.90	0.198 (0.008)	0.62 (0.184)	0.007 (0.005)	0 - 0.02	0.001 (0)	-231.04 (9.52)	0.28 (0.184)	0.067 - 0.629
RG	50	49	0.30	0.10	0.323 (0.013)	0.647 (0.079)	0.023 (0.012)	0 - 0.051	0.001 (0)	-247.2 (10.21)	0.547 (0.079)	0.382 - 0.748
RG	50	49	0.30	0.20	0.318 (0.013)	0.591 (0.078)	0.018 (0.012)	0 - 0.052	0.001 (0)	-246.65 (8.9)	0.391 (0.078)	0.271 - 0.516
RG	50	49	0.30	0.30	0.311 (0.012)	0.583 (0.072)	0.013 (0.009)	0 - 0.04	0.001 (0)	-247.24 (10.14)	0.283 (0.072)	0.178 - 0.428
RG	50	49	0.30	0.40	0.305 (0.011)	0.577 (0.074)	0.009 (0.008)	0 - 0.035	0.001 (0)	-244.91 (8.76)	0.177 (0.074)	0.07 - 0.325
RG	50	49	0.30	0.50	0.299 (0.01)	0.567 (0.084)	0.008 (0.006)	0 - 0.025	0.001 (0)	-245.26 (11.51)	0.069 (0.083)	0 - 0.325
RG	50	49	0.30	0.60	0.298 (0.01)	0.579 (0.087)	0.008 (0.006)	0 - 0.023	0.001 (0)	-246.24 (11.6)	0.085 (0.028)	0.018 - 0.164
RG	50	49	0.30	0.70	0.297 (0.01)	0.583 (0.126)	0.008 (0.006)	0 - 0.026	0.001 (0)	-244.12 (11.36)	0.141 (0.098)	0 - 0.428
RG	50	49	0.30	0.80	0.298 (0.011)	0.542 (0.121)	0.009 (0.007)	0 - 0.027	0.001 (0)	-244.77 (11.38)	0.259 (0.118)	0.066 - 0.565
RG	50	49	0.30	0.90	0.298 (0.009)	0.582 (0.141)	0.008 (0.006)	0 - 0.028	0.001 (0)	-247.09 (16.68)	0.318 (0.141)	0.036 - 0.736
RG	50	49	0.40	0.10	0.419 (0.012)	0.623 (0.086)	0.02 (0.011)	0.003 - 0.044	0.002 (0)	-252.51 (8.81)	0.523 (0.086)	0.417 - 0.818
RG	50	49	0.40	0.20	0.425 (0.016)	0.586 (0.053)	0.025 (0.015)	0.001 - 0.072	0.002 (0)	-253.4 (10.52)	0.386 (0.053)	0.299 - 0.759
RG	50	49	0.40	0.30	0.417 (0.015)	0.569 (0.045)	0.018 (0.013)	0.002 - 0.069	0.002 (0)	-252.07 (10.3)	0.269 (0.045)	0.191 - 0.605
RG	50	49	0.40	0.40	0.414 (0.014)	0.553 (0.032)	0.016 (0.011)	0 - 0.06	0.002 (0)	-250.39 (9.68)	0.153 (0.032)	0.092 - 0.209
RG	50	49	0.40	0.50	0.406 (0.015)	0.55 (0.032)	0.014 (0.008)	0 - 0.039	0.002 (0)	-253.02 (10.51)	0.05 (0.031)	0 - 0.118
RG	50	49	0.40	0.60	0.401 (0.012)	0.547 (0.046)	0.01 (0.007)	0 - 0.027	0.002 (0)	-253.64 (11.59)	0.058 (0.039)	0.012 - 0.25
RG	50	49	0.40	0.70	0.4 (0.01)	0.545 (0.039)	0.008 (0.007)	0 - 0.045	0.002 (0)	-253.74 (12.11)	0.156 (0.037)	0.026 - 0.226

Continued on next page

Table S1 – *Continued from previous page*

Structure	T	N	p	p_e/p_w	$\overline{\hat{p}}$ (SD)	$\overline{\hat{p}_e}/\overline{\hat{p}_w}$ (SD)	$\overline{\lvert\Delta\rvert}_p$ (SD)	Range $\lvert\Delta\rvert_p$	$\overline{SE}\lvert\Delta\rvert_p$ (SD)	$\overline{BIC}$ (SD)	$\overline{\lvert\Delta\rvert}_{p_e/p_w}$ (SD)	Range $\lvert\Delta\rvert_{p_e/p_w}$
RG	50	49	0.40	0.80	0.394 (0.011)	0.539 (0.035)	0.01 (0.007)	0 - 0.032	0.002 (0)	-256.38 (12.13)	0.261 (0.035)	0.208 - 0.34
RG	50	49	0.40	0.90	0.392 (0.012)	0.54 (0.041)	0.012 (0.009)	0 - 0.043	0.002 (0)	-256.77 (13.15)	0.36 (0.041)	0.192 - 0.439
RG	50	49	0.50	0.10	0.499 (0.02)	0.591 (0.25)	0.018 (0.009)	0 - 0.044	0.002 (0)	-251.37 (10.39)	0.492 (0.246)	0.079 - 2.329
RG	50	49	0.50	0.20	0.501 (0.02)	0.586 (0.165)	0.018 (0.009)	0.001 - 0.049	0.002 (0)	-250.88 (8.95)	0.386 (0.163)	0.046 - 0.807
RG	50	49	0.50	0.30	0.502 (0.021)	0.534 (0.141)	0.019 (0.009)	0.003 - 0.045	0.002 (0)	-253.1 (10.21)	0.248 (0.115)	0.094 - 0.632
RG	50	49	0.50	0.40	0.501 (0.02)	0.547 (0.176)	0.018 (0.009)	0.002 - 0.043	0.002 (0)	-252.75 (9.4)	0.176 (0.146)	0 - 0.597
RG	50	49	0.50	0.50	0.5 (0.021)	0.574 (0.156)	0.018 (0.012)	0 - 0.066	0.002 (0)	-251.29 (9.12)	0.108 (0.135)	0 - 0.557
RG	50	49	0.50	0.60	0.502 (0.021)	0.625 (0.562)	0.019 (0.009)	0.002 - 0.045	0.002 (0)	-252.15 (9.97)	0.191 (0.529)	0.002 - 5.272
RG	50	49	0.50	0.70	0.501 (0.02)	0.583 (0.269)	0.018 (0.009)	0.001 - 0.058	0.002 (0)	-253.4 (10.78)	0.196 (0.217)	0.009 - 2.043
RG	50	49	0.50	0.80	0.5 (0.02)	0.579 (0.291)	0.018 (0.009)	0.002 - 0.044	0.002 (0)	-252.59 (10.24)	0.282 (0.232)	0.035 - 2.299
RG	50	49	0.50	0.90	0.504 (0.02)	0.553 (0.159)	0.018 (0.01)	0.004 - 0.044	0.002 (0)	-251.95 (8.86)	0.35 (0.151)	0 - 0.796
RG	50	49	0.60	0.10	0.545 (0.044)	0.576 (0.208)	0.055 (0.044)	0.011 - 0.162	0.002 (0)	-252.72 (8.95)	0.485 (0.186)	0.01 - 0.867
RG	50	49	0.60	0.20	0.567 (0.014)	0.581 (0.026)	0.033 (0.014)	0.002 - 0.074	0.002 (0)	-251.38 (9.7)	0.381 (0.026)	0.337 - 0.452
RG	50	49	0.60	0.30	0.578 (0.016)	0.544 (0.021)	0.023 (0.015)	0.001 - 0.064	0.002 (0)	-252.82 (8.64)	0.244 (0.021)	0.2 - 0.288
RG	50	49	0.60	0.40	0.59 (0.015)	0.529 (0.018)	0.014 (0.012)	0 - 0.06	0.002 (0)	-251.52 (9.38)	0.129 (0.018)	0.093 - 0.181
RG	50	49	0.60	0.50	0.598 (0.014)	0.521 (0.018)	0.011 (0.009)	0 - 0.041	0.002 (0)	-250.09 (9.5)	0.023 (0.015)	0 - 0.061
RG	50	49	0.60	0.60	0.605 (0.014)	0.515 (0.016)	0.011 (0.009)	0 - 0.042	0.002 (0)	-251.2 (8.96)	0.085 (0.016)	0.039 - 0.127
RG	50	49	0.60	0.70	0.608 (0.013)	0.511 (0.015)	0.013 (0.009)	0 - 0.036	0.002 (0)	-252.25 (10.3)	0.189 (0.015)	0.156 - 0.226

Continued on next page

Table S1 – *Continued from previous page*

Structure	T	N	p	p_e/p_w	$\bar{\hat{p}}$ (SD)	$\overline{\hat{p}_e}/\overline{\hat{p}_w}$ (SD)	$\overline{\lvert\Delta\rvert}_p$ (SD)	Range $\lvert\Delta\rvert_p$	$\overline{SE}\lvert\Delta\rvert_p$ (SD)	$\overline{BIC}$ (SD)	$\overline{\lvert\Delta\rvert}_{p_e/p_w}$ (SD)	Range $\lvert\Delta\rvert_{p_e/p_w}$
RG	50	49	0.60	0.80	0.612 (0.013)	0.508 (0.016)	0.015 (0.01)	0 - 0.043	0.002 (0)	-250.51 (8.4)	0.292 (0.016)	0.255 - 0.343
RG	50	49	0.60	0.90	0.617 (0.011)	0.505 (0.014)	0.017 (0.01)	0 - 0.038	0.002 (0)	-253.83 (10.26)	0.395 (0.014)	0.357 - 0.428
RG	50	49	0.70	0.10	0.546 (0.094)	0.76 (1.173)	0.154 (0.094)	0.057 - 0.289	0.002 (0)	-263 (13.22)	0.673 (1.165)	0.006 - 8.395
RG	50	49	0.70	0.20	0.642 (0.021)	0.584 (0.016)	0.058 (0.021)	0.012 - 0.108	0.002 (0)	-254.02 (10.49)	0.384 (0.016)	0.339 - 0.428
RG	50	49	0.70	0.30	0.678 (0.015)	0.538 (0.027)	0.023 (0.014)	0 - 0.073	0.002 (0)	-251.08 (10.43)	0.238 (0.027)	0.154 - 0.287
RG	50	49	0.70	0.40	0.69 (0.012)	0.52 (0.035)	0.012 (0.009)	0 - 0.036	0.001 (0)	-249.15 (13.27)	0.12 (0.035)	0.049 - 0.188
RG	50	49	0.70	0.50	0.699 (0.012)	0.508 (0.029)	0.009 (0.007)	0 - 0.037	0.001 (0)	-247.47 (12.62)	0.024 (0.018)	0 - 0.087
RG	50	49	0.70	0.60	0.702 (0.01)	0.503 (0.027)	0.008 (0.006)	0 - 0.024	0.001 (0)	-245.03 (10.76)	0.097 (0.027)	0.034 - 0.147
RG	50	49	0.70	0.70	0.705 (0.01)	0.499 (0.025)	0.009 (0.007)	0 - 0.027	0.001 (0)	-246.15 (11.78)	0.201 (0.025)	0.136 - 0.262
RG	50	49	0.70	0.80	0.704 (0.009)	0.495 (0.02)	0.008 (0.006)	0 - 0.024	0.001 (0)	-245.46 (12.31)	0.305 (0.02)	0.255 - 0.351
RG	50	49	0.70	0.90	0.705 (0.008)	0.494 (0.026)	0.007 (0.006)	0 - 0.027	0.001 (0)	-245.92 (11.04)	0.406 (0.026)	0.312 - 0.456
RG	50	49	0.80	0.10	0.671 (0.066)	0.676 (0.168)	0.129 (0.066)	0.067 - 0.406	0.002 (0)	-271.54 (20.84)	0.581 (0.149)	0.012 - 1.451
RG	50	49	0.80	0.20	0.774 (0.018)	0.617 (0.048)	0.027 (0.017)	0 - 0.068	0.001 (0)	-241.4 (15.24)	0.417 (0.048)	0.24 - 0.496
RG	50	49	0.80	0.30	0.795 (0.01)	0.498 (0.083)	0.009 (0.006)	0 - 0.028	0.001 (0)	-232.83 (11.51)	0.198 (0.083)	0.071 - 0.356
RG	50	49	0.80	0.40	0.801 (0.009)	0.48 (0.081)	0.007 (0.005)	0 - 0.025	0.001 (0)	-229.7 (8.91)	0.084 (0.077)	0 - 0.263
RG	50	49	0.80	0.50	0.802 (0.008)	0.476 (0.076)	0.007 (0.005)	0 - 0.022	0.001 (0)	-230.43 (9.87)	0.069 (0.038)	0 - 0.155
RG	50	49	0.80	0.60	0.801 (0.008)	0.459 (0.066)	0.006 (0.005)	0 - 0.025	0.001 (0)	-229.5 (8.6)	0.142 (0.065)	0.003 - 0.248
RG	50	49	0.80	0.70	0.8 (0.008)	0.47 (0.066)	0.006 (0.005)	0 - 0.02	0.001 (0)	-230.33 (9.33)	0.23 (0.066)	0.075 - 0.338
RG	50	49	0.80	0.80	0.801 (0.008)	0.475 (0.076)	0.006 (0.005)	0 - 0.026	0.001 (0)	-230.7 (9.84)	0.325 (0.076)	0.158 - 0.462
RG	50	49	0.80	0.90	0.802 (0.009)	0.447 (0.069)	0.007 (0.005)	0 - 0.025	0.001 (0)	-229.32 (9.9)	0.453 (0.069)	0.256 - 0.57

Continued on next page

Table S1 – *Continued from previous page*

Structure	T	N	p	p_e/p_w	$\overline{\hat{p}}$ (SD)	$\overline{\hat{p}_e}/\overline{\hat{p}_w}$ (SD)	$\overline{\|\Delta\|}_p$ (SD)	Range $\|\Delta\|_p$	$\overline{SE}\|\Delta\|_p$ (SD)	$\overline{BIC}$ (SD)	$\overline{\|\Delta\|}_{p_e/p_w}$ (SD)	Range $\|\Delta\|_{p_e/p_w}$
RG	50	49	0.90	0.10	0.831 (0.024)	0.769 (0.045)	0.069 (0.024)	0.018 - 0.167	0.001 (0)	-275.28 (22.87)	0.669 (0.045)	0.497 - 0.742
RG	50	49	0.90	0.20	0.895 (0.007)	0.511 (0.148)	0.007 (0.005)	0 - 0.018	0.001 (0)	-204.02 (10.77)	0.311 (0.148)	0.072 - 0.573
RG	50	49	0.90	0.30	0.901 (0.006)	0.415 (0.119)	0.005 (0.004)	0 - 0.017	0.001 (0)	-200.26 (9.11)	0.128 (0.105)	0.001 - 0.418
RG	50	49	0.90	0.40	0.9 (0.006)	0.407 (0.129)	0.005 (0.004)	0 - 0.019	0.001 (0)	-200.85 (8.51)	0.106 (0.074)	0.007 - 0.335
RG	50	49	0.90	0.50	0.901 (0.006)	0.42 (0.129)	0.005 (0.004)	0 - 0.018	0.001 (0)	-201.95 (9.3)	0.127 (0.082)	0.002 - 0.288
RG	50	49	0.90	0.60	0.9 (0.006)	0.421 (0.12)	0.005 (0.004)	0 - 0.018	0.001 (0)	-201.21 (9.84)	0.189 (0.103)	0.007 - 0.36
RG	50	49	0.90	0.70	0.9 (0.007)	0.392 (0.125)	0.005 (0.004)	0 - 0.017	0.001 (0)	-201.23 (8.36)	0.309 (0.123)	0.004 - 0.511
RG	50	49	0.90	0.80	0.902 (0.006)	0.399 (0.121)	0.005 (0.004)	0 - 0.018	0.001 (0)	-200.44 (8.4)	0.401 (0.121)	0.094 - 0.576
RG	50	49	0.90	0.90	0.9 (0.006)	0.41 (0.132)	0.005 (0.004)	0 - 0.017	0.001 (0)	-200.45 (8.94)	0.49 (0.132)	0.165 - 0.697
RG	50	100	0.10	0.10	0.104 (0.006)	0.756 (0.068)	0.006 (0.005)	0 - 0.024	0.001 (0)	-240.03 (10.38)	0.656 (0.068)	0.484 - 0.781
RG	50	100	0.10	0.20	0.1 (0.004)	0.747 (0.08)	0.003 (0.003)	0 - 0.011	0.001 (0)	-237.27 (8.6)	0.547 (0.08)	0.415 - 0.682
RG	50	100	0.10	0.30	0.1 (0.004)	0.75 (0.086)	0.003 (0.003)	0 - 0.014	0.001 (0)	-237.84 (9.82)	0.45 (0.086)	0.245 - 0.601
RG	50	100	0.10	0.40	0.1 (0.004)	0.732 (0.076)	0.003 (0.003)	0 - 0.014	0.001 (0)	-236.2 (9.14)	0.332 (0.076)	0.172 - 0.457
RG	50	100	0.10	0.50	0.1 (0.005)	0.739 (0.079)	0.004 (0.003)	0 - 0.012	0.001 (0)	-236.14 (9.82)	0.239 (0.079)	0.08 - 0.381
RG	50	100	0.10	0.60	0.1 (0.004)	0.744 (0.085)	0.003 (0.003)	0 - 0.014	0.001 (0)	-236.33 (9.38)	0.146 (0.081)	0.004 - 0.281
RG	50	100	0.10	0.70	0.1 (0.005)	0.755 (0.079)	0.004 (0.003)	0 - 0.013	0.001 (0)	-238.23 (9.77)	0.081 (0.051)	0.003 - 0.181
RG	50	100	0.10	0.80	0.1 (0.004)	0.752 (0.083)	0.003 (0.003)	0 - 0.012	0.001 (0)	-237.5 (8.74)	0.077 (0.057)	0.003 - 0.258
RG	50	100	0.10	0.90	0.1 (0.004)	0.751 (0.08)	0.003 (0.002)	0 - 0.012	0.001 (0)	-237.15 (10.09)	0.149 (0.08)	0.019 - 0.334
RG	50	100	0.20	0.10	0.216 (0.018)	0.694 (0.118)	0.018 (0.017)	0 - 0.088	0.001 (0)	-290.55 (27.34)	0.594 (0.118)	0.425 - 0.734
RG	50	100	0.20	0.20	0.201 (0.006)	0.699 (0.112)	0.005 (0.003)	0 - 0.015	0.001 (0)	-264.98 (9.82)	0.499 (0.112)	0.244 - 0.634

Continued on next page

Table S1 – *Continued from previous page*

Structure	T	N	p	p_e/p_w	$\overline{\hat{p}}$ (SD)	$\overline{\hat{p}_e}/\overline{\hat{p}_w}$ (SD)	$\overline{\lvert\Delta\rvert}_p$ (SD)	Range $\lvert\Delta\rvert_p$	$\overline{SE}\lvert\Delta\rvert_p$ (SD)	$\overline{BIC}$ (SD)	$\overline{\lvert\Delta\rvert}_{p_e/p_w}$ (SD)	Range $\lvert\Delta\rvert_{p_e/p_w}$
RG	50	100	0.20	0.30	0.199 (0.006)	0.702 (0.117)	0.005 (0.004)	0 - 0.02	0.001 (0)	-264.95 (8.82)	0.402 (0.117)	0.167 - 0.535
RG	50	100	0.20	0.40	0.199 (0.006)	0.698 (0.116)	0.005 (0.003)	0 - 0.017	0.001 (0)	-266.26 (10.29)	0.298 (0.115)	0.013 - 0.435
RG	50	100	0.20	0.50	0.2 (0.006)	0.667 (0.13)	0.005 (0.003)	0 - 0.015	0.001 (0)	-267.55 (9.89)	0.175 (0.119)	0.002 - 0.334
RG	50	100	0.20	0.60	0.2 (0.005)	0.687 (0.126)	0.004 (0.003)	0 - 0.017	0.001 (0)	-264.58 (9.57)	0.126 (0.088)	0 - 0.231
RG	50	100	0.20	0.70	0.199 (0.006)	0.692 (0.144)	0.005 (0.003)	0 - 0.014	0.001 (0)	-265.52 (9.57)	0.132 (0.056)	0.051 - 0.354
RG	50	100	0.20	0.80	0.2 (0.006)	0.676 (0.128)	0.005 (0.003)	0 - 0.016	0.001 (0)	-265.64 (9.85)	0.133 (0.118)	0 - 0.41
RG	50	100	0.20	0.90	0.199 (0.006)	0.687 (0.124)	0.005 (0.003)	0 - 0.016	0.001 (0)	-264.4 (9.57)	0.213 (0.124)	0.069 - 0.478
RG	50	100	0.30	0.10	0.323 (0.009)	0.633 (0.073)	0.023 (0.009)	0.003 - 0.045	0.001 (0)	-286.07 (11.71)	0.533 (0.073)	0.365 - 0.62
RG	50	100	0.30	0.20	0.311 (0.009)	0.612 (0.08)	0.012 (0.008)	0 - 0.042	0.001 (0)	-285.49 (11.29)	0.412 (0.08)	0.284 - 0.527
RG	50	100	0.30	0.30	0.304 (0.008)	0.613 (0.094)	0.007 (0.005)	0 - 0.024	0.001 (0)	-282.29 (11.77)	0.313 (0.094)	0.086 - 0.539
RG	50	100	0.30	0.40	0.301 (0.007)	0.599 (0.103)	0.006 (0.004)	0 - 0.019	0.001 (0)	-278.91 (9.71)	0.202 (0.096)	0.005 - 0.415
RG	50	100	0.30	0.50	0.299 (0.008)	0.575 (0.108)	0.006 (0.005)	0 - 0.02	0.001 (0)	-277.94 (9.69)	0.097 (0.089)	0.003 - 0.298
RG	50	100	0.30	0.60	0.299 (0.006)	0.593 (0.122)	0.005 (0.004)	0 - 0.014	0.001 (0)	-277.44 (10.7)	0.112 (0.046)	0.037 - 0.263
RG	50	100	0.30	0.70	0.298 (0.007)	0.585 (0.122)	0.006 (0.004)	0 - 0.018	0.001 (0)	-279.7 (8.86)	0.124 (0.112)	0.001 - 0.426
RG	50	100	0.30	0.80	0.299 (0.006)	0.589 (0.135)	0.004 (0.004)	0 - 0.018	0.001 (0)	-279.01 (9.41)	0.212 (0.132)	0.011 - 0.476
RG	50	100	0.30	0.90	0.298 (0.007)	0.59 (0.123)	0.006 (0.004)	0 - 0.017	0.001 (0)	-279.87 (10.59)	0.31 (0.123)	0.056 - 0.569
RG	50	100	0.40	0.10	0.431 (0.009)	0.558 (0.043)	0.031 (0.009)	0.009 - 0.057	0.001 (0)	-286.62 (9.22)	0.458 (0.043)	0.412 - 0.696
RG	50	100	0.40	0.20	0.42 (0.011)	0.525 (0.019)	0.02 (0.01)	0.001 - 0.054	0.001 (0)	-287.59 (11.05)	0.325 (0.019)	0.292 - 0.404
RG	50	100	0.40	0.30	0.413 (0.009)	0.527 (0.022)	0.013 (0.007)	0 - 0.039	0.001 (0)	-286.98 (8.82)	0.227 (0.022)	0.185 - 0.287

Continued on next page

Table S1 – *Continued from previous page*

Structure	T	N	p	p_e/p_w	$\overline{\hat{p}}$ (SD)	$\overline{\hat{p}_e}/\overline{\hat{p}_w}$ (SD)	$\overline{\lvert\Delta\rvert}_p$ (SD)	Range $\lvert\Delta\rvert_p$	$\overline{SE}\lvert\Delta\rvert_p$ (SD)	$\overline{BIC}$ (SD)	$\overline{\lvert\Delta\rvert}_{p_e/p_w}$ (SD)	Range $\lvert\Delta\rvert_{p_e/p_w}$
RG	50	100	0.40	0.40	0.407 (0.01)	0.518 (0.015)	0.01 (0.008)	0 - 0.039	0.001 (0)	-287.37 (9.52)	0.118 (0.015)	0.094 - 0.18
RG	50	100	0.40	0.50	0.402 (0.007)	0.511 (0.014)	0.006 (0.004)	0 - 0.023	0.001 (0)	-284.78 (8.76)	0.014 (0.012)	0.002 - 0.057
RG	50	100	0.40	0.60	0.399 (0.009)	0.516 (0.03)	0.007 (0.005)	0 - 0.033	0.001 (0)	-285.6 (9.43)	0.087 (0.018)	0.016 - 0.127
RG	50	100	0.40	0.70	0.396 (0.008)	0.515 (0.04)	0.007 (0.005)	0 - 0.023	0.001 (0)	-284.79 (9.38)	0.187 (0.03)	0.043 - 0.286
RG	50	100	0.40	0.80	0.394 (0.006)	0.525 (0.051)	0.007 (0.005)	0 - 0.02	0.001 (0)	-287.77 (10.12)	0.275 (0.051)	0.058 - 0.335
RG	50	100	0.40	0.90	0.395 (0.007)	0.516 (0.051)	0.007 (0.005)	0 - 0.027	0.001 (0)	-288.89 (12.98)	0.384 (0.051)	0.187 - 0.493
RG	50	100	0.50	0.10	0.501 (0.015)	0.539 (0.102)	0.013 (0.008)	0 - 0.035	0.001 (0)	-286.41 (9.85)	0.439 (0.102)	0.084 - 0.764
RG	50	100	0.50	0.20	0.5 (0.015)	0.557 (0.093)	0.013 (0.007)	0 - 0.029	0.001 (0)	-286.53 (8.82)	0.357 (0.093)	0.232 - 0.628
RG	50	100	0.50	0.30	0.502 (0.014)	0.56 (0.11)	0.012 (0.007)	0.001 - 0.042	0.001 (0)	-286.89 (9.52)	0.263 (0.102)	0.126 - 0.605
RG	50	100	0.50	0.40	0.5 (0.015)	0.537 (0.112)	0.013 (0.007)	0.002 - 0.033	0.001 (0)	-285.74 (9)	0.153 (0.089)	0.046 - 0.498
RG	50	100	0.50	0.50	0.5 (0.014)	0.559 (0.106)	0.012 (0.007)	0.001 - 0.032	0.001 (0)	-287.38 (9.82)	0.078 (0.093)	0.003 - 0.397
RG	50	100	0.50	0.60	0.501 (0.014)	0.564 (0.133)	0.012 (0.006)	0.001 - 0.03	0.001 (0)	-285.71 (9.3)	0.108 (0.085)	0.002 - 0.527
RG	50	100	0.50	0.70	0.502 (0.014)	0.579 (0.156)	0.012 (0.006)	0.001 - 0.029	0.001 (0)	-288.15 (9.57)	0.174 (0.092)	0.004 - 0.863
RG	50	100	0.50	0.80	0.502 (0.014)	0.582 (0.172)	0.013 (0.006)	0.003 - 0.033	0.001 (0)	-286.38 (9.1)	0.244 (0.132)	0.004 - 0.63
RG	50	100	0.50	0.90	0.499 (0.012)	0.552 (0.135)	0.011 (0.007)	0 - 0.03	0.001 (0)	-286.17 (9.17)	0.348 (0.135)	0.03 - 0.894
RG	50	100	0.60	0.10	0.556 (0.011)	0.567 (0.045)	0.044 (0.011)	0.016 - 0.079	0.001 (0)	-289.96 (9.64)	0.467 (0.045)	0.424 - 0.789
RG	50	100	0.60	0.20	0.569 (0.011)	0.531 (0.013)	0.031 (0.011)	0.008 - 0.067	0.001 (0)	-287.22 (10.76)	0.331 (0.013)	0.289 - 0.367
RG	50	100	0.60	0.30	0.582 (0.012)	0.522 (0.011)	0.018 (0.011)	0 - 0.05	0.001 (0)	-286.91 (9.83)	0.222 (0.011)	0.193 - 0.247
RG	50	100	0.60	0.40	0.591 (0.009)	0.513 (0.009)	0.01 (0.008)	0 - 0.046	0.001 (0)	-286.75 (9.86)	0.113 (0.009)	0.087 - 0.137

Continued on next page

Table S1 – *Continued from previous page*

Structure	T	N	p	p_e/p_w	$\overline{\hat{p}}$ (SD)	$\overline{\hat{p_e}}/\overline{\hat{p_w}}$ (SD)	$\overline{\lvert\Delta\rvert}_p$ (SD)	Range $\lvert\Delta\rvert_p$	$\overline{SE}\lvert\Delta\rvert_p$ (SD)	$\overline{BIC}$ (SD)	$\overline{\lvert\Delta\rvert}_{p_e/p_w}$ (SD)	Range $\lvert\Delta\rvert_{p_e/p_w}$
RG	50	100	0.60	0.50	0.597 (0.009)	0.509 (0.009)	0.007 (0.006)	0 - 0.03	0.001 (0)	-285.32 (10.31)	0.011 (0.007)	0 - 0.029
RG	50	100	0.60	0.60	0.602 (0.009)	0.506 (0.009)	0.008 (0.005)	0 - 0.028	0.001 (0)	-286.59 (9.2)	0.094 (0.009)	0.071 - 0.116
RG	50	100	0.60	0.70	0.606 (0.007)	0.505 (0.01)	0.008 (0.005)	0 - 0.022	0.001 (0)	-286.78 (9.71)	0.195 (0.01)	0.166 - 0.214
RG	50	100	0.60	0.80	0.608 (0.007)	0.503 (0.01)	0.009 (0.006)	0 - 0.024	0.001 (0)	-287.26 (12.52)	0.297 (0.01)	0.268 - 0.325
RG	50	100	0.60	0.90	0.608 (0.007)	0.499 (0.011)	0.009 (0.006)	0 - 0.026	0.001 (0)	-290.16 (11.41)	0.401 (0.011)	0.373 - 0.426
RG	50	100	0.70	0.10	0.623 (0.018)	0.571 (0.012)	0.077 (0.018)	0.032 - 0.127	0.001 (0)	-300.38 (14.81)	0.471 (0.012)	0.443 - 0.496
RG	50	100	0.70	0.20	0.68 (0.012)	0.533 (0.039)	0.02 (0.011)	0 - 0.049	0.001 (0)	-287.67 (12.09)	0.333 (0.039)	0.231 - 0.399
RG	50	100	0.70	0.30	0.695 (0.008)	0.515 (0.043)	0.007 (0.006)	0 - 0.033	0.001 (0)	-280.09 (10.67)	0.215 (0.043)	0.13 - 0.285
RG	50	100	0.70	0.40	0.698 (0.006)	0.499 (0.039)	0.005 (0.004)	0 - 0.016	0.001 (0)	-278.97 (10.72)	0.099 (0.039)	0.013 - 0.185
RG	50	100	0.70	0.50	0.702 (0.006)	0.499 (0.044)	0.005 (0.004)	0 - 0.014	0.001 (0)	-277.73 (10.27)	0.036 (0.025)	0 - 0.097
RG	50	100	0.70	0.60	0.702 (0.007)	0.488 (0.043)	0.006 (0.005)	0 - 0.018	0.001 (0)	-278.39 (9)	0.112 (0.043)	0.017 - 0.196
RG	50	100	0.70	0.70	0.701 (0.007)	0.497 (0.042)	0.006 (0.004)	0 - 0.023	0.001 (0)	-279.9 (10.57)	0.203 (0.042)	0.091 - 0.293
RG	50	100	0.70	0.80	0.701 (0.007)	0.489 (0.041)	0.006 (0.004)	0 - 0.016	0.001 (0)	-281.26 (8.87)	0.311 (0.041)	0.206 - 0.397
RG	50	100	0.70	0.90	0.702 (0.006)	0.484 (0.039)	0.005 (0.004)	0 - 0.017	0.001 (0)	-278.36 (9.08)	0.416 (0.039)	0.317 - 0.489
RG	50	100	0.80	0.10	0.78 (0.011)	0.568 (0.117)	0.02 (0.01)	0.001 - 0.043	0.001 (0)	-276.75 (12.1)	0.468 (0.117)	0.262 - 0.616
RG	50	100	0.80	0.20	0.797 (0.006)	0.437 (0.069)	0.005 (0.004)	0 - 0.018	0.001 (0)	-267.17 (9.07)	0.237 (0.069)	0.125 - 0.464
RG	50	100	0.80	0.30	0.801 (0.006)	0.444 (0.082)	0.005 (0.004)	0 - 0.017	0.001 (0)	-264.41 (9.61)	0.144 (0.082)	0 - 0.362
RG	50	100	0.80	0.40	0.8 (0.005)	0.437 (0.067)	0.004 (0.003)	0 - 0.012	0.001 (0)	-265.43 (10.08)	0.057 (0.051)	0.002 - 0.266

Continued on next page

Table S1 – *Continued from previous page*

Structure	T	N	p	p_e/p_w	$\bar{\hat{p}}$ (SD)	$\overline{\hat{p}_e}/\overline{\hat{p}_w}$ (SD)	$\overline{\lvert\Delta\rvert}_p$ (SD)	Range $\lvert\Delta\rvert_p$	$\overline{SE}\lvert\Delta\rvert_p$ (SD)	$\overline{BIC}$ (SD)	$\overline{\lvert\Delta\rvert}_{p_e/p_w}$ (SD)	Range $\lvert\Delta\rvert_{p_e/p_w}$
RG	50	100	0.80	0.50	0.801 (0.006)	0.446 (0.084)	0.005 (0.004)	0 - 0.016	0.001 (0)	-265.33 (10.46)	0.085 (0.052)	0.001 - 0.216
RG	50	100	0.80	0.60	0.802 (0.005)	0.436 (0.07)	0.004 (0.004)	0 - 0.016	0.001 (0)	-266.31 (9.54)	0.168 (0.061)	0.011 - 0.306
RG	50	100	0.80	0.70	0.8 (0.005)	0.432 (0.089)	0.004 (0.003)	0 - 0.018	0.001 (0)	-264.75 (9.24)	0.268 (0.089)	0.023 - 0.39
RG	50	100	0.80	0.80	0.8 (0.005)	0.434 (0.07)	0.004 (0.003)	0 - 0.016	0.001 (0)	-267.12 (10.15)	0.366 (0.07)	0.141 - 0.478
RG	50	100	0.80	0.90	0.8 (0.006)	0.446 (0.07)	0.005 (0.004)	0 - 0.02	0.001 (0)	-265.44 (9.71)	0.454 (0.07)	0.227 - 0.595
RG	50	100	0.90	0.10	0.896 (0.005)	0.392 (0.119)	0.005 (0.003)	0 - 0.015	0.001 (0)	-239.92 (9.42)	0.292 (0.119)	0.11 - 0.521
RG	50	100	0.90	0.20	0.9 (0.005)	0.388 (0.122)	0.004 (0.002)	0 - 0.01	0.001 (0)	-238.37 (11.22)	0.188 (0.122)	0 - 0.416
RG	50	100	0.90	0.30	0.9 (0.004)	0.378 (0.122)	0.003 (0.003)	0 - 0.011	0.001 (0)	-236.83 (9.47)	0.125 (0.073)	0.012 - 0.311
RG	50	100	0.90	0.40	0.901 (0.004)	0.37 (0.121)	0.004 (0.003)	0 - 0.01	0.001 (0)	-237.29 (8.8)	0.105 (0.066)	0.001 - 0.24
RG	50	100	0.90	0.50	0.9 (0.005)	0.396 (0.119)	0.004 (0.003)	0 - 0.013	0.001 (0)	-237.86 (8.97)	0.119 (0.103)	0 - 0.308
RG	50	100	0.90	0.60	0.9 (0.004)	0.376 (0.119)	0.003 (0.003)	0 - 0.011	0.001 (0)	-235.92 (9.63)	0.225 (0.119)	0.002 - 0.408
RG	50	100	0.90	0.70	0.9 (0.004)	0.368 (0.122)	0.003 (0.003)	0 - 0.011	0.001 (0)	-237.62 (10.18)	0.332 (0.122)	0.09 - 0.513
RG	50	100	0.90	0.80	0.901 (0.004)	0.35 (0.122)	0.003 (0.003)	0 - 0.011	0.001 (0)	-237.68 (9.55)	0.45 (0.122)	0.179 - 0.613
RG	50	100	0.90	0.90	0.9 (0.005)	0.39 (0.13)	0.004 (0.002)	0 - 0.011	0.001 (0)	-237.07 (9.26)	0.51 (0.13)	0.282 - 0.712
RG	100	16	0.10	0.10	0.13 (0.011)	0.776 (0.05)	0.03 (0.011)	0.003 - 0.059	0.001 (0)	-326.55 (16.63)	0.676 (0.05)	0.39 - 0.725
RG	100	16	0.10	0.20	0.111 (0.009)	0.787 (0.052)	0.012 (0.008)	0 - 0.033	0.001 (0)	-314.87 (19.52)	0.587 (0.052)	0.294 - 0.769
RG	100	16	0.10	0.30	0.118 (0.011)	0.789 (0.042)	0.019 (0.01)	0 - 0.046	0.001 (0)	-326.08 (25.02)	0.489 (0.042)	0.274 - 0.645
RG	100	16	0.10	0.40	0.106 (0.01)	0.786 (0.082)	0.009 (0.008)	0 - 0.035	0.001 (0)	-327.75 (43.56)	0.386 (0.082)	0.119 - 0.61
RG	100	16	0.10	0.50	0.096 (0.016)	0.768 (0.082)	0.01 (0.013)	0 - 0.075	0.001 (0)	-332.19 (43.94)	0.268 (0.082)	0.16 - 0.444
RG	100	16	0.10	0.60	0.085 (0.025)	0.767 (0.093)	0.018 (0.022)	0 - 0.075	0.001 (0)	-337.86 (46.17)	0.17 (0.088)	0.081 - 0.345
RG	100	16	0.10	0.70	0.086 (0.02)	0.772 (0.1)	0.016 (0.018)	0 - 0.075	0.001 (0)	-331.95 (43.44)	0.081 (0.093)	0 - 0.305

Continued on next page

Table S1 – *Continued from previous page*

Structure	T	N	p	p_e/p_w	$\overline{\hat{p}}$ (SD)	$\overline{\hat{p_e}}/\overline{\hat{p_w}}$ (SD)	$\overline{\lvert\Delta\rvert}_p$ (SD)	Range $\lvert\Delta\rvert_p$	$\overline{SE}\lvert\Delta\rvert_p$ (SD)	$\overline{BIC}$ (SD)	$\overline{\lvert\Delta\rvert}_{p_e/p_w}$ (SD)	Range $\lvert\Delta\rvert_{p_e/p_w}$
RG	100	16	0.10	0.80	0.089 (0.021)	0.788 (0.102)	0.014 (0.019)	0 - 0.075	0.001 (0)	-332.05 (48.82)	0.089 (0.049)	0.002 - 0.28
RG	100	16	0.10	0.90	0.089 (0.021)	0.775 (0.092)	0.015 (0.019)	0 - 0.075	0.001 (0)	-337.88 (48.38)	0.134 (0.08)	0.002 - 0.338
RG	100	16	0.20	0.10	0.245 (0.013)	0.693 (0.059)	0.045 (0.013)	0.006 - 0.079	0.001 (0)	-375.34 (13.27)	0.593 (0.059)	0.41 - 0.766
RG	100	16	0.20	0.20	0.221 (0.011)	0.712 (0.042)	0.021 (0.011)	0 - 0.045	0.001 (0)	-373.11 (13.65)	0.512 (0.042)	0.312 - 0.7
RG	100	16	0.20	0.30	0.23 (0.011)	0.682 (0.064)	0.03 (0.011)	0.001 - 0.06	0.001 (0)	-374.11 (16.32)	0.382 (0.064)	0.21 - 0.458
RG	100	16	0.20	0.40	0.212 (0.013)	0.715 (0.064)	0.015 (0.01)	0 - 0.044	0.001 (0)	-370.68 (16.11)	0.315 (0.064)	0.117 - 0.505
RG	100	16	0.20	0.50	0.209 (0.013)	0.7 (0.055)	0.013 (0.009)	0 - 0.038	0.001 (0)	-376.27 (22.57)	0.2 (0.055)	0.03 - 0.348
RG	100	16	0.20	0.60	0.203 (0.012)	0.714 (0.074)	0.01 (0.008)	0 - 0.026	0.001 (0)	-378.14 (29.94)	0.116 (0.07)	0.016 - 0.396
RG	100	16	0.20	0.70	0.201 (0.014)	0.713 (0.085)	0.011 (0.008)	0.001 - 0.042	0.001 (0)	-382.23 (34.29)	0.061 (0.059)	0.005 - 0.262
RG	100	16	0.20	0.80	0.199 (0.013)	0.714 (0.091)	0.01 (0.008)	0 - 0.05	0.001 (0)	-386.48 (35.07)	0.105 (0.067)	0.002 - 0.283
RG	100	16	0.20	0.90	0.197 (0.016)	0.703 (0.077)	0.012 (0.011)	0.001 - 0.051	0.001 (0)	-391.12 (38.48)	0.199 (0.073)	0.023 - 0.361
RG	100	16	0.30	0.10	0.341 (0.014)	0.674 (0.07)	0.041 (0.014)	0.007 - 0.073	0.001 (0)	-398.73 (14.28)	0.574 (0.07)	0.471 - 0.98
RG	100	16	0.30	0.20	0.326 (0.015)	0.664 (0.036)	0.027 (0.013)	0 - 0.069	0.001 (0)	-395.02 (14.07)	0.464 (0.036)	0.37 - 0.746
RG	100	16	0.30	0.30	0.334 (0.013)	0.649 (0.026)	0.035 (0.012)	0.007 - 0.077	0.001 (0)	-395.15 (12.85)	0.349 (0.026)	0.228 - 0.431
RG	100	16	0.30	0.40	0.321 (0.014)	0.649 (0.031)	0.022 (0.012)	0.001 - 0.055	0.001 (0)	-399.27 (15.31)	0.249 (0.031)	0.124 - 0.321
RG	100	16	0.30	0.50	0.319 (0.013)	0.633 (0.041)	0.02 (0.011)	0 - 0.047	0.001 (0)	-400.81 (16.81)	0.133 (0.041)	0.007 - 0.171
RG	100	16	0.30	0.60	0.312 (0.014)	0.645 (0.038)	0.016 (0.009)	0 - 0.046	0.001 (0)	-401.81 (17.98)	0.05 (0.031)	0.001 - 0.239
RG	100	16	0.30	0.70	0.309 (0.016)	0.647 (0.027)	0.016 (0.009)	0 - 0.043	0.001 (0)	-400.39 (16.28)	0.053 (0.027)	0.021 - 0.185

Continued on next page

Table S1 – *Continued from previous page*

Structure	T	N	p	p_e/p_w	$\overline{\hat{p}}$ (SD)	$\overline{\hat{p_e}}/\overline{\hat{p_w}}$ (SD)	$\overline{\lvert\Delta\rvert}_p$ (SD)	Range $\lvert\Delta\rvert_p$	$\overline{SE}\lvert\Delta\rvert_p$ (SD)	$\overline{BIC}$ (SD)	$\overline{\lvert\Delta\rvert}_{p_e/p_w}$ (SD)	Range $\lvert\Delta\rvert_{p_e/p_w}$
RG	100	16	0.30	0.80	0.303 (0.012)	0.65 (0.027)	0.01 (0.007)	0 - 0.03	0.001 (0)	-400.53 (18.59)	0.15 (0.027)	0.079 - 0.225
RG	100	16	0.30	0.90	0.303 (0.013)	0.643 (0.029)	0.01 (0.008)	0 - 0.036	0.001 (0)	-409.01 (19.68)	0.257 (0.029)	0.164 - 0.322
RG	100	16	0.40	0.10	0.419 (0.013)	0.658 (0.138)	0.019 (0.013)	0 - 0.07	0.001 (0)	-406.45 (14.92)	0.558 (0.138)	0.474 - 1.319
RG	100	16	0.40	0.20	0.417 (0.014)	0.632 (0.075)	0.019 (0.012)	0 - 0.059	0.001 (0)	-405.33 (12.92)	0.432 (0.075)	0.357 - 0.827
RG	100	16	0.40	0.30	0.419 (0.016)	0.614 (0.066)	0.021 (0.014)	0 - 0.067	0.002 (0)	-406.91 (14.1)	0.314 (0.066)	0.165 - 0.627
RG	100	16	0.40	0.40	0.411 (0.014)	0.591 (0.031)	0.014 (0.011)	0 - 0.064	0.002 (0)	-406.68 (16.21)	0.191 (0.031)	0.113 - 0.284
RG	100	16	0.40	0.50	0.408 (0.015)	0.578 (0.036)	0.013 (0.01)	0 - 0.047	0.002 (0)	-405.69 (14.66)	0.079 (0.032)	0.01 - 0.159
RG	100	16	0.40	0.60	0.405 (0.018)	0.57 (0.042)	0.015 (0.011)	0 - 0.05	0.002 (0)	-406.23 (13.89)	0.035 (0.038)	0 - 0.157
RG	100	16	0.40	0.70	0.4 (0.015)	0.586 (0.029)	0.013 (0.009)	0 - 0.035	0.002 (0)	-407.07 (15.29)	0.114 (0.029)	0.027 - 0.187
RG	100	16	0.40	0.80	0.398 (0.013)	0.58 (0.034)	0.011 (0.008)	0 - 0.032	0.002 (0)	-407.51 (14.08)	0.22 (0.034)	0.127 - 0.341
RG	100	16	0.40	0.90	0.396 (0.014)	0.569 (0.044)	0.012 (0.008)	0 - 0.033	0.002 (0)	-408.03 (12.89)	0.331 (0.044)	0.281 - 0.453
RG	100	16	0.50	0.10	0.505 (0.022)	0.572 (0.163)	0.02 (0.01)	0.001 - 0.058	0.002 (0)	-407.4 (14.18)	0.473 (0.158)	0.093 - 0.913
RG	100	16	0.50	0.20	0.498 (0.024)	0.667 (0.933)	0.021 (0.011)	0.003 - 0.054	0.002 (0)	-406.89 (12.91)	0.47 (0.931)	0.111 - 9.584
RG	100	16	0.50	0.30	0.498 (0.023)	0.586 (0.281)	0.02 (0.011)	0.003 - 0.05	0.002 (0)	-407.1 (12.63)	0.286 (0.281)	0.019 - 2.856
RG	100	16	0.50	0.40	0.5 (0.023)	0.59 (0.17)	0.021 (0.01)	0 - 0.045	0.002 (0)	-405.18 (13.18)	0.203 (0.155)	0.012 - 0.782
RG	100	16	0.50	0.50	0.501 (0.022)	0.571 (0.15)	0.02 (0.011)	0 - 0.046	0.002 (0)	-407.35 (15.3)	0.11 (0.124)	0 - 0.543
RG	100	16	0.50	0.60	0.502 (0.022)	0.579 (0.206)	0.019 (0.011)	0.003 - 0.054	0.002 (0)	-406.76 (13.18)	0.12 (0.169)	0 - 1.369
RG	100	16	0.50	0.70	0.501 (0.024)	0.572 (0.132)	0.021 (0.011)	0.004 - 0.055	0.002 (0)	-407.38 (13.79)	0.162 (0.087)	0.006 - 0.424
RG	100	16	0.50	0.80	0.504 (0.023)	0.562 (0.125)	0.02 (0.012)	0.001 - 0.069	0.002 (0)	-408.18 (13.96)	0.249 (0.099)	0.016 - 0.777
RG	100	16	0.50	0.90	0.501 (0.021)	0.588 (0.178)	0.018 (0.011)	0.001 - 0.058	0.002 (0)	-407.04 (14.22)	0.334 (0.13)	0.009 - 0.895

Continued on next page

Table S1 – *Continued from previous page*

Structure	T	N	p	p_e/p_w	$\overline{\hat{p}}$ (SD)	$\overline{\hat{p}_e}/\overline{\hat{p}_w}$ (SD)	$\overline{\lvert\Delta\rvert}_p$ (SD)	Range $\lvert\Delta\rvert_p$	$\overline{SE}\lvert\Delta\rvert_p$ (SD)	$\overline{BIC}$ (SD)	$\overline{\lvert\Delta\rvert}_{p_e/p_w}$ (SD)	Range $\lvert\Delta\rvert_{p_e/p_w}$
RG	100	16	0.60	0.10	0.548 (0.041)	0.795 (0.796)	0.052 (0.041)	0.004 - 0.176	0.002 (0)	-414.12 (19.15)	0.695 (0.795)	0.026 - 5.812
RG	100	16	0.60	0.20	0.568 (0.025)	0.661 (0.206)	0.032 (0.025)	0.002 - 0.161	0.002 (0)	-411.12 (13.64)	0.463 (0.201)	0.029 - 2.082
RG	100	16	0.60	0.30	0.571 (0.019)	0.643 (0.101)	0.03 (0.018)	0.001 - 0.073	0.002 (0)	-407.31 (13.77)	0.343 (0.101)	0.2 - 0.844
RG	100	16	0.60	0.40	0.584 (0.018)	0.587 (0.06)	0.019 (0.014)	0.001 - 0.063	0.002 (0)	-409.23 (16.32)	0.187 (0.06)	0.11 - 0.345
RG	100	16	0.60	0.50	0.596 (0.019)	0.547 (0.034)	0.015 (0.012)	0 - 0.055	0.002 (0)	-405.36 (11.81)	0.047 (0.033)	0.01 - 0.15
RG	100	16	0.60	0.60	0.603 (0.018)	0.543 (0.033)	0.015 (0.011)	0 - 0.05	0.002 (0)	-407.83 (13.82)	0.059 (0.028)	0.001 - 0.147
RG	100	16	0.60	0.70	0.61 (0.019)	0.545 (0.03)	0.018 (0.012)	0 - 0.044	0.002 (0)	-405.54 (13.57)	0.155 (0.03)	0.076 - 0.217
RG	100	16	0.60	0.80	0.62 (0.017)	0.541 (0.023)	0.022 (0.014)	0 - 0.061	0.002 (0)	-406.19 (12.94)	0.259 (0.023)	0.181 - 0.31
RG	100	16	0.60	0.90	0.624 (0.017)	0.532 (0.019)	0.026 (0.015)	0 - 0.057	0.002 (0)	-407.96 (13.65)	0.368 (0.019)	0.288 - 0.427
RG	100	16	0.70	0.10	0.468 (0.065)	0.584 (0.341)	0.232 (0.065)	0.04 - 0.339	0.002 (0)	-467.13 (34.43)	0.484 (0.34)	0.01 - 3.671
RG	100	16	0.70	0.20	0.621 (0.042)	0.704 (0.257)	0.079 (0.042)	0.017 - 0.292	0.002 (0)	-415.38 (25.42)	0.505 (0.255)	0.05 - 2.82
RG	100	16	0.70	0.30	0.63 (0.024)	0.65 (0.07)	0.07 (0.024)	0.015 - 0.114	0.002 (0)	-408.75 (21)	0.35 (0.07)	0.212 - 0.496
RG	100	16	0.70	0.40	0.669 (0.019)	0.584 (0.036)	0.031 (0.018)	0.001 - 0.074	0.002 (0)	-406.45 (16.03)	0.184 (0.036)	0.111 - 0.293
RG	100	16	0.70	0.50	0.69 (0.017)	0.549 (0.022)	0.016 (0.012)	0 - 0.072	0.002 (0)	-401.64 (14.3)	0.049 (0.022)	0.012 - 0.119
RG	100	16	0.70	0.60	0.71 (0.018)	0.549 (0.024)	0.016 (0.012)	0 - 0.05	0.002 (0)	-396.87 (14.55)	0.053 (0.02)	0.001 - 0.087
RG	100	16	0.70	0.70	0.711 (0.014)	0.544 (0.014)	0.015 (0.01)	0 - 0.04	0.002 (0)	-397.71 (15.24)	0.156 (0.014)	0.097 - 0.19
RG	100	16	0.70	0.80	0.716 (0.013)	0.537 (0.017)	0.017 (0.012)	0 - 0.055	0.001 (0)	-395.19 (13.16)	0.263 (0.017)	0.208 - 0.337
RG	100	16	0.70	0.90	0.724 (0.013)	0.523 (0.025)	0.024 (0.012)	0 - 0.047	0.001 (0)	-399.54 (13.72)	0.377 (0.025)	0.341 - 0.446
RG	100	16	0.80	0.10	0.353 (0.024)	0.402 (0.182)	0.447 (0.024)	0.359 - 0.483	0.002 (0)	-467.06 (58.55)	0.323 (0.14)	0.017 - 0.446

Continued on next page

Table S1 – *Continued from previous page*

Structure	T	N	p	p_e/p_w	$\overline{\hat{p}}$ (SD)	$\overline{\hat{p_e}}/\overline{\hat{p_w}}$ (SD)	$\overline{\lvert\Delta\rvert}_p$ (SD)	Range $\lvert\Delta\rvert_p$	$\overline{SE}\lvert\Delta\rvert_p$ (SD)	$\overline{BIC}$ (SD)	$\overline{\lvert\Delta\rvert}_{p_e/p_w}$ (SD)	Range $\lvert\Delta\rvert_{p_e/p_w}$
RG	100	16	0.80	0.20	0.677 (0.045)	0.803 (1.028)	0.123 (0.045)	0.02 - 0.263	0.002 (0)	-423.31 (45.47)	0.603 (1.028)	0.31 - 10.737
RG	100	16	0.80	0.30	0.701 (0.026)	0.649 (0.059)	0.099 (0.026)	0.045 - 0.179	0.002 (0)	-402.9 (29.1)	0.349 (0.059)	0.21 - 0.451
RG	100	16	0.80	0.40	0.764 (0.018)	0.596 (0.026)	0.036 (0.018)	0.004 - 0.112	0.001 (0)	-393.14 (16.06)	0.196 (0.026)	0.119 - 0.277
RG	100	16	0.80	0.50	0.794 (0.012)	0.537 (0.03)	0.01 (0.008)	0 - 0.033	0.001 (0)	-372.28 (14.05)	0.041 (0.025)	0.009 - 0.113
RG	100	16	0.80	0.60	0.804 (0.011)	0.534 (0.023)	0.01 (0.006)	0 - 0.028	0.001 (0)	-370.24 (14.66)	0.066 (0.022)	0.003 - 0.131
RG	100	16	0.80	0.70	0.807 (0.01)	0.523 (0.039)	0.009 (0.007)	0 - 0.036	0.001 (0)	-369.16 (13.28)	0.177 (0.039)	0.093 - 0.308
RG	100	16	0.80	0.80	0.808 (0.009)	0.495 (0.049)	0.01 (0.007)	0 - 0.028	0.001 (0)	-370.91 (14.18)	0.305 (0.049)	0.244 - 0.409
RG	100	16	0.80	0.90	0.811 (0.01)	0.474 (0.054)	0.012 (0.008)	0 - 0.034	0.001 (0)	-372.15 (15.69)	0.426 (0.054)	0.343 - 0.517
RG	100	16	0.90	0.10	0.298 (0.012)	0.162 (0.15)	0.602 (0.012)	0.513 - 0.624	0.001 (0)	-390.3 (46.12)	0.117 (0.112)	0.01 - 0.506
RG	100	16	0.90	0.20	0.758 (0.043)	0.705 (0.084)	0.142 (0.043)	0.069 - 0.307	0.001 (0)	-428.67 (61.27)	0.505 (0.084)	0.31 - 0.63
RG	100	16	0.90	0.30	0.796 (0.031)	0.671 (0.06)	0.104 (0.031)	0.055 - 0.212	0.001 (0)	-390.87 (36.23)	0.371 (0.06)	0.212 - 0.454
RG	100	16	0.90	0.40	0.876 (0.009)	0.545 (0.065)	0.024 (0.009)	0.001 - 0.056	0.001 (0)	-337.28 (15.02)	0.145 (0.065)	0.047 - 0.294
RG	100	16	0.90	0.50	0.897 (0.009)	0.458 (0.043)	0.007 (0.007)	0 - 0.028	0.001 (0)	-305.36 (15.42)	0.053 (0.029)	0.017 - 0.128
RG	100	16	0.90	0.60	0.899 (0.009)	0.438 (0.037)	0.007 (0.005)	0 - 0.023	0.001 (0)	-300.1 (15.48)	0.162 (0.037)	0.065 - 0.22
RG	100	16	0.90	0.70	0.901 (0.007)	0.441 (0.043)	0.005 (0.004)	0 - 0.017	0.001 (0)	-298.46 (12.8)	0.259 (0.043)	0.112 - 0.342
RG	100	16	0.90	0.80	0.902 (0.007)	0.424 (0.039)	0.006 (0.005)	0 - 0.02	0.001 (0)	-297.27 (15.73)	0.376 (0.039)	0.256 - 0.485
RG	100	16	0.90	0.90	0.901 (0.007)	0.429 (0.046)	0.006 (0.004)	0 - 0.02	0.001 (0)	-296.3 (13.61)	0.471 (0.046)	0.366 - 0.57
RG	100	25	0.10	0.10	0.113 (0.008)	0.746 (0.095)	0.014 (0.008)	0 - 0.034	0.001 (0)	-363.51 (19.94)	0.646 (0.095)	0.457 - 0.77
RG	100	25	0.10	0.20	0.104 (0.008)	0.729 (0.123)	0.007 (0.006)	0 - 0.028	0.001 (0)	-360.37 (23.91)	0.529 (0.123)	0.302 - 0.67

Continued on next page

Table S1 – *Continued from previous page*

Structure	T	N	p	p_e/p_w	$\overline{\hat{p}}$ (SD)	$\overline{\hat{p}_e}/\overline{\hat{p}_w}$ (SD)	$\overline{\lvert\Delta\rvert}_p$ (SD)	Range $\lvert\Delta\rvert_p$	$\overline{SE}\lvert\Delta\rvert_p$ (SD)	$\overline{BIC}$ (SD)	$\overline{\lvert\Delta\rvert}_{p_e/p_w}$ (SD)	Range $\lvert\Delta\rvert_{p_e/p_w}$
RG	100	25	0.10	0.30	0.103 (0.007)	0.731 (0.111)	0.006 (0.005)	0 - 0.022	0.001 (0)	-355.41 (18.42)	0.431 (0.111)	0.23 - 0.57
RG	100	25	0.10	0.40	0.102 (0.008)	0.701 (0.129)	0.006 (0.005)	0 - 0.029	0.001 (0)	-351.18 (16.67)	0.301 (0.129)	0.106 - 0.485
RG	100	25	0.10	0.50	0.1 (0.007)	0.676 (0.158)	0.006 (0.004)	0 - 0.019	0.001 (0)	-343.97 (12.82)	0.194 (0.135)	0 - 0.37
RG	100	25	0.10	0.60	0.099 (0.006)	0.69 (0.166)	0.005 (0.004)	0 - 0.017	0.001 (0)	-344.59 (13.23)	0.159 (0.1)	0.003 - 0.365
RG	100	25	0.10	0.70	0.099 (0.006)	0.678 (0.17)	0.005 (0.003)	0 - 0.017	0.001 (0)	-344.26 (13.33)	0.158 (0.064)	0.064 - 0.432
RG	100	25	0.10	0.80	0.099 (0.006)	0.663 (0.169)	0.005 (0.003)	0 - 0.015	0.001 (0)	-346.19 (13.62)	0.177 (0.126)	0.01 - 0.554
RG	100	25	0.10	0.90	0.099 (0.007)	0.684 (0.176)	0.006 (0.004)	0 - 0.016	0.001 (0)	-343.24 (12.94)	0.216 (0.176)	0.013 - 0.659
RG	100	25	0.20	0.10	0.227 (0.011)	0.644 (0.115)	0.027 (0.01)	0.004 - 0.05	0.001 (0)	-418.25 (14.94)	0.544 (0.115)	0.355 - 0.904
RG	100	25	0.20	0.20	0.21 (0.009)	0.711 (0.106)	0.011 (0.008)	0 - 0.034	0.001 (0)	-411.74 (16.17)	0.511 (0.106)	0.303 - 0.678
RG	100	25	0.20	0.30	0.207 (0.01)	0.695 (0.099)	0.009 (0.007)	0 - 0.033	0.001 (0)	-411.9 (16.08)	0.395 (0.099)	0.195 - 0.535
RG	100	25	0.20	0.40	0.205 (0.009)	0.693 (0.114)	0.008 (0.006)	0 - 0.028	0.001 (0)	-407.66 (15.65)	0.293 (0.114)	0.097 - 0.431
RG	100	25	0.20	0.50	0.201 (0.01)	0.708 (0.118)	0.008 (0.006)	0 - 0.028	0.001 (0)	-411.84 (15.55)	0.208 (0.118)	0.009 - 0.338
RG	100	25	0.20	0.60	0.201 (0.008)	0.649 (0.146)	0.006 (0.005)	0 - 0.021	0.001 (0)	-407.04 (13.9)	0.135 (0.071)	0.001 - 0.238
RG	100	25	0.20	0.70	0.2 (0.008)	0.635 (0.153)	0.007 (0.005)	0 - 0.023	0.001 (0)	-408.26 (14.98)	0.154 (0.062)	0.054 - 0.331
RG	100	25	0.20	0.80	0.198 (0.008)	0.578 (0.195)	0.007 (0.005)	0 - 0.023	0.001 (0)	-407 (14.9)	0.23 (0.186)	0 - 0.717
RG	100	25	0.20	0.90	0.2 (0.008)	0.622 (0.225)	0.007 (0.005)	0 - 0.021	0.001 (0)	-407.46 (13.89)	0.278 (0.225)	0.062 - 0.843
RG	100	25	0.30	0.10	0.333 (0.013)	0.638 (0.052)	0.034 (0.012)	0.002 - 0.062	0.001 (0)	-442.91 (15.35)	0.538 (0.052)	0.434 - 0.856
RG	100	25	0.30	0.20	0.317 (0.012)	0.62 (0.055)	0.017 (0.011)	0.001 - 0.049	0.001 (0)	-440.42 (15.22)	0.42 (0.055)	0.327 - 0.622
RG	100	25	0.30	0.30	0.314 (0.012)	0.609 (0.051)	0.016 (0.009)	0 - 0.036	0.001 (0)	-438.88 (13.4)	0.309 (0.051)	0.186 - 0.409

Continued on next page

Table S1 – *Continued from previous page*

Structure	T	N	p	p_e/p_w	$\overline{\hat{p}}$ (SD)	$\overline{\hat{p_e}}/\overline{\hat{p_w}}$ (SD)	$\overline{\lvert\Delta\rvert}_p$ (SD)	Range $\lvert\Delta\rvert_p$	$\overline{SE}\lvert\Delta\rvert_p$ (SD)	$\overline{BIC}$ (SD)	$\overline{\lvert\Delta\rvert}_{p_e/p_w}$ (SD)	Range $\lvert\Delta\rvert_{p_e/p_w}$
RG	100	25	0.30	0.40	0.31 (0.012)	0.586 (0.053)	0.013 (0.009)	0 - 0.038	0.001 (0)	-439.11 (13.99)	0.186 (0.053)	0.103 - 0.339
RG	100	25	0.30	0.50	0.304 (0.012)	0.593 (0.05)	0.01 (0.007)	0 - 0.037	0.001 (0)	-438.63 (13.37)	0.093 (0.05)	0.001 - 0.212
RG	100	25	0.30	0.60	0.301 (0.011)	0.583 (0.055)	0.008 (0.007)	0 - 0.026	0.001 (0)	-437.7 (14.93)	0.044 (0.036)	0 - 0.137
RG	100	25	0.30	0.70	0.296 (0.01)	0.58 (0.05)	0.009 (0.007)	0 - 0.027	0.001 (0)	-437.07 (14.62)	0.121 (0.049)	0.006 - 0.215
RG	100	25	0.30	0.80	0.297 (0.011)	0.595 (0.063)	0.009 (0.007)	0 - 0.03	0.001 (0)	-438.77 (17.58)	0.205 (0.063)	0.062 - 0.318
RG	100	25	0.30	0.90	0.294 (0.01)	0.573 (0.068)	0.009 (0.007)	0 - 0.027	0.001 (0)	-445.27 (22.14)	0.327 (0.068)	0.159 - 0.452
RG	100	25	0.40	0.10	0.421 (0.014)	0.656 (0.093)	0.022 (0.013)	0 - 0.05	0.001 (0)	-448.6 (12.75)	0.556 (0.093)	0.447 - 0.918
RG	100	25	0.40	0.20	0.414 (0.012)	0.605 (0.059)	0.015 (0.01)	0 - 0.054	0.001 (0)	-451.25 (14.98)	0.405 (0.059)	0.32 - 0.824
RG	100	25	0.40	0.30	0.413 (0.013)	0.599 (0.036)	0.015 (0.01)	0 - 0.051	0.001 (0)	-449.87 (14.33)	0.299 (0.036)	0.236 - 0.463
RG	100	25	0.40	0.40	0.415 (0.011)	0.575 (0.025)	0.016 (0.009)	0 - 0.046	0.001 (0)	-450.28 (15.05)	0.175 (0.025)	0.094 - 0.235
RG	100	25	0.40	0.50	0.408 (0.015)	0.573 (0.034)	0.014 (0.009)	0 - 0.046	0.001 (0)	-451.63 (15.77)	0.073 (0.034)	0.003 - 0.247
RG	100	25	0.40	0.60	0.405 (0.013)	0.568 (0.035)	0.011 (0.009)	0 - 0.047	0.001 (0)	-453.8 (14.44)	0.034 (0.033)	0.004 - 0.135
RG	100	25	0.40	0.70	0.402 (0.012)	0.571 (0.025)	0.011 (0.007)	0 - 0.025	0.001 (0)	-455.02 (16.54)	0.129 (0.025)	0.104 - 0.215
RG	100	25	0.40	0.80	0.398 (0.013)	0.572 (0.026)	0.01 (0.009)	0 - 0.045	0.001 (0)	-455.35 (14.74)	0.228 (0.026)	0.174 - 0.314
RG	100	25	0.40	0.90	0.394 (0.014)	0.568 (0.031)	0.011 (0.01)	0 - 0.045	0.001 (0)	-459.54 (18.2)	0.332 (0.031)	0.301 - 0.416
RG	100	25	0.50	0.10	0.503 (0.02)	0.622 (0.173)	0.017 (0.01)	0 - 0.047	0.001 (0)	-449.2 (14.16)	0.522 (0.173)	0.238 - 1.053
RG	100	25	0.50	0.20	0.5 (0.02)	0.571 (0.16)	0.018 (0.01)	0 - 0.044	0.001 (0)	-449.68 (14.56)	0.372 (0.158)	0.024 - 0.953
RG	100	25	0.50	0.30	0.5 (0.02)	0.597 (0.17)	0.018 (0.008)	0.003 - 0.042	0.001 (0)	-452.68 (14.12)	0.307 (0.151)	0.048 - 0.788
RG	100	25	0.50	0.40	0.501 (0.019)	0.566 (0.16)	0.017 (0.01)	0.001 - 0.044	0.001 (0)	-449.95 (14.79)	0.188 (0.133)	0.013 - 0.666
RG	100	25	0.50	0.50	0.501 (0.019)	0.579 (0.185)	0.017 (0.009)	0.001 - 0.043	0.001 (0)	-450.63 (12.46)	0.128 (0.155)	0.001 - 0.585

Continued on next page

Table S1 – *Continued from previous page*

Structure	T	N	p	p_e/p_w	$\overline{\hat{p}}$ (SD)	$\overline{\hat{p}_e}/\overline{\hat{p}_w}$ (SD)	$\overline{\lvert\Delta\rvert}_p$ (SD)	Range $\lvert\Delta\rvert_p$	$\overline{SE}\lvert\Delta\rvert_p$ (SD)	$\overline{BIC}$ (SD)	$\overline{\lvert\Delta\rvert}_{p_e/p_w}$ (SD)	Range $\lvert\Delta\rvert_{p_e/p_w}$
RG	100	25	0.50	0.60	0.503 (0.02)	0.591 (0.15)	0.016 (0.011)	0.002 - 0.052	0.001 (0)	-453.46 (14.54)	0.108 (0.104)	0.003 - 0.44
RG	100	25	0.50	0.70	0.502 (0.019)	0.602 (0.146)	0.017 (0.009)	0.001 - 0.046	0.001 (0)	-448.82 (13.45)	0.158 (0.077)	0.003 - 0.366
RG	100	25	0.50	0.80	0.501 (0.021)	0.564 (0.152)	0.018 (0.011)	0.001 - 0.06	0.001 (0)	-453.87 (13.99)	0.259 (0.107)	0 - 0.676
RG	100	25	0.50	0.90	0.501 (0.02)	0.577 (0.178)	0.017 (0.01)	0.003 - 0.06	0.001 (0)	-451.97 (12.94)	0.342 (0.136)	0.001 - 0.742
RG	100	25	0.60	0.10	0.557 (0.03)	0.752 (0.164)	0.043 (0.03)	0.01 - 0.184	0.001 (0)	-448.84 (14.2)	0.652 (0.164)	0.081 - 1.028
RG	100	25	0.60	0.20	0.574 (0.015)	0.634 (0.058)	0.027 (0.015)	0 - 0.056	0.001 (0)	-450.83 (12.65)	0.434 (0.058)	0.372 - 0.867
RG	100	25	0.60	0.30	0.575 (0.016)	0.623 (0.043)	0.026 (0.014)	0 - 0.059	0.001 (0)	-451.66 (15.59)	0.323 (0.043)	0.241 - 0.501
RG	100	25	0.60	0.40	0.588 (0.015)	0.562 (0.026)	0.015 (0.011)	0 - 0.051	0.002 (0)	-449.18 (13.52)	0.162 (0.026)	0.088 - 0.23
RG	100	25	0.60	0.50	0.604 (0.015)	0.543 (0.022)	0.012 (0.01)	0 - 0.04	0.002 (0)	-449.86 (13.8)	0.043 (0.022)	0 - 0.096
RG	100	25	0.60	0.60	0.605 (0.015)	0.527 (0.02)	0.012 (0.01)	0 - 0.043	0.002 (0)	-450.86 (13.27)	0.073 (0.02)	0.036 - 0.116
RG	100	25	0.60	0.70	0.613 (0.013)	0.528 (0.021)	0.015 (0.011)	0.001 - 0.05	0.001 (0)	-450.23 (13.63)	0.172 (0.021)	0.114 - 0.213
RG	100	25	0.60	0.80	0.616 (0.014)	0.53 (0.021)	0.018 (0.011)	0.001 - 0.05	0.001 (0)	-451.97 (14.1)	0.27 (0.021)	0.208 - 0.315
RG	100	25	0.60	0.90	0.624 (0.012)	0.512 (0.022)	0.024 (0.011)	0.001 - 0.051	0.001 (0)	-452.22 (14.61)	0.388 (0.022)	0.343 - 0.445
RG	100	25	0.70	0.10	0.579 (0.079)	0.812 (0.402)	0.121 (0.079)	0.051 - 0.311	0.001 (0)	-451.76 (18.38)	0.718 (0.392)	0.031 - 2.4
RG	100	25	0.70	0.20	0.649 (0.015)	0.637 (0.021)	0.051 (0.015)	0.018 - 0.094	0.001 (0)	-449.76 (13.67)	0.437 (0.021)	0.379 - 0.513
RG	100	25	0.70	0.30	0.653 (0.016)	0.623 (0.018)	0.047 (0.016)	0.013 - 0.091	0.001 (0)	-446.68 (13.19)	0.323 (0.018)	0.278 - 0.376
RG	100	25	0.70	0.40	0.677 (0.015)	0.559 (0.023)	0.023 (0.015)	0 - 0.062	0.001 (0)	-446.13 (12.92)	0.159 (0.023)	0.126 - 0.198
RG	100	25	0.70	0.50	0.699 (0.012)	0.533 (0.029)	0.01 (0.007)	0 - 0.028	0.001 (0)	-443.41 (14.22)	0.043 (0.01)	0.011 - 0.088
RG	100	25	0.70	0.60	0.702 (0.014)	0.499 (0.044)	0.011 (0.008)	0 - 0.034	0.001 (0)	-449.89 (16.97)	0.101 (0.044)	0.003 - 0.152

Continued on next page

Table S1 – *Continued from previous page*

Structure	T	N	p	p_e/p_w	$\overline{\hat{p}}$ (SD)	$\overline{\hat{p_e}}/\overline{\hat{p_w}}$ (SD)	$\overline{\lvert\Delta\rvert}_p$ (SD)	Range $\lvert\Delta\rvert_p$	$\overline{SE}\lvert\Delta\rvert_p$ (SD)	$\overline{BIC}$ (SD)	$\overline{\lvert\Delta\rvert}_{p_e/p_w}$ (SD)	Range $\lvert\Delta\rvert_{p_e/p_w}$
RG	100	25	0.70	0.70	0.708 (0.014)	0.5 (0.037)	0.014 (0.009)	0 - 0.034	0.001 (0)	-450.65 (20.61)	0.2 (0.037)	0.143 - 0.252
RG	100	25	0.70	0.80	0.712 (0.013)	0.501 (0.035)	0.015 (0.01)	0 - 0.042	0.001 (0)	-451.54 (22.02)	0.299 (0.035)	0.245 - 0.353
RG	100	25	0.70	0.90	0.716 (0.01)	0.496 (0.024)	0.016 (0.009)	0.001 - 0.042	0.001 (0)	-445.78 (19.94)	0.404 (0.024)	0.347 - 0.45
RG	100	25	0.80	0.10	0.594 (0.121)	0.967 (0.904)	0.206 (0.121)	0.083 - 0.431	0.001 (0)	-473.44 (36.78)	0.875 (0.895)	0.001 - 5.571
RG	100	25	0.80	0.20	0.741 (0.019)	0.646 (0.019)	0.059 (0.019)	0.01 - 0.125	0.001 (0)	-440.02 (17.03)	0.446 (0.019)	0.414 - 0.481
RG	100	25	0.80	0.30	0.751 (0.015)	0.626 (0.016)	0.049 (0.015)	0.005 - 0.081	0.001 (0)	-439.69 (15.05)	0.326 (0.016)	0.266 - 0.377
RG	100	25	0.80	0.40	0.786 (0.01)	0.57 (0.023)	0.014 (0.009)	0 - 0.041	0.001 (0)	-420.16 (16.11)	0.17 (0.023)	0.094 - 0.216
RG	100	25	0.80	0.50	0.8 (0.007)	0.534 (0.025)	0.006 (0.004)	0 - 0.019	0.001 (0)	-409.75 (13.97)	0.035 (0.023)	0.001 - 0.096
RG	100	25	0.80	0.60	0.803 (0.008)	0.515 (0.024)	0.007 (0.005)	0 - 0.026	0.001 (0)	-408.18 (14.45)	0.085 (0.024)	0.009 - 0.142
RG	100	25	0.80	0.70	0.804 (0.008)	0.5 (0.029)	0.007 (0.005)	0 - 0.033	0.001 (0)	-408.4 (12.5)	0.2 (0.029)	0.149 - 0.295
RG	100	25	0.80	0.80	0.804 (0.007)	0.5 (0.03)	0.006 (0.004)	0 - 0.021	0.001 (0)	-408.89 (15.14)	0.3 (0.03)	0.21 - 0.353
RG	100	25	0.80	0.90	0.805 (0.008)	0.486 (0.026)	0.008 (0.005)	0.001 - 0.023	0.001 (0)	-408.75 (16.49)	0.414 (0.026)	0.354 - 0.488
RG	100	25	0.90	0.10	0.688 (0.115)	0.982 (1.12)	0.212 (0.115)	0.126 - 0.548	0.001 (0)	-478.97 (57.03)	0.884 (1.118)	0.002 - 10.023
RG	100	25	0.90	0.20	0.868 (0.01)	0.683 (0.04)	0.032 (0.01)	0 - 0.066	0.001 (0)	-386.04 (16.87)	0.483 (0.04)	0.379 - 0.564
RG	100	25	0.90	0.30	0.883 (0.008)	0.657 (0.049)	0.017 (0.008)	0.003 - 0.043	0.001 (0)	-371.89 (16.22)	0.357 (0.049)	0.101 - 0.455
RG	100	25	0.90	0.40	0.898 (0.006)	0.448 (0.089)	0.005 (0.004)	0 - 0.022	0.001 (0)	-349.37 (13.07)	0.063 (0.078)	0.001 - 0.259
RG	100	25	0.90	0.50	0.9 (0.006)	0.419 (0.056)	0.005 (0.004)	0 - 0.017	0.001 (0)	-344.49 (11.76)	0.094 (0.031)	0.002 - 0.204
RG	100	25	0.90	0.60	0.901 (0.006)	0.401 (0.051)	0.005 (0.004)	0 - 0.016	0.001 (0)	-341.83 (12.64)	0.199 (0.051)	0.019 - 0.315
RG	100	25	0.90	0.70	0.9 (0.006)	0.412 (0.058)	0.005 (0.004)	0 - 0.018	0.001 (0)	-343.83 (12.78)	0.288 (0.058)	0.103 - 0.416
RG	100	25	0.90	0.80	0.901 (0.006)	0.416 (0.056)	0.005 (0.003)	0 - 0.018	0.001 (0)	-343.05 (13.74)	0.384 (0.056)	0.167 - 0.495

Continued on next page

Table S1 – *Continued from previous page*

Structure	T	N	p	p_e/p_w	$\overline{\hat{p}}$ (SD)	$\overline{\hat{p}_e}/\overline{\hat{p}_w}$ (SD)	$\overline{\lvert\Delta\rvert}_p$ (SD)	Range $\lvert\Delta\rvert_p$	$\overline{SE}\lvert\Delta\rvert_p$ (SD)	$\overline{BIC}$ (SD)	$\overline{\lvert\Delta\rvert}_{p_e/p_w}$ (SD)	Range $\lvert\Delta\rvert_{p_e/p_w}$
RG	100	25	0.90	0.90	0.9 (0.007)	0.418 (0.073)	0.005 (0.004)	0 - 0.018	0.001 (0)	-345.68 (12.91)	0.482 (0.073)	0.222 - 0.617
RG	100	49	0.10	0.10	0.106 (0.007)	0.736 (0.116)	0.007 (0.005)	0 - 0.02	0 (0)	-448.28 (37)	0.636 (0.116)	0.421 - 0.775
RG	100	49	0.10	0.20	0.108 (0.011)	0.755 (0.094)	0.01 (0.009)	0 - 0.034	0 (0)	-427.45 (24.41)	0.555 (0.094)	0.392 - 0.675
RG	100	49	0.10	0.30	0.1 (0.004)	0.737 (0.12)	0.003 (0.002)	0 - 0.009	0 (0)	-414.49 (13.35)	0.437 (0.12)	0.118 - 0.575
RG	100	49	0.10	0.40	0.099 (0.004)	0.705 (0.127)	0.004 (0.003)	0 - 0.014	0 (0)	-413.13 (14.27)	0.305 (0.127)	0.015 - 0.47
RG	100	49	0.10	0.50	0.1 (0.005)	0.727 (0.133)	0.004 (0.003)	0 - 0.013	0 (0)	-414.31 (13.88)	0.231 (0.126)	0.002 - 0.362
RG	100	49	0.10	0.60	0.1 (0.004)	0.702 (0.149)	0.003 (0.003)	0 - 0.01	0 (0)	-416.27 (14.54)	0.153 (0.097)	0.003 - 0.275
RG	100	49	0.10	0.70	0.1 (0.004)	0.721 (0.138)	0.004 (0.002)	0 - 0.01	0 (0)	-415.99 (12.88)	0.123 (0.065)	0.003 - 0.31
RG	100	49	0.10	0.80	0.1 (0.004)	0.722 (0.152)	0.004 (0.002)	0 - 0.013	0 (0)	-411.82 (14.81)	0.129 (0.112)	0.022 - 0.452
RG	100	49	0.10	0.90	0.1 (0.004)	0.729 (0.141)	0.004 (0.002)	0 - 0.011	0 (0)	-414.36 (11.67)	0.171 (0.141)	0.025 - 0.544
RG	100	49	0.20	0.10	0.213 (0.007)	0.701 (0.099)	0.013 (0.007)	0.001 - 0.028	0.001 (0)	-487.94 (14.39)	0.601 (0.099)	0.345 - 0.8
RG	100	49	0.20	0.20	0.207 (0.007)	0.699 (0.107)	0.008 (0.006)	0 - 0.024	0.001 (0)	-485.07 (17.18)	0.499 (0.107)	0.296 - 0.628
RG	100	49	0.20	0.30	0.205 (0.007)	0.698 (0.124)	0.006 (0.005)	0 - 0.023	0.001 (0)	-477.15 (16.38)	0.398 (0.124)	0.04 - 0.528
RG	100	49	0.20	0.40	0.201 (0.006)	0.679 (0.137)	0.005 (0.004)	0 - 0.019	0.001 (0)	-472.6 (13.05)	0.28 (0.135)	0.043 - 0.437
RG	100	49	0.20	0.50	0.2 (0.005)	0.668 (0.159)	0.004 (0.003)	0 - 0.012	0.001 (0)	-472.88 (14.08)	0.19 (0.132)	0 - 0.332
RG	100	49	0.20	0.60	0.199 (0.006)	0.664 (0.164)	0.005 (0.004)	0 - 0.016	0.001 (0)	-472.16 (12.84)	0.159 (0.075)	0.001 - 0.352
RG	100	49	0.20	0.70	0.199 (0.006)	0.639 (0.188)	0.005 (0.003)	0 - 0.015	0.001 (0)	-473.31 (12.36)	0.173 (0.094)	0.069 - 0.459
RG	100	49	0.20	0.80	0.2 (0.006)	0.663 (0.18)	0.004 (0.004)	0 - 0.018	0.001 (0)	-473.8 (13.89)	0.152 (0.168)	0 - 0.604

Continued on next page

Table S1 – *Continued from previous page*

Structure	T	N	p	p_e/p_w	$\overline{\hat{p}}$ (SD)	$\overline{\hat{p_e}}/\overline{\hat{p_w}}$ (SD)	$\overline{\lvert\Delta\rvert}_p$ (SD)	Range $\lvert\Delta\rvert_p$	$\overline{SE}\lvert\Delta\rvert_p$ (SD)	$\overline{BIC}$ (SD)	$\overline{\lvert\Delta\rvert}_{p_e/p_w}$ (SD)	Range $\lvert\Delta\rvert_{p_e/p_w}$
RG	100	49	0.20	0.90	0.201 (0.005)	0.62 (0.212)	0.004 (0.003)	0 - 0.016	0.001 (0)	-475.96 (13.55)	0.28 (0.212)	0.063 - 0.683
RG	100	49	0.30	0.10	0.326 (0.009)	0.632 (0.073)	0.026 (0.009)	0.006 - 0.046	0.001 (0)	-513.09 (16.49)	0.532 (0.073)	0.399 - 0.7
RG	100	49	0.30	0.20	0.319 (0.01)	0.596 (0.076)	0.019 (0.01)	0.001 - 0.048	0.001 (0)	-509.18 (14.89)	0.396 (0.076)	0.272 - 0.527
RG	100	49	0.30	0.30	0.31 (0.009)	0.566 (0.07)	0.011 (0.008)	0 - 0.031	0.001 (0)	-506.15 (15.5)	0.266 (0.07)	0.173 - 0.421
RG	100	49	0.30	0.40	0.305 (0.008)	0.563 (0.075)	0.007 (0.005)	0 - 0.027	0.001 (0)	-504 (13.86)	0.163 (0.075)	0.074 - 0.327
RG	100	49	0.30	0.50	0.302 (0.007)	0.55 (0.064)	0.006 (0.005)	0 - 0.028	0.001 (0)	-501.74 (12.08)	0.052 (0.063)	0.001 - 0.232
RG	100	49	0.30	0.60	0.299 (0.008)	0.575 (0.08)	0.006 (0.005)	0 - 0.025	0.001 (0)	-504.45 (16.68)	0.08 (0.025)	0.012 - 0.138
RG	100	49	0.30	0.70	0.299 (0.007)	0.583 (0.09)	0.006 (0.004)	0 - 0.021	0.001 (0)	-504.05 (12.59)	0.122 (0.082)	0.001 - 0.264
RG	100	49	0.30	0.80	0.298 (0.008)	0.56 (0.114)	0.006 (0.005)	0 - 0.022	0.001 (0)	-503.05 (15.66)	0.243 (0.107)	0.009 - 0.507
RG	100	49	0.30	0.90	0.296 (0.008)	0.552 (0.119)	0.007 (0.005)	0 - 0.025	0.001 (0)	-508.8 (30.62)	0.348 (0.119)	0.032 - 0.692
RG	100	49	0.40	0.10	0.423 (0.008)	0.602 (0.055)	0.023 (0.008)	0.002 - 0.049	0.001 (0)	-516.54 (13.98)	0.502 (0.055)	0.429 - 0.823
RG	100	49	0.40	0.20	0.422 (0.009)	0.581 (0.036)	0.022 (0.009)	0.002 - 0.048	0.001 (0)	-516.95 (14.19)	0.381 (0.036)	0.322 - 0.694
RG	100	49	0.40	0.30	0.418 (0.009)	0.555 (0.029)	0.019 (0.009)	0.001 - 0.052	0.001 (0)	-522.15 (15.14)	0.255 (0.029)	0.196 - 0.328
RG	100	49	0.40	0.40	0.415 (0.008)	0.551 (0.03)	0.015 (0.007)	0 - 0.036	0.001 (0)	-519.16 (13.48)	0.151 (0.03)	0.096 - 0.191
RG	100	49	0.40	0.50	0.408 (0.01)	0.549 (0.031)	0.011 (0.007)	0 - 0.036	0.001 (0)	-519.93 (14.77)	0.049 (0.03)	0.001 - 0.091
RG	100	49	0.40	0.60	0.404 (0.01)	0.549 (0.033)	0.01 (0.006)	0 - 0.024	0.001 (0)	-522.89 (13.81)	0.051 (0.033)	0.009 - 0.106
RG	100	49	0.40	0.70	0.4 (0.011)	0.547 (0.031)	0.01 (0.005)	0 - 0.021	0.001 (0)	-522.72 (16.77)	0.153 (0.031)	0.108 - 0.209
RG	100	49	0.40	0.80	0.398 (0.012)	0.546 (0.031)	0.009 (0.007)	0 - 0.03	0.001 (0)	-523.13 (17.47)	0.254 (0.031)	0.209 - 0.306
RG	100	49	0.40	0.90	0.394 (0.009)	0.54 (0.034)	0.009 (0.007)	0 - 0.025	0.001 (0)	-532.56 (18.72)	0.36 (0.034)	0.31 - 0.424
RG	100	49	0.50	0.10	0.5 (0.014)	0.556 (0.172)	0.013 (0.006)	0 - 0.026	0.001 (0)	-518.71 (15.55)	0.456 (0.172)	0.085 - 1.616
RG	100	49	0.50	0.20	0.499 (0.014)	0.57 (0.198)	0.012 (0.007)	0.001 - 0.033	0.001 (0)	-516.13 (13.1)	0.37 (0.198)	0.134 - 1.885
RG	100	49	0.50	0.30	0.5 (0.014)	0.571 (0.185)	0.012 (0.007)	0 - 0.032	0.001 (0)	-517.83 (13.45)	0.275 (0.178)	0.053 - 1.276

Continued on next page

Table S1 – *Continued from previous page*

Structure	T	N	p	p_e/p_w	$\overline{\hat{p}}$ (SD)	$\overline{\hat{p}_e}/\overline{\hat{p}_w}$ (SD)	$\overline{\lvert\Delta\rvert}_p$ (SD)	Range $\lvert\Delta\rvert_p$	$\overline{SE}\lvert\Delta\rvert_p$ (SD)	$\overline{BIC}$ (SD)	$\overline{\lvert\Delta\rvert}_{p_e/p_w}$ (SD)	Range $\lvert\Delta\rvert_{p_e/p_w}$
RG	100	49	0.50	0.40	0.502 (0.016)	0.566 (0.163)	0.014 (0.008)	0.002 - 0.047	0.001 (0)	-518.27 (10.75)	0.173 (0.156)	0.01 - 0.962
RG	100	49	0.50	0.50	0.499 (0.015)	0.614 (0.365)	0.014 (0.007)	0.001 - 0.035	0.001 (0)	-515.62 (12.68)	0.141 (0.356)	0 - 3.411
RG	100	49	0.50	0.60	0.5 (0.015)	0.532 (0.114)	0.013 (0.007)	0.001 - 0.03	0.001 (0)	-516.28 (13.39)	0.111 (0.071)	0.008 - 0.318
RG	100	49	0.50	0.70	0.5 (0.015)	0.581 (0.174)	0.014 (0.006)	0.002 - 0.03	0.001 (0)	-518.24 (15.67)	0.192 (0.087)	0.001 - 0.6
RG	100	49	0.50	0.80	0.5 (0.015)	0.552 (0.151)	0.013 (0.008)	0 - 0.049	0.001 (0)	-517.71 (12.95)	0.268 (0.111)	0.029 - 0.681
RG	100	49	0.50	0.90	0.501 (0.014)	0.544 (0.127)	0.012 (0.007)	0 - 0.038	0.001 (0)	-518.81 (14.23)	0.358 (0.12)	0.009 - 0.751
RG	100	49	0.60	0.10	0.555 (0.023)	0.625 (0.119)	0.045 (0.023)	0.02 - 0.158	0.001 (0)	-518.17 (13.9)	0.528 (0.107)	0.011 - 0.853
RG	100	49	0.60	0.20	0.567 (0.012)	0.578 (0.02)	0.033 (0.012)	0.006 - 0.07	0.001 (0)	-517.67 (13.42)	0.378 (0.02)	0.321 - 0.449
RG	100	49	0.60	0.30	0.58 (0.011)	0.544 (0.014)	0.02 (0.011)	0 - 0.049	0.001 (0)	-517.84 (13.01)	0.244 (0.014)	0.198 - 0.287
RG	100	49	0.60	0.40	0.589 (0.012)	0.528 (0.011)	0.012 (0.01)	0 - 0.058	0.001 (0)	-515.63 (12.55)	0.128 (0.011)	0.101 - 0.159
RG	100	49	0.60	0.50	0.6 (0.01)	0.521 (0.011)	0.007 (0.007)	0 - 0.037	0.001 (0)	-514.87 (13.79)	0.021 (0.011)	0.001 - 0.049
RG	100	49	0.60	0.60	0.606 (0.009)	0.516 (0.011)	0.009 (0.006)	0 - 0.032	0.001 (0)	-515.29 (13.66)	0.084 (0.011)	0.057 - 0.107
RG	100	49	0.60	0.70	0.61 (0.01)	0.511 (0.012)	0.012 (0.008)	0.001 - 0.036	0.001 (0)	-517.94 (13.23)	0.189 (0.012)	0.158 - 0.222
RG	100	49	0.60	0.80	0.615 (0.008)	0.509 (0.011)	0.015 (0.007)	0.001 - 0.033	0.001 (0)	-515.92 (14.39)	0.291 (0.011)	0.257 - 0.308
RG	100	49	0.60	0.90	0.618 (0.008)	0.504 (0.011)	0.018 (0.008)	0 - 0.035	0.001 (0)	-522.22 (15.37)	0.396 (0.011)	0.359 - 0.419
RG	100	49	0.70	0.10	0.593 (0.062)	0.731 (1.096)	0.107 (0.062)	0.061 - 0.277	0.001 (0)	-529.68 (16.38)	0.633 (1.095)	0.008 - 11.208
RG	100	49	0.70	0.20	0.64 (0.012)	0.586 (0.011)	0.06 (0.012)	0.035 - 0.092	0.001 (0)	-519.75 (15.38)	0.386 (0.011)	0.359 - 0.41
RG	100	49	0.70	0.30	0.68 (0.01)	0.543 (0.023)	0.02 (0.01)	0.001 - 0.048	0.001 (0)	-512.2 (15.66)	0.243 (0.023)	0.16 - 0.27

Continued on next page

Table S1 – *Continued from previous page*

Structure	T	N	p	p_e/p_w	$\overline{\hat{p}}$ (SD)	$\overline{\hat{p_e}}/\overline{\hat{p_w}}$ (SD)	$\overline{\|\Delta\|}_p$ (SD)	Range $\|\Delta\|_p$	$\overline{SE}\|\Delta\|_p$ (SD)	$\overline{BIC}$ (SD)	$\overline{\|\Delta\|}_{p_e/p_w}$ (SD)	Range $\|\Delta\|_{p_e/p_w}$
RG	100	49	0.70	0.40	0.689 (0.009)	0.511 (0.035)	0.012 (0.007)	0 - 0.027	0.001 (0)	-513.33 (18.89)	0.111 (0.035)	0.049 - 0.168
RG	100	49	0.70	0.50	0.7 (0.008)	0.504 (0.027)	0.007 (0.005)	0 - 0.02	0.001 (0)	-510.68 (21.09)	0.023 (0.013)	0 - 0.05
RG	100	49	0.70	0.60	0.702 (0.007)	0.5 (0.019)	0.006 (0.004)	0 - 0.017	0.001 (0)	-504.62 (18.33)	0.1 (0.019)	0.056 - 0.148
RG	100	49	0.70	0.70	0.704 (0.007)	0.505 (0.019)	0.007 (0.005)	0 - 0.018	0.001 (0)	-503.7 (16.74)	0.195 (0.019)	0.16 - 0.245
RG	100	49	0.70	0.80	0.705 (0.006)	0.5 (0.018)	0.006 (0.005)	0 - 0.02	0.001 (0)	-504.62 (17.45)	0.3 (0.018)	0.262 - 0.349
RG	100	49	0.70	0.90	0.706 (0.006)	0.497 (0.021)	0.007 (0.005)	0 - 0.022	0.001 (0)	-505.74 (15.05)	0.403 (0.021)	0.356 - 0.457
RG	100	49	0.80	0.10	0.685 (0.015)	0.677 (0.01)	0.115 (0.015)	0.078 - 0.161	0.001 (0)	-550.76 (19.91)	0.577 (0.01)	0.56 - 0.595
RG	100	49	0.80	0.20	0.769 (0.013)	0.627 (0.023)	0.031 (0.013)	0.008 - 0.083	0.001 (0)	-504.45 (23.12)	0.427 (0.023)	0.363 - 0.473
RG	100	49	0.80	0.30	0.796 (0.007)	0.529 (0.072)	0.007 (0.004)	0 - 0.017	0.001 (0)	-478.96 (15.31)	0.229 (0.072)	0.089 - 0.343
RG	100	49	0.80	0.40	0.799 (0.006)	0.504 (0.068)	0.005 (0.003)	0 - 0.014	0.001 (0)	-474.12 (16.91)	0.105 (0.065)	0 - 0.223
RG	100	49	0.80	0.50	0.8 (0.005)	0.486 (0.06)	0.004 (0.003)	0 - 0.017	0.001 (0)	-473.85 (14.29)	0.049 (0.037)	0.001 - 0.138
RG	100	49	0.80	0.60	0.801 (0.006)	0.471 (0.06)	0.005 (0.004)	0 - 0.022	0.001 (0)	-471.19 (13.52)	0.129 (0.059)	0.001 - 0.228
RG	100	49	0.80	0.70	0.802 (0.005)	0.468 (0.057)	0.005 (0.003)	0 - 0.015	0.001 (0)	-472.83 (14.51)	0.232 (0.057)	0.115 - 0.35
RG	100	49	0.80	0.80	0.801 (0.006)	0.461 (0.055)	0.005 (0.003)	0 - 0.019	0.001 (0)	-470.06 (12.02)	0.339 (0.055)	0.172 - 0.469
RG	100	49	0.80	0.90	0.801 (0.005)	0.469 (0.058)	0.005 (0.003)	0 - 0.015	0.001 (0)	-473.9 (14.4)	0.431 (0.058)	0.298 - 0.523
RG	100	49	0.90	0.10	0.845 (0.01)	0.754 (0.051)	0.055 (0.01)	0.04 - 0.101	0.001 (0)	-540.26 (36.41)	0.654 (0.051)	0.503 - 0.699
RG	100	49	0.90	0.20	0.894 (0.005)	0.518 (0.154)	0.007 (0.004)	0 - 0.018	0 (0)	-424.08 (16.61)	0.318 (0.154)	0.066 - 0.554
RG	100	49	0.90	0.30	0.9 (0.005)	0.432 (0.125)	0.004 (0.003)	0 - 0.015	0 (0)	-414.45 (12.42)	0.14 (0.117)	0.001 - 0.435
RG	100	49	0.90	0.40	0.9 (0.004)	0.429 (0.128)	0.003 (0.003)	0 - 0.011	0 (0)	-415.08 (13.94)	0.105 (0.078)	0.004 - 0.331
RG	100	49	0.90	0.50	0.9 (0.005)	0.41 (0.111)	0.004 (0.003)	0 - 0.011	0 (0)	-413.53 (14.29)	0.125 (0.068)	0.003 - 0.252

Continued on next page

Table S1 – *Continued from previous page*

Structure	T	N	p	p_e/p_w	$\overline{\hat{p}}$ (SD)	$\overline{\hat{p}_e}/\overline{\hat{p}_w}$ (SD)	$\overline{\lvert\Delta\rvert}_p$ (SD)	Range $\lvert\Delta\rvert_p$	$\overline{SE}\lvert\Delta\rvert_p$ (SD)	$\overline{BIC}$ (SD)	$\overline{\lvert\Delta\rvert}_{p_e/p_w}$ (SD)	Range $\lvert\Delta\rvert_{p_e/p_w}$
RG	100	49	0.90	0.60	0.9 (0.004)	0.424 (0.122)	0.003 (0.003)	0 - 0.01	0 (0)	-409.75 (10.97)	0.19 (0.099)	0.001 - 0.358
RG	100	49	0.90	0.70	0.901 (0.004)	0.424 (0.111)	0.003 (0.002)	0 - 0.01	0 (0)	-414.55 (12.5)	0.277 (0.111)	0.004 - 0.452
RG	100	49	0.90	0.80	0.9 (0.004)	0.417 (0.113)	0.004 (0.002)	0 - 0.009	0 (0)	-415.57 (14.6)	0.383 (0.113)	0.098 - 0.557
RG	100	49	0.90	0.90	0.9 (0.005)	0.403 (0.119)	0.004 (0.003)	0 - 0.012	0 (0)	-413.15 (13.53)	0.497 (0.119)	0.175 - 0.652
RG	100	100	0.10	0.10	0.108 (0.009)	0.76 (0.063)	0.009 (0.008)	0 - 0.024	0 (0)	-496 (17.7)	0.66 (0.063)	0.447 - 0.757
RG	100	100	0.10	0.20	0.1 (0.003)	0.757 (0.083)	0.003 (0.002)	0 - 0.008	0 (0)	-487.72 (15.11)	0.557 (0.083)	0.366 - 0.659
RG	100	100	0.10	0.30	0.1 (0.003)	0.74 (0.087)	0.003 (0.002)	0 - 0.007	0 (0)	-489.52 (13.87)	0.44 (0.087)	0.236 - 0.578
RG	100	100	0.10	0.40	0.1 (0.003)	0.748 (0.084)	0.003 (0.002)	0 - 0.009	0 (0)	-487.72 (14.43)	0.348 (0.084)	0.15 - 0.461
RG	100	100	0.10	0.50	0.1 (0.003)	0.746 (0.082)	0.003 (0.002)	0 - 0.009	0 (0)	-489.4 (13.18)	0.246 (0.082)	0.085 - 0.353
RG	100	100	0.10	0.60	0.1 (0.003)	0.753 (0.082)	0.002 (0.002)	0 - 0.007	0 (0)	-486.36 (14.04)	0.153 (0.082)	0.004 - 0.303
RG	100	100	0.10	0.70	0.1 (0.003)	0.75 (0.086)	0.002 (0.002)	0 - 0.009	0 (0)	-486.13 (12.94)	0.086 (0.049)	0.008 - 0.165
RG	100	100	0.10	0.80	0.1 (0.003)	0.75 (0.083)	0.002 (0.002)	0 - 0.007	0 (0)	-487.79 (13.69)	0.075 (0.061)	0.001 - 0.268
RG	100	100	0.10	0.90	0.1 (0.003)	0.749 (0.084)	0.003 (0.002)	0 - 0.009	0 (0)	-487.25 (13.18)	0.151 (0.084)	0.026 - 0.333
RG	100	100	0.20	0.10	0.207 (0.007)	0.694 (0.114)	0.008 (0.005)	0 - 0.024	0 (0)	-569.91 (28.99)	0.594 (0.114)	0.414 - 0.729
RG	100	100	0.20	0.20	0.201 (0.005)	0.694 (0.118)	0.004 (0.003)	0 - 0.014	0 (0)	-544.45 (14.06)	0.494 (0.118)	0.222 - 0.629
RG	100	100	0.20	0.30	0.2 (0.004)	0.665 (0.134)	0.003 (0.002)	0 - 0.011	0 (0)	-546.08 (14.12)	0.365 (0.134)	0.115 - 0.529
RG	100	100	0.20	0.40	0.199 (0.004)	0.694 (0.133)	0.003 (0.002)	0 - 0.009	0 (0)	-544.9 (13.71)	0.294 (0.133)	0.054 - 0.434
RG	100	100	0.20	0.50	0.199 (0.004)	0.683 (0.131)	0.004 (0.002)	0 - 0.01	0 (0)	-546.65 (15.02)	0.191 (0.12)	0.005 - 0.332
RG	100	100	0.20	0.60	0.2 (0.004)	0.661 (0.133)	0.003 (0.002)	0 - 0.012	0 (0)	-545.95 (13.35)	0.116 (0.089)	0.002 - 0.229

Continued on next page

Table S1 – *Continued from previous page*

Structure	T	N	p	p_e/p_w	$\overline{\hat{p}}$ (SD)	$\overline{\hat{p}_e}/\overline{\hat{p}_w}$ (SD)	$\overline{\lvert\Delta\rvert}_p$ (SD)	Range $\lvert\Delta\rvert_p$	$\overline{SE}\lvert\Delta\rvert_p$ (SD)	$\overline{BIC}$ (SD)	$\overline{\lvert\Delta\rvert}_{p_e/p_w}$ (SD)	Range $\lvert\Delta\rvert_{p_e/p_w}$
RG	100	100	0.20	0.70	0.2 (0.005)	0.685 (0.133)	0.004 (0.003)	0 - 0.013	0 (0)	-546.01 (13.66)	0.124 (0.05)	0.051 - 0.297
RG	100	100	0.20	0.80	0.2 (0.004)	0.679 (0.138)	0.003 (0.003)	0 - 0.011	0 (0)	-544.2 (13.41)	0.13 (0.13)	0 - 0.726
RG	100	100	0.20	0.90	0.199 (0.004)	0.669 (0.144)	0.003 (0.002)	0 - 0.009	0 (0)	-545.46 (13.68)	0.231 (0.144)	0.062 - 0.535
RG	100	100	0.30	0.10	0.318 (0.006)	0.605 (0.078)	0.018 (0.006)	0.007 - 0.035	0 (0)	-585.7 (17.56)	0.505 (0.078)	0.352 - 0.611
RG	100	100	0.30	0.20	0.31 (0.007)	0.611 (0.081)	0.011 (0.006)	0 - 0.028	0 (0)	-581.88 (18.46)	0.411 (0.081)	0.247 - 0.523
RG	100	100	0.30	0.30	0.305 (0.006)	0.619 (0.083)	0.006 (0.005)	0 - 0.021	0 (0)	-574.54 (15.33)	0.319 (0.083)	0.183 - 0.425
RG	100	100	0.30	0.40	0.301 (0.005)	0.603 (0.094)	0.004 (0.003)	0 - 0.012	0 (0)	-573.77 (15.82)	0.203 (0.094)	0.064 - 0.327
RG	100	100	0.30	0.50	0.3 (0.005)	0.603 (0.111)	0.004 (0.003)	0 - 0.012	0 (0)	-570.58 (13.99)	0.12 (0.092)	0.003 - 0.23
RG	100	100	0.30	0.60	0.299 (0.004)	0.601 (0.117)	0.004 (0.002)	0 - 0.011	0 (0)	-571.63 (13.63)	0.111 (0.037)	0.044 - 0.312
RG	100	100	0.30	0.70	0.299 (0.005)	0.588 (0.125)	0.004 (0.003)	0 - 0.013	0 (0)	-570.84 (12.9)	0.128 (0.109)	0 - 0.382
RG	100	100	0.30	0.80	0.299 (0.005)	0.586 (0.137)	0.004 (0.003)	0 - 0.017	0 (0)	-570.81 (13.39)	0.215 (0.135)	0 - 0.547
RG	100	100	0.30	0.90	0.299 (0.005)	0.567 (0.115)	0.004 (0.003)	0 - 0.011	0 (0)	-571.52 (14.02)	0.333 (0.115)	0.176 - 0.586
RG	100	100	0.40	0.10	0.428 (0.007)	0.55 (0.034)	0.028 (0.007)	0.012 - 0.054	0.001 (0)	-590.93 (15.56)	0.45 (0.034)	0.41 - 0.646
RG	100	100	0.40	0.20	0.422 (0.008)	0.522 (0.011)	0.022 (0.008)	0.003 - 0.042	0.001 (0)	-588.18 (14.76)	0.322 (0.011)	0.304 - 0.37
RG	100	100	0.40	0.30	0.413 (0.007)	0.52 (0.013)	0.013 (0.006)	0 - 0.031	0.001 (0)	-589.72 (15.5)	0.22 (0.013)	0.202 - 0.27
RG	100	100	0.40	0.40	0.407 (0.007)	0.515 (0.012)	0.008 (0.006)	0 - 0.029	0.001 (0)	-586.21 (14.37)	0.115 (0.012)	0.098 - 0.17
RG	100	100	0.40	0.50	0.4 (0.007)	0.513 (0.013)	0.005 (0.004)	0 - 0.016	0.001 (0)	-584.77 (15.52)	0.014 (0.012)	0.001 - 0.069
RG	100	100	0.40	0.60	0.397 (0.006)	0.518 (0.026)	0.005 (0.004)	0 - 0.014	0.001 (0)	-584.26 (14.23)	0.084 (0.015)	0.03 - 0.14

Continued on next page

Table S1 – *Continued from previous page*

Structure	T	N	p	p_e/p_w	$\overline{\hat{p}}$ (SD)	$\overline{\hat{p}_e}/\overline{\hat{p}_w}$ (SD)	$\overline{\lvert\Delta\rvert}_p$ (SD)	Range $\lvert\Delta\rvert_p$	$\overline{SE}\lvert\Delta\rvert_p$ (SD)	$\overline{BIC}$ (SD)	$\overline{\lvert\Delta\rvert}_{p_e/p_w}$ (SD)	Range $\lvert\Delta\rvert_{p_e/p_w}$
RG	100	100	0.40	0.70	0.395 (0.006)	0.515 (0.017)	0.007 (0.004)	0 - 0.023	0.001 (0)	-584.99 (12.84)	0.185 (0.017)	0.131 - 0.217
RG	100	100	0.40	0.80	0.395 (0.006)	0.512 (0.022)	0.006 (0.004)	0 - 0.018	0.001 (0)	-589 (13.57)	0.288 (0.022)	0.144 - 0.325
RG	100	100	0.40	0.90	0.393 (0.005)	0.511 (0.031)	0.007 (0.004)	0 - 0.019	0.001 (0)	-591.64 (16.25)	0.389 (0.031)	0.203 - 0.514
RG	100	100	0.50	0.10	0.501 (0.009)	0.56 (0.222)	0.008 (0.005)	0 - 0.021	0.001 (0)	-588.5 (13.4)	0.46 (0.222)	0.035 - 2.326
RG	100	100	0.50	0.20	0.5 (0.01)	0.546 (0.149)	0.009 (0.005)	0.002 - 0.029	0.001 (0)	-586.27 (11.11)	0.349 (0.141)	0.07 - 1.304
RG	100	100	0.50	0.30	0.5 (0.01)	0.575 (0.192)	0.008 (0.004)	0 - 0.022	0.001 (0)	-589.01 (13.32)	0.28 (0.184)	0.065 - 1.683
RG	100	100	0.50	0.40	0.5 (0.009)	0.573 (0.258)	0.008 (0.004)	0.001 - 0.021	0.001 (0)	-591.09 (14.04)	0.176 (0.256)	0.026 - 2.495
RG	100	100	0.50	0.50	0.501 (0.011)	0.574 (0.223)	0.01 (0.005)	0.002 - 0.03	0.001 (0)	-588.46 (14.75)	0.092 (0.216)	0.001 - 2.043
RG	100	100	0.50	0.60	0.502 (0.01)	0.635 (0.606)	0.009 (0.004)	0 - 0.018	0.001 (0)	-588.66 (12.91)	0.152 (0.587)	0.001 - 5.923
RG	100	100	0.50	0.70	0.5 (0.01)	0.536 (0.115)	0.009 (0.005)	0.001 - 0.02	0.001 (0)	-586.49 (14.08)	0.185 (0.076)	0.033 - 0.654
RG	100	100	0.50	0.80	0.5 (0.011)	0.55 (0.106)	0.01 (0.005)	0.001 - 0.022	0.001 (0)	-590.43 (13.42)	0.255 (0.094)	0.008 - 0.389
RG	100	100	0.50	0.90	0.501 (0.01)	0.569 (0.119)	0.008 (0.005)	0.001 - 0.022	0.001 (0)	-589.84 (14.18)	0.331 (0.119)	0.001 - 0.745
RG	100	100	0.60	0.10	0.553 (0.008)	0.576 (0.013)	0.047 (0.008)	0.027 - 0.069	0.001 (0)	-593.96 (14.91)	0.476 (0.013)	0.445 - 0.539
RG	100	100	0.60	0.20	0.568 (0.008)	0.532 (0.008)	0.032 (0.008)	0.014 - 0.055	0.001 (0)	-589.43 (14.21)	0.332 (0.008)	0.313 - 0.356
RG	100	100	0.60	0.30	0.58 (0.009)	0.522 (0.008)	0.02 (0.008)	0.001 - 0.037	0.001 (0)	-585.16 (15.67)	0.222 (0.008)	0.205 - 0.238
RG	100	100	0.60	0.40	0.592 (0.006)	0.513 (0.007)	0.008 (0.006)	0 - 0.027	0.001 (0)	-586.68 (16.65)	0.113 (0.007)	0.101 - 0.129
RG	100	100	0.60	0.50	0.599 (0.006)	0.509 (0.006)	0.004 (0.004)	0 - 0.018	0.001 (0)	-585.32 (14.14)	0.009 (0.006)	0.001 - 0.027
RG	100	100	0.60	0.60	0.605 (0.005)	0.507 (0.006)	0.006 (0.004)	0 - 0.018	0.001 (0)	-587.22 (15.74)	0.093 (0.006)	0.071 - 0.107

Continued on next page

Table S1 – *Continued from previous page*

Structure	T	N	p	p_e/p_w	$\overline{\hat{p}}$ (SD)	$\overline{\hat{p}_e}/\overline{\hat{p}_w}$ (SD)	$\overline{\lvert\Delta\rvert}_p$ (SD)	Range $\lvert\Delta\rvert_p$	$\overline{SE}\lvert\Delta\rvert_p$ (SD)	$\overline{BIC}$ (SD)	$\overline{\lvert\Delta\rvert}_{p_e/p_w}$ (SD)	Range $\lvert\Delta\rvert_{p_e/p_w}$
RG	100	100	0.60	0.70	0.606 (0.005)	0.504 (0.007)	0.007 (0.005)	0 - 0.018	0.001 (0)	-589.43 (12.99)	0.196 (0.007)	0.174 - 0.215
RG	100	100	0.60	0.80	0.609 (0.005)	0.502 (0.007)	0.009 (0.005)	0.001 - 0.021	0.001 (0)	-592.02 (13.6)	0.298 (0.007)	0.283 - 0.318
RG	100	100	0.60	0.90	0.61 (0.004)	0.5 (0.008)	0.01 (0.004)	0 - 0.021	0.001 (0)	-594.32 (16.62)	0.4 (0.008)	0.384 - 0.424
RG	100	100	0.70	0.10	0.618 (0.012)	0.584 (0.007)	0.082 (0.012)	0.053 - 0.107	0.001 (0)	-614.08 (18.21)	0.484 (0.007)	0.465 - 0.499
RG	100	100	0.70	0.20	0.681 (0.008)	0.534 (0.032)	0.019 (0.008)	0.001 - 0.039	0 (0)	-589.7 (21.26)	0.334 (0.032)	0.256 - 0.396
RG	100	100	0.70	0.30	0.696 (0.006)	0.522 (0.035)	0.006 (0.005)	0 - 0.024	0 (0)	-572.1 (14.4)	0.222 (0.035)	0.125 - 0.274
RG	100	100	0.70	0.40	0.7 (0.005)	0.507 (0.038)	0.004 (0.003)	0 - 0.014	0 (0)	-569.85 (13.33)	0.107 (0.038)	0.036 - 0.178
RG	100	100	0.70	0.50	0.701 (0.004)	0.497 (0.031)	0.004 (0.003)	0 - 0.014	0 (0)	-568.69 (15.4)	0.024 (0.019)	0.001 - 0.073
RG	100	100	0.70	0.60	0.701 (0.004)	0.497 (0.033)	0.004 (0.003)	0 - 0.011	0 (0)	-571.7 (13.21)	0.103 (0.033)	0.023 - 0.167
RG	100	100	0.70	0.70	0.701 (0.005)	0.495 (0.032)	0.004 (0.003)	0 - 0.012	0 (0)	-571.49 (13.59)	0.205 (0.032)	0.134 - 0.259
RG	100	100	0.70	0.80	0.701 (0.005)	0.496 (0.029)	0.004 (0.003)	0 - 0.012	0 (0)	-572.31 (14.26)	0.304 (0.029)	0.233 - 0.367
RG	100	100	0.70	0.90	0.701 (0.005)	0.496 (0.032)	0.004 (0.003)	0 - 0.012	0 (0)	-573.29 (14.73)	0.404 (0.032)	0.313 - 0.476
RG	100	100	0.80	0.10	0.774 (0.007)	0.638 (0.074)	0.026 (0.007)	0.01 - 0.047	0 (0)	-582.9 (19.64)	0.538 (0.074)	0.324 - 0.6
RG	100	100	0.80	0.20	0.799 (0.004)	0.468 (0.091)	0.003 (0.003)	0 - 0.012	0 (0)	-544.87 (14.34)	0.268 (0.091)	0.143 - 0.486
RG	100	100	0.80	0.30	0.801 (0.004)	0.45 (0.075)	0.004 (0.003)	0 - 0.013	0 (0)	-545.65 (15.25)	0.15 (0.075)	0.032 - 0.357
RG	100	100	0.80	0.40	0.801 (0.004)	0.432 (0.055)	0.003 (0.002)	0 - 0.009	0 (0)	-542.93 (16.22)	0.05 (0.04)	0.001 - 0.239
RG	100	100	0.80	0.50	0.8 (0.004)	0.446 (0.07)	0.003 (0.002)	0 - 0.011	0 (0)	-547.3 (15.17)	0.077 (0.042)	0.001 - 0.165
RG	100	100	0.80	0.60	0.8 (0.004)	0.436 (0.066)	0.003 (0.002)	0 - 0.01	0 (0)	-543.31 (11.84)	0.168 (0.056)	0.003 - 0.266
RG	100	100	0.80	0.70	0.8 (0.004)	0.443 (0.071)	0.003 (0.002)	0 - 0.01	0 (0)	-546.83 (13.79)	0.257 (0.071)	0.034 - 0.378

Continued on next page

Table S1 – *Continued from previous page*

Structure	T	N	p	p_e/p_w	$\overline{\hat{p}}$ (SD)	$\overline{\hat{p_e}}/\overline{\hat{p_w}}$ (SD)	$\overline{\lvert\Delta\rvert}_p$ (SD)	Range $\lvert\Delta\rvert_p$	$\overline{SE}\lvert\Delta\rvert_p$ (SD)	$\overline{BIC}$ (SD)	$\overline{\lvert\Delta\rvert}_{p_e/p_w}$ (SD)	Range $\lvert\Delta\rvert_{p_e/p_w}$
RG	100	100	0.80	0.80	0.8 (0.004)	0.433 (0.064)	0.003 (0.002)	0 - 0.01	0 (0)	-542.05 (13.29)	0.367 (0.064)	0.142 - 0.469
RG	100	100	0.80	0.90	0.801 (0.004)	0.449 (0.068)	0.003 (0.002)	0 - 0.009	0 (0)	-543.99 (14.12)	0.451 (0.068)	0.256 - 0.551
RG	100	100	0.90	0.10	0.893 (0.004)	0.458 (0.118)	0.007 (0.004)	0 - 0.016	0 (0)	-498.15 (14.83)	0.358 (0.118)	0.144 - 0.668
RG	100	100	0.90	0.20	0.901 (0.003)	0.365 (0.117)	0.003 (0.002)	0 - 0.007	0 (0)	-487.31 (13.72)	0.165 (0.117)	0.001 - 0.412
RG	100	100	0.90	0.30	0.9 (0.003)	0.366 (0.116)	0.003 (0.002)	0 - 0.008	0 (0)	-486.61 (12.77)	0.111 (0.074)	0 - 0.316
RG	100	100	0.90	0.40	0.9 (0.003)	0.383 (0.113)	0.002 (0.002)	0 - 0.009	0 (0)	-485.81 (14.57)	0.101 (0.052)	0.001 - 0.211
RG	100	100	0.90	0.50	0.901 (0.003)	0.354 (0.118)	0.003 (0.002)	0 - 0.01	0 (0)	-486.2 (14.86)	0.158 (0.101)	0 - 0.307
RG	100	100	0.90	0.60	0.901 (0.003)	0.369 (0.112)	0.002 (0.002)	0 - 0.008	0 (0)	-486.81 (13.74)	0.231 (0.112)	0.018 - 0.394
RG	100	100	0.90	0.70	0.9 (0.003)	0.369 (0.115)	0.003 (0.002)	0 - 0.01	0 (0)	-484.05 (13.69)	0.331 (0.115)	0.093 - 0.481
RG	100	100	0.90	0.80	0.9 (0.003)	0.372 (0.117)	0.002 (0.002)	0 - 0.008	0 (0)	-486.33 (14.04)	0.428 (0.117)	0.2 - 0.588
RG	100	100	0.90	0.90	0.9 (0.003)	0.407 (0.123)	0.003 (0.002)	0 - 0.009	0 (0)	-488.18 (13.72)	0.493 (0.123)	0.291 - 0.696
RG	200	16	0.10	0.10	0.122 (0.008)	0.788 (0.017)	0.022 (0.008)	0.003 - 0.04	0 (0)	-657.97 (30.33)	0.688 (0.017)	0.649 - 0.776
RG	200	16	0.10	0.20	0.134 (0.008)	0.77 (0.047)	0.034 (0.008)	0.015 - 0.054	0 (0)	-675.11 (28.14)	0.57 (0.047)	0.251 - 0.596
RG	200	16	0.10	0.30	0.104 (0.007)	0.793 (0.073)	0.006 (0.005)	0 - 0.024	0 (0)	-630.47 (43.18)	0.493 (0.073)	0.167 - 0.636
RG	200	16	0.10	0.40	0.103 (0.006)	0.793 (0.078)	0.005 (0.004)	0 - 0.017	0 (0)	-642.64 (56.85)	0.393 (0.078)	0.146 - 0.609
RG	200	16	0.10	0.50	0.098 (0.013)	0.772 (0.086)	0.007 (0.011)	0 - 0.075	0 (0)	-667.94 (73.58)	0.272 (0.086)	0.033 - 0.474
RG	200	16	0.10	0.60	0.092 (0.017)	0.777 (0.085)	0.011 (0.015)	0 - 0.075	0 (0)	-665.46 (76.15)	0.178 (0.082)	0.074 - 0.374
RG	200	16	0.10	0.70	0.089 (0.021)	0.784 (0.095)	0.013 (0.019)	0 - 0.075	0 (0)	-676.14 (86.31)	0.092 (0.087)	0 - 0.255

Continued on next page

Table S1 – *Continued from previous page*

Structure	T	N	p	p_e/p_w	$\overline{\hat{p}}$ (SD)	$\overline{\hat{p_e}}/\overline{\hat{p_w}}$ (SD)	$\overline{\lvert\Delta\rvert}_p$ (SD)	Range $\lvert\Delta\rvert_p$	$\overline{SE}\lvert\Delta\rvert_p$ (SD)	$\overline{BIC}$ (SD)	$\overline{\lvert\Delta\rvert}_{p_e/p_w}$ (SD)	Range $\lvert\Delta\rvert_{p_e/p_w}$
RG	200	16	0.10	0.80	0.086 (0.02)	0.76 (0.097)	0.016 (0.019)	0 - 0.075	0 (0)	-693.28 (87.62)	0.094 (0.046)	0.003 - 0.313
RG	200	16	0.10	0.90	0.087 (0.022)	0.78 (0.096)	0.015 (0.02)	0 - 0.075	0 (0)	-689.46 (92.21)	0.131 (0.08)	0 - 0.215
RG	200	16	0.20	0.10	0.234 (0.009)	0.698 (0.033)	0.034 (0.009)	0.008 - 0.054	0.001 (0)	-757.41 (18.74)	0.598 (0.033)	0.423 - 0.658
RG	200	16	0.20	0.20	0.246 (0.01)	0.634 (0.069)	0.046 (0.01)	0.02 - 0.081	0.001 (0)	-766.46 (19.19)	0.434 (0.069)	0.259 - 0.552
RG	200	16	0.20	0.30	0.21 (0.009)	0.708 (0.05)	0.011 (0.008)	0 - 0.038	0.001 (0)	-744.18 (22.47)	0.408 (0.05)	0.23 - 0.523
RG	200	16	0.20	0.40	0.206 (0.009)	0.689 (0.049)	0.008 (0.006)	0 - 0.029	0.001 (0)	-743.72 (22.45)	0.289 (0.049)	0.134 - 0.363
RG	200	16	0.20	0.50	0.205 (0.009)	0.688 (0.06)	0.008 (0.006)	0 - 0.024	0.001 (0)	-753.18 (34.3)	0.188 (0.06)	0 - 0.291
RG	200	16	0.20	0.60	0.203 (0.009)	0.687 (0.083)	0.008 (0.005)	0 - 0.023	0.001 (0)	-754 (41.52)	0.098 (0.07)	0 - 0.513
RG	200	16	0.20	0.70	0.201 (0.01)	0.678 (0.065)	0.008 (0.006)	0 - 0.028	0.001 (0)	-769.25 (53.45)	0.053 (0.043)	0 - 0.225
RG	200	16	0.20	0.80	0.199 (0.01)	0.69 (0.066)	0.008 (0.006)	0 - 0.026	0.001 (0)	-775.72 (62.85)	0.112 (0.063)	0.004 - 0.286
RG	200	16	0.20	0.90	0.196 (0.01)	0.68 (0.066)	0.008 (0.007)	0 - 0.038	0.001 (0)	-788.05 (78.74)	0.22 (0.066)	0.08 - 0.378
RG	200	16	0.30	0.10	0.335 (0.008)	0.658 (0.012)	0.035 (0.008)	0.018 - 0.056	0.001 (0)	-804.75 (19.58)	0.558 (0.012)	0.51 - 0.61
RG	200	16	0.30	0.20	0.347 (0.009)	0.638 (0.04)	0.047 (0.009)	0.029 - 0.076	0.001 (0)	-812.66 (18.96)	0.438 (0.04)	0.37 - 0.741
RG	200	16	0.30	0.30	0.318 (0.01)	0.656 (0.021)	0.019 (0.008)	0 - 0.035	0.001 (0)	-808.94 (20.03)	0.356 (0.021)	0.21 - 0.372
RG	200	16	0.30	0.40	0.316 (0.011)	0.654 (0.021)	0.017 (0.009)	0.001 - 0.043	0.001 (0)	-807.32 (21.5)	0.254 (0.021)	0.122 - 0.272
RG	200	16	0.30	0.50	0.314 (0.011)	0.652 (0.022)	0.016 (0.009)	0 - 0.04	0.001 (0)	-810.5 (24.13)	0.152 (0.022)	0.08 - 0.171
RG	200	16	0.30	0.60	0.312 (0.009)	0.652 (0.02)	0.013 (0.007)	0 - 0.029	0.001 (0)	-816.62 (23.26)	0.052 (0.019)	0.003 - 0.071
RG	200	16	0.30	0.70	0.313 (0.01)	0.644 (0.026)	0.014 (0.008)	0 - 0.036	0.001 (0)	-829.46 (23.17)	0.056 (0.026)	0.029 - 0.154
RG	200	16	0.30	0.80	0.305 (0.01)	0.644 (0.024)	0.009 (0.007)	0 - 0.028	0.001 (0)	-824.33 (31.49)	0.156 (0.024)	0.128 - 0.223
RG	200	16	0.30	0.90	0.303 (0.01)	0.643 (0.023)	0.008 (0.005)	0 - 0.023	0.001 (0)	-834.69 (33.34)	0.257 (0.023)	0.23 - 0.317
RG	200	16	0.40	0.10	0.419 (0.007)	0.615 (0.063)	0.019 (0.007)	0.002 - 0.036	0.001 (0)	-826.4 (18.81)	0.515 (0.063)	0.472 - 0.818

Continued on next page

Table S1 – *Continued from previous page*

Structure	T	N	p	p_e/p_w	$\overline{\hat{p}}$ (SD)	$\overline{\hat{p_e}}/\overline{\hat{p_w}}$ (SD)	$\overline{\lvert\Delta\rvert}_p$ (SD)	Range $\lvert\Delta\rvert_p$	$\overline{SE}\lvert\Delta\rvert_p$ (SD)	$\overline{BIC}$ (SD)	$\overline{\lvert\Delta\rvert}_{p_e/p_w}$ (SD)	Range $\lvert\Delta\rvert_{p_e/p_w}$
RG	200	16	0.40	0.20	0.424 (0.011)	0.603 (0.053)	0.024 (0.011)	0.001 - 0.059	0.001 (0)	-824.97 (19.37)	0.403 (0.053)	0.372 - 0.795
RG	200	16	0.40	0.30	0.412 (0.011)	0.601 (0.041)	0.013 (0.009)	0 - 0.041	0.001 (0)	-824.99 (17.91)	0.301 (0.041)	0.217 - 0.499
RG	200	16	0.40	0.40	0.408 (0.011)	0.599 (0.029)	0.011 (0.008)	0 - 0.029	0.001 (0)	-825.38 (20.35)	0.199 (0.029)	0.137 - 0.354
RG	200	16	0.40	0.50	0.405 (0.012)	0.581 (0.031)	0.01 (0.008)	0 - 0.032	0.001 (0)	-823.63 (18.74)	0.082 (0.028)	0.002 - 0.149
RG	200	16	0.40	0.60	0.404 (0.012)	0.587 (0.025)	0.01 (0.008)	0 - 0.034	0.001 (0)	-823.23 (22.3)	0.019 (0.02)	0 - 0.084
RG	200	16	0.40	0.70	0.4 (0.012)	0.569 (0.035)	0.01 (0.006)	0 - 0.024	0.001 (0)	-826.01 (20.89)	0.131 (0.035)	0.078 - 0.253
RG	200	16	0.40	0.80	0.397 (0.01)	0.58 (0.03)	0.009 (0.006)	0 - 0.027	0.001 (0)	-827.54 (22.32)	0.22 (0.03)	0.181 - 0.298
RG	200	16	0.40	0.90	0.394 (0.009)	0.584 (0.03)	0.01 (0.006)	0 - 0.039	0.001 (0)	-836.49 (21.41)	0.316 (0.03)	0.278 - 0.421
RG	200	16	0.50	0.10	0.498 (0.015)	0.602 (0.214)	0.013 (0.007)	0 - 0.031	0.001 (0)	-829.83 (18.37)	0.502 (0.214)	0.196 - 2.183
RG	200	16	0.50	0.20	0.499 (0.017)	0.538 (0.141)	0.015 (0.008)	0 - 0.037	0.001 (0)	-826.91 (17.45)	0.339 (0.14)	0.021 - 0.832
RG	200	16	0.50	0.30	0.501 (0.017)	0.579 (0.224)	0.015 (0.008)	0 - 0.038	0.001 (0)	-826.81 (19.14)	0.281 (0.222)	0.036 - 2.265
RG	200	16	0.50	0.40	0.501 (0.017)	0.577 (0.163)	0.015 (0.008)	0 - 0.044	0.001 (0)	-828.69 (19.41)	0.184 (0.154)	0.01 - 1.008
RG	200	16	0.50	0.50	0.5 (0.015)	0.582 (0.145)	0.013 (0.007)	0.001 - 0.034	0.001 (0)	-828.32 (21.35)	0.11 (0.125)	0.003 - 0.542
RG	200	16	0.50	0.60	0.499 (0.018)	0.572 (0.16)	0.016 (0.01)	0.002 - 0.058	0.001 (0)	-823.38 (16.08)	0.105 (0.124)	0.001 - 0.633
RG	200	16	0.50	0.70	0.499 (0.015)	0.563 (0.121)	0.013 (0.007)	0 - 0.035	0.001 (0)	-826.9 (21.01)	0.166 (0.076)	0.018 - 0.393
RG	200	16	0.50	0.80	0.5 (0.015)	0.575 (0.243)	0.013 (0.007)	0 - 0.033	0.001 (0)	-824.12 (20.43)	0.277 (0.18)	0.059 - 1.784
RG	200	16	0.50	0.90	0.499 (0.015)	0.58 (0.163)	0.013 (0.006)	0.003 - 0.034	0.001 (0)	-829.34 (18.56)	0.332 (0.137)	0.016 - 0.852
RG	200	16	0.60	0.10	0.556 (0.028)	0.841 (1.222)	0.044 (0.028)	0.01 - 0.179	0.001 (0)	-835.12 (27.33)	0.741 (1.222)	0.023 - 9.397
RG	200	16	0.60	0.20	0.563 (0.016)	0.634 (0.076)	0.037 (0.015)	0.003 - 0.078	0.001 (0)	-829.21 (21.13)	0.434 (0.076)	0.3 - 0.622

Continued on next page

Table S1 – *Continued from previous page*

Structure	T	N	p	p_e/p_w	$\overline{\hat{p}}$ (SD)	$\overline{\hat{p}_e}/\overline{\hat{p}_w}$ (SD)	$\overline{\lvert\Delta\rvert}_p$ (SD)	Range $\lvert\Delta\rvert_p$	$\overline{SE}\lvert\Delta\rvert_p$ (SD)	$\overline{BIC}$ (SD)	$\overline{\lvert\Delta\rvert}_{p_e/p_w}$ (SD)	Range $\lvert\Delta\rvert_{p_e/p_w}$
RG	200	16	0.60	0.30	0.582 (0.012)	0.606 (0.056)	0.018 (0.011)	0 - 0.048	0.001 (0)	-829.39 (18.65)	0.306 (0.056)	0.21 - 0.449
RG	200	16	0.60	0.40	0.591 (0.014)	0.58 (0.041)	0.013 (0.01)	0 - 0.042	0.001 (0)	-829.08 (18.51)	0.18 (0.041)	0.111 - 0.269
RG	200	16	0.60	0.50	0.597 (0.013)	0.557 (0.034)	0.01 (0.008)	0 - 0.036	0.001 (0)	-825.1 (20.23)	0.057 (0.034)	0.011 - 0.12
RG	200	16	0.60	0.60	0.606 (0.014)	0.547 (0.029)	0.013 (0.009)	0 - 0.035	0.001 (0)	-823.21 (19.28)	0.056 (0.023)	0.001 - 0.087
RG	200	16	0.60	0.70	0.61 (0.015)	0.536 (0.017)	0.015 (0.01)	0 - 0.042	0.001 (0)	-822.3 (18.54)	0.164 (0.017)	0.086 - 0.203
RG	200	16	0.60	0.80	0.622 (0.012)	0.535 (0.017)	0.023 (0.011)	0 - 0.048	0.001 (0)	-825.93 (22.04)	0.265 (0.017)	0.176 - 0.317
RG	200	16	0.60	0.90	0.624 (0.012)	0.533 (0.011)	0.025 (0.01)	0.004 - 0.055	0.001 (0)	-826.71 (17.81)	0.367 (0.011)	0.325 - 0.411
RG	200	16	0.70	0.10	0.538 (0.086)	0.743 (0.693)	0.162 (0.086)	0.051 - 0.315	0.001 (0)	-872.03 (70.64)	0.659 (0.677)	0.043 - 5.311
RG	200	16	0.70	0.20	0.61 (0.027)	0.667 (0.095)	0.09 (0.027)	0.04 - 0.164	0.001 (0)	-844.54 (43.19)	0.467 (0.095)	0.3 - 0.807
RG	200	16	0.70	0.30	0.651 (0.016)	0.61 (0.059)	0.049 (0.016)	0.014 - 0.11	0.001 (0)	-843.12 (37.7)	0.31 (0.059)	0.2 - 0.39
RG	200	16	0.70	0.40	0.682 (0.013)	0.592 (0.03)	0.019 (0.012)	0.001 - 0.063	0.001 (0)	-818.96 (21.76)	0.192 (0.03)	0.11 - 0.286
RG	200	16	0.70	0.50	0.694 (0.013)	0.56 (0.03)	0.012 (0.008)	0 - 0.048	0.001 (0)	-811.43 (20.49)	0.06 (0.03)	0.013 - 0.126
RG	200	16	0.70	0.60	0.704 (0.01)	0.553 (0.023)	0.008 (0.006)	0 - 0.026	0.001 (0)	-816.55 (19.38)	0.049 (0.019)	0.005 - 0.09
RG	200	16	0.70	0.70	0.714 (0.01)	0.535 (0.015)	0.015 (0.008)	0.001 - 0.039	0.001 (0)	-808.33 (18.37)	0.165 (0.015)	0.142 - 0.192
RG	200	16	0.70	0.80	0.723 (0.008)	0.524 (0.018)	0.023 (0.008)	0.002 - 0.041	0.001 (0)	-808.97 (20.07)	0.276 (0.018)	0.237 - 0.34
RG	200	16	0.70	0.90	0.724 (0.007)	0.522 (0.018)	0.024 (0.007)	0.003 - 0.044	0.001 (0)	-815.01 (19.13)	0.378 (0.018)	0.338 - 0.433
RG	200	16	0.80	0.10	0.435 (0.041)	0.643 (1.142)	0.365 (0.041)	0.168 - 0.449	0.001 (0)	-1042.39 (62.44)	0.543 (1.142)	0.374 - 11.848
RG	200	16	0.80	0.20	0.662 (0.046)	0.699 (0.088)	0.138 (0.046)	0.083 - 0.263	0.001 (0)	-854.69 (81.23)	0.499 (0.088)	0.31 - 0.67
RG	200	16	0.80	0.30	0.746 (0.023)	0.626 (0.056)	0.054 (0.023)	0.018 - 0.109	0.001 (0)	-830.53 (57.55)	0.326 (0.056)	0.21 - 0.395

Continued on next page

Table S1 – *Continued from previous page*

Structure	T	N	p	p_e/p_w	$\overline{\hat{p}}$ (SD)	$\overline{\hat{p}_e}/\overline{\hat{p}_w}$ (SD)	$\overline{\lvert\Delta\rvert}_p$ (SD)	Range $\lvert\Delta\rvert_p$	$\overline{SE}\lvert\Delta\rvert_p$ (SD)	$\overline{BIC}$ (SD)	$\overline{\lvert\Delta\rvert}_{p_e/p_w}$ (SD)	Range $\lvert\Delta\rvert_{p_e/p_w}$
RG	200	16	0.80	0.40	0.78 (0.009)	0.605 (0.018)	0.02 (0.009)	0.002 - 0.04	0.001 (0)	-774.32 (18.22)	0.205 (0.018)	0.17 - 0.287
RG	200	16	0.80	0.50	0.797 (0.009)	0.554 (0.027)	0.008 (0.006)	0 - 0.025	0.001 (0)	-757.61 (17.07)	0.054 (0.027)	0.009 - 0.119
RG	200	16	0.80	0.60	0.803 (0.009)	0.542 (0.024)	0.007 (0.006)	0 - 0.023	0.001 (0)	-755.69 (21.79)	0.059 (0.023)	0.009 - 0.135
RG	200	16	0.80	0.70	0.807 (0.008)	0.514 (0.039)	0.009 (0.006)	0 - 0.023	0.001 (0)	-749.05 (18.9)	0.186 (0.039)	0.138 - 0.3
RG	200	16	0.80	0.80	0.811 (0.007)	0.471 (0.047)	0.011 (0.006)	0 - 0.026	0.001 (0)	-756.02 (22.47)	0.329 (0.047)	0.241 - 0.409
RG	200	16	0.80	0.90	0.811 (0.006)	0.461 (0.049)	0.011 (0.006)	0 - 0.026	0.001 (0)	-767.14 (21.97)	0.439 (0.049)	0.348 - 0.511
RG	200	16	0.90	0.10	0.361 (0.028)	0.463 (0.125)	0.539 (0.028)	0.463 - 0.584	0.001 (0)	-1036.16 (119.72)	0.371 (0.096)	0.025 - 0.447
RG	200	16	0.90	0.20	0.716 (0.058)	0.721 (0.112)	0.184 (0.058)	0.11 - 0.416	0.001 (0)	-867.48 (132.16)	0.521 (0.112)	0.31 - 1.125
RG	200	16	0.90	0.30	0.867 (0.012)	0.658 (0.064)	0.033 (0.012)	0.004 - 0.055	0 (0)	-717.93 (31.19)	0.358 (0.064)	0.219 - 0.455
RG	200	16	0.90	0.40	0.883 (0.007)	0.525 (0.052)	0.017 (0.007)	0.006 - 0.036	0 (0)	-661.47 (25.9)	0.125 (0.052)	0.053 - 0.274
RG	200	16	0.90	0.50	0.897 (0.005)	0.467 (0.039)	0.005 (0.004)	0 - 0.014	0 (0)	-618.9 (18.75)	0.044 (0.025)	0.01 - 0.13
RG	200	16	0.90	0.60	0.898 (0.006)	0.463 (0.045)	0.005 (0.004)	0 - 0.017	0 (0)	-618.86 (21.93)	0.138 (0.044)	0.004 - 0.218
RG	200	16	0.90	0.70	0.902 (0.005)	0.442 (0.045)	0.004 (0.004)	0 - 0.018	0 (0)	-604.72 (20.51)	0.258 (0.045)	0.141 - 0.33
RG	200	16	0.90	0.80	0.902 (0.005)	0.431 (0.033)	0.004 (0.003)	0 - 0.017	0 (0)	-607.47 (18.43)	0.369 (0.033)	0.271 - 0.422
RG	200	16	0.90	0.90	0.902 (0.006)	0.426 (0.037)	0.005 (0.003)	0 - 0.016	0 (0)	-605.05 (20.95)	0.474 (0.037)	0.354 - 0.524
RG	200	25	0.10	0.10	0.124 (0.006)	0.75 (0.075)	0.024 (0.006)	0.012 - 0.037	0 (0)	-744.65 (24.22)	0.65 (0.075)	0.443 - 0.724
RG	200	25	0.10	0.20	0.104 (0.005)	0.758 (0.104)	0.005 (0.004)	0 - 0.015	0 (0)	-718.42 (25.69)	0.558 (0.104)	0.337 - 0.666
RG	200	25	0.10	0.30	0.105 (0.008)	0.739 (0.115)	0.007 (0.007)	0 - 0.035	0 (0)	-728.62 (38.96)	0.439 (0.115)	0.244 - 0.565

Continued on next page

Table S1 – *Continued from previous page*

Structure	T	N	p	p_e/p_w	$\overline{\hat{p}}$ (SD)	$\overline{\hat{p}_e}/\overline{\hat{p}_w}$ (SD)	$\overline{\lvert\Delta\rvert}_p$ (SD)	Range $\lvert\Delta\rvert_p$	$\overline{SE}\lvert\Delta\rvert_p$ (SD)	$\overline{BIC}$ (SD)	$\overline{\lvert\Delta\rvert}_{p_e/p_w}$ (SD)	Range $\lvert\Delta\rvert_{p_e/p_w}$
RG	200	25	0.10	0.40	0.102 (0.005)	0.69 (0.133)	0.005 (0.003)	0 - 0.017	0 (0)	-712.63 (21.26)	0.29 (0.133)	0.1 - 0.458
RG	200	25	0.10	0.50	0.099 (0.004)	0.723 (0.149)	0.004 (0.002)	0 - 0.012	0 (0)	-701.86 (19.17)	0.23 (0.139)	0.001 - 0.366
RG	200	25	0.10	0.60	0.1 (0.004)	0.693 (0.165)	0.003 (0.003)	0 - 0.012	0 (0)	-703.41 (18.52)	0.161 (0.1)	0.002 - 0.331
RG	200	25	0.10	0.70	0.099 (0.004)	0.687 (0.166)	0.003 (0.002)	0 - 0.011	0 (0)	-699.65 (17.51)	0.157 (0.054)	0.064 - 0.36
RG	200	25	0.10	0.80	0.099 (0.005)	0.658 (0.178)	0.004 (0.003)	0 - 0.01	0 (0)	-702.68 (18.4)	0.182 (0.137)	0.025 - 0.628
RG	200	25	0.10	0.90	0.1 (0.005)	0.679 (0.174)	0.004 (0.003)	0 - 0.01	0 (0)	-700.82 (16.66)	0.221 (0.174)	0.043 - 0.673
RG	200	25	0.20	0.10	0.239 (0.01)	0.601 (0.109)	0.039 (0.01)	0.018 - 0.062	0 (0)	-851.66 (21.77)	0.501 (0.109)	0.346 - 0.827
RG	200	25	0.20	0.20	0.211 (0.007)	0.733 (0.091)	0.011 (0.007)	0 - 0.028	0 (0)	-841.28 (21.02)	0.533 (0.091)	0.298 - 0.635
RG	200	25	0.20	0.30	0.205 (0.007)	0.695 (0.109)	0.007 (0.005)	0 - 0.022	0 (0)	-838.84 (21.29)	0.395 (0.109)	0.184 - 0.529
RG	200	25	0.20	0.40	0.203 (0.007)	0.694 (0.109)	0.006 (0.005)	0 - 0.023	0 (0)	-830.35 (19.17)	0.294 (0.109)	0.131 - 0.433
RG	200	25	0.20	0.50	0.201 (0.005)	0.665 (0.115)	0.004 (0.003)	0 - 0.013	0 (0)	-833.23 (17.66)	0.166 (0.114)	0.005 - 0.329
RG	200	25	0.20	0.60	0.2 (0.007)	0.645 (0.124)	0.006 (0.004)	0 - 0.017	0 (0)	-836.56 (24.96)	0.103 (0.082)	0 - 0.237
RG	200	25	0.20	0.70	0.199 (0.007)	0.663 (0.146)	0.006 (0.004)	0 - 0.016	0 (0)	-832.06 (21.15)	0.141 (0.051)	0.049 - 0.29
RG	200	25	0.20	0.80	0.199 (0.006)	0.626 (0.183)	0.005 (0.003)	0 - 0.017	0 (0)	-829.35 (26.44)	0.182 (0.175)	0 - 0.632
RG	200	25	0.20	0.90	0.198 (0.006)	0.605 (0.208)	0.005 (0.004)	0 - 0.019	0 (0)	-827.09 (25.38)	0.295 (0.208)	0.065 - 0.782
RG	200	25	0.30	0.10	0.346 (0.01)	0.645 (0.035)	0.046 (0.01)	0.019 - 0.07	0 (0)	-903.87 (19.38)	0.545 (0.035)	0.434 - 0.605
RG	200	25	0.30	0.20	0.321 (0.01)	0.615 (0.041)	0.021 (0.01)	0.002 - 0.053	0 (0)	-900.23 (22.64)	0.415 (0.041)	0.33 - 0.519
RG	200	25	0.30	0.30	0.316 (0.01)	0.582 (0.04)	0.016 (0.009)	0.001 - 0.043	0 (0)	-899.44 (23.04)	0.282 (0.04)	0.213 - 0.372

Continued on next page

Table S1 – *Continued from previous page*

Structure	T	N	p	p_e/p_w	$\overline{\hat{p}}$ (SD)	$\overline{\hat{p}_e}/\overline{\hat{p}_w}$ (SD)	$\overline{\lvert\Delta\rvert}_p$ (SD)	Range $\lvert\Delta\rvert_p$	$\overline{SE}\lvert\Delta\rvert_p$ (SD)	$\overline{BIC}$ (SD)	$\overline{\lvert\Delta\rvert}_{p_e/p_w}$ (SD)	Range $\lvert\Delta\rvert_{p_e/p_w}$
RG	200	25	0.30	0.40	0.308 (0.008)	0.582 (0.044)	0.009 (0.007)	0 - 0.032	0 (0)	-892.07 (21.24)	0.182 (0.044)	0.102 - 0.282
RG	200	25	0.30	0.50	0.305 (0.008)	0.585 (0.044)	0.007 (0.005)	0 - 0.023	0 (0)	-891.32 (22.4)	0.085 (0.044)	0.027 - 0.218
RG	200	25	0.30	0.60	0.302 (0.009)	0.585 (0.043)	0.008 (0.005)	0 - 0.021	0 (0)	-894.25 (25.99)	0.036 (0.028)	0.002 - 0.121
RG	200	25	0.30	0.70	0.299 (0.009)	0.585 (0.037)	0.007 (0.005)	0 - 0.022	0 (0)	-898.85 (24.94)	0.115 (0.036)	0.016 - 0.198
RG	200	25	0.30	0.80	0.296 (0.008)	0.582 (0.05)	0.007 (0.006)	0 - 0.026	0 (0)	-897.9 (25.38)	0.218 (0.05)	0.088 - 0.306
RG	200	25	0.30	0.90	0.295 (0.009)	0.576 (0.052)	0.008 (0.006)	0 - 0.03	0 (0)	-900.7 (28.34)	0.324 (0.052)	0.161 - 0.409
RG	200	25	0.40	0.10	0.424 (0.009)	0.617 (0.053)	0.024 (0.009)	0.008 - 0.044	0.001 (0)	-912.89 (17.52)	0.517 (0.053)	0.468 - 0.739
RG	200	25	0.40	0.20	0.416 (0.01)	0.608 (0.044)	0.016 (0.009)	0 - 0.041	0.001 (0)	-912.29 (17.98)	0.408 (0.044)	0.37 - 0.58
RG	200	25	0.40	0.30	0.416 (0.01)	0.584 (0.017)	0.016 (0.009)	0 - 0.046	0.001 (0)	-916.04 (19.06)	0.284 (0.017)	0.228 - 0.336
RG	200	25	0.40	0.40	0.412 (0.008)	0.581 (0.01)	0.013 (0.008)	0 - 0.031	0.001 (0)	-918 (19.23)	0.181 (0.01)	0.134 - 0.196
RG	200	25	0.40	0.50	0.409 (0.009)	0.577 (0.019)	0.011 (0.007)	0 - 0.024	0.001 (0)	-917.69 (23.34)	0.077 (0.018)	0.002 - 0.096
RG	200	25	0.40	0.60	0.406 (0.01)	0.576 (0.021)	0.01 (0.007)	0 - 0.037	0.001 (0)	-919.38 (20.37)	0.024 (0.021)	0.003 - 0.099
RG	200	25	0.40	0.70	0.402 (0.011)	0.576 (0.023)	0.009 (0.007)	0 - 0.029	0.001 (0)	-922.68 (22.76)	0.124 (0.023)	0.104 - 0.216
RG	200	25	0.40	0.80	0.4 (0.009)	0.578 (0.021)	0.007 (0.006)	0 - 0.023	0.001 (0)	-930.76 (23.59)	0.222 (0.021)	0.204 - 0.307
RG	200	25	0.40	0.90	0.399 (0.01)	0.574 (0.024)	0.008 (0.007)	0 - 0.03	0.001 (0)	-931.67 (23.19)	0.326 (0.024)	0.304 - 0.413
RG	200	25	0.50	0.10	0.501 (0.014)	0.652 (0.434)	0.012 (0.007)	0.001 - 0.042	0.001 (0)	-916.55 (19.28)	0.552 (0.434)	0.303 - 4.573
RG	200	25	0.50	0.20	0.5 (0.013)	0.614 (0.169)	0.011 (0.006)	0.001 - 0.027	0.001 (0)	-913.77 (18.52)	0.417 (0.161)	0.173 - 0.958
RG	200	25	0.50	0.30	0.498 (0.013)	0.597 (0.181)	0.012 (0.007)	0.001 - 0.046	0.001 (0)	-916.85 (17.19)	0.297 (0.179)	0.021 - 0.974

Continued on next page

Table S1 – *Continued from previous page*

Structure	T	N	p	p_e/p_w	$\bar{\hat{p}}$ (SD)	$\overline{\hat{p}_e}/\overline{\hat{p}_w}$ (SD)	$\overline{\lvert\Delta\rvert}_p$ (SD)	Range $\lvert\Delta\rvert_p$	$\overline{SE}\lvert\Delta\rvert_p$ (SD)	$\overline{BIC}$ (SD)	$\overline{\lvert\Delta\rvert}_{p_e/p_w}$ (SD)	Range $\lvert\Delta\rvert_{p_e/p_w}$
RG	200	25	0.50	0.40	0.499 (0.013)	0.582 (0.157)	0.012 (0.006)	0.003 - 0.026	0.001 (0)	-919.9 (20.89)	0.19 (0.146)	0.019 - 0.646
RG	200	25	0.50	0.50	0.499 (0.015)	0.562 (0.137)	0.013 (0.007)	0 - 0.036	0.001 (0)	-915.47 (19.24)	0.095 (0.117)	0 - 0.53
RG	200	25	0.50	0.60	0.503 (0.014)	0.613 (0.219)	0.012 (0.007)	0.002 - 0.032	0.001 (0)	-919.79 (19.82)	0.139 (0.169)	0.005 - 1.291
RG	200	25	0.50	0.70	0.501 (0.015)	0.572 (0.258)	0.013 (0.008)	0 - 0.038	0.001 (0)	-917.72 (16.93)	0.201 (0.205)	0.009 - 2.017
RG	200	25	0.50	0.80	0.501 (0.014)	0.579 (0.193)	0.012 (0.007)	0.001 - 0.033	0.001 (0)	-917.22 (19.75)	0.262 (0.131)	0.003 - 0.783
RG	200	25	0.50	0.90	0.502 (0.014)	0.613 (0.175)	0.013 (0.006)	0.001 - 0.031	0.001 (0)	-915.06 (18.59)	0.311 (0.128)	0.007 - 0.618
RG	200	25	0.60	0.10	0.562 (0.009)	0.711 (0.08)	0.038 (0.009)	0.018 - 0.061	0.001 (0)	-915.5 (20.8)	0.611 (0.08)	0.517 - 1.066
RG	200	25	0.60	0.20	0.569 (0.01)	0.678 (0.069)	0.031 (0.01)	0.01 - 0.058	0.001 (0)	-913.67 (21.11)	0.478 (0.069)	0.379 - 0.937
RG	200	25	0.60	0.30	0.581 (0.012)	0.589 (0.02)	0.02 (0.011)	0.001 - 0.05	0.001 (0)	-918.66 (19.64)	0.289 (0.02)	0.232 - 0.34
RG	200	25	0.60	0.40	0.591 (0.011)	0.558 (0.021)	0.012 (0.008)	0.001 - 0.036	0.001 (0)	-916.2 (18.54)	0.158 (0.021)	0.128 - 0.201
RG	200	25	0.60	0.50	0.599 (0.011)	0.544 (0.016)	0.009 (0.006)	0 - 0.024	0.001 (0)	-915.27 (20.17)	0.044 (0.016)	0 - 0.127
RG	200	25	0.60	0.60	0.606 (0.01)	0.532 (0.018)	0.01 (0.007)	0 - 0.03	0.001 (0)	-916.72 (20.59)	0.068 (0.018)	0.045 - 0.111
RG	200	25	0.60	0.70	0.613 (0.009)	0.531 (0.019)	0.013 (0.008)	0 - 0.03	0.001 (0)	-915.1 (17.02)	0.169 (0.019)	0.142 - 0.212
RG	200	25	0.60	0.80	0.619 (0.009)	0.515 (0.02)	0.019 (0.009)	0.001 - 0.038	0.001 (0)	-920.44 (19.76)	0.285 (0.02)	0.247 - 0.311
RG	200	25	0.60	0.90	0.622 (0.01)	0.511 (0.02)	0.022 (0.01)	0 - 0.045	0.001 (0)	-921.29 (24.06)	0.389 (0.02)	0.343 - 0.414
RG	200	25	0.70	0.10	0.608 (0.038)	0.819 (0.284)	0.092 (0.038)	0.056 - 0.297	0.001 (0)	-915.45 (18.69)	0.72 (0.281)	0.032 - 1.371
RG	200	25	0.70	0.20	0.631 (0.011)	0.691 (0.022)	0.069 (0.011)	0.046 - 0.096	0.001 (0)	-916.95 (20.24)	0.491 (0.022)	0.457 - 0.526
RG	200	25	0.70	0.30	0.662 (0.013)	0.595 (0.016)	0.038 (0.013)	0.011 - 0.098	0.001 (0)	-914.21 (21.85)	0.295 (0.016)	0.268 - 0.342
RG	200	25	0.70	0.40	0.685 (0.009)	0.556 (0.021)	0.016 (0.008)	0 - 0.036	0.001 (0)	-901.29 (21)	0.156 (0.021)	0.128 - 0.196

Continued on next page

Table S1 – *Continued from previous page*

Structure	T	N	p	p_e/p_w	$\bar{\hat{p}}$ (SD)	$\overline{\hat{p}_e}/\overline{\hat{p}_w}$ (SD)	$\overline{\lvert\Delta\rvert}_p$ (SD)	Range $\lvert\Delta\rvert_p$	$\overline{SE}\lvert\Delta\rvert_p$ (SD)	$\overline{BIC}$ (SD)	$\overline{\lvert\Delta\rvert}_{p_e/p_w}$ (SD)	Range $\lvert\Delta\rvert_{p_e/p_w}$
RG	200	25	0.70	0.50	0.699 (0.008)	0.538 (0.02)	0.007 (0.005)	0 - 0.024	0.001 (0)	-895.57 (22.72)	0.042 (0.008)	0.024 - 0.061
RG	200	25	0.70	0.60	0.702 (0.01)	0.5 (0.042)	0.008 (0.006)	0 - 0.023	0.001 (0)	-915.9 (30.51)	0.1 (0.042)	0.042 - 0.155
RG	200	25	0.70	0.70	0.708 (0.01)	0.495 (0.038)	0.011 (0.008)	0 - 0.029	0.001 (0)	-917.59 (37.43)	0.205 (0.038)	0.142 - 0.253
RG	200	25	0.70	0.80	0.712 (0.008)	0.493 (0.024)	0.013 (0.007)	0 - 0.025	0.001 (0)	-913.24 (31.03)	0.307 (0.024)	0.248 - 0.353
RG	200	25	0.70	0.90	0.714 (0.007)	0.494 (0.016)	0.015 (0.007)	0 - 0.028	0.001 (0)	-908.34 (28.78)	0.406 (0.016)	0.365 - 0.453
RG	200	25	0.80	0.10	0.573 (0.123)	0.783 (0.691)	0.227 (0.123)	0.113 - 0.423	0.001 (0)	-942.1 (68.76)	0.707 (0.666)	0.004 - 4.948
RG	200	25	0.80	0.20	0.701 (0.011)	0.71 (0.013)	0.099 (0.011)	0.068 - 0.123	0.001 (0)	-922.56 (21.69)	0.51 (0.013)	0.47 - 0.564
RG	200	25	0.80	0.30	0.768 (0.008)	0.605 (0.018)	0.032 (0.008)	0.015 - 0.05	0 (0)	-880.42 (21.66)	0.305 (0.018)	0.274 - 0.337
RG	200	25	0.80	0.40	0.791 (0.007)	0.566 (0.022)	0.01 (0.006)	0 - 0.022	0 (0)	-845.74 (21.61)	0.166 (0.022)	0.125 - 0.2
RG	200	25	0.80	0.50	0.801 (0.006)	0.539 (0.021)	0.005 (0.003)	0 - 0.014	0 (0)	-831 (19.94)	0.04 (0.019)	0 - 0.096
RG	200	25	0.80	0.60	0.802 (0.006)	0.514 (0.019)	0.005 (0.003)	0 - 0.017	0 (0)	-831.16 (18.54)	0.086 (0.019)	0.044 - 0.115
RG	200	25	0.80	0.70	0.803 (0.005)	0.502 (0.021)	0.005 (0.003)	0 - 0.016	0 (0)	-832.23 (19.75)	0.198 (0.021)	0.152 - 0.256
RG	200	25	0.80	0.80	0.804 (0.005)	0.492 (0.019)	0.005 (0.004)	0 - 0.015	0 (0)	-831.07 (22.53)	0.308 (0.019)	0.254 - 0.35
RG	200	25	0.80	0.90	0.804 (0.005)	0.49 (0.02)	0.005 (0.004)	0 - 0.017	0 (0)	-832.09 (20.88)	0.41 (0.02)	0.354 - 0.474
RG	200	25	0.90	0.10	0.53 (0.166)	0.564 (0.435)	0.37 (0.166)	0.159 - 0.547	0.001 (0)	-1015.19 (151.5)	0.491 (0.404)	0.004 - 1.797
RG	200	25	0.90	0.20	0.81 (0.017)	0.748 (0.014)	0.09 (0.017)	0.042 - 0.125	0 (0)	-937.35 (29.45)	0.548 (0.014)	0.505 - 0.591
RG	200	25	0.90	0.30	0.887 (0.005)	0.601 (0.08)	0.013 (0.005)	0 - 0.027	0 (0)	-746.5 (20.67)	0.301 (0.08)	0.103 - 0.404
RG	200	25	0.90	0.40	0.897 (0.005)	0.429 (0.05)	0.004 (0.003)	0 - 0.015	0 (0)	-717.22 (21.73)	0.032 (0.048)	0 - 0.222
RG	200	25	0.90	0.50	0.901 (0.004)	0.417 (0.044)	0.003 (0.002)	0 - 0.012	0 (0)	-698.71 (18.78)	0.09 (0.027)	0.002 - 0.173
RG	200	25	0.90	0.60	0.9 (0.004)	0.415 (0.033)	0.003 (0.003)	0 - 0.013	0 (0)	-703.37 (19.68)	0.185 (0.033)	0.019 - 0.263

Continued on next page

Table S1 – *Continued from previous page*

Structure	T	N	p	p_e/p_w	$\overline{\hat{p}}$ (SD)	$\overline{\hat{p_e}}/\overline{\hat{p_w}}$ (SD)	$\overline{\lvert\Delta\rvert}_p$ (SD)	Range $\lvert\Delta\rvert_p$	$\overline{SE}\lvert\Delta\rvert_p$ (SD)	$\overline{BIC}$ (SD)	$\overline{\lvert\Delta\rvert}_{p_e/p_w}$ (SD)	Range $\lvert\Delta\rvert_{p_e/p_w}$
RG	200	25	0.90	0.70	0.901 (0.004)	0.404 (0.023)	0.004 (0.003)	0 - 0.012	0 (0)	-698.56 (18.73)	0.296 (0.023)	0.241 - 0.377
RG	200	25	0.90	0.80	0.901 (0.004)	0.41 (0.04)	0.003 (0.003)	0 - 0.013	0 (0)	-698.56 (17.38)	0.39 (0.04)	0.218 - 0.512
RG	200	25	0.90	0.90	0.901 (0.004)	0.41 (0.031)	0.004 (0.003)	0 - 0.012	0 (0)	-701.03 (17.94)	0.49 (0.031)	0.319 - 0.576
RG	200	49	0.10	0.10	0.108 (0.004)	0.738 (0.098)	0.008 (0.004)	0 - 0.018	0 (0)	-882.24 (37.85)	0.638 (0.098)	0.433 - 0.759
RG	200	49	0.10	0.20	0.103 (0.004)	0.75 (0.096)	0.004 (0.003)	0 - 0.014	0 (0)	-848.39 (20.16)	0.55 (0.096)	0.425 - 0.666
RG	200	49	0.10	0.30	0.1 (0.003)	0.71 (0.134)	0.002 (0.002)	0 - 0.009	0 (0)	-844.41 (19.47)	0.41 (0.134)	0.126 - 0.576
RG	200	49	0.10	0.40	0.1 (0.003)	0.712 (0.15)	0.002 (0.002)	0 - 0.008	0 (0)	-843.24 (17.15)	0.313 (0.148)	0.001 - 0.47
RG	200	49	0.10	0.50	0.1 (0.003)	0.712 (0.151)	0.002 (0.002)	0 - 0.008	0 (0)	-841.65 (19.72)	0.229 (0.122)	0.022 - 0.37
RG	200	49	0.10	0.60	0.099 (0.003)	0.71 (0.149)	0.002 (0.002)	0 - 0.011	0 (0)	-841.03 (18.74)	0.154 (0.102)	0.001 - 0.27
RG	200	49	0.10	0.70	0.1 (0.003)	0.692 (0.149)	0.003 (0.002)	0 - 0.009	0 (0)	-845.99 (18.87)	0.132 (0.068)	0.017 - 0.408
RG	200	49	0.10	0.80	0.1 (0.003)	0.699 (0.149)	0.003 (0.002)	0 - 0.009	0 (0)	-844.14 (20.32)	0.146 (0.104)	0.023 - 0.4
RG	200	49	0.10	0.90	0.1 (0.003)	0.718 (0.152)	0.002 (0.002)	0 - 0.011	0 (0)	-843.36 (21.12)	0.182 (0.152)	0.024 - 0.615
RG	200	49	0.20	0.10	0.22 (0.005)	0.701 (0.107)	0.02 (0.005)	0.007 - 0.033	0 (0)	-986.85 (21.89)	0.601 (0.107)	0.334 - 0.713
RG	200	49	0.20	0.20	0.206 (0.005)	0.703 (0.111)	0.006 (0.005)	0 - 0.022	0 (0)	-975.94 (24.63)	0.503 (0.111)	0.318 - 0.625
RG	200	49	0.20	0.30	0.204 (0.005)	0.68 (0.122)	0.005 (0.005)	0 - 0.02	0 (0)	-971.11 (19.37)	0.38 (0.122)	0.215 - 0.527
RG	200	49	0.20	0.40	0.201 (0.004)	0.684 (0.134)	0.003 (0.003)	0 - 0.013	0 (0)	-962.92 (21.58)	0.284 (0.134)	0.067 - 0.437
RG	200	49	0.20	0.50	0.2 (0.004)	0.669 (0.155)	0.003 (0.003)	0 - 0.015	0 (0)	-962.07 (18.07)	0.181 (0.141)	0 - 0.326
RG	200	49	0.20	0.60	0.2 (0.004)	0.63 (0.182)	0.004 (0.002)	0 - 0.012	0 (0)	-964.99 (19.91)	0.168 (0.076)	0.009 - 0.348
RG	200	49	0.20	0.70	0.2 (0.004)	0.675 (0.176)	0.003 (0.002)	0 - 0.01	0 (0)	-960.65 (17.87)	0.157 (0.082)	0.068 - 0.493
RG	200	49	0.20	0.80	0.2 (0.004)	0.604 (0.179)	0.003 (0.003)	0 - 0.011	0 (0)	-958.89 (19.95)	0.204 (0.169)	0.001 - 0.573
RG	200	49	0.20	0.90	0.2 (0.004)	0.624 (0.191)	0.003 (0.002)	0 - 0.01	0 (0)	-960.27 (18.03)	0.276 (0.191)	0.066 - 0.607

Continued on next page

Table S1 – *Continued from previous page*

Structure	T	N	p	p_e/p_w	$\overline{\hat{p}}$ (SD)	$\overline{\hat{p_e}}/\overline{\hat{p_w}}$ (SD)	$\overline{\lvert\Delta\rvert}_p$ (SD)	Range $\lvert\Delta\rvert_p$	$\overline{SE}\lvert\Delta\rvert_p$ (SD)	$\overline{BIC}$ (SD)	$\overline{\lvert\Delta\rvert}_{p_e/p_w}$ (SD)	Range $\lvert\Delta\rvert_{p_e/p_w}$
RG	200	49	0.30	0.10	0.331 (0.006)	0.599 (0.068)	0.031 (0.006)	0.012 - 0.05	0 (0)	-1035.84 (18.75)	0.499 (0.068)	0.401 - 0.656
RG	200	49	0.30	0.20	0.315 (0.007)	0.571 (0.073)	0.015 (0.007)	0 - 0.031	0 (0)	-1029.94 (24.07)	0.371 (0.073)	0.293 - 0.53
RG	200	49	0.30	0.30	0.309 (0.006)	0.553 (0.061)	0.009 (0.006)	0 - 0.023	0 (0)	-1026.66 (20.44)	0.253 (0.061)	0.192 - 0.414
RG	200	49	0.30	0.40	0.305 (0.006)	0.55 (0.058)	0.006 (0.005)	0 - 0.025	0 (0)	-1023.52 (21.35)	0.15 (0.058)	0.084 - 0.315
RG	200	49	0.30	0.50	0.302 (0.005)	0.554 (0.065)	0.004 (0.004)	0 - 0.019	0 (0)	-1022.89 (19.48)	0.054 (0.065)	0 - 0.218
RG	200	49	0.30	0.60	0.299 (0.005)	0.555 (0.07)	0.004 (0.003)	0 - 0.013	0 (0)	-1019.48 (21.48)	0.08 (0.022)	0.03 - 0.129
RG	200	49	0.30	0.70	0.299 (0.005)	0.561 (0.083)	0.004 (0.003)	0 - 0.013	0 (0)	-1018.68 (20.99)	0.145 (0.072)	0.003 - 0.243
RG	200	49	0.30	0.80	0.297 (0.005)	0.557 (0.09)	0.005 (0.004)	0 - 0.015	0 (0)	-1023.19 (37.1)	0.243 (0.09)	0.04 - 0.371
RG	200	49	0.30	0.90	0.298 (0.005)	0.552 (0.11)	0.004 (0.003)	0 - 0.02	0 (0)	-1023.84 (27.4)	0.348 (0.11)	0.061 - 0.546
RG	200	49	0.40	0.10	0.427 (0.007)	0.603 (0.056)	0.027 (0.007)	0.01 - 0.045	0 (0)	-1047.95 (18.54)	0.503 (0.056)	0.441 - 0.852
RG	200	49	0.40	0.20	0.421 (0.006)	0.564 (0.02)	0.021 (0.006)	0.008 - 0.042	0 (0)	-1051.51 (20.21)	0.364 (0.02)	0.32 - 0.404
RG	200	49	0.40	0.30	0.418 (0.006)	0.554 (0.026)	0.018 (0.006)	0.001 - 0.034	0 (0)	-1052.34 (19.82)	0.254 (0.026)	0.205 - 0.292
RG	200	49	0.40	0.40	0.416 (0.007)	0.554 (0.029)	0.016 (0.006)	0.001 - 0.031	0 (0)	-1054.59 (19.93)	0.154 (0.029)	0.1 - 0.189
RG	200	49	0.40	0.50	0.409 (0.008)	0.55 (0.031)	0.01 (0.006)	0 - 0.024	0 (0)	-1058.6 (24.47)	0.051 (0.03)	0 - 0.092
RG	200	49	0.40	0.60	0.404 (0.009)	0.553 (0.03)	0.008 (0.006)	0 - 0.023	0 (0)	-1060.03 (21.97)	0.047 (0.03)	0.009 - 0.106
RG	200	49	0.40	0.70	0.398 (0.01)	0.541 (0.032)	0.008 (0.006)	0 - 0.02	0 (0)	-1060.39 (25.6)	0.159 (0.032)	0.109 - 0.207
RG	200	49	0.40	0.80	0.396 (0.009)	0.543 (0.033)	0.008 (0.006)	0 - 0.023	0 (0)	-1077.85 (32.27)	0.257 (0.033)	0.209 - 0.309
RG	200	49	0.40	0.90	0.395 (0.007)	0.551 (0.024)	0.007 (0.006)	0 - 0.022	0 (0)	-1084.27 (34.32)	0.349 (0.024)	0.311 - 0.403
RG	200	49	0.50	0.10	0.502 (0.009)	0.57 (0.154)	0.008 (0.005)	0.001 - 0.022	0.001 (0)	-1054.37 (20.45)	0.473 (0.143)	0.076 - 0.908
RG	200	49	0.50	0.20	0.501 (0.01)	0.54 (0.133)	0.009 (0.005)	0 - 0.024	0.001 (0)	-1050.96 (19.15)	0.346 (0.117)	0.129 - 0.739
RG	200	49	0.50	0.30	0.502 (0.011)	0.543 (0.153)	0.01 (0.005)	0.001 - 0.024	0.001 (0)	-1050.67 (16.88)	0.257 (0.127)	0.09 - 0.697
RG	200	49	0.50	0.40	0.501 (0.01)	0.565 (0.151)	0.008 (0.005)	0 - 0.025	0.001 (0)	-1051.28 (20.53)	0.174 (0.141)	0.001 - 0.639

Continued on next page

Table S1 – *Continued from previous page*

Structure	T	N	p	p_e/p_w	$\overline{\hat{p}}$ (SD)	$\overline{\hat{p_e}}/\overline{\hat{p_w}}$ (SD)	$\overline{\lvert\Delta\rvert}_p$ (SD)	Range $\lvert\Delta\rvert_p$	$\overline{SE}\lvert\Delta\rvert_p$ (SD)	$\overline{BIC}$ (SD)	$\overline{\lvert\Delta\rvert}_{p_e/p_w}$ (SD)	Range $\lvert\Delta\rvert_{p_e/p_w}$
RG	200	49	0.50	0.50	0.498 (0.01)	0.568 (0.174)	0.009 (0.004)	0.002 - 0.023	0.001 (0)	-1050.36 (17.74)	0.119 (0.144)	0 - 0.558
RG	200	49	0.50	0.60	0.499 (0.01)	0.548 (0.186)	0.009 (0.005)	0 - 0.024	0.001 (0)	-1052.91 (18.48)	0.136 (0.137)	0.002 - 0.661
RG	200	49	0.50	0.70	0.499 (0.011)	0.565 (0.151)	0.009 (0.005)	0.001 - 0.028	0.001 (0)	-1049.38 (19.29)	0.181 (0.091)	0.002 - 0.617
RG	200	49	0.50	0.80	0.501 (0.011)	0.575 (0.274)	0.009 (0.005)	0 - 0.023	0.001 (0)	-1052.02 (17.25)	0.284 (0.211)	0.016 - 2.161
RG	200	49	0.50	0.90	0.5 (0.01)	0.552 (0.169)	0.009 (0.004)	0 - 0.021	0.001 (0)	-1051.41 (17.69)	0.358 (0.145)	0.022 - 0.842
RG	200	49	0.60	0.10	0.556 (0.008)	0.649 (0.059)	0.044 (0.008)	0.022 - 0.063	0 (0)	-1046.95 (16.42)	0.549 (0.059)	0.483 - 0.958
RG	200	49	0.60	0.20	0.57 (0.008)	0.562 (0.015)	0.03 (0.008)	0.015 - 0.052	0.001 (0)	-1053.12 (17.99)	0.362 (0.015)	0.332 - 0.392
RG	200	49	0.60	0.30	0.579 (0.009)	0.543 (0.012)	0.021 (0.009)	0.004 - 0.044	0.001 (0)	-1048.79 (19.4)	0.243 (0.012)	0.213 - 0.27
RG	200	49	0.60	0.40	0.589 (0.008)	0.528 (0.009)	0.011 (0.008)	0 - 0.035	0.001 (0)	-1045.14 (17.41)	0.128 (0.009)	0.113 - 0.147
RG	200	49	0.60	0.50	0.6 (0.007)	0.522 (0.01)	0.005 (0.004)	0 - 0.017	0.001 (0)	-1051.88 (19.34)	0.022 (0.009)	0.001 - 0.044
RG	200	49	0.60	0.60	0.607 (0.006)	0.518 (0.009)	0.008 (0.004)	0 - 0.02	0 (0)	-1046.34 (18.63)	0.082 (0.009)	0.07 - 0.102
RG	200	49	0.60	0.70	0.61 (0.006)	0.513 (0.009)	0.01 (0.005)	0 - 0.026	0 (0)	-1048.96 (17.41)	0.187 (0.009)	0.171 - 0.204
RG	200	49	0.60	0.80	0.615 (0.006)	0.506 (0.01)	0.015 (0.006)	0 - 0.03	0 (0)	-1056.09 (23.35)	0.294 (0.01)	0.272 - 0.324
RG	200	49	0.60	0.90	0.618 (0.006)	0.505 (0.009)	0.018 (0.006)	0.006 - 0.03	0 (0)	-1062.91 (19.13)	0.395 (0.009)	0.375 - 0.409
RG	200	49	0.70	0.10	0.596 (0.043)	0.652 (0.211)	0.104 (0.043)	0.072 - 0.272	0 (0)	-1066.1 (23.9)	0.556 (0.201)	0.008 - 1.925
RG	200	49	0.70	0.20	0.652 (0.008)	0.569 (0.009)	0.048 (0.008)	0.026 - 0.066	0 (0)	-1056.76 (19.81)	0.369 (0.009)	0.357 - 0.391
RG	200	49	0.70	0.30	0.677 (0.007)	0.543 (0.01)	0.023 (0.007)	0.006 - 0.044	0 (0)	-1043.32 (22.67)	0.243 (0.01)	0.222 - 0.266
RG	200	49	0.70	0.40	0.689 (0.007)	0.498 (0.034)	0.011 (0.007)	0 - 0.026	0 (0)	-1053.25 (35.02)	0.098 (0.034)	0.052 - 0.148
RG	200	49	0.70	0.50	0.701 (0.006)	0.512 (0.023)	0.005 (0.003)	0 - 0.016	0 (0)	-1026.39 (35.31)	0.023 (0.012)	0 - 0.049

Continued on next page

Table S1 – *Continued from previous page*

Structure	T	N	p	p_e/p_w	$\overline{\hat{p}}$ (SD)	$\overline{\hat{p}_e}/\overline{\hat{p}_w}$ (SD)	$\overline{\|\Delta\|}_p$ (SD)	Range $\|\Delta\|_p$	$\overline{SE}\|\Delta\|_p$ (SD)	$\overline{BIC}$ (SD)	$\overline{\|\Delta\|}_{p_e/p_w}$ (SD)	Range $\|\Delta\|_{p_e/p_w}$
RG	200	49	0.70	0.60	0.702 (0.005)	0.509 (0.015)	0.005 (0.003)	0 - 0.014	0 (0)	-1022.07 (26.19)	0.091 (0.015)	0.062 - 0.147
RG	200	49	0.70	0.70	0.704 (0.004)	0.503 (0.012)	0.005 (0.003)	0 - 0.013	0 (0)	-1020.75 (20.54)	0.197 (0.012)	0.17 - 0.244
RG	200	49	0.70	0.80	0.706 (0.005)	0.5 (0.01)	0.006 (0.004)	0 - 0.018	0 (0)	-1025.93 (20.36)	0.3 (0.01)	0.272 - 0.337
RG	200	49	0.70	0.90	0.706 (0.004)	0.495 (0.014)	0.006 (0.004)	0 - 0.016	0 (0)	-1022.68 (23.44)	0.405 (0.014)	0.376 - 0.444
RG	200	49	0.80	0.10	0.668 (0.009)	0.675 (0.009)	0.132 (0.009)	0.105 - 0.148	0 (0)	-1092.88 (30.81)	0.575 (0.009)	0.56 - 0.594
RG	200	49	0.80	0.20	0.783 (0.007)	0.609 (0.032)	0.017 (0.007)	0.002 - 0.035	0 (0)	-995.54 (28.8)	0.409 (0.032)	0.254 - 0.456
RG	200	49	0.80	0.30	0.795 (0.005)	0.535 (0.062)	0.006 (0.004)	0 - 0.021	0 (0)	-971.81 (23.16)	0.235 (0.062)	0.117 - 0.331
RG	200	49	0.80	0.40	0.799 (0.004)	0.504 (0.056)	0.003 (0.003)	0 - 0.015	0 (0)	-964.48 (19.47)	0.104 (0.056)	0.005 - 0.203
RG	200	49	0.80	0.50	0.801 (0.004)	0.491 (0.054)	0.003 (0.002)	0 - 0.01	0 (0)	-960.73 (21.32)	0.045 (0.03)	0.001 - 0.11
RG	200	49	0.80	0.60	0.8 (0.005)	0.482 (0.047)	0.004 (0.003)	0 - 0.013	0 (0)	-962.13 (20.34)	0.118 (0.045)	0.011 - 0.208
RG	200	49	0.80	0.70	0.8 (0.004)	0.479 (0.049)	0.003 (0.003)	0 - 0.013	0 (0)	-963.84 (21.17)	0.221 (0.049)	0.112 - 0.328
RG	200	49	0.80	0.80	0.801 (0.004)	0.475 (0.045)	0.004 (0.003)	0 - 0.011	0 (0)	-960.62 (18.95)	0.325 (0.045)	0.223 - 0.41
RG	200	49	0.80	0.90	0.801 (0.004)	0.467 (0.046)	0.003 (0.002)	0 - 0.013	0 (0)	-959.78 (20.27)	0.433 (0.046)	0.318 - 0.511
RG	200	49	0.90	0.10	0.818 (0.02)	0.741 (0.023)	0.082 (0.02)	0.048 - 0.138	0 (0)	-1153.26 (74.52)	0.641 (0.023)	0.551 - 0.68
RG	200	49	0.90	0.20	0.897 (0.004)	0.466 (0.133)	0.004 (0.003)	0 - 0.01	0 (0)	-849.04 (18.8)	0.266 (0.133)	0.073 - 0.529
RG	200	49	0.90	0.30	0.9 (0.003)	0.411 (0.099)	0.003 (0.002)	0 - 0.008	0 (0)	-841.83 (17.83)	0.115 (0.094)	0.003 - 0.372
RG	200	49	0.90	0.40	0.901 (0.003)	0.418 (0.108)	0.003 (0.002)	0 - 0.008	0 (0)	-842.15 (21.11)	0.087 (0.067)	0.004 - 0.305
RG	200	49	0.90	0.50	0.9 (0.003)	0.4 (0.099)	0.003 (0.002)	0 - 0.008	0 (0)	-840.33 (19.37)	0.126 (0.062)	0.001 - 0.214

Continued on next page

Table S1 – *Continued from previous page*

Structure	T	N	p	p_e/p_w	$\overline{\hat{p}}$ (SD)	$\overline{\hat{p}_e}/\overline{\hat{p}_w}$ (SD)	$\overline{\lvert\Delta\rvert}_p$ (SD)	Range $\lvert\Delta\rvert_p$	$\overline{SE}\lvert\Delta\rvert_p$ (SD)	$\overline{BIC}$ (SD)	$\overline{\lvert\Delta\rvert}_{p_e/p_w}$ (SD)	Range $\lvert\Delta\rvert_{p_e/p_w}$
RG	200	49	0.90	0.60	0.9 (0.003)	0.441 (0.116)	0.003 (0.002)	0 - 0.008	0 (0)	-843.79 (20.15)	0.173 (0.093)	0.001 - 0.36
RG	200	49	0.90	0.70	0.9 (0.003)	0.417 (0.094)	0.003 (0.002)	0 - 0.008	0 (0)	-843.57 (19.16)	0.283 (0.094)	0.006 - 0.425
RG	200	49	0.90	0.80	0.901 (0.003)	0.435 (0.102)	0.003 (0.002)	0 - 0.007	0 (0)	-842.45 (19.25)	0.365 (0.102)	0.116 - 0.552
RG	200	49	0.90	0.90	0.901 (0.003)	0.424 (0.101)	0.003 (0.002)	0 - 0.008	0 (0)	-841.5 (18.5)	0.476 (0.101)	0.192 - 0.656
RG	200	100	0.10	0.10	0.106 (0.006)	0.763 (0.064)	0.006 (0.005)	0 - 0.017	0 (0)	-999.39 (28.78)	0.663 (0.064)	0.498 - 0.784
RG	200	100	0.10	0.20	0.1 (0.002)	0.757 (0.084)	0.002 (0.001)	0 - 0.006	0 (0)	-984.78 (18.97)	0.557 (0.084)	0.377 - 0.66
RG	200	100	0.10	0.30	0.1 (0.002)	0.74 (0.086)	0.002 (0.001)	0 - 0.006	0 (0)	-987.95 (18.48)	0.44 (0.086)	0.289 - 0.575
RG	200	100	0.10	0.40	0.1 (0.002)	0.745 (0.087)	0.002 (0.001)	0 - 0.005	0 (0)	-986.32 (19.93)	0.345 (0.087)	0.177 - 0.481
RG	200	100	0.10	0.50	0.1 (0.002)	0.744 (0.08)	0.002 (0.001)	0 - 0.006	0 (0)	-984.59 (21.78)	0.244 (0.08)	0.101 - 0.37
RG	200	100	0.10	0.60	0.1 (0.002)	0.747 (0.081)	0.002 (0.001)	0 - 0.007	0 (0)	-987.78 (18.58)	0.148 (0.079)	0.003 - 0.275
RG	200	100	0.10	0.70	0.1 (0.002)	0.747 (0.083)	0.002 (0.001)	0 - 0.006	0 (0)	-989.5 (20.38)	0.082 (0.049)	0.003 - 0.175
RG	200	100	0.10	0.80	0.1 (0.002)	0.763 (0.078)	0.002 (0.001)	0 - 0.006	0 (0)	-984.9 (19.86)	0.064 (0.058)	0 - 0.235
RG	200	100	0.10	0.90	0.1 (0.002)	0.752 (0.083)	0.002 (0.001)	0 - 0.004	0 (0)	-988.32 (22.23)	0.148 (0.083)	0.019 - 0.325
RG	200	100	0.20	0.10	0.205 (0.004)	0.712 (0.114)	0.006 (0.004)	0 - 0.014	0 (0)	-1131.29 (37.64)	0.612 (0.114)	0.424 - 0.729
RG	200	100	0.20	0.20	0.201 (0.003)	0.694 (0.119)	0.003 (0.002)	0 - 0.009	0 (0)	-1102.81 (19.35)	0.494 (0.119)	0.218 - 0.629
RG	200	100	0.20	0.30	0.2 (0.003)	0.689 (0.122)	0.002 (0.002)	0 - 0.009	0 (0)	-1101.6 (16.77)	0.389 (0.122)	0.099 - 0.529
RG	200	100	0.20	0.40	0.199 (0.003)	0.694 (0.128)	0.002 (0.002)	0 - 0.007	0 (0)	-1104.36 (22.65)	0.294 (0.128)	0.044 - 0.429
RG	200	100	0.20	0.50	0.2 (0.003)	0.687 (0.129)	0.002 (0.002)	0 - 0.007	0 (0)	-1102.73 (20.42)	0.191 (0.122)	0.004 - 0.33

Continued on next page

Table S1 – *Continued from previous page*

Structure	T	N	p	p_e/p_w	$\bar{\hat{p}}$ (SD)	$\overline{\hat{p_e}}/\overline{\hat{p_w}}$ (SD)	$\overline{\lvert\Delta\rvert}_p$ (SD)	Range $\lvert\Delta\rvert_p$	$\overline{SE}\lvert\Delta\rvert_p$ (SD)	$\overline{BIC}$ (SD)	$\overline{\lvert\Delta\rvert}_{p_e/p_w}$ (SD)	Range $\lvert\Delta\rvert_{p_e/p_w}$
RG	200	100	0.20	0.60	0.2 (0.003)	0.682 (0.13)	0.002 (0.002)	0 - 0.009	0 (0)	-1104.94 (20.47)	0.122 (0.094)	0.001 - 0.237
RG	200	100	0.20	0.70	0.2 (0.003)	0.72 (0.126)	0.002 (0.002)	0 - 0.008	0 (0)	-1103.81 (20.62)	0.121 (0.038)	0.072 - 0.305
RG	200	100	0.20	0.80	0.2 (0.003)	0.67 (0.136)	0.002 (0.002)	0 - 0.007	0 (0)	-1102.7 (20.14)	0.143 (0.123)	0.001 - 0.367
RG	200	100	0.20	0.90	0.2 (0.003)	0.682 (0.145)	0.002 (0.002)	0 - 0.009	0 (0)	-1105.05 (19.82)	0.218 (0.145)	0.071 - 0.522
RG	200	100	0.30	0.10	0.321 (0.005)	0.615 (0.081)	0.021 (0.005)	0.011 - 0.036	0 (0)	-1185.44 (23.49)	0.515 (0.081)	0.354 - 0.61
RG	200	100	0.30	0.20	0.307 (0.005)	0.616 (0.081)	0.007 (0.005)	0 - 0.018	0 (0)	-1168.3 (25.39)	0.416 (0.081)	0.266 - 0.527
RG	200	100	0.30	0.30	0.304 (0.004)	0.605 (0.086)	0.004 (0.003)	0 - 0.017	0 (0)	-1163.42 (21.34)	0.305 (0.086)	0.173 - 0.432
RG	200	100	0.30	0.40	0.301 (0.004)	0.602 (0.095)	0.003 (0.003)	0 - 0.011	0 (0)	-1160.83 (22.16)	0.202 (0.095)	0.073 - 0.414
RG	200	100	0.30	0.50	0.3 (0.004)	0.622 (0.107)	0.003 (0.002)	0 - 0.01	0 (0)	-1161.17 (19.46)	0.13 (0.096)	0.001 - 0.338
RG	200	100	0.30	0.60	0.3 (0.003)	0.599 (0.111)	0.003 (0.002)	0 - 0.009	0 (0)	-1160.05 (20.95)	0.107 (0.028)	0.071 - 0.275
RG	200	100	0.30	0.70	0.3 (0.003)	0.6 (0.118)	0.003 (0.002)	0 - 0.009	0 (0)	-1155.85 (19.08)	0.118 (0.101)	0 - 0.347
RG	200	100	0.30	0.80	0.3 (0.003)	0.581 (0.128)	0.002 (0.002)	0 - 0.009	0 (0)	-1160.84 (20.55)	0.219 (0.128)	0.003 - 0.485
RG	200	100	0.30	0.90	0.3 (0.003)	0.573 (0.139)	0.003 (0.002)	0 - 0.009	0 (0)	-1159.16 (19.01)	0.327 (0.139)	0.036 - 0.619
RG	200	100	0.40	0.10	0.43 (0.005)	0.549 (0.035)	0.03 (0.005)	0.019 - 0.045	0 (0)	-1199.58 (24.15)	0.449 (0.035)	0.414 - 0.728
RG	200	100	0.40	0.20	0.421 (0.005)	0.52 (0.008)	0.021 (0.005)	0.009 - 0.036	0 (0)	-1193.86 (18.1)	0.32 (0.008)	0.302 - 0.338
RG	200	100	0.40	0.30	0.412 (0.005)	0.519 (0.01)	0.012 (0.005)	0 - 0.024	0 (0)	-1194.31 (21.08)	0.219 (0.01)	0.203 - 0.27
RG	200	100	0.40	0.40	0.406 (0.005)	0.514 (0.013)	0.007 (0.004)	0 - 0.02	0 (0)	-1190.16 (21.97)	0.114 (0.013)	0.096 - 0.17
RG	200	100	0.40	0.50	0.402 (0.005)	0.519 (0.018)	0.005 (0.003)	0 - 0.015	0 (0)	-1187.98 (21.66)	0.019 (0.018)	0.003 - 0.07
RG	200	100	0.40	0.60	0.398 (0.004)	0.511 (0.009)	0.004 (0.003)	0 - 0.013	0 (0)	-1185.45 (18.8)	0.089 (0.009)	0.053 - 0.106
RG	200	100	0.40	0.70	0.395 (0.004)	0.51 (0.01)	0.005 (0.003)	0 - 0.016	0 (0)	-1191.86 (21.02)	0.19 (0.01)	0.144 - 0.207
RG	200	100	0.40	0.80	0.393 (0.004)	0.509 (0.01)	0.007 (0.004)	0 - 0.016	0 (0)	-1197.1 (19.11)	0.291 (0.01)	0.246 - 0.308

Continued on next page

Table S1 – *Continued from previous page*

Structure	T	N	p	p_e/p_w	$\bar{\hat{p}}$ (SD)	$\overline{\hat{p}_e}/\overline{\hat{p}_w}$ (SD)	$\overline{\lvert\Delta\rvert}_p$ (SD)	Range $\lvert\Delta\rvert_p$	$\overline{SE}\lvert\Delta\rvert_p$ (SD)	$\overline{BIC}$ (SD)	$\overline{\lvert\Delta\rvert}_{p_e/p_w}$ (SD)	Range $\lvert\Delta\rvert_{p_e/p_w}$
RG	200	100	0.40	0.90	0.393 (0.004)	0.507 (0.011)	0.007 (0.003)	0 - 0.015	0 (0)	-1199.09 (18.12)	0.393 (0.011)	0.356 - 0.416
RG	200	100	0.50	0.10	0.5 (0.007)	0.552 (0.116)	0.006 (0.004)	0 - 0.016	0 (0)	-1193.61 (18.55)	0.452 (0.116)	0.052 - 0.95
RG	200	100	0.50	0.20	0.501 (0.008)	0.551 (0.137)	0.007 (0.004)	0 - 0.019	0 (0)	-1192.12 (20.95)	0.354 (0.128)	0.031 - 0.946
RG	200	100	0.50	0.30	0.5 (0.007)	0.567 (0.187)	0.006 (0.003)	0.001 - 0.019	0 (0)	-1192.05 (18.96)	0.27 (0.183)	0.061 - 1.651
RG	200	100	0.50	0.40	0.499 (0.007)	0.563 (0.146)	0.006 (0.003)	0.001 - 0.015	0 (0)	-1194.23 (19.81)	0.169 (0.138)	0.021 - 1.048
RG	200	100	0.50	0.50	0.5 (0.007)	0.546 (0.103)	0.006 (0.003)	0.001 - 0.021	0 (0)	-1194.45 (20.31)	0.073 (0.086)	0.002 - 0.423
RG	200	100	0.50	0.60	0.501 (0.007)	0.548 (0.121)	0.006 (0.003)	0 - 0.019	0 (0)	-1192.98 (19.56)	0.103 (0.082)	0.002 - 0.546
RG	200	100	0.50	0.70	0.501 (0.007)	0.559 (0.144)	0.006 (0.004)	0 - 0.017	0 (0)	-1192.25 (17.52)	0.179 (0.092)	0.001 - 0.577
RG	200	100	0.50	0.80	0.5 (0.007)	0.551 (0.105)	0.006 (0.003)	0 - 0.014	0 (0)	-1196.38 (16.98)	0.25 (0.102)	0 - 0.628
RG	200	100	0.50	0.90	0.5 (0.006)	0.562 (0.12)	0.006 (0.003)	0.001 - 0.015	0 (0)	-1194.21 (21.22)	0.338 (0.119)	0.006 - 0.875
RG	200	100	0.60	0.10	0.551 (0.006)	0.572 (0.011)	0.049 (0.006)	0.033 - 0.063	0 (0)	-1196.57 (19.93)	0.472 (0.011)	0.446 - 0.498
RG	200	100	0.60	0.20	0.568 (0.006)	0.529 (0.006)	0.032 (0.006)	0.016 - 0.05	0 (0)	-1197.93 (19.52)	0.329 (0.006)	0.312 - 0.344
RG	200	100	0.60	0.30	0.582 (0.005)	0.521 (0.005)	0.018 (0.005)	0.006 - 0.032	0 (0)	-1192.6 (18.21)	0.221 (0.005)	0.211 - 0.237
RG	200	100	0.60	0.40	0.591 (0.004)	0.515 (0.005)	0.009 (0.004)	0 - 0.019	0 (0)	-1189.94 (19.05)	0.115 (0.005)	0.101 - 0.129
RG	200	100	0.60	0.50	0.599 (0.004)	0.51 (0.006)	0.003 (0.003)	0 - 0.012	0 (0)	-1186.45 (20.02)	0.01 (0.006)	0.001 - 0.027
RG	200	100	0.60	0.60	0.605 (0.004)	0.508 (0.005)	0.005 (0.003)	0 - 0.013	0 (0)	-1186.22 (21.51)	0.092 (0.005)	0.081 - 0.106
RG	200	100	0.60	0.70	0.607 (0.004)	0.504 (0.005)	0.007 (0.004)	0.001 - 0.016	0 (0)	-1191.54 (20.56)	0.196 (0.005)	0.183 - 0.209
RG	200	100	0.60	0.80	0.609 (0.003)	0.502 (0.005)	0.009 (0.003)	0.001 - 0.017	0 (0)	-1197.37 (17.44)	0.298 (0.005)	0.285 - 0.308

Continued on next page

Table S1 – *Continued from previous page*

Structure	T	N	p	p_e/p_w	$\overline{\hat{p}}$ (SD)	$\overline{\hat{p}_e}/\overline{\hat{p}_w}$ (SD)	$\overline{\lvert\Delta\rvert}_p$ (SD)	Range $\lvert\Delta\rvert_p$	$\overline{SE}\lvert\Delta\rvert_p$ (SD)	$\overline{BIC}$ (SD)	$\overline{\lvert\Delta\rvert}_{p_e/p_w}$ (SD)	Range $\lvert\Delta\rvert_{p_e/p_w}$
RG	200	100	0.60	0.90	0.61 (0.003)	0.501 (0.006)	0.01 (0.003)	0.004 - 0.018	0 (0)	-1206.15 (21.59)	0.399 (0.006)	0.382 - 0.413
RG	200	100	0.70	0.10	0.612 (0.009)	0.578 (0.006)	0.088 (0.009)	0.067 - 0.107	0 (0)	-1236.08 (25.69)	0.478 (0.006)	0.457 - 0.49
RG	200	100	0.70	0.20	0.681 (0.005)	0.539 (0.026)	0.019 (0.005)	0.006 - 0.034	0 (0)	-1192.84 (24.25)	0.339 (0.026)	0.28 - 0.379
RG	200	100	0.70	0.30	0.695 (0.004)	0.535 (0.024)	0.005 (0.004)	0 - 0.015	0 (0)	-1167.86 (19.11)	0.235 (0.024)	0.164 - 0.269
RG	200	100	0.70	0.40	0.699 (0.004)	0.515 (0.028)	0.003 (0.002)	0 - 0.01	0 (0)	-1160.6 (21.52)	0.115 (0.028)	0.042 - 0.168
RG	200	100	0.70	0.50	0.7 (0.004)	0.499 (0.026)	0.003 (0.002)	0 - 0.01	0 (0)	-1158.49 (20.46)	0.02 (0.016)	0.001 - 0.071
RG	200	100	0.70	0.60	0.7 (0.003)	0.499 (0.022)	0.003 (0.002)	0 - 0.01	0 (0)	-1159.16 (16.57)	0.101 (0.022)	0.053 - 0.164
RG	200	100	0.70	0.70	0.701 (0.003)	0.498 (0.021)	0.003 (0.002)	0 - 0.008	0 (0)	-1162.99 (21.04)	0.202 (0.021)	0.153 - 0.247
RG	200	100	0.70	0.80	0.701 (0.003)	0.497 (0.024)	0.003 (0.002)	0 - 0.007	0 (0)	-1156.16 (15.73)	0.303 (0.024)	0.232 - 0.358
RG	200	100	0.70	0.90	0.701 (0.003)	0.492 (0.02)	0.003 (0.002)	0 - 0.009	0 (0)	-1158.53 (20.75)	0.408 (0.02)	0.353 - 0.458
RG	200	100	0.80	0.10	0.774 (0.005)	0.643 (0.059)	0.026 (0.005)	0.011 - 0.041	0 (0)	-1180.15 (34.3)	0.543 (0.059)	0.325 - 0.588
RG	200	100	0.80	0.20	0.799 (0.003)	0.465 (0.085)	0.002 (0.002)	0 - 0.009	0 (0)	-1097.99 (19.09)	0.265 (0.085)	0.141 - 0.457
RG	200	100	0.80	0.30	0.8 (0.003)	0.444 (0.073)	0.002 (0.002)	0 - 0.006	0 (0)	-1101.65 (22.17)	0.144 (0.073)	0.04 - 0.345
RG	200	100	0.80	0.40	0.8 (0.003)	0.447 (0.053)	0.002 (0.002)	0 - 0.009	0 (0)	-1103.44 (16.91)	0.056 (0.043)	0 - 0.232
RG	200	100	0.80	0.50	0.8 (0.003)	0.446 (0.067)	0.002 (0.002)	0 - 0.008	0 (0)	-1104.19 (19.29)	0.073 (0.046)	0.002 - 0.154
RG	200	100	0.80	0.60	0.8 (0.003)	0.448 (0.064)	0.002 (0.002)	0 - 0.006	0 (0)	-1104.14 (17.77)	0.153 (0.061)	0.004 - 0.259
RG	200	100	0.80	0.70	0.8 (0.003)	0.456 (0.069)	0.002 (0.002)	0 - 0.007	0 (0)	-1102.54 (20.58)	0.244 (0.069)	0.055 - 0.349
RG	200	100	0.80	0.80	0.8 (0.003)	0.451 (0.072)	0.002 (0.002)	0 - 0.007	0 (0)	-1102.06 (20.73)	0.349 (0.072)	0.134 - 0.467

Continued on next page

Table S1 – *Continued from previous page*

Structure	T	N	p	p_e/p_w	$\overline{\hat{p}}$ (SD)	$\overline{\hat{p}_e}/\overline{\hat{p}_w}$ (SD)	$\overline{\lvert\Delta\rvert}_p$ (SD)	Range $\lvert\Delta\rvert_p$	$\overline{SE}\lvert\Delta\rvert_p$ (SD)	$\overline{BIC}$ (SD)	$\overline{\lvert\Delta\rvert}_{p_e/p_w}$ (SD)	Range $\lvert\Delta\rvert_{p_e/p_w}$
RG	200	100	0.80	0.90	0.8 (0.003)	0.436 (0.058)	0.002 (0.002)	0 - 0.008	0 (0)	-1103.47 (17.83)	0.464 (0.058)	0.278 - 0.565
RG	200	100	0.90	0.10	0.895 (0.002)	0.431 (0.116)	0.005 (0.002)	0.001 - 0.011	0 (0)	-1008.65 (22.38)	0.331 (0.116)	0.136 - 0.617
RG	200	100	0.90	0.20	0.9 (0.002)	0.376 (0.107)	0.002 (0.001)	0 - 0.005	0 (0)	-986.16 (18.51)	0.176 (0.107)	0.014 - 0.403
RG	200	100	0.90	0.30	0.9 (0.002)	0.38 (0.113)	0.002 (0.001)	0 - 0.005	0 (0)	-989.46 (21.06)	0.117 (0.074)	0.003 - 0.404
RG	200	100	0.90	0.40	0.9 (0.002)	0.377 (0.107)	0.002 (0.001)	0 - 0.006	0 (0)	-987.88 (20.12)	0.099 (0.047)	0 - 0.186
RG	200	100	0.90	0.50	0.9 (0.002)	0.383 (0.109)	0.002 (0.001)	0 - 0.005	0 (0)	-985.68 (18.22)	0.129 (0.095)	0 - 0.274
RG	200	100	0.90	0.60	0.9 (0.002)	0.368 (0.102)	0.002 (0.001)	0 - 0.006	0 (0)	-987.15 (18.96)	0.233 (0.102)	0.01 - 0.386
RG	200	100	0.90	0.70	0.9 (0.002)	0.362 (0.105)	0.002 (0.001)	0 - 0.006	0 (0)	-988.61 (19.94)	0.338 (0.105)	0.119 - 0.475
RG	200	100	0.90	0.80	0.9 (0.002)	0.376 (0.118)	0.002 (0.002)	0 - 0.008	0 (0)	-990.19 (22.49)	0.424 (0.118)	0.189 - 0.591
RG	200	100	0.90	0.90	0.9 (0.002)	0.373 (0.103)	0.002 (0.001)	0 - 0.006	0 (0)	-984.86 (17.49)	0.527 (0.103)	0.348 - 0.686
RG	500	16	0.10	0.10	0.13 (0.004)	0.785 (0.007)	0.03 (0.004)	0.021 - 0.041	0 (0)	-1663.32 (37.02)	0.685 (0.007)	0.655 - 0.695
RG	500	16	0.10	0.20	0.104 (0.004)	0.801 (0.06)	0.004 (0.003)	0 - 0.014	0 (0)	-1551.39 (28.5)	0.601 (0.06)	0.263 - 0.73
RG	500	16	0.10	0.30	0.104 (0.004)	0.787 (0.059)	0.004 (0.003)	0 - 0.017	0 (0)	-1556.82 (36.33)	0.487 (0.059)	0.227 - 0.637
RG	500	16	0.10	0.40	0.103 (0.003)	0.789 (0.053)	0.003 (0.002)	0 - 0.013	0 (0)	-1573.65 (53.04)	0.389 (0.053)	0.222 - 0.611
RG	500	16	0.10	0.50	0.101 (0.003)	0.792 (0.052)	0.003 (0.002)	0 - 0.013	0 (0)	-1600.88 (122.52)	0.292 (0.052)	0.056 - 0.4
RG	500	16	0.10	0.60	0.097 (0.006)	0.773 (0.078)	0.005 (0.005)	0 - 0.022	0 (0)	-1669.36 (174.88)	0.177 (0.068)	0.069 - 0.374
RG	500	16	0.10	0.70	0.096 (0.008)	0.788 (0.087)	0.006 (0.006)	0 - 0.021	0 (0)	-1667.92 (207.87)	0.101 (0.071)	0.002 - 0.257
RG	500	16	0.10	0.80	0.092 (0.011)	0.776 (0.091)	0.01 (0.01)	0 - 0.075	0 (0)	-1710.78 (193.72)	0.082 (0.045)	0 - 0.202

Continued on next page

Table S1 – *Continued from previous page*

Structure	T	N	p	p_e/p_w	$\overline{\hat{p}}$ (SD)	$\overline{\hat{p_e}}/\overline{\hat{p_w}}$ (SD)	$\overline{\lvert\Delta\rvert}_p$ (SD)	Range $\lvert\Delta\rvert_p$	$\overline{SE}\lvert\Delta\rvert_p$ (SD)	$\overline{BIC}$ (SD)	$\overline{\lvert\Delta\rvert}_{p_e/p_w}$ (SD)	Range $\lvert\Delta\rvert_{p_e/p_w}$
RG	500	16	0.10	0.90	0.09 (0.013)	0.777 (0.097)	0.011 (0.012)	0 - 0.075	0 (0)	-1711.79 (196.48)	0.135 (0.08)	0.005 - 0.214
RG	500	16	0.20	0.10	0.244 (0.006)	0.672 (0.059)	0.044 (0.006)	0.03 - 0.056	0 (0)	-1936.02 (28.5)	0.572 (0.059)	0.419 - 0.663
RG	500	16	0.20	0.20	0.211 (0.005)	0.734 (0.032)	0.011 (0.005)	0 - 0.024	0 (0)	-1886.77 (27.6)	0.534 (0.032)	0.424 - 0.679
RG	500	16	0.20	0.30	0.217 (0.006)	0.723 (0.033)	0.017 (0.006)	0.001 - 0.03	0 (0)	-1905.41 (35.09)	0.423 (0.033)	0.31 - 0.576
RG	500	16	0.20	0.40	0.208 (0.006)	0.692 (0.055)	0.008 (0.005)	0 - 0.021	0 (0)	-1885.7 (39.76)	0.292 (0.055)	0.114 - 0.354
RG	500	16	0.20	0.50	0.206 (0.007)	0.682 (0.056)	0.007 (0.005)	0 - 0.024	0 (0)	-1885.44 (46.59)	0.182 (0.056)	0.006 - 0.254
RG	500	16	0.20	0.60	0.203 (0.006)	0.674 (0.051)	0.005 (0.004)	0 - 0.022	0 (0)	-1880.4 (44.74)	0.079 (0.042)	0.007 - 0.156
RG	500	16	0.20	0.70	0.203 (0.006)	0.672 (0.051)	0.005 (0.004)	0 - 0.019	0 (0)	-1885.58 (42.71)	0.047 (0.034)	0 - 0.2
RG	500	16	0.20	0.80	0.2 (0.006)	0.666 (0.064)	0.005 (0.003)	0 - 0.014	0 (0)	-1891.05 (73.73)	0.135 (0.061)	0.013 - 0.287
RG	500	16	0.20	0.90	0.197 (0.005)	0.651 (0.066)	0.005 (0.004)	0 - 0.016	0 (0)	-1904.35 (103.15)	0.249 (0.066)	0.087 - 0.396
RG	500	16	0.30	0.10	0.342 (0.006)	0.65 (0.017)	0.042 (0.006)	0.027 - 0.058	0 (0)	-2034.36 (29.83)	0.55 (0.017)	0.475 - 0.583
RG	500	16	0.30	0.20	0.315 (0.006)	0.66 (0.015)	0.015 (0.006)	0 - 0.029	0 (0)	-2023.43 (36.92)	0.46 (0.015)	0.414 - 0.534
RG	500	16	0.30	0.30	0.326 (0.008)	0.661 (0.01)	0.026 (0.007)	0.004 - 0.04	0 (0)	-2037.22 (33.18)	0.361 (0.01)	0.281 - 0.371
RG	500	16	0.30	0.40	0.321 (0.007)	0.662 (0.006)	0.021 (0.007)	0.004 - 0.035	0 (0)	-2044.78 (33.79)	0.262 (0.006)	0.248 - 0.271
RG	500	16	0.30	0.50	0.319 (0.007)	0.66 (0.008)	0.019 (0.007)	0.004 - 0.032	0 (0)	-2049.31 (36.98)	0.16 (0.008)	0.113 - 0.17
RG	500	16	0.30	0.60	0.315 (0.007)	0.66 (0.01)	0.015 (0.007)	0.001 - 0.029	0 (0)	-2055 (42.12)	0.06 (0.01)	0.009 - 0.071
RG	500	16	0.30	0.70	0.313 (0.007)	0.656 (0.014)	0.014 (0.006)	0 - 0.026	0 (0)	-2069.65 (43.14)	0.044 (0.014)	0.029 - 0.11
RG	500	16	0.30	0.80	0.309 (0.007)	0.656 (0.015)	0.01 (0.006)	0 - 0.024	0 (0)	-2090.42 (51.95)	0.144 (0.015)	0.129 - 0.206
RG	500	16	0.30	0.90	0.305 (0.007)	0.655 (0.013)	0.006 (0.005)	0 - 0.02	0 (0)	-2097.85 (56.78)	0.245 (0.013)	0.228 - 0.296
RG	500	16	0.40	0.10	0.421 (0.007)	0.61 (0.067)	0.021 (0.007)	0.004 - 0.046	0 (0)	-2093.06 (29.69)	0.51 (0.067)	0.472 - 0.899

Continued on next page

Table S1 – *Continued from previous page*

Structure	T	N	p	p_e/p_w	$\overline{\hat{p}}$ (SD)	$\overline{\hat{p_e}}/\overline{\hat{p_w}}$ (SD)	$\overline{\lvert\Delta\rvert}_p$ (SD)	Range $\lvert\Delta\rvert_p$	$\overline{SE}\lvert\Delta\rvert_p$ (SD)	$\overline{BIC}$ (SD)	$\overline{\lvert\Delta\rvert}_{p_e/p_w}$ (SD)	Range $\lvert\Delta\rvert_{p_e/p_w}$
RG	500	16	0.40	0.20	0.412 (0.006)	0.702 (0.096)	0.012 (0.006)	0.001 - 0.023	0 (0)	-2083.61 (29.23)	0.502 (0.096)	0.372 - 0.827
RG	500	16	0.40	0.30	0.414 (0.006)	0.598 (0.028)	0.014 (0.006)	0 - 0.03	0 (0)	-2080.26 (29.29)	0.298 (0.028)	0.272 - 0.378
RG	500	16	0.40	0.40	0.411 (0.007)	0.594 (0.021)	0.012 (0.006)	0 - 0.029	0 (0)	-2081.16 (28.1)	0.194 (0.021)	0.172 - 0.283
RG	500	16	0.40	0.50	0.408 (0.008)	0.592 (0.018)	0.01 (0.006)	0 - 0.027	0 (0)	-2080.63 (30.04)	0.092 (0.018)	0.014 - 0.112
RG	500	16	0.40	0.60	0.404 (0.009)	0.58 (0.028)	0.008 (0.006)	0 - 0.029	0 (0)	-2084.95 (26.03)	0.024 (0.024)	0 - 0.099
RG	500	16	0.40	0.70	0.402 (0.01)	0.575 (0.027)	0.008 (0.006)	0 - 0.025	0 (0)	-2088.8 (33.17)	0.125 (0.027)	0.082 - 0.185
RG	500	16	0.40	0.80	0.397 (0.008)	0.57 (0.03)	0.007 (0.005)	0 - 0.02	0 (0)	-2093.35 (30.86)	0.23 (0.03)	0.179 - 0.3
RG	500	16	0.40	0.90	0.394 (0.007)	0.583 (0.027)	0.007 (0.005)	0 - 0.02	0 (0)	-2105.61 (35.27)	0.317 (0.027)	0.277 - 0.388
RG	500	16	0.50	0.10	0.5 (0.011)	0.55 (0.155)	0.009 (0.005)	0 - 0.027	0 (0)	-2091.2 (28.58)	0.45 (0.155)	0.143 - 1.211
RG	500	16	0.50	0.20	0.502 (0.01)	0.566 (0.156)	0.009 (0.005)	0.001 - 0.024	0 (0)	-2090.46 (28.87)	0.369 (0.147)	0.004 - 0.894
RG	500	16	0.50	0.30	0.502 (0.011)	0.599 (0.164)	0.009 (0.005)	0 - 0.026	0 (0)	-2092.85 (27.64)	0.299 (0.164)	0.004 - 1.145
RG	500	16	0.50	0.40	0.499 (0.01)	0.591 (0.178)	0.009 (0.004)	0 - 0.022	0 (0)	-2093.89 (34.23)	0.194 (0.175)	0.015 - 1.228
RG	500	16	0.50	0.50	0.5 (0.01)	0.548 (0.182)	0.009 (0.005)	0 - 0.026	0 (0)	-2094.87 (29.25)	0.119 (0.146)	0 - 0.631
RG	500	16	0.50	0.60	0.501 (0.01)	0.553 (0.113)	0.009 (0.005)	0.001 - 0.027	0 (0)	-2091.68 (28.25)	0.089 (0.084)	0.001 - 0.505
RG	500	16	0.50	0.70	0.5 (0.01)	0.556 (0.141)	0.009 (0.005)	0 - 0.026	0 (0)	-2092.87 (30.69)	0.174 (0.101)	0.013 - 0.668
RG	500	16	0.50	0.80	0.501 (0.011)	0.575 (0.146)	0.01 (0.006)	0 - 0.026	0 (0)	-2093.51 (33.16)	0.254 (0.086)	0.018 - 0.547
RG	500	16	0.50	0.90	0.5 (0.01)	0.593 (0.152)	0.009 (0.005)	0 - 0.025	0 (0)	-2092.39 (29.97)	0.319 (0.124)	0.032 - 0.884
RG	500	16	0.60	0.10	0.559 (0.013)	0.759 (0.606)	0.041 (0.013)	0.017 - 0.079	0 (0)	-2114.51 (49.88)	0.659 (0.606)	0.41 - 6.067
RG	500	16	0.60	0.20	0.563 (0.018)	0.653 (0.177)	0.037 (0.018)	0.001 - 0.063	0 (0)	-2144.58 (63.24)	0.453 (0.177)	0.31 - 1.769

Continued on next page

Table S1 – *Continued from previous page*

Structure	T	N	p	p_e/p_w	$\overline{\hat{p}}$ (SD)	$\overline{\hat{p_e}}/\overline{\hat{p_w}}$ (SD)	$\overline{\lvert\Delta\rvert}_p$ (SD)	Range $\lvert\Delta\rvert_p$	$\overline{SE}\lvert\Delta\rvert_p$ (SD)	$\overline{BIC}$ (SD)	$\overline{\lvert\Delta\rvert}_{p_e/p_w}$ (SD)	Range $\lvert\Delta\rvert_{p_e/p_w}$
RG	500	16	0.60	0.30	0.575 (0.01)	0.617 (0.048)	0.025 (0.01)	0.008 - 0.063	0 (0)	-2089.89 (32.05)	0.317 (0.048)	0.215 - 0.443
RG	500	16	0.60	0.40	0.584 (0.009)	0.585 (0.039)	0.016 (0.008)	0 - 0.038	0 (0)	-2090.73 (28.77)	0.185 (0.039)	0.11 - 0.298
RG	500	16	0.60	0.50	0.593 (0.008)	0.556 (0.036)	0.009 (0.006)	0 - 0.03	0 (0)	-2088.32 (28.42)	0.056 (0.036)	0.01 - 0.124
RG	500	16	0.60	0.60	0.605 (0.009)	0.538 (0.02)	0.008 (0.006)	0 - 0.025	0 (0)	-2086.38 (27.36)	0.063 (0.017)	0.002 - 0.089
RG	500	16	0.60	0.70	0.608 (0.009)	0.538 (0.021)	0.01 (0.006)	0 - 0.033	0 (0)	-2087.56 (26.23)	0.162 (0.021)	0.084 - 0.189
RG	500	16	0.60	0.80	0.618 (0.008)	0.537 (0.01)	0.018 (0.008)	0.001 - 0.039	0 (0)	-2085.53 (27.98)	0.263 (0.01)	0.226 - 0.288
RG	500	16	0.60	0.90	0.624 (0.008)	0.535 (0.011)	0.024 (0.008)	0.006 - 0.042	0 (0)	-2088.51 (30.45)	0.365 (0.011)	0.345 - 0.389
RG	500	16	0.70	0.10	0.478 (0.039)	0.576 (0.512)	0.222 (0.039)	0.066 - 0.313	0 (0)	-2405.21 (134.55)	0.483 (0.505)	0.006 - 4.647
RG	500	16	0.70	0.20	0.577 (0.094)	0.76 (0.623)	0.123 (0.094)	0.041 - 0.313	0 (0)	-2153.1 (165.16)	0.59 (0.595)	0.167 - 5.457
RG	500	16	0.70	0.30	0.643 (0.011)	0.626 (0.04)	0.057 (0.011)	0.036 - 0.107	0 (0)	-2082.44 (45.61)	0.326 (0.04)	0.2 - 0.383
RG	500	16	0.70	0.40	0.67 (0.011)	0.591 (0.024)	0.03 (0.011)	0.01 - 0.061	0 (0)	-2059.2 (37.51)	0.191 (0.024)	0.11 - 0.274
RG	500	16	0.70	0.50	0.684 (0.008)	0.567 (0.036)	0.016 (0.008)	0.003 - 0.038	0 (0)	-2055.61 (30.91)	0.067 (0.036)	0.01 - 0.125
RG	500	16	0.70	0.60	0.705 (0.007)	0.546 (0.008)	0.007 (0.005)	0 - 0.024	0 (0)	-2046.06 (30.64)	0.054 (0.008)	0.042 - 0.09
RG	500	16	0.70	0.70	0.708 (0.007)	0.546 (0.007)	0.009 (0.006)	0 - 0.029	0 (0)	-2038.77 (30.7)	0.154 (0.007)	0.141 - 0.179
RG	500	16	0.70	0.80	0.72 (0.005)	0.536 (0.017)	0.02 (0.005)	0.002 - 0.032	0 (0)	-2036.45 (27.91)	0.264 (0.017)	0.24 - 0.292
RG	500	16	0.70	0.90	0.725 (0.006)	0.52 (0.012)	0.025 (0.006)	0.009 - 0.037	0 (0)	-2058.6 (30.54)	0.38 (0.012)	0.34 - 0.395
RG	500	16	0.80	0.10	0.366 (0.013)	0.506 (0.023)	0.434 (0.013)	0.413 - 0.472	0 (0)	-2538.23 (120.52)	0.406 (0.023)	0.299 - 0.439
RG	500	16	0.80	0.20	0.503 (0.092)	0.563 (0.141)	0.297 (0.092)	0.091 - 0.446	0 (0)	-2666.45 (313.6)	0.372 (0.117)	0.133 - 0.698
RG	500	16	0.80	0.30	0.71 (0.014)	0.618 (0.066)	0.09 (0.014)	0.062 - 0.114	0 (0)	-2115.78 (127.34)	0.318 (0.066)	0.21 - 0.396
RG	500	16	0.80	0.40	0.767 (0.007)	0.606 (0.013)	0.033 (0.007)	0.017 - 0.047	0 (0)	-1970.31 (29.35)	0.206 (0.013)	0.177 - 0.222

Continued on next page

Table S1 – *Continued from previous page*

Structure	T	N	p	p_e/p_w	$\overline{\hat{p}}$ (SD)	$\overline{\hat{p_e}}/\overline{\hat{p_w}}$ (SD)	$\overline{\lvert\Delta\rvert}_p$ (SD)	Range $\lvert\Delta\rvert_p$	$\overline{SE}\lvert\Delta\rvert_p$ (SD)	$\overline{BIC}$ (SD)	$\overline{\lvert\Delta\rvert}_{p_e/p_w}$ (SD)	Range $\lvert\Delta\rvert_{p_e/p_w}$
RG	500	16	0.80	0.50	0.79 (0.006)	0.577 (0.029)	0.01 (0.006)	0 - 0.027	0 (0)	-1942.44 (33.63)	0.077 (0.029)	0.012 - 0.129
RG	500	16	0.80	0.60	0.804 (0.005)	0.539 (0.015)	0.005 (0.004)	0 - 0.016	0 (0)	-1899.71 (37.09)	0.061 (0.015)	0.039 - 0.089
RG	500	16	0.80	0.70	0.804 (0.004)	0.536 (0.014)	0.005 (0.004)	0 - 0.018	0 (0)	-1894.9 (33.38)	0.164 (0.014)	0.143 - 0.187
RG	500	16	0.80	0.80	0.809 (0.005)	0.512 (0.04)	0.01 (0.004)	0.001 - 0.02	0 (0)	-1899.62 (32.4)	0.288 (0.04)	0.24 - 0.396
RG	500	16	0.80	0.90	0.81 (0.004)	0.473 (0.04)	0.01 (0.004)	0.002 - 0.018	0 (0)	-1919.28 (42.19)	0.427 (0.04)	0.343 - 0.51
RG	500	16	0.90	0.10	0.288 (0.003)	0.163 (0.11)	0.612 (0.003)	0.591 - 0.618	0 (0)	-1922.73 (108.44)	0.103 (0.073)	0.006 - 0.497
RG	500	16	0.90	0.20	0.478 (0.102)	0.539 (0.123)	0.422 (0.102)	0.139 - 0.568	0 (0)	-2918.92 (394.22)	0.345 (0.105)	0.019 - 0.623
RG	500	16	0.90	0.30	0.825 (0.012)	0.643 (0.03)	0.075 (0.012)	0.053 - 0.104	0 (0)	-1915.54 (58.3)	0.343 (0.03)	0.284 - 0.383
RG	500	16	0.90	0.40	0.878 (0.007)	0.558 (0.06)	0.022 (0.007)	0.009 - 0.037	0 (0)	-1704.28 (59.92)	0.158 (0.06)	0.05 - 0.281
RG	500	16	0.90	0.50	0.895 (0.004)	0.479 (0.031)	0.006 (0.004)	0 - 0.015	0 (0)	-1595.86 (43.45)	0.034 (0.016)	0.008 - 0.111
RG	500	16	0.90	0.60	0.899 (0.004)	0.483 (0.042)	0.003 (0.002)	0 - 0.01	0 (0)	-1551.28 (34.62)	0.117 (0.042)	0.046 - 0.208
RG	500	16	0.90	0.70	0.9 (0.004)	0.465 (0.044)	0.003 (0.002)	0 - 0.01	0 (0)	-1549.81 (31.88)	0.235 (0.044)	0.149 - 0.313
RG	500	16	0.90	0.80	0.901 (0.003)	0.442 (0.036)	0.003 (0.002)	0 - 0.012	0 (0)	-1540.9 (31.85)	0.358 (0.036)	0.264 - 0.424
RG	500	16	0.90	0.90	0.901 (0.003)	0.434 (0.035)	0.003 (0.002)	0 - 0.01	0 (0)	-1537.25 (31.39)	0.466 (0.035)	0.348 - 0.516
RG	500	25	0.10	0.10	0.121 (0.004)	0.776 (0.07)	0.021 (0.004)	0.012 - 0.035	0 (0)	-1871.16 (41.02)	0.676 (0.07)	0.392 - 0.725
RG	500	25	0.10	0.20	0.102 (0.003)	0.782 (0.088)	0.003 (0.002)	0 - 0.01	0 (0)	-1793.98 (28.93)	0.582 (0.088)	0.335 - 0.671
RG	500	25	0.10	0.30	0.101 (0.003)	0.745 (0.113)	0.003 (0.002)	0 - 0.009	0 (0)	-1790.19 (39.27)	0.445 (0.113)	0.203 - 0.575
RG	500	25	0.10	0.40	0.1 (0.003)	0.714 (0.138)	0.002 (0.002)	0 - 0.007	0 (0)	-1780.53 (30.1)	0.314 (0.138)	0.131 - 0.475
RG	500	25	0.10	0.50	0.1 (0.004)	0.676 (0.136)	0.003 (0.002)	0 - 0.008	0 (0)	-1783.81 (28.45)	0.177 (0.135)	0.002 - 0.364
RG	500	25	0.10	0.60	0.1 (0.003)	0.707 (0.154)	0.002 (0.002)	0 - 0.008	0 (0)	-1780.18 (27.66)	0.156 (0.104)	0.005 - 0.275

Continued on next page

Table S1 – *Continued from previous page*

Structure	T	N	p	p_e/p_w	$\overline{\hat{p}}$ (SD)	$\overline{\hat{p}_e}/\overline{\hat{p}_w}$ (SD)	$\overline{\lvert\Delta\rvert}_p$ (SD)	Range $\lvert\Delta\rvert_p$	$\overline{SE}\lvert\Delta\rvert_p$ (SD)	$\overline{BIC}$ (SD)	$\overline{\lvert\Delta\rvert}_{p_e/p_w}$ (SD)	Range $\lvert\Delta\rvert_{p_e/p_w}$
RG	500	25	0.10	0.70	0.099 (0.003)	0.701 (0.152)	0.003 (0.002)	0 - 0.009	0 (0)	-1779.89 (30.01)	0.146 (0.036)	0.064 - 0.25
RG	500	25	0.10	0.80	0.099 (0.003)	0.695 (0.158)	0.003 (0.002)	0 - 0.008	0 (0)	-1782.48 (29.82)	0.154 (0.11)	0.021 - 0.468
RG	500	25	0.10	0.90	0.099 (0.003)	0.684 (0.167)	0.002 (0.002)	0 - 0.008	0 (0)	-1773.85 (30.51)	0.216 (0.167)	0.029 - 0.692
RG	500	25	0.20	0.10	0.235 (0.006)	0.581 (0.077)	0.035 (0.006)	0.025 - 0.056	0 (0)	-2154.76 (32.68)	0.481 (0.077)	0.383 - 0.71
RG	500	25	0.20	0.20	0.211 (0.004)	0.763 (0.069)	0.011 (0.004)	0 - 0.019	0 (0)	-2126.13 (37.86)	0.563 (0.069)	0.337 - 0.711
RG	500	25	0.20	0.30	0.206 (0.004)	0.719 (0.099)	0.006 (0.004)	0 - 0.016	0 (0)	-2121.38 (37.4)	0.419 (0.099)	0.198 - 0.517
RG	500	25	0.20	0.40	0.205 (0.004)	0.704 (0.105)	0.005 (0.003)	0 - 0.013	0 (0)	-2108.96 (34.04)	0.304 (0.105)	0.135 - 0.431
RG	500	25	0.20	0.50	0.201 (0.004)	0.705 (0.108)	0.004 (0.003)	0 - 0.014	0 (0)	-2102.67 (34.62)	0.205 (0.108)	0.005 - 0.326
RG	500	25	0.20	0.60	0.199 (0.004)	0.684 (0.109)	0.003 (0.003)	0 - 0.016	0 (0)	-2104.26 (49.34)	0.103 (0.091)	0 - 0.228
RG	500	25	0.20	0.70	0.199 (0.005)	0.675 (0.128)	0.004 (0.003)	0 - 0.014	0 (0)	-2099.23 (40.5)	0.124 (0.04)	0.049 - 0.233
RG	500	25	0.20	0.80	0.198 (0.005)	0.638 (0.153)	0.004 (0.004)	0 - 0.018	0 (0)	-2105.58 (55.46)	0.173 (0.14)	0 - 0.465
RG	500	25	0.20	0.90	0.198 (0.004)	0.636 (0.188)	0.003 (0.003)	0 - 0.015	0 (0)	-2098.96 (61.1)	0.264 (0.188)	0.066 - 0.728
RG	500	25	0.30	0.10	0.343 (0.006)	0.655 (0.032)	0.043 (0.006)	0.025 - 0.054	0 (0)	-2270.25 (32.04)	0.555 (0.032)	0.481 - 0.758
RG	500	25	0.30	0.20	0.32 (0.005)	0.612 (0.035)	0.02 (0.005)	0.007 - 0.034	0 (0)	-2265.99 (34.36)	0.412 (0.035)	0.331 - 0.503
RG	500	25	0.30	0.30	0.313 (0.007)	0.569 (0.037)	0.013 (0.007)	0.001 - 0.034	0 (0)	-2254.91 (31.09)	0.269 (0.037)	0.232 - 0.372
RG	500	25	0.30	0.40	0.313 (0.006)	0.572 (0.034)	0.013 (0.006)	0.001 - 0.03	0 (0)	-2266.74 (38.67)	0.172 (0.034)	0.127 - 0.276
RG	500	25	0.30	0.50	0.303 (0.007)	0.578 (0.046)	0.006 (0.005)	0 - 0.021	0 (0)	-2255.86 (36.62)	0.078 (0.046)	0.026 - 0.205

Continued on next page

Table S1 – *Continued from previous page*

Structure	T	N	p	p_e/p_w	$\overline{\hat{p}}$ (SD)	$\overline{\hat{p_e}}/\overline{\hat{p_w}}$ (SD)	$\overline{\lvert\Delta\rvert}_p$ (SD)	Range $\lvert\Delta\rvert_p$	$\overline{SE}\lvert\Delta\rvert_p$ (SD)	$\overline{BIC}$ (SD)	$\overline{\lvert\Delta\rvert}_{p_e/p_w}$ (SD)	Range $\lvert\Delta\rvert_{p_e/p_w}$
RG	500	25	0.30	0.60	0.302 (0.006)	0.574 (0.033)	0.005 (0.004)	0 - 0.017	0 (0)	-2256.86 (42.07)	0.035 (0.024)	0.001 - 0.079
RG	500	25	0.30	0.70	0.3 (0.006)	0.58 (0.033)	0.005 (0.003)	0 - 0.016	0 (0)	-2261.19 (42.83)	0.121 (0.033)	0.01 - 0.204
RG	500	25	0.30	0.80	0.296 (0.006)	0.572 (0.034)	0.005 (0.005)	0 - 0.023	0 (0)	-2282.57 (46.14)	0.228 (0.034)	0.096 - 0.307
RG	500	25	0.30	0.90	0.294 (0.006)	0.575 (0.034)	0.006 (0.005)	0 - 0.021	0 (0)	-2293.99 (48.17)	0.325 (0.034)	0.186 - 0.413
RG	500	25	0.40	0.10	0.422 (0.006)	0.609 (0.045)	0.022 (0.006)	0.009 - 0.037	0 (0)	-2311.8 (31.49)	0.509 (0.045)	0.473 - 0.7
RG	500	25	0.40	0.20	0.415 (0.006)	0.596 (0.03)	0.015 (0.006)	0.002 - 0.035	0 (0)	-2309.29 (28.86)	0.396 (0.03)	0.366 - 0.511
RG	500	25	0.40	0.30	0.416 (0.005)	0.583 (0.008)	0.016 (0.005)	0.002 - 0.029	0 (0)	-2312.09 (34.17)	0.283 (0.008)	0.26 - 0.296
RG	500	25	0.40	0.40	0.416 (0.005)	0.579 (0.019)	0.016 (0.005)	0.001 - 0.025	0 (0)	-2316.02 (29.97)	0.179 (0.019)	0.095 - 0.197
RG	500	25	0.40	0.50	0.408 (0.007)	0.582 (0.012)	0.009 (0.005)	0 - 0.022	0 (0)	-2315.54 (32.27)	0.082 (0.012)	0.03 - 0.096
RG	500	25	0.40	0.60	0.408 (0.007)	0.582 (0.01)	0.009 (0.005)	0 - 0.024	0 (0)	-2322.56 (33.98)	0.018 (0.01)	0.004 - 0.064
RG	500	25	0.40	0.70	0.405 (0.006)	0.582 (0.012)	0.006 (0.005)	0 - 0.021	0 (0)	-2334.62 (30.07)	0.118 (0.012)	0.104 - 0.205
RG	500	25	0.40	0.80	0.403 (0.007)	0.583 (0.01)	0.006 (0.005)	0 - 0.018	0 (0)	-2349.99 (35.32)	0.217 (0.01)	0.205 - 0.293
RG	500	25	0.40	0.90	0.4 (0.005)	0.583 (0.007)	0.004 (0.003)	0 - 0.012	0 (0)	-2374.43 (40.68)	0.317 (0.007)	0.304 - 0.328
RG	500	25	0.50	0.10	0.499 (0.008)	0.57 (0.142)	0.007 (0.004)	0.001 - 0.024	0 (0)	-2319.01 (27.71)	0.471 (0.14)	0.029 - 0.892
RG	500	25	0.50	0.20	0.501 (0.008)	0.584 (0.141)	0.007 (0.004)	0.001 - 0.022	0 (0)	-2318.42 (33.23)	0.384 (0.141)	0.138 - 0.851
RG	500	25	0.50	0.30	0.5 (0.009)	0.607 (0.173)	0.007 (0.004)	0.001 - 0.023	0 (0)	-2312.26 (34.66)	0.307 (0.172)	0.016 - 0.89
RG	500	25	0.50	0.40	0.5 (0.009)	0.584 (0.179)	0.008 (0.004)	0 - 0.018	0 (0)	-2314.39 (28.56)	0.196 (0.165)	0.011 - 0.959
RG	500	25	0.50	0.50	0.501 (0.008)	0.604 (0.178)	0.007 (0.004)	0 - 0.021	0 (0)	-2315.45 (31.42)	0.139 (0.152)	0.001 - 0.587
RG	500	25	0.50	0.60	0.499 (0.009)	0.582 (0.141)	0.008 (0.005)	0 - 0.025	0 (0)	-2314.97 (33.73)	0.103 (0.098)	0 - 0.542
RG	500	25	0.50	0.70	0.501 (0.008)	0.571 (0.154)	0.007 (0.004)	0.001 - 0.024	0 (0)	-2312.85 (29.98)	0.174 (0.101)	0.002 - 0.661

Continued on next page

Table S1 – *Continued from previous page*

Structure	T	N	p	p_e/p_w	$\overline{\hat{p}}$ (SD)	$\overline{\hat{p}_e}/\overline{\hat{p}_w}$ (SD)	$\overline{\lvert\Delta\rvert}_p$ (SD)	Range $\lvert\Delta\rvert_p$	$\overline{SE}\lvert\Delta\rvert_p$ (SD)	$\overline{BIC}$ (SD)	$\overline{\lvert\Delta\rvert}_{p_e/p_w}$ (SD)	Range $\lvert\Delta\rvert_{p_e/p_w}$
RG	500	25	0.50	0.80	0.5 (0.009)	0.589 (0.143)	0.007 (0.005)	0 - 0.023	0 (0)	-2313.89 (30.26)	0.231 (0.108)	0.001 - 0.59
RG	500	25	0.50	0.90	0.5 (0.008)	0.581 (0.173)	0.008 (0.004)	0 - 0.021	0 (0)	-2318.91 (35.47)	0.337 (0.134)	0.069 - 0.862
RG	500	25	0.60	0.10	0.563 (0.006)	0.721 (0.047)	0.037 (0.006)	0.023 - 0.051	0 (0)	-2310.37 (29.53)	0.621 (0.047)	0.551 - 0.89
RG	500	25	0.60	0.20	0.57 (0.007)	0.644 (0.022)	0.03 (0.007)	0.014 - 0.043	0 (0)	-2317.05 (32.07)	0.444 (0.022)	0.377 - 0.516
RG	500	25	0.60	0.30	0.579 (0.007)	0.581 (0.014)	0.021 (0.007)	0.003 - 0.036	0 (0)	-2312.83 (34.25)	0.281 (0.014)	0.23 - 0.308
RG	500	25	0.60	0.40	0.59 (0.008)	0.542 (0.009)	0.011 (0.006)	0 - 0.031	0 (0)	-2308.72 (33.66)	0.142 (0.009)	0.126 - 0.18
RG	500	25	0.60	0.50	0.601 (0.007)	0.545 (0.014)	0.006 (0.004)	0 - 0.021	0 (0)	-2302.99 (30.23)	0.045 (0.014)	0.028 - 0.1
RG	500	25	0.60	0.60	0.603 (0.007)	0.54 (0.008)	0.006 (0.004)	0 - 0.016	0 (0)	-2312.23 (30.37)	0.06 (0.008)	0.046 - 0.081
RG	500	25	0.60	0.70	0.612 (0.006)	0.535 (0.017)	0.012 (0.006)	0.002 - 0.03	0 (0)	-2308.73 (30.87)	0.165 (0.017)	0.146 - 0.216
RG	500	25	0.60	0.80	0.618 (0.006)	0.514 (0.022)	0.018 (0.006)	0.008 - 0.035	0 (0)	-2315.12 (30.77)	0.286 (0.022)	0.246 - 0.317
RG	500	25	0.60	0.90	0.623 (0.005)	0.507 (0.016)	0.023 (0.005)	0.011 - 0.033	0 (0)	-2323.45 (28.92)	0.393 (0.016)	0.346 - 0.416
RG	500	25	0.70	0.10	0.605 (0.025)	1.069 (0.765)	0.095 (0.025)	0.067 - 0.296	0 (0)	-2324.24 (41.67)	0.969 (0.765)	0.593 - 7.822
RG	500	25	0.70	0.20	0.633 (0.007)	0.663 (0.012)	0.067 (0.007)	0.052 - 0.085	0 (0)	-2314.44 (37.39)	0.463 (0.012)	0.42 - 0.478
RG	500	25	0.70	0.30	0.665 (0.007)	0.588 (0.007)	0.035 (0.007)	0.018 - 0.05	0 (0)	-2289.38 (30.65)	0.288 (0.007)	0.268 - 0.302
RG	500	25	0.70	0.40	0.685 (0.006)	0.542 (0.007)	0.015 (0.006)	0 - 0.029	0 (0)	-2273.88 (27.19)	0.142 (0.007)	0.128 - 0.157
RG	500	25	0.70	0.50	0.699 (0.006)	0.541 (0.007)	0.005 (0.004)	0 - 0.015	0 (0)	-2256.07 (30.39)	0.041 (0.007)	0.023 - 0.061
RG	500	25	0.70	0.60	0.703 (0.006)	0.525 (0.035)	0.005 (0.003)	0 - 0.015	0 (0)	-2284.75 (61.63)	0.075 (0.035)	0.045 - 0.154
RG	500	25	0.70	0.70	0.704 (0.006)	0.474 (0.031)	0.005 (0.004)	0 - 0.02	0 (0)	-2355.29 (61.86)	0.226 (0.031)	0.146 - 0.254

Continued on next page

Table S1 – *Continued from previous page*

Structure	T	N	p	p_e/p_w	$\overline{\hat{p}}$ (SD)	$\overline{\hat{p_e}}/\overline{\hat{p_w}}$ (SD)	$\overline{\lvert\Delta\rvert}_p$ (SD)	Range $\lvert\Delta\rvert_p$	$\overline{SE}\lvert\Delta\rvert_p$ (SD)	$\overline{BIC}$ (SD)	$\overline{\lvert\Delta\rvert}_{p_e/p_w}$ (SD)	Range $\lvert\Delta\rvert_{p_e/p_w}$
RG	500	25	0.70	0.80	0.712 (0.006)	0.488 (0.021)	0.012 (0.006)	0 - 0.028	0 (0)	-2298.73 (58.68)	0.312 (0.021)	0.282 - 0.35
RG	500	25	0.70	0.90	0.716 (0.004)	0.498 (0.01)	0.016 (0.004)	0.005 - 0.026	0 (0)	-2294.81 (37.92)	0.402 (0.01)	0.361 - 0.435
RG	500	25	0.80	0.10	0.467 (0.124)	0.567 (1.146)	0.333 (0.124)	0.117 - 0.429	0 (0)	-2497.64 (195.04)	0.53 (1.118)	0.002 - 10.059
RG	500	25	0.80	0.20	0.714 (0.007)	0.667 (0.009)	0.086 (0.007)	0.066 - 0.102	0 (0)	-2330.2 (37.93)	0.467 (0.009)	0.45 - 0.5
RG	500	25	0.80	0.30	0.773 (0.006)	0.585 (0.009)	0.027 (0.006)	0.01 - 0.043	0 (0)	-2210.85 (34.76)	0.285 (0.009)	0.269 - 0.331
RG	500	25	0.80	0.40	0.792 (0.005)	0.547 (0.02)	0.008 (0.004)	0 - 0.02	0 (0)	-2129.53 (36.94)	0.147 (0.02)	0.089 - 0.198
RG	500	25	0.80	0.50	0.8 (0.004)	0.546 (0.015)	0.003 (0.002)	0 - 0.01	0 (0)	-2106.95 (32.43)	0.046 (0.015)	0.005 - 0.09
RG	500	25	0.80	0.60	0.802 (0.004)	0.53 (0.019)	0.003 (0.003)	0 - 0.012	0 (0)	-2094.81 (31.53)	0.07 (0.019)	0.042 - 0.108
RG	500	25	0.80	0.70	0.803 (0.004)	0.503 (0.012)	0.004 (0.003)	0 - 0.011	0 (0)	-2096.31 (35)	0.197 (0.012)	0.15 - 0.218
RG	500	25	0.80	0.80	0.804 (0.004)	0.501 (0.011)	0.005 (0.003)	0 - 0.012	0 (0)	-2103.36 (33.84)	0.299 (0.011)	0.246 - 0.34
RG	500	25	0.80	0.90	0.805 (0.003)	0.491 (0.016)	0.005 (0.003)	0 - 0.012	0 (0)	-2100.77 (31.37)	0.409 (0.016)	0.388 - 0.448
RG	500	25	0.90	0.10	0.382 (0.06)	0.292 (0.253)	0.518 (0.06)	0.171 - 0.557	0 (0)	-2837.16 (429.63)	0.253 (0.192)	0.005 - 1.01
RG	500	25	0.90	0.20	0.837 (0.011)	0.718 (0.018)	0.063 (0.011)	0.032 - 0.091	0 (0)	-2285.9 (57.25)	0.518 (0.018)	0.491 - 0.56
RG	500	25	0.90	0.30	0.894 (0.004)	0.488 (0.098)	0.006 (0.003)	0 - 0.015	0 (0)	-1847.61 (39.13)	0.188 (0.098)	0.102 - 0.363
RG	500	25	0.90	0.40	0.899 (0.003)	0.417 (0.023)	0.002 (0.002)	0 - 0.007	0 (0)	-1789.57 (33.94)	0.017 (0.023)	0.001 - 0.228
RG	500	25	0.90	0.50	0.899 (0.002)	0.414 (0.02)	0.002 (0.001)	0 - 0.006	0 (0)	-1782.5 (30.32)	0.088 (0.009)	0.076 - 0.139
RG	500	25	0.90	0.60	0.901 (0.003)	0.415 (0.017)	0.002 (0.002)	0 - 0.007	0 (0)	-1773.71 (34.52)	0.185 (0.017)	0.044 - 0.219
RG	500	25	0.90	0.70	0.9 (0.003)	0.413 (0.016)	0.002 (0.002)	0 - 0.006	0 (0)	-1773.9 (29.53)	0.287 (0.016)	0.165 - 0.33
RG	500	25	0.90	0.80	0.901 (0.002)	0.411 (0.013)	0.002 (0.001)	0 - 0.007	0 (0)	-1776.25 (29.78)	0.389 (0.013)	0.327 - 0.462

Continued on next page

Table S1 – *Continued from previous page*

Structure	T	N	p	p_e/p_w	$\overline{\hat{p}}$ (SD)	$\overline{\hat{p_e}}/\overline{\hat{p_w}}$ (SD)	$\overline{\lvert\Delta\rvert}_p$ (SD)	Range $\lvert\Delta\rvert_p$	$\overline{SE}\lvert\Delta\rvert_p$ (SD)	$\overline{BIC}$ (SD)	$\overline{\lvert\Delta\rvert}_{p_e/p_w}$ (SD)	Range $\lvert\Delta\rvert_{p_e/p_w}$
RG	500	25	0.90	0.90	0.901 (0.003)	0.411 (0.012)	0.002 (0.002)	0 - 0.007	0 (0)	-1770.46 (29.6)	0.489 (0.012)	0.448 - 0.535
RG	500	49	0.10	0.10	0.103 (0.002)	0.734 (0.116)	0.003 (0.002)	0 - 0.009	0 (0)	-2172.71 (45.6)	0.634 (0.116)	0.458 - 0.77
RG	500	49	0.10	0.20	0.106 (0.006)	0.754 (0.102)	0.006 (0.005)	0 - 0.017	0 (0)	-2175.71 (59.68)	0.554 (0.102)	0.334 - 0.67
RG	500	49	0.10	0.30	0.1 (0.002)	0.741 (0.127)	0.001 (0.001)	0 - 0.006	0 (0)	-2132.09 (31.38)	0.441 (0.127)	0.124 - 0.566
RG	500	49	0.10	0.40	0.1 (0.002)	0.738 (0.159)	0.001 (0.001)	0 - 0.005	0 (0)	-2123.51 (27.36)	0.34 (0.155)	0.01 - 0.475
RG	500	49	0.10	0.50	0.1 (0.002)	0.7 (0.163)	0.002 (0.001)	0 - 0.005	0 (0)	-2125.66 (33.34)	0.222 (0.132)	0.006 - 0.37
RG	500	49	0.10	0.60	0.1 (0.002)	0.68 (0.166)	0.002 (0.001)	0 - 0.007	0 (0)	-2126.53 (29.6)	0.152 (0.104)	0.003 - 0.264
RG	500	49	0.10	0.70	0.1 (0.002)	0.686 (0.169)	0.001 (0.001)	0 - 0.003	0 (0)	-2126.53 (28.53)	0.156 (0.065)	0.036 - 0.411
RG	500	49	0.10	0.80	0.1 (0.002)	0.716 (0.149)	0.001 (0.001)	0 - 0.005	0 (0)	-2135.97 (31.59)	0.139 (0.098)	0.031 - 0.42
RG	500	49	0.10	0.90	0.1 (0.002)	0.724 (0.154)	0.001 (0.001)	0 - 0.006	0 (0)	-2124.74 (30.66)	0.176 (0.154)	0.031 - 0.54
RG	500	49	0.20	0.10	0.212 (0.003)	0.748 (0.073)	0.012 (0.003)	0.001 - 0.02	0 (0)	-2465.57 (35.63)	0.648 (0.073)	0.455 - 0.719
RG	500	49	0.20	0.20	0.205 (0.003)	0.703 (0.106)	0.005 (0.003)	0 - 0.014	0 (0)	-2451.56 (33.52)	0.503 (0.106)	0.338 - 0.628
RG	500	49	0.20	0.30	0.202 (0.003)	0.681 (0.12)	0.003 (0.003)	0 - 0.01	0 (0)	-2444.29 (34.58)	0.381 (0.12)	0.224 - 0.529
RG	500	49	0.20	0.40	0.201 (0.003)	0.682 (0.133)	0.002 (0.002)	0 - 0.008	0 (0)	-2431.59 (33.82)	0.282 (0.133)	0.119 - 0.43
RG	500	49	0.20	0.50	0.2 (0.003)	0.677 (0.146)	0.002 (0.002)	0 - 0.007	0 (0)	-2428.33 (29.55)	0.18 (0.142)	0.003 - 0.327
RG	500	49	0.20	0.60	0.2 (0.003)	0.645 (0.169)	0.002 (0.002)	0 - 0.007	0 (0)	-2423.47 (30.44)	0.163 (0.061)	0.032 - 0.277
RG	500	49	0.20	0.70	0.2 (0.003)	0.628 (0.185)	0.002 (0.002)	0 - 0.008	0 (0)	-2422.49 (29.82)	0.18 (0.083)	0.094 - 0.451
RG	500	49	0.20	0.80	0.2 (0.002)	0.617 (0.194)	0.002 (0.001)	0 - 0.008	0 (0)	-2425.01 (32.82)	0.197 (0.179)	0.001 - 0.565
RG	500	49	0.20	0.90	0.2 (0.002)	0.584 (0.214)	0.002 (0.001)	0 - 0.005	0 (0)	-2430.25 (32.38)	0.316 (0.214)	0.073 - 0.731

Continued on next page

Table S1 – *Continued from previous page*

Structure	T	N	p	p_e/p_w	$\overline{\hat{p}}$ (SD)	$\overline{\hat{p_e}}/\overline{\hat{p_w}}$ (SD)	$\overline{\lvert\Delta\rvert}_p$ (SD)	Range $\lvert\Delta\rvert_p$	$\overline{SE}\lvert\Delta\rvert_p$ (SD)	$\overline{BIC}$ (SD)	$\overline{\lvert\Delta\rvert}_{p_e/p_w}$ (SD)	Range $\lvert\Delta\rvert_{p_e/p_w}$
RG	500	49	0.30	0.10	0.326 (0.005)	0.608 (0.076)	0.026 (0.005)	0.013 - 0.038	0 (0)	-2621.14 (36.02)	0.508 (0.076)	0.402 - 0.635
RG	500	49	0.30	0.20	0.318 (0.006)	0.558 (0.07)	0.018 (0.006)	0.007 - 0.03	0 (0)	-2612.39 (37.92)	0.358 (0.07)	0.294 - 0.509
RG	500	49	0.30	0.30	0.308 (0.004)	0.54 (0.053)	0.008 (0.004)	0 - 0.02	0 (0)	-2585.24 (32.51)	0.24 (0.053)	0.183 - 0.412
RG	500	49	0.30	0.40	0.304 (0.003)	0.546 (0.058)	0.004 (0.003)	0 - 0.014	0 (0)	-2588.32 (32.76)	0.146 (0.058)	0.095 - 0.316
RG	500	49	0.30	0.50	0.301 (0.003)	0.56 (0.068)	0.003 (0.002)	0 - 0.011	0 (0)	-2572.01 (37.61)	0.061 (0.068)	0 - 0.215
RG	500	49	0.30	0.60	0.299 (0.004)	0.561 (0.069)	0.003 (0.002)	0 - 0.009	0 (0)	-2572.72 (29.91)	0.076 (0.02)	0.027 - 0.125
RG	500	49	0.30	0.70	0.299 (0.004)	0.562 (0.067)	0.003 (0.002)	0 - 0.01	0 (0)	-2570.23 (31.4)	0.138 (0.065)	0.006 - 0.206
RG	500	49	0.30	0.80	0.297 (0.004)	0.568 (0.077)	0.004 (0.003)	0 - 0.013	0 (0)	-2586.82 (61.65)	0.232 (0.077)	0.072 - 0.357
RG	500	49	0.30	0.90	0.298 (0.004)	0.553 (0.08)	0.004 (0.003)	0 - 0.013	0 (0)	-2575.82 (49.93)	0.347 (0.08)	0.126 - 0.464
RG	500	49	0.40	0.10	0.426 (0.004)	0.588 (0.013)	0.026 (0.004)	0.012 - 0.038	0 (0)	-2653.36 (29.99)	0.488 (0.013)	0.457 - 0.543
RG	500	49	0.40	0.20	0.425 (0.004)	0.559 (0.018)	0.025 (0.004)	0.016 - 0.035	0 (0)	-2651.18 (32.53)	0.359 (0.018)	0.332 - 0.391
RG	500	49	0.40	0.30	0.42 (0.005)	0.562 (0.024)	0.02 (0.005)	0.002 - 0.026	0 (0)	-2657.06 (32.72)	0.262 (0.024)	0.214 - 0.292
RG	500	49	0.40	0.40	0.414 (0.006)	0.55 (0.03)	0.014 (0.006)	0.002 - 0.025	0 (0)	-2668.28 (39.7)	0.15 (0.03)	0.112 - 0.191
RG	500	49	0.40	0.50	0.411 (0.007)	0.561 (0.025)	0.012 (0.006)	0.001 - 0.023	0 (0)	-2683.22 (42.04)	0.061 (0.025)	0.004 - 0.091
RG	500	49	0.40	0.60	0.406 (0.009)	0.555 (0.03)	0.009 (0.005)	0 - 0.022	0 (0)	-2697.63 (52.76)	0.045 (0.03)	0.009 - 0.108
RG	500	49	0.40	0.70	0.403 (0.008)	0.558 (0.025)	0.008 (0.004)	0 - 0.021	0 (0)	-2702.41 (50.87)	0.142 (0.025)	0.109 - 0.205
RG	500	49	0.40	0.80	0.397 (0.007)	0.55 (0.026)	0.005 (0.005)	0 - 0.021	0 (0)	-2713.66 (57.03)	0.25 (0.026)	0.211 - 0.308
RG	500	49	0.40	0.90	0.396 (0.007)	0.557 (0.022)	0.006 (0.006)	0 - 0.021	0 (0)	-2756.18 (74.56)	0.343 (0.022)	0.313 - 0.404
RG	500	49	0.50	0.10	0.5 (0.006)	0.545 (0.152)	0.006 (0.003)	0 - 0.016	0 (0)	-2653.1 (30.64)	0.445 (0.149)	0.049 - 0.908
RG	500	49	0.50	0.20	0.501 (0.007)	0.517 (0.127)	0.006 (0.003)	0 - 0.014	0 (0)	-2652.87 (28.06)	0.324 (0.107)	0.133 - 0.802
RG	500	49	0.50	0.30	0.501 (0.007)	0.582 (0.214)	0.006 (0.003)	0.001 - 0.017	0 (0)	-2649.88 (32.21)	0.284 (0.211)	0.013 - 1.758

Continued on next page

Table S1 – *Continued from previous page*

Structure	T	N	p	p_e/p_w	$\overline{\hat{p}}$ (SD)	$\overline{\hat{p}_e}/\overline{\hat{p}_w}$ (SD)	$\overline{\lvert\Delta\rvert}_p$ (SD)	Range $\lvert\Delta\rvert_p$	$\overline{SE}\lvert\Delta\rvert_p$ (SD)	$\overline{BIC}$ (SD)	$\overline{\lvert\Delta\rvert}_{p_e/p_w}$ (SD)	Range $\lvert\Delta\rvert_{p_e/p_w}$
RG	500	49	0.50	0.40	0.5 (0.007)	0.558 (0.264)	0.006 (0.003)	0.001 - 0.017	0 (0)	-2651.09 (33.45)	0.192 (0.24)	0.003 - 2.157
RG	500	49	0.50	0.50	0.5 (0.007)	0.545 (0.132)	0.006 (0.003)	0.001 - 0.018	0 (0)	-2653.23 (30.91)	0.09 (0.106)	0 - 0.484
RG	500	49	0.50	0.60	0.501 (0.007)	0.557 (0.135)	0.006 (0.003)	0 - 0.015	0 (0)	-2653.77 (29.23)	0.112 (0.086)	0.001 - 0.452
RG	500	49	0.50	0.70	0.501 (0.006)	0.553 (0.106)	0.005 (0.003)	0 - 0.018	0 (0)	-2648.7 (28.92)	0.164 (0.075)	0.003 - 0.392
RG	500	49	0.50	0.80	0.5 (0.006)	0.558 (0.142)	0.006 (0.003)	0 - 0.015	0 (0)	-2655.62 (29.84)	0.262 (0.1)	0.024 - 0.489
RG	500	49	0.50	0.90	0.501 (0.006)	0.555 (0.158)	0.006 (0.003)	0.002 - 0.016	0 (0)	-2650.18 (31.3)	0.35 (0.147)	0.005 - 0.872
RG	500	49	0.60	0.10	0.557 (0.004)	0.631 (0.014)	0.043 (0.004)	0.031 - 0.053	0 (0)	-2652.1 (35.21)	0.531 (0.014)	0.496 - 0.564
RG	500	49	0.60	0.20	0.564 (0.006)	0.571 (0.01)	0.036 (0.006)	0.025 - 0.054	0 (0)	-2651.33 (28.2)	0.371 (0.01)	0.355 - 0.39
RG	500	49	0.60	0.30	0.577 (0.005)	0.546 (0.008)	0.023 (0.005)	0.011 - 0.034	0 (0)	-2649.66 (34.84)	0.246 (0.008)	0.235 - 0.266
RG	500	49	0.60	0.40	0.589 (0.005)	0.533 (0.008)	0.011 (0.005)	0.001 - 0.027	0 (0)	-2649.11 (33.15)	0.133 (0.008)	0.119 - 0.147
RG	500	49	0.60	0.50	0.599 (0.004)	0.523 (0.004)	0.003 (0.002)	0 - 0.011	0 (0)	-2645.57 (34.7)	0.023 (0.004)	0.014 - 0.039
RG	500	49	0.60	0.60	0.607 (0.004)	0.517 (0.008)	0.007 (0.004)	0 - 0.018	0 (0)	-2636.21 (26.29)	0.083 (0.008)	0.07 - 0.1
RG	500	49	0.60	0.70	0.611 (0.004)	0.516 (0.009)	0.011 (0.004)	0.001 - 0.02	0 (0)	-2648.67 (31.02)	0.184 (0.009)	0.171 - 0.205
RG	500	49	0.60	0.80	0.615 (0.003)	0.507 (0.009)	0.015 (0.003)	0.007 - 0.026	0 (0)	-2662.22 (33.45)	0.293 (0.009)	0.275 - 0.309
RG	500	49	0.60	0.90	0.618 (0.004)	0.504 (0.008)	0.018 (0.004)	0.007 - 0.026	0 (0)	-2684.83 (34.54)	0.396 (0.008)	0.375 - 0.409
RG	500	49	0.70	0.10	0.61 (0.006)	0.639 (0.009)	0.09 (0.006)	0.077 - 0.105	0 (0)	-2675.23 (36.6)	0.539 (0.009)	0.519 - 0.553
RG	500	49	0.70	0.20	0.64 (0.007)	0.575 (0.01)	0.06 (0.007)	0.043 - 0.075	0 (0)	-2675.52 (33.44)	0.375 (0.01)	0.354 - 0.392
RG	500	49	0.70	0.30	0.673 (0.005)	0.549 (0.01)	0.027 (0.005)	0.013 - 0.037	0 (0)	-2639.11 (34.57)	0.249 (0.01)	0.235 - 0.269
RG	500	49	0.70	0.40	0.688 (0.004)	0.495 (0.035)	0.012 (0.004)	0 - 0.023	0 (0)	-2683.78 (78.38)	0.095 (0.035)	0.05 - 0.144

Continued on next page

Table S1 – *Continued from previous page*

Structure	T	N	p	p_e/p_w	$\overline{\hat{p}}$ (SD)	$\overline{\hat{p}_e}/\overline{\hat{p}_w}$ (SD)	$\overline{\lvert\Delta\rvert}_p$ (SD)	Range $\lvert\Delta\rvert_p$	$\overline{SE}\lvert\Delta\rvert_p$ (SD)	$\overline{BIC}$ (SD)	$\overline{\lvert\Delta\rvert}_{p_e/p_w}$ (SD)	Range $\lvert\Delta\rvert_{p_e/p_w}$
RG	500	49	0.70	0.50	0.698 (0.005)	0.508 (0.027)	0.004 (0.003)	0 - 0.016	0 (0)	-2610.35 (84.62)	0.026 (0.01)	0.004 - 0.049
RG	500	49	0.70	0.60	0.703 (0.003)	0.508 (0.009)	0.003 (0.002)	0 - 0.009	0 (0)	-2575.24 (33.82)	0.092 (0.009)	0.076 - 0.106
RG	500	49	0.70	0.70	0.704 (0.003)	0.508 (0.009)	0.005 (0.003)	0 - 0.012	0 (0)	-2574.7 (31.59)	0.192 (0.009)	0.173 - 0.208
RG	500	49	0.70	0.80	0.705 (0.003)	0.5 (0.006)	0.005 (0.002)	0 - 0.01	0 (0)	-2584.78 (32.57)	0.3 (0.006)	0.283 - 0.324
RG	500	49	0.70	0.90	0.705 (0.003)	0.497 (0.01)	0.005 (0.003)	0 - 0.012	0 (0)	-2592.68 (37.16)	0.403 (0.01)	0.379 - 0.428
RG	500	49	0.80	0.10	0.685 (0.007)	0.652 (0.009)	0.115 (0.007)	0.101 - 0.134	0 (0)	-2811.59 (42.64)	0.552 (0.009)	0.537 - 0.576
RG	500	49	0.80	0.20	0.772 (0.006)	0.615 (0.013)	0.028 (0.006)	0.016 - 0.042	0 (0)	-2583.66 (50.12)	0.415 (0.013)	0.385 - 0.446
RG	500	49	0.80	0.30	0.794 (0.004)	0.566 (0.052)	0.006 (0.004)	0 - 0.016	0 (0)	-2461.43 (41.59)	0.266 (0.052)	0.143 - 0.325
RG	500	49	0.80	0.40	0.799 (0.003)	0.531 (0.047)	0.003 (0.002)	0 - 0.01	0 (0)	-2430.29 (32.92)	0.132 (0.046)	0.004 - 0.191
RG	500	49	0.80	0.50	0.8 (0.003)	0.511 (0.036)	0.002 (0.002)	0 - 0.007	0 (0)	-2431.73 (33.05)	0.032 (0.02)	0 - 0.085
RG	500	49	0.80	0.60	0.8 (0.002)	0.483 (0.037)	0.002 (0.001)	0 - 0.005	0 (0)	-2426.28 (30.61)	0.117 (0.037)	0.053 - 0.209
RG	500	49	0.80	0.70	0.801 (0.003)	0.481 (0.036)	0.002 (0.002)	0 - 0.008	0 (0)	-2428.6 (33.87)	0.219 (0.036)	0.157 - 0.308
RG	500	49	0.80	0.80	0.801 (0.002)	0.479 (0.034)	0.002 (0.002)	0 - 0.006	0 (0)	-2429.24 (31.66)	0.321 (0.034)	0.239 - 0.411
RG	500	49	0.80	0.90	0.801 (0.003)	0.479 (0.037)	0.002 (0.002)	0 - 0.007	0 (0)	-2428.26 (27.23)	0.421 (0.037)	0.334 - 0.509
RG	500	49	0.90	0.10	0.858 (0.008)	0.714 (0.06)	0.042 (0.008)	0.023 - 0.056	0 (0)	-2604.06 (121.7)	0.614 (0.06)	0.497 - 0.676
RG	500	49	0.90	0.20	0.895 (0.002)	0.505 (0.142)	0.005 (0.002)	0.001 - 0.01	0 (0)	-2175.09 (35.3)	0.305 (0.142)	0.126 - 0.536
RG	500	49	0.90	0.30	0.899 (0.002)	0.417 (0.097)	0.002 (0.001)	0 - 0.007	0 (0)	-2131.75 (30.92)	0.118 (0.096)	0.004 - 0.374
RG	500	49	0.90	0.40	0.9 (0.002)	0.414 (0.091)	0.001 (0.001)	0 - 0.004	0 (0)	-2131 (27.7)	0.067 (0.062)	0 - 0.273
RG	500	49	0.90	0.50	0.9 (0.002)	0.422 (0.077)	0.002 (0.001)	0 - 0.005	0 (0)	-2126.9 (30.29)	0.095 (0.054)	0.003 - 0.193

Continued on next page

Table S1 – *Continued from previous page*

Structure	T	N	p	p_e/p_w	$\overline{\hat{p}}$ (SD)	$\overline{\hat{p_e}}/\overline{\hat{p_w}}$ (SD)	$\overline{\lvert\Delta\rvert}_p$ (SD)	Range $\lvert\Delta\rvert_p$	$\overline{SE}\lvert\Delta\rvert_p$ (SD)	$\overline{BIC}$ (SD)	$\overline{\lvert\Delta\rvert}_{p_e/p_w}$ (SD)	Range $\lvert\Delta\rvert_{p_e/p_w}$
RG	500	49	0.90	0.60	0.9 (0.002)	0.413 (0.081)	0.002 (0.001)	0 - 0.006	0 (0)	-2126.55 (30.57)	0.192 (0.07)	0.001 - 0.304
RG	500	49	0.90	0.70	0.9 (0.002)	0.417 (0.084)	0.002 (0.001)	0 - 0.005	0 (0)	-2128.92 (30.55)	0.283 (0.084)	0.025 - 0.404
RG	500	49	0.90	0.80	0.9 (0.002)	0.445 (0.097)	0.001 (0.001)	0 - 0.006	0 (0)	-2132.43 (31.96)	0.355 (0.097)	0.129 - 0.532
RG	500	49	0.90	0.90	0.9 (0.002)	0.434 (0.082)	0.001 (0.001)	0 - 0.005	0 (0)	-2131.23 (31.74)	0.466 (0.082)	0.237 - 0.603
RG	500	100	0.10	0.10	0.103 (0.003)	0.773 (0.063)	0.003 (0.003)	0 - 0.009	0 (0)	-2504.83 (33.37)	0.673 (0.063)	0.534 - 0.781
RG	500	100	0.10	0.20	0.1 (0.001)	0.731 (0.086)	0.001 (0.001)	0 - 0.003	0 (0)	-2495.32 (33.38)	0.531 (0.086)	0.346 - 0.674
RG	500	100	0.10	0.30	0.1 (0.001)	0.742 (0.092)	0.001 (0.001)	0 - 0.003	0 (0)	-2490.67 (29.07)	0.442 (0.092)	0.25 - 0.575
RG	500	100	0.10	0.40	0.1 (0.001)	0.745 (0.083)	0.001 (0.001)	0 - 0.003	0 (0)	-2494.88 (30.19)	0.345 (0.083)	0.187 - 0.457
RG	500	100	0.10	0.50	0.1 (0.001)	0.744 (0.09)	0.001 (0.001)	0 - 0.005	0 (0)	-2492.85 (26.36)	0.244 (0.09)	0.06 - 0.406
RG	500	100	0.10	0.60	0.1 (0.002)	0.757 (0.083)	0.001 (0.001)	0 - 0.004	0 (0)	-2496.09 (31.5)	0.159 (0.079)	0.002 - 0.275
RG	500	100	0.10	0.70	0.1 (0.001)	0.761 (0.09)	0.001 (0.001)	0 - 0.004	0 (0)	-2496.85 (32.26)	0.097 (0.048)	0.008 - 0.181
RG	500	100	0.10	0.80	0.1 (0.001)	0.731 (0.091)	0.001 (0.001)	0 - 0.005	0 (0)	-2496.78 (28.09)	0.092 (0.067)	0.009 - 0.243
RG	500	100	0.10	0.90	0.1 (0.001)	0.744 (0.087)	0.001 (0.001)	0 - 0.003	0 (0)	-2493.26 (32.34)	0.156 (0.087)	0.025 - 0.336
RG	500	100	0.20	0.10	0.204 (0.002)	0.684 (0.113)	0.004 (0.002)	0 - 0.008	0 (0)	-2820.36 (43.63)	0.584 (0.113)	0.422 - 0.726
RG	500	100	0.20	0.20	0.202 (0.003)	0.72 (0.111)	0.003 (0.002)	0 - 0.007	0 (0)	-2793.72 (31.49)	0.52 (0.111)	0.159 - 0.629
RG	500	100	0.20	0.30	0.2 (0.002)	0.669 (0.121)	0.002 (0.001)	0 - 0.005	0 (0)	-2785.49 (29.16)	0.369 (0.121)	0.137 - 0.531
RG	500	100	0.20	0.40	0.2 (0.002)	0.689 (0.133)	0.001 (0.001)	0 - 0.004	0 (0)	-2790.31 (31.74)	0.289 (0.133)	0.03 - 0.431
RG	500	100	0.20	0.50	0.2 (0.002)	0.682 (0.135)	0.001 (0.001)	0 - 0.005	0 (0)	-2782.55 (31.02)	0.192 (0.12)	0.007 - 0.331
RG	500	100	0.20	0.60	0.2 (0.002)	0.689 (0.132)	0.001 (0.001)	0 - 0.004	0 (0)	-2780.5 (30.79)	0.128 (0.095)	0.001 - 0.231

Continued on next page

Table S1 – *Continued from previous page*

Structure	T	N	p	p_e/p_w	$\overline{\hat{p}}$ (SD)	$\overline{\hat{p_e}}/\overline{\hat{p_w}}$ (SD)	$\overline{\lvert\Delta\rvert}_p$ (SD)	Range $\lvert\Delta\rvert_p$	$\overline{SE}\lvert\Delta\rvert_p$ (SD)	$\overline{BIC}$ (SD)	$\overline{\lvert\Delta\rvert}_{p_e/p_w}$ (SD)	Range $\lvert\Delta\rvert_{p_e/p_w}$
RG	500	100	0.20	0.70	0.2 (0.002)	0.686 (0.14)	0.002 (0.001)	0 - 0.007	0 (0)	-2776.76 (32.8)	0.132 (0.046)	0.082 - 0.295
RG	500	100	0.20	0.80	0.2 (0.002)	0.686 (0.14)	0.002 (0.001)	0 - 0.006	0 (0)	-2785.89 (32.01)	0.131 (0.124)	0 - 0.379
RG	500	100	0.20	0.90	0.2 (0.002)	0.683 (0.133)	0.002 (0.001)	0 - 0.004	0 (0)	-2781.79 (31.53)	0.217 (0.133)	0.072 - 0.463
RG	500	100	0.30	0.10	0.316 (0.003)	0.592 (0.085)	0.016 (0.003)	0.01 - 0.025	0 (0)	-2977.83 (38.2)	0.492 (0.085)	0.361 - 0.608
RG	500	100	0.30	0.20	0.307 (0.002)	0.59 (0.084)	0.007 (0.002)	0 - 0.013	0 (0)	-2949.64 (32.09)	0.39 (0.084)	0.268 - 0.522
RG	500	100	0.30	0.30	0.303 (0.003)	0.608 (0.086)	0.003 (0.002)	0 - 0.011	0 (0)	-2935.12 (31.02)	0.308 (0.086)	0.194 - 0.43
RG	500	100	0.30	0.40	0.301 (0.002)	0.606 (0.09)	0.002 (0.001)	0 - 0.006	0 (0)	-2925.55 (31.93)	0.206 (0.09)	0.111 - 0.331
RG	500	100	0.30	0.50	0.3 (0.002)	0.609 (0.103)	0.002 (0.001)	0 - 0.005	0 (0)	-2917.3 (33.47)	0.113 (0.098)	0.001 - 0.231
RG	500	100	0.30	0.60	0.3 (0.002)	0.604 (0.113)	0.002 (0.001)	0 - 0.006	0 (0)	-2924.41 (31.74)	0.108 (0.03)	0.071 - 0.295
RG	500	100	0.30	0.70	0.3 (0.002)	0.599 (0.116)	0.002 (0.001)	0 - 0.006	0 (0)	-2923.92 (29.12)	0.113 (0.105)	0 - 0.311
RG	500	100	0.30	0.80	0.3 (0.002)	0.593 (0.122)	0.002 (0.001)	0 - 0.005	0 (0)	-2920 (31.32)	0.207 (0.122)	0.071 - 0.479
RG	500	100	0.30	0.90	0.3 (0.002)	0.587 (0.125)	0.002 (0.001)	0 - 0.006	0 (0)	-2921.87 (30.02)	0.313 (0.125)	0.169 - 0.566
RG	500	100	0.40	0.10	0.428 (0.004)	0.539 (0.009)	0.028 (0.004)	0.018 - 0.038	0 (0)	-3016.7 (35.24)	0.439 (0.009)	0.422 - 0.467
RG	500	100	0.40	0.20	0.42 (0.004)	0.522 (0.006)	0.02 (0.004)	0.011 - 0.03	0 (0)	-3013.73 (30.44)	0.322 (0.006)	0.31 - 0.336
RG	500	100	0.40	0.30	0.412 (0.003)	0.514 (0.004)	0.012 (0.003)	0.005 - 0.017	0 (0)	-3000.88 (27.61)	0.214 (0.004)	0.204 - 0.227
RG	500	100	0.40	0.40	0.406 (0.004)	0.518 (0.013)	0.006 (0.003)	0 - 0.015	0 (0)	-3003.29 (31.4)	0.118 (0.013)	0.103 - 0.17
RG	500	100	0.40	0.50	0.401 (0.004)	0.516 (0.013)	0.003 (0.003)	0 - 0.014	0 (0)	-2993.18 (33.48)	0.016 (0.013)	0.003 - 0.07
RG	500	100	0.40	0.60	0.397 (0.002)	0.509 (0.005)	0.003 (0.002)	0 - 0.009	0 (0)	-2994.47 (28.45)	0.091 (0.005)	0.074 - 0.099
RG	500	100	0.40	0.70	0.394 (0.003)	0.509 (0.006)	0.006 (0.003)	0 - 0.011	0 (0)	-3002.01 (30.7)	0.191 (0.006)	0.175 - 0.205

Continued on next page

Table S1 – *Continued from previous page*

Structure	T	N	p	p_e/p_w	$\overline{\hat{p}}$ (SD)	$\overline{\hat{p}_e}/\overline{\hat{p}_w}$ (SD)	$\overline{\|\Delta\|}_p$ (SD)	Range $\|\Delta\|_p$	$\overline{SE}\|\Delta\|_p$ (SD)	$\overline{BIC}$ (SD)	$\overline{\|\Delta\|}_{p_e/p_w}$ (SD)	Range $\|\Delta\|_{p_e/p_w}$
RG	500	100	0.40	0.80	0.393 (0.002)	0.508 (0.006)	0.007 (0.002)	0.002 - 0.013	0 (0)	-3010.48 (35.4)	0.292 (0.006)	0.274 - 0.308
RG	500	100	0.40	0.90	0.392 (0.002)	0.507 (0.007)	0.008 (0.002)	0.001 - 0.013	0 (0)	-3037.76 (33.81)	0.393 (0.007)	0.376 - 0.415
RG	500	100	0.50	0.10	0.501 (0.005)	0.548 (0.139)	0.004 (0.002)	0 - 0.011	0 (0)	-3001.71 (29.92)	0.45 (0.133)	0.074 - 1.321
RG	500	100	0.50	0.20	0.499 (0.004)	0.542 (0.151)	0.004 (0.002)	0 - 0.011	0 (0)	-3003.8 (28.38)	0.349 (0.133)	0.018 - 0.777
RG	500	100	0.50	0.30	0.5 (0.005)	0.55 (0.096)	0.004 (0.002)	0.001 - 0.01	0 (0)	-3012.59 (28.01)	0.253 (0.088)	0.135 - 0.55
RG	500	100	0.50	0.40	0.5 (0.005)	0.55 (0.115)	0.004 (0.002)	0 - 0.01	0 (0)	-3002.32 (32.05)	0.159 (0.102)	0.006 - 0.559
RG	500	100	0.50	0.50	0.499 (0.005)	0.556 (0.147)	0.004 (0.002)	0 - 0.012	0 (0)	-3007.97 (32.3)	0.091 (0.128)	0 - 0.731
RG	500	100	0.50	0.60	0.5 (0.004)	0.572 (0.107)	0.004 (0.002)	0 - 0.012	0 (0)	-3006.27 (31.17)	0.092 (0.061)	0 - 0.304
RG	500	100	0.50	0.70	0.5 (0.005)	0.536 (0.095)	0.004 (0.002)	0.001 - 0.011	0 (0)	-3006.59 (28.76)	0.177 (0.068)	0.007 - 0.429
RG	500	100	0.50	0.80	0.501 (0.005)	0.548 (0.128)	0.004 (0.002)	0.001 - 0.012	0 (0)	-3006.02 (28.74)	0.259 (0.112)	0.022 - 0.771
RG	500	100	0.50	0.90	0.5 (0.004)	0.555 (0.127)	0.004 (0.002)	0 - 0.009	0 (0)	-3009.19 (36.3)	0.354 (0.1)	0.032 - 0.756
RG	500	100	0.60	0.10	0.553 (0.003)	0.567 (0.005)	0.047 (0.003)	0.041 - 0.056	0 (0)	-3017.75 (29.25)	0.467 (0.005)	0.455 - 0.479
RG	500	100	0.60	0.20	0.567 (0.004)	0.533 (0.006)	0.033 (0.004)	0.026 - 0.042	0 (0)	-3019.2 (33.79)	0.333 (0.006)	0.321 - 0.35
RG	500	100	0.60	0.30	0.583 (0.003)	0.519 (0.005)	0.017 (0.003)	0.008 - 0.024	0 (0)	-3016.87 (31.18)	0.219 (0.005)	0.208 - 0.228
RG	500	100	0.60	0.40	0.592 (0.003)	0.516 (0.003)	0.008 (0.003)	0 - 0.018	0 (0)	-2992.94 (31.59)	0.116 (0.003)	0.103 - 0.128
RG	500	100	0.60	0.50	0.6 (0.003)	0.509 (0.006)	0.002 (0.002)	0 - 0.007	0 (0)	-2992.41 (35.74)	0.009 (0.006)	0 - 0.019
RG	500	100	0.60	0.60	0.604 (0.003)	0.506 (0.003)	0.004 (0.002)	0 - 0.01	0 (0)	-2999.46 (30.01)	0.094 (0.003)	0.082 - 0.099
RG	500	100	0.60	0.70	0.607 (0.002)	0.505 (0.003)	0.007 (0.002)	0.002 - 0.011	0 (0)	-3002.18 (33.55)	0.195 (0.003)	0.183 - 0.209

Continued on next page

Table S1 – *Continued from previous page*

Structure	T	N	p	p_e/p_w	$\bar{\hat{p}}$ (SD)	$\overline{\hat{p}_e}/\overline{\hat{p}_w}$ (SD)	$\overline{\lvert\Delta\rvert}_p$ (SD)	Range $\lvert\Delta\rvert_p$	$\overline{SE}\lvert\Delta\rvert_p$ (SD)	$\overline{BIC}$ (SD)	$\overline{\lvert\Delta\rvert}_{p_e/p_w}$ (SD)	Range $\lvert\Delta\rvert_{p_e/p_w}$
RG	500	100	0.60	0.80	0.609 (0.002)	0.504 (0.003)	0.009 (0.002)	0.001 - 0.015	0 (0)	-3023.55 (33.11)	0.296 (0.003)	0.291 - 0.307
RG	500	100	0.60	0.90	0.609 (0.002)	0.501 (0.005)	0.009 (0.002)	0.004 - 0.015	0 (0)	-3048.13 (37.51)	0.399 (0.005)	0.391 - 0.409
RG	500	100	0.70	0.10	0.624 (0.005)	0.575 (0.006)	0.076 (0.005)	0.063 - 0.086	0 (0)	-3135.72 (43.71)	0.475 (0.006)	0.462 - 0.49
RG	500	100	0.70	0.20	0.679 (0.004)	0.54 (0.023)	0.021 (0.004)	0.015 - 0.03	0 (0)	-3042.94 (51.83)	0.34 (0.023)	0.28 - 0.368
RG	500	100	0.70	0.30	0.696 (0.003)	0.532 (0.019)	0.004 (0.003)	0 - 0.011	0 (0)	-2939.23 (32.26)	0.232 (0.019)	0.194 - 0.258
RG	500	100	0.70	0.40	0.699 (0.002)	0.523 (0.017)	0.002 (0.001)	0 - 0.006	0 (0)	-2924.8 (31.19)	0.123 (0.017)	0.073 - 0.148
RG	500	100	0.70	0.50	0.7 (0.002)	0.507 (0.016)	0.002 (0.001)	0 - 0.005	0 (0)	-2922.89 (31.66)	0.014 (0.01)	0.001 - 0.055
RG	500	100	0.70	0.60	0.7 (0.002)	0.501 (0.013)	0.002 (0.001)	0 - 0.005	0 (0)	-2920.86 (31.38)	0.099 (0.013)	0.072 - 0.128
RG	500	100	0.70	0.70	0.701 (0.002)	0.499 (0.014)	0.002 (0.001)	0 - 0.007	0 (0)	-2923.36 (32.42)	0.201 (0.014)	0.166 - 0.246
RG	500	100	0.70	0.80	0.701 (0.002)	0.497 (0.014)	0.001 (0.001)	0 - 0.005	0 (0)	-2914.7 (30)	0.303 (0.014)	0.266 - 0.333
RG	500	100	0.70	0.90	0.701 (0.002)	0.494 (0.017)	0.002 (0.001)	0 - 0.006	0 (0)	-2921.31 (35.16)	0.406 (0.017)	0.364 - 0.447
RG	500	100	0.80	0.10	0.782 (0.003)	0.631 (0.079)	0.018 (0.003)	0.01 - 0.027	0 (0)	-2915.95 (47.35)	0.531 (0.079)	0.32 - 0.577
RG	500	100	0.80	0.20	0.798 (0.002)	0.459 (0.077)	0.002 (0.002)	0 - 0.007	0 (0)	-2795.66 (36.56)	0.259 (0.077)	0.173 - 0.439
RG	500	100	0.80	0.30	0.8 (0.002)	0.452 (0.058)	0.001 (0.001)	0 - 0.005	0 (0)	-2785.26 (30.25)	0.152 (0.058)	0.054 - 0.328
RG	500	100	0.80	0.40	0.8 (0.002)	0.463 (0.058)	0.001 (0.001)	0 - 0.004	0 (0)	-2783.88 (31.27)	0.068 (0.052)	0.001 - 0.219
RG	500	100	0.80	0.50	0.8 (0.002)	0.456 (0.057)	0.001 (0.001)	0 - 0.006	0 (0)	-2785.28 (27.43)	0.062 (0.035)	0.002 - 0.139
RG	500	100	0.80	0.60	0.8 (0.002)	0.457 (0.067)	0.001 (0.001)	0 - 0.005	0 (0)	-2779.04 (33.41)	0.144 (0.065)	0 - 0.247
RG	500	100	0.80	0.70	0.8 (0.002)	0.458 (0.065)	0.001 (0.001)	0 - 0.004	0 (0)	-2783.63 (33.08)	0.242 (0.065)	0.077 - 0.36

Continued on next page

Table S1 – *Continued from previous page*

Structure	T	N	p	p_e/p_w	$\overline{\hat{p}}$ (SD)	$\overline{\hat{p_e}}/\overline{\hat{p_w}}$ (SD)	$\overline{\lvert\Delta\rvert}_p$ (SD)	Range $\lvert\Delta\rvert_p$	$\overline{SE}\lvert\Delta\rvert_p$ (SD)	$\overline{BIC}$ (SD)	$\overline{\lvert\Delta\rvert}_{p_e/p_w}$ (SD)	Range $\lvert\Delta\rvert_{p_e/p_w}$
RG	500	100	0.80	0.80	0.8 (0.002)	0.463 (0.06)	0.002 (0.001)	0 - 0.006	0 (0)	-2784.99 (32.63)	0.337 (0.06)	0.188 - 0.426
RG	500	100	0.80	0.90	0.8 (0.002)	0.448 (0.051)	0.001 (0.001)	0 - 0.004	0 (0)	-2779.98 (31.39)	0.452 (0.051)	0.314 - 0.529
RG	500	100	0.90	0.10	0.897 (0.001)	0.406 (0.11)	0.003 (0.001)	0 - 0.007	0 (0)	-2515.99 (33.03)	0.306 (0.11)	0.161 - 0.516
RG	500	100	0.90	0.20	0.9 (0.001)	0.376 (0.095)	0.001 (0.001)	0 - 0.003	0 (0)	-2495.92 (31)	0.176 (0.095)	0.04 - 0.411
RG	500	100	0.90	0.30	0.9 (0.001)	0.381 (0.101)	0.001 (0.001)	0 - 0.004	0 (0)	-2495.79 (33.54)	0.106 (0.073)	0.006 - 0.3
RG	500	100	0.90	0.40	0.9 (0.001)	0.382 (0.104)	0.001 (0.001)	0 - 0.003	0 (0)	-2491.42 (33.24)	0.095 (0.045)	0.006 - 0.21
RG	500	100	0.90	0.50	0.9 (0.001)	0.398 (0.104)	0.001 (0.001)	0 - 0.004	0 (0)	-2492.03 (29.17)	0.112 (0.093)	0 - 0.259
RG	500	100	0.90	0.60	0.9 (0.001)	0.384 (0.094)	0.001 (0.001)	0 - 0.004	0 (0)	-2491.76 (30.66)	0.216 (0.094)	0.092 - 0.36
RG	500	100	0.90	0.70	0.9 (0.001)	0.383 (0.099)	0.001 (0.001)	0 - 0.003	0 (0)	-2491.82 (29.66)	0.317 (0.099)	0.118 - 0.46
RG	500	100	0.90	0.80	0.9 (0.001)	0.392 (0.097)	0.001 (0.001)	0 - 0.004	0 (0)	-2491.93 (31.4)	0.408 (0.097)	0.19 - 0.562
RG	500	100	0.90	0.90	0.9 (0.001)	0.364 (0.1)	0.001 (0.001)	0 - 0.004	0 (0)	-2489.4 (36.07)	0.536 (0.1)	0.295 - 0.676
RG	5000	16	0.10	0.10	0.132 (0.002)	0.786 (0.007)	0.032 (0.002)	0.028 - 0.035	0 (0)	-16845.38 (106.66)	0.686 (0.007)	0.674 - 0.696
RG	5000	16	0.10	0.20	0.104 (0.001)	0.811 (0.018)	0.004 (0.001)	0 - 0.007	0 (0)	-15652.23 (100.07)	0.611 (0.018)	0.545 - 0.631
RG	5000	16	0.10	0.30	0.102 (0.001)	0.804 (0.033)	0.002 (0.001)	0 - 0.005	0 (0)	-15506.99 (95.88)	0.504 (0.033)	0.229 - 0.531
RG	5000	16	0.10	0.40	0.101 (0.001)	0.802 (0.017)	0.001 (0.001)	0 - 0.005	0 (0)	-15474.84 (104.16)	0.402 (0.017)	0.361 - 0.432
RG	5000	16	0.10	0.50	0.101 (0.001)	0.806 (0.031)	0.001 (0.001)	0 - 0.005	0 (0)	-15573.06 (221.86)	0.306 (0.031)	0.257 - 0.494
RG	5000	16	0.10	0.60	0.1 (0.001)	0.801 (0.02)	0.001 (0.001)	0 - 0.003	0 (0)	-15618.98 (274.63)	0.201 (0.02)	0.175 - 0.265
RG	5000	16	0.10	0.70	0.099 (0.002)	0.816 (0.052)	0.001 (0.002)	0 - 0.017	0 (0)	-16386.71 (1864.69)	0.116 (0.051)	0.002 - 0.294
RG	5000	16	0.10	0.80	0.097 (0.005)	0.784 (0.063)	0.004 (0.005)	0 - 0.018	0 (0)	-17177.23 (1886.46)	0.049 (0.042)	0 - 0.138

Continued on next page

Table S1 – *Continued from previous page*

Structure	T	N	p	p_e/p_w	$\overline{\hat{p}}$ (SD)	$\overline{\hat{p}_e}/\overline{\hat{p}_w}$ (SD)	$\overline{\lvert\Delta\rvert}_p$ (SD)	Range $\lvert\Delta\rvert_p$	$\overline{SE}\lvert\Delta\rvert_p$ (SD)	$\overline{BIC}$ (SD)	$\overline{\lvert\Delta\rvert}_{p_e/p_w}$ (SD)	Range $\lvert\Delta\rvert_{p_e/p_w}$
RG	5000	16	0.10	0.90	0.098 (0.004)	0.808 (0.074)	0.003 (0.004)	0 - 0.016	0 (0)	-16609.77 (1906.87)	0.101 (0.06)	0 - 0.212
RG	5000	16	0.20	0.10	0.247 (0.005)	0.596 (0.065)	0.047 (0.005)	0.038 - 0.057	0 (0)	-19501.02 (118.6)	0.496 (0.065)	0.412 - 0.66
RG	5000	16	0.20	0.20	0.218 (0.002)	0.74 (0.008)	0.018 (0.002)	0.015 - 0.022	0 (0)	-19256.13 (107.86)	0.54 (0.008)	0.52 - 0.555
RG	5000	16	0.20	0.30	0.209 (0.002)	0.72 (0.039)	0.009 (0.002)	0.005 - 0.013	0 (0)	-18999.96 (123.75)	0.42 (0.039)	0.297 - 0.459
RG	5000	16	0.20	0.40	0.205 (0.002)	0.671 (0.034)	0.005 (0.002)	0 - 0.011	0 (0)	-18888.16 (104.49)	0.271 (0.034)	0.151 - 0.358
RG	5000	16	0.20	0.50	0.204 (0.002)	0.66 (0.026)	0.004 (0.002)	0 - 0.007	0 (0)	-18862.25 (95.2)	0.16 (0.026)	0.04 - 0.186
RG	5000	16	0.20	0.60	0.203 (0.002)	0.66 (0.02)	0.003 (0.002)	0 - 0.007	0 (0)	-18889.08 (114.88)	0.061 (0.017)	0.016 - 0.086
RG	5000	16	0.20	0.70	0.2 (0.002)	0.653 (0.033)	0.001 (0.001)	0 - 0.007	0 (0)	-18826.83 (134.88)	0.048 (0.031)	0 - 0.14
RG	5000	16	0.20	0.80	0.2 (0.003)	0.645 (0.036)	0.002 (0.001)	0 - 0.008	0 (0)	-19025.61 (217.52)	0.155 (0.036)	0.075 - 0.288
RG	5000	16	0.20	0.90	0.199 (0.002)	0.641 (0.039)	0.002 (0.002)	0 - 0.009	0 (0)	-18945.29 (203.28)	0.259 (0.039)	0.175 - 0.387
RG	5000	16	0.30	0.10	0.348 (0.002)	0.644 (0.008)	0.048 (0.002)	0.039 - 0.054	0 (0)	-20525.69 (93.56)	0.544 (0.008)	0.525 - 0.586
RG	5000	16	0.30	0.20	0.327 (0.002)	0.662 (0.005)	0.027 (0.002)	0.022 - 0.034	0 (0)	-20577.44 (100.47)	0.462 (0.005)	0.449 - 0.47
RG	5000	16	0.30	0.30	0.317 (0.002)	0.655 (0.006)	0.017 (0.002)	0.012 - 0.022	0 (0)	-20482.12 (109.2)	0.355 (0.006)	0.347 - 0.371
RG	5000	16	0.30	0.40	0.314 (0.002)	0.656 (0.007)	0.014 (0.002)	0.01 - 0.019	0 (0)	-20510.89 (103.9)	0.256 (0.007)	0.247 - 0.27
RG	5000	16	0.30	0.50	0.314 (0.002)	0.654 (0.005)	0.014 (0.002)	0.009 - 0.02	0 (0)	-20582.72 (110.3)	0.154 (0.005)	0.147 - 0.17
RG	5000	16	0.30	0.60	0.314 (0.002)	0.655 (0.007)	0.014 (0.002)	0.008 - 0.02	0 (0)	-20640.63 (115.63)	0.055 (0.007)	0.048 - 0.071
RG	5000	16	0.30	0.70	0.307 (0.002)	0.662 (0.007)	0.007 (0.002)	0.002 - 0.012	0 (0)	-20739.83 (132.14)	0.038 (0.007)	0.029 - 0.052
RG	5000	16	0.30	0.80	0.31 (0.002)	0.664 (0.007)	0.01 (0.002)	0.003 - 0.017	0 (0)	-21416.72 (227.39)	0.136 (0.007)	0.128 - 0.152
RG	5000	16	0.30	0.90	0.306 (0.002)	0.66 (0.007)	0.006 (0.002)	0.002 - 0.012	0 (0)	-21224.39 (224.89)	0.24 (0.007)	0.228 - 0.252
RG	5000	16	0.40	0.10	0.422 (0.002)	0.588 (0.006)	0.022 (0.002)	0.016 - 0.029	0 (0)	-21046.65 (103.58)	0.488 (0.006)	0.478 - 0.496

Continued on next page

Table S1 – *Continued from previous page*

Structure	T	N	p	p_e/p_w	$\overline{\hat{p}}$ (SD)	$\overline{\hat{p}_e}/\overline{\hat{p}_w}$ (SD)	$\overline{\lvert\Delta\rvert}_p$ (SD)	Range $\lvert\Delta\rvert_p$	$\overline{SE}\lvert\Delta\rvert_p$ (SD)	$\overline{BIC}$ (SD)	$\overline{\lvert\Delta\rvert}_{p_e/p_w}$ (SD)	Range $\lvert\Delta\rvert_{p_e/p_w}$
RG	5000	16	0.40	0.20	0.414 (0.003)	0.605 (0.038)	0.014 (0.003)	0.007 - 0.019	0 (0)	-21012.43 (88.31)	0.405 (0.038)	0.374 - 0.48
RG	5000	16	0.40	0.30	0.41 (0.005)	0.628 (0.028)	0.01 (0.005)	0.001 - 0.019	0 (0)	-21005.54 (94.88)	0.328 (0.028)	0.276 - 0.387
RG	5000	16	0.40	0.40	0.405 (0.002)	0.604 (0.007)	0.005 (0.002)	0 - 0.011	0 (0)	-20973.72 (94.75)	0.204 (0.007)	0.178 - 0.217
RG	5000	16	0.40	0.50	0.406 (0.002)	0.604 (0.005)	0.006 (0.002)	0.001 - 0.012	0 (0)	-20964.17 (101.86)	0.104 (0.005)	0.089 - 0.114
RG	5000	16	0.40	0.60	0.406 (0.002)	0.602 (0.008)	0.006 (0.002)	0.001 - 0.012	0 (0)	-20991.17 (95.41)	0.006 (0.005)	0 - 0.023
RG	5000	16	0.40	0.70	0.399 (0.002)	0.593 (0.018)	0.002 (0.002)	0 - 0.011	0 (0)	-21049.15 (97.51)	0.107 (0.018)	0.082 - 0.18
RG	5000	16	0.40	0.80	0.396 (0.007)	0.574 (0.031)	0.005 (0.006)	0 - 0.016	0 (0)	-21146.27 (147.97)	0.226 (0.031)	0.183 - 0.299
RG	5000	16	0.40	0.90	0.397 (0.002)	0.592 (0.013)	0.003 (0.002)	0 - 0.012	0 (0)	-21185.75 (111.87)	0.308 (0.013)	0.282 - 0.361
RG	5000	16	0.50	0.10	0.5 (0.003)	0.544 (0.12)	0.003 (0.002)	0 - 0.007	0 (0)	-21093.95 (93.49)	0.444 (0.12)	0.081 - 1.005
RG	5000	16	0.50	0.20	0.5 (0.004)	0.555 (0.144)	0.003 (0.002)	0 - 0.007	0 (0)	-21070.04 (91.55)	0.355 (0.144)	0.076 - 0.861
RG	5000	16	0.50	0.30	0.5 (0.003)	0.577 (0.158)	0.003 (0.002)	0 - 0.008	0 (0)	-21099.47 (81.16)	0.278 (0.157)	0.001 - 0.834
RG	5000	16	0.50	0.40	0.5 (0.004)	0.551 (0.122)	0.003 (0.002)	0 - 0.01	0 (0)	-21100.33 (87.12)	0.161 (0.108)	0.015 - 0.599
RG	5000	16	0.50	0.50	0.501 (0.003)	0.545 (0.101)	0.003 (0.002)	0 - 0.007	0 (0)	-21101.82 (101.99)	0.078 (0.078)	0 - 0.438
RG	5000	16	0.50	0.60	0.5 (0.004)	0.54 (0.086)	0.003 (0.002)	0 - 0.008	0 (0)	-21085.51 (88.65)	0.082 (0.064)	0 - 0.345
RG	5000	16	0.50	0.70	0.5 (0.004)	0.55 (0.117)	0.003 (0.002)	0 - 0.01	0 (0)	-21107.17 (108.7)	0.172 (0.08)	0.016 - 0.38
RG	5000	16	0.50	0.80	0.5 (0.003)	0.586 (0.141)	0.003 (0.002)	0 - 0.008	0 (0)	-21088.22 (104.22)	0.239 (0.092)	0.005 - 0.464
RG	5000	16	0.50	0.90	0.5 (0.003)	0.559 (0.105)	0.003 (0.002)	0 - 0.007	0 (0)	-21090.82 (104.04)	0.345 (0.094)	0.031 - 0.594
RG	5000	16	0.60	0.10	0.564 (0.003)	0.631 (0.064)	0.036 (0.003)	0.029 - 0.042	0 (0)	-21326.78 (249.32)	0.531 (0.064)	0.471 - 0.723
RG	5000	16	0.60	0.20	0.574 (0.003)	0.657 (0.019)	0.026 (0.003)	0.016 - 0.033	0 (0)	-21035.82 (97.74)	0.457 (0.019)	0.372 - 0.487

Continued on next page

Table S1 – *Continued from previous page*

Structure	T	N	p	p_e/p_w	$\overline{\hat{p}}$ (SD)	$\overline{\hat{p}_e}/\overline{\hat{p}_w}$ (SD)	$\overline{\lvert\Delta\rvert}_p$ (SD)	Range $\lvert\Delta\rvert_p$	$\overline{SE}\lvert\Delta\rvert_p$ (SD)	$\overline{BIC}$ (SD)	$\overline{\lvert\Delta\rvert}_{p_e/p_w}$ (SD)	Range $\lvert\Delta\rvert_{p_e/p_w}$
RG	5000	16	0.60	0.30	0.582 (0.005)	0.606 (0.034)	0.018 (0.005)	0.01 - 0.029	0 (0)	-21149.91 (177.12)	0.306 (0.034)	0.225 - 0.385
RG	5000	16	0.60	0.40	0.589 (0.002)	0.579 (0.04)	0.011 (0.002)	0.005 - 0.015	0 (0)	-21161.81 (152.58)	0.179 (0.04)	0.11 - 0.224
RG	5000	16	0.60	0.50	0.593 (0.002)	0.578 (0.037)	0.007 (0.002)	0.001 - 0.012	0 (0)	-21031.19 (115.99)	0.078 (0.037)	0.009 - 0.123
RG	5000	16	0.60	0.60	0.594 (0.003)	0.595 (0.025)	0.006 (0.003)	0 - 0.011	0 (0)	-21054.07 (101.89)	0.015 (0.02)	0 - 0.1
RG	5000	16	0.60	0.70	0.617 (0.002)	0.54 (0.01)	0.017 (0.002)	0.011 - 0.023	0 (0)	-20998.27 (90.63)	0.16 (0.01)	0.143 - 0.18
RG	5000	16	0.60	0.80	0.62 (0.003)	0.535 (0.012)	0.02 (0.003)	0.015 - 0.028	0 (0)	-21029.02 (99.19)	0.265 (0.012)	0.243 - 0.284
RG	5000	16	0.60	0.90	0.625 (0.002)	0.537 (0.011)	0.025 (0.002)	0.018 - 0.031	0 (0)	-21037.12 (101.71)	0.363 (0.011)	0.338 - 0.388
RG	5000	16	0.70	0.10	0.483 (0.018)	0.51 (0.087)	0.217 (0.018)	0.207 - 0.304	0 (0)	-24071.17 (684.79)	0.41 (0.083)	0.005 - 0.448
RG	5000	16	0.70	0.20	0.602 (0.022)	0.566 (0.075)	0.098 (0.022)	0.051 - 0.114	0 (0)	-22603.92 (980.76)	0.366 (0.075)	0.31 - 0.549
RG	5000	16	0.70	0.30	0.656 (0.006)	0.66 (0.023)	0.044 (0.006)	0.035 - 0.1	0 (0)	-20899.65 (267.16)	0.36 (0.023)	0.2 - 0.399
RG	5000	16	0.70	0.40	0.679 (0.003)	0.599 (0.012)	0.021 (0.003)	0.015 - 0.029	0 (0)	-20821.91 (106)	0.199 (0.012)	0.173 - 0.224
RG	5000	16	0.70	0.50	0.688 (0.001)	0.571 (0.041)	0.012 (0.001)	0.006 - 0.016	0 (0)	-20840.95 (228.2)	0.071 (0.041)	0.015 - 0.125
RG	5000	16	0.70	0.60	0.691 (0.003)	0.529 (0.025)	0.009 (0.003)	0 - 0.015	0 (0)	-21104.88 (153.22)	0.073 (0.019)	0.009 - 0.091
RG	5000	16	0.70	0.70	0.719 (0.002)	0.549 (0.005)	0.019 (0.002)	0.013 - 0.022	0 (0)	-20552.23 (93.45)	0.151 (0.005)	0.141 - 0.158
RG	5000	16	0.70	0.80	0.722 (0.002)	0.514 (0.006)	0.022 (0.002)	0.018 - 0.027	0 (0)	-20598.86 (99.11)	0.286 (0.006)	0.271 - 0.293
RG	5000	16	0.70	0.90	0.725 (0.002)	0.517 (0.008)	0.025 (0.002)	0.021 - 0.03	0 (0)	-20710.34 (102.21)	0.383 (0.008)	0.371 - 0.395
RG	5000	16	0.80	0.10	0.361 (0.003)	0.484 (0.006)	0.439 (0.003)	0.431 - 0.447	0 (0)	-23542.43 (195.88)	0.384 (0.006)	0.374 - 0.398
RG	5000	16	0.80	0.20	0.693 (0.024)	0.669 (0.066)	0.107 (0.024)	0.084 - 0.179	0 (0)	-21273.91 (1668.85)	0.469 (0.066)	0.311 - 0.555
RG	5000	16	0.80	0.30	0.729 (0.016)	0.637 (0.045)	0.071 (0.016)	0.058 - 0.128	0 (0)	-21443.84 (1379.48)	0.337 (0.045)	0.212 - 0.384

Continued on next page

Table S1 – *Continued from previous page*

Structure	T	N	p	p_e/p_w	$\overline{\hat{p}}$ (SD)	$\overline{\hat{p_e}}/\overline{\hat{p_w}}$ (SD)	$\overline{\lvert\Delta\rvert}_p$ (SD)	Range $\lvert\Delta\rvert_p$	$\overline{SE}\lvert\Delta\rvert_p$ (SD)	$\overline{BIC}$ (SD)	$\overline{\lvert\Delta\rvert}_{p_e/p_w}$ (SD)	Range $\lvert\Delta\rvert_{p_e/p_w}$
RG	5000	16	0.80	0.40	0.774 (0.002)	0.613 (0.005)	0.026 (0.002)	0.021 - 0.033	0 (0)	-20088.23 (110.62)	0.213 (0.005)	0.2 - 0.222
RG	5000	16	0.80	0.50	0.793 (0.002)	0.587 (0.015)	0.007 (0.002)	0 - 0.012	0 (0)	-19390.94 (98.61)	0.087 (0.015)	0.071 - 0.124
RG	5000	16	0.80	0.60	0.798 (0.002)	0.583 (0.004)	0.003 (0.002)	0 - 0.006	0 (0)	-19550.55 (115.73)	0.017 (0.004)	0.004 - 0.027
RG	5000	16	0.80	0.70	0.809 (0.001)	0.542 (0.013)	0.009 (0.001)	0.006 - 0.012	0 (0)	-19043.07 (105.26)	0.158 (0.013)	0.14 - 0.185
RG	5000	16	0.80	0.80	0.808 (0.001)	0.471 (0.011)	0.008 (0.001)	0.005 - 0.012	0 (0)	-19289.4 (119.65)	0.329 (0.011)	0.269 - 0.356
RG	5000	16	0.80	0.90	0.809 (0.001)	0.471 (0.008)	0.009 (0.001)	0.006 - 0.012	0 (0)	-19305.95 (126.24)	0.429 (0.008)	0.417 - 0.448
RG	5000	16	0.90	0.10	0.321 (0.001)	0.04 (0.03)	0.579 (0.001)	0.576 - 0.581	0 (0)	-18724.49 (85.04)	0.06 (0.03)	0.013 - 0.1
RG	5000	16	0.90	0.20	0.775 (0.02)	0.67 (0.072)	0.125 (0.02)	0.104 - 0.179	0 (0)	-21382.03 (2448.75)	0.47 (0.072)	0.312 - 0.551
RG	5000	16	0.90	0.30	0.834 (0.011)	0.668 (0.048)	0.066 (0.011)	0.047 - 0.083	0 (0)	-20296.07 (1235.75)	0.368 (0.048)	0.304 - 0.435
RG	5000	16	0.90	0.40	0.886 (0.002)	0.582 (0.025)	0.014 (0.002)	0.009 - 0.021	0 (0)	-16818.96 (241.55)	0.182 (0.025)	0.071 - 0.268
RG	5000	16	0.90	0.50	0.892 (0.001)	0.474 (0.009)	0.008 (0.001)	0.005 - 0.011	0 (0)	-16420.22 (147.59)	0.027 (0.007)	0.008 - 0.041
RG	5000	16	0.90	0.60	0.894 (0.001)	0.473 (0.005)	0.006 (0.001)	0.004 - 0.01	0 (0)	-16421.6 (156.08)	0.127 (0.005)	0.119 - 0.148
RG	5000	16	0.90	0.70	0.901 (0.001)	0.457 (0.045)	0.001 (0.001)	0 - 0.003	0 (0)	-15552.35 (108.77)	0.243 (0.045)	0.14 - 0.305
RG	5000	16	0.90	0.80	0.902 (0.001)	0.431 (0.016)	0.002 (0.001)	0 - 0.004	0 (0)	-15511.3 (132.1)	0.369 (0.016)	0.35 - 0.405
RG	5000	16	0.90	0.90	0.902 (0.001)	0.43 (0.017)	0.002 (0.001)	0 - 0.004	0 (0)	-15527.31 (145.84)	0.47 (0.017)	0.444 - 0.505
RG	5000	25	0.10	0.10	0.104 (0.001)	0.832 (0.01)	0.004 (0.001)	0.002 - 0.006	0 (0)	-18167.87 (101.54)	0.732 (0.01)	0.692 - 0.753
RG	5000	25	0.10	0.20	0.101 (0.001)	0.795 (0.083)	0.001 (0.001)	0 - 0.003	0 (0)	-17949.26 (93.02)	0.595 (0.083)	0.394 - 0.661
RG	5000	25	0.10	0.30	0.101 (0.001)	0.761 (0.096)	0.001 (0.001)	0 - 0.003	0 (0)	-17937.85 (106.3)	0.461 (0.096)	0.246 - 0.57
RG	5000	25	0.10	0.40	0.1 (0.001)	0.73 (0.121)	0.001 (0.001)	0 - 0.004	0 (0)	-17991.83 (166.94)	0.33 (0.121)	0.17 - 0.47

Continued on next page

Table S1 – *Continued from previous page*

Structure	T	N	p	p_e/p_w	$\overline{\hat{p}}$ (SD)	$\overline{\hat{p}_e}/\overline{\hat{p}_w}$ (SD)	$\overline{\lvert\Delta\rvert}_p$ (SD)	Range $\lvert\Delta\rvert_p$	$\overline{SE}\lvert\Delta\rvert_p$ (SD)	$\overline{BIC}$ (SD)	$\overline{\lvert\Delta\rvert}_{p_e/p_w}$ (SD)	Range $\lvert\Delta\rvert_{p_e/p_w}$
RG	5000	25	0.10	0.50	0.1 (0.001)	0.734 (0.118)	0.001 (0.001)	0 - 0.003	0 (0)	-17985.48 (166.4)	0.234 (0.118)	0.044 - 0.368
RG	5000	25	0.10	0.60	0.1 (0.001)	0.714 (0.155)	0.001 (0.001)	0 - 0.006	0 (0)	-17917.87 (113.87)	0.161 (0.105)	0.002 - 0.274
RG	5000	25	0.10	0.70	0.099 (0.002)	0.704 (0.163)	0.001 (0.001)	0 - 0.006	0 (0)	-17928.31 (138.49)	0.158 (0.036)	0.078 - 0.295
RG	5000	25	0.10	0.80	0.099 (0.002)	0.7 (0.166)	0.001 (0.001)	0 - 0.006	0 (0)	-17934.54 (145.41)	0.158 (0.11)	0.046 - 0.384
RG	5000	25	0.10	0.90	0.1 (0.001)	0.684 (0.187)	0.001 (0.001)	0 - 0.005	0 (0)	-17905.86 (132.92)	0.216 (0.187)	0.03 - 0.615
RG	5000	25	0.20	0.10	0.218 (0.002)	0.788 (0.009)	0.018 (0.002)	0.014 - 0.022	0 (0)	-21595.49 (106.33)	0.688 (0.009)	0.659 - 0.711
RG	5000	25	0.20	0.20	0.209 (0.001)	0.763 (0.055)	0.009 (0.001)	0.005 - 0.011	0 (0)	-21285.55 (118.4)	0.563 (0.055)	0.326 - 0.611
RG	5000	25	0.20	0.30	0.207 (0.001)	0.77 (0.064)	0.007 (0.001)	0.001 - 0.01	0 (0)	-21260.03 (120.28)	0.47 (0.064)	0.207 - 0.534
RG	5000	25	0.20	0.40	0.204 (0.002)	0.719 (0.092)	0.004 (0.002)	0 - 0.007	0 (0)	-21192.88 (121.25)	0.319 (0.092)	0.102 - 0.412
RG	5000	25	0.20	0.50	0.203 (0.002)	0.712 (0.103)	0.003 (0.002)	0 - 0.008	0 (0)	-21156.02 (120.12)	0.212 (0.103)	0.004 - 0.324
RG	5000	25	0.20	0.60	0.201 (0.001)	0.683 (0.104)	0.001 (0.001)	0 - 0.005	0 (0)	-21122.02 (148.12)	0.106 (0.08)	0 - 0.225
RG	5000	25	0.20	0.70	0.2 (0.002)	0.69 (0.105)	0.001 (0.001)	0 - 0.004	0 (0)	-21115.01 (143.73)	0.102 (0.025)	0.026 - 0.169
RG	5000	25	0.20	0.80	0.199 (0.002)	0.676 (0.106)	0.001 (0.001)	0 - 0.006	0 (0)	-21201.83 (192.86)	0.13 (0.098)	0.001 - 0.305
RG	5000	25	0.20	0.90	0.199 (0.002)	0.644 (0.102)	0.002 (0.002)	0 - 0.011	0 (0)	-21244.13 (337.84)	0.256 (0.102)	0.073 - 0.401
RG	5000	25	0.30	0.10	0.325 (0.003)	0.603 (0.023)	0.025 (0.003)	0.021 - 0.031	0 (0)	-22813.72 (107.98)	0.503 (0.023)	0.471 - 0.54
RG	5000	25	0.30	0.20	0.317 (0.002)	0.601 (0.021)	0.017 (0.002)	0.008 - 0.023	0 (0)	-22721.03 (104.15)	0.401 (0.021)	0.35 - 0.466
RG	5000	25	0.30	0.30	0.311 (0.004)	0.56 (0.028)	0.011 (0.004)	0.005 - 0.021	0 (0)	-22672.56 (114.63)	0.26 (0.028)	0.233 - 0.362
RG	5000	25	0.30	0.40	0.31 (0.007)	0.575 (0.045)	0.01 (0.007)	0.002 - 0.021	0 (0)	-22752.67 (269.52)	0.175 (0.045)	0.129 - 0.263
RG	5000	25	0.30	0.50	0.308 (0.007)	0.567 (0.043)	0.008 (0.007)	0.001 - 0.021	0 (0)	-22746.13 (254.12)	0.067 (0.043)	0.03 - 0.162

Continued on next page

Table S1 – *Continued from previous page*

Structure	T	N	p	p_e/p_w	$\overline{\hat{p}}$ (SD)	$\overline{\hat{p_e}}/\overline{\hat{p_w}}$ (SD)	$\overline{\lvert\Delta\rvert}_p$ (SD)	Range $\lvert\Delta\rvert_p$	$\overline{SE}\lvert\Delta\rvert_p$ (SD)	$\overline{BIC}$ (SD)	$\overline{\lvert\Delta\rvert}_{p_e/p_w}$ (SD)	Range $\lvert\Delta\rvert_{p_e/p_w}$
RG	5000	25	0.30	0.60	0.306 (0.002)	0.584 (0.008)	0.006 (0.002)	0.001 - 0.009	0 (0)	-22860.04 (148.37)	0.016 (0.008)	0.003 - 0.058
RG	5000	25	0.30	0.70	0.3 (0.002)	0.587 (0.02)	0.002 (0.002)	0 - 0.008	0 (0)	-22678.61 (143.31)	0.113 (0.02)	0.026 - 0.172
RG	5000	25	0.30	0.80	0.298 (0.002)	0.592 (0.006)	0.002 (0.001)	0 - 0.009	0 (0)	-23095.37 (196.53)	0.208 (0.006)	0.199 - 0.239
RG	5000	25	0.30	0.90	0.296 (0.003)	0.584 (0.012)	0.004 (0.003)	0 - 0.01	0 (0)	-23096.63 (277.05)	0.316 (0.012)	0.3 - 0.343
RG	5000	25	0.40	0.10	0.417 (0.003)	0.592 (0.032)	0.017 (0.003)	0.012 - 0.025	0 (0)	-23289.9 (89.09)	0.492 (0.032)	0.472 - 0.58
RG	5000	25	0.40	0.20	0.413 (0.002)	0.581 (0.007)	0.013 (0.002)	0.008 - 0.017	0 (0)	-23249.9 (108.85)	0.381 (0.007)	0.366 - 0.396
RG	5000	25	0.40	0.30	0.416 (0.002)	0.579 (0.006)	0.016 (0.002)	0.012 - 0.02	0 (0)	-23222.21 (97.64)	0.279 (0.006)	0.272 - 0.295
RG	5000	25	0.40	0.40	0.415 (0.002)	0.579 (0.006)	0.015 (0.002)	0.01 - 0.021	0 (0)	-23294.95 (90.43)	0.179 (0.006)	0.172 - 0.195
RG	5000	25	0.40	0.50	0.414 (0.003)	0.58 (0.008)	0.014 (0.003)	0.003 - 0.02	0 (0)	-23278.89 (98.04)	0.08 (0.008)	0.059 - 0.095
RG	5000	25	0.40	0.60	0.411 (0.002)	0.588 (0.007)	0.011 (0.002)	0.007 - 0.016	0 (0)	-23488.27 (114.31)	0.012 (0.007)	0.004 - 0.027
RG	5000	25	0.40	0.70	0.403 (0.002)	0.58 (0.006)	0.003 (0.002)	0 - 0.011	0 (0)	-23439.21 (109.66)	0.12 (0.006)	0.108 - 0.128
RG	5000	25	0.40	0.80	0.404 (0.002)	0.581 (0.007)	0.004 (0.002)	0 - 0.01	0 (0)	-23748.61 (112.02)	0.219 (0.007)	0.204 - 0.228
RG	5000	25	0.40	0.90	0.4 (0.002)	0.58 (0.004)	0.001 (0.001)	0 - 0.006	0 (0)	-23875.3 (122.47)	0.32 (0.004)	0.308 - 0.327
RG	5000	25	0.50	0.10	0.5 (0.003)	0.611 (0.233)	0.002 (0.001)	0 - 0.005	0 (0)	-23333.94 (103.27)	0.511 (0.233)	0.125 - 1.772
RG	5000	25	0.50	0.20	0.5 (0.003)	0.577 (0.159)	0.002 (0.001)	0 - 0.006	0 (0)	-23346.41 (87.54)	0.377 (0.159)	0.139 - 1.236
RG	5000	25	0.50	0.30	0.5 (0.003)	0.59 (0.195)	0.002 (0.001)	0 - 0.006	0 (0)	-23325.24 (104.29)	0.296 (0.185)	0.024 - 1.126
RG	5000	25	0.50	0.40	0.5 (0.003)	0.571 (0.149)	0.003 (0.002)	0 - 0.007	0 (0)	-23319.72 (92.01)	0.175 (0.145)	0.007 - 0.742
RG	5000	25	0.50	0.50	0.5 (0.003)	0.573 (0.163)	0.002 (0.001)	0 - 0.006	0 (0)	-23324.83 (96.23)	0.118 (0.134)	0 - 0.947
RG	5000	25	0.50	0.60	0.5 (0.003)	0.582 (0.157)	0.003 (0.001)	0 - 0.008	0 (0)	-23319.38 (110.12)	0.121 (0.101)	0.007 - 0.52
RG	5000	25	0.50	0.70	0.501 (0.003)	0.59 (0.187)	0.002 (0.001)	0 - 0.008	0 (0)	-23309.65 (104.52)	0.187 (0.108)	0.007 - 0.626

Continued on next page

Table S1 – *Continued from previous page*

Structure	T	N	p	p_e/p_w	$\overline{\hat{p}}$ (SD)	$\overline{\hat{p_e}}/\overline{\hat{p_w}}$ (SD)	$\overline{\lvert\Delta\rvert}_p$ (SD)	Range $\lvert\Delta\rvert_p$	$\overline{SE}\lvert\Delta\rvert_p$ (SD)	$\overline{BIC}$ (SD)	$\overline{\lvert\Delta\rvert}_{p_e/p_w}$ (SD)	Range $\lvert\Delta\rvert_{p_e/p_w}$
RG	5000	25	0.50	0.80	0.5 (0.003)	0.597 (0.206)	0.002 (0.001)	0 - 0.007	0 (0)	-23333.85 (104.36)	0.258 (0.129)	0.012 - 0.791
RG	5000	25	0.50	0.90	0.5 (0.003)	0.581 (0.183)	0.003 (0.001)	0 - 0.007	0 (0)	-23323.75 (106.42)	0.347 (0.122)	0.057 - 0.78
RG	5000	25	0.60	0.10	0.564 (0.002)	0.708 (0.011)	0.036 (0.002)	0.032 - 0.039	0 (0)	-23281.95 (92.97)	0.608 (0.011)	0.56 - 0.647
RG	5000	25	0.60	0.20	0.573 (0.002)	0.627 (0.007)	0.027 (0.002)	0.022 - 0.032	0 (0)	-23292.28 (94.49)	0.427 (0.007)	0.412 - 0.443
RG	5000	25	0.60	0.30	0.578 (0.002)	0.585 (0.005)	0.022 (0.002)	0.017 - 0.028	0 (0)	-23241.36 (100.04)	0.285 (0.005)	0.274 - 0.293
RG	5000	25	0.60	0.40	0.591 (0.002)	0.54 (0.008)	0.009 (0.002)	0.004 - 0.014	0 (0)	-23228.31 (97.35)	0.14 (0.008)	0.126 - 0.154
RG	5000	25	0.60	0.50	0.591 (0.002)	0.54 (0.007)	0.009 (0.002)	0.004 - 0.015	0 (0)	-23278.43 (96.88)	0.04 (0.007)	0.026 - 0.057
RG	5000	25	0.60	0.60	0.603 (0.002)	0.538 (0.005)	0.003 (0.002)	0 - 0.007	0 (0)	-23276.48 (95.77)	0.062 (0.005)	0.049 - 0.088
RG	5000	25	0.60	0.70	0.612 (0.002)	0.547 (0.007)	0.012 (0.002)	0.008 - 0.018	0 (0)	-23264.55 (106.77)	0.153 (0.007)	0.146 - 0.172
RG	5000	25	0.60	0.80	0.618 (0.002)	0.501 (0.012)	0.018 (0.002)	0.012 - 0.023	0 (0)	-23356.3 (92.38)	0.299 (0.012)	0.246 - 0.316
RG	5000	25	0.60	0.90	0.623 (0.002)	0.498 (0.006)	0.023 (0.002)	0.019 - 0.028	0 (0)	-23397.93 (104.5)	0.402 (0.006)	0.386 - 0.416
RG	5000	25	0.70	0.10	0.624 (0.007)	0.726 (0.097)	0.076 (0.007)	0.07 - 0.111	0 (0)	-23214.45 (162.65)	0.626 (0.097)	0.59 - 1.307
RG	5000	25	0.70	0.20	0.641 (0.005)	0.645 (0.021)	0.059 (0.005)	0.05 - 0.068	0 (0)	-23253.21 (100.04)	0.445 (0.021)	0.415 - 0.478
RG	5000	25	0.70	0.30	0.661 (0.002)	0.588 (0.005)	0.039 (0.002)	0.033 - 0.043	0 (0)	-23030.01 (101.82)	0.288 (0.005)	0.273 - 0.297
RG	5000	25	0.70	0.40	0.686 (0.002)	0.537 (0.005)	0.014 (0.002)	0.01 - 0.019	0 (0)	-22835.02 (95.68)	0.137 (0.005)	0.127 - 0.152
RG	5000	25	0.70	0.50	0.685 (0.002)	0.538 (0.008)	0.015 (0.002)	0.011 - 0.021	0 (0)	-22984.5 (108.06)	0.038 (0.008)	0.025 - 0.057
RG	5000	25	0.70	0.60	0.699 (0.006)	0.499 (0.047)	0.006 (0.002)	0.002 - 0.01	0 (0)	-23452.8 (703.23)	0.101 (0.047)	0.045 - 0.155
RG	5000	25	0.70	0.70	0.702 (0.002)	0.461 (0.006)	0.002 (0.002)	0 - 0.008	0 (0)	-24065.36 (141.38)	0.239 (0.006)	0.23 - 0.253

Continued on next page

Table S1 – *Continued from previous page*

Structure	T	N	p	p_e/p_w	$\overline{\hat{p}}$ (SD)	$\overline{\hat{p}_e}/\overline{\hat{p}_w}$ (SD)	$\overline{\lvert\Delta\rvert}_p$ (SD)	Range $\lvert\Delta\rvert_p$	$\overline{SE}\lvert\Delta\rvert_p$ (SD)	$\overline{BIC}$ (SD)	$\overline{\lvert\Delta\rvert}_{p_e/p_w}$ (SD)	Range $\lvert\Delta\rvert_{p_e/p_w}$
RG	5000	25	0.70	0.80	0.714 (0.001)	0.5 (0.009)	0.014 (0.001)	0.007 - 0.017	0 (0)	-22824.9 (133.75)	0.3 (0.009)	0.283 - 0.336
RG	5000	25	0.70	0.90	0.716 (0.001)	0.5 (0.006)	0.016 (0.001)	0.013 - 0.019	0 (0)	-22976.47 (104.99)	0.4 (0.006)	0.382 - 0.407
RG	5000	25	0.80	0.10	0.669 (0.01)	0.771 (0.144)	0.131 (0.01)	0.118 - 0.185	0 (0)	-23254.57 (470.72)	0.671 (0.144)	0.592 - 1.494
RG	5000	25	0.80	0.20	0.729 (0.003)	0.664 (0.006)	0.071 (0.003)	0.064 - 0.079	0 (0)	-23188.3 (121.8)	0.464 (0.006)	0.436 - 0.478
RG	5000	25	0.80	0.30	0.767 (0.002)	0.588 (0.004)	0.033 (0.002)	0.028 - 0.037	0 (0)	-22246.79 (104.32)	0.288 (0.004)	0.275 - 0.294
RG	5000	25	0.80	0.40	0.792 (0.001)	0.538 (0.012)	0.008 (0.001)	0.004 - 0.011	0 (0)	-21435.92 (111.94)	0.138 (0.012)	0.093 - 0.188
RG	5000	25	0.80	0.50	0.794 (0.002)	0.577 (0.022)	0.006 (0.002)	0.003 - 0.012	0 (0)	-21509.67 (126.53)	0.077 (0.021)	0.004 - 0.101
RG	5000	25	0.80	0.60	0.801 (0.002)	0.527 (0.018)	0.002 (0.001)	0 - 0.005	0 (0)	-21179.58 (113.39)	0.073 (0.018)	0.048 - 0.107
RG	5000	25	0.80	0.70	0.803 (0.001)	0.499 (0.006)	0.003 (0.001)	0 - 0.006	0 (0)	-21125.49 (117)	0.201 (0.006)	0.166 - 0.209
RG	5000	25	0.80	0.80	0.804 (0.001)	0.498 (0.005)	0.004 (0.001)	0 - 0.006	0 (0)	-21143.15 (105.5)	0.302 (0.005)	0.285 - 0.307
RG	5000	25	0.80	0.90	0.804 (0.001)	0.499 (0.005)	0.004 (0.001)	0.002 - 0.007	0 (0)	-21177.07 (96.69)	0.401 (0.005)	0.385 - 0.408
RG	5000	25	0.90	0.10	0.727 (0.004)	0.748 (0.007)	0.173 (0.004)	0.167 - 0.183	0 (0)	-23445.7 (167.2)	0.648 (0.007)	0.633 - 0.667
RG	5000	25	0.90	0.20	0.869 (0.002)	0.702 (0.014)	0.031 (0.002)	0.026 - 0.04	0 (0)	-20483.82 (205.77)	0.502 (0.014)	0.412 - 0.516
RG	5000	25	0.90	0.30	0.893 (0.003)	0.6 (0.093)	0.007 (0.003)	0.003 - 0.013	0 (0)	-18560.59 (255.38)	0.3 (0.093)	0.112 - 0.372
RG	5000	25	0.90	0.40	0.899 (0.001)	0.414 (0.006)	0.001 (0.001)	0 - 0.005	0 (0)	-18060.56 (110.07)	0.014 (0.006)	0 - 0.024
RG	5000	25	0.90	0.50	0.898 (0.001)	0.416 (0.007)	0.002 (0.001)	0 - 0.004	0 (0)	-18158.11 (114.2)	0.084 (0.007)	0.075 - 0.098
RG	5000	25	0.90	0.60	0.9 (0.001)	0.41 (0.006)	0.001 (0.001)	0 - 0.002	0 (0)	-17932.09 (99.6)	0.19 (0.006)	0.178 - 0.201
RG	5000	25	0.90	0.70	0.9 (0.001)	0.409 (0.006)	0.001 (0.001)	0 - 0.002	0 (0)	-17909.92 (103.53)	0.291 (0.006)	0.278 - 0.3
RG	5000	25	0.90	0.80	0.9 (0.001)	0.412 (0.007)	0.001 (0.001)	0 - 0.003	0 (0)	-17899.14 (93.99)	0.388 (0.007)	0.378 - 0.4

Continued on next page

Table S1 – *Continued from previous page*

Structure	T	N	p	p_e/p_w	$\overline{\hat{p}}$ (SD)	$\overline{\hat{p}_e}/\overline{\hat{p}_w}$ (SD)	$\overline{\lvert\Delta\rvert}_p$ (SD)	Range $\lvert\Delta\rvert_p$	$\overline{SE}\lvert\Delta\rvert_p$ (SD)	$\overline{BIC}$ (SD)	$\overline{\lvert\Delta\rvert}_{p_e/p_w}$ (SD)	Range $\lvert\Delta\rvert_{p_e/p_w}$
RG	5000	25	0.90	0.90	0.9 (0.001)	0.411 (0.006)	0.001 (0.001)	0 - 0.002	0 (0)	-17902.52 (93.79)	0.489 (0.006)	0.478 - 0.5
RG	5000	49	0.10	0.10	0.101 (0.001)	0.746 (0.115)	0.001 (0.001)	0 - 0.003	0 (0)	-21534.24 (108.1)	0.646 (0.115)	0.442 - 0.762
RG	5000	49	0.10	0.20	0.104 (0.004)	0.748 (0.105)	0.004 (0.004)	0 - 0.012	0 (0)	-21737.75 (298.33)	0.548 (0.105)	0.356 - 0.67
RG	5000	49	0.10	0.30	0.1 (0.001)	0.717 (0.132)	0 (0)	0 - 0.001	0 (0)	-21444.6 (88.48)	0.417 (0.132)	0.241 - 0.57
RG	5000	49	0.10	0.40	0.1 (0.001)	0.721 (0.149)	0 (0)	0 - 0.001	0 (0)	-21461.21 (94.96)	0.321 (0.149)	0.039 - 0.469
RG	5000	49	0.10	0.50	0.1 (0.001)	0.711 (0.163)	0.001 (0)	0 - 0.002	0 (0)	-21432.62 (110.85)	0.222 (0.148)	0.015 - 0.37
RG	5000	49	0.10	0.60	0.1 (0.001)	0.712 (0.162)	0.001 (0)	0 - 0.002	0 (0)	-21440.24 (102.43)	0.163 (0.11)	0.001 - 0.345
RG	5000	49	0.10	0.70	0.1 (0.001)	0.733 (0.163)	0 (0)	0 - 0.001	0 (0)	-21438.51 (94.04)	0.159 (0.045)	0.086 - 0.353
RG	5000	49	0.10	0.80	0.1 (0.001)	0.679 (0.166)	0 (0)	0 - 0.002	0 (0)	-21437.13 (93.9)	0.173 (0.111)	0.049 - 0.41
RG	5000	49	0.10	0.90	0.1 (0.001)	0.695 (0.171)	0.001 (0)	0 - 0.001	0 (0)	-21437.98 (101.86)	0.205 (0.171)	0.03 - 0.522
RG	5000	49	0.20	0.10	0.209 (0.001)	0.753 (0.076)	0.009 (0.001)	0.006 - 0.011	0 (0)	-24702.34 (114.48)	0.653 (0.076)	0.339 - 0.718
RG	5000	49	0.20	0.20	0.204 (0.001)	0.687 (0.12)	0.004 (0.001)	0.001 - 0.006	0 (0)	-24535.48 (98.62)	0.487 (0.12)	0.258 - 0.622
RG	5000	49	0.20	0.30	0.201 (0.001)	0.689 (0.129)	0.001 (0.001)	0 - 0.004	0 (0)	-24473.43 (98.05)	0.389 (0.129)	0.2 - 0.528
RG	5000	49	0.20	0.40	0.2 (0.001)	0.691 (0.137)	0.001 (0)	0 - 0.002	0 (0)	-24443.28 (102.52)	0.291 (0.137)	0.113 - 0.428
RG	5000	49	0.20	0.50	0.2 (0.001)	0.684 (0.141)	0.001 (0.001)	0 - 0.003	0 (0)	-24431.03 (90.15)	0.184 (0.141)	0.015 - 0.327
RG	5000	49	0.20	0.60	0.2 (0.001)	0.657 (0.16)	0.001 (0)	0 - 0.002	0 (0)	-24429.6 (111.53)	0.159 (0.059)	0.049 - 0.228
RG	5000	49	0.20	0.70	0.2 (0.001)	0.656 (0.177)	0.001 (0.001)	0 - 0.002	0 (0)	-24426.93 (96.72)	0.171 (0.06)	0.1 - 0.318
RG	5000	49	0.20	0.80	0.2 (0.001)	0.605 (0.178)	0.001 (0.001)	0 - 0.002	0 (0)	-24404.77 (88.34)	0.21 (0.161)	0.002 - 0.533

Continued on next page

Table S1 – *Continued from previous page*

Structure	T	N	p	p_e/p_w	$\overline{\hat{p}}$ (SD)	$\overline{\hat{p}_e}/\overline{\hat{p}_w}$ (SD)	$\overline{\lvert\Delta\rvert}_p$ (SD)	Range $\lvert\Delta\rvert_p$	$\overline{SE}\lvert\Delta\rvert_p$ (SD)	$\overline{BIC}$ (SD)	$\overline{\lvert\Delta\rvert}_{p_e/p_w}$ (SD)	Range $\lvert\Delta\rvert_{p_e/p_w}$
RG	5000	49	0.20	0.90	0.2 (0.001)	0.622 (0.198)	0.001 (0)	0 - 0.002	0 (0)	-24421.59 (92.97)	0.278 (0.198)	0.069 - 0.684
RG	5000	49	0.30	0.10	0.327 (0.002)	0.678 (0.049)	0.027 (0.002)	0.019 - 0.031	0 (0)	-26424.04 (139.34)	0.578 (0.049)	0.413 - 0.609
RG	5000	49	0.30	0.20	0.315 (0.004)	0.586 (0.074)	0.015 (0.004)	0.008 - 0.022	0 (0)	-26249.34 (242.52)	0.386 (0.074)	0.305 - 0.489
RG	5000	49	0.30	0.30	0.306 (0.002)	0.522 (0.029)	0.006 (0.002)	0.002 - 0.013	0 (0)	-25991.77 (134.86)	0.222 (0.029)	0.19 - 0.37
RG	5000	49	0.30	0.40	0.303 (0.002)	0.526 (0.021)	0.003 (0.001)	0 - 0.009	0 (0)	-25902.28 (103.43)	0.126 (0.021)	0.098 - 0.309
RG	5000	49	0.30	0.50	0.301 (0.001)	0.537 (0.043)	0.001 (0.001)	0 - 0.005	0 (0)	-25846.3 (125.24)	0.037 (0.043)	0.013 - 0.204
RG	5000	49	0.30	0.60	0.299 (0.001)	0.53 (0.019)	0.001 (0.001)	0 - 0.004	0 (0)	-25882.44 (120.22)	0.071 (0.013)	0.03 - 0.086
RG	5000	49	0.30	0.70	0.298 (0.001)	0.529 (0.021)	0.002 (0.001)	0 - 0.005	0 (0)	-25883.69 (129.06)	0.171 (0.021)	0.016 - 0.202
RG	5000	49	0.30	0.80	0.297 (0.001)	0.532 (0.017)	0.003 (0.001)	0 - 0.007	0 (0)	-25963.02 (133.24)	0.268 (0.017)	0.23 - 0.303
RG	5000	49	0.30	0.90	0.297 (0.001)	0.529 (0.02)	0.003 (0.001)	0 - 0.007	0 (0)	-26030.61 (139.5)	0.371 (0.02)	0.322 - 0.405
RG	5000	49	0.40	0.10	0.425 (0.001)	0.586 (0.003)	0.025 (0.001)	0.022 - 0.028	0 (0)	-26658.43 (106.6)	0.486 (0.003)	0.481 - 0.492
RG	5000	49	0.40	0.20	0.424 (0.002)	0.581 (0.015)	0.024 (0.002)	0.018 - 0.027	0 (0)	-26723.44 (110.14)	0.381 (0.015)	0.332 - 0.394
RG	5000	49	0.40	0.30	0.415 (0.006)	0.543 (0.027)	0.015 (0.006)	0.008 - 0.025	0 (0)	-26729.24 (188.58)	0.243 (0.027)	0.217 - 0.292
RG	5000	49	0.40	0.40	0.416 (0.008)	0.561 (0.029)	0.016 (0.008)	0.004 - 0.023	0 (0)	-26980.22 (311.83)	0.161 (0.029)	0.115 - 0.19
RG	5000	49	0.40	0.50	0.417 (0.004)	0.58 (0.01)	0.017 (0.004)	0.008 - 0.022	0 (0)	-27215.3 (197.6)	0.08 (0.01)	0.06 - 0.091
RG	5000	49	0.40	0.60	0.412 (0.006)	0.572 (0.015)	0.013 (0.005)	0.001 - 0.02	0 (0)	-27436.23 (349.58)	0.028 (0.015)	0.009 - 0.08
RG	5000	49	0.40	0.70	0.399 (0.007)	0.545 (0.021)	0.007 (0.002)	0.002 - 0.012	0 (0)	-27015.74 (422.68)	0.155 (0.021)	0.111 - 0.185
RG	5000	49	0.40	0.80	0.402 (0.002)	0.566 (0.004)	0.002 (0.001)	0 - 0.009	0 (0)	-27466.45 (158.57)	0.234 (0.004)	0.231 - 0.275
RG	5000	49	0.40	0.90	0.399 (0.001)	0.568 (0.001)	0.001 (0.001)	0 - 0.003	0 (0)	-27937.79 (170.08)	0.332 (0.001)	0.33 - 0.334

Continued on next page

Table S1 – *Continued from previous page*

Structure	T	N	p	p_e/p_w	$\overline{\hat{p}}$ (SD)	$\overline{\hat{p_e}}/\overline{\hat{p_w}}$ (SD)	$\overline{\lvert\Delta\rvert}_p$ (SD)	Range $\lvert\Delta\rvert_p$	$\overline{SE}\lvert\Delta\rvert_p$ (SD)	$\overline{BIC}$ (SD)	$\overline{\lvert\Delta\rvert}_{p_e/p_w}$ (SD)	Range $\lvert\Delta\rvert_{p_e/p_w}$
RG	5000	49	0.50	0.10	0.5 (0.002)	0.546 (0.139)	0.002 (0.001)	0 - 0.005	0 (0)	-26682.36 (95.54)	0.45 (0.126)	0.085 - 0.88
RG	5000	49	0.50	0.20	0.5 (0.002)	0.536 (0.11)	0.002 (0.001)	0 - 0.006	0 (0)	-26709.44 (96.17)	0.336 (0.11)	0.102 - 0.786
RG	5000	49	0.50	0.30	0.5 (0.002)	0.551 (0.111)	0.002 (0.001)	0 - 0.005	0 (0)	-26685.29 (95.48)	0.251 (0.111)	0.044 - 0.685
RG	5000	49	0.50	0.40	0.5 (0.002)	0.566 (0.145)	0.002 (0.001)	0 - 0.005	0 (0)	-26694.84 (105.84)	0.172 (0.137)	0.009 - 0.809
RG	5000	49	0.50	0.50	0.5 (0.002)	0.559 (0.13)	0.002 (0.001)	0 - 0.004	0 (0)	-26684.32 (105.4)	0.093 (0.109)	0 - 0.553
RG	5000	49	0.50	0.60	0.5 (0.002)	0.538 (0.135)	0.002 (0.001)	0 - 0.006	0 (0)	-26695.84 (106.7)	0.114 (0.095)	0.003 - 0.537
RG	5000	49	0.50	0.70	0.5 (0.002)	0.531 (0.15)	0.002 (0.001)	0 - 0.005	0 (0)	-26699.39 (100.82)	0.198 (0.108)	0.006 - 0.655
RG	5000	49	0.50	0.80	0.5 (0.002)	0.54 (0.128)	0.002 (0.001)	0 - 0.005	0 (0)	-26705.35 (97.1)	0.278 (0.079)	0.032 - 0.46
RG	5000	49	0.50	0.90	0.5 (0.002)	0.552 (0.15)	0.002 (0.001)	0 - 0.005	0 (0)	-26697.64 (103.19)	0.356 (0.132)	0.012 - 0.855
RG	5000	49	0.60	0.10	0.559 (0.002)	0.627 (0.007)	0.041 (0.002)	0.037 - 0.047	0 (0)	-26691.62 (95.94)	0.527 (0.007)	0.517 - 0.549
RG	5000	49	0.60	0.20	0.566 (0.002)	0.573 (0.01)	0.034 (0.002)	0.029 - 0.04	0 (0)	-26706.52 (110.67)	0.373 (0.01)	0.355 - 0.388
RG	5000	49	0.60	0.30	0.581 (0.002)	0.538 (0.004)	0.019 (0.002)	0.015 - 0.022	0 (0)	-26650.06 (99.23)	0.238 (0.004)	0.232 - 0.248
RG	5000	49	0.60	0.40	0.592 (0.002)	0.525 (0.002)	0.008 (0.002)	0.004 - 0.013	0 (0)	-26614.28 (91.08)	0.125 (0.002)	0.118 - 0.131
RG	5000	49	0.60	0.50	0.599 (0.001)	0.521 (0.003)	0.002 (0.001)	0 - 0.005	0 (0)	-26594.39 (97.32)	0.021 (0.003)	0.014 - 0.028
RG	5000	49	0.60	0.60	0.605 (0.001)	0.527 (0.003)	0.005 (0.001)	0.002 - 0.008	0 (0)	-26612.44 (96.92)	0.073 (0.003)	0.07 - 0.083
RG	5000	49	0.60	0.70	0.611 (0.001)	0.507 (0.008)	0.011 (0.001)	0.007 - 0.014	0 (0)	-26690.74 (104.67)	0.193 (0.008)	0.18 - 0.209
RG	5000	49	0.60	0.80	0.614 (0.001)	0.508 (0.009)	0.014 (0.001)	0.011 - 0.018	0 (0)	-26773.19 (119.68)	0.292 (0.009)	0.273 - 0.304
RG	5000	49	0.60	0.90	0.618 (0.001)	0.504 (0.003)	0.018 (0.001)	0.015 - 0.02	0 (0)	-27011.26 (103.77)	0.396 (0.003)	0.383 - 0.405
RG	5000	49	0.70	0.10	0.611 (0.001)	0.643 (0.003)	0.089 (0.001)	0.085 - 0.092	0 (0)	-26860.42 (117.19)	0.543 (0.003)	0.538 - 0.553
RG	5000	49	0.70	0.20	0.645 (0.002)	0.581 (0.004)	0.055 (0.002)	0.051 - 0.058	0 (0)	-26877.82 (105.08)	0.381 (0.004)	0.375 - 0.389

Continued on next page

Table S1 – *Continued from previous page*

Structure	T	N	p	p_e/p_w	$\overline{\hat{p}}$ (SD)	$\overline{\hat{p_e}}/\overline{\hat{p_w}}$ (SD)	$\overline{\lvert\Delta\rvert}_p$ (SD)	Range $\lvert\Delta\rvert_p$	$\overline{SE}\lvert\Delta\rvert_p$ (SD)	$\overline{BIC}$ (SD)	$\overline{\lvert\Delta\rvert}_{p_e/p_w}$ (SD)	Range $\lvert\Delta\rvert_{p_e/p_w}$
RG	5000	49	0.70	0.30	0.683 (0.001)	0.541 (0.004)	0.017 (0.001)	0.014 - 0.02	0 (0)	-26312.89 (105.06)	0.241 (0.004)	0.234 - 0.25
RG	5000	49	0.70	0.40	0.69 (0.001)	0.471 (0.014)	0.01 (0.001)	0.007 - 0.013	0 (0)	-27109.19 (437.02)	0.071 (0.014)	0.053 - 0.129
RG	5000	49	0.70	0.50	0.699 (0.001)	0.521 (0.011)	0.001 (0.001)	0 - 0.008	0 (0)	-25971.73 (326.71)	0.023 (0.004)	0.015 - 0.041
RG	5000	49	0.70	0.60	0.703 (0.001)	0.514 (0.006)	0.003 (0.001)	0 - 0.005	0 (0)	-25927.53 (80.98)	0.086 (0.006)	0.075 - 0.105
RG	5000	49	0.70	0.70	0.704 (0.001)	0.502 (0.003)	0.004 (0.001)	0.003 - 0.007	0 (0)	-25958.71 (102.22)	0.198 (0.003)	0.187 - 0.201
RG	5000	49	0.70	0.80	0.705 (0.001)	0.502 (0.003)	0.005 (0.001)	0.003 - 0.007	0 (0)	-26020.24 (110.56)	0.298 (0.003)	0.292 - 0.307
RG	5000	49	0.70	0.90	0.705 (0.001)	0.5 (0.003)	0.005 (0.001)	0.003 - 0.008	0 (0)	-26102.92 (105.28)	0.4 (0.003)	0.393 - 0.407
RG	5000	49	0.80	0.10	0.69 (0.002)	0.667 (0.004)	0.11 (0.002)	0.105 - 0.116	0 (0)	-28213.47 (162.46)	0.567 (0.004)	0.545 - 0.576
RG	5000	49	0.80	0.20	0.779 (0.001)	0.621 (0.005)	0.021 (0.001)	0.018 - 0.026	0 (0)	-25673.55 (162.11)	0.421 (0.005)	0.401 - 0.43
RG	5000	49	0.80	0.30	0.798 (0.001)	0.564 (0.023)	0.002 (0.001)	0 - 0.005	0 (0)	-24544.94 (99.57)	0.264 (0.023)	0.171 - 0.289
RG	5000	49	0.80	0.40	0.8 (0.001)	0.541 (0.01)	0.001 (0.001)	0 - 0.004	0 (0)	-24446.7 (95.93)	0.141 (0.01)	0.096 - 0.171
RG	5000	49	0.80	0.50	0.8 (0.001)	0.524 (0.012)	0.001 (0.001)	0 - 0.003	0 (0)	-24431.95 (97.43)	0.025 (0.011)	0.003 - 0.049
RG	5000	49	0.80	0.60	0.8 (0.001)	0.504 (0.011)	0.001 (0)	0 - 0.002	0 (0)	-24413.9 (110.02)	0.096 (0.011)	0.072 - 0.122
RG	5000	49	0.80	0.70	0.8 (0.001)	0.494 (0.011)	0.001 (0)	0 - 0.002	0 (0)	-24436.25 (100.18)	0.206 (0.011)	0.18 - 0.231
RG	5000	49	0.80	0.80	0.8 (0.001)	0.491 (0.012)	0.001 (0.001)	0 - 0.002	0 (0)	-24444.58 (95.12)	0.309 (0.012)	0.273 - 0.344
RG	5000	49	0.80	0.90	0.8 (0.001)	0.49 (0.011)	0.001 (0)	0 - 0.002	0 (0)	-24416.49 (99.82)	0.41 (0.011)	0.39 - 0.43
RG	5000	49	0.90	0.10	0.86 (0.009)	0.706 (0.074)	0.04 (0.009)	0.03 - 0.054	0 (0)	-26270.02 (1171.71)	0.606 (0.074)	0.498 - 0.675
RG	5000	49	0.90	0.20	0.895 (0.001)	0.478 (0.103)	0.005 (0.001)	0.002 - 0.007	0 (0)	-21817.02 (128.07)	0.278 (0.103)	0.168 - 0.511
RG	5000	49	0.90	0.30	0.9 (0.001)	0.457 (0.087)	0.001 (0)	0 - 0.002	0 (0)	-21457.59 (98.36)	0.157 (0.087)	0.054 - 0.344

Continued on next page

Table S1 – *Continued from previous page*

Structure	T	N	p	p_e/p_w	$\overline{\hat{p}}$ (SD)	$\overline{\hat{p}_e}/\overline{\hat{p}_w}$ (SD)	$\overline{\lvert\Delta\rvert}_p$ (SD)	Range $\lvert\Delta\rvert_p$	$\overline{SE}\lvert\Delta\rvert_p$ (SD)	$\overline{BIC}$ (SD)	$\overline{\lvert\Delta\rvert}_{p_e/p_w}$ (SD)	Range $\lvert\Delta\rvert_{p_e/p_w}$
RG	5000	49	0.90	0.40	0.9 (0.001)	0.441 (0.065)	0 (0)	0 - 0.002	0 (0)	-21452.26 (96.05)	0.054 (0.055)	0 - 0.211
RG	5000	49	0.90	0.50	0.9 (0.001)	0.452 (0.072)	0 (0)	0 - 0.001	0 (0)	-21446.95 (86.83)	0.078 (0.037)	0.008 - 0.148
RG	5000	49	0.90	0.60	0.9 (0.001)	0.453 (0.07)	0 (0)	0 - 0.002	0 (0)	-21441.13 (90.89)	0.149 (0.066)	0.001 - 0.239
RG	5000	49	0.90	0.70	0.9 (0.001)	0.442 (0.063)	0 (0)	0 - 0.002	0 (0)	-21449.66 (86.34)	0.258 (0.063)	0.085 - 0.355
RG	5000	49	0.90	0.80	0.9 (0.001)	0.442 (0.063)	0.001 (0)	0 - 0.002	0 (0)	-21446.65 (99.04)	0.358 (0.063)	0.175 - 0.447
RG	5000	49	0.90	0.90	0.9 (0.001)	0.451 (0.076)	0 (0)	0 - 0.001	0 (0)	-21432.82 (80.03)	0.449 (0.076)	0.266 - 0.575
RG	5000	100	0.10	0.10	0.106 (0.006)	0.769 (0.068)	0.006 (0.006)	0 - 0.013	0 (0)	-25538.6 (418.28)	0.669 (0.068)	0.49 - 0.781
RG	5000	100	0.10	0.20	0.1 (0)	0.741 (0.088)	0 (0)	0 - 0.001	0 (0)	-25084.9 (90.65)	0.541 (0.088)	0.356 - 0.681
RG	5000	100	0.10	0.30	0.1 (0)	0.742 (0.098)	0 (0)	0 - 0.001	0 (0)	-25099.1 (99.95)	0.442 (0.098)	0.257 - 0.58
RG	5000	100	0.10	0.40	0.1 (0)	0.758 (0.089)	0 (0)	0 - 0.001	0 (0)	-25077.12 (99.11)	0.358 (0.089)	0.154 - 0.5
RG	5000	100	0.10	0.50	0.1 (0)	0.758 (0.096)	0 (0)	0 - 0.001	0 (0)	-25100.71 (91.4)	0.258 (0.096)	0.019 - 0.381
RG	5000	100	0.10	0.60	0.1 (0)	0.732 (0.099)	0 (0)	0 - 0.001	0 (0)	-25091.28 (88.44)	0.135 (0.094)	0 - 0.281
RG	5000	100	0.10	0.70	0.1 (0)	0.748 (0.093)	0 (0)	0 - 0.001	0 (0)	-25078.4 (102)	0.091 (0.051)	0.005 - 0.181
RG	5000	100	0.10	0.80	0.1 (0)	0.733 (0.097)	0 (0)	0 - 0.001	0 (0)	-25095.33 (94.91)	0.099 (0.063)	0 - 0.227
RG	5000	100	0.10	0.90	0.1 (0)	0.751 (0.099)	0 (0)	0 - 0.002	0 (0)	-25086.67 (100.23)	0.149 (0.099)	0.019 - 0.384
RG	5000	100	0.20	0.10	0.205 (0.001)	0.691 (0.111)	0.005 (0.001)	0.003 - 0.006	0 (0)	-28177.76 (110.68)	0.591 (0.111)	0.404 - 0.718
RG	5000	100	0.20	0.20	0.202 (0.002)	0.708 (0.104)	0.002 (0.001)	0 - 0.005	0 (0)	-28090.03 (114.7)	0.508 (0.104)	0.345 - 0.627
RG	5000	100	0.20	0.30	0.2 (0.001)	0.701 (0.111)	0.001 (0)	0 - 0.002	0 (0)	-28020.42 (104.9)	0.401 (0.111)	0.163 - 0.528

Continued on next page

Table S1 – *Continued from previous page*

Structure	T	N	p	p_e/p_w	$\overline{\hat{p}}$ (SD)	$\overline{\hat{p_e}}/\overline{\hat{p_w}}$ (SD)	$\overline{\lvert\Delta\rvert}_p$ (SD)	Range $\lvert\Delta\rvert_p$	$\overline{SE}\lvert\Delta\rvert_p$ (SD)	$\overline{BIC}$ (SD)	$\overline{\lvert\Delta\rvert}_{p_e/p_w}$ (SD)	Range $\lvert\Delta\rvert_{p_e/p_w}$
RG	5000	100	0.20	0.40	0.2 (0.001)	0.714 (0.11)	0.001 (0)	0 - 0.002	0 (0)	-28040.89 (99.43)	0.314 (0.11)	0.122 - 0.427
RG	5000	100	0.20	0.50	0.2 (0.001)	0.7 (0.114)	0.001 (0)	0 - 0.001	0 (0)	-28039.06 (111.06)	0.201 (0.113)	0.011 - 0.327
RG	5000	100	0.20	0.60	0.2 (0.001)	0.695 (0.127)	0.001 (0)	0 - 0.001	0 (0)	-28026.55 (86.13)	0.123 (0.099)	0 - 0.228
RG	5000	100	0.20	0.70	0.2 (0)	0.734 (0.109)	0 (0)	0 - 0.001	0 (0)	-28022.8 (104.7)	0.112 (0.021)	0.085 - 0.237
RG	5000	100	0.20	0.80	0.2 (0.001)	0.7 (0.12)	0.001 (0)	0 - 0.001	0 (0)	-28041.33 (120.7)	0.116 (0.104)	0.001 - 0.397
RG	5000	100	0.20	0.90	0.2 (0.001)	0.726 (0.123)	0.001 (0)	0 - 0.001	0 (0)	-28034.31 (115.59)	0.174 (0.123)	0.072 - 0.535
RG	5000	100	0.30	0.10	0.321 (0.002)	0.54 (0.096)	0.021 (0.002)	0.017 - 0.025	0 (0)	-29938.91 (177.24)	0.44 (0.096)	0.371 - 0.61
RG	5000	100	0.30	0.20	0.306 (0.001)	0.591 (0.112)	0.006 (0.001)	0.003 - 0.008	0 (0)	-29569.24 (113.08)	0.391 (0.112)	0.27 - 0.522
RG	5000	100	0.30	0.30	0.303 (0.001)	0.608 (0.099)	0.003 (0.001)	0 - 0.005	0 (0)	-29487.52 (113.7)	0.308 (0.099)	0.173 - 0.415
RG	5000	100	0.30	0.40	0.301 (0.001)	0.586 (0.094)	0.001 (0.001)	0 - 0.003	0 (0)	-29410.83 (95.96)	0.186 (0.094)	0.093 - 0.325
RG	5000	100	0.30	0.50	0.3 (0.001)	0.612 (0.102)	0.001 (0)	0 - 0.002	0 (0)	-29408.63 (96)	0.113 (0.101)	0.001 - 0.226
RG	5000	100	0.30	0.60	0.3 (0.001)	0.595 (0.107)	0.001 (0)	0 - 0.002	0 (0)	-29370.19 (94.26)	0.106 (0.013)	0.077 - 0.125
RG	5000	100	0.30	0.70	0.3 (0.001)	0.595 (0.111)	0.001 (0)	0 - 0.002	0 (0)	-29392.54 (91.29)	0.12 (0.094)	0.002 - 0.235
RG	5000	100	0.30	0.80	0.3 (0.001)	0.591 (0.117)	0.001 (0)	0 - 0.002	0 (0)	-29387.71 (97.18)	0.209 (0.117)	0.07 - 0.495
RG	5000	100	0.30	0.90	0.3 (0.001)	0.582 (0.119)	0.001 (0)	0 - 0.002	0 (0)	-29394.54 (93.73)	0.318 (0.119)	0.175 - 0.574
RG	5000	100	0.40	0.10	0.431 (0.002)	0.542 (0.008)	0.031 (0.002)	0.028 - 0.034	0 (0)	-30355.72 (99.33)	0.442 (0.008)	0.431 - 0.466
RG	5000	100	0.40	0.20	0.42 (0.001)	0.525 (0.003)	0.02 (0.001)	0.016 - 0.022	0 (0)	-30340.75 (99.11)	0.325 (0.003)	0.312 - 0.33
RG	5000	100	0.40	0.30	0.413 (0.001)	0.516 (0.002)	0.013 (0.001)	0.01 - 0.015	0 (0)	-30268.82 (105.12)	0.216 (0.002)	0.21 - 0.219
RG	5000	100	0.40	0.40	0.406 (0.001)	0.516 (0.002)	0.006 (0.001)	0.004 - 0.008	0 (0)	-30170.85 (90.54)	0.116 (0.002)	0.111 - 0.119

Continued on next page

Table S1 – *Continued from previous page*

Structure	T	N	p	p_e/p_w	$\overline{\hat{p}}$ (SD)	$\overline{\hat{p_e}}/\overline{\hat{p_w}}$ (SD)	$\overline{\lvert\Delta\rvert}_p$ (SD)	Range $\lvert\Delta\rvert_p$	$\overline{SE}\lvert\Delta\rvert_p$ (SD)	$\overline{BIC}$ (SD)	$\overline{\lvert\Delta\rvert}_{p_e/p_w}$ (SD)	Range $\lvert\Delta\rvert_{p_e/p_w}$
RG	5000	100	0.40	0.50	0.4 (0.002)	0.51 (0.005)	0.001 (0.001)	0 - 0.004	0 (0)	-30150.68 (107.33)	0.01 (0.005)	0.001 - 0.018
RG	5000	100	0.40	0.60	0.397 (0.001)	0.51 (0.004)	0.003 (0.001)	0.001 - 0.005	0 (0)	-30128.56 (108.05)	0.09 (0.004)	0.081 - 0.098
RG	5000	100	0.40	0.70	0.394 (0.001)	0.509 (0.005)	0.006 (0.001)	0.003 - 0.008	0 (0)	-30220.37 (96.51)	0.191 (0.005)	0.181 - 0.196
RG	5000	100	0.40	0.80	0.393 (0.001)	0.509 (0.005)	0.007 (0.001)	0.005 - 0.009	0 (0)	-30372.77 (97.28)	0.291 (0.005)	0.281 - 0.297
RG	5000	100	0.40	0.90	0.392 (0.001)	0.507 (0.004)	0.008 (0.001)	0.006 - 0.01	0 (0)	-30561.45 (105.56)	0.393 (0.004)	0.381 - 0.398
RG	5000	100	0.50	0.10	0.5 (0.001)	0.53 (0.108)	0.001 (0.001)	0 - 0.003	0 (0)	-30268.25 (101.59)	0.43 (0.108)	0.026 - 0.762
RG	5000	100	0.50	0.20	0.5 (0.001)	0.558 (0.102)	0.001 (0.001)	0 - 0.004	0 (0)	-30253.85 (99.02)	0.358 (0.102)	0.157 - 0.777
RG	5000	100	0.50	0.30	0.5 (0.001)	0.549 (0.087)	0.001 (0.001)	0 - 0.004	0 (0)	-30244.26 (110.56)	0.249 (0.087)	0.122 - 0.531
RG	5000	100	0.50	0.40	0.5 (0.001)	0.555 (0.118)	0.001 (0.001)	0 - 0.004	0 (0)	-30262.58 (90.78)	0.16 (0.111)	0.033 - 0.553
RG	5000	100	0.50	0.50	0.5 (0.001)	0.542 (0.098)	0.001 (0.001)	0 - 0.003	0 (0)	-30243.01 (108.71)	0.06 (0.088)	0 - 0.457
RG	5000	100	0.50	0.60	0.5 (0.002)	0.551 (0.09)	0.001 (0.001)	0 - 0.004	0 (0)	-30251.63 (94.18)	0.087 (0.055)	0.004 - 0.292
RG	5000	100	0.50	0.70	0.5 (0.001)	0.563 (0.125)	0.001 (0.001)	0 - 0.004	0 (0)	-30251.55 (117.05)	0.163 (0.087)	0.006 - 0.658
RG	5000	100	0.50	0.80	0.5 (0.001)	0.556 (0.146)	0.001 (0.001)	0 - 0.003	0 (0)	-30238.11 (104.25)	0.262 (0.111)	0.018 - 0.638
RG	5000	100	0.50	0.90	0.5 (0.001)	0.574 (0.123)	0.001 (0.001)	0 - 0.004	0 (0)	-30264.44 (95.67)	0.327 (0.121)	0.011 - 0.573
RG	5000	100	0.60	0.10	0.55 (0.002)	0.568 (0.005)	0.05 (0.002)	0.047 - 0.054	0 (0)	-30327.23 (104.05)	0.468 (0.005)	0.462 - 0.479
RG	5000	100	0.60	0.20	0.567 (0.001)	0.535 (0.002)	0.033 (0.001)	0.03 - 0.037	0 (0)	-30336.28 (111.38)	0.335 (0.002)	0.331 - 0.338
RG	5000	100	0.60	0.30	0.58 (0.001)	0.521 (0.004)	0.02 (0.001)	0.018 - 0.023	0 (0)	-30309.72 (115.22)	0.221 (0.004)	0.212 - 0.228
RG	5000	100	0.60	0.40	0.592 (0.001)	0.514 (0.001)	0.008 (0.001)	0.005 - 0.011	0 (0)	-30170.85 (96.15)	0.114 (0.001)	0.112 - 0.118
RG	5000	100	0.60	0.50	0.599 (0.001)	0.508 (0.005)	0.001 (0.001)	0 - 0.004	0 (0)	-30153.62 (90.12)	0.008 (0.005)	0.001 - 0.018

Continued on next page

Table S1 – *Continued from previous page*

Structure	T	N	p	p_e/p_w	$\overline{\hat{p}}$ (SD)	$\overline{\hat{p}_e}/\overline{\hat{p}_w}$ (SD)	$\overline{\lvert\Delta\rvert}_p$ (SD)	Range $\lvert\Delta\rvert_p$	$\overline{SE}\lvert\Delta\rvert_p$ (SD)	$\overline{BIC}$ (SD)	$\overline{\lvert\Delta\rvert}_{p_e/p_w}$ (SD)	Range $\lvert\Delta\rvert_{p_e/p_w}$
RG	5000	100	0.60	0.60	0.604 (0.001)	0.505 (0.002)	0.004 (0.001)	0.002 - 0.006	0 (0)	-30140.29 (92.14)	0.095 (0.002)	0.091 - 0.1
RG	5000	100	0.60	0.70	0.607 (0.001)	0.505 (0.002)	0.007 (0.001)	0.005 - 0.009	0 (0)	-30226.64 (94.66)	0.195 (0.002)	0.191 - 0.198
RG	5000	100	0.60	0.80	0.609 (0.001)	0.505 (0.001)	0.009 (0.001)	0.007 - 0.01	0 (0)	-30374.68 (123.2)	0.295 (0.001)	0.293 - 0.299
RG	5000	100	0.60	0.90	0.61 (0.001)	0.504 (0.003)	0.01 (0.001)	0.008 - 0.011	0 (0)	-30664.9 (110.04)	0.396 (0.003)	0.393 - 0.406
RG	5000	100	0.70	0.10	0.61 (0.002)	0.576 (0.002)	0.09 (0.002)	0.086 - 0.094	0 (0)	-31298.8 (132.12)	0.476 (0.002)	0.471 - 0.48
RG	5000	100	0.70	0.20	0.68 (0.001)	0.553 (0.008)	0.02 (0.001)	0.017 - 0.026	0 (0)	-30435.75 (178.68)	0.353 (0.008)	0.292 - 0.363
RG	5000	100	0.70	0.30	0.694 (0.001)	0.53 (0.02)	0.006 (0.001)	0.003 - 0.009	0 (0)	-29670.07 (164)	0.23 (0.02)	0.184 - 0.249
RG	5000	100	0.70	0.40	0.699 (0.001)	0.524 (0.006)	0.001 (0.001)	0 - 0.003	0 (0)	-29426.82 (107.35)	0.124 (0.006)	0.11 - 0.137
RG	5000	100	0.70	0.50	0.7 (0.001)	0.509 (0.006)	0.001 (0)	0 - 0.002	0 (0)	-29390.37 (89.79)	0.01 (0.005)	0.001 - 0.02
RG	5000	100	0.70	0.60	0.701 (0.001)	0.502 (0.005)	0.001 (0)	0 - 0.002	0 (0)	-29396.9 (110.8)	0.098 (0.005)	0.083 - 0.109
RG	5000	100	0.70	0.70	0.701 (0.001)	0.499 (0.004)	0.001 (0)	0 - 0.002	0 (0)	-29405.05 (93.88)	0.201 (0.004)	0.192 - 0.209
RG	5000	100	0.70	0.80	0.701 (0.001)	0.497 (0.005)	0.001 (0)	0 - 0.002	0 (0)	-29413.55 (102.59)	0.303 (0.005)	0.291 - 0.317
RG	5000	100	0.70	0.90	0.701 (0.001)	0.497 (0.005)	0.001 (0.001)	0 - 0.002	0 (0)	-29415.56 (86.54)	0.403 (0.005)	0.391 - 0.419
RG	5000	100	0.80	0.10	0.773 (0.001)	0.66 (0.008)	0.027 (0.001)	0.024 - 0.03	0 (0)	-30042.32 (221.16)	0.56 (0.008)	0.532 - 0.572
RG	5000	100	0.80	0.20	0.798 (0.001)	0.465 (0.065)	0.002 (0.001)	0.001 - 0.004	0 (0)	-28119.9 (107.13)	0.265 (0.065)	0.201 - 0.429
RG	5000	100	0.80	0.30	0.8 (0.001)	0.468 (0.054)	0.001 (0)	0 - 0.001	0 (0)	-28014.62 (102.42)	0.168 (0.054)	0.102 - 0.296
RG	5000	100	0.80	0.40	0.8 (0.001)	0.464 (0.037)	0 (0)	0 - 0.001	0 (0)	-28003.3 (97.81)	0.064 (0.037)	0 - 0.181
RG	5000	100	0.80	0.50	0.8 (0.001)	0.465 (0.035)	0 (0)	0 - 0.002	0 (0)	-27996.99 (81.41)	0.044 (0.023)	0.001 - 0.09

Continued on next page

Table S1 – *Continued from previous page*

Structure	T	N	p	p_e/p_w	$\overline{\hat{p}}$ (SD)	$\overline{\hat{p}_e}/\overline{\hat{p}_w}$ (SD)	$\overline{\lvert\Delta\rvert}_p$ (SD)	Range $\lvert\Delta\rvert_p$	$\overline{SE}\lvert\Delta\rvert_p$ (SD)	$\overline{BIC}$ (SD)	$\overline{\lvert\Delta\rvert}_{p_e/p_w}$ (SD)	Range $\lvert\Delta\rvert_{p_e/p_w}$
RG	5000	100	0.80	0.60	0.8 (0.001)	0.464 (0.038)	0 (0)	0 - 0.002	0 (0)	-27993.69 (105.39)	0.136 (0.038)	0.012 - 0.204
RG	5000	100	0.80	0.70	0.8 (0.001)	0.469 (0.04)	0 (0)	0 - 0.001	0 (0)	-27996.69 (103.24)	0.231 (0.04)	0.121 - 0.302
RG	5000	100	0.80	0.80	0.8 (0.001)	0.466 (0.039)	0.001 (0)	0 - 0.002	0 (0)	-27999.34 (103.53)	0.334 (0.039)	0.216 - 0.402
RG	5000	100	0.80	0.90	0.8 (0.001)	0.465 (0.037)	0 (0)	0 - 0.001	0 (0)	-28003.65 (95.51)	0.435 (0.037)	0.314 - 0.507
RG	5000	100	0.90	0.10	0.895 (0.001)	0.396 (0.084)	0.005 (0.001)	0.004 - 0.007	0 (0)	-25567.43 (116.78)	0.296 (0.084)	0.197 - 0.517
RG	5000	100	0.90	0.20	0.9 (0)	0.359 (0.068)	0 (0)	0 - 0.002	0 (0)	-25090.77 (92.09)	0.159 (0.068)	0.071 - 0.3
RG	5000	100	0.90	0.30	0.9 (0)	0.384 (0.07)	0 (0)	0 - 0.001	0 (0)	-25084.85 (99)	0.089 (0.063)	0.001 - 0.2
RG	5000	100	0.90	0.40	0.9 (0)	0.371 (0.07)	0 (0)	0 - 0.001	0 (0)	-25087.48 (106.07)	0.066 (0.036)	0 - 0.142
RG	5000	100	0.90	0.50	0.9 (0)	0.363 (0.069)	0 (0)	0 - 0.001	0 (0)	-25091.59 (96.09)	0.137 (0.069)	0 - 0.231
RG	5000	100	0.90	0.60	0.9 (0)	0.384 (0.069)	0 (0)	0 - 0.001	0 (0)	-25081.83 (91.09)	0.216 (0.069)	0.1 - 0.327
RG	5000	100	0.90	0.70	0.9 (0)	0.383 (0.072)	0 (0)	0 - 0.001	0 (0)	-25083.66 (92.49)	0.317 (0.072)	0.2 - 0.423
RG	5000	100	0.90	0.80	0.9 (0)	0.379 (0.077)	0 (0)	0 - 0.001	0 (0)	-25082.85 (113.02)	0.421 (0.077)	0.275 - 0.538
RG	5000	100	0.90	0.90	0.9 (0)	0.379 (0.071)	0 (0)	0 - 0.001	0 (0)	-25072.04 (95.39)	0.521 (0.071)	0.4 - 0.623
SWG	50	16	0.10	0.10	0.106 (0.016)	0.294 (0.185)	0.013 (0.011)	0 - 0.052	0.002 (0)	-147.39 (10.36)	0.208 (0.169)	0.004 - 0.785
SWG	50	16	0.10	0.20	0.101 (0.018)	0.224 (0.158)	0.013 (0.012)	0 - 0.076	0.002 (0)	-148.29 (10.94)	0.12 (0.105)	0.001 - 0.557
SWG	50	16	0.10	0.30	0.106 (0.018)	0.278 (0.18)	0.015 (0.012)	0 - 0.061	0.002 (0)	-147.87 (11.96)	0.146 (0.107)	0 - 0.659
SWG	50	16	0.10	0.40	0.105 (0.016)	0.258 (0.173)	0.013 (0.01)	0 - 0.044	0.002 (0)	-147.97 (10.25)	0.2 (0.1)	0.005 - 0.395
SWG	50	16	0.10	0.50	0.102 (0.018)	0.244 (0.191)	0.014 (0.01)	0 - 0.047	0.002 (0)	-148.97 (12.34)	0.28 (0.154)	0.003 - 0.494
SWG	50	16	0.10	0.60	0.098 (0.048)	0.254 (0.203)	0.022 (0.043)	0 - 0.314	0.002 (0)	-146.86 (10.76)	0.359 (0.178)	0.006 - 0.599

Continued on next page

Table S1 – *Continued from previous page*

Structure	T	N	p	p_e/p_w	$\overline{\hat{p}}$ (SD)	$\overline{\hat{p}_e}/\overline{\hat{p}_w}$ (SD)	$\overline{\lvert\Delta\rvert}_p$ (SD)	Range $\lvert\Delta\rvert_p$	$\overline{SE}\lvert\Delta\rvert_p$ (SD)	$\overline{BIC}$ (SD)	$\overline{\lvert\Delta\rvert}_{p_e/p_w}$ (SD)	Range $\lvert\Delta\rvert_{p_e/p_w}$
SWG	50	16	0.10	0.70	0.103 (0.025)	0.224 (0.184)	0.016 (0.02)	0.001 - 0.156	0.002 (0)	-148.47 (11.16)	0.476 (0.184)	0.001 - 0.699
SWG	50	16	0.10	0.80	0.102 (0.036)	0.254 (0.206)	0.02 (0.029)	0 - 0.222	0.002 (0)	-148.04 (10.25)	0.55 (0.195)	0.052 - 0.797
SWG	50	16	0.10	0.90	0.109 (0.083)	0.274 (0.217)	0.034 (0.076)	0 - 0.467	0.002 (0)	-148.4 (10.32)	0.626 (0.217)	0.173 - 0.9
SWG	50	16	0.20	0.10	0.21 (0.029)	0.327 (0.155)	0.023 (0.021)	0 - 0.102	0.002 (0)	-178.83 (10.15)	0.228 (0.154)	0.005 - 0.627
SWG	50	16	0.20	0.20	0.215 (0.03)	0.32 (0.149)	0.026 (0.021)	0.001 - 0.088	0.003 (0)	-180.28 (11.05)	0.144 (0.126)	0.001 - 0.462
SWG	50	16	0.20	0.30	0.21 (0.027)	0.324 (0.124)	0.021 (0.019)	0.001 - 0.1	0.002 (0)	-179.11 (10.05)	0.102 (0.075)	0.001 - 0.325
SWG	50	16	0.20	0.40	0.21 (0.028)	0.327 (0.145)	0.023 (0.018)	0 - 0.079	0.002 (0)	-180.46 (10.24)	0.135 (0.09)	0.001 - 0.409
SWG	50	16	0.20	0.50	0.205 (0.031)	0.284 (0.154)	0.024 (0.02)	0 - 0.116	0.003 (0)	-179.24 (9.81)	0.236 (0.12)	0.009 - 0.457
SWG	50	16	0.20	0.60	0.204 (0.03)	0.322 (0.175)	0.025 (0.018)	0.001 - 0.086	0.002 (0)	-179.34 (11.21)	0.286 (0.162)	0.006 - 0.598
SWG	50	16	0.20	0.70	0.211 (0.032)	0.313 (0.169)	0.026 (0.021)	0 - 0.085	0.003 (0)	-180.2 (8.92)	0.388 (0.167)	0.008 - 0.679
SWG	50	16	0.20	0.80	0.209 (0.031)	0.306 (0.166)	0.026 (0.02)	0 - 0.113	0.003 (0)	-179.86 (9.73)	0.494 (0.166)	0.006 - 0.78
SWG	50	16	0.20	0.90	0.205 (0.03)	0.31 (0.175)	0.025 (0.019)	0 - 0.082	0.002 (0)	-179.37 (10.02)	0.59 (0.175)	0.058 - 0.879
SWG	50	16	0.30	0.10	0.306 (0.031)	0.258 (0.078)	0.024 (0.02)	0 - 0.093	0.004 (0)	-192.77 (10.64)	0.158 (0.078)	0.008 - 0.354
SWG	50	16	0.30	0.20	0.307 (0.034)	0.224 (0.087)	0.027 (0.022)	0.001 - 0.123	0.004 (0)	-193.74 (9.02)	0.071 (0.055)	0 - 0.239
SWG	50	16	0.30	0.30	0.306 (0.035)	0.264 (0.089)	0.027 (0.023)	0 - 0.106	0.004 (0)	-190.83 (8.77)	0.079 (0.054)	0.004 - 0.226
SWG	50	16	0.30	0.40	0.307 (0.035)	0.24 (0.093)	0.029 (0.021)	0.001 - 0.099	0.004 (0)	-193.7 (10.73)	0.168 (0.078)	0.008 - 0.35
SWG	50	16	0.30	0.50	0.295 (0.035)	0.187 (0.086)	0.027 (0.022)	0 - 0.08	0.004 (0)	-193.19 (9.58)	0.313 (0.086)	0.102 - 0.488
SWG	50	16	0.30	0.60	0.293 (0.035)	0.225 (0.094)	0.029 (0.021)	0.002 - 0.144	0.004 (0)	-192.61 (9.83)	0.375 (0.094)	0.102 - 0.572
SWG	50	16	0.30	0.70	0.299 (0.031)	0.205 (0.094)	0.024 (0.019)	0 - 0.102	0.004 (0)	-191.69 (9.3)	0.495 (0.094)	0.217 - 0.682

Continued on next page

Table S1 – *Continued from previous page*

Structure	T	N	p	p_e/p_w	$\overline{\hat{p}}$ (SD)	$\overline{\hat{p}_e}/\overline{\hat{p}_w}$ (SD)	$\overline{\lvert\Delta\rvert}_p$ (SD)	Range $\lvert\Delta\rvert_p$	$\overline{SE}\lvert\Delta\rvert_p$ (SD)	$\overline{BIC}$ (SD)	$\overline{\lvert\Delta\rvert}_{p_e/p_w}$ (SD)	Range $\lvert\Delta\rvert_{p_e/p_w}$
SWG	50	16	0.30	0.80	0.301 (0.036)	0.202 (0.084)	0.027 (0.024)	0 - 0.128	0.004 (0)	-191.7 (8.56)	0.598 (0.084)	0.34 - 0.75
SWG	50	16	0.30	0.90	0.301 (0.032)	0.218 (0.086)	0.025 (0.02)	0 - 0.087	0.004 (0)	-190.94 (9.06)	0.682 (0.086)	0.387 - 0.865
SWG	50	16	0.40	0.10	0.402 (0.038)	0.138 (0.051)	0.031 (0.021)	0.001 - 0.111	0.005 (0)	-195.41 (8.97)	0.052 (0.037)	0.002 - 0.17
SWG	50	16	0.40	0.20	0.401 (0.038)	0.116 (0.049)	0.03 (0.023)	0 - 0.112	0.005 (0)	-196.27 (10.31)	0.086 (0.045)	0.001 - 0.2
SWG	50	16	0.40	0.30	0.401 (0.035)	0.127 (0.063)	0.028 (0.021)	0.001 - 0.094	0.005 (0)	-196.13 (9.42)	0.173 (0.062)	0.012 - 0.291
SWG	50	16	0.40	0.40	0.402 (0.033)	0.126 (0.057)	0.026 (0.019)	0 - 0.074	0.005 (0)	-196.01 (9.63)	0.274 (0.057)	0.084 - 0.384
SWG	50	16	0.40	0.50	0.395 (0.038)	0.099 (0.06)	0.031 (0.022)	0.001 - 0.1	0.005 (0)	-195.43 (10.32)	0.401 (0.06)	0.245 - 0.498
SWG	50	16	0.40	0.60	0.385 (0.038)	0.105 (0.058)	0.033 (0.025)	0.002 - 0.108	0.005 (0)	-195.91 (8.66)	0.495 (0.058)	0.349 - 0.596
SWG	50	16	0.40	0.70	0.395 (0.041)	0.114 (0.064)	0.035 (0.021)	0.001 - 0.081	0.005 (0)	-196.5 (8.48)	0.586 (0.064)	0.426 - 0.698
SWG	50	16	0.40	0.80	0.391 (0.034)	0.104 (0.054)	0.028 (0.02)	0 - 0.09	0.005 (0)	-196.51 (9.94)	0.696 (0.054)	0.541 - 0.797
SWG	50	16	0.40	0.90	0.394 (0.034)	0.108 (0.059)	0.028 (0.021)	0 - 0.083	0.005 (0)	-195.88 (9.13)	0.792 (0.059)	0.585 - 0.899
SWG	50	16	0.50	0.10	0.507 (0.044)	0.037 (0.026)	0.035 (0.027)	0 - 0.143	0.006 (0)	-195.74 (8.3)	0.064 (0.023)	0.002 - 0.1
SWG	50	16	0.50	0.20	0.506 (0.042)	0.042 (0.03)	0.034 (0.026)	0 - 0.092	0.006 (0)	-196.52 (7.93)	0.158 (0.03)	0.04 - 0.199
SWG	50	16	0.50	0.30	0.503 (0.043)	0.041 (0.033)	0.034 (0.026)	0 - 0.114	0.006 (0)	-199.52 (9.81)	0.259 (0.033)	0.169 - 0.3
SWG	50	16	0.50	0.40	0.512 (0.042)	0.043 (0.036)	0.036 (0.024)	0.001 - 0.121	0.006 (0.001)	-197.92 (10.73)	0.357 (0.036)	0.215 - 0.396
SWG	50	16	0.50	0.50	0.504 (0.038)	0.045 (0.031)	0.029 (0.024)	0 - 0.107	0.006 (0)	-196.88 (10.11)	0.455 (0.031)	0.36 - 0.5
SWG	50	16	0.50	0.60	0.505 (0.037)	0.039 (0.033)	0.03 (0.023)	0 - 0.094	0.006 (0)	-197.5 (9.48)	0.561 (0.033)	0.452 - 0.6
SWG	50	16	0.50	0.70	0.504 (0.044)	0.041 (0.035)	0.034 (0.028)	0 - 0.166	0.006 (0)	-196.39 (8.86)	0.659 (0.035)	0.473 - 0.699

Continued on next page

Table S1 – *Continued from previous page*

Structure	T	N	p	p_e/p_w	$\bar{\hat{p}}$ (SD)	$\overline{\hat{p}_e}/\overline{\hat{p}_w}$ (SD)	$\overline{\lvert\Delta\rvert}_p$ (SD)	Range $\lvert\Delta\rvert_p$	$\overline{SE}\lvert\Delta\rvert_p$ (SD)	$\overline{BIC}$ (SD)	$\overline{\lvert\Delta\rvert}_{p_e/p_w}$ (SD)	Range $\lvert\Delta\rvert_{p_e/p_w}$
SWG	50	16	0.50	0.80	0.507 (0.041)	0.045 (0.031)	0.033 (0.025)	0 - 0.105	0.006 (0)	-195.97 (10.02)	0.755 (0.031)	0.665 - 0.799
SWG	50	16	0.50	0.90	0.508 (0.043)	0.039 (0.031)	0.035 (0.026)	0.002 - 0.123	0.006 (0)	-197.23 (9.92)	0.861 (0.031)	0.787 - 0.9
SWG	50	16	0.60	0.10	0.617 (0.042)	0.118 (0.052)	0.036 (0.027)	0 - 0.107	0.005 (0)	-197.44 (8.84)	0.041 (0.036)	0 - 0.152
SWG	50	16	0.60	0.20	0.615 (0.043)	0.104 (0.052)	0.036 (0.027)	0.001 - 0.106	0.005 (0)	-197.02 (8.48)	0.097 (0.049)	0 - 0.199
SWG	50	16	0.60	0.30	0.619 (0.044)	0.116 (0.051)	0.038 (0.028)	0 - 0.111	0.005 (0)	-197.28 (9.68)	0.184 (0.051)	0.014 - 0.296
SWG	50	16	0.60	0.40	0.625 (0.04)	0.127 (0.055)	0.038 (0.028)	0.001 - 0.122	0.005 (0)	-197.44 (10.12)	0.273 (0.055)	0.123 - 0.391
SWG	50	16	0.60	0.50	0.63 (0.042)	0.098 (0.05)	0.042 (0.03)	0 - 0.118	0.005 (0)	-196.69 (9.04)	0.402 (0.05)	0.257 - 0.5
SWG	50	16	0.60	0.60	0.632 (0.045)	0.11 (0.05)	0.046 (0.03)	0.001 - 0.13	0.005 (0)	-197.96 (10.31)	0.49 (0.05)	0.369 - 0.594
SWG	50	16	0.60	0.70	0.625 (0.041)	0.093 (0.055)	0.038 (0.029)	0.001 - 0.151	0.005 (0)	-196 (9.06)	0.607 (0.055)	0.467 - 0.697
SWG	50	16	0.60	0.80	0.63 (0.037)	0.105 (0.053)	0.038 (0.03)	0 - 0.118	0.005 (0)	-195.79 (8.58)	0.695 (0.053)	0.572 - 0.797
SWG	50	16	0.60	0.90	0.636 (0.04)	0.099 (0.049)	0.045 (0.03)	0.001 - 0.131	0.005 (0)	-195.54 (9.05)	0.801 (0.049)	0.674 - 0.898
SWG	50	16	0.70	0.10	0.726 (0.041)	0.221 (0.055)	0.041 (0.027)	0 - 0.11	0.005 (0)	-193.68 (8.79)	0.121 (0.054)	0.011 - 0.241
SWG	50	16	0.70	0.20	0.729 (0.042)	0.196 (0.05)	0.041 (0.029)	0.002 - 0.144	0.005 (0.001)	-193.57 (9.81)	0.04 (0.03)	0 - 0.165
SWG	50	16	0.70	0.30	0.731 (0.04)	0.219 (0.051)	0.044 (0.026)	0.002 - 0.102	0.005 (0)	-196.05 (10.14)	0.083 (0.048)	0.001 - 0.226
SWG	50	16	0.70	0.40	0.733 (0.04)	0.214 (0.047)	0.043 (0.029)	0 - 0.146	0.005 (0)	-193.62 (9.39)	0.186 (0.047)	0.057 - 0.315
SWG	50	16	0.70	0.50	0.738 (0.033)	0.151 (0.048)	0.043 (0.027)	0 - 0.121	0.004 (0)	-195.8 (11.26)	0.349 (0.048)	0.236 - 0.49
SWG	50	16	0.70	0.60	0.755 (0.032)	0.195 (0.041)	0.057 (0.028)	0.003 - 0.115	0.004 (0)	-195.41 (9.84)	0.405 (0.041)	0.272 - 0.491
SWG	50	16	0.70	0.70	0.736 (0.04)	0.166 (0.051)	0.045 (0.029)	0 - 0.122	0.004 (0.001)	-194.57 (10.65)	0.534 (0.051)	0.423 - 0.658

Continued on next page

Table S1 – *Continued from previous page*

Structure	T	N	p	p_e/p_w	$\overline{\hat{p}}$ (SD)	$\overline{\hat{p_e}}/\overline{\hat{p_w}}$ (SD)	$\overline{\lvert\Delta\rvert}_p$ (SD)	Range $\lvert\Delta\rvert_p$	$\overline{SE}\lvert\Delta\rvert_p$ (SD)	$\overline{BIC}$ (SD)	$\overline{\lvert\Delta\rvert}_{p_e/p_w}$ (SD)	Range $\lvert\Delta\rvert_{p_e/p_w}$
SWG	50	16	0.70	0.80	0.739 (0.035)	0.164 (0.052)	0.044 (0.029)	0 - 0.121	0.004 (0)	-195.85 (10.24)	0.636 (0.052)	0.495 - 0.741
SWG	50	16	0.70	0.90	0.746 (0.032)	0.175 (0.049)	0.049 (0.028)	0 - 0.114	0.004 (0)	-195.1 (9.23)	0.725 (0.049)	0.587 - 0.841
SWG	50	16	0.80	0.10	0.812 (0.037)	0.234 (0.052)	0.031 (0.023)	0 - 0.104	0.004 (0.001)	-190.11 (9.38)	0.134 (0.052)	0.032 - 0.297
SWG	50	16	0.80	0.20	0.824 (0.023)	0.221 (0.045)	0.028 (0.018)	0.003 - 0.081	0.004 (0)	-189.82 (9.51)	0.037 (0.033)	0 - 0.146
SWG	50	16	0.80	0.30	0.825 (0.035)	0.246 (0.05)	0.036 (0.023)	0 - 0.116	0.004 (0.001)	-191.97 (9.47)	0.06 (0.043)	0 - 0.171
SWG	50	16	0.80	0.40	0.826 (0.027)	0.235 (0.045)	0.031 (0.021)	0 - 0.078	0.003 (0)	-192 (10.4)	0.165 (0.045)	0.046 - 0.318
SWG	50	16	0.80	0.50	0.832 (0.025)	0.156 (0.05)	0.036 (0.018)	0 - 0.086	0.003 (0.001)	-185.34 (8.96)	0.344 (0.05)	0.17 - 0.476
SWG	50	16	0.80	0.60	0.846 (0.021)	0.172 (0.053)	0.046 (0.021)	0.003 - 0.095	0.003 (0)	-186.71 (10.36)	0.428 (0.053)	0.278 - 0.577
SWG	50	16	0.80	0.70	0.83 (0.024)	0.173 (0.055)	0.034 (0.019)	0.003 - 0.087	0.003 (0)	-191.68 (11.14)	0.527 (0.055)	0.391 - 0.675
SWG	50	16	0.80	0.80	0.832 (0.023)	0.154 (0.054)	0.034 (0.02)	0 - 0.081	0.003 (0)	-188.84 (11.08)	0.646 (0.054)	0.509 - 0.788
SWG	50	16	0.80	0.90	0.84 (0.02)	0.146 (0.05)	0.04 (0.02)	0 - 0.085	0.003 (0)	-188.77 (11.15)	0.754 (0.05)	0.616 - 0.859
SWG	50	16	0.90	0.10	0.895 (0.035)	0.119 (0.051)	0.023 (0.027)	0 - 0.179	0.002 (0.001)	-173.78 (16.24)	0.043 (0.032)	0 - 0.157
SWG	50	16	0.90	0.20	0.906 (0.026)	0.086 (0.044)	0.019 (0.018)	0 - 0.113	0.002 (0.001)	-161.74 (15.39)	0.116 (0.038)	0.01 - 0.198
SWG	50	16	0.90	0.30	0.912 (0.017)	0.126 (0.062)	0.018 (0.011)	0.001 - 0.049	0.002 (0)	-167.86 (19.29)	0.175 (0.06)	0.01 - 0.274
SWG	50	16	0.90	0.40	0.914 (0.025)	0.113 (0.056)	0.021 (0.02)	0 - 0.187	0.002 (0.001)	-165.72 (17.78)	0.287 (0.056)	0.069 - 0.397
SWG	50	16	0.90	0.50	0.912 (0.014)	0.06 (0.037)	0.015 (0.011)	0 - 0.044	0.002 (0)	-154.72 (12.05)	0.44 (0.037)	0.336 - 0.498
SWG	50	16	0.90	0.60	0.916 (0.013)	0.052 (0.034)	0.017 (0.011)	0 - 0.044	0.002 (0)	-151.87 (13.42)	0.548 (0.034)	0.454 - 0.599
SWG	50	16	0.90	0.70	0.911 (0.015)	0.052 (0.031)	0.016 (0.01)	0 - 0.054	0.002 (0)	-154.36 (13.4)	0.648 (0.031)	0.575 - 0.699

Continued on next page

Table S1 – *Continued from previous page*

Structure	T	N	p	p_e/p_w	$\overline{\hat{p}}$ (SD)	$\overline{\hat{p_e}}/\overline{\hat{p_w}}$ (SD)	$\overline{\lvert\Delta\rvert}_p$ (SD)	Range $\lvert\Delta\rvert_p$	$\overline{SE}\lvert\Delta\rvert_p$ (SD)	$\overline{BIC}$ (SD)	$\overline{\lvert\Delta\rvert}_{p_e/p_w}$ (SD)	Range $\lvert\Delta\rvert_{p_e/p_w}$
SWG	50	16	0.90	0.80	0.914 (0.012)	0.053 (0.033)	0.016 (0.01)	0 - 0.04	0.002 (0)	-152.95 (12.25)	0.747 (0.033)	0.584 - 0.8
SWG	50	16	0.90	0.90	0.915 (0.012)	0.037 (0.028)	0.016 (0.01)	0 - 0.04	0.002 (0)	-149.12 (13.53)	0.863 (0.028)	0.79 - 0.9
SWG	50	25	0.10	0.10	0.106 (0.014)	0.202 (0.139)	0.011 (0.01)	0 - 0.044	0.001 (0)	-173.04 (11.11)	0.118 (0.124)	0.003 - 0.558
SWG	50	25	0.10	0.20	0.109 (0.017)	0.245 (0.153)	0.015 (0.013)	0 - 0.061	0.001 (0)	-171.36 (10.25)	0.115 (0.109)	0.001 - 0.548
SWG	50	25	0.10	0.30	0.104 (0.015)	0.204 (0.129)	0.011 (0.01)	0 - 0.046	0.001 (0)	-170.76 (10.44)	0.144 (0.07)	0.005 - 0.351
SWG	50	25	0.10	0.40	0.104 (0.018)	0.223 (0.156)	0.014 (0.013)	0 - 0.074	0.001 (0)	-172.5 (10.77)	0.213 (0.1)	0.003 - 0.373
SWG	50	25	0.10	0.50	0.105 (0.016)	0.227 (0.17)	0.013 (0.011)	0 - 0.061	0.001 (0)	-169.69 (10.64)	0.291 (0.137)	0.008 - 0.48
SWG	50	25	0.10	0.60	0.103 (0.019)	0.194 (0.158)	0.014 (0.013)	0 - 0.06	0.001 (0)	-173.6 (10.14)	0.41 (0.147)	0.007 - 0.599
SWG	50	25	0.10	0.70	0.098 (0.022)	0.184 (0.15)	0.014 (0.017)	0 - 0.128	0.001 (0)	-172.84 (12.92)	0.516 (0.15)	0.144 - 0.697
SWG	50	25	0.10	0.80	0.096 (0.042)	0.211 (0.173)	0.025 (0.033)	0 - 0.188	0.001 (0)	-172.22 (10.54)	0.589 (0.173)	0.135 - 0.795
SWG	50	25	0.10	0.90	0.113 (0.085)	0.181 (0.155)	0.025 (0.082)	0.001 - 0.671	0.001 (0)	-175.1 (11.16)	0.719 (0.155)	0.021 - 0.892
SWG	50	25	0.20	0.10	0.208 (0.024)	0.236 (0.116)	0.019 (0.016)	0 - 0.067	0.002 (0)	-201.66 (9.63)	0.139 (0.113)	0.001 - 0.488
SWG	50	25	0.20	0.20	0.207 (0.03)	0.256 (0.143)	0.024 (0.019)	0 - 0.092	0.002 (0)	-203.7 (11.07)	0.111 (0.105)	0.001 - 0.566
SWG	50	25	0.20	0.30	0.212 (0.026)	0.274 (0.131)	0.023 (0.018)	0 - 0.084	0.002 (0)	-202 (11.16)	0.113 (0.07)	0.003 - 0.36
SWG	50	25	0.20	0.40	0.208 (0.031)	0.274 (0.132)	0.024 (0.022)	0 - 0.101	0.002 (0)	-202.01 (12.07)	0.162 (0.083)	0.002 - 0.315
SWG	50	25	0.20	0.50	0.21 (0.029)	0.256 (0.136)	0.024 (0.019)	0 - 0.087	0.002 (0)	-201.98 (11.47)	0.253 (0.117)	0.02 - 0.448
SWG	50	25	0.20	0.60	0.212 (0.033)	0.294 (0.169)	0.027 (0.023)	0 - 0.089	0.002 (0)	-201.57 (12.12)	0.314 (0.154)	0.018 - 0.59
SWG	50	25	0.20	0.70	0.204 (0.031)	0.261 (0.148)	0.025 (0.019)	0.001 - 0.095	0.002 (0)	-200.03 (11.21)	0.439 (0.146)	0.04 - 0.668

Continued on next page

Table S1 – *Continued from previous page*

Structure	T	N	p	p_e/p_w	$\overline{\hat{p}}$ (SD)	$\overline{\hat{p_e}}/\overline{\hat{p_w}}$ (SD)	$\overline{\lvert\Delta\rvert}_p$ (SD)	Range $\lvert\Delta\rvert_p$	$\overline{SE}\lvert\Delta\rvert_p$ (SD)	$\overline{BIC}$ (SD)	$\overline{\lvert\Delta\rvert}_{p_e/p_w}$ (SD)	Range $\lvert\Delta\rvert_{p_e/p_w}$
SWG	50	25	0.20	0.80	0.206 (0.032)	0.24 (0.147)	0.025 (0.021)	0 - 0.082	0.002 (0)	-202.24 (10.13)	0.56 (0.147)	0.174 - 0.773
SWG	50	25	0.20	0.90	0.21 (0.031)	0.247 (0.149)	0.024 (0.022)	0 - 0.117	0.002 (0)	-201.1 (11.04)	0.653 (0.149)	0.162 - 0.874
SWG	50	25	0.30	0.10	0.308 (0.032)	0.197 (0.072)	0.026 (0.02)	0 - 0.101	0.003 (0)	-213.74 (10.57)	0.099 (0.07)	0.001 - 0.323
SWG	50	25	0.30	0.20	0.309 (0.033)	0.225 (0.07)	0.027 (0.021)	0 - 0.13	0.003 (0)	-215.27 (9.89)	0.058 (0.046)	0.002 - 0.228
SWG	50	25	0.30	0.30	0.308 (0.033)	0.22 (0.08)	0.027 (0.019)	0.001 - 0.095	0.003 (0)	-213.84 (11.21)	0.1 (0.053)	0.001 - 0.187
SWG	50	25	0.30	0.40	0.307 (0.032)	0.212 (0.077)	0.025 (0.02)	0 - 0.11	0.003 (0)	-213.73 (9.88)	0.189 (0.075)	0.011 - 0.334
SWG	50	25	0.30	0.50	0.302 (0.035)	0.204 (0.085)	0.028 (0.021)	0 - 0.107	0.003 (0)	-212.89 (9.59)	0.296 (0.085)	0.043 - 0.445
SWG	50	25	0.30	0.60	0.304 (0.031)	0.223 (0.078)	0.026 (0.017)	0 - 0.097	0.003 (0)	-215.29 (10.38)	0.377 (0.078)	0.161 - 0.527
SWG	50	25	0.30	0.70	0.306 (0.032)	0.217 (0.082)	0.026 (0.019)	0.002 - 0.103	0.003 (0)	-215.98 (10.52)	0.483 (0.082)	0.218 - 0.636
SWG	50	25	0.30	0.80	0.291 (0.026)	0.163 (0.063)	0.022 (0.016)	0 - 0.068	0.003 (0)	-214.42 (10.97)	0.637 (0.063)	0.456 - 0.746
SWG	50	25	0.30	0.90	0.305 (0.031)	0.196 (0.083)	0.024 (0.019)	0 - 0.088	0.003 (0)	-213.04 (10.07)	0.704 (0.083)	0.346 - 0.846
SWG	50	25	0.40	0.10	0.398 (0.037)	0.091 (0.04)	0.029 (0.023)	0 - 0.101	0.005 (0)	-218.35 (10.11)	0.034 (0.023)	0 - 0.114
SWG	50	25	0.40	0.20	0.404 (0.036)	0.12 (0.042)	0.028 (0.023)	0 - 0.1	0.005 (0)	-216.77 (8.75)	0.081 (0.041)	0.001 - 0.164
SWG	50	25	0.40	0.30	0.398 (0.034)	0.111 (0.042)	0.028 (0.02)	0 - 0.085	0.005 (0)	-217.57 (9.52)	0.189 (0.042)	0.095 - 0.287
SWG	50	25	0.40	0.40	0.4 (0.034)	0.113 (0.049)	0.028 (0.019)	0 - 0.085	0.005 (0)	-219.05 (8.7)	0.287 (0.049)	0.148 - 0.397
SWG	50	25	0.40	0.50	0.39 (0.031)	0.101 (0.04)	0.027 (0.019)	0.001 - 0.085	0.005 (0)	-218.75 (8.52)	0.399 (0.04)	0.261 - 0.499
SWG	50	25	0.40	0.60	0.398 (0.04)	0.118 (0.043)	0.032 (0.024)	0 - 0.116	0.005 (0)	-217.24 (8.49)	0.482 (0.043)	0.363 - 0.558
SWG	50	25	0.40	0.70	0.389 (0.035)	0.106 (0.042)	0.03 (0.022)	0 - 0.112	0.005 (0)	-218.84 (9.92)	0.594 (0.042)	0.478 - 0.694
SWG	50	25	0.40	0.80	0.398 (0.038)	0.101 (0.043)	0.031 (0.023)	0 - 0.103	0.005 (0)	-218.21 (9.45)	0.699 (0.043)	0.573 - 0.783
SWG	50	25	0.40	0.90	0.395 (0.034)	0.086 (0.045)	0.027 (0.021)	0.002 - 0.093	0.005 (0)	-215.8 (8.77)	0.814 (0.045)	0.684 - 0.898

Continued on next page

Table S1 – *Continued from previous page*

Structure	T	N	p	p_e/p_w	$\overline{\hat{p}}$ (SD)	$\overline{\hat{p_e}/\hat{p_w}}$ (SD)	$\overline{\lvert\Delta\rvert}_p$ (SD)	Range $\lvert\Delta\rvert_p$	$\overline{SE}\lvert\Delta\rvert_p$ (SD)	$\overline{BIC}$ (SD)	$\overline{\lvert\Delta\rvert}_{p_e/p_w}$ (SD)	Range $\lvert\Delta\rvert_{p_e/p_w}$
SWG	50	25	0.50	0.10	0.508 (0.037)	0.026 (0.02)	0.03 (0.022)	0 - 0.106	0.005 (0)	-219.22 (8.47)	0.074 (0.02)	0.01 - 0.1
SWG	50	25	0.50	0.20	0.504 (0.04)	0.031 (0.025)	0.031 (0.025)	0.002 - 0.123	0.005 (0)	-219.5 (9.69)	0.169 (0.025)	0.095 - 0.2
SWG	50	25	0.50	0.30	0.503 (0.039)	0.024 (0.022)	0.031 (0.024)	0 - 0.11	0.006 (0)	-217.7 (9.38)	0.276 (0.022)	0.189 - 0.3
SWG	50	25	0.50	0.40	0.509 (0.04)	0.029 (0.021)	0.032 (0.025)	0 - 0.128	0.005 (0)	-218.65 (8.46)	0.371 (0.021)	0.311 - 0.399
SWG	50	25	0.50	0.50	0.5 (0.042)	0.03 (0.022)	0.036 (0.022)	0.001 - 0.102	0.005 (0)	-219.87 (10.68)	0.47 (0.022)	0.401 - 0.5
SWG	50	25	0.50	0.60	0.5 (0.037)	0.028 (0.021)	0.028 (0.023)	0 - 0.109	0.005 (0)	-219.49 (10.68)	0.572 (0.021)	0.495 - 0.6
SWG	50	25	0.50	0.70	0.505 (0.037)	0.027 (0.023)	0.03 (0.022)	0 - 0.094	0.005 (0)	-218.01 (8.99)	0.673 (0.023)	0.606 - 0.699
SWG	50	25	0.50	0.80	0.505 (0.039)	0.027 (0.022)	0.031 (0.024)	0 - 0.119	0.005 (0)	-221.14 (10.4)	0.773 (0.022)	0.689 - 0.8
SWG	50	25	0.50	0.90	0.508 (0.039)	0.028 (0.023)	0.031 (0.024)	0.001 - 0.123	0.005 (0)	-220.41 (9.86)	0.872 (0.023)	0.789 - 0.9
SWG	50	25	0.60	0.10	0.615 (0.042)	0.096 (0.035)	0.036 (0.026)	0.001 - 0.125	0.005 (0)	-216.78 (9.01)	0.027 (0.021)	0.001 - 0.101
SWG	50	25	0.60	0.20	0.617 (0.042)	0.104 (0.038)	0.038 (0.024)	0 - 0.094	0.005 (0)	-216.95 (9.36)	0.096 (0.038)	0.006 - 0.18
SWG	50	25	0.60	0.30	0.619 (0.041)	0.104 (0.038)	0.036 (0.027)	0 - 0.103	0.005 (0)	-218.81 (10.36)	0.196 (0.038)	0.091 - 0.282
SWG	50	25	0.60	0.40	0.622 (0.039)	0.108 (0.035)	0.036 (0.028)	0.001 - 0.107	0.005 (0)	-219.33 (9.72)	0.292 (0.035)	0.209 - 0.368
SWG	50	25	0.60	0.50	0.628 (0.042)	0.103 (0.036)	0.042 (0.029)	0.001 - 0.125	0.005 (0)	-218.45 (9.82)	0.397 (0.036)	0.271 - 0.48
SWG	50	25	0.60	0.60	0.638 (0.035)	0.109 (0.031)	0.043 (0.028)	0 - 0.113	0.005 (0)	-218.63 (9.28)	0.491 (0.031)	0.407 - 0.552
SWG	50	25	0.60	0.70	0.631 (0.038)	0.096 (0.038)	0.039 (0.031)	0.001 - 0.111	0.005 (0)	-216.68 (7.75)	0.604 (0.038)	0.516 - 0.691
SWG	50	25	0.60	0.80	0.625 (0.036)	0.081 (0.03)	0.036 (0.025)	0 - 0.117	0.005 (0)	-217.97 (8.95)	0.719 (0.03)	0.642 - 0.779
SWG	50	25	0.60	0.90	0.625 (0.042)	0.081 (0.032)	0.041 (0.027)	0 - 0.129	0.005 (0)	-219.19 (9.96)	0.819 (0.032)	0.741 - 0.896
SWG	50	25	0.70	0.10	0.717 (0.04)	0.17 (0.035)	0.036 (0.024)	0.001 - 0.096	0.004 (0)	-215.24 (9.44)	0.071 (0.035)	0.002 - 0.166

Continued on next page

Table S1 – *Continued from previous page*

Structure	T	N	p	p_e/p_w	$\overline{\hat{p}}$ (SD)	$\overline{\hat{p_e}}/\overline{\hat{p_w}}$ (SD)	$\overline{\lvert\Delta\rvert}_p$ (SD)	Range $\lvert\Delta\rvert_p$	$\overline{SE}\lvert\Delta\rvert_p$ (SD)	$\overline{BIC}$ (SD)	$\overline{\lvert\Delta\rvert}_{p_e/p_w}$ (SD)	Range $\lvert\Delta\rvert_{p_e/p_w}$
SWG	50	25	0.70	0.20	0.731 (0.034)	0.198 (0.03)	0.037 (0.026)	0 - 0.103	0.004 (0)	-218.6 (11.04)	0.024 (0.017)	0.001 - 0.072
SWG	50	25	0.70	0.30	0.733 (0.034)	0.197 (0.033)	0.04 (0.024)	0.002 - 0.104	0.004 (0)	-217.83 (9.5)	0.103 (0.033)	0.022 - 0.166
SWG	50	25	0.70	0.40	0.74 (0.032)	0.198 (0.038)	0.045 (0.024)	0 - 0.097	0.004 (0)	-215.21 (9.62)	0.202 (0.038)	0.089 - 0.296
SWG	50	25	0.70	0.50	0.738 (0.033)	0.177 (0.037)	0.042 (0.027)	0.001 - 0.103	0.004 (0)	-215.61 (10.4)	0.323 (0.037)	0.214 - 0.397
SWG	50	25	0.70	0.60	0.748 (0.029)	0.188 (0.039)	0.049 (0.026)	0 - 0.115	0.004 (0)	-217.71 (10.05)	0.412 (0.039)	0.297 - 0.489
SWG	50	25	0.70	0.70	0.745 (0.032)	0.181 (0.037)	0.048 (0.027)	0.001 - 0.131	0.004 (0)	-217.4 (9.61)	0.519 (0.037)	0.389 - 0.609
SWG	50	25	0.70	0.80	0.743 (0.034)	0.151 (0.034)	0.048 (0.026)	0 - 0.113	0.004 (0)	-217.5 (10.79)	0.649 (0.034)	0.561 - 0.723
SWG	50	25	0.70	0.90	0.746 (0.032)	0.147 (0.038)	0.049 (0.026)	0.003 - 0.117	0.004 (0)	-216.36 (9.65)	0.753 (0.038)	0.653 - 0.846
SWG	50	25	0.80	0.10	0.817 (0.026)	0.202 (0.033)	0.026 (0.017)	0.001 - 0.078	0.003 (0.001)	-208.42 (8.57)	0.102 (0.033)	0.003 - 0.195
SWG	50	25	0.80	0.20	0.83 (0.024)	0.23 (0.034)	0.034 (0.018)	0.002 - 0.083	0.003 (0)	-214.18 (11.05)	0.036 (0.027)	0.001 - 0.113
SWG	50	25	0.80	0.30	0.826 (0.028)	0.225 (0.036)	0.032 (0.02)	0 - 0.111	0.003 (0)	-215.37 (10.64)	0.076 (0.035)	0.002 - 0.185
SWG	50	25	0.80	0.40	0.835 (0.023)	0.208 (0.038)	0.037 (0.018)	0.001 - 0.075	0.003 (0)	-215.84 (11.19)	0.192 (0.038)	0.11 - 0.277
SWG	50	25	0.80	0.50	0.837 (0.022)	0.185 (0.038)	0.038 (0.02)	0 - 0.083	0.003 (0)	-211.13 (10.56)	0.315 (0.038)	0.215 - 0.402
SWG	50	25	0.80	0.60	0.84 (0.02)	0.185 (0.042)	0.042 (0.017)	0.002 - 0.082	0.003 (0)	-215.14 (12.98)	0.415 (0.042)	0.322 - 0.531
SWG	50	25	0.80	0.70	0.839 (0.021)	0.165 (0.035)	0.04 (0.018)	0.001 - 0.08	0.003 (0)	-214.43 (12.3)	0.535 (0.035)	0.458 - 0.613
SWG	50	25	0.80	0.80	0.839 (0.018)	0.133 (0.038)	0.04 (0.016)	0.003 - 0.076	0.002 (0)	-211.98 (11.41)	0.667 (0.038)	0.572 - 0.744
SWG	50	25	0.80	0.90	0.831 (0.016)	0.146 (0.033)	0.032 (0.015)	0.001 - 0.064	0.003 (0)	-214.37 (10.27)	0.754 (0.033)	0.682 - 0.829
SWG	50	25	0.90	0.10	0.905 (0.015)	0.085 (0.043)	0.013 (0.008)	0 - 0.039	0.002 (0)	-187.69 (15.86)	0.037 (0.026)	0 - 0.12

Continued on next page

Table S1 – *Continued from previous page*

Structure	T	N	p	p_e/p_w	$\overline{\hat{p}}$ (SD)	$\overline{\hat{p}_e}/\overline{\hat{p}_w}$ (SD)	$\overline{\lvert\Delta\rvert}_p$ (SD)	Range $\lvert\Delta\rvert_p$	$\overline{SE}\lvert\Delta\rvert_p$ (SD)	$\overline{BIC}$ (SD)	$\overline{\lvert\Delta\rvert}_{p_e/p_w}$ (SD)	Range $\lvert\Delta\rvert_{p_e/p_w}$
SWG	50	25	0.90	0.20	0.912 (0.011)	0.087 (0.038)	0.014 (0.008)	0 - 0.037	0.001 (0)	-189.21 (17.47)	0.114 (0.035)	0.012 - 0.175
SWG	50	25	0.90	0.30	0.911 (0.012)	0.084 (0.043)	0.013 (0.009)	0 - 0.034	0.001 (0)	-192.14 (19.63)	0.216 (0.043)	0.098 - 0.28
SWG	50	25	0.90	0.40	0.913 (0.011)	0.063 (0.027)	0.014 (0.009)	0 - 0.041	0.001 (0)	-182.69 (17.08)	0.337 (0.027)	0.24 - 0.399
SWG	50	25	0.90	0.50	0.912 (0.011)	0.05 (0.025)	0.014 (0.01)	0 - 0.039	0.001 (0)	-179 (14.07)	0.45 (0.025)	0.379 - 0.499
SWG	50	25	0.90	0.60	0.914 (0.011)	0.045 (0.021)	0.016 (0.009)	0 - 0.036	0.001 (0)	-177.72 (12.2)	0.555 (0.021)	0.49 - 0.598
SWG	50	25	0.90	0.70	0.914 (0.01)	0.039 (0.02)	0.015 (0.009)	0 - 0.044	0.001 (0)	-175.47 (12.38)	0.661 (0.02)	0.605 - 0.7
SWG	50	25	0.90	0.80	0.91 (0.01)	0.032 (0.019)	0.012 (0.008)	0 - 0.038	0.001 (0)	-175.96 (11.55)	0.768 (0.019)	0.717 - 0.798
SWG	50	25	0.90	0.90	0.909 (0.012)	0.047 (0.024)	0.012 (0.009)	0 - 0.048	0.001 (0)	-177.65 (11.97)	0.853 (0.024)	0.778 - 0.9
SWG	50	49	0.10	0.10	0.106 (0.013)	0.157 (0.103)	0.01 (0.01)	0 - 0.054	0.001 (0)	-209.11 (13.11)	0.081 (0.085)	0 - 0.432
SWG	50	49	0.10	0.20	0.107 (0.013)	0.166 (0.094)	0.011 (0.01)	0 - 0.036	0.001 (0)	-207.73 (11)	0.087 (0.05)	0.001 - 0.296
SWG	50	49	0.10	0.30	0.108 (0.014)	0.172 (0.118)	0.011 (0.011)	0 - 0.056	0.001 (0)	-211.46 (12.84)	0.159 (0.071)	0 - 0.275
SWG	50	49	0.10	0.40	0.105 (0.013)	0.162 (0.121)	0.011 (0.01)	0 - 0.052	0.001 (0)	-209.18 (11.73)	0.249 (0.094)	0 - 0.398
SWG	50	49	0.10	0.50	0.105 (0.014)	0.158 (0.122)	0.011 (0.01)	0 - 0.049	0.001 (0)	-209.85 (13.08)	0.343 (0.12)	0.001 - 0.492
SWG	50	49	0.10	0.60	0.104 (0.014)	0.152 (0.114)	0.01 (0.01)	0.001 - 0.049	0.001 (0)	-209.64 (12.29)	0.448 (0.114)	0.067 - 0.591
SWG	50	49	0.10	0.70	0.106 (0.015)	0.173 (0.115)	0.012 (0.01)	0.001 - 0.054	0.001 (0)	-211.33 (12.62)	0.527 (0.115)	0.092 - 0.665
SWG	50	49	0.10	0.80	0.112 (0.06)	0.164 (0.13)	0.025 (0.056)	0 - 0.396	0.001 (0)	-209.39 (13.87)	0.636 (0.13)	0.27 - 0.785
SWG	50	49	0.10	0.90	0.139 (0.126)	0.199 (0.146)	0.054 (0.12)	0 - 0.715	0.001 (0)	-212.39 (13.85)	0.701 (0.146)	0.068 - 0.865
SWG	50	49	0.20	0.10	0.212 (0.025)	0.213 (0.102)	0.021 (0.018)	0 - 0.078	0.001 (0)	-237.39 (11.27)	0.117 (0.098)	0.001 - 0.384
SWG	50	49	0.20	0.20	0.208 (0.026)	0.207 (0.118)	0.02 (0.018)	0 - 0.086	0.001 (0)	-237.36 (12.15)	0.094 (0.072)	0.001 - 0.345

Continued on next page

Table S1 – *Continued from previous page*

Structure	T	N	p	p_e/p_w	$\bar{\hat{p}}$ (SD)	$\overline{\hat{p}_e}/\overline{\hat{p}_w}$ (SD)	$\overline{\lvert\Delta\rvert}_p$ (SD)	Range $\lvert\Delta\rvert_p$	$\overline{SE}\lvert\Delta\rvert_p$ (SD)	$\overline{BIC}$ (SD)	$\overline{\lvert\Delta\rvert}_{p_e/p_w}$ (SD)	Range $\lvert\Delta\rvert_{p_e/p_w}$
SWG	50	49	0.20	0.30	0.206 (0.027)	0.203 (0.121)	0.021 (0.019)	0 - 0.085	0.001 (0)	-235.9 (10.12)	0.142 (0.062)	0.002 - 0.248
SWG	50	49	0.20	0.40	0.208 (0.028)	0.215 (0.123)	0.021 (0.02)	0 - 0.086	0.001 (0)	-236.29 (11.31)	0.201 (0.096)	0.002 - 0.337
SWG	50	49	0.20	0.50	0.209 (0.026)	0.226 (0.125)	0.022 (0.017)	0 - 0.088	0.001 (0)	-235.44 (10.25)	0.276 (0.119)	0.004 - 0.45
SWG	50	49	0.20	0.60	0.208 (0.027)	0.218 (0.116)	0.022 (0.017)	0.001 - 0.072	0.001 (0)	-234.39 (10.12)	0.382 (0.116)	0.045 - 0.538
SWG	50	49	0.20	0.70	0.208 (0.029)	0.22 (0.13)	0.024 (0.019)	0 - 0.073	0.001 (0)	-235.12 (11.06)	0.48 (0.13)	0.148 - 0.682
SWG	50	49	0.20	0.80	0.206 (0.031)	0.208 (0.136)	0.026 (0.018)	0 - 0.084	0.001 (0)	-236.26 (10.32)	0.592 (0.136)	0.232 - 0.772
SWG	50	49	0.20	0.90	0.211 (0.028)	0.246 (0.132)	0.024 (0.018)	0.001 - 0.077	0.001 (0)	-235.66 (11.35)	0.654 (0.132)	0.349 - 0.861
SWG	50	49	0.30	0.10	0.309 (0.035)	0.182 (0.068)	0.029 (0.022)	0 - 0.097	0.002 (0)	-248.81 (11.33)	0.084 (0.065)	0.001 - 0.295
SWG	50	49	0.30	0.20	0.306 (0.03)	0.181 (0.059)	0.026 (0.017)	0 - 0.082	0.002 (0)	-248.25 (9.44)	0.053 (0.032)	0 - 0.141
SWG	50	49	0.30	0.30	0.302 (0.031)	0.177 (0.062)	0.025 (0.018)	0.001 - 0.078	0.002 (0)	-249.12 (9.34)	0.127 (0.053)	0.001 - 0.226
SWG	50	49	0.30	0.40	0.303 (0.029)	0.191 (0.069)	0.024 (0.017)	0.001 - 0.085	0.002 (0)	-247.96 (9.76)	0.209 (0.067)	0.004 - 0.324
SWG	50	49	0.30	0.50	0.301 (0.03)	0.175 (0.06)	0.025 (0.017)	0 - 0.088	0.002 (0)	-248.17 (10.81)	0.325 (0.06)	0.116 - 0.427
SWG	50	49	0.30	0.60	0.296 (0.031)	0.168 (0.066)	0.027 (0.017)	0 - 0.094	0.002 (0)	-246.74 (9.23)	0.432 (0.066)	0.263 - 0.53
SWG	50	49	0.30	0.70	0.297 (0.031)	0.172 (0.065)	0.025 (0.019)	0 - 0.105	0.002 (0)	-248.02 (10.31)	0.528 (0.065)	0.32 - 0.634
SWG	50	49	0.30	0.80	0.289 (0.03)	0.158 (0.062)	0.027 (0.017)	0 - 0.081	0.002 (0)	-248.15 (10.07)	0.642 (0.062)	0.415 - 0.739
SWG	50	49	0.30	0.90	0.294 (0.029)	0.173 (0.062)	0.025 (0.016)	0 - 0.076	0.002 (0)	-245.63 (9.71)	0.727 (0.062)	0.518 - 0.837
SWG	50	49	0.40	0.10	0.405 (0.031)	0.099 (0.033)	0.026 (0.018)	0.001 - 0.073	0.004 (0)	-251.7 (10.21)	0.027 (0.019)	0 - 0.071
SWG	50	49	0.40	0.20	0.396 (0.031)	0.092 (0.028)	0.025 (0.02)	0 - 0.125	0.004 (0)	-251.32 (8.97)	0.108 (0.028)	0.023 - 0.161

Continued on next page

Table S1 – *Continued from previous page*

Structure	T	N	p	p_e/p_w	$\overline{\hat{p}}$ (SD)	$\overline{\hat{p_e}}/\overline{\hat{p_w}}$ (SD)	$\overline{\lvert\Delta\rvert}_p$ (SD)	Range $\lvert\Delta\rvert_p$	$\overline{SE}\lvert\Delta\rvert_p$ (SD)	$\overline{BIC}$ (SD)	$\overline{\lvert\Delta\rvert}_{p_e/p_w}$ (SD)	Range $\lvert\Delta\rvert_{p_e/p_w}$
SWG	50	49	0.40	0.30	0.396 (0.03)	0.094 (0.029)	0.024 (0.018)	0 - 0.072	0.004 (0)	-252.18 (8.45)	0.206 (0.029)	0.116 - 0.272
SWG	50	49	0.40	0.40	0.396 (0.032)	0.094 (0.029)	0.026 (0.018)	0 - 0.076	0.004 (0)	-250.83 (9.17)	0.306 (0.029)	0.218 - 0.364
SWG	50	49	0.40	0.50	0.391 (0.034)	0.093 (0.028)	0.028 (0.02)	0 - 0.093	0.004 (0)	-250.33 (10.24)	0.407 (0.028)	0.31 - 0.459
SWG	50	49	0.40	0.60	0.393 (0.03)	0.085 (0.026)	0.026 (0.017)	0 - 0.067	0.004 (0)	-251.08 (9.85)	0.515 (0.026)	0.419 - 0.566
SWG	50	49	0.40	0.70	0.391 (0.029)	0.093 (0.031)	0.025 (0.018)	0 - 0.072	0.004 (0)	-252.83 (8.6)	0.607 (0.031)	0.495 - 0.684
SWG	50	49	0.40	0.80	0.388 (0.028)	0.083 (0.025)	0.026 (0.017)	0 - 0.078	0.004 (0)	-250.3 (8.98)	0.717 (0.025)	0.637 - 0.772
SWG	50	49	0.40	0.90	0.388 (0.028)	0.091 (0.03)	0.024 (0.018)	0.001 - 0.077	0.004 (0)	-253.01 (10.3)	0.809 (0.03)	0.711 - 0.869
SWG	50	49	0.50	0.10	0.506 (0.037)	0.017 (0.012)	0.03 (0.021)	0.001 - 0.093	0.005 (0)	-251.69 (9.2)	0.083 (0.012)	0.047 - 0.1
SWG	50	49	0.50	0.20	0.511 (0.035)	0.02 (0.015)	0.03 (0.021)	0.001 - 0.085	0.005 (0)	-250.23 (8.84)	0.18 (0.015)	0.132 - 0.2
SWG	50	49	0.50	0.30	0.509 (0.039)	0.016 (0.013)	0.033 (0.022)	0.001 - 0.097	0.005 (0)	-251.33 (9.4)	0.284 (0.013)	0.242 - 0.3
SWG	50	49	0.50	0.40	0.501 (0.038)	0.017 (0.014)	0.03 (0.023)	0 - 0.087	0.005 (0)	-252.12 (8.97)	0.383 (0.014)	0.34 - 0.4
SWG	50	49	0.50	0.50	0.508 (0.038)	0.018 (0.013)	0.031 (0.022)	0.001 - 0.092	0.005 (0)	-251.01 (10.38)	0.482 (0.013)	0.439 - 0.5
SWG	50	49	0.50	0.60	0.508 (0.033)	0.019 (0.015)	0.027 (0.02)	0 - 0.094	0.005 (0)	-251.75 (9.3)	0.581 (0.015)	0.523 - 0.6
SWG	50	49	0.50	0.70	0.505 (0.037)	0.02 (0.014)	0.029 (0.023)	0 - 0.124	0.005 (0)	-250.51 (9.67)	0.68 (0.014)	0.633 - 0.699
SWG	50	49	0.50	0.80	0.508 (0.035)	0.018 (0.014)	0.03 (0.02)	0.001 - 0.1	0.005 (0)	-252.95 (9.7)	0.782 (0.014)	0.745 - 0.8
SWG	50	49	0.50	0.90	0.511 (0.039)	0.018 (0.014)	0.034 (0.023)	0.001 - 0.125	0.005 (0)	-252.08 (10.59)	0.882 (0.014)	0.838 - 0.9
SWG	50	49	0.60	0.10	0.615 (0.035)	0.096 (0.024)	0.03 (0.023)	0 - 0.135	0.004 (0)	-250.87 (9.58)	0.018 (0.015)	0 - 0.066
SWG	50	49	0.60	0.20	0.623 (0.035)	0.103 (0.025)	0.034 (0.024)	0 - 0.119	0.004 (0)	-252.03 (9.31)	0.097 (0.025)	0.035 - 0.16
SWG	50	49	0.60	0.30	0.624 (0.03)	0.094 (0.026)	0.031 (0.023)	0.001 - 0.122	0.004 (0)	-252.91 (10.22)	0.206 (0.026)	0.129 - 0.267

Continued on next page

Table S1 – *Continued from previous page*

Structure	T	N	p	p_e/p_w	$\overline{\hat{p}}$ (SD)	$\overline{\hat{p_e}}/\overline{\hat{p_w}}$ (SD)	$\overline{\|\Delta\|}_p$ (SD)	Range $\|\Delta\|_p$	$\overline{SE}\|\Delta\|_p$ (SD)	$\overline{BIC}$ (SD)	$\overline{\|\Delta\|}_{p_e/p_w}$ (SD)	Range $\|\Delta\|_{p_e/p_w}$
SWG	50	49	0.60	0.40	0.615 (0.036)	0.089 (0.021)	0.031 (0.023)	0 - 0.109	0.004 (0)	-251.4 (9.75)	0.311 (0.021)	0.263 - 0.38
SWG	50	49	0.60	0.50	0.626 (0.035)	0.091 (0.028)	0.036 (0.025)	0.001 - 0.138	0.004 (0)	-253.54 (10.12)	0.409 (0.028)	0.351 - 0.484
SWG	50	49	0.60	0.60	0.623 (0.034)	0.089 (0.027)	0.034 (0.024)	0.002 - 0.106	0.004 (0)	-252.43 (9.61)	0.511 (0.027)	0.417 - 0.566
SWG	50	49	0.60	0.70	0.631 (0.034)	0.091 (0.022)	0.039 (0.024)	0 - 0.091	0.005 (0)	-249.24 (8.29)	0.609 (0.022)	0.553 - 0.663
SWG	50	49	0.60	0.80	0.626 (0.033)	0.081 (0.024)	0.035 (0.023)	0 - 0.095	0.004 (0)	-252.42 (10.67)	0.719 (0.024)	0.652 - 0.768
SWG	50	49	0.60	0.90	0.633 (0.037)	0.09 (0.025)	0.039 (0.029)	0.001 - 0.156	0.004 (0)	-251.03 (10.67)	0.81 (0.025)	0.733 - 0.875
SWG	50	49	0.70	0.10	0.722 (0.032)	0.176 (0.024)	0.033 (0.021)	0 - 0.088	0.004 (0)	-250.2 (9.95)	0.077 (0.024)	0.009 - 0.14
SWG	50	49	0.70	0.20	0.725 (0.034)	0.179 (0.025)	0.034 (0.025)	0 - 0.102	0.004 (0)	-247.77 (7.77)	0.027 (0.018)	0.001 - 0.073
SWG	50	49	0.70	0.30	0.724 (0.034)	0.175 (0.022)	0.035 (0.023)	0.001 - 0.108	0.004 (0)	-251.2 (9.83)	0.125 (0.022)	0.081 - 0.174
SWG	50	49	0.70	0.40	0.738 (0.03)	0.183 (0.022)	0.042 (0.024)	0.001 - 0.111	0.004 (0)	-250.2 (10.6)	0.217 (0.022)	0.16 - 0.275
SWG	50	49	0.70	0.50	0.738 (0.029)	0.169 (0.022)	0.042 (0.022)	0 - 0.089	0.004 (0)	-250.51 (10.02)	0.331 (0.022)	0.285 - 0.387
SWG	50	49	0.70	0.60	0.739 (0.027)	0.17 (0.023)	0.042 (0.023)	0.001 - 0.091	0.003 (0)	-251.5 (10.19)	0.43 (0.023)	0.37 - 0.486
SWG	50	49	0.70	0.70	0.747 (0.028)	0.169 (0.023)	0.048 (0.026)	0.001 - 0.109	0.003 (0)	-250.63 (10.38)	0.531 (0.023)	0.478 - 0.598
SWG	50	49	0.70	0.80	0.745 (0.025)	0.146 (0.021)	0.046 (0.024)	0 - 0.105	0.003 (0)	-251.57 (10.85)	0.654 (0.021)	0.59 - 0.703
SWG	50	49	0.70	0.90	0.751 (0.024)	0.152 (0.018)	0.052 (0.023)	0.005 - 0.114	0.003 (0)	-250.61 (10.88)	0.748 (0.018)	0.713 - 0.802
SWG	50	49	0.80	0.10	0.813 (0.024)	0.213 (0.022)	0.022 (0.015)	0 - 0.062	0.003 (0)	-248.67 (10.73)	0.113 (0.022)	0.05 - 0.18
SWG	50	49	0.80	0.20	0.815 (0.021)	0.217 (0.022)	0.022 (0.014)	0 - 0.07	0.003 (0)	-250.55 (11.58)	0.023 (0.016)	0 - 0.073
SWG	50	49	0.80	0.30	0.823 (0.022)	0.209 (0.025)	0.027 (0.016)	0 - 0.067	0.002 (0)	-252.37 (12.92)	0.091 (0.025)	0.034 - 0.143

Continued on next page

Table S1 – *Continued from previous page*

Structure	T	N	p	p_e/p_w	$\overline{\hat{p}}$ (SD)	$\overline{\hat{p_e}/\hat{p_w}}$ (SD)	$\overline{\lvert\Delta\rvert}_p$ (SD)	Range $\lvert\Delta\rvert_p$	$\overline{SE}\lvert\Delta\rvert_p$ (SD)	$\overline{BIC}$ (SD)	$\overline{\lvert\Delta\rvert}_{p_e/p_w}$ (SD)	Range $\lvert\Delta\rvert_{p_e/p_w}$
SWG	50	49	0.80	0.40	0.833 (0.017)	0.203 (0.023)	0.034 (0.015)	0.002 - 0.063	0.002 (0)	-252.26 (11.51)	0.197 (0.023)	0.143 - 0.25
SWG	50	49	0.80	0.50	0.833 (0.017)	0.185 (0.025)	0.034 (0.015)	0 - 0.07	0.002 (0)	-250.53 (13.17)	0.315 (0.025)	0.252 - 0.365
SWG	50	49	0.80	0.60	0.836 (0.015)	0.169 (0.03)	0.037 (0.014)	0.004 - 0.067	0.002 (0)	-252.35 (11.62)	0.431 (0.03)	0.346 - 0.491
SWG	50	49	0.80	0.70	0.842 (0.015)	0.168 (0.028)	0.042 (0.015)	0 - 0.077	0.002 (0)	-252.06 (11.21)	0.532 (0.028)	0.472 - 0.601
SWG	50	49	0.80	0.80	0.84 (0.01)	0.128 (0.026)	0.04 (0.01)	0.012 - 0.06	0.002 (0)	-247.04 (11.4)	0.672 (0.026)	0.598 - 0.732
SWG	50	49	0.80	0.90	0.844 (0.011)	0.134 (0.027)	0.044 (0.011)	0.01 - 0.069	0.002 (0)	-248.1 (13.05)	0.766 (0.027)	0.693 - 0.818
SWG	50	49	0.90	0.10	0.899 (0.014)	0.089 (0.031)	0.01 (0.009)	0 - 0.053	0.001 (0)	-231.82 (18.81)	0.028 (0.018)	0 - 0.081
SWG	50	49	0.90	0.20	0.903 (0.013)	0.083 (0.03)	0.01 (0.008)	0 - 0.037	0.001 (0)	-228.3 (17.7)	0.117 (0.03)	0.024 - 0.165
SWG	50	49	0.90	0.30	0.907 (0.009)	0.06 (0.018)	0.009 (0.006)	0 - 0.027	0.001 (0)	-223.29 (19)	0.24 (0.018)	0.19 - 0.288
SWG	50	49	0.90	0.40	0.91 (0.008)	0.051 (0.015)	0.011 (0.007)	0 - 0.027	0.001 (0)	-214.92 (13.3)	0.349 (0.015)	0.305 - 0.383
SWG	50	49	0.90	0.50	0.909 (0.008)	0.045 (0.014)	0.01 (0.007)	0 - 0.029	0.001 (0)	-213.98 (13.49)	0.455 (0.014)	0.423 - 0.487
SWG	50	49	0.90	0.60	0.912 (0.008)	0.041 (0.014)	0.012 (0.007)	0 - 0.03	0.001 (0)	-211.3 (13.17)	0.559 (0.014)	0.518 - 0.584
SWG	50	49	0.90	0.70	0.91 (0.007)	0.037 (0.014)	0.011 (0.006)	0 - 0.025	0.001 (0)	-211.75 (11.26)	0.663 (0.014)	0.635 - 0.699
SWG	50	49	0.90	0.80	0.909 (0.008)	0.024 (0.013)	0.01 (0.007)	0 - 0.033	0.001 (0)	-206.94 (11.5)	0.776 (0.013)	0.737 - 0.8
SWG	50	49	0.90	0.90	0.911 (0.007)	0.029 (0.014)	0.011 (0.007)	0.001 - 0.036	0.001 (0)	-208.16 (13.31)	0.871 (0.014)	0.836 - 0.899
SWG	50	100	0.10	0.10	0.118 (0.009)	0.241 (0.075)	0.018 (0.009)	0 - 0.044	0.001 (0)	-242.17 (10.72)	0.142 (0.073)	0.001 - 0.359
SWG	50	100	0.10	0.20	0.118 (0.011)	0.239 (0.091)	0.018 (0.01)	0.001 - 0.046	0.001 (0)	-241.31 (12.06)	0.076 (0.064)	0 - 0.264
SWG	50	100	0.10	0.30	0.116 (0.01)	0.223 (0.081)	0.016 (0.009)	0 - 0.043	0.001 (0)	-240.6 (9.93)	0.09 (0.066)	0.004 - 0.297
SWG	50	100	0.10	0.40	0.114 (0.012)	0.219 (0.102)	0.015 (0.011)	0 - 0.049	0.001 (0)	-243.32 (11.42)	0.186 (0.094)	0.004 - 0.396

Continued on next page

Table S1 – *Continued from previous page*

Structure	T	N	p	p_e/p_w	$\overline{\hat{p}}$ (SD)	$\overline{\hat{p_e}}/\overline{\hat{p_w}}$ (SD)	$\overline{\lvert\Delta\rvert}_p$ (SD)	Range $\lvert\Delta\rvert_p$	$\overline{SE}\lvert\Delta\rvert_p$ (SD)	$\overline{BIC}$ (SD)	$\overline{\lvert\Delta\rvert}_{p_e/p_w}$ (SD)	Range $\lvert\Delta\rvert_{p_e/p_w}$
SWG	50	100	0.10	0.50	0.106 (0.012)	0.157 (0.096)	0.009 (0.009)	0 - 0.044	0.001 (0)	-246.8 (14.41)	0.343 (0.095)	0.015 - 0.495
SWG	50	100	0.10	0.60	0.106 (0.012)	0.157 (0.098)	0.009 (0.01)	0 - 0.05	0.001 (0)	-246.84 (15.32)	0.443 (0.098)	0.141 - 0.589
SWG	50	100	0.10	0.70	0.105 (0.012)	0.144 (0.108)	0.009 (0.009)	0 - 0.046	0.001 (0)	-249.26 (15.93)	0.556 (0.108)	0.167 - 0.698
SWG	50	100	0.10	0.80	0.102 (0.023)	0.141 (0.108)	0.011 (0.02)	0 - 0.196	0.001 (0)	-252.66 (17.01)	0.659 (0.108)	0.283 - 0.787
SWG	50	100	0.10	0.90	0.152 (0.181)	0.198 (0.173)	0.086 (0.167)	0.001 - 0.715	0.001 (0)	-250.57 (18.75)	0.702 (0.173)	0.094 - 0.889
SWG	50	100	0.20	0.10	0.228 (0.019)	0.252 (0.072)	0.029 (0.018)	0 - 0.086	0.001 (0)	-268.19 (9.06)	0.152 (0.071)	0.012 - 0.453
SWG	50	100	0.20	0.20	0.228 (0.022)	0.274 (0.091)	0.031 (0.019)	0 - 0.088	0.001 (0)	-268.22 (9.73)	0.092 (0.073)	0 - 0.383
SWG	50	100	0.20	0.30	0.226 (0.022)	0.254 (0.085)	0.027 (0.02)	0 - 0.08	0.001 (0)	-268.84 (9.46)	0.079 (0.055)	0.001 - 0.206
SWG	50	100	0.20	0.40	0.225 (0.026)	0.269 (0.107)	0.028 (0.023)	0 - 0.123	0.001 (0)	-269.9 (10.52)	0.148 (0.082)	0.005 - 0.346
SWG	50	100	0.20	0.50	0.209 (0.026)	0.205 (0.114)	0.019 (0.02)	0 - 0.079	0.001 (0)	-271.38 (11.55)	0.297 (0.11)	0.021 - 0.415
SWG	50	100	0.20	0.60	0.213 (0.03)	0.232 (0.129)	0.024 (0.023)	0 - 0.105	0.001 (0)	-272.08 (12.63)	0.368 (0.129)	0.006 - 0.531
SWG	50	100	0.20	0.70	0.206 (0.026)	0.197 (0.109)	0.021 (0.016)	0 - 0.074	0.001 (0)	-272.3 (12.85)	0.503 (0.109)	0.221 - 0.635
SWG	50	100	0.20	0.80	0.212 (0.03)	0.227 (0.132)	0.025 (0.02)	0 - 0.088	0.001 (0)	-271.67 (12.96)	0.573 (0.132)	0.265 - 0.737
SWG	50	100	0.20	0.90	0.206 (0.029)	0.201 (0.122)	0.025 (0.015)	0 - 0.062	0.001 (0)	-274.67 (12.77)	0.699 (0.122)	0.464 - 0.841
SWG	50	100	0.30	0.10	0.327 (0.025)	0.196 (0.045)	0.029 (0.022)	0.001 - 0.103	0.002 (0)	-281.61 (9.84)	0.096 (0.045)	0.022 - 0.229
SWG	50	100	0.30	0.20	0.327 (0.03)	0.214 (0.054)	0.032 (0.024)	0.001 - 0.097	0.002 (0)	-280.98 (8.57)	0.043 (0.035)	0.001 - 0.164
SWG	50	100	0.30	0.30	0.321 (0.028)	0.196 (0.049)	0.026 (0.023)	0 - 0.108	0.002 (0)	-283.51 (8.58)	0.105 (0.046)	0.002 - 0.205

Continued on next page

Table S1 – *Continued from previous page*

Structure	T	N	p	p_e/p_w	$\overline{\hat{p}}$ (SD)	$\overline{\hat{p_e}}/\overline{\hat{p_w}}$ (SD)	$\overline{\lvert\Delta\rvert}_p$ (SD)	Range $\lvert\Delta\rvert_p$	$\overline{SE}\lvert\Delta\rvert_p$ (SD)	$\overline{BIC}$ (SD)	$\overline{\lvert\Delta\rvert}_{p_e/p_w}$ (SD)	Range $\lvert\Delta\rvert_{p_e/p_w}$
SWG	50	100	0.30	0.40	0.317 (0.034)	0.205 (0.066)	0.03 (0.024)	0 - 0.122	0.002 (0)	-282.2 (10.41)	0.196 (0.063)	0.031 - 0.293
SWG	50	100	0.30	0.50	0.3 (0.033)	0.166 (0.062)	0.028 (0.019)	0.001 - 0.087	0.002 (0)	-283.21 (10.09)	0.334 (0.062)	0.137 - 0.421
SWG	50	100	0.30	0.60	0.302 (0.033)	0.182 (0.067)	0.026 (0.019)	0.001 - 0.092	0.002 (0)	-282.87 (9.81)	0.418 (0.067)	0.212 - 0.516
SWG	50	100	0.30	0.70	0.3 (0.035)	0.173 (0.071)	0.029 (0.018)	0.001 - 0.094	0.002 (0)	-286.05 (12.02)	0.527 (0.071)	0.33 - 0.623
SWG	50	100	0.30	0.80	0.296 (0.033)	0.171 (0.069)	0.03 (0.016)	0.001 - 0.068	0.002 (0)	-281.92 (11.76)	0.629 (0.069)	0.468 - 0.736
SWG	50	100	0.30	0.90	0.296 (0.033)	0.168 (0.061)	0.028 (0.017)	0.001 - 0.086	0.002 (0)	-284.42 (11.3)	0.732 (0.061)	0.581 - 0.834
SWG	50	100	0.40	0.10	0.408 (0.034)	0.096 (0.025)	0.029 (0.02)	0 - 0.073	0.003 (0)	-286.98 (9.41)	0.021 (0.014)	0 - 0.06
SWG	50	100	0.40	0.20	0.411 (0.033)	0.101 (0.024)	0.027 (0.022)	0.001 - 0.088	0.003 (0)	-285.72 (9.46)	0.099 (0.024)	0.04 - 0.152
SWG	50	100	0.40	0.30	0.402 (0.03)	0.098 (0.025)	0.023 (0.019)	0 - 0.115	0.003 (0)	-285.2 (8.42)	0.202 (0.025)	0.134 - 0.253
SWG	50	100	0.40	0.40	0.399 (0.032)	0.102 (0.023)	0.025 (0.019)	0.001 - 0.083	0.003 (0)	-285.85 (10.16)	0.298 (0.023)	0.247 - 0.348
SWG	50	100	0.40	0.50	0.396 (0.028)	0.091 (0.021)	0.024 (0.015)	0.002 - 0.06	0.003 (0)	-286.7 (8.94)	0.409 (0.021)	0.348 - 0.45
SWG	50	100	0.40	0.60	0.39 (0.025)	0.094 (0.021)	0.021 (0.016)	0 - 0.07	0.003 (0)	-284.7 (8.85)	0.506 (0.021)	0.438 - 0.551
SWG	50	100	0.40	0.70	0.394 (0.031)	0.089 (0.019)	0.025 (0.018)	0 - 0.089	0.003 (0)	-286.44 (10.36)	0.611 (0.019)	0.559 - 0.648
SWG	50	100	0.40	0.80	0.385 (0.027)	0.089 (0.02)	0.026 (0.017)	0 - 0.069	0.003 (0)	-287.72 (10.17)	0.711 (0.02)	0.645 - 0.741
SWG	50	100	0.40	0.90	0.384 (0.028)	0.083 (0.023)	0.027 (0.018)	0.001 - 0.069	0.003 (0)	-289.49 (10.89)	0.817 (0.023)	0.746 - 0.859
SWG	50	100	0.50	0.10	0.504 (0.031)	0.013 (0.01)	0.024 (0.02)	0 - 0.092	0.005 (0.001)	-286.47 (9.63)	0.087 (0.01)	0.056 - 0.1
SWG	50	100	0.50	0.20	0.504 (0.04)	0.013 (0.008)	0.032 (0.024)	0.001 - 0.103	0.005 (0.001)	-287.13 (10.68)	0.187 (0.008)	0.164 - 0.199
SWG	50	100	0.50	0.30	0.497 (0.035)	0.012 (0.01)	0.028 (0.021)	0 - 0.09	0.005 (0.001)	-289.01 (10.55)	0.288 (0.01)	0.263 - 0.299

Continued on next page

Table S1 – *Continued from previous page*

Structure	T	N	p	p_e/p_w	$\overline{\hat{p}}$ (SD)	$\overline{\hat{p_e}}/\overline{\hat{p_w}}$ (SD)	$\overline{\lvert\Delta\rvert}_p$ (SD)	Range $\lvert\Delta\rvert_p$	$\overline{SE}\lvert\Delta\rvert_p$ (SD)	$\overline{BIC}$ (SD)	$\overline{\lvert\Delta\rvert}_{p_e/p_w}$ (SD)	Range $\lvert\Delta\rvert_{p_e/p_w}$
SWG	50	100	0.50	0.40	0.51 (0.032)	0.014 (0.01)	0.027 (0.018)	0.001 - 0.084	0.005 (0)	-285.87 (9.02)	0.386 (0.01)	0.349 - 0.4
SWG	50	100	0.50	0.50	0.507 (0.031)	0.013 (0.009)	0.027 (0.017)	0.001 - 0.082	0.005 (0)	-285.84 (9.87)	0.487 (0.009)	0.459 - 0.5
SWG	50	100	0.50	0.60	0.5 (0.03)	0.012 (0.008)	0.023 (0.019)	0 - 0.094	0.004 (0)	-285.33 (10.23)	0.588 (0.008)	0.561 - 0.6
SWG	50	100	0.50	0.70	0.5 (0.035)	0.01 (0.007)	0.028 (0.02)	0 - 0.098	0.004 (0)	-286.71 (8.9)	0.69 (0.007)	0.662 - 0.7
SWG	50	100	0.50	0.80	0.504 (0.032)	0.011 (0.008)	0.025 (0.019)	0.001 - 0.106	0.004 (0)	-286.57 (9.2)	0.789 (0.008)	0.764 - 0.8
SWG	50	100	0.50	0.90	0.51 (0.026)	0.013 (0.01)	0.022 (0.017)	0 - 0.085	0.004 (0)	-288.53 (9.79)	0.887 (0.01)	0.854 - 0.9
SWG	50	100	0.60	0.10	0.608 (0.036)	0.093 (0.017)	0.028 (0.023)	0 - 0.131	0.004 (0)	-287.09 (8.45)	0.015 (0.01)	0 - 0.042
SWG	50	100	0.60	0.20	0.613 (0.037)	0.098 (0.018)	0.031 (0.024)	0 - 0.122	0.004 (0)	-285.52 (9.2)	0.102 (0.018)	0.047 - 0.14
SWG	50	100	0.60	0.30	0.608 (0.033)	0.093 (0.018)	0.026 (0.02)	0 - 0.095	0.004 (0)	-284.64 (9.36)	0.207 (0.018)	0.148 - 0.247
SWG	50	100	0.60	0.40	0.621 (0.031)	0.1 (0.015)	0.029 (0.023)	0 - 0.09	0.004 (0)	-287.46 (8.24)	0.3 (0.015)	0.27 - 0.335
SWG	50	100	0.60	0.50	0.623 (0.036)	0.089 (0.014)	0.035 (0.024)	0 - 0.107	0.004 (0)	-285.93 (9.55)	0.411 (0.014)	0.376 - 0.451
SWG	50	100	0.60	0.60	0.628 (0.032)	0.098 (0.018)	0.035 (0.024)	0.001 - 0.109	0.004 (0)	-286.83 (9.78)	0.502 (0.018)	0.447 - 0.536
SWG	50	100	0.60	0.70	0.629 (0.032)	0.091 (0.018)	0.036 (0.024)	0 - 0.113	0.004 (0)	-287.89 (11.18)	0.609 (0.018)	0.565 - 0.662
SWG	50	100	0.60	0.80	0.628 (0.033)	0.089 (0.017)	0.036 (0.024)	0 - 0.126	0.004 (0)	-288.59 (9.02)	0.711 (0.017)	0.668 - 0.764
SWG	50	100	0.60	0.90	0.624 (0.034)	0.083 (0.017)	0.034 (0.023)	0 - 0.105	0.004 (0)	-284.5 (9.09)	0.817 (0.017)	0.778 - 0.856
SWG	50	100	0.70	0.10	0.707 (0.034)	0.169 (0.017)	0.028 (0.02)	0 - 0.091	0.004 (0)	-282.83 (8.98)	0.069 (0.017)	0.031 - 0.101
SWG	50	100	0.70	0.20	0.715 (0.028)	0.182 (0.017)	0.026 (0.018)	0 - 0.087	0.003 (0)	-284.08 (8.99)	0.021 (0.013)	0 - 0.067
SWG	50	100	0.70	0.30	0.726 (0.031)	0.179 (0.018)	0.033 (0.022)	0 - 0.087	0.003 (0)	-282.46 (9.86)	0.121 (0.018)	0.072 - 0.165
SWG	50	100	0.70	0.40	0.735 (0.029)	0.192 (0.019)	0.04 (0.022)	0.001 - 0.088	0.003 (0)	-283.79 (9.8)	0.208 (0.019)	0.152 - 0.253

Continued on next page

Table S1 – *Continued from previous page*

Structure	T	N	p	p_e/p_w	$\overline{\hat{p}}$ (SD)	$\overline{\hat{p}_e}/\overline{\hat{p}_w}$ (SD)	$\overline{\lvert\Delta\rvert}_p$ (SD)	Range $\lvert\Delta\rvert_p$	$\overline{SE}\lvert\Delta\rvert_p$ (SD)	$\overline{BIC}$ (SD)	$\overline{\lvert\Delta\rvert}_{p_e/p_w}$ (SD)	Range $\lvert\Delta\rvert_{p_e/p_w}$
SWG	50	100	0.70	0.50	0.736 (0.025)	0.165 (0.014)	0.039 (0.021)	0.001 - 0.095	0.003 (0)	-285.59 (8.66)	0.335 (0.014)	0.304 - 0.369
SWG	50	100	0.70	0.60	0.742 (0.033)	0.182 (0.017)	0.046 (0.026)	0 - 0.1	0.003 (0)	-286.19 (10.35)	0.418 (0.017)	0.364 - 0.458
SWG	50	100	0.70	0.70	0.748 (0.029)	0.17 (0.014)	0.049 (0.026)	0.001 - 0.106	0.003 (0)	-286.18 (11.18)	0.53 (0.014)	0.503 - 0.578
SWG	50	100	0.70	0.80	0.758 (0.026)	0.173 (0.016)	0.058 (0.026)	0 - 0.111	0.003 (0)	-287.2 (9.14)	0.627 (0.016)	0.588 - 0.668
SWG	50	100	0.70	0.90	0.75 (0.029)	0.154 (0.016)	0.053 (0.023)	0.002 - 0.099	0.003 (0)	-288.16 (10.42)	0.746 (0.016)	0.686 - 0.786
SWG	50	100	0.80	0.10	0.793 (0.025)	0.215 (0.015)	0.02 (0.016)	0.001 - 0.082	0.003 (0)	-277.48 (7.68)	0.115 (0.015)	0.076 - 0.158
SWG	50	100	0.80	0.20	0.806 (0.025)	0.233 (0.016)	0.02 (0.016)	0 - 0.083	0.002 (0)	-282.24 (11.48)	0.033 (0.016)	0 - 0.067
SWG	50	100	0.80	0.30	0.813 (0.023)	0.221 (0.017)	0.023 (0.013)	0 - 0.061	0.002 (0)	-283.54 (9.93)	0.079 (0.017)	0.045 - 0.132
SWG	50	100	0.80	0.40	0.827 (0.021)	0.224 (0.017)	0.031 (0.015)	0.001 - 0.059	0.002 (0)	-288.09 (10.66)	0.176 (0.017)	0.126 - 0.216
SWG	50	100	0.80	0.50	0.834 (0.013)	0.18 (0.021)	0.034 (0.013)	0.003 - 0.064	0.002 (0)	-291.11 (14.57)	0.32 (0.021)	0.264 - 0.363
SWG	50	100	0.80	0.60	0.839 (0.011)	0.186 (0.025)	0.039 (0.011)	0.009 - 0.063	0.002 (0)	-293.68 (13.72)	0.414 (0.025)	0.351 - 0.473
SWG	50	100	0.80	0.70	0.842 (0.011)	0.158 (0.024)	0.042 (0.011)	0.005 - 0.059	0.001 (0)	-291.41 (15.47)	0.542 (0.024)	0.475 - 0.588
SWG	50	100	0.80	0.80	0.846 (0.007)	0.142 (0.025)	0.046 (0.007)	0.017 - 0.064	0.001 (0)	-288.51 (15.76)	0.658 (0.025)	0.59 - 0.727
SWG	50	100	0.80	0.90	0.844 (0.007)	0.115 (0.019)	0.044 (0.007)	0.026 - 0.058	0.001 (0)	-286.98 (14.68)	0.785 (0.019)	0.722 - 0.822
SWG	50	100	0.90	0.10	0.861 (0.042)	0.135 (0.043)	0.04 (0.041)	0 - 0.177	0.001 (0.001)	-276.16 (16.95)	0.045 (0.032)	0 - 0.119
SWG	50	100	0.90	0.20	0.886 (0.025)	0.133 (0.051)	0.018 (0.023)	0 - 0.114	0.001 (0.001)	-287.42 (23.28)	0.075 (0.038)	0 - 0.149
SWG	50	100	0.90	0.30	0.901 (0.013)	0.095 (0.044)	0.008 (0.011)	0 - 0.072	0.001 (0)	-284.63 (31.48)	0.205 (0.044)	0.079 - 0.255
SWG	50	100	0.90	0.40	0.908 (0.006)	0.06 (0.014)	0.008 (0.005)	0 - 0.021	0.001 (0)	-262.19 (20.35)	0.34 (0.014)	0.287 - 0.365
SWG	50	100	0.90	0.50	0.909 (0.006)	0.038 (0.01)	0.009 (0.005)	0 - 0.02	0.001 (0)	-248.7 (12.85)	0.462 (0.01)	0.438 - 0.49

Continued on next page

Table S1 – *Continued from previous page*

Structure	T	N	p	p_e/p_w	$\overline{\hat{p}}$ (SD)	$\overline{\hat{p}_e}/\overline{\hat{p}_w}$ (SD)	$\overline{\lvert\Delta\rvert}_p$ (SD)	Range $\lvert\Delta\rvert_p$	$\overline{SE}\lvert\Delta\rvert_p$ (SD)	$\overline{BIC}$ (SD)	$\overline{\lvert\Delta\rvert}_{p_e/p_w}$ (SD)	Range $\lvert\Delta\rvert_{p_e/p_w}$
SWG	50	100	0.90	0.60	0.909 (0.005)	0.04 (0.01)	0.01 (0.005)	0 - 0.022	0.001 (0)	-250.45 (13.32)	0.56 (0.01)	0.54 - 0.582
SWG	50	100	0.90	0.70	0.909 (0.006)	0.032 (0.01)	0.009 (0.005)	0 - 0.023	0.001 (0)	-245.97 (12.54)	0.668 (0.01)	0.645 - 0.692
SWG	50	100	0.90	0.80	0.908 (0.005)	0.027 (0.01)	0.008 (0.004)	0.001 - 0.019	0.001 (0)	-244.19 (10.07)	0.773 (0.01)	0.739 - 0.8
SWG	50	100	0.90	0.90	0.909 (0.005)	0.021 (0.009)	0.009 (0.005)	0 - 0.021	0.001 (0)	-243.4 (11.3)	0.879 (0.009)	0.855 - 0.899
SWG	100	16	0.10	0.10	0.107 (0.015)	0.283 (0.168)	0.012 (0.011)	0 - 0.053	0.001 (0)	-303.09 (15.5)	0.194 (0.155)	0.001 - 0.713
SWG	100	16	0.10	0.20	0.106 (0.015)	0.284 (0.166)	0.013 (0.01)	0 - 0.044	0.001 (0)	-302.48 (16.37)	0.141 (0.121)	0.001 - 0.477
SWG	100	16	0.10	0.30	0.106 (0.016)	0.286 (0.177)	0.013 (0.011)	0 - 0.055	0.001 (0)	-303.31 (16.08)	0.154 (0.088)	0 - 0.376
SWG	100	16	0.10	0.40	0.105 (0.013)	0.283 (0.154)	0.011 (0.009)	0 - 0.038	0.001 (0)	-303.26 (13.93)	0.161 (0.106)	0.002 - 0.39
SWG	100	16	0.10	0.50	0.103 (0.013)	0.228 (0.15)	0.01 (0.008)	0 - 0.038	0.001 (0)	-303.21 (15.27)	0.28 (0.135)	0.001 - 0.494
SWG	100	16	0.10	0.60	0.105 (0.015)	0.267 (0.179)	0.012 (0.011)	0 - 0.059	0.001 (0)	-302.5 (14.59)	0.343 (0.159)	0.005 - 0.592
SWG	100	16	0.10	0.70	0.103 (0.012)	0.231 (0.159)	0.01 (0.008)	0.001 - 0.035	0.001 (0)	-304.07 (15.26)	0.469 (0.159)	0.103 - 0.698
SWG	100	16	0.10	0.80	0.104 (0.015)	0.258 (0.188)	0.012 (0.009)	0.001 - 0.038	0.001 (0)	-304.53 (17.26)	0.542 (0.188)	0.186 - 0.798
SWG	100	16	0.10	0.90	0.103 (0.016)	0.247 (0.182)	0.013 (0.01)	0 - 0.056	0.001 (0)	-304.43 (15.7)	0.653 (0.182)	0.205 - 0.891
SWG	100	16	0.20	0.10	0.211 (0.025)	0.354 (0.133)	0.02 (0.018)	0 - 0.095	0.001 (0)	-368.87 (14.3)	0.254 (0.133)	0.028 - 0.655
SWG	100	16	0.20	0.20	0.214 (0.025)	0.365 (0.135)	0.023 (0.017)	0.001 - 0.07	0.001 (0)	-367.72 (13.18)	0.177 (0.12)	0.001 - 0.46
SWG	100	16	0.20	0.30	0.215 (0.025)	0.385 (0.132)	0.023 (0.019)	0 - 0.081	0.001 (0)	-363.82 (13.39)	0.123 (0.098)	0 - 0.408
SWG	100	16	0.20	0.40	0.217 (0.025)	0.404 (0.145)	0.024 (0.019)	0 - 0.089	0.001 (0)	-368.53 (14.78)	0.117 (0.085)	0 - 0.369
SWG	100	16	0.20	0.50	0.208 (0.022)	0.289 (0.11)	0.017 (0.015)	0.001 - 0.066	0.001 (0)	-370.15 (13.64)	0.215 (0.101)	0.002 - 0.4
SWG	100	16	0.20	0.60	0.213 (0.026)	0.383 (0.141)	0.023 (0.018)	0.001 - 0.084	0.001 (0)	-367.13 (15.79)	0.224 (0.129)	0.002 - 0.489
SWG	100	16	0.20	0.70	0.208 (0.022)	0.288 (0.117)	0.018 (0.015)	0 - 0.07	0.001 (0)	-367.94 (12.8)	0.412 (0.117)	0.082 - 0.622

Continued on next page

Table S1 – *Continued from previous page*

Structure	T	N	p	p_e/p_w	$\bar{\hat{p}}$ (SD)	$\overline{\hat{p}_e}/\overline{\hat{p}_w}$ (SD)	$\overline{\lvert\Delta\rvert}_p$ (SD)	Range $\lvert\Delta\rvert_p$	$\overline{SE}\lvert\Delta\rvert_p$ (SD)	$\overline{BIC}$ (SD)	$\overline{\lvert\Delta\rvert}_{p_e/p_w}$ (SD)	Range $\lvert\Delta\rvert_{p_e/p_w}$
SWG	100	16	0.20	0.80	0.205 (0.025)	0.303 (0.138)	0.02 (0.015)	0 - 0.071	0.001 (0)	-370.79 (15.35)	0.497 (0.138)	0.121 - 0.728
SWG	100	16	0.20	0.90	0.212 (0.025)	0.343 (0.138)	0.022 (0.017)	0 - 0.069	0.001 (0)	-367.76 (15.35)	0.557 (0.138)	0.25 - 0.787
SWG	100	16	0.30	0.10	0.309 (0.024)	0.273 (0.068)	0.02 (0.015)	0 - 0.064	0.002 (0)	-396.33 (14.62)	0.173 (0.068)	0.016 - 0.409
SWG	100	16	0.30	0.20	0.305 (0.024)	0.274 (0.068)	0.019 (0.015)	0 - 0.083	0.002 (0)	-392.66 (12.49)	0.082 (0.058)	0 - 0.23
SWG	100	16	0.30	0.30	0.307 (0.025)	0.272 (0.075)	0.021 (0.016)	0 - 0.076	0.002 (0)	-393.39 (13.71)	0.064 (0.047)	0.001 - 0.278
SWG	100	16	0.30	0.40	0.309 (0.023)	0.298 (0.067)	0.019 (0.017)	0 - 0.082	0.002 (0)	-395.86 (13.83)	0.108 (0.057)	0.002 - 0.233
SWG	100	16	0.30	0.50	0.3 (0.021)	0.207 (0.063)	0.017 (0.011)	0 - 0.053	0.002 (0)	-394.82 (13.42)	0.293 (0.063)	0.105 - 0.424
SWG	100	16	0.30	0.60	0.308 (0.026)	0.286 (0.077)	0.02 (0.018)	0 - 0.09	0.002 (0)	-395.13 (15.32)	0.314 (0.077)	0.111 - 0.463
SWG	100	16	0.30	0.70	0.294 (0.023)	0.184 (0.063)	0.019 (0.015)	0.001 - 0.057	0.002 (0)	-396.93 (13.35)	0.516 (0.063)	0.376 - 0.659
SWG	100	16	0.30	0.80	0.299 (0.024)	0.234 (0.067)	0.019 (0.015)	0 - 0.061	0.002 (0)	-394.8 (13.88)	0.566 (0.067)	0.361 - 0.698
SWG	100	16	0.30	0.90	0.302 (0.02)	0.237 (0.069)	0.016 (0.013)	0 - 0.061	0.002 (0)	-392.96 (13.43)	0.663 (0.069)	0.467 - 0.804
SWG	100	16	0.40	0.10	0.403 (0.029)	0.136 (0.052)	0.024 (0.016)	0.002 - 0.076	0.003 (0)	-403.31 (12.68)	0.047 (0.042)	0.001 - 0.256
SWG	100	16	0.40	0.20	0.4 (0.023)	0.135 (0.04)	0.019 (0.013)	0 - 0.044	0.003 (0)	-405.71 (15.48)	0.066 (0.038)	0.003 - 0.17
SWG	100	16	0.40	0.30	0.395 (0.025)	0.135 (0.039)	0.02 (0.015)	0 - 0.06	0.003 (0)	-405.2 (12.59)	0.165 (0.039)	0.047 - 0.263
SWG	100	16	0.40	0.40	0.4 (0.025)	0.154 (0.044)	0.02 (0.015)	0 - 0.076	0.003 (0)	-403.9 (13.56)	0.246 (0.044)	0.135 - 0.361
SWG	100	16	0.40	0.50	0.391 (0.029)	0.092 (0.044)	0.023 (0.02)	0 - 0.127	0.003 (0)	-404.55 (14)	0.408 (0.044)	0.283 - 0.493
SWG	100	16	0.40	0.60	0.387 (0.026)	0.132 (0.046)	0.023 (0.018)	0.001 - 0.081	0.003 (0)	-401.22 (15.09)	0.468 (0.046)	0.346 - 0.56
SWG	100	16	0.40	0.70	0.395 (0.028)	0.092 (0.042)	0.024 (0.016)	0 - 0.076	0.003 (0)	-404.77 (12.98)	0.608 (0.042)	0.479 - 0.699

Continued on next page

Table S1 – *Continued from previous page*

Structure	T	N	p	p_e/p_w	$\bar{\hat{p}}$ (SD)	$\overline{\hat{p_e}}/\overline{\hat{p_w}}$ (SD)	$\overline{\lvert\Delta\rvert}_p$ (SD)	Range $\lvert\Delta\rvert_p$	$\overline{SE}\lvert\Delta\rvert_p$ (SD)	$\overline{BIC}$ (SD)	$\overline{\lvert\Delta\rvert}_{p_e/p_w}$ (SD)	Range $\lvert\Delta\rvert_{p_e/p_w}$
SWG	100	16	0.40	0.80	0.39 (0.026)	0.099 (0.048)	0.023 (0.016)	0.001 - 0.065	0.003 (0)	-405.23 (12.94)	0.701 (0.048)	0.56 - 0.792
SWG	100	16	0.40	0.90	0.388 (0.027)	0.113 (0.034)	0.023 (0.018)	0 - 0.085	0.003 (0)	-405 (15.03)	0.787 (0.034)	0.688 - 0.855
SWG	100	16	0.50	0.10	0.504 (0.033)	0.027 (0.022)	0.027 (0.019)	0 - 0.078	0.003 (0)	-407.39 (13.98)	0.073 (0.022)	0.004 - 0.1
SWG	100	16	0.50	0.20	0.502 (0.032)	0.029 (0.022)	0.026 (0.018)	0 - 0.096	0.003 (0)	-408.53 (13.16)	0.171 (0.022)	0.105 - 0.199
SWG	100	16	0.50	0.30	0.5 (0.03)	0.03 (0.029)	0.025 (0.017)	0 - 0.071	0.003 (0)	-404 (13.75)	0.27 (0.029)	0.135 - 0.3
SWG	100	16	0.50	0.40	0.504 (0.03)	0.032 (0.025)	0.024 (0.019)	0 - 0.095	0.003 (0)	-405.72 (14)	0.368 (0.025)	0.3 - 0.399
SWG	100	16	0.50	0.50	0.501 (0.029)	0.029 (0.023)	0.025 (0.016)	0 - 0.087	0.003 (0)	-404.3 (12.59)	0.471 (0.023)	0.388 - 0.5
SWG	100	16	0.50	0.60	0.508 (0.031)	0.028 (0.023)	0.025 (0.021)	0 - 0.085	0.003 (0)	-406.46 (13.24)	0.572 (0.023)	0.493 - 0.6
SWG	100	16	0.50	0.70	0.509 (0.031)	0.033 (0.027)	0.025 (0.019)	0.001 - 0.082	0.003 (0)	-407.29 (15.32)	0.667 (0.027)	0.525 - 0.7
SWG	100	16	0.50	0.80	0.503 (0.033)	0.026 (0.019)	0.026 (0.02)	0 - 0.088	0.003 (0)	-408.59 (13.69)	0.774 (0.019)	0.714 - 0.799
SWG	100	16	0.50	0.90	0.506 (0.03)	0.029 (0.022)	0.025 (0.018)	0.001 - 0.088	0.003 (0)	-406.24 (13.14)	0.871 (0.022)	0.81 - 0.9
SWG	100	16	0.60	0.10	0.622 (0.033)	0.13 (0.039)	0.032 (0.023)	0 - 0.112	0.003 (0)	-403.62 (13.34)	0.041 (0.028)	0.001 - 0.105
SWG	100	16	0.60	0.20	0.628 (0.029)	0.136 (0.033)	0.033 (0.023)	0.002 - 0.112	0.003 (0)	-406.38 (15.01)	0.064 (0.032)	0.006 - 0.149
SWG	100	16	0.60	0.30	0.63 (0.032)	0.132 (0.036)	0.036 (0.024)	0.002 - 0.094	0.003 (0)	-405.18 (14.64)	0.168 (0.036)	0.057 - 0.254
SWG	100	16	0.60	0.40	0.625 (0.034)	0.14 (0.04)	0.034 (0.024)	0 - 0.105	0.003 (0)	-405.69 (12.8)	0.26 (0.04)	0.148 - 0.362
SWG	100	16	0.60	0.50	0.62 (0.029)	0.096 (0.037)	0.029 (0.02)	0.001 - 0.084	0.003 (0)	-403.52 (12.39)	0.404 (0.037)	0.295 - 0.482
SWG	100	16	0.60	0.60	0.626 (0.027)	0.129 (0.031)	0.032 (0.019)	0 - 0.094	0.003 (0)	-405.13 (12.36)	0.471 (0.031)	0.388 - 0.55
SWG	100	16	0.60	0.70	0.621 (0.029)	0.097 (0.034)	0.03 (0.019)	0.001 - 0.086	0.003 (0)	-403.19 (11.26)	0.603 (0.034)	0.527 - 0.695
SWG	100	16	0.60	0.80	0.628 (0.027)	0.109 (0.031)	0.031 (0.023)	0.001 - 0.104	0.003 (0)	-408.64 (11.75)	0.691 (0.031)	0.607 - 0.768

Continued on next page

Table S1 – *Continued from previous page*

Structure	T	N	p	p_e/p_w	$\overline{\hat{p}}$ (SD)	$\overline{\hat{p_e}}/\overline{\hat{p_w}}$ (SD)	$\overline{\lvert\Delta\rvert}_p$ (SD)	Range $\lvert\Delta\rvert_p$	$\overline{SE}\lvert\Delta\rvert_p$ (SD)	$\overline{BIC}$ (SD)	$\overline{\lvert\Delta\rvert}_{p_e/p_w}$ (SD)	Range $\lvert\Delta\rvert_{p_e/p_w}$
SWG	100	16	0.60	0.90	0.623 (0.027)	0.112 (0.036)	0.028 (0.021)	0 - 0.111	0.003 (0)	-407.52 (15.04)	0.788 (0.036)	0.716 - 0.885
SWG	100	16	0.70	0.10	0.73 (0.029)	0.223 (0.038)	0.034 (0.023)	0.001 - 0.094	0.002 (0)	-402.44 (12.7)	0.123 (0.038)	0.04 - 0.216
SWG	100	16	0.70	0.20	0.744 (0.028)	0.224 (0.033)	0.046 (0.025)	0.005 - 0.113	0.002 (0)	-401.12 (15.43)	0.032 (0.026)	0 - 0.101
SWG	100	16	0.70	0.30	0.741 (0.027)	0.232 (0.034)	0.043 (0.023)	0 - 0.103	0.002 (0)	-401.87 (14.53)	0.07 (0.029)	0.005 - 0.147
SWG	100	16	0.70	0.40	0.742 (0.027)	0.252 (0.033)	0.043 (0.024)	0 - 0.104	0.002 (0)	-403.19 (12.93)	0.148 (0.033)	0.076 - 0.218
SWG	100	16	0.70	0.50	0.735 (0.026)	0.18 (0.034)	0.038 (0.022)	0.003 - 0.094	0.002 (0)	-396.53 (14.78)	0.32 (0.034)	0.254 - 0.426
SWG	100	16	0.70	0.60	0.75 (0.025)	0.241 (0.034)	0.051 (0.024)	0.001 - 0.105	0.002 (0)	-406.16 (13.9)	0.359 (0.034)	0.264 - 0.444
SWG	100	16	0.70	0.70	0.737 (0.025)	0.161 (0.034)	0.038 (0.022)	0 - 0.094	0.002 (0)	-400.01 (14.89)	0.539 (0.034)	0.462 - 0.622
SWG	100	16	0.70	0.80	0.743 (0.023)	0.192 (0.031)	0.044 (0.021)	0.001 - 0.1	0.002 (0)	-401.44 (13.83)	0.608 (0.031)	0.5 - 0.688
SWG	100	16	0.70	0.90	0.739 (0.027)	0.204 (0.03)	0.04 (0.025)	0 - 0.1	0.002 (0)	-401.7 (13.69)	0.696 (0.03)	0.633 - 0.776
SWG	100	16	0.80	0.10	0.819 (0.021)	0.237 (0.034)	0.024 (0.016)	0 - 0.066	0.002 (0)	-396.43 (12.83)	0.137 (0.034)	0.055 - 0.214
SWG	100	16	0.80	0.20	0.833 (0.022)	0.234 (0.031)	0.036 (0.019)	0 - 0.076	0.002 (0)	-391.56 (13.04)	0.038 (0.026)	0 - 0.117
SWG	100	16	0.80	0.30	0.842 (0.016)	0.246 (0.037)	0.042 (0.015)	0.006 - 0.079	0.002 (0)	-398.24 (14)	0.057 (0.031)	0 - 0.125
SWG	100	16	0.80	0.40	0.837 (0.016)	0.274 (0.034)	0.038 (0.015)	0.002 - 0.071	0.002 (0)	-403.21 (15.65)	0.126 (0.034)	0.048 - 0.185
SWG	100	16	0.80	0.50	0.835 (0.019)	0.192 (0.037)	0.036 (0.016)	0.002 - 0.075	0.002 (0)	-391.54 (14.59)	0.308 (0.037)	0.21 - 0.435
SWG	100	16	0.80	0.60	0.846 (0.015)	0.241 (0.032)	0.046 (0.015)	0.009 - 0.076	0.002 (0)	-406.39 (17.2)	0.359 (0.032)	0.27 - 0.441
SWG	100	16	0.80	0.70	0.835 (0.017)	0.178 (0.038)	0.035 (0.017)	0 - 0.084	0.002 (0)	-393.31 (15.86)	0.522 (0.038)	0.43 - 0.66
SWG	100	16	0.80	0.80	0.841 (0.016)	0.185 (0.035)	0.041 (0.016)	0.006 - 0.081	0.002 (0)	-394.78 (16.47)	0.615 (0.035)	0.529 - 0.708

Continued on next page

Table S1 – *Continued from previous page*

Structure	T	N	p	p_e/p_w	$\overline{\hat{p}}$ (SD)	$\overline{\hat{p}_e}/\overline{\hat{p}_w}$ (SD)	$\overline{\lvert\Delta\rvert}_p$ (SD)	Range $\lvert\Delta\rvert_p$	$\overline{SE}\lvert\Delta\rvert_p$ (SD)	$\overline{BIC}$ (SD)	$\overline{\lvert\Delta\rvert}_{p_e/p_w}$ (SD)	Range $\lvert\Delta\rvert_{p_e/p_w}$
SWG	100	16	0.80	0.90	0.838 (0.018)	0.213 (0.035)	0.039 (0.017)	0.001 - 0.072	0.002 (0)	-396.78 (16.31)	0.687 (0.035)	0.597 - 0.775
SWG	100	16	0.90	0.10	0.905 (0.025)	0.095 (0.031)	0.018 (0.017)	0 - 0.14	0.001 (0)	-350.38 (28.21)	0.024 (0.02)	0 - 0.105
SWG	100	16	0.90	0.20	0.916 (0.01)	0.1 (0.036)	0.016 (0.009)	0 - 0.034	0.001 (0)	-342.76 (25.08)	0.101 (0.035)	0.004 - 0.169
SWG	100	16	0.90	0.30	0.917 (0.009)	0.083 (0.032)	0.017 (0.009)	0 - 0.042	0.001 (0)	-339.95 (25.8)	0.217 (0.032)	0.126 - 0.296
SWG	100	16	0.90	0.40	0.912 (0.012)	0.13 (0.041)	0.014 (0.01)	0 - 0.035	0.001 (0)	-351.34 (24.9)	0.27 (0.041)	0.153 - 0.353
SWG	100	16	0.90	0.50	0.912 (0.011)	0.071 (0.032)	0.014 (0.008)	0 - 0.035	0.001 (0)	-328.81 (20.06)	0.429 (0.032)	0.33 - 0.493
SWG	100	16	0.90	0.60	0.916 (0.008)	0.082 (0.032)	0.016 (0.008)	0.001 - 0.042	0.001 (0)	-338.36 (29.27)	0.518 (0.032)	0.41 - 0.586
SWG	100	16	0.90	0.70	0.915 (0.011)	0.06 (0.028)	0.016 (0.01)	0 - 0.036	0.001 (0)	-327.68 (24.68)	0.64 (0.028)	0.567 - 0.695
SWG	100	16	0.90	0.80	0.916 (0.009)	0.054 (0.024)	0.017 (0.008)	0.001 - 0.041	0.001 (0)	-323.6 (19.07)	0.746 (0.024)	0.664 - 0.793
SWG	100	16	0.90	0.90	0.916 (0.009)	0.071 (0.042)	0.016 (0.008)	0.001 - 0.039	0.001 (0)	-330.93 (27.08)	0.829 (0.042)	0.638 - 0.898
SWG	100	25	0.10	0.10	0.106 (0.013)	0.21 (0.132)	0.011 (0.01)	0 - 0.038	0.001 (0)	-354.15 (14.25)	0.126 (0.116)	0.002 - 0.421
SWG	100	25	0.10	0.20	0.106 (0.012)	0.198 (0.106)	0.01 (0.008)	0 - 0.039	0.001 (0)	-350.75 (14.35)	0.09 (0.056)	0 - 0.223
SWG	100	25	0.10	0.30	0.107 (0.014)	0.218 (0.12)	0.011 (0.01)	0 - 0.065	0.001 (0)	-350.23 (14.56)	0.124 (0.075)	0.001 - 0.333
SWG	100	25	0.10	0.40	0.105 (0.013)	0.198 (0.113)	0.01 (0.009)	0 - 0.044	0.001 (0)	-354.64 (15.88)	0.209 (0.1)	0.006 - 0.368
SWG	100	25	0.10	0.50	0.105 (0.013)	0.183 (0.117)	0.01 (0.009)	0 - 0.044	0.001 (0)	-355.21 (14.57)	0.317 (0.117)	0.001 - 0.496
SWG	100	25	0.10	0.60	0.105 (0.012)	0.199 (0.122)	0.01 (0.008)	0 - 0.031	0.001 (0)	-354.22 (17.75)	0.401 (0.122)	0.06 - 0.576
SWG	100	25	0.10	0.70	0.106 (0.013)	0.208 (0.135)	0.011 (0.01)	0 - 0.042	0.001 (0)	-352.17 (13.72)	0.492 (0.135)	0.15 - 0.691
SWG	100	25	0.10	0.80	0.104 (0.012)	0.181 (0.127)	0.01 (0.008)	0 - 0.034	0.001 (0)	-351.42 (16.67)	0.619 (0.127)	0.291 - 0.781
SWG	100	25	0.10	0.90	0.101 (0.02)	0.175 (0.148)	0.014 (0.015)	0 - 0.122	0.001 (0)	-353.61 (16.09)	0.725 (0.148)	0.258 - 0.892
SWG	100	25	0.20	0.10	0.215 (0.024)	0.291 (0.117)	0.022 (0.018)	0.001 - 0.078	0.001 (0)	-413.15 (12.9)	0.193 (0.114)	0.001 - 0.486

Continued on next page

Table S1 – *Continued from previous page*

Structure	T	N	p	p_e/p_w	$\overline{\hat{p}}$ (SD)	$\overline{\hat{p_e}}/\overline{\hat{p_w}}$ (SD)	$\overline{\lvert\Delta\rvert}_p$ (SD)	Range $\lvert\Delta\rvert_p$	$\overline{SE}\lvert\Delta\rvert_p$ (SD)	$\overline{BIC}$ (SD)	$\overline{\lvert\Delta\rvert}_{p_e/p_w}$ (SD)	Range $\lvert\Delta\rvert_{p_e/p_w}$
SWG	100	25	0.20	0.20	0.214 (0.022)	0.275 (0.104)	0.022 (0.016)	0 - 0.063	0.001 (0)	-417.6 (13.37)	0.103 (0.075)	0 - 0.318
SWG	100	25	0.20	0.30	0.219 (0.022)	0.307 (0.11)	0.023 (0.018)	0.001 - 0.073	0.001 (0)	-413.22 (13.79)	0.09 (0.063)	0 - 0.318
SWG	100	25	0.20	0.40	0.216 (0.026)	0.306 (0.126)	0.023 (0.02)	0 - 0.081	0.001 (0)	-412.54 (15.42)	0.132 (0.086)	0.001 - 0.355
SWG	100	25	0.20	0.50	0.212 (0.022)	0.271 (0.105)	0.019 (0.016)	0 - 0.072	0.001 (0)	-413.08 (14.61)	0.23 (0.104)	0.006 - 0.41
SWG	100	25	0.20	0.60	0.21 (0.023)	0.254 (0.11)	0.02 (0.016)	0 - 0.08	0.001 (0)	-414.46 (16.09)	0.346 (0.11)	0.079 - 0.547
SWG	100	25	0.20	0.70	0.211 (0.023)	0.287 (0.118)	0.019 (0.017)	0 - 0.078	0.001 (0)	-416.41 (17.36)	0.413 (0.118)	0.112 - 0.643
SWG	100	25	0.20	0.80	0.214 (0.021)	0.276 (0.106)	0.02 (0.016)	0 - 0.073	0.001 (0)	-415.99 (15.19)	0.524 (0.106)	0.217 - 0.717
SWG	100	25	0.20	0.90	0.209 (0.023)	0.266 (0.132)	0.02 (0.014)	0 - 0.062	0.001 (0)	-415.06 (16.72)	0.634 (0.132)	0.31 - 0.842
SWG	100	25	0.30	0.10	0.316 (0.021)	0.239 (0.054)	0.022 (0.015)	0.001 - 0.067	0.002 (0)	-438.59 (12.16)	0.14 (0.053)	0.027 - 0.265
SWG	100	25	0.30	0.20	0.312 (0.022)	0.213 (0.044)	0.02 (0.015)	0 - 0.064	0.002 (0)	-442.14 (12.38)	0.036 (0.029)	0 - 0.124
SWG	100	25	0.30	0.30	0.31 (0.026)	0.226 (0.054)	0.022 (0.017)	0 - 0.08	0.002 (0)	-438.98 (15.43)	0.081 (0.043)	0.002 - 0.159
SWG	100	25	0.30	0.40	0.305 (0.02)	0.22 (0.049)	0.017 (0.011)	0.001 - 0.05	0.002 (0)	-440.5 (13.69)	0.18 (0.049)	0.054 - 0.286
SWG	100	25	0.30	0.50	0.307 (0.021)	0.206 (0.053)	0.017 (0.014)	0 - 0.062	0.002 (0)	-439.49 (14.36)	0.294 (0.053)	0.116 - 0.393
SWG	100	25	0.30	0.60	0.304 (0.025)	0.191 (0.049)	0.021 (0.014)	0 - 0.072	0.002 (0)	-437.16 (12.49)	0.409 (0.049)	0.294 - 0.506
SWG	100	25	0.30	0.70	0.312 (0.022)	0.251 (0.06)	0.019 (0.017)	0 - 0.081	0.001 (0)	-439.12 (13.49)	0.449 (0.06)	0.255 - 0.573
SWG	100	25	0.30	0.80	0.304 (0.024)	0.206 (0.063)	0.019 (0.015)	0 - 0.088	0.002 (0)	-437.73 (14.02)	0.594 (0.063)	0.416 - 0.695
SWG	100	25	0.30	0.90	0.298 (0.023)	0.182 (0.049)	0.018 (0.015)	0 - 0.062	0.002 (0)	-441.56 (12.86)	0.718 (0.049)	0.595 - 0.81
SWG	100	25	0.40	0.10	0.4 (0.021)	0.122 (0.028)	0.017 (0.013)	0 - 0.055	0.002 (0)	-449.22 (14.28)	0.029 (0.021)	0 - 0.11
SWG	100	25	0.40	0.20	0.403 (0.027)	0.108 (0.028)	0.02 (0.018)	0 - 0.075	0.002 (0)	-448.74 (15.04)	0.092 (0.028)	0.015 - 0.159

Continued on next page

Table S1 – *Continued from previous page*

Structure	T	N	p	p_e/p_w	$\overline{\hat{p}}$ (SD)	$\overline{\hat{p}_e}/\overline{\hat{p}_w}$ (SD)	$\overline{\lvert\Delta\rvert}_p$ (SD)	Range $\lvert\Delta\rvert_p$	$\overline{SE}\lvert\Delta\rvert_p$ (SD)	$\overline{BIC}$ (SD)	$\overline{\lvert\Delta\rvert}_{p_e/p_w}$ (SD)	Range $\lvert\Delta\rvert_{p_e/p_w}$
SWG	100	25	0.40	0.30	0.402 (0.024)	0.115 (0.035)	0.019 (0.015)	0 - 0.069	0.002 (0)	-449.76 (12.4)	0.185 (0.035)	0.067 - 0.254
SWG	100	25	0.40	0.40	0.394 (0.026)	0.111 (0.03)	0.021 (0.016)	0 - 0.076	0.002 (0)	-451.09 (14.07)	0.289 (0.03)	0.216 - 0.367
SWG	100	25	0.40	0.50	0.402 (0.025)	0.103 (0.029)	0.019 (0.016)	0 - 0.079	0.002 (0)	-448.46 (11.91)	0.397 (0.029)	0.31 - 0.46
SWG	100	25	0.40	0.60	0.396 (0.025)	0.094 (0.029)	0.02 (0.016)	0 - 0.075	0.002 (0)	-448.21 (12.21)	0.506 (0.029)	0.418 - 0.568
SWG	100	25	0.40	0.70	0.398 (0.029)	0.116 (0.033)	0.023 (0.016)	0 - 0.077	0.002 (0)	-450.8 (13.61)	0.584 (0.033)	0.471 - 0.653
SWG	100	25	0.40	0.80	0.396 (0.024)	0.101 (0.033)	0.018 (0.015)	0 - 0.073	0.002 (0)	-449.65 (13.43)	0.699 (0.033)	0.601 - 0.772
SWG	100	25	0.40	0.90	0.384 (0.024)	0.083 (0.032)	0.024 (0.016)	0.001 - 0.067	0.002 (0)	-447.15 (14.61)	0.817 (0.032)	0.737 - 0.881
SWG	100	25	0.50	0.10	0.503 (0.028)	0.02 (0.014)	0.023 (0.016)	0 - 0.075	0.003 (0)	-449.91 (14.03)	0.08 (0.014)	0.036 - 0.1
SWG	100	25	0.50	0.20	0.503 (0.031)	0.022 (0.017)	0.026 (0.018)	0 - 0.071	0.003 (0)	-452.43 (12.87)	0.178 (0.017)	0.136 - 0.2
SWG	100	25	0.50	0.30	0.502 (0.028)	0.022 (0.016)	0.023 (0.017)	0 - 0.075	0.003 (0)	-451.44 (12.55)	0.278 (0.016)	0.194 - 0.3
SWG	100	25	0.50	0.40	0.509 (0.025)	0.02 (0.016)	0.022 (0.014)	0.001 - 0.063	0.003 (0)	-450.35 (13)	0.38 (0.016)	0.334 - 0.4
SWG	100	25	0.50	0.50	0.504 (0.028)	0.018 (0.015)	0.022 (0.018)	0 - 0.074	0.003 (0)	-450.33 (13.85)	0.482 (0.015)	0.417 - 0.5
SWG	100	25	0.50	0.60	0.509 (0.026)	0.021 (0.016)	0.022 (0.016)	0 - 0.088	0.003 (0)	-451.74 (13.48)	0.579 (0.016)	0.522 - 0.6
SWG	100	25	0.50	0.70	0.503 (0.027)	0.02 (0.015)	0.021 (0.016)	0 - 0.07	0.003 (0)	-453.55 (15.92)	0.68 (0.015)	0.623 - 0.7
SWG	100	25	0.50	0.80	0.499 (0.022)	0.019 (0.015)	0.017 (0.014)	0 - 0.061	0.003 (0)	-454.28 (14.24)	0.781 (0.015)	0.733 - 0.8
SWG	100	25	0.50	0.90	0.504 (0.032)	0.021 (0.016)	0.024 (0.022)	0 - 0.095	0.003 (0)	-450.47 (13.61)	0.879 (0.016)	0.832 - 0.9
SWG	100	25	0.60	0.10	0.615 (0.029)	0.11 (0.024)	0.025 (0.021)	0 - 0.11	0.003 (0)	-448.92 (14.87)	0.021 (0.016)	0 - 0.094
SWG	100	25	0.60	0.20	0.622 (0.031)	0.103 (0.032)	0.031 (0.022)	0 - 0.093	0.003 (0)	-452.03 (14.26)	0.097 (0.032)	0.011 - 0.156
SWG	100	25	0.60	0.30	0.613 (0.028)	0.109 (0.028)	0.025 (0.018)	0 - 0.066	0.003 (0)	-450.87 (13.38)	0.191 (0.028)	0.117 - 0.248
SWG	100	25	0.60	0.40	0.619 (0.032)	0.105 (0.027)	0.031 (0.022)	0.001 - 0.112	0.003 (0)	-447.58 (13.22)	0.295 (0.027)	0.229 - 0.356

Continued on next page

Table S1 – *Continued from previous page*

Structure	T	N	p	p_e/p_w	$\overline{\hat{p}}$ (SD)	$\overline{\hat{p_e}}/\overline{\hat{p_w}}$ (SD)	$\overline{\lvert\Delta\rvert}_p$ (SD)	Range $\lvert\Delta\rvert_p$	$\overline{SE}\lvert\Delta\rvert_p$ (SD)	$\overline{BIC}$ (SD)	$\overline{\lvert\Delta\rvert}_{p_e/p_w}$ (SD)	Range $\lvert\Delta\rvert_{p_e/p_w}$
SWG	100	25	0.60	0.50	0.621 (0.027)	0.098 (0.027)	0.028 (0.019)	0.001 - 0.076	0.003 (0)	-449.95 (14.48)	0.402 (0.027)	0.331 - 0.474
SWG	100	25	0.60	0.60	0.622 (0.027)	0.092 (0.025)	0.029 (0.019)	0.001 - 0.081	0.003 (0)	-450.72 (14.17)	0.508 (0.025)	0.439 - 0.561
SWG	100	25	0.60	0.70	0.624 (0.029)	0.112 (0.027)	0.031 (0.022)	0 - 0.092	0.003 (0)	-450.67 (14.5)	0.588 (0.027)	0.532 - 0.668
SWG	100	25	0.60	0.80	0.623 (0.024)	0.099 (0.023)	0.027 (0.018)	0.001 - 0.072	0.003 (0)	-450.37 (14.05)	0.701 (0.023)	0.632 - 0.759
SWG	100	25	0.60	0.90	0.633 (0.027)	0.089 (0.027)	0.037 (0.023)	0.001 - 0.09	0.003 (0)	-451.68 (14.99)	0.811 (0.027)	0.75 - 0.885
SWG	100	25	0.70	0.10	0.734 (0.025)	0.209 (0.024)	0.037 (0.02)	0 - 0.086	0.002 (0)	-446.76 (13.51)	0.109 (0.024)	0.063 - 0.173
SWG	100	25	0.70	0.20	0.728 (0.026)	0.188 (0.026)	0.032 (0.02)	0.002 - 0.084	0.002 (0)	-445.48 (13.32)	0.023 (0.017)	0 - 0.07
SWG	100	25	0.70	0.30	0.726 (0.024)	0.199 (0.024)	0.03 (0.019)	0 - 0.068	0.002 (0)	-444.79 (13.11)	0.101 (0.024)	0.047 - 0.16
SWG	100	25	0.70	0.40	0.744 (0.023)	0.198 (0.021)	0.045 (0.022)	0.001 - 0.095	0.002 (0)	-445.94 (12.07)	0.202 (0.021)	0.135 - 0.249
SWG	100	25	0.70	0.50	0.732 (0.024)	0.184 (0.023)	0.034 (0.02)	0 - 0.079	0.002 (0)	-447.32 (14.14)	0.316 (0.023)	0.265 - 0.371
SWG	100	25	0.70	0.60	0.739 (0.027)	0.167 (0.026)	0.042 (0.021)	0.003 - 0.099	0.002 (0)	-443.93 (14.34)	0.433 (0.026)	0.355 - 0.498
SWG	100	25	0.70	0.70	0.744 (0.026)	0.209 (0.025)	0.045 (0.023)	0.001 - 0.096	0.002 (0)	-448.05 (14.95)	0.491 (0.025)	0.431 - 0.544
SWG	100	25	0.70	0.80	0.738 (0.021)	0.179 (0.023)	0.039 (0.019)	0.001 - 0.091	0.002 (0)	-444.79 (14.39)	0.621 (0.023)	0.57 - 0.676
SWG	100	25	0.70	0.90	0.749 (0.023)	0.157 (0.026)	0.05 (0.021)	0.006 - 0.102	0.002 (0)	-447.45 (14.29)	0.743 (0.026)	0.67 - 0.799
SWG	100	25	0.80	0.10	0.829 (0.018)	0.245 (0.028)	0.03 (0.016)	0.001 - 0.067	0.002 (0)	-440.5 (15)	0.145 (0.028)	0.082 - 0.206
SWG	100	25	0.80	0.20	0.824 (0.02)	0.22 (0.027)	0.026 (0.017)	0 - 0.073	0.002 (0)	-439.97 (15.94)	0.028 (0.019)	0.001 - 0.084
SWG	100	25	0.80	0.30	0.823 (0.018)	0.236 (0.026)	0.025 (0.015)	0.001 - 0.058	0.002 (0)	-438.31 (14.24)	0.064 (0.026)	0.008 - 0.12
SWG	100	25	0.80	0.40	0.84 (0.014)	0.208 (0.026)	0.04 (0.014)	0.002 - 0.065	0.001 (0)	-442.61 (14.84)	0.192 (0.026)	0.121 - 0.257

Continued on next page

Table S1 – *Continued from previous page*

Structure	T	N	p	p_e/p_w	$\overline{\hat{p}}$ (SD)	$\overline{\hat{p}_e}/\overline{\hat{p}_w}$ (SD)	$\overline{\lvert\Delta\rvert}_p$ (SD)	Range $\lvert\Delta\rvert_p$	$\overline{SE}\lvert\Delta\rvert_p$ (SD)	$\overline{BIC}$ (SD)	$\overline{\lvert\Delta\rvert}_{p_e/p_w}$ (SD)	Range $\lvert\Delta\rvert_{p_e/p_w}$
SWG	100	25	0.80	0.50	0.828 (0.019)	0.204 (0.027)	0.031 (0.015)	0 - 0.067	0.002 (0)	-437.92 (12.82)	0.296 (0.027)	0.22 - 0.378
SWG	100	25	0.80	0.60	0.834 (0.013)	0.176 (0.028)	0.034 (0.013)	0 - 0.067	0.001 (0)	-438.44 (14.36)	0.424 (0.028)	0.345 - 0.513
SWG	100	25	0.80	0.70	0.837 (0.017)	0.223 (0.031)	0.038 (0.015)	0 - 0.067	0.001 (0)	-452.08 (19.21)	0.477 (0.031)	0.402 - 0.581
SWG	100	25	0.80	0.80	0.838 (0.016)	0.186 (0.028)	0.039 (0.015)	0.003 - 0.072	0.001 (0)	-439.28 (13.28)	0.614 (0.028)	0.542 - 0.68
SWG	100	25	0.80	0.90	0.844 (0.011)	0.142 (0.027)	0.044 (0.011)	0.018 - 0.065	0.001 (0)	-437.1 (18.38)	0.758 (0.027)	0.685 - 0.828
SWG	100	25	0.90	0.10	0.909 (0.009)	0.113 (0.043)	0.011 (0.006)	0 - 0.027	0.001 (0)	-413.93 (29.77)	0.033 (0.03)	0 - 0.169
SWG	100	25	0.90	0.20	0.906 (0.011)	0.1 (0.042)	0.01 (0.006)	0.001 - 0.026	0.001 (0)	-405.69 (29.42)	0.101 (0.037)	0.001 - 0.185
SWG	100	25	0.90	0.30	0.908 (0.011)	0.1 (0.031)	0.011 (0.007)	0 - 0.028	0.001 (0)	-403.44 (28.19)	0.2 (0.031)	0.117 - 0.263
SWG	100	25	0.90	0.40	0.913 (0.007)	0.055 (0.016)	0.014 (0.007)	0.001 - 0.033	0.001 (0)	-373.62 (20.45)	0.345 (0.016)	0.31 - 0.389
SWG	100	25	0.90	0.50	0.91 (0.007)	0.065 (0.024)	0.011 (0.006)	0 - 0.025	0.001 (0)	-383.47 (24.43)	0.435 (0.024)	0.36 - 0.483
SWG	100	25	0.90	0.60	0.911 (0.007)	0.053 (0.018)	0.011 (0.007)	0.001 - 0.035	0.001 (0)	-371.11 (19.43)	0.547 (0.018)	0.483 - 0.588
SWG	100	25	0.90	0.70	0.911 (0.007)	0.064 (0.025)	0.011 (0.007)	0.001 - 0.029	0.001 (0)	-387.26 (27.67)	0.636 (0.025)	0.566 - 0.68
SWG	100	25	0.90	0.80	0.913 (0.008)	0.052 (0.017)	0.013 (0.007)	0 - 0.029	0.001 (0)	-371.04 (23.27)	0.748 (0.017)	0.706 - 0.788
SWG	100	25	0.90	0.90	0.913 (0.008)	0.032 (0.017)	0.013 (0.007)	0.001 - 0.032	0.001 (0)	-360.11 (17.99)	0.868 (0.017)	0.829 - 0.899
SWG	100	49	0.10	0.10	0.106 (0.01)	0.16 (0.083)	0.009 (0.008)	0 - 0.033	0 (0)	-425.19 (17.57)	0.073 (0.071)	0.002 - 0.334
SWG	100	49	0.10	0.20	0.108 (0.01)	0.177 (0.097)	0.01 (0.008)	0 - 0.03	0 (0)	-424.2 (15.32)	0.085 (0.051)	0 - 0.258
SWG	100	49	0.10	0.30	0.107 (0.011)	0.17 (0.095)	0.009 (0.009)	0 - 0.053	0 (0)	-422.35 (16.91)	0.145 (0.07)	0.001 - 0.283
SWG	100	49	0.10	0.40	0.106 (0.01)	0.157 (0.095)	0.008 (0.008)	0 - 0.036	0 (0)	-425.57 (17.34)	0.245 (0.09)	0.004 - 0.38
SWG	100	49	0.10	0.50	0.106 (0.011)	0.164 (0.108)	0.009 (0.009)	0 - 0.052	0 (0)	-426.14 (19.14)	0.336 (0.106)	0.033 - 0.497

Continued on next page

Table S1 – *Continued from previous page*

Structure	T	N	p	p_e/p_w	$\bar{\hat{p}}$ (SD)	$\overline{\hat{p_e}}/\overline{\hat{p_w}}$ (SD)	$\overline{\lvert\Delta\rvert}_p$ (SD)	Range $\lvert\Delta\rvert_p$	$\overline{SE}\lvert\Delta\rvert_p$ (SD)	$\overline{BIC}$ (SD)	$\overline{\lvert\Delta\rvert}_{p_e/p_w}$ (SD)	Range $\lvert\Delta\rvert_{p_e/p_w}$
SWG	100	49	0.10	0.60	0.106 (0.011)	0.171 (0.106)	0.01 (0.008)	0 - 0.036	0 (0)	-423.6 (16.17)	0.429 (0.106)	0.148 - 0.574
SWG	100	49	0.10	0.70	0.105 (0.011)	0.154 (0.096)	0.009 (0.008)	0 - 0.033	0 (0)	-425.8 (16.88)	0.546 (0.096)	0.268 - 0.674
SWG	100	49	0.10	0.80	0.108 (0.054)	0.146 (0.127)	0.015 (0.053)	0 - 0.529	0 (0)	-428.05 (18.73)	0.654 (0.127)	0.177 - 0.796
SWG	100	49	0.10	0.90	0.116 (0.081)	0.155 (0.14)	0.023 (0.079)	0 - 0.652	0 (0)	-431.41 (18.08)	0.745 (0.14)	0.136 - 0.886
SWG	100	49	0.20	0.10	0.212 (0.017)	0.226 (0.076)	0.016 (0.014)	0 - 0.052	0.001 (0)	-481.74 (13.04)	0.126 (0.075)	0.005 - 0.303
SWG	100	49	0.20	0.20	0.214 (0.022)	0.233 (0.1)	0.02 (0.018)	0 - 0.07	0.001 (0)	-484.45 (15.28)	0.08 (0.068)	0.001 - 0.255
SWG	100	49	0.20	0.30	0.212 (0.024)	0.24 (0.108)	0.019 (0.019)	0 - 0.063	0.001 (0)	-484.06 (15.84)	0.11 (0.055)	0.001 - 0.213
SWG	100	49	0.20	0.40	0.214 (0.024)	0.244 (0.105)	0.02 (0.019)	0 - 0.077	0.001 (0)	-481.9 (15.1)	0.163 (0.094)	0.002 - 0.306
SWG	100	49	0.20	0.50	0.211 (0.024)	0.242 (0.11)	0.021 (0.017)	0 - 0.072	0.001 (0)	-483.36 (16.41)	0.258 (0.11)	0.02 - 0.43
SWG	100	49	0.20	0.60	0.208 (0.024)	0.231 (0.108)	0.02 (0.016)	0 - 0.071	0.001 (0)	-482.81 (17.34)	0.369 (0.108)	0.052 - 0.564
SWG	100	49	0.20	0.70	0.216 (0.023)	0.238 (0.102)	0.023 (0.017)	0 - 0.066	0.001 (0)	-483.57 (16.02)	0.462 (0.102)	0.218 - 0.63
SWG	100	49	0.20	0.80	0.204 (0.026)	0.217 (0.116)	0.021 (0.015)	0 - 0.071	0.001 (0)	-482.36 (17.99)	0.583 (0.116)	0.268 - 0.738
SWG	100	49	0.20	0.90	0.205 (0.025)	0.214 (0.121)	0.021 (0.014)	0.001 - 0.058	0.001 (0)	-484.63 (16.49)	0.686 (0.121)	0.4 - 0.847
SWG	100	49	0.30	0.10	0.317 (0.026)	0.204 (0.049)	0.025 (0.018)	0 - 0.086	0.001 (0)	-507.4 (14.59)	0.104 (0.049)	0.024 - 0.212
SWG	100	49	0.30	0.20	0.309 (0.025)	0.196 (0.051)	0.02 (0.017)	0 - 0.077	0.001 (0)	-508.98 (17.18)	0.042 (0.029)	0 - 0.142
SWG	100	49	0.30	0.30	0.309 (0.024)	0.205 (0.053)	0.021 (0.014)	0 - 0.067	0.001 (0)	-509.22 (13.29)	0.097 (0.05)	0.001 - 0.195
SWG	100	49	0.30	0.40	0.31 (0.026)	0.206 (0.052)	0.022 (0.017)	0 - 0.074	0.001 (0)	-509.28 (14.63)	0.194 (0.052)	0.081 - 0.294
SWG	100	49	0.30	0.50	0.302 (0.024)	0.196 (0.05)	0.02 (0.014)	0 - 0.057	0.001 (0)	-510.36 (14.8)	0.304 (0.05)	0.176 - 0.406
SWG	100	49	0.30	0.60	0.301 (0.024)	0.185 (0.052)	0.019 (0.015)	0 - 0.075	0.001 (0)	-505.74 (15.01)	0.415 (0.052)	0.234 - 0.511
SWG	100	49	0.30	0.70	0.304 (0.024)	0.177 (0.046)	0.019 (0.015)	0 - 0.071	0.001 (0)	-510.1 (13.49)	0.523 (0.046)	0.389 - 0.602

Continued on next page

Table S1 – *Continued from previous page*

Structure	T	N	p	p_e/p_w	$\overline{\hat{p}}$ (SD)	$\overline{\hat{p_e}}/\overline{\hat{p_w}}$ (SD)	$\overline{\lvert\Delta\rvert}_p$ (SD)	Range $\lvert\Delta\rvert_p$	$\overline{SE}\lvert\Delta\rvert_p$ (SD)	$\overline{BIC}$ (SD)	$\overline{\lvert\Delta\rvert}_{p_e/p_w}$ (SD)	Range $\lvert\Delta\rvert_{p_e/p_w}$
SWG	100	49	0.30	0.80	0.302 (0.025)	0.188 (0.055)	0.021 (0.014)	0.001 - 0.062	0.001 (0)	-506.19 (14.79)	0.612 (0.055)	0.47 - 0.714
SWG	100	49	0.30	0.90	0.296 (0.024)	0.172 (0.051)	0.02 (0.013)	0.001 - 0.062	0.001 (0)	-506.62 (14.61)	0.728 (0.051)	0.573 - 0.821
SWG	100	49	0.40	0.10	0.403 (0.024)	0.106 (0.022)	0.018 (0.015)	0 - 0.081	0.002 (0)	-518.87 (14.54)	0.018 (0.015)	0 - 0.092
SWG	100	49	0.40	0.20	0.399 (0.025)	0.103 (0.02)	0.02 (0.016)	0.001 - 0.084	0.002 (0)	-515.05 (12.72)	0.097 (0.02)	0.046 - 0.138
SWG	100	49	0.40	0.30	0.402 (0.023)	0.111 (0.02)	0.019 (0.014)	0 - 0.061	0.002 (0)	-514.25 (12.52)	0.189 (0.02)	0.137 - 0.234
SWG	100	49	0.40	0.40	0.404 (0.024)	0.111 (0.02)	0.019 (0.015)	0 - 0.065	0.002 (0)	-519.25 (15.18)	0.289 (0.02)	0.238 - 0.345
SWG	100	49	0.40	0.50	0.395 (0.024)	0.103 (0.02)	0.02 (0.014)	0 - 0.056	0.002 (0)	-515.32 (13.27)	0.397 (0.02)	0.337 - 0.441
SWG	100	49	0.40	0.60	0.392 (0.024)	0.092 (0.019)	0.019 (0.016)	0 - 0.061	0.002 (0)	-515.9 (14.71)	0.508 (0.019)	0.466 - 0.55
SWG	100	49	0.40	0.70	0.394 (0.024)	0.09 (0.021)	0.02 (0.015)	0 - 0.065	0.002 (0)	-518.64 (14.18)	0.61 (0.021)	0.55 - 0.652
SWG	100	49	0.40	0.80	0.392 (0.026)	0.094 (0.021)	0.022 (0.015)	0 - 0.066	0.002 (0)	-516.94 (13.26)	0.706 (0.021)	0.657 - 0.752
SWG	100	49	0.40	0.90	0.388 (0.025)	0.079 (0.019)	0.022 (0.016)	0.001 - 0.063	0.002 (0)	-514.47 (13.31)	0.821 (0.019)	0.766 - 0.873
SWG	100	49	0.50	0.10	0.504 (0.028)	0.012 (0.008)	0.022 (0.016)	0.001 - 0.068	0.003 (0)	-518.62 (12.36)	0.088 (0.008)	0.064 - 0.1
SWG	100	49	0.50	0.20	0.504 (0.029)	0.013 (0.009)	0.023 (0.018)	0 - 0.084	0.003 (0)	-517.85 (13.43)	0.187 (0.009)	0.159 - 0.2
SWG	100	49	0.50	0.30	0.502 (0.027)	0.012 (0.009)	0.022 (0.015)	0 - 0.067	0.003 (0)	-516.08 (12.75)	0.288 (0.009)	0.262 - 0.3
SWG	100	49	0.50	0.40	0.503 (0.027)	0.012 (0.009)	0.022 (0.016)	0 - 0.066	0.003 (0)	-518.73 (13.67)	0.388 (0.009)	0.357 - 0.4
SWG	100	49	0.50	0.50	0.502 (0.025)	0.013 (0.009)	0.02 (0.014)	0 - 0.057	0.003 (0)	-520.37 (13.5)	0.487 (0.009)	0.464 - 0.5
SWG	100	49	0.50	0.60	0.501 (0.026)	0.013 (0.01)	0.021 (0.015)	0 - 0.075	0.003 (0)	-518.61 (13.31)	0.587 (0.01)	0.556 - 0.6
SWG	100	49	0.50	0.70	0.501 (0.03)	0.012 (0.01)	0.024 (0.018)	0 - 0.086	0.003 (0)	-517.74 (14.97)	0.688 (0.01)	0.647 - 0.699
SWG	100	49	0.50	0.80	0.503 (0.026)	0.011 (0.01)	0.02 (0.016)	0.001 - 0.062	0.003 (0)	-516.43 (13.24)	0.789 (0.01)	0.755 - 0.8
SWG	100	49	0.50	0.90	0.505 (0.033)	0.013 (0.01)	0.027 (0.019)	0 - 0.08	0.003 (0)	-516.96 (14.34)	0.887 (0.01)	0.856 - 0.9
SWG	100	49	0.60	0.10	0.618 (0.026)	0.102 (0.016)	0.026 (0.018)	0 - 0.07	0.002 (0)	-518.31 (13.76)	0.013 (0.01)	0 - 0.038
SWG	100	49	0.60	0.20	0.616 (0.024)	0.101 (0.019)	0.023 (0.017)	0 - 0.076	0.002 (0)	-517.12 (14.4)	0.099 (0.019)	0.043 - 0.15

Continued on next page

Table S1 – *Continued from previous page*

Structure	T	N	p	p_e/p_w	$\overline{\hat{p}}$ (SD)	$\overline{\hat{p}_e}/\overline{\hat{p}_w}$ (SD)	$\overline{\lvert\Delta\rvert}_p$ (SD)	Range $\lvert\Delta\rvert_p$	$\overline{SE}\lvert\Delta\rvert_p$ (SD)	$\overline{BIC}$ (SD)	$\overline{\lvert\Delta\rvert}_{p_e/p_w}$ (SD)	Range $\lvert\Delta\rvert_{p_e/p_w}$
SWG	100	49	0.60	0.30	0.623 (0.025)	0.111 (0.018)	0.027 (0.019)	0 - 0.081	0.002 (0)	-514.92 (12.76)	0.189 (0.018)	0.147 - 0.234
SWG	100	49	0.60	0.40	0.624 (0.029)	0.114 (0.018)	0.03 (0.022)	0 - 0.086	0.002 (0)	-516.51 (13.06)	0.286 (0.018)	0.238 - 0.318
SWG	100	49	0.60	0.50	0.628 (0.028)	0.105 (0.016)	0.033 (0.022)	0 - 0.103	0.002 (0)	-520.69 (14.32)	0.395 (0.016)	0.35 - 0.436
SWG	100	49	0.60	0.60	0.623 (0.025)	0.091 (0.017)	0.029 (0.019)	0.002 - 0.075	0.002 (0)	-515.31 (16.02)	0.509 (0.017)	0.47 - 0.55
SWG	100	49	0.60	0.70	0.626 (0.027)	0.088 (0.019)	0.031 (0.021)	0.001 - 0.105	0.002 (0)	-516.32 (14.8)	0.612 (0.019)	0.557 - 0.658
SWG	100	49	0.60	0.80	0.636 (0.029)	0.09 (0.019)	0.04 (0.024)	0.002 - 0.114	0.002 (0)	-515.36 (14.1)	0.71 (0.019)	0.665 - 0.755
SWG	100	49	0.60	0.90	0.632 (0.028)	0.085 (0.02)	0.036 (0.023)	0.003 - 0.096	0.002 (0)	-519.35 (13.23)	0.815 (0.02)	0.759 - 0.859
SWG	100	49	0.70	0.10	0.726 (0.025)	0.191 (0.017)	0.03 (0.019)	0.002 - 0.081	0.002 (0)	-509.56 (13.05)	0.091 (0.017)	0.053 - 0.14
SWG	100	49	0.70	0.20	0.729 (0.026)	0.192 (0.018)	0.032 (0.021)	0 - 0.085	0.002 (0)	-513.6 (13.52)	0.015 (0.012)	0 - 0.059
SWG	100	49	0.70	0.30	0.738 (0.026)	0.205 (0.017)	0.04 (0.022)	0.001 - 0.107	0.002 (0)	-513.33 (14.78)	0.095 (0.017)	0.051 - 0.133
SWG	100	49	0.70	0.40	0.732 (0.024)	0.203 (0.017)	0.034 (0.021)	0.003 - 0.08	0.002 (0)	-516.69 (14.15)	0.197 (0.017)	0.162 - 0.238
SWG	100	49	0.70	0.50	0.742 (0.021)	0.191 (0.016)	0.043 (0.019)	0.002 - 0.087	0.002 (0)	-518.09 (14.92)	0.309 (0.016)	0.271 - 0.358
SWG	100	49	0.70	0.60	0.745 (0.022)	0.175 (0.016)	0.045 (0.021)	0.004 - 0.092	0.002 (0)	-513.21 (12.93)	0.425 (0.016)	0.388 - 0.463
SWG	100	49	0.70	0.70	0.742 (0.021)	0.169 (0.016)	0.043 (0.019)	0 - 0.083	0.002 (0)	-513.56 (13.09)	0.531 (0.016)	0.488 - 0.572
SWG	100	49	0.70	0.80	0.756 (0.021)	0.169 (0.018)	0.056 (0.021)	0.005 - 0.104	0.002 (0)	-512.44 (14.01)	0.631 (0.018)	0.593 - 0.667
SWG	100	49	0.70	0.90	0.753 (0.019)	0.153 (0.014)	0.053 (0.019)	0.004 - 0.093	0.002 (0)	-515.14 (14.73)	0.747 (0.014)	0.715 - 0.792
SWG	100	49	0.80	0.10	0.811 (0.019)	0.23 (0.015)	0.018 (0.012)	0 - 0.052	0.001 (0)	-505.89 (14.7)	0.13 (0.015)	0.085 - 0.166
SWG	100	49	0.80	0.20	0.825 (0.015)	0.231 (0.015)	0.026 (0.013)	0.001 - 0.059	0.001 (0)	-511.56 (13.96)	0.031 (0.014)	0 - 0.066
SWG	100	49	0.80	0.30	0.836 (0.013)	0.241 (0.02)	0.036 (0.013)	0 - 0.062	0.001 (0)	-519.17 (18.63)	0.059 (0.02)	0.026 - 0.132

Continued on next page

Table S1 – *Continued from previous page*

Structure	T	N	p	p_e/p_w	$\overline{\hat{p}}$ (SD)	$\overline{\hat{p_e}}/\overline{\hat{p_w}}$ (SD)	$\overline{\lvert\Delta\rvert}_p$ (SD)	Range $\lvert\Delta\rvert_p$	$\overline{SE}\lvert\Delta\rvert_p$ (SD)	$\overline{BIC}$ (SD)	$\overline{\lvert\Delta\rvert}_{p_e/p_w}$ (SD)	Range $\lvert\Delta\rvert_{p_e/p_w}$
SWG	100	49	0.80	0.40	0.827 (0.014)	0.244 (0.016)	0.028 (0.013)	0.002 - 0.054	0.001 (0)	-517.23 (17.22)	0.156 (0.016)	0.123 - 0.196
SWG	100	49	0.80	0.50	0.842 (0.011)	0.207 (0.019)	0.042 (0.011)	0.001 - 0.069	0.001 (0)	-527.13 (16.39)	0.293 (0.019)	0.257 - 0.343
SWG	100	49	0.80	0.60	0.843 (0.009)	0.176 (0.023)	0.043 (0.009)	0.011 - 0.064	0.001 (0)	-515.48 (16.02)	0.424 (0.023)	0.362 - 0.478
SWG	100	49	0.80	0.70	0.835 (0.011)	0.176 (0.018)	0.035 (0.011)	0.005 - 0.056	0.001 (0)	-518.68 (16.58)	0.524 (0.018)	0.478 - 0.586
SWG	100	49	0.80	0.80	0.847 (0.008)	0.138 (0.017)	0.047 (0.008)	0.03 - 0.066	0.001 (0)	-511.71 (17.96)	0.662 (0.017)	0.615 - 0.692
SWG	100	49	0.80	0.90	0.844 (0.007)	0.132 (0.021)	0.044 (0.007)	0.023 - 0.06	0.001 (0)	-514.45 (17.67)	0.768 (0.021)	0.716 - 0.825
SWG	100	49	0.90	0.10	0.897 (0.011)	0.109 (0.034)	0.009 (0.008)	0 - 0.045	0.001 (0)	-490.73 (31.2)	0.026 (0.024)	0 - 0.122
SWG	100	49	0.90	0.20	0.907 (0.007)	0.077 (0.026)	0.009 (0.005)	0.001 - 0.021	0.001 (0)	-466.94 (35.2)	0.123 (0.026)	0.021 - 0.162
SWG	100	49	0.90	0.30	0.91 (0.006)	0.064 (0.013)	0.01 (0.006)	0 - 0.021	0.001 (0)	-460.16 (26.58)	0.236 (0.013)	0.205 - 0.263
SWG	100	49	0.90	0.40	0.909 (0.006)	0.077 (0.017)	0.009 (0.006)	0 - 0.026	0.001 (0)	-471.65 (31.2)	0.323 (0.017)	0.244 - 0.356
SWG	100	49	0.90	0.50	0.911 (0.005)	0.049 (0.012)	0.011 (0.005)	0 - 0.024	0 (0)	-444.64 (20.45)	0.451 (0.012)	0.421 - 0.479
SWG	100	49	0.90	0.60	0.911 (0.005)	0.036 (0.009)	0.011 (0.005)	0.001 - 0.028	0 (0)	-434.41 (16.6)	0.564 (0.009)	0.541 - 0.59
SWG	100	49	0.90	0.70	0.908 (0.006)	0.042 (0.01)	0.008 (0.005)	0 - 0.023	0 (0)	-438.76 (17.48)	0.658 (0.01)	0.637 - 0.684
SWG	100	49	0.90	0.80	0.912 (0.005)	0.029 (0.01)	0.012 (0.005)	0.001 - 0.024	0 (0)	-428.08 (17.93)	0.771 (0.01)	0.744 - 0.797
SWG	100	49	0.90	0.90	0.911 (0.005)	0.027 (0.01)	0.011 (0.005)	0 - 0.025	0 (0)	-428.82 (16.91)	0.873 (0.01)	0.843 - 0.893
SWG	100	100	0.10	0.10	0.116 (0.007)	0.225 (0.064)	0.016 (0.007)	0.001 - 0.04	0 (0)	-489.65 (12.88)	0.125 (0.063)	0.005 - 0.337
SWG	100	100	0.10	0.20	0.116 (0.009)	0.239 (0.081)	0.017 (0.009)	0 - 0.043	0 (0)	-488.02 (15.25)	0.067 (0.059)	0.001 - 0.287
SWG	100	100	0.10	0.30	0.112 (0.008)	0.199 (0.074)	0.012 (0.007)	0 - 0.033	0 (0)	-491.65 (15.07)	0.11 (0.061)	0 - 0.297
SWG	100	100	0.10	0.40	0.108 (0.009)	0.16 (0.088)	0.009 (0.008)	0 - 0.042	0 (0)	-493.99 (20.29)	0.242 (0.082)	0.025 - 0.387

Continued on next page

Table S1 – *Continued from previous page*

Structure	T	N	p	p_e/p_w	$\overline{\hat{p}}$ (SD)	$\overline{\hat{p_e}}/\overline{\hat{p_w}}$ (SD)	$\overline{\lvert\Delta\rvert}_p$ (SD)	Range $\lvert\Delta\rvert_p$	$\overline{SE}\lvert\Delta\rvert_p$ (SD)	$\overline{BIC}$ (SD)	$\overline{\lvert\Delta\rvert}_{p_e/p_w}$ (SD)	Range $\lvert\Delta\rvert_{p_e/p_w}$
SWG	100	100	0.10	0.50	0.108 (0.009)	0.169 (0.083)	0.009 (0.008)	0 - 0.038	0 (0)	-493.89 (17.55)	0.331 (0.083)	0.087 - 0.488
SWG	100	100	0.10	0.60	0.105 (0.009)	0.132 (0.081)	0.007 (0.007)	0 - 0.029	0 (0)	-503.64 (20.65)	0.468 (0.081)	0.228 - 0.593
SWG	100	100	0.10	0.70	0.105 (0.01)	0.141 (0.101)	0.008 (0.008)	0 - 0.037	0 (0)	-498.81 (21.21)	0.559 (0.101)	0.248 - 0.696
SWG	100	100	0.10	0.80	0.105 (0.015)	0.142 (0.104)	0.009 (0.013)	0 - 0.098	0 (0)	-504.46 (22.48)	0.658 (0.104)	0.343 - 0.761
SWG	100	100	0.10	0.90	0.104 (0.012)	0.128 (0.107)	0.009 (0.008)	0 - 0.038	0 (0)	-507.42 (27)	0.772 (0.107)	0.432 - 0.894
SWG	100	100	0.20	0.10	0.226 (0.015)	0.239 (0.062)	0.026 (0.015)	0.002 - 0.076	0 (0)	-550.3 (14.12)	0.139 (0.062)	0.011 - 0.384
SWG	100	100	0.20	0.20	0.228 (0.017)	0.269 (0.064)	0.028 (0.017)	0 - 0.092	0 (0)	-550.49 (14.61)	0.076 (0.056)	0.002 - 0.276
SWG	100	100	0.20	0.30	0.222 (0.019)	0.252 (0.076)	0.024 (0.016)	0 - 0.063	0 (0)	-553.99 (16.6)	0.074 (0.051)	0 - 0.216
SWG	100	100	0.20	0.40	0.216 (0.025)	0.236 (0.11)	0.022 (0.02)	0 - 0.087	0 (0)	-554.21 (16.33)	0.174 (0.094)	0.002 - 0.316
SWG	100	100	0.20	0.50	0.214 (0.022)	0.225 (0.097)	0.019 (0.018)	0 - 0.059	0 (0)	-553.49 (17.99)	0.275 (0.097)	0.067 - 0.395
SWG	100	100	0.20	0.60	0.212 (0.025)	0.218 (0.109)	0.021 (0.017)	0.001 - 0.07	0 (0)	-554.42 (16.71)	0.382 (0.109)	0.12 - 0.529
SWG	100	100	0.20	0.70	0.214 (0.027)	0.231 (0.116)	0.025 (0.017)	0.001 - 0.085	0 (0)	-551.17 (15.9)	0.469 (0.116)	0.155 - 0.645
SWG	100	100	0.20	0.80	0.208 (0.025)	0.202 (0.111)	0.022 (0.014)	0.002 - 0.068	0 (0)	-552.13 (16.94)	0.598 (0.111)	0.33 - 0.731
SWG	100	100	0.20	0.90	0.214 (0.031)	0.239 (0.136)	0.028 (0.019)	0.001 - 0.081	0 (0)	-554.36 (16.39)	0.661 (0.136)	0.384 - 0.857
SWG	100	100	0.30	0.10	0.326 (0.021)	0.187 (0.032)	0.028 (0.018)	0 - 0.089	0.001 (0)	-574.39 (13.97)	0.087 (0.032)	0.025 - 0.187
SWG	100	100	0.30	0.20	0.322 (0.02)	0.202 (0.037)	0.024 (0.017)	0.001 - 0.076	0.001 (0)	-578.48 (15.91)	0.029 (0.023)	0.001 - 0.128
SWG	100	100	0.30	0.30	0.316 (0.025)	0.198 (0.049)	0.023 (0.019)	0.001 - 0.089	0.001 (0)	-578.33 (15.17)	0.103 (0.047)	0.007 - 0.2
SWG	100	100	0.30	0.40	0.305 (0.025)	0.174 (0.047)	0.021 (0.015)	0.001 - 0.081	0.001 (0)	-578.87 (13.82)	0.226 (0.047)	0.085 - 0.319

Continued on next page

Table S1 – *Continued from previous page*

Structure	T	N	p	p_e/p_w	$\overline{\hat{p}}$ (SD)	$\overline{\hat{p}_e}/\overline{\hat{p}_w}$ (SD)	$\overline{\lvert\Delta\rvert}_p$ (SD)	Range $\lvert\Delta\rvert_p$	$\overline{SE}\lvert\Delta\rvert_p$ (SD)	$\overline{BIC}$ (SD)	$\overline{\lvert\Delta\rvert}_{p_e/p_w}$ (SD)	Range $\lvert\Delta\rvert_{p_e/p_w}$
SWG	100	100	0.30	0.50	0.308 (0.025)	0.181 (0.047)	0.021 (0.016)	0 - 0.076	0.001 (0)	-578.64 (16.04)	0.319 (0.047)	0.22 - 0.402
SWG	100	100	0.30	0.60	0.301 (0.025)	0.17 (0.045)	0.021 (0.014)	0 - 0.069	0.001 (0)	-581.45 (15.44)	0.43 (0.045)	0.308 - 0.519
SWG	100	100	0.30	0.70	0.3 (0.028)	0.174 (0.055)	0.024 (0.015)	0.002 - 0.073	0.001 (0)	-578.04 (15.12)	0.526 (0.055)	0.409 - 0.619
SWG	100	100	0.30	0.80	0.297 (0.027)	0.163 (0.052)	0.023 (0.013)	0 - 0.055	0.001 (0)	-581.38 (15.44)	0.637 (0.052)	0.515 - 0.727
SWG	100	100	0.30	0.90	0.295 (0.029)	0.171 (0.058)	0.026 (0.015)	0.002 - 0.061	0.001 (0)	-580.5 (12.97)	0.729 (0.058)	0.567 - 0.813
SWG	100	100	0.40	0.10	0.408 (0.022)	0.091 (0.016)	0.019 (0.014)	0.001 - 0.062	0.002 (0)	-586.61 (11.27)	0.015 (0.011)	0 - 0.044
SWG	100	100	0.40	0.20	0.406 (0.022)	0.099 (0.016)	0.017 (0.015)	0 - 0.065	0.002 (0)	-589.6 (14.16)	0.101 (0.016)	0.06 - 0.138
SWG	100	100	0.40	0.30	0.403 (0.024)	0.097 (0.017)	0.019 (0.014)	0 - 0.053	0.002 (0)	-586.43 (13.47)	0.203 (0.017)	0.16 - 0.233
SWG	100	100	0.40	0.40	0.393 (0.022)	0.089 (0.016)	0.018 (0.013)	0 - 0.066	0.002 (0)	-586.01 (15.6)	0.311 (0.016)	0.267 - 0.34
SWG	100	100	0.40	0.50	0.397 (0.021)	0.093 (0.016)	0.017 (0.013)	0 - 0.057	0.002 (0)	-587.59 (14.09)	0.407 (0.016)	0.368 - 0.448
SWG	100	100	0.40	0.60	0.389 (0.024)	0.084 (0.018)	0.023 (0.014)	0 - 0.06	0.002 (0)	-587.89 (14.22)	0.516 (0.018)	0.46 - 0.547
SWG	100	100	0.40	0.70	0.399 (0.022)	0.09 (0.016)	0.017 (0.013)	0.001 - 0.057	0.002 (0)	-586.33 (13.85)	0.61 (0.016)	0.562 - 0.645
SWG	100	100	0.40	0.80	0.389 (0.025)	0.081 (0.016)	0.022 (0.016)	0 - 0.082	0.002 (0)	-585.56 (13.92)	0.719 (0.016)	0.673 - 0.75
SWG	100	100	0.40	0.90	0.391 (0.023)	0.092 (0.019)	0.02 (0.015)	0 - 0.071	0.002 (0)	-586.06 (13.95)	0.808 (0.019)	0.759 - 0.854
SWG	100	100	0.50	0.10	0.507 (0.027)	0.007 (0.006)	0.022 (0.016)	0 - 0.08	0.003 (0)	-588.76 (13.61)	0.093 (0.006)	0.07 - 0.1
SWG	100	100	0.50	0.20	0.51 (0.026)	0.008 (0.007)	0.022 (0.018)	0 - 0.085	0.003 (0)	-587.39 (13.68)	0.192 (0.007)	0.166 - 0.2
SWG	100	100	0.50	0.30	0.502 (0.027)	0.007 (0.007)	0.023 (0.015)	0 - 0.068	0.003 (0)	-585.72 (13.8)	0.293 (0.007)	0.27 - 0.3
SWG	100	100	0.50	0.40	0.506 (0.022)	0.008 (0.007)	0.018 (0.013)	0 - 0.056	0.002 (0)	-587.28 (14.33)	0.392 (0.007)	0.371 - 0.4

Continued on next page

Table S1 – *Continued from previous page*

Structure	T	N	p	p_e/p_w	$\overline{\hat{p}}$ (SD)	$\overline{\hat{p}_e}/\overline{\hat{p}_w}$ (SD)	$\overline{\lvert\Delta\rvert}_p$ (SD)	Range $\lvert\Delta\rvert_p$	$\overline{SE}\lvert\Delta\rvert_p$ (SD)	$\overline{BIC}$ (SD)	$\overline{\lvert\Delta\rvert}_{p_e/p_w}$ (SD)	Range $\lvert\Delta\rvert_{p_e/p_w}$
SWG	100	100	0.50	0.50	0.5 (0.028)	0.008 (0.006)	0.023 (0.016)	0.001 - 0.081	0.002 (0)	-587.49 (14.68)	0.492 (0.006)	0.471 - 0.499
SWG	100	100	0.50	0.60	0.5 (0.022)	0.009 (0.007)	0.017 (0.013)	0 - 0.077	0.002 (0)	-587.09 (14.27)	0.591 (0.007)	0.563 - 0.6
SWG	100	100	0.50	0.70	0.499 (0.023)	0.009 (0.006)	0.018 (0.014)	0.001 - 0.066	0.002 (0)	-590.29 (15.66)	0.691 (0.006)	0.676 - 0.7
SWG	100	100	0.50	0.80	0.503 (0.023)	0.009 (0.007)	0.019 (0.014)	0.001 - 0.055	0.002 (0)	-587.05 (12.34)	0.791 (0.007)	0.772 - 0.8
SWG	100	100	0.50	0.90	0.501 (0.022)	0.008 (0.006)	0.018 (0.013)	0 - 0.056	0.002 (0)	-590.13 (14.69)	0.892 (0.006)	0.874 - 0.9
SWG	100	100	0.60	0.10	0.607 (0.03)	0.087 (0.013)	0.025 (0.018)	0 - 0.082	0.002 (0)	-587.27 (14.3)	0.015 (0.01)	0 - 0.047
SWG	100	100	0.60	0.20	0.613 (0.029)	0.097 (0.013)	0.026 (0.018)	0 - 0.074	0.002 (0)	-584.21 (13.14)	0.103 (0.013)	0.078 - 0.133
SWG	100	100	0.60	0.30	0.617 (0.026)	0.096 (0.013)	0.025 (0.018)	0 - 0.083	0.002 (0)	-586.4 (13.8)	0.204 (0.013)	0.167 - 0.23
SWG	100	100	0.60	0.40	0.617 (0.024)	0.091 (0.012)	0.025 (0.016)	0 - 0.068	0.002 (0)	-585.44 (12.4)	0.309 (0.012)	0.281 - 0.336
SWG	100	100	0.60	0.50	0.625 (0.025)	0.093 (0.012)	0.03 (0.019)	0.001 - 0.095	0.002 (0)	-585.47 (13.12)	0.407 (0.012)	0.373 - 0.433
SWG	100	100	0.60	0.60	0.629 (0.021)	0.089 (0.011)	0.03 (0.019)	0 - 0.076	0.002 (0)	-587.73 (14.54)	0.511 (0.011)	0.487 - 0.536
SWG	100	100	0.60	0.70	0.626 (0.028)	0.089 (0.012)	0.033 (0.019)	0.001 - 0.08	0.002 (0)	-585.05 (14.08)	0.611 (0.012)	0.584 - 0.639
SWG	100	100	0.60	0.80	0.629 (0.023)	0.083 (0.012)	0.032 (0.018)	0.001 - 0.08	0.002 (0)	-589.24 (12.23)	0.717 (0.012)	0.687 - 0.739
SWG	100	100	0.60	0.90	0.637 (0.025)	0.092 (0.011)	0.039 (0.021)	0 - 0.089	0.002 (0)	-585.99 (12.87)	0.808 (0.011)	0.776 - 0.839
SWG	100	100	0.70	0.10	0.715 (0.024)	0.163 (0.012)	0.023 (0.016)	0.001 - 0.072	0.002 (0)	-577.65 (12.44)	0.063 (0.012)	0.042 - 0.1
SWG	100	100	0.70	0.20	0.721 (0.026)	0.183 (0.014)	0.028 (0.018)	0 - 0.079	0.002 (0)	-577.23 (12.27)	0.018 (0.012)	0 - 0.047
SWG	100	100	0.70	0.30	0.728 (0.022)	0.185 (0.011)	0.031 (0.018)	0.001 - 0.07	0.002 (0)	-582.59 (13.27)	0.115 (0.011)	0.091 - 0.146

Continued on next page

Table S1 – *Continued from previous page*

Structure	T	N	p	p_e/p_w	$\overline{\hat{p}}$ (SD)	$\overline{\hat{p}_e}/\overline{\hat{p}_w}$ (SD)	$\overline{\|\Delta\|}_p$ (SD)	Range $\|\Delta\|_p$	$\overline{SE}\|\Delta\|_p$ (SD)	$\overline{BIC}$ (SD)	$\overline{\|\Delta\|}_{p_e/p_w}$ (SD)	Range $\|\Delta\|_{p_e/p_w}$
SWG	100	100	0.70	0.40	0.738 (0.023)	0.173 (0.013)	0.04 (0.019)	0.001 - 0.077	0.002 (0)	-583.6 (14.03)	0.227 (0.013)	0.203 - 0.259
SWG	100	100	0.70	0.50	0.738 (0.019)	0.179 (0.011)	0.038 (0.018)	0 - 0.085	0.002 (0)	-585.63 (13.08)	0.321 (0.011)	0.295 - 0.352
SWG	100	100	0.70	0.60	0.747 (0.018)	0.163 (0.011)	0.047 (0.018)	0.009 - 0.09	0.002 (0)	-585.18 (14.68)	0.437 (0.011)	0.412 - 0.466
SWG	100	100	0.70	0.70	0.751 (0.018)	0.168 (0.011)	0.051 (0.018)	0.001 - 0.099	0.001 (0)	-587.13 (13.83)	0.532 (0.011)	0.502 - 0.565
SWG	100	100	0.70	0.80	0.753 (0.016)	0.157 (0.012)	0.053 (0.016)	0.002 - 0.084	0.001 (0)	-585.14 (14.41)	0.643 (0.012)	0.621 - 0.664
SWG	100	100	0.70	0.90	0.76 (0.017)	0.169 (0.01)	0.06 (0.017)	0.021 - 0.099	0.001 (0)	-586.96 (17.32)	0.731 (0.01)	0.708 - 0.762
SWG	100	100	0.80	0.10	0.798 (0.021)	0.204 (0.01)	0.016 (0.014)	0 - 0.093	0.001 (0)	-568.21 (12.26)	0.104 (0.01)	0.068 - 0.13
SWG	100	100	0.80	0.20	0.811 (0.017)	0.231 (0.01)	0.017 (0.01)	0.001 - 0.038	0.001 (0)	-574.33 (15.95)	0.031 (0.01)	0.001 - 0.064
SWG	100	100	0.80	0.30	0.822 (0.015)	0.232 (0.011)	0.024 (0.012)	0 - 0.05	0.001 (0)	-583.38 (17.44)	0.068 (0.011)	0.046 - 0.106
SWG	100	100	0.80	0.40	0.837 (0.009)	0.187 (0.017)	0.037 (0.009)	0.011 - 0.053	0.001 (0)	-592.78 (19.37)	0.213 (0.017)	0.17 - 0.251
SWG	100	100	0.80	0.50	0.837 (0.01)	0.2 (0.017)	0.037 (0.01)	0.01 - 0.06	0.001 (0)	-595.06 (18.89)	0.3 (0.017)	0.263 - 0.346
SWG	100	100	0.80	0.60	0.843 (0.006)	0.154 (0.016)	0.043 (0.006)	0.028 - 0.06	0.001 (0)	-598.24 (21.24)	0.446 (0.016)	0.402 - 0.479
SWG	100	100	0.80	0.70	0.845 (0.006)	0.142 (0.018)	0.045 (0.006)	0.028 - 0.063	0.001 (0)	-592.98 (20.65)	0.558 (0.018)	0.512 - 0.6
SWG	100	100	0.80	0.80	0.845 (0.005)	0.132 (0.017)	0.045 (0.005)	0.033 - 0.063	0.001 (0)	-589.6 (20.76)	0.668 (0.017)	0.614 - 0.706
SWG	100	100	0.80	0.90	0.847 (0.005)	0.125 (0.015)	0.047 (0.005)	0.032 - 0.064	0.001 (0)	-591.39 (22.54)	0.775 (0.015)	0.733 - 0.807
SWG	100	100	0.90	0.10	0.869 (0.034)	0.119 (0.034)	0.031 (0.034)	0.001 - 0.133	0.001 (0)	-573.59 (30.07)	0.03 (0.024)	0 - 0.11
SWG	100	100	0.90	0.20	0.901 (0.008)	0.089 (0.03)	0.005 (0.006)	0 - 0.045	0 (0)	-577.71 (45.11)	0.112 (0.026)	0.001 - 0.144
SWG	100	100	0.90	0.30	0.905 (0.006)	0.061 (0.018)	0.007 (0.004)	0 - 0.024	0 (0)	-540.59 (32.51)	0.239 (0.018)	0.1 - 0.26
SWG	100	100	0.90	0.40	0.909 (0.004)	0.038 (0.007)	0.009 (0.004)	0.001 - 0.018	0 (0)	-510.96 (18.34)	0.362 (0.007)	0.348 - 0.376
SWG	100	100	0.90	0.50	0.909 (0.004)	0.042 (0.007)	0.009 (0.004)	0 - 0.02	0 (0)	-515.42 (20.5)	0.458 (0.007)	0.437 - 0.475

Continued on next page

Table S1 – *Continued from previous page*

Structure	T	N	p	p_e/p_w	$\overline{\hat{p}}$ (SD)	$\overline{\hat{p}_e}/\overline{\hat{p}_w}$ (SD)	$\overline{\lvert\Delta\rvert}_p$ (SD)	Range $\lvert\Delta\rvert_p$	$\overline{SE}\lvert\Delta\rvert_p$ (SD)	$\overline{BIC}$ (SD)	$\overline{\lvert\Delta\rvert}_{p_e/p_w}$ (SD)	Range $\lvert\Delta\rvert_{p_e/p_w}$
SWG	100	100	0.90	0.60	0.909 (0.004)	0.03 (0.007)	0.009 (0.004)	0.001 - 0.018	0 (0)	-504.05 (15.15)	0.57 (0.007)	0.555 - 0.584
SWG	100	100	0.90	0.70	0.909 (0.004)	0.027 (0.006)	0.009 (0.004)	0 - 0.018	0 (0)	-503.1 (15.73)	0.673 (0.006)	0.655 - 0.687
SWG	100	100	0.90	0.80	0.909 (0.003)	0.025 (0.006)	0.009 (0.003)	0 - 0.018	0 (0)	-502.53 (15.48)	0.775 (0.006)	0.761 - 0.788
SWG	100	100	0.90	0.90	0.91 (0.004)	0.024 (0.006)	0.01 (0.004)	0 - 0.018	0 (0)	-501.22 (17.13)	0.876 (0.006)	0.864 - 0.896
SWG	200	16	0.10	0.10	0.108 (0.012)	0.307 (0.14)	0.012 (0.008)	0 - 0.035	0 (0)	-613.3 (19.16)	0.214 (0.128)	0.002 - 0.595
SWG	200	16	0.10	0.20	0.107 (0.011)	0.28 (0.135)	0.01 (0.009)	0 - 0.04	0 (0)	-616.49 (20.79)	0.127 (0.091)	0.004 - 0.432
SWG	200	16	0.10	0.30	0.105 (0.01)	0.262 (0.124)	0.009 (0.007)	0 - 0.043	0 (0)	-616.45 (20.45)	0.106 (0.075)	0 - 0.273
SWG	200	16	0.10	0.40	0.107 (0.011)	0.287 (0.129)	0.011 (0.008)	0 - 0.032	0 (0)	-614.76 (19.16)	0.144 (0.093)	0 - 0.357
SWG	200	16	0.10	0.50	0.104 (0.013)	0.257 (0.159)	0.011 (0.008)	0 - 0.031	0 (0)	-618.27 (19.61)	0.247 (0.153)	0 - 0.494
SWG	200	16	0.10	0.60	0.106 (0.012)	0.256 (0.13)	0.01 (0.008)	0 - 0.04	0 (0)	-617.61 (20.42)	0.344 (0.129)	0.029 - 0.575
SWG	200	16	0.10	0.70	0.103 (0.011)	0.24 (0.138)	0.009 (0.007)	0 - 0.039	0 (0)	-617.08 (20.63)	0.46 (0.138)	0.126 - 0.685
SWG	200	16	0.10	0.80	0.102 (0.011)	0.226 (0.138)	0.009 (0.007)	0 - 0.028	0 (0)	-615.05 (22.58)	0.574 (0.138)	0.233 - 0.792
SWG	200	16	0.10	0.90	0.104 (0.011)	0.239 (0.136)	0.009 (0.007)	0 - 0.032	0 (0)	-618.8 (22.47)	0.661 (0.136)	0.307 - 0.878
SWG	200	16	0.20	0.10	0.222 (0.018)	0.422 (0.095)	0.024 (0.015)	0 - 0.063	0.001 (0)	-748.83 (22.36)	0.322 (0.095)	0.098 - 0.527
SWG	200	16	0.20	0.20	0.215 (0.016)	0.373 (0.091)	0.018 (0.012)	0 - 0.058	0.001 (0)	-747.31 (22.11)	0.176 (0.086)	0 - 0.373
SWG	200	16	0.20	0.30	0.217 (0.018)	0.367 (0.099)	0.02 (0.014)	0.001 - 0.07	0.001 (0)	-747.93 (20.37)	0.092 (0.075)	0.001 - 0.358
SWG	200	16	0.20	0.40	0.22 (0.017)	0.395 (0.088)	0.022 (0.014)	0 - 0.062	0.001 (0)	-746.52 (23.57)	0.072 (0.05)	0 - 0.198
SWG	200	16	0.20	0.50	0.213 (0.018)	0.343 (0.1)	0.018 (0.013)	0 - 0.057	0.001 (0)	-751.82 (19.48)	0.163 (0.089)	0.009 - 0.37
SWG	200	16	0.20	0.60	0.211 (0.015)	0.304 (0.091)	0.015 (0.01)	0 - 0.051	0.001 (0)	-750.05 (22.78)	0.296 (0.091)	0.035 - 0.526
SWG	200	16	0.20	0.70	0.208 (0.018)	0.31 (0.105)	0.015 (0.012)	0 - 0.052	0.001 (0)	-746.91 (18.7)	0.39 (0.105)	0.16 - 0.66
SWG	200	16	0.20	0.80	0.216 (0.018)	0.364 (0.1)	0.02 (0.013)	0 - 0.054	0.001 (0)	-748.65 (19.9)	0.436 (0.1)	0.224 - 0.7

Continued on next page

Table S1 – *Continued from previous page*

Structure	T	N	p	p_e/p_w	$\overline{\hat{p}}$ (SD)	$\overline{\hat{p_e}}/\overline{\hat{p_w}}$ (SD)	$\overline{\lvert\Delta\rvert}_p$ (SD)	Range $\lvert\Delta\rvert_p$	$\overline{SE}\lvert\Delta\rvert_p$ (SD)	$\overline{BIC}$ (SD)	$\overline{\lvert\Delta\rvert}_{p_e/p_w}$ (SD)	Range $\lvert\Delta\rvert_{p_e/p_w}$
SWG	200	16	0.20	0.90	0.21 (0.016)	0.3 (0.091)	0.015 (0.011)	0 - 0.068	0.001 (0)	-751.5 (17.72)	0.6 (0.091)	0.364 - 0.784
SWG	200	16	0.30	0.10	0.315 (0.017)	0.309 (0.042)	0.018 (0.013)	0 - 0.071	0.001 (0)	-800.93 (20.8)	0.209 (0.042)	0.103 - 0.322
SWG	200	16	0.30	0.20	0.31 (0.018)	0.278 (0.051)	0.016 (0.013)	0 - 0.051	0.001 (0)	-804.36 (18.2)	0.08 (0.048)	0.005 - 0.211
SWG	200	16	0.30	0.30	0.31 (0.017)	0.26 (0.044)	0.016 (0.011)	0 - 0.05	0.001 (0)	-803.9 (21.45)	0.049 (0.034)	0 - 0.16
SWG	200	16	0.30	0.40	0.311 (0.017)	0.277 (0.052)	0.015 (0.013)	0 - 0.066	0.001 (0)	-803.12 (19.23)	0.124 (0.05)	0.011 - 0.267
SWG	200	16	0.30	0.50	0.299 (0.018)	0.219 (0.046)	0.014 (0.012)	0 - 0.055	0.001 (0)	-809.07 (18.59)	0.281 (0.046)	0.129 - 0.379
SWG	200	16	0.30	0.60	0.298 (0.018)	0.199 (0.04)	0.015 (0.01)	0 - 0.044	0.001 (0)	-808.02 (19.18)	0.401 (0.04)	0.299 - 0.488
SWG	200	16	0.30	0.70	0.296 (0.017)	0.207 (0.046)	0.014 (0.011)	0 - 0.051	0.001 (0)	-806.19 (20.17)	0.493 (0.046)	0.371 - 0.587
SWG	200	16	0.30	0.80	0.302 (0.016)	0.235 (0.044)	0.013 (0.01)	0 - 0.044	0.001 (0)	-802.58 (21.16)	0.565 (0.044)	0.461 - 0.65
SWG	200	16	0.30	0.90	0.3 (0.018)	0.204 (0.045)	0.015 (0.011)	0 - 0.043	0.001 (0)	-807.55 (20.66)	0.696 (0.045)	0.516 - 0.797
SWG	200	16	0.40	0.10	0.4 (0.021)	0.146 (0.027)	0.016 (0.014)	0 - 0.065	0.001 (0)	-827.94 (20.16)	0.047 (0.025)	0.002 - 0.116
SWG	200	16	0.40	0.20	0.399 (0.018)	0.132 (0.027)	0.014 (0.01)	0 - 0.046	0.001 (0)	-820.79 (19.35)	0.068 (0.026)	0.004 - 0.126
SWG	200	16	0.40	0.30	0.4 (0.016)	0.123 (0.031)	0.012 (0.01)	0 - 0.046	0.001 (0)	-823.3 (17.02)	0.177 (0.031)	0.076 - 0.246
SWG	200	16	0.40	0.40	0.398 (0.019)	0.134 (0.033)	0.014 (0.012)	0 - 0.051	0.001 (0)	-823.26 (19.8)	0.266 (0.033)	0.179 - 0.34
SWG	200	16	0.40	0.50	0.388 (0.02)	0.108 (0.034)	0.018 (0.015)	0 - 0.052	0.001 (0)	-822.83 (20.91)	0.392 (0.034)	0.264 - 0.472
SWG	200	16	0.40	0.60	0.395 (0.017)	0.098 (0.029)	0.014 (0.01)	0 - 0.052	0.001 (0)	-824 (18.47)	0.502 (0.029)	0.425 - 0.56
SWG	200	16	0.40	0.70	0.387 (0.019)	0.1 (0.032)	0.019 (0.013)	0 - 0.053	0.001 (0)	-822.51 (17.33)	0.6 (0.032)	0.495 - 0.684
SWG	200	16	0.40	0.80	0.393 (0.018)	0.113 (0.03)	0.015 (0.012)	0 - 0.048	0.001 (0)	-821.59 (17.81)	0.687 (0.03)	0.591 - 0.752
SWG	200	16	0.40	0.90	0.392 (0.018)	0.099 (0.027)	0.015 (0.012)	0 - 0.052	0.001 (0)	-820.15 (17.64)	0.801 (0.027)	0.736 - 0.873
SWG	200	16	0.50	0.10	0.504 (0.02)	0.02 (0.017)	0.016 (0.012)	0 - 0.068	0.001 (0)	-828.52 (21.55)	0.08 (0.017)	0.016 - 0.1

Continued on next page

Table S1 – *Continued from previous page*

Structure	T	N	p	p_e/p_w	$\overline{\hat{p}}$ (SD)	$\overline{\hat{p_e}}/\overline{\hat{p_w}}$ (SD)	$\overline{\lvert\Delta\rvert}_p$ (SD)	Range $\lvert\Delta\rvert_p$	$\overline{SE}\lvert\Delta\rvert_p$ (SD)	$\overline{BIC}$ (SD)	$\overline{\lvert\Delta\rvert}_{p_e/p_w}$ (SD)	Range $\lvert\Delta\rvert_{p_e/p_w}$
SWG	200	16	0.50	0.20	0.497 (0.023)	0.022 (0.016)	0.018 (0.014)	0 - 0.061	0.001 (0)	-827.94 (17.51)	0.178 (0.016)	0.127 - 0.199
SWG	200	16	0.50	0.30	0.498 (0.023)	0.02 (0.015)	0.018 (0.014)	0 - 0.072	0.001 (0)	-825.14 (18.65)	0.28 (0.015)	0.219 - 0.3
SWG	200	16	0.50	0.40	0.5 (0.023)	0.021 (0.015)	0.018 (0.014)	0 - 0.058	0.001 (0)	-828.32 (19.34)	0.379 (0.015)	0.344 - 0.4
SWG	200	16	0.50	0.50	0.503 (0.023)	0.022 (0.016)	0.018 (0.014)	0 - 0.062	0.001 (0)	-828.01 (17.77)	0.478 (0.016)	0.434 - 0.499
SWG	200	16	0.50	0.60	0.5 (0.023)	0.02 (0.015)	0.019 (0.013)	0 - 0.052	0.001 (0)	-827.12 (19.17)	0.58 (0.015)	0.521 - 0.599
SWG	200	16	0.50	0.70	0.497 (0.021)	0.023 (0.018)	0.017 (0.013)	0.001 - 0.059	0.001 (0)	-828.66 (20.85)	0.677 (0.018)	0.624 - 0.699
SWG	200	16	0.50	0.80	0.505 (0.018)	0.022 (0.016)	0.015 (0.012)	0 - 0.05	0.001 (0)	-830.38 (17.39)	0.778 (0.016)	0.721 - 0.8
SWG	200	16	0.50	0.90	0.505 (0.021)	0.021 (0.016)	0.018 (0.012)	0 - 0.063	0.001 (0)	-825.67 (17.14)	0.879 (0.016)	0.816 - 0.9
SWG	200	16	0.60	0.10	0.624 (0.024)	0.142 (0.031)	0.029 (0.018)	0 - 0.092	0.001 (0)	-826.46 (19.45)	0.045 (0.027)	0 - 0.118
SWG	200	16	0.60	0.20	0.621 (0.021)	0.129 (0.026)	0.025 (0.016)	0 - 0.073	0.001 (0)	-825.97 (19.17)	0.071 (0.026)	0.018 - 0.139
SWG	200	16	0.60	0.30	0.616 (0.02)	0.122 (0.026)	0.021 (0.015)	0 - 0.063	0.001 (0)	-826.83 (20.47)	0.178 (0.026)	0.106 - 0.235
SWG	200	16	0.60	0.40	0.618 (0.021)	0.131 (0.026)	0.023 (0.015)	0 - 0.068	0.001 (0)	-822.43 (19.53)	0.269 (0.026)	0.193 - 0.348
SWG	200	16	0.60	0.50	0.627 (0.021)	0.106 (0.027)	0.029 (0.017)	0.002 - 0.083	0.001 (0)	-824.62 (21.58)	0.394 (0.027)	0.336 - 0.449
SWG	200	16	0.60	0.60	0.62 (0.022)	0.1 (0.028)	0.024 (0.017)	0 - 0.074	0.001 (0)	-825.32 (19.66)	0.5 (0.028)	0.426 - 0.559
SWG	200	16	0.60	0.70	0.624 (0.021)	0.096 (0.026)	0.027 (0.018)	0 - 0.065	0.001 (0)	-820.11 (16.78)	0.604 (0.026)	0.525 - 0.665
SWG	200	16	0.60	0.80	0.626 (0.021)	0.112 (0.026)	0.028 (0.019)	0.001 - 0.075	0.001 (0)	-823.55 (18.29)	0.688 (0.026)	0.62 - 0.747
SWG	200	16	0.60	0.90	0.622 (0.021)	0.099 (0.026)	0.025 (0.017)	0 - 0.073	0.001 (0)	-826.13 (20.13)	0.801 (0.026)	0.742 - 0.869
SWG	200	16	0.70	0.10	0.737 (0.018)	0.246 (0.025)	0.037 (0.017)	0.001 - 0.071	0.001 (0)	-819.55 (19.2)	0.146 (0.025)	0.087 - 0.2
SWG	200	16	0.70	0.20	0.739 (0.018)	0.23 (0.025)	0.039 (0.018)	0.002 - 0.085	0.001 (0)	-812.28 (17.81)	0.032 (0.023)	0 - 0.091

Continued on next page

Table S1 – *Continued from previous page*

Structure	T	N	p	p_e/p_w	$\overline{\hat{p}}$ (SD)	$\overline{\hat{p_e}}/\overline{\hat{p_w}}$ (SD)	$\overline{\lvert\Delta\rvert}_p$ (SD)	Range $\lvert\Delta\rvert_p$	$\overline{SE}\lvert\Delta\rvert_p$ (SD)	$\overline{BIC}$ (SD)	$\overline{\lvert\Delta\rvert}_{p_e/p_w}$ (SD)	Range $\lvert\Delta\rvert_{p_e/p_w}$
SWG	200	16	0.70	0.30	0.732 (0.018)	0.228 (0.025)	0.033 (0.017)	0 - 0.072	0.001 (0)	-811.22 (19.71)	0.072 (0.025)	0.004 - 0.129
SWG	200	16	0.70	0.40	0.729 (0.021)	0.231 (0.025)	0.031 (0.018)	0 - 0.07	0.001 (0)	-818.23 (18.06)	0.169 (0.025)	0.113 - 0.22
SWG	200	16	0.70	0.50	0.738 (0.019)	0.187 (0.027)	0.038 (0.019)	0 - 0.083	0.001 (0)	-820.15 (20.77)	0.313 (0.027)	0.254 - 0.401
SWG	200	16	0.70	0.60	0.729 (0.018)	0.184 (0.024)	0.03 (0.016)	0 - 0.069	0.001 (0)	-818.43 (19.58)	0.416 (0.024)	0.358 - 0.467
SWG	200	16	0.70	0.70	0.745 (0.017)	0.17 (0.024)	0.045 (0.017)	0.002 - 0.085	0.001 (0)	-816.67 (17.8)	0.53 (0.024)	0.465 - 0.584
SWG	200	16	0.70	0.80	0.742 (0.018)	0.196 (0.025)	0.042 (0.018)	0 - 0.079	0.001 (0)	-818.96 (17.38)	0.604 (0.025)	0.523 - 0.65
SWG	200	16	0.70	0.90	0.735 (0.019)	0.17 (0.024)	0.036 (0.017)	0.002 - 0.075	0.001 (0)	-821.69 (20.37)	0.73 (0.024)	0.672 - 0.785
SWG	200	16	0.80	0.10	0.837 (0.012)	0.271 (0.025)	0.037 (0.012)	0.007 - 0.074	0.001 (0)	-811.26 (21.62)	0.171 (0.025)	0.094 - 0.229
SWG	200	16	0.80	0.20	0.832 (0.012)	0.246 (0.026)	0.032 (0.012)	0.001 - 0.066	0.001 (0)	-805.01 (23.64)	0.047 (0.025)	0 - 0.114
SWG	200	16	0.80	0.30	0.827 (0.014)	0.271 (0.024)	0.027 (0.013)	0.001 - 0.055	0.001 (0)	-807.02 (19.35)	0.032 (0.019)	0 - 0.081
SWG	200	16	0.80	0.40	0.823 (0.015)	0.267 (0.025)	0.024 (0.013)	0.003 - 0.052	0.001 (0)	-806.2 (21.08)	0.133 (0.025)	0.076 - 0.203
SWG	200	16	0.80	0.50	0.837 (0.012)	0.191 (0.026)	0.037 (0.012)	0.005 - 0.064	0.001 (0)	-804.55 (22.34)	0.309 (0.026)	0.227 - 0.355
SWG	200	16	0.80	0.60	0.83 (0.014)	0.203 (0.024)	0.03 (0.013)	0.001 - 0.061	0.001 (0)	-792.98 (21.2)	0.397 (0.024)	0.34 - 0.457
SWG	200	16	0.80	0.70	0.838 (0.01)	0.165 (0.026)	0.038 (0.01)	0.005 - 0.06	0.001 (0)	-789.3 (21.6)	0.535 (0.026)	0.469 - 0.593
SWG	200	16	0.80	0.80	0.838 (0.012)	0.202 (0.029)	0.038 (0.012)	0.002 - 0.062	0.001 (0)	-803.43 (20.28)	0.598 (0.029)	0.535 - 0.684
SWG	200	16	0.80	0.90	0.832 (0.013)	0.19 (0.028)	0.032 (0.013)	0.001 - 0.061	0.001 (0)	-809.89 (19.62)	0.71 (0.028)	0.637 - 0.766
SWG	200	16	0.90	0.10	0.915 (0.007)	0.105 (0.026)	0.015 (0.006)	0.001 - 0.032	0 (0)	-711.51 (36.81)	0.021 (0.016)	0 - 0.065
SWG	200	16	0.90	0.20	0.914 (0.009)	0.116 (0.038)	0.015 (0.007)	0 - 0.039	0 (0)	-722.13 (45.01)	0.085 (0.036)	0.005 - 0.15
SWG	200	16	0.90	0.30	0.915 (0.008)	0.132 (0.034)	0.015 (0.007)	0 - 0.033	0 (0)	-728.53 (44.95)	0.168 (0.034)	0.068 - 0.257

Continued on next page

Table S1 – *Continued from previous page*

Structure	T	N	p	p_e/p_w	$\overline{\hat{p}}$ (SD)	$\overline{\hat{p}_e}/\overline{\hat{p}_w}$ (SD)	$\overline{\lvert\Delta\rvert}_p$ (SD)	Range $\lvert\Delta\rvert_p$	$\overline{SE}\lvert\Delta\rvert_p$ (SD)	$\overline{BIC}$ (SD)	$\overline{\lvert\Delta\rvert}_{p_e/p_w}$ (SD)	Range $\lvert\Delta\rvert_{p_e/p_w}$
SWG	200	16	0.90	0.40	0.906 (0.01)	0.151 (0.03)	0.009 (0.006)	0 - 0.026	0.001 (0)	-741.11 (30.34)	0.249 (0.03)	0.175 - 0.32
SWG	200	16	0.90	0.50	0.915 (0.007)	0.062 (0.022)	0.015 (0.007)	0.001 - 0.03	0 (0)	-672.82 (35.2)	0.438 (0.022)	0.35 - 0.488
SWG	200	16	0.90	0.60	0.912 (0.007)	0.083 (0.023)	0.012 (0.007)	0.001 - 0.027	0 (0)	-687.07 (30.74)	0.517 (0.023)	0.458 - 0.576
SWG	200	16	0.90	0.70	0.914 (0.007)	0.047 (0.017)	0.014 (0.007)	0.001 - 0.033	0 (0)	-649.3 (24.47)	0.653 (0.017)	0.592 - 0.698
SWG	200	16	0.90	0.80	0.915 (0.007)	0.064 (0.021)	0.015 (0.006)	0.001 - 0.032	0 (0)	-671.72 (37.07)	0.736 (0.021)	0.665 - 0.777
SWG	200	16	0.90	0.90	0.913 (0.007)	0.077 (0.029)	0.013 (0.006)	0.001 - 0.025	0 (0)	-694.69 (39.76)	0.823 (0.029)	0.716 - 0.874
SWG	200	25	0.10	0.10	0.107 (0.01)	0.222 (0.099)	0.009 (0.008)	0 - 0.042	0 (0)	-712.45 (20.32)	0.126 (0.093)	0 - 0.475
SWG	200	25	0.10	0.20	0.106 (0.009)	0.211 (0.098)	0.008 (0.007)	0 - 0.037	0 (0)	-710.43 (22.15)	0.081 (0.055)	0.004 - 0.285
SWG	200	25	0.10	0.30	0.107 (0.009)	0.214 (0.101)	0.009 (0.007)	0 - 0.035	0 (0)	-712.5 (26.42)	0.114 (0.068)	0.003 - 0.293
SWG	200	25	0.10	0.40	0.106 (0.01)	0.208 (0.099)	0.008 (0.008)	0 - 0.034	0 (0)	-710.41 (21.02)	0.195 (0.093)	0.004 - 0.364
SWG	200	25	0.10	0.50	0.108 (0.011)	0.239 (0.118)	0.011 (0.008)	0 - 0.034	0 (0)	-710.9 (21.54)	0.261 (0.118)	0.001 - 0.474
SWG	200	25	0.10	0.60	0.104 (0.011)	0.195 (0.115)	0.009 (0.007)	0 - 0.032	0 (0)	-710.09 (20.57)	0.405 (0.115)	0.099 - 0.581
SWG	200	25	0.10	0.70	0.103 (0.011)	0.168 (0.126)	0.009 (0.007)	0 - 0.034	0 (0)	-717.73 (19.07)	0.532 (0.126)	0.24 - 0.697
SWG	200	25	0.10	0.80	0.104 (0.01)	0.18 (0.116)	0.009 (0.007)	0.001 - 0.029	0 (0)	-715.49 (22.27)	0.62 (0.116)	0.332 - 0.776
SWG	200	25	0.10	0.90	0.104 (0.011)	0.194 (0.127)	0.009 (0.008)	0 - 0.038	0 (0)	-714.69 (24.84)	0.706 (0.127)	0.4 - 0.894
SWG	200	25	0.20	0.10	0.218 (0.017)	0.307 (0.084)	0.02 (0.015)	0 - 0.062	0 (0)	-842.11 (19.67)	0.207 (0.084)	0.039 - 0.424
SWG	200	25	0.20	0.20	0.219 (0.017)	0.293 (0.073)	0.02 (0.015)	0.001 - 0.064	0 (0)	-840.2 (22.8)	0.097 (0.068)	0.002 - 0.256
SWG	200	25	0.20	0.30	0.217 (0.016)	0.304 (0.08)	0.019 (0.014)	0 - 0.062	0 (0)	-838.4 (20.55)	0.066 (0.046)	0.002 - 0.209
SWG	200	25	0.20	0.40	0.22 (0.017)	0.314 (0.089)	0.022 (0.015)	0.001 - 0.072	0 (0)	-835.79 (18.61)	0.102 (0.07)	0 - 0.255

Continued on next page

Table S1 – *Continued from previous page*

Structure	T	N	p	p_e/p_w	$\overline{\hat{p}}$ (SD)	$\overline{\hat{p_e}}/\overline{\hat{p_w}}$ (SD)	$\overline{\lvert\Delta\rvert}_p$ (SD)	Range $\lvert\Delta\rvert_p$	$\overline{SE}\lvert\Delta\rvert_p$ (SD)	$\overline{BIC}$ (SD)	$\overline{\lvert\Delta\rvert}_{p_e/p_w}$ (SD)	Range $\lvert\Delta\rvert_{p_e/p_w}$
SWG	200	25	0.20	0.50	0.218 (0.019)	0.324 (0.096)	0.021 (0.015)	0 - 0.052	0 (0)	-841.04 (21.88)	0.176 (0.095)	0.007 - 0.363
SWG	200	25	0.20	0.60	0.213 (0.016)	0.281 (0.082)	0.016 (0.012)	0 - 0.045	0 (0)	-839.96 (22.39)	0.319 (0.082)	0.128 - 0.497
SWG	200	25	0.20	0.70	0.215 (0.02)	0.295 (0.105)	0.02 (0.014)	0 - 0.074	0 (0)	-839.48 (19.96)	0.405 (0.105)	0.155 - 0.655
SWG	200	25	0.20	0.80	0.214 (0.019)	0.272 (0.093)	0.019 (0.014)	0 - 0.061	0 (0)	-842.04 (20.58)	0.528 (0.093)	0.296 - 0.743
SWG	200	25	0.20	0.90	0.213 (0.019)	0.283 (0.098)	0.019 (0.014)	0 - 0.057	0 (0)	-841.77 (22.8)	0.617 (0.098)	0.419 - 0.859
SWG	200	25	0.30	0.10	0.317 (0.017)	0.247 (0.041)	0.019 (0.014)	0 - 0.067	0.001 (0)	-890.98 (22.26)	0.147 (0.041)	0.052 - 0.248
SWG	200	25	0.30	0.20	0.314 (0.017)	0.221 (0.036)	0.017 (0.014)	0 - 0.061	0.001 (0)	-891.54 (20.22)	0.032 (0.027)	0.001 - 0.118
SWG	200	25	0.30	0.30	0.308 (0.014)	0.22 (0.035)	0.013 (0.01)	0 - 0.052	0.001 (0)	-891.19 (19.6)	0.081 (0.034)	0.001 - 0.163
SWG	200	25	0.30	0.40	0.314 (0.016)	0.23 (0.039)	0.018 (0.012)	0.001 - 0.058	0.001 (0)	-892.79 (19.68)	0.17 (0.039)	0.088 - 0.268
SWG	200	25	0.30	0.50	0.31 (0.016)	0.241 (0.037)	0.015 (0.012)	0 - 0.048	0.001 (0)	-893.86 (21.73)	0.259 (0.037)	0.162 - 0.353
SWG	200	25	0.30	0.60	0.306 (0.017)	0.204 (0.036)	0.015 (0.011)	0 - 0.053	0.001 (0)	-891.18 (22.75)	0.396 (0.036)	0.296 - 0.467
SWG	200	25	0.30	0.70	0.304 (0.018)	0.208 (0.042)	0.015 (0.011)	0 - 0.063	0.001 (0)	-896.39 (19.89)	0.492 (0.042)	0.393 - 0.576
SWG	200	25	0.30	0.80	0.301 (0.015)	0.192 (0.036)	0.012 (0.009)	0 - 0.037	0.001 (0)	-895.42 (20.55)	0.608 (0.036)	0.518 - 0.689
SWG	200	25	0.30	0.90	0.3 (0.015)	0.19 (0.04)	0.012 (0.01)	0 - 0.043	0.001 (0)	-899.78 (24.31)	0.71 (0.04)	0.53 - 0.784
SWG	200	25	0.40	0.10	0.402 (0.017)	0.124 (0.025)	0.014 (0.01)	0 - 0.039	0.001 (0)	-914.54 (19.31)	0.028 (0.021)	0 - 0.083
SWG	200	25	0.40	0.20	0.401 (0.016)	0.114 (0.022)	0.013 (0.01)	0 - 0.047	0.001 (0)	-913.59 (17.65)	0.086 (0.022)	0.035 - 0.137
SWG	200	25	0.40	0.30	0.395 (0.017)	0.107 (0.021)	0.014 (0.011)	0 - 0.045	0.001 (0)	-914.07 (20.64)	0.193 (0.021)	0.133 - 0.241
SWG	200	25	0.40	0.40	0.398 (0.02)	0.112 (0.024)	0.016 (0.013)	0 - 0.059	0.001 (0)	-911.1 (20.14)	0.288 (0.024)	0.211 - 0.35
SWG	200	25	0.40	0.50	0.399 (0.019)	0.119 (0.024)	0.016 (0.011)	0 - 0.052	0.001 (0)	-910.91 (17.44)	0.381 (0.024)	0.263 - 0.421

Continued on next page

Table S1 – *Continued from previous page*

Structure	T	N	p	p_e/p_w	$\overline{\hat{p}}$ (SD)	$\overline{\hat{p}_e}/\overline{\hat{p}_w}$ (SD)	$\overline{\lvert\Delta\rvert}_p$ (SD)	Range $\lvert\Delta\rvert_p$	$\overline{SE}\lvert\Delta\rvert_p$ (SD)	$\overline{BIC}$ (SD)	$\overline{\lvert\Delta\rvert}_{p_e/p_w}$ (SD)	Range $\lvert\Delta\rvert_{p_e/p_w}$
SWG	200	25	0.40	0.60	0.394 (0.018)	0.099 (0.02)	0.015 (0.012)	0 - 0.053	0.001 (0)	-913.76 (19.76)	0.501 (0.02)	0.452 - 0.554
SWG	200	25	0.40	0.70	0.392 (0.02)	0.096 (0.019)	0.017 (0.012)	0 - 0.068	0.001 (0)	-913.25 (18.53)	0.604 (0.019)	0.567 - 0.652
SWG	200	25	0.40	0.80	0.387 (0.018)	0.085 (0.018)	0.019 (0.013)	0 - 0.053	0.001 (0)	-914.38 (22.33)	0.715 (0.018)	0.659 - 0.752
SWG	200	25	0.40	0.90	0.389 (0.017)	0.088 (0.02)	0.017 (0.011)	0 - 0.053	0.001 (0)	-911.33 (20.38)	0.812 (0.02)	0.749 - 0.873
SWG	200	25	0.50	0.10	0.499 (0.02)	0.015 (0.012)	0.016 (0.013)	0 - 0.055	0.001 (0)	-923.25 (21.35)	0.085 (0.012)	0.038 - 0.1
SWG	200	25	0.50	0.20	0.499 (0.021)	0.013 (0.009)	0.016 (0.013)	0 - 0.059	0.001 (0)	-919.29 (22.12)	0.187 (0.009)	0.161 - 0.2
SWG	200	25	0.50	0.30	0.504 (0.02)	0.014 (0.01)	0.017 (0.012)	0 - 0.057	0.001 (0)	-913.12 (18.45)	0.286 (0.01)	0.245 - 0.3
SWG	200	25	0.50	0.40	0.501 (0.019)	0.013 (0.011)	0.015 (0.011)	0 - 0.053	0.001 (0)	-913.48 (19.8)	0.387 (0.011)	0.341 - 0.4
SWG	200	25	0.50	0.50	0.503 (0.022)	0.013 (0.01)	0.017 (0.014)	0 - 0.063	0.001 (0)	-917.66 (22.01)	0.487 (0.01)	0.442 - 0.499
SWG	200	25	0.50	0.60	0.501 (0.018)	0.015 (0.01)	0.014 (0.011)	0 - 0.06	0.001 (0)	-915.56 (16.26)	0.585 (0.01)	0.555 - 0.6
SWG	200	25	0.50	0.70	0.498 (0.021)	0.014 (0.009)	0.016 (0.013)	0 - 0.074	0.001 (0)	-918.09 (18.91)	0.686 (0.009)	0.656 - 0.7
SWG	200	25	0.50	0.80	0.5 (0.02)	0.015 (0.01)	0.015 (0.013)	0 - 0.053	0.001 (0)	-917.69 (20.49)	0.785 (0.01)	0.756 - 0.8
SWG	200	25	0.50	0.90	0.504 (0.019)	0.013 (0.01)	0.015 (0.012)	0 - 0.06	0.001 (0)	-916.28 (19.06)	0.887 (0.01)	0.86 - 0.9
SWG	200	25	0.60	0.10	0.613 (0.018)	0.114 (0.02)	0.018 (0.013)	0 - 0.066	0.001 (0)	-914.96 (18.85)	0.02 (0.015)	0 - 0.067
SWG	200	25	0.60	0.20	0.616 (0.019)	0.105 (0.019)	0.02 (0.014)	0 - 0.065	0.001 (0)	-915.03 (19.86)	0.095 (0.019)	0.042 - 0.138
SWG	200	25	0.60	0.30	0.623 (0.023)	0.108 (0.018)	0.026 (0.019)	0 - 0.077	0.001 (0)	-913 (20.09)	0.192 (0.018)	0.145 - 0.222
SWG	200	25	0.60	0.40	0.621 (0.023)	0.111 (0.021)	0.025 (0.018)	0 - 0.073	0.001 (0)	-912.25 (16.88)	0.289 (0.021)	0.235 - 0.337
SWG	200	25	0.60	0.50	0.623 (0.02)	0.114 (0.018)	0.025 (0.017)	0 - 0.075	0.001 (0)	-918.93 (18.65)	0.386 (0.018)	0.346 - 0.43
SWG	200	25	0.60	0.60	0.624 (0.021)	0.099 (0.021)	0.027 (0.018)	0 - 0.07	0.001 (0)	-912.08 (15.92)	0.501 (0.021)	0.454 - 0.564
SWG	200	25	0.60	0.70	0.626 (0.02)	0.087 (0.02)	0.028 (0.018)	0.001 - 0.084	0.001 (0)	-914.62 (20.11)	0.613 (0.02)	0.569 - 0.67
SWG	200	25	0.60	0.80	0.628 (0.019)	0.089 (0.017)	0.029 (0.017)	0 - 0.071	0.001 (0)	-917.17 (21.72)	0.711 (0.017)	0.652 - 0.748
SWG	200	25	0.60	0.90	0.63 (0.021)	0.088 (0.019)	0.032 (0.018)	0 - 0.075	0.001 (0)	-915.01 (21.01)	0.812 (0.019)	0.771 - 0.858

Continued on next page

Table S1 – *Continued from previous page*

Structure	T	N	p	p_e/p_w	$\overline{\hat{p}}$ (SD)	$\overline{\hat{p_e}}/\overline{\hat{p_w}}$ (SD)	$\overline{\lvert\Delta\rvert}_p$ (SD)	Range $\lvert\Delta\rvert_p$	$\overline{SE}\lvert\Delta\rvert_p$ (SD)	$\overline{BIC}$ (SD)	$\overline{\lvert\Delta\rvert}_{p_e/p_w}$ (SD)	Range $\lvert\Delta\rvert_{p_e/p_w}$
SWG	200	25	0.70	0.10	0.733 (0.018)	0.216 (0.018)	0.034 (0.016)	0.001 - 0.065	0.001 (0)	-902.17 (21.61)	0.116 (0.018)	0.073 - 0.166
SWG	200	25	0.70	0.20	0.727 (0.02)	0.196 (0.018)	0.03 (0.015)	0 - 0.072	0.001 (0)	-903.39 (17.63)	0.014 (0.012)	0 - 0.067
SWG	200	25	0.70	0.30	0.738 (0.016)	0.196 (0.018)	0.038 (0.016)	0.002 - 0.075	0.001 (0)	-900.03 (21.23)	0.104 (0.018)	0.061 - 0.144
SWG	200	25	0.70	0.40	0.733 (0.018)	0.206 (0.019)	0.034 (0.017)	0 - 0.084	0.001 (0)	-900.38 (19.2)	0.194 (0.019)	0.157 - 0.235
SWG	200	25	0.70	0.50	0.75 (0.019)	0.209 (0.017)	0.05 (0.018)	0.01 - 0.092	0.001 (0)	-905.94 (18.23)	0.291 (0.017)	0.249 - 0.33
SWG	200	25	0.70	0.60	0.744 (0.017)	0.182 (0.018)	0.044 (0.017)	0 - 0.082	0.001 (0)	-901.03 (17.73)	0.418 (0.018)	0.371 - 0.465
SWG	200	25	0.70	0.70	0.747 (0.017)	0.16 (0.019)	0.047 (0.017)	0.011 - 0.086	0.001 (0)	-906.86 (16.98)	0.54 (0.019)	0.493 - 0.589
SWG	200	25	0.70	0.80	0.744 (0.015)	0.154 (0.015)	0.044 (0.014)	0.002 - 0.079	0.001 (0)	-907.96 (21.74)	0.646 (0.015)	0.606 - 0.677
SWG	200	25	0.70	0.90	0.748 (0.016)	0.158 (0.016)	0.048 (0.016)	0.01 - 0.087	0.001 (0)	-904.14 (17.09)	0.742 (0.016)	0.707 - 0.779
SWG	200	25	0.80	0.10	0.829 (0.013)	0.255 (0.019)	0.029 (0.012)	0.002 - 0.059	0.001 (0)	-894.87 (22.05)	0.155 (0.019)	0.094 - 0.2
SWG	200	25	0.80	0.20	0.822 (0.014)	0.227 (0.017)	0.023 (0.011)	0.001 - 0.049	0.001 (0)	-888.76 (19.03)	0.028 (0.016)	0 - 0.064
SWG	200	25	0.80	0.30	0.835 (0.011)	0.222 (0.016)	0.035 (0.011)	0.005 - 0.056	0.001 (0)	-893.59 (21.21)	0.078 (0.016)	0.043 - 0.134
SWG	200	25	0.80	0.40	0.828 (0.012)	0.243 (0.018)	0.029 (0.012)	0.001 - 0.058	0.001 (0)	-898.76 (22.44)	0.157 (0.018)	0.109 - 0.196
SWG	200	25	0.80	0.50	0.845 (0.01)	0.213 (0.022)	0.045 (0.01)	0.017 - 0.071	0.001 (0)	-911.51 (23.87)	0.287 (0.022)	0.229 - 0.342
SWG	200	25	0.80	0.60	0.839 (0.009)	0.191 (0.018)	0.039 (0.009)	0.01 - 0.065	0.001 (0)	-892.68 (21.75)	0.409 (0.018)	0.36 - 0.453
SWG	200	25	0.80	0.70	0.84 (0.009)	0.147 (0.021)	0.04 (0.009)	0.008 - 0.058	0.001 (0)	-894.23 (21.57)	0.553 (0.021)	0.505 - 0.596
SWG	200	25	0.80	0.80	0.837 (0.009)	0.148 (0.018)	0.037 (0.009)	0.013 - 0.056	0.001 (0)	-892.96 (22.09)	0.652 (0.018)	0.599 - 0.687
SWG	200	25	0.80	0.90	0.843 (0.009)	0.144 (0.018)	0.043 (0.009)	0.009 - 0.067	0.001 (0)	-888.51 (19.74)	0.756 (0.018)	0.709 - 0.804

Continued on next page

Table S1 – *Continued from previous page*

Structure	T	N	p	p_e/p_w	$\overline{\hat{p}}$ (SD)	$\overline{\hat{p_e}}/\overline{\hat{p_w}}$ (SD)	$\overline{\lvert\Delta\rvert}_p$ (SD)	Range $\lvert\Delta\rvert_p$	$\overline{SE}\lvert\Delta\rvert_p$ (SD)	$\overline{BIC}$ (SD)	$\overline{\lvert\Delta\rvert}_{p_e/p_w}$ (SD)	Range $\lvert\Delta\rvert_{p_e/p_w}$
SWG	200	25	0.90	0.10	0.911 (0.007)	0.111 (0.024)	0.012 (0.006)	0 - 0.024	0 (0)	-837.57 (41.62)	0.02 (0.017)	0 - 0.086
SWG	200	25	0.90	0.20	0.909 (0.007)	0.098 (0.029)	0.01 (0.006)	0.001 - 0.024	0 (0)	-821.47 (44.3)	0.102 (0.029)	0.011 - 0.164
SWG	200	25	0.90	0.30	0.913 (0.006)	0.068 (0.014)	0.013 (0.006)	0 - 0.028	0 (0)	-781.15 (33.45)	0.232 (0.014)	0.203 - 0.258
SWG	200	25	0.90	0.40	0.91 (0.006)	0.105 (0.024)	0.01 (0.006)	0.001 - 0.026	0 (0)	-832.35 (50.32)	0.295 (0.024)	0.215 - 0.339
SWG	200	25	0.90	0.50	0.914 (0.005)	0.051 (0.013)	0.014 (0.005)	0.001 - 0.028	0 (0)	-759.51 (36.99)	0.449 (0.013)	0.402 - 0.475
SWG	200	25	0.90	0.60	0.914 (0.004)	0.05 (0.012)	0.014 (0.004)	0.005 - 0.024	0 (0)	-749.49 (28.42)	0.55 (0.012)	0.513 - 0.584
SWG	200	25	0.90	0.70	0.913 (0.005)	0.031 (0.011)	0.013 (0.005)	0.002 - 0.025	0 (0)	-735.85 (23.01)	0.669 (0.011)	0.636 - 0.699
SWG	200	25	0.90	0.80	0.909 (0.005)	0.041 (0.012)	0.009 (0.004)	0 - 0.021	0 (0)	-749.91 (25.45)	0.759 (0.012)	0.736 - 0.796
SWG	200	25	0.90	0.90	0.913 (0.006)	0.031 (0.011)	0.013 (0.006)	0 - 0.028	0 (0)	-735.08 (24.57)	0.869 (0.011)	0.844 - 0.9
SWG	200	49	0.10	0.10	0.106 (0.007)	0.152 (0.06)	0.007 (0.006)	0 - 0.027	0 (0)	-855.66 (21.25)	0.06 (0.051)	0.001 - 0.258
SWG	200	49	0.10	0.20	0.106 (0.008)	0.161 (0.075)	0.008 (0.006)	0 - 0.027	0 (0)	-851.67 (21.65)	0.074 (0.042)	0.002 - 0.168
SWG	200	49	0.10	0.30	0.105 (0.007)	0.155 (0.07)	0.006 (0.006)	0 - 0.024	0 (0)	-854.25 (21.98)	0.149 (0.063)	0.005 - 0.279
SWG	200	49	0.10	0.40	0.106 (0.007)	0.17 (0.064)	0.007 (0.006)	0 - 0.024	0 (0)	-852.08 (21.19)	0.23 (0.064)	0.047 - 0.338
SWG	200	49	0.10	0.50	0.106 (0.008)	0.166 (0.074)	0.007 (0.007)	0 - 0.03	0 (0)	-850.32 (24.14)	0.334 (0.074)	0.154 - 0.453
SWG	200	49	0.10	0.60	0.105 (0.008)	0.151 (0.08)	0.006 (0.007)	0 - 0.026	0 (0)	-856.46 (25.39)	0.449 (0.08)	0.218 - 0.578
SWG	200	49	0.10	0.70	0.107 (0.008)	0.176 (0.082)	0.008 (0.007)	0 - 0.035	0 (0)	-852.37 (22.94)	0.524 (0.082)	0.275 - 0.669
SWG	200	49	0.10	0.80	0.105 (0.01)	0.163 (0.105)	0.009 (0.007)	0 - 0.039	0 (0)	-856.22 (23.73)	0.637 (0.105)	0.289 - 0.781
SWG	200	49	0.10	0.90	0.103 (0.01)	0.14 (0.104)	0.008 (0.007)	0 - 0.028	0 (0)	-860.25 (23.23)	0.76 (0.104)	0.478 - 0.876
SWG	200	49	0.20	0.10	0.216 (0.014)	0.244 (0.069)	0.017 (0.013)	0 - 0.056	0 (0)	-978.16 (21.11)	0.144 (0.069)	0.025 - 0.306
SWG	200	49	0.20	0.20	0.218 (0.018)	0.252 (0.082)	0.021 (0.015)	0 - 0.059	0 (0)	-972.35 (20.03)	0.078 (0.058)	0.002 - 0.253

Continued on next page

Table S1 – *Continued from previous page*

Structure	T	N	p	p_e/p_w	$\overline{\hat{p}}$ (SD)	$\overline{\hat{p}_e}/\overline{\hat{p}_w}$ (SD)	$\overline{\lvert\Delta\rvert}_p$ (SD)	Range $\lvert\Delta\rvert_p$	$\overline{SE}\lvert\Delta\rvert_p$ (SD)	$\overline{BIC}$ (SD)	$\overline{\lvert\Delta\rvert}_{p_e/p_w}$ (SD)	Range $\lvert\Delta\rvert_{p_e/p_w}$
SWG	200	49	0.20	0.30	0.217 (0.018)	0.251 (0.085)	0.019 (0.016)	0 - 0.056	0 (0)	-981.07 (20.97)	0.085 (0.048)	0.001 - 0.201
SWG	200	49	0.20	0.40	0.216 (0.017)	0.264 (0.082)	0.019 (0.015)	0 - 0.063	0 (0)	-975.83 (24.57)	0.139 (0.077)	0 - 0.297
SWG	200	49	0.20	0.50	0.219 (0.019)	0.264 (0.081)	0.022 (0.015)	0 - 0.056	0 (0)	-976.42 (21.59)	0.236 (0.081)	0.037 - 0.38
SWG	200	49	0.20	0.60	0.217 (0.02)	0.261 (0.09)	0.02 (0.015)	0 - 0.052	0 (0)	-975.34 (24.22)	0.339 (0.09)	0.165 - 0.502
SWG	200	49	0.20	0.70	0.218 (0.02)	0.265 (0.09)	0.021 (0.016)	0 - 0.066	0 (0)	-975.99 (21.54)	0.435 (0.09)	0.237 - 0.605
SWG	200	49	0.20	0.80	0.214 (0.022)	0.253 (0.1)	0.021 (0.016)	0 - 0.064	0 (0)	-973.08 (20.73)	0.547 (0.1)	0.336 - 0.729
SWG	200	49	0.20	0.90	0.212 (0.022)	0.235 (0.099)	0.02 (0.016)	0 - 0.061	0 (0)	-977.22 (24.12)	0.665 (0.099)	0.441 - 0.835
SWG	200	49	0.30	0.10	0.315 (0.017)	0.208 (0.034)	0.018 (0.013)	0 - 0.059	0.001 (0)	-1026.82 (21)	0.108 (0.034)	0.001 - 0.199
SWG	200	49	0.30	0.20	0.31 (0.018)	0.19 (0.034)	0.015 (0.014)	0 - 0.056	0.001 (0)	-1031.45 (18.76)	0.029 (0.02)	0 - 0.1
SWG	200	49	0.30	0.30	0.313 (0.018)	0.203 (0.035)	0.018 (0.013)	0 - 0.054	0.001 (0)	-1030.82 (20.28)	0.097 (0.035)	0.015 - 0.202
SWG	200	49	0.30	0.40	0.313 (0.017)	0.218 (0.037)	0.017 (0.012)	0 - 0.05	0.001 (0)	-1028.03 (21.48)	0.182 (0.037)	0.088 - 0.255
SWG	200	49	0.30	0.50	0.313 (0.019)	0.209 (0.036)	0.018 (0.013)	0 - 0.054	0.001 (0)	-1026.32 (21.72)	0.291 (0.036)	0.211 - 0.373
SWG	200	49	0.30	0.60	0.311 (0.02)	0.209 (0.04)	0.018 (0.013)	0 - 0.059	0.001 (0)	-1029.92 (20.21)	0.391 (0.04)	0.255 - 0.471
SWG	200	49	0.30	0.70	0.31 (0.018)	0.205 (0.037)	0.016 (0.012)	0 - 0.051	0.001 (0)	-1026.62 (21.46)	0.495 (0.037)	0.392 - 0.591
SWG	200	49	0.30	0.80	0.307 (0.017)	0.197 (0.035)	0.015 (0.011)	0 - 0.045	0.001 (0)	-1024.61 (19.66)	0.603 (0.035)	0.525 - 0.673
SWG	200	49	0.30	0.90	0.304 (0.016)	0.183 (0.035)	0.013 (0.011)	0 - 0.049	0.001 (0)	-1029.29 (21.4)	0.717 (0.035)	0.614 - 0.791
SWG	200	49	0.40	0.10	0.404 (0.018)	0.108 (0.017)	0.014 (0.011)	0 - 0.057	0.001 (0)	-1050.22 (17.77)	0.015 (0.012)	0 - 0.055
SWG	200	49	0.40	0.20	0.399 (0.018)	0.095 (0.015)	0.014 (0.011)	0.001 - 0.065	0.001 (0)	-1045.12 (19.58)	0.105 (0.015)	0.07 - 0.135
SWG	200	49	0.40	0.30	0.399 (0.016)	0.099 (0.015)	0.013 (0.009)	0 - 0.041	0.001 (0)	-1046.04 (18.95)	0.201 (0.015)	0.148 - 0.243
SWG	200	49	0.40	0.40	0.4 (0.018)	0.113 (0.018)	0.014 (0.011)	0 - 0.044	0.001 (0)	-1045.71 (18.67)	0.287 (0.018)	0.237 - 0.326

Continued on next page

Table S1 – *Continued from previous page*

Structure	T	N	p	p_e/p_w	$\overline{\hat{p}}$ (SD)	$\overline{\hat{p_e}}/\overline{\hat{p_w}}$ (SD)	$\overline{\lvert\Delta\rvert}_p$ (SD)	Range $\lvert\Delta\rvert_p$	$\overline{SE}\lvert\Delta\rvert_p$ (SD)	$\overline{BIC}$ (SD)	$\overline{\lvert\Delta\rvert}_{p_e/p_w}$ (SD)	Range $\lvert\Delta\rvert_{p_e/p_w}$
SWG	200	49	0.40	0.50	0.399 (0.017)	0.103 (0.017)	0.014 (0.011)	0 - 0.045	0.001 (0)	-1047.7 (17.17)	0.397 (0.017)	0.337 - 0.429
SWG	200	49	0.40	0.60	0.402 (0.018)	0.105 (0.015)	0.014 (0.011)	0 - 0.058	0.001 (0)	-1046.64 (20.94)	0.495 (0.015)	0.458 - 0.524
SWG	200	49	0.40	0.70	0.395 (0.016)	0.098 (0.014)	0.013 (0.01)	0 - 0.043	0.001 (0)	-1044.68 (21.98)	0.602 (0.014)	0.55 - 0.633
SWG	200	49	0.40	0.80	0.388 (0.015)	0.088 (0.014)	0.016 (0.011)	0 - 0.049	0.001 (0)	-1047.09 (18.53)	0.712 (0.014)	0.657 - 0.744
SWG	200	49	0.40	0.90	0.386 (0.017)	0.083 (0.014)	0.018 (0.013)	0.001 - 0.057	0.001 (0)	-1046.97 (20.84)	0.817 (0.014)	0.789 - 0.862
SWG	200	49	0.50	0.10	0.502 (0.017)	0.009 (0.007)	0.014 (0.01)	0 - 0.04	0.001 (0)	-1047.28 (20.37)	0.091 (0.007)	0.074 - 0.1
SWG	200	49	0.50	0.20	0.498 (0.02)	0.01 (0.006)	0.016 (0.011)	0 - 0.056	0.001 (0)	-1051.7 (21.05)	0.19 (0.006)	0.173 - 0.2
SWG	200	49	0.50	0.30	0.499 (0.019)	0.008 (0.007)	0.016 (0.011)	0 - 0.054	0.001 (0)	-1051.98 (18.74)	0.292 (0.007)	0.272 - 0.3
SWG	200	49	0.50	0.40	0.505 (0.018)	0.01 (0.007)	0.015 (0.01)	0.001 - 0.044	0.001 (0)	-1050.5 (18.81)	0.39 (0.007)	0.366 - 0.4
SWG	200	49	0.50	0.50	0.502 (0.02)	0.008 (0.006)	0.016 (0.012)	0 - 0.066	0.001 (0)	-1048.43 (19.05)	0.492 (0.006)	0.473 - 0.5
SWG	200	49	0.50	0.60	0.503 (0.021)	0.009 (0.007)	0.017 (0.013)	0 - 0.046	0.001 (0)	-1052.68 (21.69)	0.591 (0.007)	0.567 - 0.6
SWG	200	49	0.50	0.70	0.5 (0.02)	0.009 (0.006)	0.016 (0.011)	0 - 0.054	0.001 (0)	-1050.86 (19.22)	0.691 (0.006)	0.674 - 0.7
SWG	200	49	0.50	0.80	0.5 (0.016)	0.008 (0.007)	0.013 (0.009)	0 - 0.039	0.001 (0)	-1052.65 (19.73)	0.792 (0.007)	0.768 - 0.8
SWG	200	49	0.50	0.90	0.502 (0.016)	0.009 (0.007)	0.012 (0.01)	0 - 0.047	0.001 (0)	-1050.88 (17.5)	0.891 (0.007)	0.865 - 0.9
SWG	200	49	0.60	0.10	0.613 (0.019)	0.105 (0.012)	0.019 (0.013)	0 - 0.057	0.001 (0)	-1047.41 (19.72)	0.01 (0.008)	0 - 0.035
SWG	200	49	0.60	0.20	0.62 (0.019)	0.095 (0.012)	0.023 (0.015)	0 - 0.056	0.001 (0)	-1049.36 (19.72)	0.105 (0.012)	0.072 - 0.132
SWG	200	49	0.60	0.30	0.615 (0.02)	0.098 (0.011)	0.021 (0.014)	0 - 0.061	0.001 (0)	-1047.99 (20.36)	0.202 (0.011)	0.17 - 0.224
SWG	200	49	0.60	0.40	0.615 (0.018)	0.11 (0.01)	0.019 (0.014)	0.001 - 0.059	0.001 (0)	-1049.05 (20.99)	0.29 (0.01)	0.265 - 0.316
SWG	200	49	0.60	0.50	0.622 (0.018)	0.103 (0.011)	0.024 (0.015)	0 - 0.058	0.001 (0)	-1048.16 (18.95)	0.397 (0.011)	0.367 - 0.418

Continued on next page

Table S1 – *Continued from previous page*

Structure	T	N	p	p_e/p_w	$\overline{\hat{p}}$ (SD)	$\overline{\hat{p_e}}/\overline{\hat{p_w}}$ (SD)	$\overline{\lvert\Delta\rvert}_p$ (SD)	Range $\lvert\Delta\rvert_p$	$\overline{SE}\lvert\Delta\rvert_p$ (SD)	$\overline{BIC}$ (SD)	$\overline{\lvert\Delta\rvert}_{p_e/p_w}$ (SD)	Range $\lvert\Delta\rvert_{p_e/p_w}$
SWG	200	49	0.60	0.60	0.626 (0.018)	0.103 (0.012)	0.027 (0.016)	0.001 - 0.066	0.001 (0)	-1045.61 (19.13)	0.497 (0.012)	0.462 - 0.529
SWG	200	49	0.60	0.70	0.623 (0.019)	0.099 (0.013)	0.025 (0.015)	0 - 0.063	0.001 (0)	-1050.18 (16.81)	0.601 (0.013)	0.567 - 0.633
SWG	200	49	0.60	0.80	0.628 (0.019)	0.09 (0.014)	0.029 (0.017)	0.001 - 0.074	0.001 (0)	-1049.71 (21.85)	0.71 (0.014)	0.674 - 0.739
SWG	200	49	0.60	0.90	0.632 (0.019)	0.086 (0.013)	0.032 (0.017)	0.001 - 0.069	0.001 (0)	-1051.03 (20.3)	0.814 (0.013)	0.781 - 0.839
SWG	200	49	0.70	0.10	0.724 (0.018)	0.197 (0.012)	0.026 (0.015)	0 - 0.063	0.001 (0)	-1037.4 (18.5)	0.097 (0.012)	0.064 - 0.129
SWG	200	49	0.70	0.20	0.729 (0.014)	0.176 (0.011)	0.029 (0.013)	0.001 - 0.057	0.001 (0)	-1038.47 (18.69)	0.024 (0.011)	0 - 0.05
SWG	200	49	0.70	0.30	0.733 (0.017)	0.19 (0.011)	0.033 (0.017)	0 - 0.084	0.001 (0)	-1037.49 (20.4)	0.11 (0.011)	0.08 - 0.134
SWG	200	49	0.70	0.40	0.736 (0.019)	0.213 (0.013)	0.037 (0.018)	0.001 - 0.077	0.001 (0)	-1041.72 (19.03)	0.187 (0.013)	0.157 - 0.221
SWG	200	49	0.70	0.50	0.742 (0.015)	0.192 (0.014)	0.042 (0.015)	0.007 - 0.082	0.001 (0)	-1039.61 (19.39)	0.308 (0.014)	0.272 - 0.354
SWG	200	49	0.70	0.60	0.744 (0.014)	0.191 (0.011)	0.044 (0.014)	0.002 - 0.073	0.001 (0)	-1044.66 (17.99)	0.409 (0.011)	0.383 - 0.437
SWG	200	49	0.70	0.70	0.741 (0.017)	0.186 (0.011)	0.041 (0.017)	0 - 0.081	0.001 (0)	-1045.7 (20.21)	0.514 (0.011)	0.489 - 0.544
SWG	200	49	0.70	0.80	0.752 (0.013)	0.168 (0.009)	0.052 (0.013)	0.021 - 0.08	0.001 (0)	-1046.56 (20.23)	0.632 (0.009)	0.61 - 0.657
SWG	200	49	0.70	0.90	0.751 (0.013)	0.157 (0.011)	0.051 (0.013)	0.021 - 0.081	0.001 (0)	-1043.14 (18.89)	0.743 (0.011)	0.72 - 0.771
SWG	200	49	0.80	0.10	0.817 (0.012)	0.245 (0.011)	0.018 (0.01)	0 - 0.047	0.001 (0)	-1033.46 (19.84)	0.145 (0.011)	0.117 - 0.172
SWG	200	49	0.80	0.20	0.827 (0.011)	0.206 (0.013)	0.027 (0.01)	0.007 - 0.051	0.001 (0)	-1031.81 (23.6)	0.011 (0.008)	0 - 0.036
SWG	200	49	0.80	0.30	0.828 (0.01)	0.228 (0.013)	0.028 (0.009)	0.001 - 0.047	0.001 (0)	-1036.92 (21.75)	0.072 (0.013)	0.045 - 0.105
SWG	200	49	0.80	0.40	0.832 (0.01)	0.261 (0.013)	0.032 (0.01)	0.007 - 0.055	0.001 (0)	-1043.63 (28.65)	0.139 (0.013)	0.105 - 0.174
SWG	200	49	0.80	0.50	0.836 (0.009)	0.227 (0.017)	0.036 (0.009)	0.008 - 0.052	0.001 (0)	-1044.33 (21.78)	0.273 (0.017)	0.238 - 0.313

Continued on next page

Table S1 – *Continued from previous page*

Structure	T	N	p	p_e/p_w	$\bar{\hat{p}}$ (SD)	$\overline{\hat{p}_e}/\overline{\hat{p}_w}$ (SD)	$\overline{\lvert\Delta\rvert}_p$ (SD)	Range $\lvert\Delta\rvert_p$	$\overline{SE}\lvert\Delta\rvert_p$ (SD)	$\overline{BIC}$ (SD)	$\overline{\lvert\Delta\rvert}_{p_e/p_w}$ (SD)	Range $\lvert\Delta\rvert_{p_e/p_w}$
SWG	200	49	0.80	0.60	0.841 (0.007)	0.208 (0.014)	0.041 (0.007)	0.022 - 0.057	0.001 (0)	-1057.29 (23.74)	0.392 (0.014)	0.347 - 0.423
SWG	200	49	0.80	0.70	0.84 (0.008)	0.198 (0.014)	0.04 (0.008)	0.013 - 0.057	0.001 (0)	-1058.85 (26.26)	0.502 (0.014)	0.466 - 0.541
SWG	200	49	0.80	0.80	0.844 (0.006)	0.153 (0.015)	0.044 (0.006)	0.024 - 0.056	0 (0)	-1048.73 (25.1)	0.647 (0.015)	0.606 - 0.682
SWG	200	49	0.80	0.90	0.843 (0.006)	0.134 (0.014)	0.043 (0.006)	0.028 - 0.056	0 (0)	-1044.17 (24.52)	0.766 (0.014)	0.722 - 0.795
SWG	200	49	0.90	0.10	0.905 (0.005)	0.087 (0.016)	0.006 (0.004)	0 - 0.016	0 (0)	-980.03 (49.21)	0.018 (0.01)	0 - 0.045
SWG	200	49	0.90	0.20	0.908 (0.004)	0.054 (0.007)	0.008 (0.004)	0 - 0.018	0 (0)	-916.44 (32.87)	0.146 (0.007)	0.121 - 0.163
SWG	200	49	0.90	0.30	0.909 (0.004)	0.064 (0.008)	0.009 (0.004)	0 - 0.018	0 (0)	-931.23 (33.78)	0.236 (0.008)	0.21 - 0.261
SWG	200	49	0.90	0.40	0.91 (0.004)	0.08 (0.014)	0.01 (0.004)	0 - 0.019	0 (0)	-964.77 (48.6)	0.32 (0.014)	0.245 - 0.344
SWG	200	49	0.90	0.50	0.911 (0.004)	0.054 (0.009)	0.011 (0.004)	0.001 - 0.02	0 (0)	-917.32 (28.7)	0.446 (0.009)	0.423 - 0.466
SWG	200	49	0.90	0.60	0.91 (0.003)	0.048 (0.007)	0.01 (0.003)	0 - 0.02	0 (0)	-898.24 (25.81)	0.552 (0.007)	0.534 - 0.567
SWG	200	49	0.90	0.70	0.911 (0.004)	0.045 (0.006)	0.011 (0.004)	0.003 - 0.021	0 (0)	-896.37 (27.23)	0.655 (0.006)	0.632 - 0.673
SWG	200	49	0.90	0.80	0.911 (0.003)	0.029 (0.007)	0.011 (0.003)	0.005 - 0.017	0 (0)	-874.63 (23.15)	0.771 (0.007)	0.756 - 0.788
SWG	200	49	0.90	0.90	0.91 (0.004)	0.025 (0.008)	0.01 (0.004)	0.001 - 0.019	0 (0)	-868.37 (23.44)	0.875 (0.008)	0.857 - 0.894
SWG	200	100	0.10	0.10	0.113 (0.006)	0.192 (0.051)	0.013 (0.006)	0 - 0.028	0 (0)	-998.79 (20.95)	0.092 (0.05)	0.003 - 0.242
SWG	200	100	0.10	0.20	0.114 (0.006)	0.212 (0.05)	0.014 (0.005)	0.001 - 0.026	0 (0)	-993.87 (21.91)	0.039 (0.034)	0.001 - 0.178
SWG	200	100	0.10	0.30	0.112 (0.006)	0.201 (0.063)	0.012 (0.006)	0 - 0.027	0 (0)	-992.7 (21.26)	0.102 (0.058)	0.001 - 0.244
SWG	200	100	0.10	0.40	0.11 (0.006)	0.178 (0.061)	0.01 (0.006)	0 - 0.025	0 (0)	-996.26 (20.59)	0.222 (0.061)	0.085 - 0.332
SWG	200	100	0.10	0.50	0.109 (0.007)	0.167 (0.07)	0.009 (0.007)	0 - 0.031	0 (0)	-999.85 (19.84)	0.333 (0.07)	0.12 - 0.486
SWG	200	100	0.10	0.60	0.107 (0.008)	0.149 (0.081)	0.008 (0.007)	0 - 0.026	0 (0)	-1007.72 (25.97)	0.451 (0.081)	0.242 - 0.546

Continued on next page

Table S1 – *Continued from previous page*

Structure	T	N	p	p_e/p_w	$\overline{\hat{p}}$ (SD)	$\overline{\hat{p}_e}/\overline{\hat{p}_w}$ (SD)	$\overline{\lvert\Delta\rvert}_p$ (SD)	Range $\lvert\Delta\rvert_p$	$\overline{SE}\lvert\Delta\rvert_p$ (SD)	$\overline{BIC}$ (SD)	$\overline{\lvert\Delta\rvert}_{p_e/p_w}$ (SD)	Range $\lvert\Delta\rvert_{p_e/p_w}$
SWG	200	100	0.10	0.70	0.107 (0.008)	0.152 (0.085)	0.008 (0.007)	0 - 0.027	0 (0)	-1003.59 (23.29)	0.548 (0.085)	0.315 - 0.661
SWG	200	100	0.10	0.80	0.104 (0.009)	0.125 (0.089)	0.007 (0.007)	0 - 0.033	0 (0)	-1010.74 (26.99)	0.675 (0.089)	0.372 - 0.781
SWG	200	100	0.10	0.90	0.104 (0.036)	0.124 (0.098)	0.012 (0.034)	0 - 0.31	0 (0)	-1019.18 (34.46)	0.776 (0.098)	0.377 - 0.877
SWG	200	100	0.20	0.10	0.229 (0.012)	0.247 (0.044)	0.029 (0.012)	0.001 - 0.057	0 (0)	-1120.57 (17.63)	0.147 (0.044)	0.041 - 0.283
SWG	200	100	0.20	0.20	0.227 (0.012)	0.267 (0.047)	0.027 (0.012)	0.002 - 0.057	0 (0)	-1114.23 (19.98)	0.069 (0.044)	0 - 0.182
SWG	200	100	0.20	0.30	0.222 (0.017)	0.256 (0.071)	0.023 (0.016)	0 - 0.072	0 (0)	-1116.43 (23.52)	0.071 (0.044)	0 - 0.188
SWG	200	100	0.20	0.40	0.217 (0.017)	0.237 (0.076)	0.019 (0.015)	0 - 0.073	0 (0)	-1117.48 (24.26)	0.165 (0.07)	0 - 0.329
SWG	200	100	0.20	0.50	0.218 (0.02)	0.243 (0.087)	0.021 (0.017)	0 - 0.077	0 (0)	-1116.02 (22.43)	0.258 (0.086)	0.016 - 0.395
SWG	200	100	0.20	0.60	0.213 (0.022)	0.225 (0.097)	0.02 (0.016)	0 - 0.057	0 (0)	-1117.78 (23.68)	0.375 (0.097)	0.18 - 0.514
SWG	200	100	0.20	0.70	0.218 (0.023)	0.246 (0.105)	0.024 (0.017)	0 - 0.058	0 (0)	-1119.1 (22.37)	0.454 (0.105)	0.269 - 0.598
SWG	200	100	0.20	0.80	0.215 (0.024)	0.233 (0.106)	0.023 (0.017)	0.001 - 0.064	0 (0)	-1116.73 (19.79)	0.567 (0.106)	0.357 - 0.72
SWG	200	100	0.20	0.90	0.209 (0.025)	0.206 (0.108)	0.021 (0.016)	0 - 0.067	0 (0)	-1123.75 (25.54)	0.694 (0.108)	0.462 - 0.832
SWG	200	100	0.30	0.10	0.323 (0.014)	0.181 (0.021)	0.023 (0.013)	0 - 0.065	0 (0)	-1166.04 (17.32)	0.081 (0.021)	0.047 - 0.143
SWG	200	100	0.30	0.20	0.324 (0.014)	0.201 (0.026)	0.024 (0.013)	0.001 - 0.061	0 (0)	-1170.73 (17.77)	0.02 (0.016)	0 - 0.059
SWG	200	100	0.30	0.30	0.319 (0.018)	0.209 (0.036)	0.021 (0.016)	0 - 0.07	0 (0)	-1165.86 (20.75)	0.091 (0.036)	0.001 - 0.167
SWG	200	100	0.30	0.40	0.316 (0.019)	0.196 (0.036)	0.02 (0.014)	0.001 - 0.074	0 (0)	-1172.21 (19.12)	0.204 (0.036)	0.102 - 0.28
SWG	200	100	0.30	0.50	0.311 (0.019)	0.192 (0.036)	0.018 (0.014)	0 - 0.07	0 (0)	-1171.23 (21.73)	0.308 (0.036)	0.214 - 0.374

Continued on next page

Table S1 – *Continued from previous page*

Structure	T	N	p	p_e/p_w	$\overline{\hat{p}}$ (SD)	$\overline{\hat{p_e}}/\overline{\hat{p_w}}$ (SD)	$\overline{\lvert\Delta\rvert}_p$ (SD)	Range $\lvert\Delta\rvert_p$	$\overline{SE}\lvert\Delta\rvert_p$ (SD)	$\overline{BIC}$ (SD)	$\overline{\lvert\Delta\rvert}_{p_e/p_w}$ (SD)	Range $\lvert\Delta\rvert_{p_e/p_w}$
SWG	200	100	0.30	0.60	0.307 (0.019)	0.186 (0.037)	0.016 (0.011)	0 - 0.051	0 (0)	-1172.46 (23.95)	0.414 (0.037)	0.315 - 0.479
SWG	200	100	0.30	0.70	0.307 (0.02)	0.187 (0.039)	0.017 (0.012)	0 - 0.059	0 (0)	-1172.8 (22.02)	0.513 (0.039)	0.426 - 0.58
SWG	200	100	0.30	0.80	0.301 (0.02)	0.171 (0.04)	0.017 (0.011)	0 - 0.048	0 (0)	-1172.83 (22.24)	0.629 (0.04)	0.534 - 0.708
SWG	200	100	0.30	0.90	0.301 (0.019)	0.172 (0.037)	0.016 (0.01)	0 - 0.042	0 (0)	-1172.45 (20.49)	0.728 (0.037)	0.643 - 0.796
SWG	200	100	0.40	0.10	0.412 (0.016)	0.092 (0.01)	0.015 (0.013)	0 - 0.057	0.001 (0)	-1190.75 (19.42)	0.011 (0.007)	0 - 0.027
SWG	200	100	0.40	0.20	0.406 (0.024)	0.1 (0.015)	0.017 (0.018)	0 - 0.16	0.001 (0)	-1191.38 (20.42)	0.1 (0.015)	0.018 - 0.121
SWG	200	100	0.40	0.30	0.404 (0.016)	0.103 (0.012)	0.013 (0.009)	0 - 0.035	0.001 (0)	-1185.84 (17.31)	0.197 (0.012)	0.175 - 0.223
SWG	200	100	0.40	0.40	0.397 (0.016)	0.093 (0.011)	0.013 (0.01)	0 - 0.037	0.001 (0)	-1188.23 (20.5)	0.307 (0.011)	0.279 - 0.328
SWG	200	100	0.40	0.50	0.397 (0.014)	0.094 (0.01)	0.012 (0.009)	0 - 0.036	0.001 (0)	-1189.72 (19.74)	0.406 (0.01)	0.37 - 0.427
SWG	200	100	0.40	0.60	0.392 (0.016)	0.091 (0.011)	0.015 (0.011)	0 - 0.054	0.001 (0)	-1191.54 (17.74)	0.509 (0.011)	0.475 - 0.535
SWG	200	100	0.40	0.70	0.394 (0.019)	0.091 (0.014)	0.017 (0.011)	0 - 0.042	0.001 (0)	-1186.48 (19.6)	0.609 (0.014)	0.579 - 0.64
SWG	200	100	0.40	0.80	0.389 (0.015)	0.083 (0.011)	0.014 (0.011)	0 - 0.052	0.001 (0)	-1192.18 (18.93)	0.717 (0.011)	0.681 - 0.744
SWG	200	100	0.40	0.90	0.39 (0.019)	0.085 (0.012)	0.018 (0.012)	0 - 0.055	0.001 (0)	-1187 (19.29)	0.815 (0.012)	0.784 - 0.842
SWG	200	100	0.50	0.10	0.498 (0.02)	0.006 (0.004)	0.016 (0.012)	0 - 0.05	0.001 (0)	-1194.04 (19.01)	0.094 (0.004)	0.084 - 0.1
SWG	200	100	0.50	0.20	0.498 (0.02)	0.006 (0.004)	0.015 (0.013)	0 - 0.071	0.001 (0)	-1195.94 (20.28)	0.194 (0.004)	0.181 - 0.2
SWG	200	100	0.50	0.30	0.501 (0.018)	0.006 (0.005)	0.014 (0.012)	0 - 0.059	0.001 (0)	-1195.45 (22.61)	0.294 (0.005)	0.278 - 0.3
SWG	200	100	0.50	0.40	0.5 (0.017)	0.006 (0.004)	0.014 (0.01)	0 - 0.039	0.001 (0)	-1191.8 (18.11)	0.394 (0.004)	0.38 - 0.4
SWG	200	100	0.50	0.50	0.501 (0.018)	0.006 (0.005)	0.014 (0.011)	0 - 0.041	0.001 (0)	-1194.02 (20.78)	0.494 (0.005)	0.475 - 0.5
SWG	200	100	0.50	0.60	0.498 (0.017)	0.006 (0.005)	0.014 (0.011)	0.001 - 0.053	0.001 (0)	-1192.27 (20.6)	0.594 (0.005)	0.579 - 0.6

Continued on next page

Table S1 – *Continued from previous page*

Structure	T	N	p	p_e/p_w	$\overline{\hat{p}}$ (SD)	$\overline{\hat{p}_e}/\overline{\hat{p}_w}$ (SD)	$\overline{\lvert\Delta\rvert}_p$ (SD)	Range $\lvert\Delta\rvert_p$	$\overline{SE}\lvert\Delta\rvert_p$ (SD)	$\overline{BIC}$ (SD)	$\overline{\lvert\Delta\rvert}_{p_e/p_w}$ (SD)	Range $\lvert\Delta\rvert_{p_e/p_w}$
SWG	200	100	0.50	0.70	0.504 (0.017)	0.006 (0.004)	0.014 (0.011)	0 - 0.052	0.001 (0)	-1194.95 (18.18)	0.694 (0.004)	0.679 - 0.7
SWG	200	100	0.50	0.80	0.504 (0.019)	0.006 (0.004)	0.016 (0.011)	0 - 0.053	0.001 (0)	-1190.81 (21.72)	0.794 (0.004)	0.78 - 0.8
SWG	200	100	0.50	0.90	0.5 (0.018)	0.006 (0.005)	0.014 (0.011)	0 - 0.056	0.001 (0)	-1193.08 (20.57)	0.894 (0.005)	0.882 - 0.9
SWG	200	100	0.60	0.10	0.608 (0.019)	0.087 (0.009)	0.017 (0.012)	0 - 0.047	0.001 (0)	-1186.63 (16.73)	0.014 (0.008)	0.001 - 0.031
SWG	200	100	0.60	0.20	0.611 (0.021)	0.094 (0.009)	0.02 (0.013)	0 - 0.063	0.001 (0)	-1189.4 (22.11)	0.106 (0.009)	0.088 - 0.128
SWG	200	100	0.60	0.30	0.614 (0.02)	0.099 (0.009)	0.02 (0.014)	0 - 0.062	0.001 (0)	-1188.57 (20)	0.201 (0.009)	0.178 - 0.222
SWG	200	100	0.60	0.40	0.62 (0.018)	0.093 (0.01)	0.022 (0.015)	0 - 0.063	0.001 (0)	-1189.63 (18.62)	0.307 (0.01)	0.281 - 0.328
SWG	200	100	0.60	0.50	0.622 (0.019)	0.095 (0.008)	0.025 (0.014)	0 - 0.069	0.001 (0)	-1186.9 (18.78)	0.405 (0.008)	0.388 - 0.423
SWG	200	100	0.60	0.60	0.626 (0.019)	0.09 (0.008)	0.028 (0.017)	0 - 0.064	0.001 (0)	-1188.21 (19.14)	0.51 (0.008)	0.487 - 0.527
SWG	200	100	0.60	0.70	0.627 (0.018)	0.089 (0.009)	0.028 (0.017)	0.002 - 0.068	0.001 (0)	-1190.4 (20.8)	0.611 (0.009)	0.58 - 0.632
SWG	200	100	0.60	0.80	0.628 (0.017)	0.083 (0.009)	0.029 (0.015)	0 - 0.074	0.001 (0)	-1192.34 (17.93)	0.717 (0.009)	0.688 - 0.738
SWG	200	100	0.60	0.90	0.632 (0.019)	0.084 (0.009)	0.033 (0.017)	0.001 - 0.076	0.001 (0)	-1192.54 (17.66)	0.816 (0.009)	0.798 - 0.84
SWG	200	100	0.70	0.10	0.711 (0.018)	0.162 (0.009)	0.017 (0.012)	0 - 0.05	0.001 (0)	-1172.03 (17.9)	0.062 (0.009)	0.045 - 0.095
SWG	200	100	0.70	0.20	0.718 (0.017)	0.177 (0.01)	0.021 (0.013)	0 - 0.053	0.001 (0)	-1174.89 (17.38)	0.023 (0.01)	0.001 - 0.06
SWG	200	100	0.70	0.30	0.734 (0.017)	0.19 (0.01)	0.035 (0.015)	0.003 - 0.067	0.001 (0)	-1177.56 (21.18)	0.11 (0.01)	0.09 - 0.135
SWG	200	100	0.70	0.40	0.735 (0.015)	0.178 (0.008)	0.035 (0.015)	0.003 - 0.072	0.001 (0)	-1178.8 (19.63)	0.222 (0.008)	0.199 - 0.238
SWG	200	100	0.70	0.50	0.742 (0.016)	0.183 (0.009)	0.042 (0.016)	0.002 - 0.076	0.001 (0)	-1184.45 (20.77)	0.317 (0.009)	0.297 - 0.336
SWG	200	100	0.70	0.60	0.744 (0.015)	0.168 (0.007)	0.045 (0.014)	0.006 - 0.072	0.001 (0)	-1184.61 (18.98)	0.432 (0.007)	0.411 - 0.454
SWG	200	100	0.70	0.70	0.75 (0.016)	0.172 (0.008)	0.05 (0.016)	0.001 - 0.086	0.001 (0)	-1182.78 (19.43)	0.528 (0.008)	0.51 - 0.547

Continued on next page

Table S1 – *Continued from previous page*

Structure	T	N	p	p_e/p_w	$\overline{\hat{p}}$ (SD)	$\overline{\hat{p}_e}/\overline{\hat{p}_w}$ (SD)	$\overline{\lvert\Delta\rvert}_p$ (SD)	Range $\lvert\Delta\rvert_p$	$\overline{SE}\lvert\Delta\rvert_p$ (SD)	$\overline{BIC}$ (SD)	$\overline{\lvert\Delta\rvert}_{p_e/p_w}$ (SD)	Range $\lvert\Delta\rvert_{p_e/p_w}$
SWG	200	100	0.70	0.80	0.749 (0.013)	0.157 (0.008)	0.049 (0.013)	0.016 - 0.081	0.001 (0)	-1182.52 (20.81)	0.643 (0.008)	0.616 - 0.661
SWG	200	100	0.70	0.90	0.756 (0.012)	0.156 (0.008)	0.056 (0.012)	0.021 - 0.079	0.001 (0)	-1188.63 (21.13)	0.744 (0.008)	0.719 - 0.765
SWG	200	100	0.80	0.10	0.797 (0.014)	0.207 (0.007)	0.012 (0.009)	0 - 0.047	0.001 (0)	-1154.83 (17.25)	0.107 (0.007)	0.09 - 0.127
SWG	200	100	0.80	0.20	0.815 (0.013)	0.225 (0.007)	0.017 (0.01)	0.001 - 0.042	0.001 (0)	-1166.63 (20.42)	0.025 (0.007)	0.003 - 0.04
SWG	200	100	0.80	0.30	0.832 (0.01)	0.235 (0.008)	0.032 (0.009)	0.005 - 0.05	0.001 (0)	-1179.24 (21.84)	0.065 (0.008)	0.041 - 0.084
SWG	200	100	0.80	0.40	0.836 (0.007)	0.209 (0.01)	0.036 (0.007)	0.017 - 0.05	0 (0)	-1199.08 (23.72)	0.191 (0.01)	0.172 - 0.217
SWG	200	100	0.80	0.50	0.839 (0.007)	0.21 (0.013)	0.039 (0.007)	0.013 - 0.051	0 (0)	-1212.59 (27.78)	0.29 (0.013)	0.262 - 0.325
SWG	200	100	0.80	0.60	0.844 (0.005)	0.165 (0.012)	0.044 (0.005)	0.032 - 0.055	0 (0)	-1208.35 (23.8)	0.435 (0.012)	0.4 - 0.466
SWG	200	100	0.80	0.70	0.846 (0.004)	0.157 (0.014)	0.046 (0.004)	0.034 - 0.056	0 (0)	-1211.04 (29.47)	0.543 (0.014)	0.505 - 0.574
SWG	200	100	0.80	0.80	0.843 (0.004)	0.136 (0.012)	0.043 (0.004)	0.033 - 0.052	0 (0)	-1201.46 (28.41)	0.664 (0.012)	0.636 - 0.687
SWG	200	100	0.80	0.90	0.845 (0.003)	0.121 (0.01)	0.045 (0.003)	0.037 - 0.053	0 (0)	-1194.42 (35.23)	0.779 (0.01)	0.755 - 0.801
SWG	200	100	0.90	0.10	0.882 (0.015)	0.113 (0.03)	0.018 (0.015)	0 - 0.081	0 (0)	-1187.39 (56.47)	0.024 (0.022)	0 - 0.099
SWG	200	100	0.90	0.20	0.9 (0.005)	0.075 (0.014)	0.004 (0.003)	0 - 0.012	0 (0)	-1146.09 (67.19)	0.125 (0.014)	0.064 - 0.145
SWG	200	100	0.90	0.30	0.907 (0.003)	0.057 (0.006)	0.007 (0.003)	0 - 0.013	0 (0)	-1079.16 (39.26)	0.243 (0.006)	0.226 - 0.255
SWG	200	100	0.90	0.40	0.908 (0.002)	0.043 (0.005)	0.008 (0.002)	0.003 - 0.014	0 (0)	-1054.01 (29.09)	0.357 (0.005)	0.344 - 0.37
SWG	200	100	0.90	0.50	0.909 (0.003)	0.044 (0.004)	0.009 (0.003)	0.001 - 0.016	0 (0)	-1045.59 (24.5)	0.456 (0.004)	0.445 - 0.465
SWG	200	100	0.90	0.60	0.909 (0.003)	0.032 (0.004)	0.009 (0.003)	0.002 - 0.016	0 (0)	-1028.4 (22.02)	0.568 (0.004)	0.556 - 0.576
SWG	200	100	0.90	0.70	0.91 (0.003)	0.031 (0.005)	0.01 (0.003)	0.004 - 0.016	0 (0)	-1020.08 (20.96)	0.669 (0.005)	0.659 - 0.682
SWG	200	100	0.90	0.80	0.909 (0.002)	0.024 (0.005)	0.009 (0.002)	0.003 - 0.018	0 (0)	-1014 (20.7)	0.776 (0.005)	0.764 - 0.787

Continued on next page

Table S1 – *Continued from previous page*

Structure	T	N	p	p_e/p_w	$\overline{\hat{p}}$ (SD)	$\overline{\hat{p_e}}/\overline{\hat{p_w}}$ (SD)	$\overline{\lvert\Delta\rvert}_p$ (SD)	Range $\lvert\Delta\rvert_p$	$\overline{SE}\lvert\Delta\rvert_p$ (SD)	$\overline{BIC}$ (SD)	$\overline{\lvert\Delta\rvert}_{p_e/p_w}$ (SD)	Range $\lvert\Delta\rvert_{p_e/p_w}$
SWG	200	100	0.90	0.90	0.909 (0.002)	0.022 (0.004)	0.009 (0.002)	0.004 - 0.014	0 (0)	-1010.59 (22.64)	0.878 (0.004)	0.867 - 0.89
SWG	500	16	0.10	0.10	0.112 (0.007)	0.349 (0.085)	0.012 (0.006)	0 - 0.027	0 (0)	-1548.73 (33.86)	0.25 (0.081)	0.058 - 0.486
SWG	500	16	0.10	0.20	0.109 (0.007)	0.322 (0.078)	0.01 (0.005)	0.001 - 0.024	0 (0)	-1543.1 (29.66)	0.133 (0.059)	0.009 - 0.283
SWG	500	16	0.10	0.30	0.108 (0.009)	0.319 (0.121)	0.01 (0.007)	0 - 0.033	0 (0)	-1541.27 (30.01)	0.102 (0.068)	0 - 0.266
SWG	500	16	0.10	0.40	0.109 (0.007)	0.323 (0.081)	0.01 (0.006)	0 - 0.024	0 (0)	-1544.05 (27.44)	0.089 (0.068)	0.003 - 0.327
SWG	500	16	0.10	0.50	0.109 (0.008)	0.326 (0.095)	0.01 (0.006)	0 - 0.026	0 (0)	-1543.77 (33.56)	0.175 (0.093)	0.022 - 0.439
SWG	500	16	0.10	0.60	0.106 (0.009)	0.302 (0.123)	0.01 (0.006)	0 - 0.026	0 (0)	-1544.09 (32.86)	0.298 (0.123)	0.064 - 0.588
SWG	500	16	0.10	0.70	0.106 (0.009)	0.272 (0.122)	0.008 (0.006)	0 - 0.024	0 (0)	-1552.3 (33.98)	0.428 (0.122)	0.204 - 0.665
SWG	500	16	0.10	0.80	0.106 (0.01)	0.295 (0.124)	0.01 (0.007)	0 - 0.03	0 (0)	-1547.9 (35.86)	0.505 (0.124)	0.275 - 0.77
SWG	500	16	0.10	0.90	0.105 (0.008)	0.255 (0.1)	0.008 (0.005)	0 - 0.022	0 (0)	-1554.47 (38.3)	0.645 (0.1)	0.456 - 0.86
SWG	500	16	0.20	0.10	0.224 (0.01)	0.408 (0.05)	0.024 (0.01)	0.002 - 0.047	0 (0)	-1887.98 (33.87)	0.308 (0.05)	0.191 - 0.434
SWG	500	16	0.20	0.20	0.224 (0.009)	0.396 (0.053)	0.024 (0.009)	0.001 - 0.048	0 (0)	-1889.58 (31.46)	0.196 (0.053)	0.05 - 0.33
SWG	500	16	0.20	0.30	0.218 (0.012)	0.385 (0.075)	0.019 (0.011)	0 - 0.042	0 (0)	-1889.26 (39.28)	0.096 (0.06)	0 - 0.275
SWG	500	16	0.20	0.40	0.221 (0.01)	0.384 (0.052)	0.021 (0.01)	0 - 0.045	0 (0)	-1886.17 (29.54)	0.043 (0.034)	0 - 0.182
SWG	500	16	0.20	0.50	0.223 (0.011)	0.406 (0.055)	0.023 (0.011)	0.001 - 0.045	0 (0)	-1886.35 (38.21)	0.097 (0.051)	0.007 - 0.202
SWG	500	16	0.20	0.60	0.222 (0.011)	0.414 (0.063)	0.022 (0.01)	0.001 - 0.046	0 (0)	-1881.98 (31.34)	0.186 (0.063)	0.044 - 0.357
SWG	500	16	0.20	0.70	0.222 (0.012)	0.407 (0.067)	0.023 (0.011)	0.001 - 0.055	0 (0)	-1890.19 (38.23)	0.293 (0.067)	0.125 - 0.508
SWG	500	16	0.20	0.80	0.22 (0.013)	0.405 (0.076)	0.021 (0.012)	0.001 - 0.051	0 (0)	-1883.1 (28.86)	0.395 (0.076)	0.215 - 0.565

Continued on next page

Table S1 – *Continued from previous page*

Structure	T	N	p	p_e/p_w	$\overline{\hat{p}}$ (SD)	$\overline{\hat{p}_e}/\overline{\hat{p}_w}$ (SD)	$\overline{\lvert\Delta\rvert}_p$ (SD)	Range $\lvert\Delta\rvert_p$	$\overline{SE}\lvert\Delta\rvert_p$ (SD)	$\overline{BIC}$ (SD)	$\overline{\lvert\Delta\rvert}_{p_e/p_w}$ (SD)	Range $\lvert\Delta\rvert_{p_e/p_w}$
SWG	500	16	0.20	0.90	0.209 (0.013)	0.297 (0.071)	0.013 (0.008)	0 - 0.038	0 (0)	-1895.67 (33.39)	0.603 (0.071)	0.455 - 0.746
SWG	500	16	0.30	0.10	0.312 (0.01)	0.267 (0.023)	0.013 (0.009)	0 - 0.037	0 (0)	-2027.52 (27.58)	0.167 (0.023)	0.107 - 0.226
SWG	500	16	0.30	0.20	0.312 (0.01)	0.262 (0.027)	0.014 (0.008)	0 - 0.033	0 (0)	-2029.19 (29.1)	0.062 (0.027)	0.011 - 0.13
SWG	500	16	0.30	0.30	0.301 (0.011)	0.244 (0.031)	0.009 (0.007)	0 - 0.029	0 (0)	-2027.43 (31.54)	0.058 (0.027)	0 - 0.143
SWG	500	16	0.30	0.40	0.307 (0.011)	0.253 (0.03)	0.01 (0.008)	0 - 0.031	0 (0)	-2033.98 (30.03)	0.147 (0.03)	0.077 - 0.223
SWG	500	16	0.30	0.50	0.311 (0.011)	0.267 (0.033)	0.013 (0.009)	0 - 0.04	0 (0)	-2027.24 (27.62)	0.233 (0.033)	0.146 - 0.322
SWG	500	16	0.30	0.60	0.305 (0.011)	0.256 (0.032)	0.01 (0.007)	0 - 0.029	0 (0)	-2033.35 (31.36)	0.344 (0.032)	0.265 - 0.433
SWG	500	16	0.30	0.70	0.304 (0.012)	0.254 (0.034)	0.01 (0.008)	0 - 0.038	0 (0)	-2029.37 (29.86)	0.446 (0.034)	0.359 - 0.519
SWG	500	16	0.30	0.80	0.307 (0.011)	0.264 (0.029)	0.01 (0.007)	0 - 0.031	0 (0)	-2038.61 (31.51)	0.536 (0.029)	0.455 - 0.619
SWG	500	16	0.30	0.90	0.294 (0.012)	0.184 (0.031)	0.011 (0.008)	0 - 0.029	0 (0)	-2036.19 (29.56)	0.716 (0.031)	0.638 - 0.781
SWG	500	16	0.40	0.10	0.399 (0.013)	0.13 (0.018)	0.01 (0.008)	0 - 0.052	0.001 (0)	-2079.39 (30.51)	0.031 (0.017)	0.001 - 0.084
SWG	500	16	0.40	0.20	0.399 (0.01)	0.128 (0.018)	0.008 (0.006)	0 - 0.029	0.001 (0)	-2075.33 (30.27)	0.072 (0.018)	0.025 - 0.138
SWG	500	16	0.40	0.30	0.39 (0.011)	0.113 (0.018)	0.013 (0.008)	0 - 0.035	0.001 (0)	-2087.68 (31.77)	0.187 (0.018)	0.135 - 0.225
SWG	500	16	0.40	0.40	0.397 (0.013)	0.123 (0.02)	0.011 (0.008)	0 - 0.039	0.001 (0)	-2077.98 (29.13)	0.277 (0.02)	0.237 - 0.321
SWG	500	16	0.40	0.50	0.394 (0.012)	0.124 (0.019)	0.011 (0.008)	0 - 0.034	0.001 (0)	-2081.22 (29.93)	0.376 (0.019)	0.324 - 0.417
SWG	500	16	0.40	0.60	0.39 (0.011)	0.118 (0.02)	0.012 (0.008)	0 - 0.036	0.001 (0)	-2084.02 (31.91)	0.482 (0.02)	0.423 - 0.534
SWG	500	16	0.40	0.70	0.393 (0.012)	0.121 (0.021)	0.011 (0.009)	0 - 0.041	0.001 (0)	-2081.94 (30.74)	0.579 (0.021)	0.529 - 0.643
SWG	500	16	0.40	0.80	0.39 (0.011)	0.12 (0.017)	0.012 (0.009)	0 - 0.036	0.001 (0)	-2085.97 (31.59)	0.68 (0.017)	0.629 - 0.712
SWG	500	16	0.40	0.90	0.387 (0.013)	0.086 (0.018)	0.015 (0.011)	0 - 0.047	0.001 (0)	-2082.46 (32.39)	0.814 (0.018)	0.752 - 0.86
SWG	500	16	0.50	0.10	0.5 (0.012)	0.013 (0.009)	0.01 (0.007)	0 - 0.032	0.001 (0)	-2091.35 (30.87)	0.087 (0.009)	0.062 - 0.099

Continued on next page

Table S1 – *Continued from previous page*

Structure	T	N	p	p_e/p_w	$\overline{\hat{p}}$ (SD)	$\overline{\hat{p}_e}/\overline{\hat{p}_w}$ (SD)	$\overline{\lvert\Delta\rvert}_p$ (SD)	Range $\lvert\Delta\rvert_p$	$\overline{SE}\lvert\Delta\rvert_p$ (SD)	$\overline{BIC}$ (SD)	$\overline{\lvert\Delta\rvert}_{p_e/p_w}$ (SD)	Range $\lvert\Delta\rvert_{p_e/p_w}$
SWG	500	16	0.50	0.20	0.499 (0.013)	0.012 (0.01)	0.011 (0.008)	0 - 0.044	0.001 (0)	-2094.06 (34.14)	0.188 (0.01)	0.155 - 0.2
SWG	500	16	0.50	0.30	0.5 (0.015)	0.014 (0.01)	0.011 (0.009)	0 - 0.053	0.001 (0)	-2092.79 (28.6)	0.286 (0.01)	0.255 - 0.3
SWG	500	16	0.50	0.40	0.502 (0.012)	0.014 (0.009)	0.01 (0.008)	0 - 0.029	0.001 (0)	-2089.19 (29.48)	0.386 (0.009)	0.36 - 0.399
SWG	500	16	0.50	0.50	0.502 (0.013)	0.013 (0.009)	0.011 (0.008)	0 - 0.042	0.001 (0)	-2091.68 (30.63)	0.487 (0.009)	0.458 - 0.5
SWG	500	16	0.50	0.60	0.5 (0.013)	0.014 (0.011)	0.011 (0.007)	0 - 0.033	0.001 (0)	-2090.95 (31.85)	0.586 (0.011)	0.542 - 0.6
SWG	500	16	0.50	0.70	0.502 (0.013)	0.014 (0.009)	0.01 (0.009)	0 - 0.039	0.001 (0)	-2093.26 (28.35)	0.686 (0.009)	0.659 - 0.7
SWG	500	16	0.50	0.80	0.501 (0.013)	0.012 (0.009)	0.009 (0.009)	0 - 0.052	0.001 (0)	-2096.43 (33.28)	0.788 (0.009)	0.747 - 0.8
SWG	500	16	0.50	0.90	0.501 (0.012)	0.012 (0.01)	0.01 (0.007)	0 - 0.037	0.001 (0)	-2097.97 (32.64)	0.888 (0.01)	0.843 - 0.9
SWG	500	16	0.60	0.10	0.617 (0.013)	0.123 (0.017)	0.019 (0.011)	0 - 0.049	0.001 (0)	-2085.73 (29.56)	0.024 (0.016)	0.001 - 0.063
SWG	500	16	0.60	0.20	0.615 (0.014)	0.122 (0.014)	0.017 (0.011)	0 - 0.045	0.001 (0)	-2081.14 (32.36)	0.078 (0.014)	0.05 - 0.111
SWG	500	16	0.60	0.30	0.625 (0.014)	0.111 (0.016)	0.026 (0.013)	0.001 - 0.057	0.001 (0)	-2082.23 (30.49)	0.189 (0.016)	0.157 - 0.228
SWG	500	16	0.60	0.40	0.618 (0.013)	0.117 (0.017)	0.019 (0.011)	0.001 - 0.049	0.001 (0)	-2083.62 (27.53)	0.283 (0.017)	0.239 - 0.324
SWG	500	16	0.60	0.50	0.621 (0.016)	0.12 (0.018)	0.022 (0.015)	0 - 0.062	0.001 (0)	-2084.56 (28.25)	0.38 (0.018)	0.345 - 0.438
SWG	500	16	0.60	0.60	0.626 (0.014)	0.115 (0.016)	0.026 (0.013)	0.003 - 0.063	0.001 (0)	-2084.9 (29.18)	0.485 (0.016)	0.444 - 0.522
SWG	500	16	0.60	0.70	0.626 (0.016)	0.117 (0.018)	0.026 (0.015)	0.001 - 0.065	0.001 (0)	-2081.86 (30.24)	0.583 (0.018)	0.538 - 0.628
SWG	500	16	0.60	0.80	0.629 (0.013)	0.12 (0.014)	0.029 (0.013)	0.001 - 0.061	0.001 (0)	-2089.94 (32.05)	0.68 (0.014)	0.651 - 0.714
SWG	500	16	0.60	0.90	0.624 (0.015)	0.086 (0.016)	0.024 (0.014)	0 - 0.055	0.001 (0)	-2081.79 (32.27)	0.814 (0.016)	0.778 - 0.852
SWG	500	16	0.70	0.10	0.728 (0.013)	0.215 (0.014)	0.028 (0.013)	0.003 - 0.075	0 (0)	-2057.38 (33.23)	0.115 (0.014)	0.083 - 0.171
SWG	500	16	0.70	0.20	0.727 (0.013)	0.223 (0.014)	0.027 (0.013)	0.001 - 0.063	0 (0)	-2054.73 (29.62)	0.024 (0.014)	0.001 - 0.061
SWG	500	16	0.70	0.30	0.745 (0.012)	0.196 (0.014)	0.045 (0.012)	0.013 - 0.071	0 (0)	-2060.89 (30.68)	0.104 (0.014)	0.07 - 0.133

Continued on next page

Table S1 – *Continued from previous page*

Structure	T	N	p	p_e/p_w	$\overline{\hat{p}}$ (SD)	$\overline{\hat{p_e}}/\overline{\hat{p_w}}$ (SD)	$\overline{\lvert\Delta\rvert}_p$ (SD)	Range $\lvert\Delta\rvert_p$	$\overline{SE}\lvert\Delta\rvert_p$ (SD)	$\overline{BIC}$ (SD)	$\overline{\lvert\Delta\rvert}_{p_e/p_w}$ (SD)	Range $\lvert\Delta\rvert_{p_e/p_w}$
SWG	500	16	0.70	0.40	0.733 (0.012)	0.208 (0.016)	0.033 (0.012)	0.009 - 0.064	0 (0)	-2058.67 (29.62)	0.192 (0.016)	0.158 - 0.227
SWG	500	16	0.70	0.50	0.735 (0.011)	0.211 (0.014)	0.036 (0.011)	0.002 - 0.058	0 (0)	-2062.11 (31.97)	0.289 (0.014)	0.252 - 0.326
SWG	500	16	0.70	0.60	0.746 (0.012)	0.209 (0.017)	0.046 (0.012)	0.006 - 0.073	0 (0)	-2067.32 (29.57)	0.391 (0.017)	0.35 - 0.434
SWG	500	16	0.70	0.70	0.743 (0.01)	0.204 (0.016)	0.043 (0.01)	0.02 - 0.079	0 (0)	-2063.43 (27.82)	0.496 (0.016)	0.463 - 0.539
SWG	500	16	0.70	0.80	0.747 (0.011)	0.215 (0.014)	0.047 (0.011)	0.02 - 0.073	0 (0)	-2075.23 (32.52)	0.585 (0.014)	0.556 - 0.622
SWG	500	16	0.70	0.90	0.74 (0.012)	0.157 (0.015)	0.04 (0.012)	0.009 - 0.07	0 (0)	-2046.5 (25.79)	0.743 (0.015)	0.701 - 0.782
SWG	500	16	0.80	0.10	0.817 (0.012)	0.228 (0.015)	0.018 (0.01)	0 - 0.041	0 (0)	-2030.94 (26.65)	0.128 (0.015)	0.09 - 0.161
SWG	500	16	0.80	0.20	0.821 (0.011)	0.258 (0.014)	0.022 (0.01)	0.002 - 0.049	0 (0)	-2020.79 (35.14)	0.058 (0.014)	0.032 - 0.087
SWG	500	16	0.80	0.30	0.841 (0.007)	0.202 (0.017)	0.041 (0.007)	0.023 - 0.061	0 (0)	-2024.19 (31.5)	0.098 (0.017)	0.055 - 0.141
SWG	500	16	0.80	0.40	0.832 (0.009)	0.236 (0.015)	0.032 (0.009)	0.012 - 0.051	0 (0)	-2023.35 (33.69)	0.164 (0.015)	0.12 - 0.201
SWG	500	16	0.80	0.50	0.834 (0.009)	0.228 (0.014)	0.034 (0.009)	0.005 - 0.053	0 (0)	-2024.93 (36.42)	0.272 (0.014)	0.244 - 0.313
SWG	500	16	0.80	0.60	0.841 (0.008)	0.212 (0.016)	0.041 (0.008)	0.022 - 0.056	0 (0)	-2044.64 (34.5)	0.388 (0.016)	0.34 - 0.438
SWG	500	16	0.80	0.70	0.842 (0.007)	0.21 (0.016)	0.042 (0.007)	0.025 - 0.055	0 (0)	-2031.48 (29.7)	0.49 (0.016)	0.454 - 0.54
SWG	500	16	0.80	0.80	0.845 (0.007)	0.215 (0.017)	0.045 (0.007)	0.028 - 0.059	0 (0)	-2050.6 (35.31)	0.585 (0.017)	0.538 - 0.64
SWG	500	16	0.80	0.90	0.836 (0.007)	0.164 (0.016)	0.036 (0.007)	0.018 - 0.051	0 (0)	-1981.65 (29.79)	0.736 (0.016)	0.699 - 0.786
SWG	500	16	0.90	0.10	0.899 (0.013)	0.105 (0.018)	0.01 (0.009)	0 - 0.07	0 (0)	-1899.4 (58.15)	0.015 (0.011)	0.001 - 0.054
SWG	500	16	0.90	0.20	0.908 (0.008)	0.139 (0.023)	0.01 (0.005)	0.001 - 0.028	0 (0)	-1879.52 (52.68)	0.061 (0.022)	0.007 - 0.106
SWG	500	16	0.90	0.30	0.916 (0.004)	0.063 (0.013)	0.016 (0.004)	0.007 - 0.025	0 (0)	-1695.13 (47.88)	0.237 (0.013)	0.21 - 0.264

Continued on next page

Table S1 – *Continued from previous page*

Structure	T	N	p	p_e/p_w	$\overline{\hat{p}}$ (SD)	$\overline{\hat{p_e}}/\overline{\hat{p_w}}$ (SD)	$\overline{\lvert\Delta\rvert}_p$ (SD)	Range $\lvert\Delta\rvert_p$	$\overline{SE}\lvert\Delta\rvert_p$ (SD)	$\overline{BIC}$ (SD)	$\overline{\lvert\Delta\rvert}_{p_e/p_w}$ (SD)	Range $\lvert\Delta\rvert_{p_e/p_w}$
SWG	500	16	0.90	0.40	0.914 (0.005)	0.101 (0.016)	0.014 (0.005)	0.001 - 0.026	0 (0)	-1794.63 (58.07)	0.299 (0.016)	0.256 - 0.349
SWG	500	16	0.90	0.50	0.914 (0.004)	0.091 (0.013)	0.014 (0.004)	0.006 - 0.026	0 (0)	-1752.75 (50.3)	0.409 (0.013)	0.372 - 0.437
SWG	500	16	0.90	0.60	0.916 (0.004)	0.068 (0.014)	0.016 (0.004)	0.006 - 0.025	0 (0)	-1722.57 (56.83)	0.532 (0.014)	0.486 - 0.562
SWG	500	16	0.90	0.70	0.916 (0.004)	0.069 (0.014)	0.016 (0.004)	0.009 - 0.029	0 (0)	-1722.82 (63.02)	0.631 (0.014)	0.584 - 0.657
SWG	500	16	0.90	0.80	0.917 (0.004)	0.065 (0.012)	0.017 (0.004)	0.007 - 0.028	0 (0)	-1705.04 (56.39)	0.735 (0.012)	0.71 - 0.767
SWG	500	16	0.90	0.90	0.914 (0.004)	0.056 (0.01)	0.014 (0.004)	0.005 - 0.024	0 (0)	-1656.85 (34.1)	0.844 (0.01)	0.817 - 0.874
SWG	500	25	0.10	0.10	0.111 (0.006)	0.264 (0.055)	0.011 (0.006)	0.001 - 0.025	0 (0)	-1789.68 (30.41)	0.164 (0.055)	0.059 - 0.299
SWG	500	25	0.10	0.20	0.111 (0.005)	0.264 (0.056)	0.012 (0.005)	0 - 0.023	0 (0)	-1789.12 (27.49)	0.073 (0.044)	0.001 - 0.21
SWG	500	25	0.10	0.30	0.111 (0.006)	0.269 (0.065)	0.011 (0.006)	0 - 0.026	0 (0)	-1786.37 (24.91)	0.053 (0.048)	0 - 0.204
SWG	500	25	0.10	0.40	0.108 (0.007)	0.236 (0.083)	0.009 (0.006)	0 - 0.023	0 (0)	-1789.76 (29.85)	0.164 (0.083)	0.009 - 0.358
SWG	500	25	0.10	0.50	0.109 (0.007)	0.254 (0.077)	0.01 (0.006)	0 - 0.028	0 (0)	-1788.89 (30.06)	0.246 (0.077)	0.063 - 0.442
SWG	500	25	0.10	0.60	0.107 (0.008)	0.225 (0.09)	0.009 (0.006)	0 - 0.024	0 (0)	-1790.71 (34.66)	0.375 (0.09)	0.191 - 0.585
SWG	500	25	0.10	0.70	0.106 (0.009)	0.218 (0.106)	0.009 (0.007)	0 - 0.027	0 (0)	-1795.28 (32.4)	0.482 (0.106)	0.255 - 0.667
SWG	500	25	0.10	0.80	0.106 (0.01)	0.213 (0.117)	0.01 (0.006)	0.001 - 0.024	0 (0)	-1799.58 (30.21)	0.587 (0.117)	0.375 - 0.79
SWG	500	25	0.10	0.90	0.108 (0.01)	0.23 (0.114)	0.011 (0.007)	0 - 0.026	0 (0)	-1796.23 (34.05)	0.67 (0.114)	0.468 - 0.896
SWG	500	25	0.20	0.10	0.227 (0.01)	0.341 (0.046)	0.027 (0.01)	0.003 - 0.05	0 (0)	-2109.89 (31.22)	0.241 (0.046)	0.098 - 0.346
SWG	500	25	0.20	0.20	0.226 (0.01)	0.339 (0.045)	0.026 (0.01)	0.006 - 0.047	0 (0)	-2116.62 (34.61)	0.139 (0.045)	0.037 - 0.239
SWG	500	25	0.20	0.30	0.226 (0.009)	0.349 (0.046)	0.026 (0.009)	0.003 - 0.044	0 (0)	-2110.16 (30.38)	0.057 (0.035)	0.001 - 0.152
SWG	500	25	0.20	0.40	0.224 (0.01)	0.335 (0.048)	0.024 (0.01)	0.001 - 0.055	0 (0)	-2112.69 (33.2)	0.068 (0.043)	0.007 - 0.212

Continued on next page

Table S1 – *Continued from previous page*

Structure	T	N	p	p_e/p_w	$\overline{\hat{p}}$ (SD)	$\overline{\hat{p_e}}/\overline{\hat{p_w}}$ (SD)	$\overline{\lvert\Delta\rvert}_p$ (SD)	Range $\lvert\Delta\rvert_p$	$\overline{SE}\lvert\Delta\rvert_p$ (SD)	$\overline{BIC}$ (SD)	$\overline{\lvert\Delta\rvert}_{p_e/p_w}$ (SD)	Range $\lvert\Delta\rvert_{p_e/p_w}$
SWG	500	25	0.20	0.50	0.226 (0.011)	0.363 (0.049)	0.026 (0.011)	0.002 - 0.049	0 (0)	-2111.93 (33.91)	0.137 (0.049)	0.024 - 0.271
SWG	500	25	0.20	0.60	0.222 (0.012)	0.335 (0.061)	0.023 (0.01)	0.003 - 0.045	0 (0)	-2108.17 (31.05)	0.265 (0.061)	0.149 - 0.529
SWG	500	25	0.20	0.70	0.221 (0.014)	0.337 (0.069)	0.021 (0.012)	0 - 0.05	0 (0)	-2115.32 (33.78)	0.363 (0.069)	0.216 - 0.564
SWG	500	25	0.20	0.80	0.221 (0.015)	0.336 (0.075)	0.023 (0.013)	0 - 0.055	0 (0)	-2115.04 (35.08)	0.464 (0.075)	0.314 - 0.656
SWG	500	25	0.20	0.90	0.219 (0.014)	0.326 (0.071)	0.02 (0.013)	0 - 0.059	0 (0)	-2116.01 (33.39)	0.574 (0.071)	0.383 - 0.724
SWG	500	25	0.30	0.10	0.321 (0.009)	0.241 (0.022)	0.021 (0.009)	0.001 - 0.044	0 (0)	-2252.7 (34.04)	0.141 (0.022)	0.089 - 0.193
SWG	500	25	0.30	0.20	0.32 (0.012)	0.246 (0.022)	0.02 (0.011)	0 - 0.052	0 (0)	-2254.36 (33.25)	0.046 (0.022)	0.003 - 0.107
SWG	500	25	0.30	0.30	0.315 (0.01)	0.236 (0.018)	0.016 (0.009)	0 - 0.04	0 (0)	-2251.33 (26.36)	0.064 (0.018)	0.022 - 0.102
SWG	500	25	0.30	0.40	0.312 (0.01)	0.223 (0.025)	0.014 (0.008)	0.001 - 0.038	0 (0)	-2246.21 (28.45)	0.177 (0.025)	0.124 - 0.253
SWG	500	25	0.30	0.50	0.314 (0.01)	0.246 (0.022)	0.014 (0.009)	0 - 0.038	0 (0)	-2251.28 (29.53)	0.254 (0.022)	0.198 - 0.311
SWG	500	25	0.30	0.60	0.309 (0.01)	0.225 (0.025)	0.011 (0.008)	0 - 0.039	0 (0)	-2254.17 (32.8)	0.375 (0.025)	0.281 - 0.425
SWG	500	25	0.30	0.70	0.306 (0.01)	0.22 (0.028)	0.009 (0.007)	0 - 0.032	0 (0)	-2254.49 (28)	0.48 (0.028)	0.414 - 0.557
SWG	500	25	0.30	0.80	0.306 (0.011)	0.22 (0.027)	0.01 (0.008)	0 - 0.042	0 (0)	-2250.55 (29.96)	0.58 (0.027)	0.501 - 0.646
SWG	500	25	0.30	0.90	0.304 (0.009)	0.215 (0.023)	0.008 (0.006)	0 - 0.027	0 (0)	-2255.43 (31.89)	0.685 (0.023)	0.612 - 0.769
SWG	500	25	0.40	0.10	0.402 (0.012)	0.113 (0.013)	0.01 (0.007)	0 - 0.033	0 (0)	-2303.68 (27.27)	0.015 (0.011)	0 - 0.054
SWG	500	25	0.40	0.20	0.402 (0.011)	0.117 (0.014)	0.009 (0.007)	0 - 0.033	0 (0)	-2302.55 (31.18)	0.083 (0.014)	0.046 - 0.118
SWG	500	25	0.40	0.30	0.399 (0.011)	0.115 (0.014)	0.008 (0.006)	0 - 0.029	0 (0)	-2302.63 (30.64)	0.185 (0.014)	0.148 - 0.22
SWG	500	25	0.40	0.40	0.395 (0.011)	0.102 (0.013)	0.009 (0.008)	0 - 0.034	0 (0)	-2298.88 (29.93)	0.298 (0.013)	0.266 - 0.336

Continued on next page

Table S1 – *Continued from previous page*

Structure	T	N	p	p_e/p_w	$\overline{\hat{p}}$ (SD)	$\overline{\hat{p_e}}/\overline{\hat{p_w}}$ (SD)	$\overline{\|\Delta\|}_p$ (SD)	Range $\|\Delta\|_p$	$\overline{SE}\|\Delta\|_p$ (SD)	$\overline{BIC}$ (SD)	$\overline{\|\Delta\|}_{p_e/p_w}$ (SD)	Range $\|\Delta\|_{p_e/p_w}$
SWG	500	25	0.40	0.50	0.396 (0.011)	0.112 (0.015)	0.009 (0.007)	0 - 0.028	0 (0)	-2299.93 (31.26)	0.388 (0.015)	0.354 - 0.42
SWG	500	25	0.40	0.60	0.392 (0.011)	0.103 (0.014)	0.011 (0.008)	0 - 0.031	0 (0)	-2310.45 (31.73)	0.497 (0.014)	0.469 - 0.536
SWG	500	25	0.40	0.70	0.392 (0.012)	0.101 (0.013)	0.012 (0.009)	0 - 0.034	0 (0)	-2303.52 (28.05)	0.599 (0.013)	0.558 - 0.638
SWG	500	25	0.40	0.80	0.389 (0.01)	0.099 (0.014)	0.012 (0.008)	0 - 0.035	0 (0)	-2298.43 (34)	0.701 (0.014)	0.666 - 0.735
SWG	500	25	0.40	0.90	0.388 (0.011)	0.093 (0.013)	0.014 (0.009)	0 - 0.037	0 (0)	-2303.25 (31.64)	0.807 (0.013)	0.777 - 0.836
SWG	500	25	0.50	0.10	0.501 (0.013)	0.009 (0.006)	0.01 (0.008)	0 - 0.038	0.001 (0)	-2316.73 (28.57)	0.091 (0.006)	0.072 - 0.1
SWG	500	25	0.50	0.20	0.5 (0.014)	0.008 (0.007)	0.011 (0.008)	0 - 0.032	0.001 (0)	-2315.02 (33.61)	0.192 (0.007)	0.173 - 0.2
SWG	500	25	0.50	0.30	0.5 (0.014)	0.009 (0.007)	0.012 (0.008)	0 - 0.033	0.001 (0)	-2314.6 (32.84)	0.291 (0.007)	0.27 - 0.3
SWG	500	25	0.50	0.40	0.5 (0.012)	0.009 (0.006)	0.01 (0.007)	0 - 0.024	0.001 (0)	-2319.09 (28.81)	0.391 (0.006)	0.377 - 0.4
SWG	500	25	0.50	0.50	0.502 (0.012)	0.009 (0.006)	0.01 (0.007)	0 - 0.026	0.001 (0)	-2318.4 (31.5)	0.491 (0.006)	0.47 - 0.5
SWG	500	25	0.50	0.60	0.499 (0.013)	0.009 (0.006)	0.01 (0.009)	0 - 0.034	0.001 (0)	-2319.62 (31.61)	0.591 (0.006)	0.572 - 0.6
SWG	500	25	0.50	0.70	0.501 (0.013)	0.009 (0.007)	0.011 (0.008)	0 - 0.035	0.001 (0)	-2317.47 (34.1)	0.691 (0.007)	0.671 - 0.7
SWG	500	25	0.50	0.80	0.499 (0.014)	0.008 (0.007)	0.011 (0.008)	0 - 0.035	0.001 (0)	-2318.45 (31.24)	0.792 (0.007)	0.773 - 0.8
SWG	500	25	0.50	0.90	0.499 (0.013)	0.009 (0.007)	0.011 (0.008)	0 - 0.037	0.001 (0)	-2318.16 (33.37)	0.891 (0.007)	0.866 - 0.9
SWG	500	25	0.60	0.10	0.615 (0.016)	0.111 (0.011)	0.018 (0.012)	0.001 - 0.058	0.001 (0)	-2306.53 (28.29)	0.013 (0.009)	0 - 0.036
SWG	500	25	0.60	0.20	0.616 (0.012)	0.114 (0.012)	0.018 (0.011)	0 - 0.044	0.001 (0)	-2308.47 (34.77)	0.086 (0.012)	0.053 - 0.111
SWG	500	25	0.60	0.30	0.616 (0.013)	0.113 (0.011)	0.018 (0.011)	0 - 0.046	0.001 (0)	-2305.88 (32.73)	0.187 (0.011)	0.157 - 0.209

Continued on next page

Table S1 – *Continued from previous page*

Structure	T	N	p	p_e/p_w	$\overline{\hat{p}}$ (SD)	$\overline{\hat{p}_e}/\overline{\hat{p}_w}$ (SD)	$\overline{\lvert\Delta\rvert}_p$ (SD)	Range $\lvert\Delta\rvert_p$	$\overline{SE}\lvert\Delta\rvert_p$ (SD)	$\overline{BIC}$ (SD)	$\overline{\lvert\Delta\rvert}_{p_e/p_w}$ (SD)	Range $\lvert\Delta\rvert_{p_e/p_w}$
SWG	500	25	0.60	0.40	0.62 (0.012)	0.1 (0.011)	0.02 (0.011)	0 - 0.052	0.001 (0)	-2306.93 (31.34)	0.3 (0.011)	0.271 - 0.334
SWG	500	25	0.60	0.50	0.623 (0.011)	0.11 (0.011)	0.023 (0.011)	0 - 0.053	0.001 (0)	-2299.42 (29.26)	0.39 (0.011)	0.35 - 0.422
SWG	500	25	0.60	0.60	0.625 (0.013)	0.098 (0.011)	0.025 (0.013)	0.001 - 0.052	0.001 (0)	-2308.76 (34.05)	0.502 (0.011)	0.467 - 0.523
SWG	500	25	0.60	0.70	0.627 (0.014)	0.101 (0.011)	0.027 (0.014)	0 - 0.06	0.001 (0)	-2307.17 (30.2)	0.599 (0.011)	0.572 - 0.632
SWG	500	25	0.60	0.80	0.629 (0.011)	0.097 (0.011)	0.029 (0.011)	0.003 - 0.052	0.001 (0)	-2305.75 (30.51)	0.703 (0.011)	0.68 - 0.733
SWG	500	25	0.60	0.90	0.626 (0.011)	0.097 (0.011)	0.026 (0.011)	0 - 0.049	0.001 (0)	-2309.34 (29.97)	0.803 (0.011)	0.78 - 0.837
SWG	500	25	0.70	0.10	0.727 (0.012)	0.205 (0.012)	0.027 (0.012)	0.001 - 0.055	0 (0)	-2277.02 (31.88)	0.105 (0.012)	0.08 - 0.137
SWG	500	25	0.70	0.20	0.727 (0.011)	0.207 (0.011)	0.027 (0.011)	0.002 - 0.05	0 (0)	-2283.97 (29.02)	0.011 (0.008)	0 - 0.034
SWG	500	25	0.70	0.30	0.73 (0.011)	0.203 (0.013)	0.03 (0.011)	0.003 - 0.055	0 (0)	-2285.71 (30.94)	0.097 (0.013)	0.062 - 0.121
SWG	500	25	0.70	0.40	0.736 (0.011)	0.183 (0.011)	0.036 (0.011)	0.002 - 0.066	0 (0)	-2274.58 (30)	0.217 (0.011)	0.192 - 0.244
SWG	500	25	0.70	0.50	0.744 (0.01)	0.206 (0.011)	0.044 (0.01)	0.018 - 0.065	0 (0)	-2281.47 (30.71)	0.294 (0.011)	0.267 - 0.32
SWG	500	25	0.70	0.60	0.743 (0.01)	0.177 (0.01)	0.043 (0.01)	0.018 - 0.076	0 (0)	-2285.8 (35.05)	0.423 (0.01)	0.397 - 0.452
SWG	500	25	0.70	0.70	0.751 (0.012)	0.182 (0.01)	0.051 (0.012)	0.021 - 0.074	0 (0)	-2289.91 (30.75)	0.518 (0.01)	0.494 - 0.54
SWG	500	25	0.70	0.80	0.754 (0.01)	0.174 (0.011)	0.054 (0.01)	0.026 - 0.073	0 (0)	-2282.81 (29.7)	0.626 (0.011)	0.603 - 0.665
SWG	500	25	0.70	0.90	0.749 (0.011)	0.177 (0.011)	0.049 (0.011)	0.018 - 0.074	0 (0)	-2288.36 (28.8)	0.723 (0.011)	0.697 - 0.753
SWG	500	25	0.80	0.10	0.823 (0.009)	0.246 (0.009)	0.023 (0.009)	0 - 0.044	0 (0)	-2243.87 (30.43)	0.146 (0.009)	0.123 - 0.174
SWG	500	25	0.80	0.20	0.823 (0.008)	0.245 (0.01)	0.023 (0.008)	0.006 - 0.045	0 (0)	-2255.89 (30.52)	0.045 (0.01)	0.011 - 0.073
SWG	500	25	0.80	0.30	0.827 (0.009)	0.245 (0.01)	0.027 (0.009)	0.008 - 0.055	0 (0)	-2255.24 (34.42)	0.055 (0.01)	0.032 - 0.074
SWG	500	25	0.80	0.40	0.836 (0.007)	0.202 (0.01)	0.036 (0.007)	0.02 - 0.049	0 (0)	-2251.79 (32.08)	0.198 (0.01)	0.179 - 0.224
SWG	500	25	0.80	0.50	0.843 (0.006)	0.222 (0.013)	0.043 (0.006)	0.027 - 0.059	0 (0)	-2285.73 (35.1)	0.278 (0.013)	0.239 - 0.31
SWG	500	25	0.80	0.60	0.841 (0.006)	0.176 (0.011)	0.041 (0.006)	0.025 - 0.054	0 (0)	-2259.92 (32.71)	0.424 (0.011)	0.396 - 0.453

Continued on next page

Table S1 – *Continued from previous page*

Structure	T	N	p	p_e/p_w	$\overline{\hat{p}}$ (SD)	$\overline{\hat{p}_e}/\overline{\hat{p}_w}$ (SD)	$\overline{\lvert\Delta\rvert}_p$ (SD)	Range $\lvert\Delta\rvert_p$	$\overline{SE}\lvert\Delta\rvert_p$ (SD)	$\overline{BIC}$ (SD)	$\overline{\lvert\Delta\rvert}_{p_e/p_w}$ (SD)	Range $\lvert\Delta\rvert_{p_e/p_w}$
SWG	500	25	0.80	0.70	0.845 (0.006)	0.177 (0.011)	0.045 (0.006)	0.034 - 0.059	0 (0)	-2269.19 (28.52)	0.523 (0.011)	0.488 - 0.553
SWG	500	25	0.80	0.80	0.846 (0.005)	0.154 (0.012)	0.046 (0.005)	0.031 - 0.058	0 (0)	-2257.65 (37.75)	0.646 (0.012)	0.62 - 0.676
SWG	500	25	0.80	0.90	0.843 (0.006)	0.168 (0.013)	0.043 (0.006)	0.033 - 0.059	0 (0)	-2269.02 (31.44)	0.732 (0.013)	0.704 - 0.763
SWG	500	25	0.90	0.10	0.909 (0.004)	0.115 (0.017)	0.009 (0.004)	0 - 0.019	0 (0)	-2131.63 (65.21)	0.017 (0.015)	0 - 0.074
SWG	500	25	0.90	0.20	0.909 (0.005)	0.109 (0.014)	0.01 (0.004)	0.001 - 0.019	0 (0)	-2103.81 (68.3)	0.091 (0.014)	0.051 - 0.12
SWG	500	25	0.90	0.30	0.912 (0.004)	0.099 (0.014)	0.012 (0.004)	0.003 - 0.02	0 (0)	-2094.35 (74.25)	0.201 (0.014)	0.152 - 0.234
SWG	500	25	0.90	0.40	0.913 (0.003)	0.053 (0.008)	0.013 (0.003)	0.003 - 0.022	0 (0)	-1933.67 (48.75)	0.347 (0.008)	0.325 - 0.365
SWG	500	25	0.90	0.50	0.914 (0.004)	0.061 (0.008)	0.014 (0.004)	0.007 - 0.021	0 (0)	-1954.5 (55.88)	0.439 (0.008)	0.414 - 0.461
SWG	500	25	0.90	0.60	0.912 (0.003)	0.042 (0.006)	0.012 (0.003)	0.006 - 0.021	0 (0)	-1902.42 (41.09)	0.558 (0.006)	0.536 - 0.568
SWG	500	25	0.90	0.70	0.914 (0.003)	0.039 (0.008)	0.014 (0.003)	0.004 - 0.024	0 (0)	-1874.03 (50.69)	0.661 (0.008)	0.644 - 0.683
SWG	500	25	0.90	0.80	0.913 (0.003)	0.029 (0.007)	0.013 (0.003)	0.003 - 0.019	0 (0)	-1853.91 (42.92)	0.771 (0.007)	0.75 - 0.788
SWG	500	25	0.90	0.90	0.913 (0.003)	0.039 (0.008)	0.013 (0.003)	0.004 - 0.021	0 (0)	-1887.58 (37.57)	0.861 (0.008)	0.844 - 0.888
SWG	500	49	0.10	0.10	0.111 (0.005)	0.203 (0.048)	0.011 (0.005)	0.001 - 0.024	0 (0)	-2143.08 (32.5)	0.103 (0.047)	0.005 - 0.245
SWG	500	49	0.10	0.20	0.111 (0.005)	0.209 (0.044)	0.011 (0.005)	0.001 - 0.02	0 (0)	-2139.87 (29.23)	0.036 (0.026)	0.001 - 0.103
SWG	500	49	0.10	0.30	0.11 (0.005)	0.204 (0.044)	0.011 (0.004)	0 - 0.02	0 (0)	-2143.85 (31.78)	0.096 (0.044)	0.013 - 0.268
SWG	500	49	0.10	0.40	0.109 (0.006)	0.197 (0.052)	0.01 (0.005)	0 - 0.022	0 (0)	-2140.5 (29.16)	0.203 (0.052)	0.067 - 0.308
SWG	500	49	0.10	0.50	0.109 (0.005)	0.196 (0.052)	0.01 (0.005)	0 - 0.02	0 (0)	-2141.01 (33.01)	0.304 (0.052)	0.194 - 0.438

Continued on next page

Table S1 – *Continued from previous page*

Structure	T	N	p	p_e/p_w	$\overline{\hat{p}}$ (SD)	$\overline{\hat{p}_e}/\overline{\hat{p}_w}$ (SD)	$\overline{\vert\Delta\vert}_p$ (SD)	Range $\vert\Delta\vert_p$	$\overline{SE}\vert\Delta\vert_p$ (SD)	$\overline{BIC}$ (SD)	$\overline{\vert\Delta\vert}_{p_e/p_w}$ (SD)	Range $\vert\Delta\vert_{p_e/p_w}$
SWG	500	49	0.10	0.60	0.107 (0.007)	0.18 (0.066)	0.008 (0.005)	0 - 0.022	0 (0)	-2139.35 (29.81)	0.42 (0.066)	0.268 - 0.545
SWG	500	49	0.10	0.70	0.108 (0.008)	0.194 (0.08)	0.009 (0.007)	0 - 0.024	0 (0)	-2147.08 (28.79)	0.506 (0.08)	0.347 - 0.668
SWG	500	49	0.10	0.80	0.107 (0.008)	0.171 (0.085)	0.008 (0.007)	0 - 0.026	0 (0)	-2149.27 (36.12)	0.629 (0.085)	0.421 - 0.757
SWG	500	49	0.10	0.90	0.104 (0.009)	0.142 (0.09)	0.008 (0.005)	0 - 0.026	0 (0)	-2155.29 (41.15)	0.758 (0.09)	0.555 - 0.882
SWG	500	49	0.20	0.10	0.229 (0.009)	0.286 (0.037)	0.029 (0.009)	0.008 - 0.052	0 (0)	-2456.98 (34.98)	0.186 (0.037)	0.092 - 0.267
SWG	500	49	0.20	0.20	0.229 (0.008)	0.295 (0.033)	0.029 (0.008)	0.007 - 0.056	0 (0)	-2449.1 (28.64)	0.095 (0.033)	0.014 - 0.18
SWG	500	49	0.20	0.30	0.228 (0.009)	0.298 (0.04)	0.028 (0.009)	0.003 - 0.05	0 (0)	-2455.54 (30.45)	0.032 (0.025)	0 - 0.127
SWG	500	49	0.20	0.40	0.224 (0.009)	0.29 (0.041)	0.024 (0.009)	0 - 0.044	0 (0)	-2452.78 (33.45)	0.11 (0.041)	0.027 - 0.225
SWG	500	49	0.20	0.50	0.225 (0.01)	0.294 (0.046)	0.025 (0.01)	0 - 0.048	0 (0)	-2453.8 (35.66)	0.206 (0.046)	0.097 - 0.33
SWG	500	49	0.20	0.60	0.223 (0.015)	0.299 (0.07)	0.023 (0.014)	0 - 0.054	0 (0)	-2451.71 (34.29)	0.301 (0.07)	0.157 - 0.427
SWG	500	49	0.20	0.70	0.221 (0.014)	0.289 (0.061)	0.022 (0.013)	0 - 0.055	0 (0)	-2458.6 (32.34)	0.411 (0.061)	0.271 - 0.534
SWG	500	49	0.20	0.80	0.224 (0.013)	0.296 (0.063)	0.024 (0.013)	0 - 0.052	0 (0)	-2455.94 (32.56)	0.504 (0.063)	0.355 - 0.654
SWG	500	49	0.20	0.90	0.22 (0.014)	0.263 (0.065)	0.021 (0.013)	0.001 - 0.047	0 (0)	-2454.84 (36.64)	0.637 (0.065)	0.52 - 0.769
SWG	500	49	0.30	0.10	0.322 (0.009)	0.206 (0.019)	0.022 (0.009)	0.003 - 0.046	0 (0)	-2590.37 (32.11)	0.106 (0.019)	0.053 - 0.149
SWG	500	49	0.30	0.20	0.326 (0.01)	0.219 (0.019)	0.026 (0.01)	0.003 - 0.054	0 (0)	-2588.13 (30.77)	0.023 (0.016)	0 - 0.072
SWG	500	49	0.30	0.30	0.324 (0.01)	0.222 (0.019)	0.024 (0.01)	0.001 - 0.049	0 (0)	-2587.1 (33.1)	0.078 (0.019)	0.029 - 0.116
SWG	500	49	0.30	0.40	0.319 (0.011)	0.219 (0.023)	0.02 (0.01)	0.002 - 0.053	0 (0)	-2590.68 (30.67)	0.181 (0.023)	0.112 - 0.237
SWG	500	49	0.30	0.50	0.318 (0.011)	0.212 (0.021)	0.019 (0.01)	0.001 - 0.048	0 (0)	-2594.18 (32.94)	0.288 (0.021)	0.228 - 0.334
SWG	500	49	0.30	0.60	0.319 (0.011)	0.233 (0.023)	0.019 (0.01)	0 - 0.06	0 (0)	-2595.49 (35.13)	0.367 (0.023)	0.31 - 0.417
SWG	500	49	0.30	0.70	0.316 (0.011)	0.225 (0.024)	0.016 (0.01)	0 - 0.044	0 (0)	-2589.31 (32.48)	0.475 (0.024)	0.415 - 0.526

Continued on next page

Table S1 – *Continued from previous page*

Structure	T	N	p	p_e/p_w	$\bar{\hat{p}}$ (SD)	$\overline{\hat{p}_e}/\overline{\hat{p}_w}$ (SD)	$\overline{\lvert\Delta\rvert}_p$ (SD)	Range $\lvert\Delta\rvert_p$	$\overline{SE}\lvert\Delta\rvert_p$ (SD)	$\overline{BIC}$ (SD)	$\overline{\lvert\Delta\rvert}_{p_e/p_w}$ (SD)	Range $\lvert\Delta\rvert_{p_e/p_w}$
SWG	500	49	0.30	0.80	0.313 (0.009)	0.212 (0.021)	0.013 (0.008)	0 - 0.031	0 (0)	-2587.89 (33.53)	0.588 (0.021)	0.544 - 0.632
SWG	500	49	0.30	0.90	0.308 (0.01)	0.179 (0.019)	0.01 (0.008)	0 - 0.034	0 (0)	-2593 (32.2)	0.721 (0.019)	0.669 - 0.758
SWG	500	49	0.40	0.10	0.406 (0.012)	0.099 (0.009)	0.011 (0.008)	0 - 0.036	0 (0)	-2639.35 (26.86)	0.007 (0.006)	0 - 0.023
SWG	500	49	0.40	0.20	0.409 (0.011)	0.106 (0.009)	0.012 (0.008)	0 - 0.037	0 (0)	-2638.11 (29.64)	0.094 (0.009)	0.068 - 0.113
SWG	500	49	0.40	0.30	0.402 (0.011)	0.105 (0.009)	0.009 (0.007)	0 - 0.033	0 (0)	-2637.19 (31.33)	0.195 (0.009)	0.171 - 0.215
SWG	500	49	0.40	0.40	0.399 (0.01)	0.101 (0.009)	0.008 (0.006)	0 - 0.029	0 (0)	-2641.88 (33.64)	0.299 (0.009)	0.267 - 0.318
SWG	500	49	0.40	0.50	0.401 (0.01)	0.101 (0.009)	0.008 (0.006)	0 - 0.026	0 (0)	-2643.21 (34.74)	0.399 (0.009)	0.368 - 0.421
SWG	500	49	0.40	0.60	0.397 (0.012)	0.106 (0.011)	0.01 (0.007)	0 - 0.025	0 (0)	-2640.75 (31.63)	0.494 (0.011)	0.47 - 0.523
SWG	500	49	0.40	0.70	0.397 (0.011)	0.101 (0.01)	0.009 (0.007)	0 - 0.025	0 (0)	-2637.81 (31.58)	0.599 (0.01)	0.572 - 0.622
SWG	500	49	0.40	0.80	0.393 (0.01)	0.096 (0.01)	0.011 (0.007)	0 - 0.028	0 (0)	-2644.39 (33.3)	0.704 (0.01)	0.678 - 0.73
SWG	500	49	0.40	0.90	0.389 (0.012)	0.08 (0.009)	0.013 (0.009)	0.001 - 0.038	0 (0)	-2644.2 (32.02)	0.82 (0.009)	0.787 - 0.835
SWG	500	49	0.50	0.10	0.499 (0.011)	0.005 (0.005)	0.009 (0.007)	0 - 0.028	0.001 (0)	-2656.81 (28.6)	0.095 (0.005)	0.082 - 0.1
SWG	500	49	0.50	0.20	0.501 (0.01)	0.005 (0.004)	0.008 (0.006)	0 - 0.027	0.001 (0)	-2642.66 (29.68)	0.195 (0.004)	0.18 - 0.2
SWG	500	49	0.50	0.30	0.502 (0.011)	0.005 (0.004)	0.009 (0.007)	0 - 0.032	0.001 (0)	-2650.46 (32.31)	0.295 (0.004)	0.283 - 0.3
SWG	500	49	0.50	0.40	0.499 (0.013)	0.006 (0.004)	0.01 (0.007)	0 - 0.033	0.001 (0)	-2650.4 (29.5)	0.394 (0.004)	0.385 - 0.4
SWG	500	49	0.50	0.50	0.501 (0.012)	0.006 (0.004)	0.01 (0.007)	0 - 0.031	0.001 (0)	-2647.65 (31.42)	0.494 (0.004)	0.481 - 0.5
SWG	500	49	0.50	0.60	0.501 (0.013)	0.005 (0.004)	0.01 (0.008)	0 - 0.033	0.001 (0)	-2649.87 (32.01)	0.595 (0.004)	0.585 - 0.6
SWG	500	49	0.50	0.70	0.502 (0.011)	0.006 (0.004)	0.009 (0.007)	0 - 0.026	0.001 (0)	-2652.98 (33.62)	0.694 (0.004)	0.681 - 0.7
SWG	500	49	0.50	0.80	0.501 (0.013)	0.006 (0.006)	0.011 (0.008)	0 - 0.033	0.001 (0)	-2651.37 (30.44)	0.794 (0.006)	0.779 - 0.8

Continued on next page

Table S1 – *Continued from previous page*

Structure	T	N	p	p_e/p_w	$\overline{\hat{p}}$ (SD)	$\overline{\hat{p}_e}/\overline{\hat{p}_w}$ (SD)	$\overline{\lvert\Delta\rvert}_p$ (SD)	Range $\lvert\Delta\rvert_p$	$\overline{SE}\lvert\Delta\rvert_p$ (SD)	$\overline{BIC}$ (SD)	$\overline{\lvert\Delta\rvert}_{p_e/p_w}$ (SD)	Range $\lvert\Delta\rvert_{p_e/p_w}$
SWG	500	49	0.50	0.90	0.501 (0.013)	0.006 (0.004)	0.01 (0.008)	0 - 0.037	0.001 (0)	-2649.85 (28.64)	0.894 (0.004)	0.88 - 0.9
SWG	500	49	0.60	0.10	0.611 (0.014)	0.095 (0.008)	0.015 (0.01)	0 - 0.042	0 (0)	-2640.41 (32.69)	0.007 (0.005)	0 - 0.021
SWG	500	49	0.60	0.20	0.61 (0.013)	0.103 (0.008)	0.014 (0.009)	0 - 0.045	0 (0)	-2637.62 (30.76)	0.097 (0.008)	0.076 - 0.113
SWG	500	49	0.60	0.30	0.616 (0.013)	0.101 (0.007)	0.017 (0.011)	0 - 0.05	0 (0)	-2641.75 (31.67)	0.199 (0.007)	0.184 - 0.215
SWG	500	49	0.60	0.40	0.62 (0.012)	0.099 (0.007)	0.02 (0.011)	0.001 - 0.046	0 (0)	-2644.36 (30.24)	0.301 (0.007)	0.283 - 0.315
SWG	500	49	0.60	0.50	0.62 (0.013)	0.097 (0.008)	0.02 (0.012)	0 - 0.051	0 (0)	-2643.49 (29.19)	0.403 (0.008)	0.377 - 0.421
SWG	500	49	0.60	0.60	0.625 (0.013)	0.103 (0.008)	0.026 (0.013)	0.002 - 0.056	0 (0)	-2646.08 (29.05)	0.497 (0.008)	0.477 - 0.519
SWG	500	49	0.60	0.70	0.627 (0.013)	0.098 (0.008)	0.027 (0.013)	0.003 - 0.06	0 (0)	-2636.66 (30.67)	0.602 (0.008)	0.578 - 0.616
SWG	500	49	0.60	0.80	0.628 (0.013)	0.096 (0.007)	0.028 (0.012)	0.001 - 0.06	0 (0)	-2644.7 (34.88)	0.704 (0.007)	0.688 - 0.721
SWG	500	49	0.60	0.90	0.627 (0.014)	0.081 (0.008)	0.028 (0.013)	0.002 - 0.062	0 (0)	-2642.87 (31.78)	0.819 (0.008)	0.799 - 0.835
SWG	500	49	0.70	0.10	0.718 (0.011)	0.179 (0.006)	0.019 (0.01)	0.001 - 0.039	0 (0)	-2606.2 (27.53)	0.079 (0.006)	0.063 - 0.095
SWG	500	49	0.70	0.20	0.722 (0.011)	0.194 (0.007)	0.022 (0.01)	0 - 0.049	0 (0)	-2608.57 (33.22)	0.008 (0.006)	0 - 0.024
SWG	500	49	0.70	0.30	0.729 (0.011)	0.19 (0.007)	0.029 (0.011)	0.002 - 0.059	0 (0)	-2611.78 (29.54)	0.11 (0.007)	0.093 - 0.129
SWG	500	49	0.70	0.40	0.738 (0.01)	0.188 (0.007)	0.038 (0.01)	0.004 - 0.06	0 (0)	-2621.15 (34.22)	0.212 (0.007)	0.197 - 0.227
SWG	500	49	0.70	0.50	0.738 (0.011)	0.186 (0.008)	0.038 (0.011)	0.013 - 0.062	0 (0)	-2618.37 (25.93)	0.314 (0.008)	0.297 - 0.333
SWG	500	49	0.70	0.60	0.752 (0.01)	0.194 (0.008)	0.052 (0.01)	0.02 - 0.076	0 (0)	-2623.34 (31.12)	0.406 (0.008)	0.389 - 0.424
SWG	500	49	0.70	0.70	0.75 (0.01)	0.184 (0.008)	0.05 (0.01)	0.022 - 0.076	0 (0)	-2619.62 (31.1)	0.516 (0.008)	0.494 - 0.54
SWG	500	49	0.70	0.80	0.749 (0.01)	0.181 (0.007)	0.049 (0.01)	0.02 - 0.071	0 (0)	-2620.28 (30.94)	0.619 (0.007)	0.601 - 0.637

Continued on next page

Table S1 – *Continued from previous page*

Structure	T	N	p	p_e/p_w	$\overline{\hat{p}}$ (SD)	$\overline{\hat{p}_e}/\overline{\hat{p}_w}$ (SD)	$\overline{\lvert\Delta\rvert}_p$ (SD)	Range $\lvert\Delta\rvert_p$	$\overline{SE}\lvert\Delta\rvert_p$ (SD)	$\overline{BIC}$ (SD)	$\overline{\lvert\Delta\rvert}_{p_e/p_w}$ (SD)	Range $\lvert\Delta\rvert_{p_e/p_w}$
SWG	500	49	0.70	0.90	0.746 (0.009)	0.15 (0.007)	0.046 (0.009)	0.026 - 0.072	0 (0)	-2623 (33.06)	0.75 (0.007)	0.735 - 0.767
SWG	500	49	0.80	0.10	0.81 (0.008)	0.219 (0.007)	0.011 (0.007)	0 - 0.028	0 (0)	-2567.59 (29.92)	0.119 (0.007)	0.095 - 0.136
SWG	500	49	0.80	0.20	0.815 (0.008)	0.244 (0.006)	0.015 (0.008)	0 - 0.037	0 (0)	-2572 (27.46)	0.044 (0.006)	0.027 - 0.056
SWG	500	49	0.80	0.30	0.823 (0.007)	0.23 (0.008)	0.023 (0.007)	0.007 - 0.04	0 (0)	-2602.58 (31.57)	0.07 (0.008)	0.051 - 0.091
SWG	500	49	0.80	0.40	0.835 (0.006)	0.218 (0.008)	0.035 (0.006)	0.014 - 0.049	0 (0)	-2624.03 (39.21)	0.182 (0.008)	0.161 - 0.204
SWG	500	49	0.80	0.50	0.835 (0.006)	0.213 (0.008)	0.035 (0.006)	0.021 - 0.047	0 (0)	-2623.79 (37.07)	0.287 (0.008)	0.266 - 0.307
SWG	500	49	0.80	0.60	0.845 (0.004)	0.195 (0.01)	0.045 (0.004)	0.032 - 0.058	0 (0)	-2686.62 (36.6)	0.405 (0.01)	0.382 - 0.424
SWG	500	49	0.80	0.70	0.846 (0.004)	0.176 (0.009)	0.046 (0.004)	0.035 - 0.058	0 (0)	-2665.37 (39.49)	0.524 (0.009)	0.497 - 0.545
SWG	500	49	0.80	0.80	0.845 (0.004)	0.18 (0.01)	0.045 (0.004)	0.034 - 0.054	0 (0)	-2655.39 (43.38)	0.62 (0.01)	0.591 - 0.645
SWG	500	49	0.80	0.90	0.842 (0.004)	0.137 (0.008)	0.042 (0.004)	0.029 - 0.049	0 (0)	-2629.17 (39.5)	0.763 (0.008)	0.739 - 0.784
SWG	500	49	0.90	0.10	0.9 (0.004)	0.094 (0.014)	0.003 (0.003)	0 - 0.011	0 (0)	-2532.07 (81.19)	0.012 (0.009)	0 - 0.038
SWG	500	49	0.90	0.20	0.902 (0.003)	0.107 (0.014)	0.003 (0.002)	0 - 0.01	0 (0)	-2573.11 (86.45)	0.093 (0.014)	0.052 - 0.132
SWG	500	49	0.90	0.30	0.907 (0.003)	0.071 (0.008)	0.007 (0.003)	0.001 - 0.015	0 (0)	-2410.67 (76.09)	0.229 (0.008)	0.205 - 0.246
SWG	500	49	0.90	0.40	0.909 (0.002)	0.056 (0.006)	0.009 (0.002)	0.003 - 0.015	0 (0)	-2319.74 (51.01)	0.344 (0.006)	0.329 - 0.358
SWG	500	49	0.90	0.50	0.91 (0.002)	0.052 (0.005)	0.01 (0.002)	0.004 - 0.015	0 (0)	-2302.56 (56.6)	0.448 (0.005)	0.428 - 0.458
SWG	500	49	0.90	0.60	0.911 (0.002)	0.04 (0.004)	0.011 (0.002)	0.006 - 0.016	0 (0)	-2251.68 (42.83)	0.56 (0.004)	0.549 - 0.569
SWG	500	49	0.90	0.70	0.91 (0.002)	0.034 (0.004)	0.01 (0.002)	0.004 - 0.016	0 (0)	-2233.66 (40.93)	0.666 (0.004)	0.654 - 0.678
SWG	500	49	0.90	0.80	0.911 (0.002)	0.038 (0.004)	0.011 (0.002)	0.005 - 0.017	0 (0)	-2240.22 (40.78)	0.762 (0.004)	0.749 - 0.774
SWG	500	49	0.90	0.90	0.91 (0.002)	0.025 (0.005)	0.01 (0.002)	0.004 - 0.016	0 (0)	-2205.83 (36.23)	0.875 (0.005)	0.864 - 0.888
SWG	500	100	0.10	0.10	0.111 (0.005)	0.175 (0.045)	0.011 (0.005)	0 - 0.023	0 (0)	-2508.67 (32.76)	0.076 (0.043)	0.003 - 0.194

Continued on next page

Table S1 – *Continued from previous page*

Structure	T	N	p	p_e/p_w	$\overline{\hat{p}}$ (SD)	$\overline{\hat{p_e}}/\overline{\hat{p_w}}$ (SD)	$\overline{\lvert\Delta\rvert}_p$ (SD)	Range $\lvert\Delta\rvert_p$	$\overline{SE}\lvert\Delta\rvert_p$ (SD)	$\overline{BIC}$ (SD)	$\overline{\lvert\Delta\rvert}_{p_e/p_w}$ (SD)	Range $\lvert\Delta\rvert_{p_e/p_w}$
SWG	500	100	0.10	0.20	0.113 (0.004)	0.204 (0.04)	0.013 (0.004)	0.002 - 0.025	0 (0)	-2502.31 (37.31)	0.031 (0.026)	0.001 - 0.13
SWG	500	100	0.10	0.30	0.111 (0.004)	0.189 (0.044)	0.011 (0.004)	0.001 - 0.021	0 (0)	-2506.16 (34.89)	0.111 (0.044)	0.002 - 0.246
SWG	500	100	0.10	0.40	0.109 (0.004)	0.17 (0.039)	0.009 (0.004)	0 - 0.02	0 (0)	-2508.11 (29.52)	0.23 (0.039)	0.142 - 0.313
SWG	500	100	0.10	0.50	0.108 (0.006)	0.159 (0.057)	0.008 (0.005)	0 - 0.018	0 (0)	-2504.43 (32.45)	0.341 (0.057)	0.224 - 0.448
SWG	500	100	0.10	0.60	0.106 (0.006)	0.142 (0.063)	0.007 (0.006)	0 - 0.021	0 (0)	-2507.94 (34.66)	0.458 (0.063)	0.301 - 0.55
SWG	500	100	0.10	0.70	0.107 (0.007)	0.147 (0.068)	0.007 (0.006)	0 - 0.023	0 (0)	-2510.16 (36.57)	0.553 (0.068)	0.405 - 0.659
SWG	500	100	0.10	0.80	0.106 (0.007)	0.139 (0.066)	0.007 (0.005)	0 - 0.021	0 (0)	-2514.76 (35.88)	0.661 (0.066)	0.512 - 0.76
SWG	500	100	0.10	0.90	0.104 (0.007)	0.124 (0.074)	0.007 (0.005)	0 - 0.019	0 (0)	-2522.94 (41.01)	0.776 (0.074)	0.634 - 0.87
SWG	500	100	0.20	0.10	0.226 (0.007)	0.24 (0.029)	0.026 (0.007)	0.009 - 0.043	0 (0)	-2818.36 (30.47)	0.14 (0.029)	0.068 - 0.209
SWG	500	100	0.20	0.20	0.231 (0.007)	0.286 (0.028)	0.031 (0.007)	0.015 - 0.048	0 (0)	-2805.65 (31.43)	0.086 (0.028)	0.02 - 0.157
SWG	500	100	0.20	0.30	0.228 (0.01)	0.275 (0.044)	0.028 (0.01)	0.004 - 0.049	0 (0)	-2812.67 (35.41)	0.039 (0.031)	0.001 - 0.129
SWG	500	100	0.20	0.40	0.224 (0.013)	0.26 (0.055)	0.024 (0.012)	0.002 - 0.049	0 (0)	-2817.36 (38.05)	0.14 (0.055)	0.02 - 0.276
SWG	500	100	0.20	0.50	0.219 (0.014)	0.248 (0.062)	0.02 (0.013)	0 - 0.046	0 (0)	-2811.83 (33.75)	0.252 (0.062)	0.138 - 0.37
SWG	500	100	0.20	0.60	0.22 (0.016)	0.253 (0.071)	0.021 (0.015)	0 - 0.05	0 (0)	-2816 (35.37)	0.347 (0.071)	0.208 - 0.472
SWG	500	100	0.20	0.70	0.215 (0.017)	0.231 (0.073)	0.017 (0.014)	0 - 0.053	0 (0)	-2820.69 (38.68)	0.469 (0.073)	0.311 - 0.585
SWG	500	100	0.20	0.80	0.217 (0.018)	0.245 (0.079)	0.019 (0.016)	0 - 0.05	0 (0)	-2809.39 (36.89)	0.555 (0.079)	0.398 - 0.705
SWG	500	100	0.20	0.90	0.216 (0.019)	0.24 (0.083)	0.019 (0.016)	0 - 0.055	0 (0)	-2818.26 (40.15)	0.66 (0.083)	0.502 - 0.784
SWG	500	100	0.30	0.10	0.324 (0.01)	0.185 (0.016)	0.024 (0.01)	0.001 - 0.055	0 (0)	-2956.08 (30.77)	0.085 (0.016)	0.051 - 0.135
SWG	500	100	0.30	0.20	0.327 (0.009)	0.215 (0.017)	0.027 (0.009)	0.01 - 0.052	0 (0)	-2945.81 (30.37)	0.018 (0.013)	0 - 0.052
SWG	500	100	0.30	0.30	0.32 (0.011)	0.202 (0.02)	0.021 (0.01)	0.002 - 0.047	0 (0)	-2943.19 (25.8)	0.098 (0.02)	0.054 - 0.161

Continued on next page

Table S1 – *Continued from previous page*

Structure	T	N	p	p_e/p_w	$\overline{\hat{p}}$ (SD)	$\overline{\hat{p_e}}/\overline{\hat{p_w}}$ (SD)	$\overline{\lvert\Delta\rvert}_p$ (SD)	Range $\lvert\Delta\rvert_p$	$\overline{SE}\lvert\Delta\rvert_p$ (SD)	$\overline{BIC}$ (SD)	$\overline{\lvert\Delta\rvert}_{p_e/p_w}$ (SD)	Range $\lvert\Delta\rvert_{p_e/p_w}$
SWG	500	100	0.30	0.40	0.318 (0.011)	0.198 (0.021)	0.018 (0.011)	0 - 0.049	0 (0)	-2954.06 (33.92)	0.202 (0.021)	0.148 - 0.254
SWG	500	100	0.30	0.50	0.317 (0.013)	0.202 (0.026)	0.018 (0.011)	0 - 0.044	0 (0)	-2951.85 (31.11)	0.298 (0.026)	0.244 - 0.363
SWG	500	100	0.30	0.60	0.314 (0.012)	0.199 (0.024)	0.016 (0.011)	0 - 0.04	0 (0)	-2949.14 (30.89)	0.401 (0.024)	0.348 - 0.46
SWG	500	100	0.30	0.70	0.314 (0.014)	0.198 (0.026)	0.016 (0.011)	0 - 0.045	0 (0)	-2948.78 (33.81)	0.502 (0.026)	0.452 - 0.574
SWG	500	100	0.30	0.80	0.312 (0.013)	0.199 (0.026)	0.014 (0.01)	0 - 0.042	0 (0)	-2948.76 (31.86)	0.601 (0.026)	0.534 - 0.658
SWG	500	100	0.30	0.90	0.309 (0.014)	0.193 (0.027)	0.013 (0.011)	0 - 0.046	0 (0)	-2949.09 (33.73)	0.707 (0.027)	0.641 - 0.772
SWG	500	100	0.40	0.10	0.407 (0.01)	0.09 (0.007)	0.01 (0.007)	0 - 0.033	0 (0)	-2998.36 (27.94)	0.011 (0.006)	0 - 0.025
SWG	500	100	0.40	0.20	0.407 (0.011)	0.104 (0.008)	0.01 (0.008)	0 - 0.049	0 (0)	-2999.55 (27.74)	0.096 (0.008)	0.071 - 0.112
SWG	500	100	0.40	0.30	0.404 (0.012)	0.096 (0.008)	0.01 (0.007)	0 - 0.031	0 (0)	-2992.81 (29.09)	0.204 (0.008)	0.184 - 0.22
SWG	500	100	0.40	0.40	0.401 (0.011)	0.096 (0.008)	0.009 (0.007)	0 - 0.041	0 (0)	-2995.53 (27.05)	0.304 (0.008)	0.286 - 0.327
SWG	500	100	0.40	0.50	0.398 (0.011)	0.095 (0.007)	0.009 (0.007)	0 - 0.031	0 (0)	-2991.19 (31.68)	0.405 (0.007)	0.386 - 0.421
SWG	500	100	0.40	0.60	0.395 (0.011)	0.091 (0.007)	0.01 (0.007)	0.001 - 0.03	0 (0)	-2996.42 (33.3)	0.509 (0.007)	0.492 - 0.528
SWG	500	100	0.40	0.70	0.392 (0.01)	0.087 (0.007)	0.011 (0.007)	0 - 0.029	0 (0)	-2996.02 (32.79)	0.613 (0.007)	0.588 - 0.627
SWG	500	100	0.40	0.80	0.392 (0.01)	0.09 (0.008)	0.011 (0.007)	0 - 0.032	0 (0)	-2998.87 (31.42)	0.71 (0.008)	0.686 - 0.731
SWG	500	100	0.40	0.90	0.39 (0.01)	0.088 (0.007)	0.012 (0.008)	0 - 0.043	0 (0)	-3001.87 (32.92)	0.812 (0.007)	0.796 - 0.834
SWG	500	100	0.50	0.10	0.498 (0.014)	0.003 (0.003)	0.011 (0.009)	0 - 0.033	0.001 (0)	-3006.1 (34.54)	0.097 (0.003)	0.088 - 0.1
SWG	500	100	0.50	0.20	0.501 (0.011)	0.004 (0.003)	0.009 (0.007)	0 - 0.029	0.001 (0)	-3011.8 (33.84)	0.196 (0.003)	0.186 - 0.2
SWG	500	100	0.50	0.30	0.502 (0.012)	0.004 (0.003)	0.01 (0.008)	0 - 0.038	0.001 (0)	-3007.13 (32.14)	0.296 (0.003)	0.288 - 0.3

Continued on next page

Table S1 – *Continued from previous page*

Structure	T	N	p	p_e/p_w	$\bar{\hat{p}}$ (SD)	$\overline{\hat{p_e}}/\overline{\hat{p_w}}$ (SD)	$\overline{\|\Delta\|}_p$ (SD)	Range $\|\Delta\|_p$	$\overline{SE}\|\Delta\|_p$ (SD)	$\overline{BIC}$ (SD)	$\overline{\|\Delta\|}_{p_e/p_w}$ (SD)	Range $\|\Delta\|_{p_e/p_w}$
SWG	500	100	0.50	0.40	0.501 (0.011)	0.004 (0.003)	0.008 (0.006)	0 - 0.027	0.001 (0)	-3009.27 (32.99)	0.396 (0.003)	0.387 - 0.4
SWG	500	100	0.50	0.50	0.499 (0.011)	0.004 (0.003)	0.009 (0.006)	0 - 0.028	0.001 (0)	-3006 (32.5)	0.496 (0.003)	0.485 - 0.5
SWG	500	100	0.50	0.60	0.5 (0.012)	0.004 (0.003)	0.009 (0.007)	0 - 0.031	0.001 (0)	-3007.05 (34.65)	0.596 (0.003)	0.585 - 0.6
SWG	500	100	0.50	0.70	0.5 (0.012)	0.003 (0.003)	0.009 (0.008)	0 - 0.034	0.001 (0)	-3006.35 (30.26)	0.697 (0.003)	0.689 - 0.7
SWG	500	100	0.50	0.80	0.501 (0.011)	0.004 (0.003)	0.008 (0.007)	0 - 0.036	0.001 (0)	-3004.15 (30.48)	0.796 (0.003)	0.782 - 0.8
SWG	500	100	0.50	0.90	0.502 (0.01)	0.004 (0.003)	0.008 (0.006)	0 - 0.027	0.001 (0)	-3007.68 (29.94)	0.896 (0.003)	0.883 - 0.9
SWG	500	100	0.60	0.10	0.606 (0.013)	0.087 (0.006)	0.011 (0.009)	0 - 0.039	0 (0)	-2996.39 (32.61)	0.013 (0.006)	0.001 - 0.027
SWG	500	100	0.60	0.20	0.611 (0.013)	0.1 (0.005)	0.015 (0.009)	0.001 - 0.038	0 (0)	-3001.72 (29.09)	0.1 (0.005)	0.088 - 0.113
SWG	500	100	0.60	0.30	0.616 (0.012)	0.095 (0.005)	0.017 (0.01)	0.001 - 0.044	0 (0)	-2992.26 (28.45)	0.205 (0.005)	0.195 - 0.218
SWG	500	100	0.60	0.40	0.619 (0.011)	0.094 (0.006)	0.019 (0.011)	0.001 - 0.045	0 (0)	-3003.11 (33.86)	0.306 (0.006)	0.284 - 0.323
SWG	500	100	0.60	0.50	0.622 (0.012)	0.095 (0.005)	0.022 (0.011)	0.001 - 0.054	0 (0)	-2998.24 (31.87)	0.405 (0.005)	0.393 - 0.417
SWG	500	100	0.60	0.60	0.625 (0.013)	0.091 (0.006)	0.025 (0.013)	0.001 - 0.061	0 (0)	-3001.06 (29.51)	0.509 (0.006)	0.492 - 0.521
SWG	500	100	0.60	0.70	0.626 (0.012)	0.087 (0.005)	0.026 (0.012)	0 - 0.056	0 (0)	-3000.04 (31.65)	0.613 (0.005)	0.598 - 0.628
SWG	500	100	0.60	0.80	0.627 (0.012)	0.09 (0.005)	0.027 (0.012)	0.001 - 0.053	0 (0)	-3001.86 (33.04)	0.71 (0.005)	0.698 - 0.723
SWG	500	100	0.60	0.90	0.63 (0.012)	0.086 (0.005)	0.03 (0.012)	0.001 - 0.059	0 (0)	-3003.11 (30.39)	0.814 (0.005)	0.803 - 0.831
SWG	500	100	0.70	0.10	0.715 (0.01)	0.163 (0.005)	0.015 (0.009)	0 - 0.04	0 (0)	-2965.36 (31.43)	0.063 (0.005)	0.052 - 0.075
SWG	500	100	0.70	0.20	0.723 (0.01)	0.19 (0.006)	0.023 (0.01)	0.002 - 0.048	0 (0)	-2964.4 (27.61)	0.01 (0.006)	0.001 - 0.023
SWG	500	100	0.70	0.30	0.734 (0.011)	0.18 (0.006)	0.034 (0.011)	0.008 - 0.061	0 (0)	-2969.71 (29.35)	0.12 (0.006)	0.105 - 0.138
SWG	500	100	0.70	0.40	0.735 (0.011)	0.181 (0.006)	0.035 (0.011)	0.003 - 0.057	0 (0)	-2968.22 (27.39)	0.219 (0.006)	0.202 - 0.234

Continued on next page

Table S1 – *Continued from previous page*

Structure	T	N	p	p_e/p_w	$\overline{\hat{p}}$ (SD)	$\overline{\hat{p_e}}/\overline{\hat{p_w}}$ (SD)	$\overline{\lvert\Delta\rvert}_p$ (SD)	Range $\lvert\Delta\rvert_p$	$\overline{SE}\lvert\Delta\rvert_p$ (SD)	$\overline{BIC}$ (SD)	$\overline{\lvert\Delta\rvert}_{p_e/p_w}$ (SD)	Range $\lvert\Delta\rvert_{p_e/p_w}$
SWG	500	100	0.70	0.50	0.741 (0.011)	0.182 (0.005)	0.041 (0.011)	0.019 - 0.069	0 (0)	-2975.32 (33.41)	0.318 (0.005)	0.306 - 0.335
SWG	500	100	0.70	0.60	0.747 (0.01)	0.173 (0.005)	0.047 (0.01)	0.023 - 0.07	0 (0)	-2980.98 (30.89)	0.427 (0.005)	0.413 - 0.439
SWG	500	100	0.70	0.70	0.749 (0.01)	0.164 (0.005)	0.049 (0.01)	0.02 - 0.068	0 (0)	-2980.27 (34.66)	0.536 (0.005)	0.524 - 0.545
SWG	500	100	0.70	0.80	0.754 (0.009)	0.175 (0.005)	0.054 (0.009)	0.027 - 0.07	0 (0)	-2986.34 (32.3)	0.625 (0.005)	0.615 - 0.64
SWG	500	100	0.70	0.90	0.76 (0.008)	0.165 (0.005)	0.06 (0.008)	0.04 - 0.078	0 (0)	-2992.25 (28.7)	0.735 (0.005)	0.722 - 0.746
SWG	500	100	0.80	0.10	0.8 (0.008)	0.206 (0.005)	0.006 (0.005)	0 - 0.023	0 (0)	-2909.82 (29.91)	0.106 (0.005)	0.094 - 0.116
SWG	500	100	0.80	0.20	0.816 (0.007)	0.244 (0.005)	0.016 (0.007)	0.001 - 0.031	0 (0)	-2937.96 (32.51)	0.044 (0.005)	0.031 - 0.056
SWG	500	100	0.80	0.30	0.832 (0.005)	0.22 (0.006)	0.032 (0.005)	0.019 - 0.043	0 (0)	-2981.12 (36.44)	0.08 (0.006)	0.068 - 0.097
SWG	500	100	0.80	0.40	0.833 (0.005)	0.222 (0.006)	0.033 (0.005)	0.015 - 0.045	0 (0)	-2993.98 (37.49)	0.178 (0.006)	0.163 - 0.197
SWG	500	100	0.80	0.50	0.842 (0.004)	0.204 (0.008)	0.042 (0.004)	0.031 - 0.05	0 (0)	-3043.52 (47.1)	0.296 (0.008)	0.277 - 0.316
SWG	500	100	0.80	0.60	0.844 (0.003)	0.178 (0.009)	0.044 (0.003)	0.034 - 0.051	0 (0)	-3055.38 (43.82)	0.422 (0.009)	0.401 - 0.443
SWG	500	100	0.80	0.70	0.844 (0.003)	0.152 (0.009)	0.044 (0.003)	0.037 - 0.05	0 (0)	-3031.28 (42.68)	0.548 (0.009)	0.525 - 0.566
SWG	500	100	0.80	0.80	0.848 (0.002)	0.155 (0.009)	0.048 (0.002)	0.041 - 0.054	0 (0)	-3056.57 (49.18)	0.645 (0.009)	0.624 - 0.666
SWG	500	100	0.80	0.90	0.847 (0.002)	0.128 (0.007)	0.047 (0.002)	0.043 - 0.055	0 (0)	-3034 (52.58)	0.772 (0.007)	0.756 - 0.792
SWG	500	100	0.90	0.10	0.89 (0.01)	0.091 (0.017)	0.01 (0.01)	0 - 0.084	0 (0)	-2958.9 (103.88)	0.016 (0.01)	0 - 0.072
SWG	500	100	0.90	0.20	0.901 (0.003)	0.084 (0.008)	0.002 (0.002)	0 - 0.008	0 (0)	-2892.57 (107.74)	0.116 (0.008)	0.092 - 0.132
SWG	500	100	0.90	0.30	0.907 (0.002)	0.05 (0.004)	0.007 (0.002)	0.002 - 0.013	0 (0)	-2695.09 (66.63)	0.25 (0.004)	0.241 - 0.257
SWG	500	100	0.90	0.40	0.908 (0.002)	0.049 (0.003)	0.008 (0.002)	0.003 - 0.013	0 (0)	-2678.66 (47.03)	0.351 (0.003)	0.343 - 0.357

Continued on next page

Table S1 – *Continued from previous page*

Structure	T	N	p	p_e/p_w	$\overline{\hat{p}}$ (SD)	$\overline{\hat{p}_e}/\overline{\hat{p}_w}$ (SD)	$\overline{\lvert\Delta\rvert}_p$ (SD)	Range $\lvert\Delta\rvert_p$	$\overline{SE}\lvert\Delta\rvert_p$ (SD)	$\overline{BIC}$ (SD)	$\overline{\lvert\Delta\rvert}_{p_e/p_w}$ (SD)	Range $\lvert\Delta\rvert_{p_e/p_w}$
SWG	500	100	0.90	0.50	0.909 (0.001)	0.041 (0.003)	0.009 (0.001)	0.004 - 0.012	0 (0)	-2631.09 (48.18)	0.459 (0.003)	0.454 - 0.466
SWG	500	100	0.90	0.60	0.909 (0.002)	0.035 (0.003)	0.009 (0.002)	0.005 - 0.012	0 (0)	-2599.41 (39.04)	0.565 (0.003)	0.558 - 0.573
SWG	500	100	0.90	0.70	0.909 (0.002)	0.03 (0.003)	0.009 (0.002)	0.004 - 0.014	0 (0)	-2585.54 (38.91)	0.67 (0.003)	0.663 - 0.678
SWG	500	100	0.90	0.80	0.909 (0.002)	0.028 (0.003)	0.009 (0.002)	0.005 - 0.014	0 (0)	-2574.95 (38.57)	0.772 (0.003)	0.763 - 0.778
SWG	500	100	0.90	0.90	0.909 (0.002)	0.025 (0.003)	0.009 (0.002)	0.004 - 0.013	0 (0)	-2565.92 (37.84)	0.875 (0.003)	0.867 - 0.885
SWG	5000	16	0.10	0.10	0.109 (0.002)	0.308 (0.023)	0.009 (0.002)	0.004 - 0.014	0 (0)	-15653.39 (110.28)	0.208 (0.023)	0.15 - 0.258
SWG	5000	16	0.10	0.20	0.112 (0.002)	0.364 (0.022)	0.012 (0.002)	0.008 - 0.017	0 (0)	-15571.2 (82.63)	0.164 (0.022)	0.111 - 0.233
SWG	5000	16	0.10	0.30	0.11 (0.002)	0.325 (0.027)	0.01 (0.002)	0.004 - 0.016	0 (0)	-15614.69 (93.56)	0.031 (0.02)	0 - 0.083
SWG	5000	16	0.10	0.40	0.113 (0.002)	0.381 (0.026)	0.013 (0.002)	0.007 - 0.017	0 (0)	-15555.89 (99.07)	0.025 (0.02)	0 - 0.096
SWG	5000	16	0.10	0.50	0.112 (0.002)	0.371 (0.025)	0.012 (0.002)	0.005 - 0.018	0 (0)	-15551.64 (98.73)	0.129 (0.025)	0.067 - 0.22
SWG	5000	16	0.10	0.60	0.11 (0.003)	0.326 (0.039)	0.01 (0.003)	0.002 - 0.016	0 (0)	-15630.11 (100.62)	0.274 (0.039)	0.206 - 0.394
SWG	5000	16	0.10	0.70	0.11 (0.003)	0.328 (0.032)	0.01 (0.003)	0.002 - 0.018	0 (0)	-15627.88 (102.57)	0.372 (0.032)	0.282 - 0.453
SWG	5000	16	0.10	0.80	0.111 (0.003)	0.36 (0.038)	0.011 (0.003)	0.004 - 0.017	0 (0)	-15571.34 (103.16)	0.44 (0.038)	0.368 - 0.542
SWG	5000	16	0.10	0.90	0.109 (0.005)	0.314 (0.062)	0.009 (0.004)	0 - 0.018	0 (0)	-15603.97 (108.69)	0.586 (0.062)	0.496 - 0.803
SWG	5000	16	0.20	0.10	0.216 (0.003)	0.329 (0.017)	0.016 (0.003)	0.009 - 0.022	0 (0)	-19185.75 (113.7)	0.229 (0.017)	0.192 - 0.262
SWG	5000	16	0.20	0.20	0.227 (0.003)	0.444 (0.016)	0.027 (0.003)	0.02 - 0.036	0 (0)	-18968.73 (108.31)	0.244 (0.016)	0.202 - 0.283
SWG	5000	16	0.20	0.30	0.218 (0.003)	0.346 (0.014)	0.018 (0.003)	0.011 - 0.023	0 (0)	-19100.26 (96.57)	0.046 (0.014)	0.008 - 0.075
SWG	5000	16	0.20	0.40	0.229 (0.003)	0.464 (0.016)	0.029 (0.003)	0.02 - 0.039	0 (0)	-18950.98 (105.46)	0.064 (0.016)	0.025 - 0.12

Continued on next page

Table S1 – *Continued from previous page*

Structure	T	N	p	p_e/p_w	$\overline{\hat{p}}$ (SD)	$\overline{\hat{p_e}}/\overline{\hat{p_w}}$ (SD)	$\overline{\lvert\Delta\rvert}_p$ (SD)	Range $\lvert\Delta\rvert_p$	$\overline{SE}\lvert\Delta\rvert_p$ (SD)	$\overline{BIC}$ (SD)	$\overline{\lvert\Delta\rvert}_{p_e/p_w}$ (SD)	Range $\lvert\Delta\rvert_{p_e/p_w}$
SWG	5000	16	0.20	0.50	0.23 (0.003)	0.468 (0.016)	0.03 (0.003)	0.023 - 0.037	0 (0)	-18925.96 (83.95)	0.032 (0.015)	0.003 - 0.079
SWG	5000	16	0.20	0.60	0.222 (0.003)	0.39 (0.017)	0.022 (0.003)	0.014 - 0.031	0 (0)	-19008.62 (104.91)	0.21 (0.017)	0.168 - 0.249
SWG	5000	16	0.20	0.70	0.223 (0.003)	0.398 (0.016)	0.023 (0.003)	0.015 - 0.031	0 (0)	-19036.8 (99.75)	0.302 (0.016)	0.264 - 0.335
SWG	5000	16	0.20	0.80	0.222 (0.003)	0.414 (0.016)	0.022 (0.003)	0.013 - 0.029	0 (0)	-18999.54 (104.83)	0.386 (0.016)	0.347 - 0.432
SWG	5000	16	0.20	0.90	0.215 (0.004)	0.351 (0.022)	0.015 (0.004)	0.007 - 0.025	0 (0)	-19033.4 (111.61)	0.549 (0.022)	0.483 - 0.618
SWG	5000	16	0.30	0.10	0.307 (0.003)	0.224 (0.009)	0.007 (0.003)	0.001 - 0.016	0 (0)	-20510.91 (88.55)	0.124 (0.009)	0.1 - 0.145
SWG	5000	16	0.30	0.20	0.317 (0.004)	0.3 (0.01)	0.017 (0.004)	0.008 - 0.029	0 (0)	-20418.07 (91.03)	0.1 (0.01)	0.075 - 0.124
SWG	5000	16	0.30	0.30	0.304 (0.003)	0.22 (0.008)	0.004 (0.002)	0 - 0.012	0 (0)	-20505.27 (87.92)	0.08 (0.008)	0.064 - 0.098
SWG	5000	16	0.30	0.40	0.313 (0.003)	0.298 (0.009)	0.013 (0.003)	0.004 - 0.021	0 (0)	-20431.27 (104.54)	0.102 (0.009)	0.08 - 0.125
SWG	5000	16	0.30	0.50	0.317 (0.003)	0.312 (0.01)	0.017 (0.003)	0.008 - 0.03	0 (0)	-20394.74 (113.29)	0.188 (0.01)	0.158 - 0.216
SWG	5000	16	0.30	0.60	0.303 (0.003)	0.231 (0.009)	0.003 (0.002)	0 - 0.011	0 (0)	-20493.75 (93.61)	0.369 (0.009)	0.348 - 0.388
SWG	5000	16	0.30	0.70	0.306 (0.004)	0.245 (0.01)	0.006 (0.003)	0 - 0.014	0 (0)	-20473.06 (85.62)	0.455 (0.01)	0.432 - 0.491
SWG	5000	16	0.30	0.80	0.299 (0.003)	0.236 (0.01)	0.003 (0.002)	0 - 0.011	0 (0)	-20501.64 (100.03)	0.564 (0.01)	0.533 - 0.582
SWG	5000	16	0.30	0.90	0.291 (0.004)	0.191 (0.01)	0.009 (0.004)	0 - 0.017	0 (0)	-20488.84 (100.86)	0.709 (0.01)	0.682 - 0.733
SWG	5000	16	0.40	0.10	0.396 (0.004)	0.111 (0.006)	0.004 (0.003)	0 - 0.013	0 (0)	-20985.44 (90.68)	0.011 (0.006)	0 - 0.029
SWG	5000	16	0.40	0.20	0.401 (0.004)	0.143 (0.007)	0.003 (0.002)	0 - 0.012	0 (0)	-20974.35 (87.82)	0.057 (0.007)	0.041 - 0.075
SWG	5000	16	0.40	0.30	0.394 (0.004)	0.107 (0.006)	0.007 (0.004)	0 - 0.017	0 (0)	-20977.38 (88.76)	0.193 (0.006)	0.179 - 0.206
SWG	5000	16	0.40	0.40	0.396 (0.003)	0.139 (0.006)	0.005 (0.003)	0 - 0.012	0 (0)	-20965.2 (103.15)	0.261 (0.006)	0.25 - 0.279
SWG	5000	16	0.40	0.50	0.398 (0.004)	0.145 (0.007)	0.004 (0.003)	0 - 0.011	0 (0)	-20963.83 (89.65)	0.355 (0.007)	0.341 - 0.368
SWG	5000	16	0.40	0.60	0.39 (0.003)	0.109 (0.007)	0.01 (0.003)	0.002 - 0.018	0 (0)	-20986.08 (96.9)	0.491 (0.007)	0.47 - 0.509
SWG	5000	16	0.40	0.70	0.393 (0.004)	0.117 (0.006)	0.007 (0.003)	0 - 0.015	0 (0)	-20962.42 (91.33)	0.583 (0.006)	0.571 - 0.601

Continued on next page

Table S1 – *Continued from previous page*

Structure	T	N	p	p_e/p_w	$\overline{\hat{p}}$ (SD)	$\overline{\hat{p_e}}/\overline{\hat{p_w}}$ (SD)	$\overline{\lvert\Delta\rvert}_p$ (SD)	Range $\lvert\Delta\rvert_p$	$\overline{SE}\lvert\Delta\rvert_p$ (SD)	$\overline{BIC}$ (SD)	$\overline{\lvert\Delta\rvert}_{p_e/p_w}$ (SD)	Range $\lvert\Delta\rvert_{p_e/p_w}$
SWG	5000	16	0.40	0.80	0.387 (0.004)	0.11 (0.006)	0.013 (0.004)	0.004 - 0.026	0 (0)	-21005.86 (99.28)	0.69 (0.006)	0.677 - 0.707
SWG	5000	16	0.40	0.90	0.383 (0.004)	0.091 (0.006)	0.017 (0.004)	0.009 - 0.029	0 (0)	-20995.71 (80.02)	0.809 (0.006)	0.792 - 0.825
SWG	5000	16	0.50	0.10	0.5 (0.005)	0.004 (0.003)	0.004 (0.003)	0 - 0.015	0 (0)	-21080.16 (98.87)	0.096 (0.003)	0.088 - 0.1
SWG	5000	16	0.50	0.20	0.5 (0.004)	0.004 (0.003)	0.003 (0.002)	0 - 0.01	0 (0)	-21100.52 (89.67)	0.196 (0.003)	0.187 - 0.2
SWG	5000	16	0.50	0.30	0.5 (0.004)	0.004 (0.003)	0.003 (0.003)	0 - 0.015	0 (0)	-21107.68 (112.41)	0.296 (0.003)	0.287 - 0.3
SWG	5000	16	0.50	0.40	0.5 (0.004)	0.004 (0.003)	0.003 (0.003)	0 - 0.017	0 (0)	-21102.09 (90.65)	0.396 (0.003)	0.388 - 0.4
SWG	5000	16	0.50	0.50	0.5 (0.004)	0.004 (0.003)	0.003 (0.003)	0 - 0.011	0 (0)	-21106.65 (96.44)	0.496 (0.003)	0.484 - 0.5
SWG	5000	16	0.50	0.60	0.499 (0.004)	0.004 (0.003)	0.003 (0.002)	0 - 0.013	0 (0)	-21110.69 (102.32)	0.596 (0.003)	0.585 - 0.6
SWG	5000	16	0.50	0.70	0.501 (0.004)	0.004 (0.003)	0.003 (0.003)	0 - 0.012	0 (0)	-21094.9 (97.56)	0.696 (0.003)	0.688 - 0.7
SWG	5000	16	0.50	0.80	0.5 (0.005)	0.004 (0.003)	0.004 (0.003)	0 - 0.011	0 (0)	-21073.15 (103.46)	0.796 (0.003)	0.785 - 0.8
SWG	5000	16	0.50	0.90	0.5 (0.004)	0.004 (0.003)	0.004 (0.003)	0 - 0.011	0 (0)	-21095.24 (84.18)	0.896 (0.003)	0.886 - 0.9
SWG	5000	16	0.60	0.10	0.614 (0.004)	0.108 (0.005)	0.014 (0.004)	0.004 - 0.023	0 (0)	-20991.83 (92.45)	0.008 (0.004)	0 - 0.019
SWG	5000	16	0.60	0.20	0.617 (0.004)	0.136 (0.005)	0.017 (0.004)	0.007 - 0.028	0 (0)	-20977.58 (94.41)	0.064 (0.005)	0.046 - 0.078
SWG	5000	16	0.60	0.30	0.618 (0.004)	0.106 (0.005)	0.018 (0.004)	0.006 - 0.03	0 (0)	-20999.04 (93.71)	0.194 (0.005)	0.18 - 0.205
SWG	5000	16	0.60	0.40	0.623 (0.004)	0.137 (0.005)	0.023 (0.004)	0.01 - 0.038	0 (0)	-21023.31 (94.31)	0.263 (0.005)	0.247 - 0.275
SWG	5000	16	0.60	0.50	0.621 (0.005)	0.141 (0.006)	0.021 (0.005)	0.01 - 0.036	0 (0)	-20998.45 (98.78)	0.359 (0.006)	0.341 - 0.373
SWG	5000	16	0.60	0.60	0.624 (0.004)	0.11 (0.005)	0.024 (0.004)	0.013 - 0.034	0 (0)	-21032.86 (93.85)	0.49 (0.005)	0.475 - 0.502
SWG	5000	16	0.60	0.70	0.621 (0.005)	0.113 (0.005)	0.021 (0.005)	0.009 - 0.032	0 (0)	-21016.48 (88.66)	0.587 (0.005)	0.572 - 0.601

Continued on next page

Table S1 – *Continued from previous page*

Structure	T	N	p	p_e/p_w	$\overline{\hat{p}}$ (SD)	$\overline{\hat{p_e}}/\overline{\hat{p_w}}$ (SD)	$\overline{\lvert\Delta\rvert}_p$ (SD)	Range $\lvert\Delta\rvert_p$	$\overline{SE}\lvert\Delta\rvert_p$ (SD)	$\overline{BIC}$ (SD)	$\overline{\lvert\Delta\rvert}_{p_e/p_w}$ (SD)	Range $\lvert\Delta\rvert_{p_e/p_w}$
SWG	5000	16	0.60	0.80	0.628 (0.004)	0.109 (0.005)	0.028 (0.004)	0.017 - 0.038	0 (0)	-21026.09 (91.32)	0.691 (0.005)	0.679 - 0.707
SWG	5000	16	0.60	0.90	0.628 (0.004)	0.088 (0.005)	0.028 (0.004)	0.017 - 0.037	0 (0)	-20995.7 (92.45)	0.812 (0.005)	0.8 - 0.827
SWG	5000	16	0.70	0.10	0.723 (0.004)	0.19 (0.005)	0.023 (0.004)	0.012 - 0.033	0 (0)	-20729.97 (85.09)	0.09 (0.005)	0.079 - 0.101
SWG	5000	16	0.70	0.20	0.73 (0.004)	0.243 (0.005)	0.03 (0.004)	0.02 - 0.042	0 (0)	-20791.41 (79.76)	0.043 (0.005)	0.03 - 0.055
SWG	5000	16	0.70	0.30	0.729 (0.004)	0.184 (0.006)	0.029 (0.004)	0.019 - 0.037	0 (0)	-20716 (95.9)	0.116 (0.006)	0.104 - 0.13
SWG	5000	16	0.70	0.40	0.742 (0.004)	0.252 (0.005)	0.042 (0.004)	0.032 - 0.051	0 (0)	-20853 (95.66)	0.148 (0.005)	0.137 - 0.167
SWG	5000	16	0.70	0.50	0.74 (0.004)	0.258 (0.005)	0.04 (0.004)	0.029 - 0.05	0 (0)	-20800.8 (106.62)	0.242 (0.005)	0.231 - 0.255
SWG	5000	16	0.70	0.60	0.744 (0.003)	0.201 (0.005)	0.044 (0.003)	0.035 - 0.052	0 (0)	-20820.11 (94.39)	0.399 (0.005)	0.387 - 0.411
SWG	5000	16	0.70	0.70	0.737 (0.004)	0.205 (0.005)	0.037 (0.004)	0.029 - 0.047	0 (0)	-20738.64 (97.76)	0.495 (0.005)	0.484 - 0.506
SWG	5000	16	0.70	0.80	0.749 (0.004)	0.195 (0.005)	0.049 (0.004)	0.041 - 0.057	0 (0)	-20859.48 (106.97)	0.605 (0.005)	0.592 - 0.62
SWG	5000	16	0.70	0.90	0.747 (0.003)	0.154 (0.005)	0.047 (0.003)	0.04 - 0.054	0 (0)	-20656.79 (111.57)	0.746 (0.005)	0.733 - 0.757
SWG	5000	16	0.80	0.10	0.809 (0.004)	0.198 (0.005)	0.009 (0.004)	0 - 0.017	0 (0)	-20449.21 (79.97)	0.098 (0.005)	0.088 - 0.109
SWG	5000	16	0.80	0.20	0.82 (0.004)	0.262 (0.005)	0.02 (0.004)	0.011 - 0.027	0 (0)	-20610.69 (100.91)	0.062 (0.005)	0.05 - 0.073
SWG	5000	16	0.80	0.30	0.824 (0.003)	0.196 (0.004)	0.024 (0.003)	0.018 - 0.031	0 (0)	-20217.75 (88.87)	0.104 (0.004)	0.092 - 0.113
SWG	5000	16	0.80	0.40	0.836 (0.003)	0.281 (0.005)	0.036 (0.003)	0.029 - 0.043	0 (0)	-20789.48 (101.92)	0.119 (0.005)	0.107 - 0.132
SWG	5000	16	0.80	0.50	0.838 (0.003)	0.287 (0.005)	0.038 (0.003)	0.031 - 0.044	0 (0)	-20668.44 (102.53)	0.213 (0.005)	0.2 - 0.226
SWG	5000	16	0.80	0.60	0.841 (0.002)	0.204 (0.005)	0.041 (0.002)	0.036 - 0.047	0 (0)	-20437.2 (101.64)	0.396 (0.005)	0.384 - 0.412
SWG	5000	16	0.80	0.70	0.833 (0.003)	0.224 (0.005)	0.033 (0.003)	0.026 - 0.04	0 (0)	-20337.43 (98.99)	0.476 (0.005)	0.462 - 0.488

Continued on next page

Table S1 – *Continued from previous page*

Structure	T	N	p	p_e/p_w	$\overline{\hat{p}}$ (SD)	$\overline{\hat{p}_e}/\overline{\hat{p}_w}$ (SD)	$\overline{\lvert\Delta\rvert}_p$ (SD)	Range $\lvert\Delta\rvert_p$	$\overline{SE}\lvert\Delta\rvert_p$ (SD)	$\overline{BIC}$ (SD)	$\overline{\lvert\Delta\rvert}_{p_e/p_w}$ (SD)	Range $\lvert\Delta\rvert_{p_e/p_w}$
SWG	5000	16	0.80	0.80	0.843 (0.002)	0.191 (0.005)	0.043 (0.002)	0.038 - 0.05	0 (0)	-20444.02 (119.51)	0.609 (0.005)	0.592 - 0.622
SWG	5000	16	0.80	0.90	0.84 (0.002)	0.139 (0.005)	0.04 (0.002)	0.034 - 0.046	0 (0)	-19930.76 (99.72)	0.761 (0.005)	0.75 - 0.772
SWG	5000	16	0.90	0.10	0.891 (0.005)	0.089 (0.004)	0.009 (0.005)	0 - 0.027	0 (0)	-19446.27 (153.95)	0.011 (0.004)	0.002 - 0.022
SWG	5000	16	0.90	0.20	0.903 (0.003)	0.137 (0.006)	0.004 (0.003)	0 - 0.01	0 (0)	-19492.75 (156.05)	0.063 (0.006)	0.051 - 0.077
SWG	5000	16	0.90	0.30	0.91 (0.002)	0.078 (0.005)	0.01 (0.002)	0.006 - 0.015	0 (0)	-17971.23 (190.96)	0.222 (0.005)	0.21 - 0.232
SWG	5000	16	0.90	0.40	0.916 (0.002)	0.135 (0.008)	0.016 (0.002)	0.012 - 0.021	0 (0)	-19246.86 (249.41)	0.265 (0.008)	0.247 - 0.283
SWG	5000	16	0.90	0.50	0.917 (0.001)	0.121 (0.006)	0.017 (0.001)	0.014 - 0.02	0 (0)	-18382.39 (190.81)	0.379 (0.006)	0.36 - 0.39
SWG	5000	16	0.90	0.60	0.915 (0.001)	0.06 (0.004)	0.015 (0.001)	0.012 - 0.019	0 (0)	-16970.02 (162.93)	0.54 (0.004)	0.529 - 0.549
SWG	5000	16	0.90	0.70	0.913 (0.001)	0.093 (0.004)	0.013 (0.001)	0.009 - 0.016	0 (0)	-17728.61 (160.07)	0.607 (0.004)	0.595 - 0.62
SWG	5000	16	0.90	0.80	0.916 (0.001)	0.057 (0.004)	0.016 (0.001)	0.012 - 0.02	0 (0)	-16897.36 (167.74)	0.743 (0.004)	0.736 - 0.751
SWG	5000	16	0.90	0.90	0.914 (0.001)	0.036 (0.004)	0.014 (0.001)	0.011 - 0.017	0 (0)	-16294.16 (133.42)	0.864 (0.004)	0.852 - 0.873
SWG	5000	25	0.10	0.10	0.111 (0.002)	0.265 (0.02)	0.011 (0.002)	0.005 - 0.015	0 (0)	-18035.57 (97.83)	0.165 (0.02)	0.09 - 0.21
SWG	5000	25	0.10	0.20	0.111 (0.002)	0.258 (0.019)	0.011 (0.002)	0.003 - 0.014	0 (0)	-18038.23 (102.97)	0.058 (0.017)	0.007 - 0.091
SWG	5000	25	0.10	0.30	0.111 (0.002)	0.261 (0.026)	0.011 (0.002)	0.004 - 0.016	0 (0)	-18025.11 (94.77)	0.04 (0.024)	0 - 0.117
SWG	5000	25	0.10	0.40	0.111 (0.002)	0.27 (0.024)	0.011 (0.002)	0.004 - 0.017	0 (0)	-18010.73 (100.02)	0.13 (0.024)	0.07 - 0.208
SWG	5000	25	0.10	0.50	0.111 (0.003)	0.272 (0.033)	0.011 (0.003)	0 - 0.016	0 (0)	-18001.22 (89.33)	0.228 (0.033)	0.16 - 0.356
SWG	5000	25	0.10	0.60	0.11 (0.003)	0.258 (0.038)	0.01 (0.003)	0 - 0.017	0 (0)	-18022.02 (98.7)	0.342 (0.038)	0.275 - 0.455
SWG	5000	25	0.10	0.70	0.109 (0.004)	0.254 (0.052)	0.009 (0.004)	0 - 0.015	0 (0)	-18003.89 (96.29)	0.446 (0.052)	0.372 - 0.621
SWG	5000	25	0.10	0.80	0.109 (0.004)	0.246 (0.049)	0.009 (0.004)	0 - 0.015	0 (0)	-18025.64 (94.01)	0.554 (0.049)	0.474 - 0.71

Continued on next page

Table S1 – *Continued from previous page*

Structure	T	N	p	p_e/p_w	$\overline{\hat{p}}$ (SD)	$\overline{\hat{p}_e}/\overline{\hat{p}_w}$ (SD)	$\overline{\|\Delta\|}_p$ (SD)	Range $\|\Delta\|_p$	$\overline{SE}\|\Delta\|_p$ (SD)	$\overline{BIC}$ (SD)	$\overline{\|\Delta\|}_{p_e/p_w}$ (SD)	Range $\|\Delta\|_{p_e/p_w}$
SWG	5000	25	0.10	0.90	0.108 (0.004)	0.239 (0.053)	0.008 (0.004)	0 - 0.015	0 (0)	-18001.46 (114.89)	0.661 (0.053)	0.592 - 0.807
SWG	5000	25	0.20	0.10	0.23 (0.003)	0.361 (0.012)	0.03 (0.003)	0.025 - 0.037	0 (0)	-21283.17 (111)	0.261 (0.012)	0.237 - 0.288
SWG	5000	25	0.20	0.20	0.227 (0.002)	0.34 (0.011)	0.027 (0.002)	0.02 - 0.032	0 (0)	-21291 (99.71)	0.14 (0.011)	0.103 - 0.16
SWG	5000	25	0.20	0.30	0.226 (0.003)	0.34 (0.013)	0.026 (0.003)	0.019 - 0.034	0 (0)	-21253.09 (100.59)	0.04 (0.013)	0.005 - 0.07
SWG	5000	25	0.20	0.40	0.228 (0.003)	0.366 (0.016)	0.028 (0.003)	0.021 - 0.038	0 (0)	-21233.75 (101.63)	0.035 (0.015)	0.001 - 0.077
SWG	5000	25	0.20	0.50	0.229 (0.003)	0.369 (0.012)	0.029 (0.003)	0.024 - 0.036	0 (0)	-21242.06 (102.38)	0.131 (0.012)	0.095 - 0.162
SWG	5000	25	0.20	0.60	0.228 (0.003)	0.368 (0.013)	0.028 (0.003)	0.021 - 0.039	0 (0)	-21240.69 (115.1)	0.232 (0.013)	0.185 - 0.264
SWG	5000	25	0.20	0.70	0.226 (0.003)	0.361 (0.013)	0.026 (0.003)	0.02 - 0.033	0 (0)	-21235.62 (103.78)	0.339 (0.013)	0.31 - 0.37
SWG	5000	25	0.20	0.80	0.225 (0.003)	0.346 (0.017)	0.025 (0.003)	0.016 - 0.032	0 (0)	-21219.32 (110.35)	0.454 (0.017)	0.415 - 0.499
SWG	5000	25	0.20	0.90	0.222 (0.003)	0.331 (0.016)	0.022 (0.003)	0.012 - 0.029	0 (0)	-21253.36 (97.5)	0.569 (0.016)	0.536 - 0.608
SWG	5000	25	0.30	0.10	0.321 (0.003)	0.25 (0.008)	0.021 (0.003)	0.013 - 0.031	0 (0)	-22673 (97.05)	0.15 (0.008)	0.13 - 0.174
SWG	5000	25	0.30	0.20	0.319 (0.004)	0.236 (0.007)	0.019 (0.004)	0.011 - 0.028	0 (0)	-22676.92 (91.89)	0.036 (0.007)	0.016 - 0.054
SWG	5000	25	0.30	0.30	0.312 (0.003)	0.218 (0.007)	0.012 (0.003)	0.004 - 0.019	0 (0)	-22657.61 (97.5)	0.082 (0.007)	0.067 - 0.096
SWG	5000	25	0.30	0.40	0.315 (0.003)	0.237 (0.007)	0.015 (0.003)	0.007 - 0.023	0 (0)	-22680.79 (84.72)	0.163 (0.007)	0.148 - 0.18
SWG	5000	25	0.30	0.50	0.312 (0.003)	0.233 (0.008)	0.012 (0.003)	0.003 - 0.021	0 (0)	-22678.81 (95.25)	0.267 (0.008)	0.24 - 0.287
SWG	5000	25	0.30	0.60	0.31 (0.004)	0.228 (0.008)	0.01 (0.004)	0.002 - 0.019	0 (0)	-22714.93 (98.42)	0.372 (0.008)	0.348 - 0.397
SWG	5000	25	0.30	0.70	0.306 (0.003)	0.214 (0.009)	0.006 (0.003)	0 - 0.013	0 (0)	-22710.95 (103.29)	0.486 (0.009)	0.462 - 0.508
SWG	5000	25	0.30	0.80	0.304 (0.003)	0.202 (0.008)	0.004 (0.003)	0 - 0.015	0 (0)	-22697.61 (102.32)	0.598 (0.008)	0.573 - 0.619
SWG	5000	25	0.30	0.90	0.298 (0.003)	0.182 (0.008)	0.003 (0.002)	0 - 0.009	0 (0)	-22778.9 (93.5)	0.718 (0.008)	0.695 - 0.734

Continued on next page

Table S1 – *Continued from previous page*

Structure	T	N	p	p_e/p_w	$\overline{\hat{p}}$ (SD)	$\overline{\hat{p_e}}/\overline{\hat{p_w}}$ (SD)	$\overline{\lvert\Delta\rvert}_p$ (SD)	Range $\lvert\Delta\rvert_p$	$\overline{SE}\lvert\Delta\rvert_p$ (SD)	$\overline{BIC}$ (SD)	$\overline{\lvert\Delta\rvert}_{p_e/p_w}$ (SD)	Range $\lvert\Delta\rvert_{p_e/p_w}$
SWG	5000	25	0.40	0.10	0.403 (0.003)	0.119 (0.004)	0.004 (0.003)	0 - 0.01	0 (0)	-23193.62 (98.4)	0.019 (0.004)	0.008 - 0.029
SWG	5000	25	0.40	0.20	0.402 (0.003)	0.113 (0.005)	0.003 (0.002)	0 - 0.009	0 (0)	-23187.12 (95.66)	0.087 (0.005)	0.075 - 0.099
SWG	5000	25	0.40	0.30	0.395 (0.003)	0.101 (0.004)	0.005 (0.003)	0 - 0.013	0 (0)	-23193 (97.76)	0.199 (0.004)	0.187 - 0.209
SWG	5000	25	0.40	0.40	0.397 (0.004)	0.109 (0.004)	0.004 (0.003)	0 - 0.011	0 (0)	-23210.79 (92.19)	0.291 (0.004)	0.283 - 0.305
SWG	5000	25	0.40	0.50	0.393 (0.004)	0.106 (0.004)	0.007 (0.004)	0 - 0.014	0 (0)	-23198.26 (97.68)	0.394 (0.004)	0.383 - 0.403
SWG	5000	25	0.40	0.60	0.393 (0.003)	0.103 (0.004)	0.008 (0.003)	0 - 0.017	0 (0)	-23219.43 (102.83)	0.497 (0.004)	0.488 - 0.508
SWG	5000	25	0.40	0.70	0.389 (0.003)	0.095 (0.005)	0.011 (0.003)	0.003 - 0.02	0 (0)	-23230.84 (89.44)	0.605 (0.005)	0.592 - 0.619
SWG	5000	25	0.40	0.80	0.389 (0.004)	0.091 (0.004)	0.011 (0.004)	0.003 - 0.022	0 (0)	-23202.08 (96.63)	0.709 (0.004)	0.701 - 0.717
SWG	5000	25	0.40	0.90	0.385 (0.004)	0.081 (0.004)	0.015 (0.004)	0.005 - 0.024	0 (0)	-23232.84 (91.99)	0.819 (0.004)	0.811 - 0.828
SWG	5000	25	0.50	0.10	0.5 (0.004)	0.003 (0.002)	0.003 (0.003)	0 - 0.013	0 (0)	-23333.59 (91.79)	0.097 (0.002)	0.091 - 0.1
SWG	5000	25	0.50	0.20	0.5 (0.005)	0.003 (0.002)	0.004 (0.003)	0 - 0.014	0 (0)	-23321.31 (103.93)	0.197 (0.002)	0.188 - 0.2
SWG	5000	25	0.50	0.30	0.5 (0.004)	0.003 (0.002)	0.003 (0.003)	0 - 0.012	0 (0)	-23325.85 (110.98)	0.297 (0.002)	0.289 - 0.3
SWG	5000	25	0.50	0.40	0.5 (0.004)	0.003 (0.002)	0.003 (0.003)	0 - 0.013	0 (0)	-23315.57 (99.5)	0.397 (0.002)	0.388 - 0.4
SWG	5000	25	0.50	0.50	0.5 (0.004)	0.003 (0.002)	0.004 (0.002)	0 - 0.01	0 (0)	-23331.22 (98.94)	0.497 (0.002)	0.488 - 0.5
SWG	5000	25	0.50	0.60	0.5 (0.004)	0.003 (0.002)	0.003 (0.002)	0 - 0.01	0 (0)	-23341.3 (88.64)	0.597 (0.002)	0.591 - 0.6
SWG	5000	25	0.50	0.70	0.5 (0.004)	0.003 (0.002)	0.003 (0.003)	0 - 0.011	0 (0)	-23321.17 (100.65)	0.697 (0.002)	0.69 - 0.7
SWG	5000	25	0.50	0.80	0.501 (0.004)	0.003 (0.002)	0.004 (0.003)	0 - 0.011	0 (0)	-23343.75 (93.74)	0.797 (0.002)	0.79 - 0.8

Continued on next page

Table S1 – *Continued from previous page*

Structure	T	N	p	p_e/p_w	$\overline{\hat{p}}$ (SD)	$\overline{\hat{p}_e}/\overline{\hat{p}_w}$ (SD)	$\overline{\lvert\Delta\rvert}_p$ (SD)	Range $\lvert\Delta\rvert_p$	$\overline{SE}\lvert\Delta\rvert_p$ (SD)	$\overline{BIC}$ (SD)	$\overline{\lvert\Delta\rvert}_{p_e/p_w}$ (SD)	Range $\lvert\Delta\rvert_{p_e/p_w}$
SWG	5000	25	0.50	0.90	0.5 (0.004)	0.003 (0.002)	0.003 (0.002)	0 - 0.008	0 (0)	-23316.51 (86.3)	0.897 (0.002)	0.887 - 0.9
SWG	5000	25	0.60	0.10	0.613 (0.004)	0.113 (0.003)	0.013 (0.004)	0.001 - 0.022	0 (0)	-23240.47 (104)	0.013 (0.003)	0.005 - 0.021
SWG	5000	25	0.60	0.20	0.613 (0.004)	0.11 (0.003)	0.013 (0.004)	0.004 - 0.022	0 (0)	-23209.96 (92.85)	0.09 (0.003)	0.083 - 0.097
SWG	5000	25	0.60	0.30	0.619 (0.005)	0.099 (0.003)	0.019 (0.005)	0.008 - 0.029	0 (0)	-23237.5 (104.73)	0.201 (0.003)	0.192 - 0.21
SWG	5000	25	0.60	0.40	0.62 (0.004)	0.108 (0.003)	0.02 (0.004)	0.01 - 0.03	0 (0)	-23243.58 (90.38)	0.292 (0.003)	0.282 - 0.302
SWG	5000	25	0.60	0.50	0.624 (0.004)	0.104 (0.004)	0.024 (0.004)	0.014 - 0.036	0 (0)	-23237.57 (98.69)	0.396 (0.004)	0.389 - 0.405
SWG	5000	25	0.60	0.60	0.625 (0.004)	0.103 (0.003)	0.025 (0.004)	0.017 - 0.033	0 (0)	-23256.59 (101.2)	0.497 (0.003)	0.487 - 0.504
SWG	5000	25	0.60	0.70	0.628 (0.004)	0.092 (0.004)	0.028 (0.004)	0.014 - 0.039	0 (0)	-23260.57 (101.47)	0.608 (0.004)	0.599 - 0.618
SWG	5000	25	0.60	0.80	0.626 (0.004)	0.091 (0.004)	0.026 (0.004)	0.016 - 0.034	0 (0)	-23237.91 (102.33)	0.709 (0.004)	0.7 - 0.717
SWG	5000	25	0.60	0.90	0.628 (0.004)	0.081 (0.004)	0.028 (0.004)	0.016 - 0.036	0 (0)	-23248.09 (90.23)	0.819 (0.004)	0.808 - 0.83
SWG	5000	25	0.70	0.10	0.725 (0.003)	0.208 (0.003)	0.025 (0.003)	0.014 - 0.035	0 (0)	-22934.82 (86.55)	0.108 (0.003)	0.098 - 0.115
SWG	5000	25	0.70	0.20	0.726 (0.004)	0.201 (0.004)	0.026 (0.004)	0.017 - 0.034	0 (0)	-22914.42 (88.5)	0.003 (0.002)	0 - 0.008
SWG	5000	25	0.70	0.30	0.735 (0.004)	0.184 (0.003)	0.035 (0.004)	0.026 - 0.044	0 (0)	-22974.53 (102.83)	0.116 (0.003)	0.108 - 0.123
SWG	5000	25	0.70	0.40	0.739 (0.004)	0.204 (0.003)	0.039 (0.004)	0.031 - 0.048	0 (0)	-23042.5 (101.59)	0.196 (0.003)	0.189 - 0.203
SWG	5000	25	0.70	0.50	0.743 (0.003)	0.187 (0.003)	0.043 (0.003)	0.033 - 0.052	0 (0)	-23036.91 (109.24)	0.313 (0.003)	0.305 - 0.321
SWG	5000	25	0.70	0.60	0.748 (0.003)	0.194 (0.003)	0.048 (0.003)	0.04 - 0.054	0 (0)	-23142.57 (103.2)	0.406 (0.003)	0.4 - 0.414
SWG	5000	25	0.70	0.70	0.75 (0.003)	0.166 (0.003)	0.05 (0.003)	0.042 - 0.056	0 (0)	-23049.96 (106.53)	0.534 (0.003)	0.526 - 0.542
SWG	5000	25	0.70	0.80	0.748 (0.004)	0.164 (0.004)	0.048 (0.004)	0.039 - 0.056	0 (0)	-22985.93 (92.66)	0.636 (0.004)	0.628 - 0.644

Continued on next page

Table S1 – *Continued from previous page*

Structure	T	N	p	p_e/p_w	$\overline{\hat{p}}$ (SD)	$\overline{\hat{p}_e}/\overline{\hat{p}_w}$ (SD)	$\overline{\lvert\Delta\rvert}_p$ (SD)	Range $\lvert\Delta\rvert_p$	$\overline{SE}\lvert\Delta\rvert_p$ (SD)	$\overline{BIC}$ (SD)	$\overline{\lvert\Delta\rvert}_{p_e/p_w}$ (SD)	Range $\lvert\Delta\rvert_{p_e/p_w}$
SWG	5000	25	0.70	0.90	0.748 (0.003)	0.144 (0.003)	0.048 (0.003)	0.04 - 0.056	0 (0)	-23007.57 (93.84)	0.756 (0.003)	0.747 - 0.765
SWG	5000	25	0.80	0.10	0.819 (0.003)	0.248 (0.003)	0.019 (0.003)	0.011 - 0.025	0 (0)	-22589.23 (102.7)	0.148 (0.003)	0.141 - 0.155
SWG	5000	25	0.80	0.20	0.821 (0.003)	0.239 (0.003)	0.021 (0.003)	0.015 - 0.029	0 (0)	-22590.67 (108.64)	0.039 (0.003)	0.032 - 0.047
SWG	5000	25	0.80	0.30	0.833 (0.002)	0.209 (0.004)	0.033 (0.002)	0.028 - 0.039	0 (0)	-22694.89 (103.61)	0.091 (0.004)	0.081 - 0.1
SWG	5000	25	0.80	0.40	0.837 (0.002)	0.234 (0.004)	0.037 (0.002)	0.031 - 0.043	0 (0)	-23043.6 (122.34)	0.166 (0.004)	0.154 - 0.176
SWG	5000	25	0.80	0.50	0.839 (0.002)	0.194 (0.003)	0.039 (0.002)	0.032 - 0.044	0 (0)	-22848.36 (102.05)	0.306 (0.003)	0.297 - 0.313
SWG	5000	25	0.80	0.60	0.844 (0.002)	0.199 (0.004)	0.044 (0.002)	0.038 - 0.048	0 (0)	-23183.14 (125.9)	0.401 (0.004)	0.391 - 0.41
SWG	5000	25	0.80	0.70	0.845 (0.001)	0.154 (0.003)	0.045 (0.001)	0.042 - 0.048	0 (0)	-22760.27 (94.15)	0.546 (0.003)	0.536 - 0.554
SWG	5000	25	0.80	0.80	0.843 (0.001)	0.156 (0.004)	0.043 (0.001)	0.039 - 0.047	0 (0)	-22571.66 (114.39)	0.644 (0.004)	0.635 - 0.652
SWG	5000	25	0.80	0.90	0.842 (0.001)	0.133 (0.003)	0.042 (0.001)	0.038 - 0.045	0 (0)	-22449.42 (94.94)	0.767 (0.003)	0.757 - 0.775
SWG	5000	25	0.90	0.10	0.908 (0.001)	0.121 (0.005)	0.008 (0.001)	0.004 - 0.011	0 (0)	-21663 (216.48)	0.021 (0.005)	0.009 - 0.035
SWG	5000	25	0.90	0.20	0.909 (0.002)	0.106 (0.005)	0.009 (0.002)	0.003 - 0.013	0 (0)	-21358.67 (217.97)	0.094 (0.005)	0.076 - 0.107
SWG	5000	25	0.90	0.30	0.911 (0.001)	0.063 (0.003)	0.011 (0.001)	0.008 - 0.014	0 (0)	-19902.49 (199.08)	0.237 (0.003)	0.227 - 0.245
SWG	5000	25	0.90	0.40	0.913 (0.001)	0.073 (0.003)	0.013 (0.001)	0.011 - 0.016	0 (0)	-20315.67 (209.15)	0.327 (0.003)	0.32 - 0.335
SWG	5000	25	0.90	0.50	0.913 (0.001)	0.05 (0.003)	0.013 (0.001)	0.01 - 0.015	0 (0)	-19507.44 (152.2)	0.45 (0.003)	0.443 - 0.458
SWG	5000	25	0.90	0.60	0.914 (0.001)	0.051 (0.003)	0.014 (0.001)	0.012 - 0.017	0 (0)	-19445.59 (176.68)	0.549 (0.003)	0.543 - 0.555
SWG	5000	25	0.90	0.70	0.913 (0.001)	0.034 (0.003)	0.013 (0.001)	0.01 - 0.016	0 (0)	-18874.95 (121)	0.666 (0.003)	0.66 - 0.673
SWG	5000	25	0.90	0.80	0.913 (0.001)	0.037 (0.002)	0.013 (0.001)	0.011 - 0.016	0 (0)	-18812.01 (125.8)	0.763 (0.002)	0.757 - 0.77

Continued on next page

Table S1 – *Continued from previous page*

Structure	T	N	p	p_e/p_w	$\overline{\hat{p}}$ (SD)	$\overline{\hat{p}_e}/\overline{\hat{p}_w}$ (SD)	$\overline{\lvert\Delta\rvert}_p$ (SD)	Range $\lvert\Delta\rvert_p$	$\overline{SE}\lvert\Delta\rvert_p$ (SD)	$\overline{BIC}$ (SD)	$\overline{\lvert\Delta\rvert}_{p_e/p_w}$ (SD)	Range $\lvert\Delta\rvert_{p_e/p_w}$
SWG	5000	25	0.90	0.90	0.912 (0.001)	0.033 (0.002)	0.012 (0.001)	0.01 - 0.015	0 (0)	-18742.64 (122.05)	0.867 (0.002)	0.861 - 0.873
SWG	5000	49	0.10	0.10	0.11 (0.002)	0.19 (0.015)	0.01 (0.002)	0.006 - 0.015	0 (0)	-21585.76 (114.65)	0.09 (0.015)	0.055 - 0.125
SWG	5000	49	0.10	0.20	0.11 (0.002)	0.2 (0.016)	0.01 (0.002)	0.007 - 0.014	0 (0)	-21575.71 (109.05)	0.013 (0.009)	0 - 0.037
SWG	5000	49	0.10	0.30	0.11 (0.002)	0.207 (0.018)	0.01 (0.002)	0.004 - 0.015	0 (0)	-21546.61 (105.82)	0.093 (0.018)	0.057 - 0.155
SWG	5000	49	0.10	0.40	0.111 (0.002)	0.213 (0.018)	0.011 (0.002)	0.007 - 0.014	0 (0)	-21541.57 (108.1)	0.187 (0.018)	0.142 - 0.232
SWG	5000	49	0.10	0.50	0.111 (0.002)	0.219 (0.021)	0.011 (0.002)	0.003 - 0.015	0 (0)	-21519.93 (96.16)	0.281 (0.021)	0.24 - 0.359
SWG	5000	49	0.10	0.60	0.108 (0.004)	0.185 (0.048)	0.008 (0.004)	0 - 0.015	0 (0)	-21538.62 (110.78)	0.415 (0.048)	0.337 - 0.527
SWG	5000	49	0.10	0.70	0.11 (0.003)	0.211 (0.032)	0.01 (0.003)	0.002 - 0.017	0 (0)	-21528.21 (101.54)	0.489 (0.032)	0.422 - 0.573
SWG	5000	49	0.10	0.80	0.108 (0.005)	0.186 (0.058)	0.008 (0.005)	0 - 0.016	0 (0)	-21566.46 (113.84)	0.614 (0.058)	0.532 - 0.76
SWG	5000	49	0.10	0.90	0.107 (0.006)	0.18 (0.064)	0.008 (0.005)	0 - 0.015	0 (0)	-21563.27 (109.04)	0.72 (0.064)	0.636 - 0.857
SWG	5000	49	0.20	0.10	0.228 (0.002)	0.279 (0.011)	0.028 (0.002)	0.022 - 0.034	0 (0)	-24734.8 (92.95)	0.179 (0.011)	0.158 - 0.201
SWG	5000	49	0.20	0.20	0.23 (0.003)	0.298 (0.011)	0.03 (0.003)	0.022 - 0.037	0 (0)	-24691.45 (99.46)	0.098 (0.011)	0.065 - 0.129
SWG	5000	49	0.20	0.30	0.23 (0.003)	0.31 (0.012)	0.03 (0.003)	0.024 - 0.036	0 (0)	-24675.48 (106.77)	0.013 (0.009)	0 - 0.034
SWG	5000	49	0.20	0.40	0.231 (0.003)	0.318 (0.014)	0.031 (0.003)	0.022 - 0.038	0 (0)	-24631.8 (118.06)	0.082 (0.014)	0.05 - 0.116
SWG	5000	49	0.20	0.50	0.232 (0.003)	0.334 (0.014)	0.032 (0.003)	0.025 - 0.038	0 (0)	-24607.42 (98.7)	0.166 (0.014)	0.137 - 0.197
SWG	5000	49	0.20	0.60	0.228 (0.003)	0.309 (0.015)	0.028 (0.003)	0.017 - 0.034	0 (0)	-24611.46 (108.89)	0.291 (0.015)	0.258 - 0.333
SWG	5000	49	0.20	0.70	0.232 (0.004)	0.343 (0.016)	0.032 (0.004)	0.02 - 0.039	0 (0)	-24603.9 (101.9)	0.357 (0.016)	0.33 - 0.414
SWG	5000	49	0.20	0.80	0.228 (0.004)	0.314 (0.018)	0.028 (0.004)	0.018 - 0.038	0 (0)	-24623.61 (105.68)	0.486 (0.018)	0.452 - 0.536
SWG	5000	49	0.20	0.90	0.232 (0.004)	0.344 (0.018)	0.032 (0.004)	0.016 - 0.04	0 (0)	-24584.98 (106.72)	0.556 (0.018)	0.518 - 0.626

Continued on next page

Table S1 – *Continued from previous page*

Structure	T	N	p	p_e/p_w	$\overline{\hat{p}}$ (SD)	$\overline{\hat{p_e}}/\overline{\hat{p_w}}$ (SD)	$\overline{\lvert\Delta\rvert}_p$ (SD)	Range $\lvert\Delta\rvert_p$	$\overline{SE}\lvert\Delta\rvert_p$ (SD)	$\overline{BIC}$ (SD)	$\overline{\lvert\Delta\rvert}_{p_e/p_w}$ (SD)	Range $\lvert\Delta\rvert_{p_e/p_w}$
SWG	5000	49	0.30	0.10	0.322 (0.003)	0.201 (0.005)	0.022 (0.003)	0.015 - 0.029	0 (0)	-26077.62 (99.44)	0.101 (0.005)	0.089 - 0.112
SWG	5000	49	0.30	0.20	0.325 (0.003)	0.217 (0.006)	0.025 (0.003)	0.019 - 0.031	0 (0)	-26033.52 (102.67)	0.017 (0.006)	0.005 - 0.029
SWG	5000	49	0.30	0.30	0.322 (0.003)	0.217 (0.006)	0.022 (0.003)	0.013 - 0.027	0 (0)	-26063.33 (98.53)	0.083 (0.006)	0.072 - 0.098
SWG	5000	49	0.30	0.40	0.322 (0.003)	0.222 (0.006)	0.022 (0.003)	0.014 - 0.029	0 (0)	-26024.2 (91.27)	0.178 (0.006)	0.166 - 0.195
SWG	5000	49	0.30	0.50	0.322 (0.003)	0.232 (0.007)	0.022 (0.003)	0.014 - 0.03	0 (0)	-26015.85 (93.25)	0.268 (0.007)	0.251 - 0.286
SWG	5000	49	0.30	0.60	0.312 (0.003)	0.2 (0.007)	0.012 (0.003)	0.005 - 0.019	0 (0)	-26044.14 (92.71)	0.4 (0.007)	0.385 - 0.413
SWG	5000	49	0.30	0.70	0.319 (0.003)	0.23 (0.007)	0.019 (0.003)	0.013 - 0.027	0 (0)	-26023.18 (107.76)	0.47 (0.007)	0.451 - 0.485
SWG	5000	49	0.30	0.80	0.311 (0.003)	0.197 (0.006)	0.011 (0.003)	0.004 - 0.019	0 (0)	-26067.97 (97.75)	0.603 (0.006)	0.589 - 0.617
SWG	5000	49	0.30	0.90	0.317 (0.003)	0.228 (0.008)	0.017 (0.003)	0.007 - 0.027	0 (0)	-26045.44 (103.72)	0.672 (0.008)	0.649 - 0.694
SWG	5000	49	0.40	0.10	0.405 (0.004)	0.096 (0.003)	0.005 (0.003)	0 - 0.015	0 (0)	-26569.88 (104.39)	0.004 (0.003)	0 - 0.012
SWG	5000	49	0.40	0.20	0.405 (0.004)	0.103 (0.003)	0.006 (0.003)	0 - 0.015	0 (0)	-26560.55 (98.09)	0.097 (0.003)	0.087 - 0.106
SWG	5000	49	0.40	0.30	0.402 (0.003)	0.101 (0.003)	0.003 (0.002)	0 - 0.009	0 (0)	-26580.89 (97.46)	0.199 (0.003)	0.191 - 0.206
SWG	5000	49	0.40	0.40	0.401 (0.004)	0.102 (0.003)	0.003 (0.002)	0 - 0.009	0 (0)	-26586.71 (104.14)	0.298 (0.003)	0.289 - 0.305
SWG	5000	49	0.40	0.50	0.4 (0.003)	0.105 (0.003)	0.003 (0.002)	0 - 0.008	0 (0)	-26577.5 (94.42)	0.395 (0.003)	0.388 - 0.401
SWG	5000	49	0.40	0.60	0.392 (0.003)	0.088 (0.003)	0.008 (0.003)	0 - 0.018	0 (0)	-26576.64 (96.62)	0.512 (0.003)	0.505 - 0.523
SWG	5000	49	0.40	0.70	0.396 (0.004)	0.101 (0.004)	0.005 (0.003)	0 - 0.012	0 (0)	-26593.47 (98.57)	0.599 (0.004)	0.588 - 0.61
SWG	5000	49	0.40	0.80	0.39 (0.003)	0.084 (0.003)	0.01 (0.003)	0.003 - 0.022	0 (0)	-26585.28 (104.17)	0.716 (0.003)	0.708 - 0.725
SWG	5000	49	0.40	0.90	0.39 (0.004)	0.096 (0.003)	0.01 (0.004)	0.002 - 0.018	0 (0)	-26581.5 (98.66)	0.804 (0.003)	0.796 - 0.811

Continued on next page

Table S1 – *Continued from previous page*

Structure	T	N	p	p_e/p_w	$\overline{\hat{p}}$ (SD)	$\overline{\hat{p_e}}/\overline{\hat{p_w}}$ (SD)	$\overline{\lvert\Delta\rvert}_p$ (SD)	Range $\lvert\Delta\rvert_p$	$\overline{SE}\lvert\Delta\rvert_p$ (SD)	$\overline{BIC}$ (SD)	$\overline{\lvert\Delta\rvert}_{p_e/p_w}$ (SD)	Range $\lvert\Delta\rvert_{p_e/p_w}$
SWG	5000	49	0.50	0.10	0.5 (0.003)	0.002 (0.001)	0.003 (0.002)	0 - 0.008	0 (0)	-26697.38 (105.14)	0.098 (0.001)	0.093 - 0.1
SWG	5000	49	0.50	0.20	0.5 (0.004)	0.002 (0.001)	0.003 (0.002)	0 - 0.008	0 (0)	-26678.43 (86.35)	0.198 (0.001)	0.194 - 0.2
SWG	5000	49	0.50	0.30	0.5 (0.004)	0.002 (0.001)	0.003 (0.002)	0 - 0.01	0 (0)	-26684.99 (104.91)	0.298 (0.001)	0.294 - 0.3
SWG	5000	49	0.50	0.40	0.5 (0.004)	0.002 (0.001)	0.003 (0.003)	0 - 0.01	0 (0)	-26659.48 (113.08)	0.398 (0.001)	0.394 - 0.4
SWG	5000	49	0.50	0.50	0.5 (0.004)	0.002 (0.001)	0.003 (0.003)	0 - 0.01	0 (0)	-26689.86 (104.33)	0.498 (0.001)	0.493 - 0.5
SWG	5000	49	0.50	0.60	0.5 (0.004)	0.002 (0.001)	0.003 (0.002)	0 - 0.01	0 (0)	-26708.15 (88.43)	0.598 (0.001)	0.593 - 0.6
SWG	5000	49	0.50	0.70	0.5 (0.004)	0.002 (0.001)	0.003 (0.003)	0 - 0.01	0 (0)	-26697.97 (89.84)	0.698 (0.001)	0.695 - 0.7
SWG	5000	49	0.50	0.80	0.5 (0.004)	0.002 (0.001)	0.003 (0.002)	0 - 0.01	0 (0)	-26691.44 (97.4)	0.798 (0.001)	0.794 - 0.8
SWG	5000	49	0.50	0.90	0.5 (0.004)	0.002 (0.001)	0.004 (0.002)	0 - 0.009	0 (0)	-26677.29 (113.17)	0.898 (0.001)	0.894 - 0.9
SWG	5000	49	0.60	0.10	0.61 (0.004)	0.094 (0.002)	0.01 (0.004)	0 - 0.019	0 (0)	-26587.63 (100.03)	0.006 (0.002)	0.001 - 0.011
SWG	5000	49	0.60	0.20	0.612 (0.004)	0.1 (0.002)	0.012 (0.004)	0.002 - 0.023	0 (0)	-26585.52 (107.54)	0.1 (0.002)	0.093 - 0.106
SWG	5000	49	0.60	0.30	0.615 (0.005)	0.099 (0.002)	0.015 (0.005)	0.005 - 0.026	0 (0)	-26583.91 (95.91)	0.201 (0.002)	0.195 - 0.205
SWG	5000	49	0.60	0.40	0.618 (0.004)	0.101 (0.002)	0.018 (0.004)	0.006 - 0.028	0 (0)	-26615.2 (92.32)	0.299 (0.002)	0.292 - 0.304
SWG	5000	49	0.60	0.50	0.622 (0.004)	0.103 (0.003)	0.022 (0.004)	0.013 - 0.032	0 (0)	-26586.69 (99.08)	0.397 (0.003)	0.39 - 0.404
SWG	5000	49	0.60	0.60	0.626 (0.004)	0.087 (0.002)	0.026 (0.004)	0.016 - 0.033	0 (0)	-26595.54 (87.47)	0.513 (0.002)	0.507 - 0.519
SWG	5000	49	0.60	0.70	0.627 (0.004)	0.1 (0.002)	0.027 (0.004)	0.016 - 0.037	0 (0)	-26624.13 (100.04)	0.6 (0.002)	0.593 - 0.606
SWG	5000	49	0.60	0.80	0.628 (0.004)	0.083 (0.002)	0.028 (0.004)	0.018 - 0.038	0 (0)	-26601.21 (90.61)	0.717 (0.002)	0.712 - 0.722
SWG	5000	49	0.60	0.90	0.633 (0.004)	0.093 (0.003)	0.033 (0.004)	0.022 - 0.043	0 (0)	-26625.03 (107.31)	0.807 (0.003)	0.801 - 0.814

Continued on next page

Table S1 – *Continued from previous page*

Structure	T	N	p	p_e/p_w	$\overline{\hat{p}}$ (SD)	$\overline{\hat{p_e}}/\overline{\hat{p_w}}$ (SD)	$\overline{\lvert\Delta\rvert}_p$ (SD)	Range $\lvert\Delta\rvert_p$	$\overline{SE}\lvert\Delta\rvert_p$ (SD)	$\overline{BIC}$ (SD)	$\overline{\lvert\Delta\rvert}_{p_e/p_w}$ (SD)	Range $\lvert\Delta\rvert_{p_e/p_w}$
SWG	5000	49	0.70	0.10	0.717 (0.004)	0.174 (0.002)	0.017 (0.004)	0.008 - 0.031	0 (0)	-26246.1 (97.11)	0.074 (0.002)	0.067 - 0.079
SWG	5000	49	0.70	0.20	0.721 (0.003)	0.188 (0.002)	0.021 (0.003)	0.013 - 0.029	0 (0)	-26280.11 (103.38)	0.012 (0.002)	0.006 - 0.018
SWG	5000	49	0.70	0.30	0.73 (0.003)	0.186 (0.002)	0.03 (0.003)	0.021 - 0.038	0 (0)	-26311.65 (92.83)	0.114 (0.002)	0.108 - 0.12
SWG	5000	49	0.70	0.40	0.736 (0.003)	0.191 (0.002)	0.036 (0.003)	0.029 - 0.044	0 (0)	-26323.91 (86.13)	0.209 (0.002)	0.204 - 0.215
SWG	5000	49	0.70	0.50	0.744 (0.003)	0.194 (0.002)	0.044 (0.003)	0.037 - 0.05	0 (0)	-26373.4 (83.31)	0.306 (0.002)	0.299 - 0.312
SWG	5000	49	0.70	0.60	0.747 (0.003)	0.161 (0.002)	0.047 (0.003)	0.039 - 0.052	0 (0)	-26364.91 (93.05)	0.439 (0.002)	0.431 - 0.445
SWG	5000	49	0.70	0.70	0.754 (0.003)	0.19 (0.002)	0.054 (0.003)	0.046 - 0.06	0 (0)	-26457.05 (104.15)	0.51 (0.002)	0.503 - 0.515
SWG	5000	49	0.70	0.80	0.75 (0.003)	0.154 (0.002)	0.05 (0.003)	0.044 - 0.056	0 (0)	-26384.94 (87.77)	0.646 (0.002)	0.64 - 0.653
SWG	5000	49	0.70	0.90	0.761 (0.003)	0.17 (0.002)	0.061 (0.003)	0.054 - 0.067	0 (0)	-26489.6 (105.29)	0.73 (0.002)	0.724 - 0.735
SWG	5000	49	0.80	0.10	0.809 (0.003)	0.216 (0.002)	0.009 (0.003)	0.002 - 0.014	0 (0)	-25840.7 (91.17)	0.116 (0.002)	0.111 - 0.121
SWG	5000	49	0.80	0.20	0.814 (0.003)	0.235 (0.002)	0.014 (0.003)	0.01 - 0.022	0 (0)	-25924.79 (107.71)	0.035 (0.002)	0.03 - 0.039
SWG	5000	49	0.80	0.30	0.826 (0.003)	0.224 (0.002)	0.026 (0.003)	0.019 - 0.031	0 (0)	-26126.35 (108.2)	0.076 (0.002)	0.072 - 0.082
SWG	5000	49	0.80	0.40	0.834 (0.002)	0.224 (0.002)	0.034 (0.002)	0.029 - 0.037	0 (0)	-26410.85 (126.63)	0.176 (0.002)	0.171 - 0.182
SWG	5000	49	0.80	0.50	0.841 (0.002)	0.215 (0.003)	0.041 (0.002)	0.038 - 0.046	0 (0)	-26613.02 (118.08)	0.285 (0.003)	0.279 - 0.294
SWG	5000	49	0.80	0.60	0.842 (0.001)	0.155 (0.003)	0.042 (0.001)	0.038 - 0.045	0 (0)	-26438.33 (110.02)	0.445 (0.003)	0.438 - 0.454
SWG	5000	49	0.80	0.70	0.848 (0.001)	0.186 (0.003)	0.048 (0.001)	0.045 - 0.051	0 (0)	-26871.45 (138.6)	0.514 (0.003)	0.505 - 0.52
SWG	5000	49	0.80	0.80	0.843 (0.001)	0.14 (0.003)	0.043 (0.001)	0.041 - 0.047	0 (0)	-26409.6 (122.75)	0.66 (0.003)	0.654 - 0.667
SWG	5000	49	0.80	0.90	0.848 (0.001)	0.135 (0.003)	0.048 (0.001)	0.046 - 0.05	0 (0)	-26595 (114.66)	0.765 (0.003)	0.759 - 0.772
SWG	5000	49	0.90	0.10	0.9 (0.001)	0.097 (0.004)	0.001 (0.001)	0 - 0.004	0 (0)	-25412.78 (237.36)	0.004 (0.003)	0 - 0.012

Continued on next page

Table S1 – *Continued from previous page*

Structure	T	N	p	p_e/p_w	$\overline{\hat{p}}$ (SD)	$\overline{\hat{p_e}}/\overline{\hat{p_w}}$ (SD)	$\overline{\lvert\Delta\rvert}_p$ (SD)	Range $\lvert\Delta\rvert_p$	$\overline{SE}\lvert\Delta\rvert_p$ (SD)	$\overline{BIC}$ (SD)	$\overline{\lvert\Delta\rvert}_{p_e/p_w}$ (SD)	Range $\lvert\Delta\rvert_{p_e/p_w}$
SWG	5000	49	0.90	0.20	0.902 (0.001)	0.101 (0.004)	0.002 (0.001)	0 - 0.006	0 (0)	-25697.7 (293.07)	0.099 (0.004)	0.086 - 0.11
SWG	5000	49	0.90	0.30	0.908 (0.001)	0.07 (0.003)	0.008 (0.001)	0.006 - 0.01	0 (0)	-23958.59 (232.99)	0.23 (0.003)	0.224 - 0.237
SWG	5000	49	0.90	0.40	0.909 (0.001)	0.057 (0.002)	0.009 (0.001)	0.006 - 0.011	0 (0)	-23447.23 (168.52)	0.343 (0.002)	0.338 - 0.348
SWG	5000	49	0.90	0.50	0.911 (0.001)	0.048 (0.001)	0.011 (0.001)	0.009 - 0.013	0 (0)	-23011.24 (150.3)	0.452 (0.001)	0.448 - 0.456
SWG	5000	49	0.90	0.60	0.91 (0.001)	0.028 (0.001)	0.01 (0.001)	0.008 - 0.012	0 (0)	-22260.79 (121.14)	0.572 (0.001)	0.569 - 0.576
SWG	5000	49	0.90	0.70	0.912 (0.001)	0.041 (0.002)	0.012 (0.001)	0.01 - 0.013	0 (0)	-22624.79 (129.35)	0.659 (0.002)	0.656 - 0.664
SWG	5000	49	0.90	0.80	0.91 (0.001)	0.025 (0.001)	0.01 (0.001)	0.009 - 0.012	0 (0)	-22184.18 (110.68)	0.775 (0.001)	0.772 - 0.778
SWG	5000	49	0.90	0.90	0.911 (0.001)	0.025 (0.001)	0.011 (0.001)	0.009 - 0.013	0 (0)	-22120.14 (127.96)	0.875 (0.001)	0.871 - 0.878
SWG	5000	100	0.10	0.10	0.109 (0.001)	0.161 (0.014)	0.009 (0.001)	0.006 - 0.014	0 (0)	-25230 (94.09)	0.061 (0.014)	0.035 - 0.108
SWG	5000	100	0.10	0.20	0.11 (0.002)	0.177 (0.015)	0.01 (0.002)	0.005 - 0.015	0 (0)	-25192.6 (96.29)	0.024 (0.015)	0 - 0.075
SWG	5000	100	0.10	0.30	0.11 (0.002)	0.18 (0.017)	0.01 (0.002)	0.002 - 0.014	0 (0)	-25178.2 (103.24)	0.12 (0.017)	0.083 - 0.193
SWG	5000	100	0.10	0.40	0.11 (0.001)	0.179 (0.014)	0.01 (0.001)	0.006 - 0.014	0 (0)	-25164.29 (105.75)	0.221 (0.014)	0.184 - 0.266
SWG	5000	100	0.10	0.50	0.109 (0.002)	0.176 (0.022)	0.009 (0.002)	0.002 - 0.014	0 (0)	-25170.16 (108.47)	0.324 (0.022)	0.284 - 0.407
SWG	5000	100	0.10	0.60	0.109 (0.004)	0.176 (0.036)	0.009 (0.004)	0.004 - 0.035	0 (0)	-25173.23 (110.96)	0.424 (0.036)	0.172 - 0.487
SWG	5000	100	0.10	0.70	0.108 (0.003)	0.163 (0.035)	0.008 (0.003)	0 - 0.016	0 (0)	-25178.52 (114.83)	0.537 (0.035)	0.468 - 0.636
SWG	5000	100	0.10	0.80	0.108 (0.004)	0.162 (0.046)	0.008 (0.004)	0 - 0.016	0 (0)	-25164.99 (105.32)	0.638 (0.046)	0.566 - 0.729
SWG	5000	100	0.10	0.90	0.108 (0.005)	0.161 (0.054)	0.008 (0.005)	0 - 0.016	0 (0)	-25169.36 (122.83)	0.739 (0.054)	0.659 - 0.846
SWG	5000	100	0.20	0.10	0.229 (0.002)	0.258 (0.01)	0.029 (0.002)	0.022 - 0.036	0 (0)	-28319.3 (84.39)	0.158 (0.01)	0.131 - 0.18
SWG	5000	100	0.20	0.20	0.232 (0.003)	0.289 (0.01)	0.032 (0.003)	0.025 - 0.039	0 (0)	-28250.84 (115.48)	0.089 (0.01)	0.059 - 0.115

Continued on next page

Table S1 – *Continued from previous page*

Structure	T	N	p	p_e/p_w	$\overline{\hat{p}}$ (SD)	$\overline{\hat{p}_e}/\overline{\hat{p}_w}$ (SD)	$\overline{\lvert\Delta\rvert}_p$ (SD)	Range $\lvert\Delta\rvert_p$	$\overline{SE}\lvert\Delta\rvert_p$ (SD)	$\overline{BIC}$ (SD)	$\overline{\lvert\Delta\rvert}_{p_e/p_w}$ (SD)	Range $\lvert\Delta\rvert_{p_e/p_w}$
SWG	5000	100	0.20	0.30	0.232 (0.003)	0.29 (0.012)	0.032 (0.003)	0.025 - 0.04	0 (0)	-28228.22 (96.47)	0.013 (0.009)	0 - 0.035
SWG	5000	100	0.20	0.40	0.231 (0.003)	0.288 (0.014)	0.031 (0.003)	0.018 - 0.038	0 (0)	-28243.27 (102.41)	0.112 (0.014)	0.081 - 0.163
SWG	5000	100	0.20	0.50	0.232 (0.004)	0.301 (0.016)	0.032 (0.004)	0.022 - 0.04	0 (0)	-28211.46 (116.15)	0.199 (0.016)	0.169 - 0.24
SWG	5000	100	0.20	0.60	0.231 (0.003)	0.299 (0.015)	0.031 (0.003)	0.022 - 0.038	0 (0)	-28181.04 (93.34)	0.301 (0.015)	0.269 - 0.339
SWG	5000	100	0.20	0.70	0.231 (0.004)	0.303 (0.018)	0.031 (0.004)	0.02 - 0.039	0 (0)	-28189.01 (103.81)	0.397 (0.018)	0.357 - 0.446
SWG	5000	100	0.20	0.80	0.232 (0.005)	0.315 (0.021)	0.032 (0.005)	0.023 - 0.04	0 (0)	-28153.16 (109.21)	0.485 (0.021)	0.45 - 0.524
SWG	5000	100	0.20	0.90	0.232 (0.005)	0.313 (0.021)	0.032 (0.005)	0.022 - 0.04	0 (0)	-28156.78 (98.24)	0.587 (0.021)	0.553 - 0.629
SWG	5000	100	0.30	0.10	0.326 (0.003)	0.194 (0.004)	0.026 (0.003)	0.02 - 0.031	0 (0)	-29661.22 (108.39)	0.094 (0.004)	0.085 - 0.103
SWG	5000	100	0.30	0.20	0.328 (0.003)	0.215 (0.005)	0.028 (0.003)	0.021 - 0.035	0 (0)	-29630.16 (90.87)	0.015 (0.005)	0.001 - 0.029
SWG	5000	100	0.30	0.30	0.326 (0.003)	0.211 (0.006)	0.026 (0.003)	0.016 - 0.032	0 (0)	-29584.79 (76.16)	0.089 (0.006)	0.076 - 0.108
SWG	5000	100	0.30	0.40	0.324 (0.003)	0.208 (0.006)	0.024 (0.003)	0.016 - 0.031	0 (0)	-29611.14 (98.72)	0.192 (0.006)	0.179 - 0.205
SWG	5000	100	0.30	0.50	0.323 (0.003)	0.215 (0.006)	0.023 (0.003)	0.015 - 0.029	0 (0)	-29623 (97.69)	0.285 (0.006)	0.273 - 0.303
SWG	5000	100	0.30	0.60	0.323 (0.003)	0.213 (0.006)	0.023 (0.003)	0.015 - 0.03	0 (0)	-29592.53 (96.33)	0.387 (0.006)	0.368 - 0.405
SWG	5000	100	0.30	0.70	0.322 (0.003)	0.216 (0.007)	0.022 (0.003)	0.015 - 0.029	0 (0)	-29607.57 (110.38)	0.484 (0.007)	0.47 - 0.498
SWG	5000	100	0.30	0.80	0.321 (0.003)	0.221 (0.007)	0.021 (0.003)	0.012 - 0.027	0 (0)	-29570.69 (101.09)	0.579 (0.007)	0.563 - 0.596
SWG	5000	100	0.30	0.90	0.319 (0.003)	0.216 (0.006)	0.019 (0.003)	0.013 - 0.027	0 (0)	-29607.39 (106.83)	0.684 (0.006)	0.669 - 0.698
SWG	5000	100	0.40	0.10	0.407 (0.003)	0.094 (0.003)	0.007 (0.003)	0 - 0.014	0 (0)	-30144.61 (93.26)	0.006 (0.003)	0.001 - 0.013
SWG	5000	100	0.40	0.20	0.407 (0.003)	0.102 (0.003)	0.007 (0.003)	0.001 - 0.015	0 (0)	-30145.98 (103.69)	0.098 (0.003)	0.093 - 0.105

Continued on next page

Table S1 – *Continued from previous page*

Structure	T	N	p	p_e/p_w	$\overline{\hat{p}}$ (SD)	$\overline{\hat{p_e}}/\overline{\hat{p_w}}$ (SD)	$\overline{\lvert\Delta\rvert}_p$ (SD)	Range $\lvert\Delta\rvert_p$	$\overline{SE}\lvert\Delta\rvert_p$ (SD)	$\overline{BIC}$ (SD)	$\overline{\lvert\Delta\rvert}_{p_e/p_w}$ (SD)	Range $\lvert\Delta\rvert_{p_e/p_w}$
SWG	5000	100	0.40	0.30	0.404 (0.003)	0.098 (0.002)	0.004 (0.003)	0 - 0.012	0 (0)	-30137 (88.91)	0.202 (0.002)	0.195 - 0.208
SWG	5000	100	0.40	0.40	0.403 (0.003)	0.096 (0.002)	0.003 (0.002)	0 - 0.011	0 (0)	-30161.02 (96.23)	0.304 (0.002)	0.296 - 0.309
SWG	5000	100	0.40	0.50	0.4 (0.004)	0.097 (0.003)	0.003 (0.002)	0 - 0.008	0 (0)	-30146.99 (90.59)	0.403 (0.003)	0.397 - 0.409
SWG	5000	100	0.40	0.60	0.4 (0.003)	0.096 (0.002)	0.003 (0.002)	0 - 0.007	0 (0)	-30122.65 (95.39)	0.504 (0.002)	0.498 - 0.51
SWG	5000	100	0.40	0.70	0.397 (0.003)	0.094 (0.002)	0.004 (0.002)	0 - 0.01	0 (0)	-30143.12 (88.64)	0.606 (0.002)	0.601 - 0.611
SWG	5000	100	0.40	0.80	0.395 (0.003)	0.095 (0.003)	0.005 (0.003)	0 - 0.012	0 (0)	-30127.7 (97.24)	0.705 (0.003)	0.698 - 0.71
SWG	5000	100	0.40	0.90	0.392 (0.003)	0.09 (0.003)	0.008 (0.003)	0 - 0.016	0 (0)	-30159.39 (102.91)	0.81 (0.003)	0.8 - 0.815
SWG	5000	100	0.50	0.10	0.501 (0.004)	0.001 (0.001)	0.003 (0.002)	0 - 0.009	0 (0)	-30252.11 (96.42)	0.099 (0.001)	0.096 - 0.1
SWG	5000	100	0.50	0.20	0.5 (0.004)	0.001 (0.001)	0.003 (0.002)	0 - 0.01	0 (0)	-30249.97 (93.74)	0.199 (0.001)	0.196 - 0.2
SWG	5000	100	0.50	0.30	0.5 (0.004)	0.001 (0.001)	0.003 (0.002)	0 - 0.009	0 (0)	-30260.33 (84.91)	0.299 (0.001)	0.295 - 0.3
SWG	5000	100	0.50	0.40	0.5 (0.004)	0.001 (0.001)	0.003 (0.002)	0 - 0.011	0 (0)	-30258.41 (93.82)	0.399 (0.001)	0.396 - 0.4
SWG	5000	100	0.50	0.50	0.5 (0.004)	0.001 (0.001)	0.003 (0.002)	0 - 0.011	0 (0)	-30257.79 (87.83)	0.499 (0.001)	0.496 - 0.5
SWG	5000	100	0.50	0.60	0.5 (0.004)	0.001 (0.001)	0.003 (0.002)	0 - 0.009	0 (0)	-30258 (101.45)	0.599 (0.001)	0.596 - 0.6
SWG	5000	100	0.50	0.70	0.5 (0.004)	0.001 (0.001)	0.003 (0.002)	0 - 0.014	0 (0)	-30247.73 (98.9)	0.699 (0.001)	0.696 - 0.7
SWG	5000	100	0.50	0.80	0.5 (0.003)	0.001 (0.001)	0.003 (0.002)	0 - 0.009	0 (0)	-30254.96 (90.11)	0.799 (0.001)	0.795 - 0.8
SWG	5000	100	0.50	0.90	0.5 (0.003)	0.001 (0.001)	0.003 (0.002)	0 - 0.008	0 (0)	-30264.61 (98.79)	0.899 (0.001)	0.896 - 0.9
SWG	5000	100	0.60	0.10	0.608 (0.004)	0.091 (0.002)	0.008 (0.004)	0 - 0.017	0 (0)	-30141.57 (102.77)	0.009 (0.002)	0.005 - 0.014
SWG	5000	100	0.60	0.20	0.612 (0.004)	0.1 (0.002)	0.012 (0.004)	0 - 0.023	0 (0)	-30144.44 (82.49)	0.1 (0.002)	0.097 - 0.104

Continued on next page

Table S1 – *Continued from previous page*

Structure	T	N	p	p_e/p_w	$\overline{\hat{p}}$ (SD)	$\overline{\hat{p}_e}/\overline{\hat{p}_w}$ (SD)	$\overline{\lvert\Delta\rvert}_p$ (SD)	Range $\lvert\Delta\rvert_p$	$\overline{SE}\lvert\Delta\rvert_p$ (SD)	$\overline{BIC}$ (SD)	$\overline{\lvert\Delta\rvert}_{p_e/p_w}$ (SD)	Range $\lvert\Delta\rvert_{p_e/p_w}$
SWG	5000	100	0.60	0.30	0.614 (0.003)	0.097 (0.002)	0.014 (0.003)	0.005 - 0.022	0 (0)	-30160.6 (92.43)	0.203 (0.002)	0.198 - 0.206
SWG	5000	100	0.60	0.40	0.616 (0.004)	0.094 (0.002)	0.016 (0.004)	0.003 - 0.024	0 (0)	-30145.18 (90.7)	0.306 (0.002)	0.301 - 0.31
SWG	5000	100	0.60	0.50	0.62 (0.003)	0.096 (0.002)	0.02 (0.003)	0.013 - 0.028	0 (0)	-30160.05 (108.67)	0.404 (0.002)	0.4 - 0.409
SWG	5000	100	0.60	0.60	0.621 (0.004)	0.095 (0.002)	0.021 (0.004)	0.012 - 0.031	0 (0)	-30150.11 (92.65)	0.505 (0.002)	0.502 - 0.511
SWG	5000	100	0.60	0.70	0.626 (0.004)	0.093 (0.002)	0.026 (0.004)	0.015 - 0.037	0 (0)	-30174.01 (93.02)	0.607 (0.002)	0.603 - 0.613
SWG	5000	100	0.60	0.80	0.629 (0.004)	0.093 (0.002)	0.029 (0.004)	0.021 - 0.041	0 (0)	-30164.69 (96.3)	0.707 (0.002)	0.702 - 0.712
SWG	5000	100	0.60	0.90	0.632 (0.004)	0.088 (0.002)	0.032 (0.004)	0.021 - 0.043	0 (0)	-30177.13 (114.71)	0.812 (0.002)	0.806 - 0.817
SWG	5000	100	0.70	0.10	0.715 (0.004)	0.169 (0.002)	0.015 (0.004)	0.003 - 0.023	0 (0)	-29781.55 (97.29)	0.069 (0.002)	0.065 - 0.075
SWG	5000	100	0.70	0.20	0.726 (0.003)	0.19 (0.002)	0.026 (0.003)	0.016 - 0.036	0 (0)	-29832.8 (90.08)	0.01 (0.002)	0.004 - 0.015
SWG	5000	100	0.70	0.30	0.729 (0.003)	0.186 (0.002)	0.029 (0.003)	0.022 - 0.037	0 (0)	-29832.57 (111.07)	0.114 (0.002)	0.109 - 0.12
SWG	5000	100	0.70	0.40	0.733 (0.003)	0.181 (0.002)	0.033 (0.003)	0.025 - 0.041	0 (0)	-29856.69 (86.12)	0.219 (0.002)	0.215 - 0.223
SWG	5000	100	0.70	0.50	0.741 (0.003)	0.184 (0.002)	0.041 (0.003)	0.034 - 0.048	0 (0)	-29932.5 (78.08)	0.316 (0.002)	0.312 - 0.32
SWG	5000	100	0.70	0.60	0.742 (0.003)	0.182 (0.002)	0.042 (0.003)	0.034 - 0.049	0 (0)	-29914.78 (90.74)	0.418 (0.002)	0.414 - 0.422
SWG	5000	100	0.70	0.70	0.753 (0.003)	0.179 (0.002)	0.053 (0.003)	0.047 - 0.061	0 (0)	-29986.02 (97.48)	0.521 (0.002)	0.516 - 0.525
SWG	5000	100	0.70	0.80	0.758 (0.003)	0.179 (0.002)	0.058 (0.003)	0.049 - 0.064	0 (0)	-29980.81 (107.44)	0.621 (0.002)	0.617 - 0.625
SWG	5000	100	0.70	0.90	0.762 (0.002)	0.166 (0.002)	0.062 (0.002)	0.056 - 0.068	0 (0)	-30006.7 (103.3)	0.734 (0.002)	0.73 - 0.738
SWG	5000	100	0.80	0.10	0.8 (0.002)	0.213 (0.002)	0.002 (0.001)	0 - 0.007	0 (0)	-29292.42 (84.26)	0.113 (0.002)	0.11 - 0.116
SWG	5000	100	0.80	0.20	0.821 (0.002)	0.243 (0.001)	0.021 (0.002)	0.015 - 0.025	0 (0)	-29545.89 (98.07)	0.043 (0.001)	0.039 - 0.047

Continued on next page

Table S1 – *Continued from previous page*

Structure	T	N	p	p_e/p_w	$\overline{\hat{p}}$ (SD)	$\overline{\hat{p}_e}/\overline{\hat{p}_w}$ (SD)	$\overline{\lvert\Delta\rvert}_p$ (SD)	Range $\lvert\Delta\rvert_p$	$\overline{SE}\lvert\Delta\rvert_p$ (SD)	$\overline{BIC}$ (SD)	$\overline{\lvert\Delta\rvert}_{p_e/p_w}$ (SD)	Range $\lvert\Delta\rvert_{p_e/p_w}$
SWG	5000	100	0.80	0.30	0.826 (0.002)	0.237 (0.001)	0.026 (0.002)	0.021 - 0.031	0 (0)	-29658.15 (113.81)	0.063 (0.001)	0.06 - 0.067
SWG	5000	100	0.80	0.40	0.831 (0.002)	0.224 (0.002)	0.031 (0.002)	0.026 - 0.035	0 (0)	-29888.99 (122.09)	0.176 (0.002)	0.173 - 0.181
SWG	5000	100	0.80	0.50	0.841 (0.001)	0.216 (0.002)	0.041 (0.001)	0.038 - 0.045	0 (0)	-30500.39 (145.29)	0.284 (0.002)	0.28 - 0.289
SWG	5000	100	0.80	0.60	0.841 (0.001)	0.21 (0.003)	0.041 (0.001)	0.037 - 0.044	0 (0)	-30509.88 (142.69)	0.39 (0.003)	0.383 - 0.397
SWG	5000	100	0.80	0.70	0.847 (0.001)	0.169 (0.003)	0.047 (0.001)	0.045 - 0.049	0 (0)	-30948.11 (143.32)	0.531 (0.003)	0.524 - 0.538
SWG	5000	100	0.80	0.80	0.849 (0.001)	0.153 (0.003)	0.049 (0.001)	0.047 - 0.05	0 (0)	-30813.06 (165.61)	0.647 (0.003)	0.64 - 0.655
SWG	5000	100	0.80	0.90	0.847 (0.001)	0.123 (0.002)	0.047 (0.001)	0.046 - 0.049	0 (0)	-30339.37 (146.24)	0.777 (0.002)	0.771 - 0.78
SWG	5000	100	0.90	0.10	0.889 (0.002)	0.107 (0.007)	0.011 (0.002)	0.008 - 0.017	0 (0)	-30856.55 (383.93)	0.008 (0.007)	0 - 0.027
SWG	5000	100	0.90	0.20	0.902 (0.001)	0.076 (0.002)	0.002 (0.001)	0 - 0.004	0 (0)	-28357.07 (272.65)	0.124 (0.002)	0.119 - 0.129
SWG	5000	100	0.90	0.30	0.905 (0.001)	0.062 (0.001)	0.005 (0.001)	0.004 - 0.007	0 (0)	-27577.59 (203.4)	0.238 (0.001)	0.234 - 0.241
SWG	5000	100	0.90	0.40	0.907 (0.001)	0.052 (0.001)	0.007 (0.001)	0.005 - 0.008	0 (0)	-26990.86 (158.64)	0.348 (0.001)	0.346 - 0.352
SWG	5000	100	0.90	0.50	0.909 (0.001)	0.045 (0.001)	0.009 (0.001)	0.007 - 0.01	0 (0)	-26657.25 (141.55)	0.455 (0.001)	0.452 - 0.457
SWG	5000	100	0.90	0.60	0.909 (0.001)	0.041 (0.001)	0.009 (0.001)	0.007 - 0.01	0 (0)	-26452.07 (125)	0.559 (0.001)	0.556 - 0.561
SWG	5000	100	0.90	0.70	0.909 (0)	0.033 (0.001)	0.009 (0)	0.008 - 0.011	0 (0)	-26102.23 (116.27)	0.667 (0.001)	0.664 - 0.668
SWG	5000	100	0.90	0.80	0.91 (0.001)	0.028 (0.001)	0.01 (0.001)	0.008 - 0.011	0 (0)	-25925.33 (118.49)	0.772 (0.001)	0.77 - 0.774
SWG	5000	100	0.90	0.90	0.909 (0.001)	0.022 (0.001)	0.009 (0.001)	0.008 - 0.01	0 (0)	-25731.61 (104.52)	0.878 (0.001)	0.876 - 0.881
